# Evaluation and comparison of statistical methods for early temporal detection of outbreaks: A simulation-based study

**DOI:** 10.1371/journal.pone.0181227

**Published:** 2017-07-17

**Authors:** Gabriel Bédubourg, Yann Le Strat

**Affiliations:** 1 CESPA, French Armed Forces Center for Epidemiology and Public Health, Marseille, France; 2 Aix Marseille Univ, INSERM, IRD, SESSTIM, Sciences Economiques & Sociales de la Santé & Traitement de l’Information Médicale, Marseille, France; 3 Santé publique France, French national public health agency, F-94415 Saint-Maurice, France; New York City Department of Health and Mental Hygiene, UNITED STATES

## Abstract

The objective of this paper is to evaluate a panel of statistical algorithms for temporal outbreak detection. Based on a large dataset of simulated weekly surveillance time series, we performed a systematic assessment of 21 statistical algorithms, 19 implemented in the R package surveillance and two other methods. We estimated false positive rate (FPR), probability of detection (POD), probability of detection during the first week, sensitivity, specificity, negative and positive predictive values and *F*_1_-measure for each detection method. Then, to identify the factors associated with these performance measures, we ran multivariate Poisson regression models adjusted for the characteristics of the simulated time series (trend, seasonality, dispersion, outbreak sizes, etc.). The FPR ranged from 0.7% to 59.9% and the POD from 43.3% to 88.7%. Some methods had a very high specificity, up to 99.4%, but a low sensitivity. Methods with a high sensitivity (up to 79.5%) had a low specificity. All methods had a high negative predictive value, over 94%, while positive predictive values ranged from 6.5% to 68.4%. Multivariate Poisson regression models showed that performance measures were strongly influenced by the characteristics of time series. Past or current outbreak size and duration strongly influenced detection performances.

## Introduction

Public health surveillance is the ongoing, systematic collection, analysis, interpretation, and dissemination of data for use in public health action to reduce morbidity and mortality of health-related events and to improve health [1]. One of the objectives of health surveillance is outbreak detection, which is crucial to enabling rapid investigation and implementation of control measures [2]. The threat of bioterrorism has stimulated interest in improving health surveillance systems for early detection of outbreaks [3, 4] as have natural disasters and humanitarian crises, such as earthquakes or the 2005 tsunami, and the recent emergence or reemergence of infectious diseases such as Middle East Respiratory Syndrome due to New Coronavirus (MERS-CoV) in 2012 [5] or Ebola in West Africa in 2014 [6].

Nowadays, a large number of surveillance systems are computer-supported. The computer support and statistical alarms are intended to improve outbreak detection for traditional or syndromic surveillance [7, 8]. These systems routinely monitor a large amount of data, recorded as time series of counts in a given geographic area for a given population. They produce statistical alarms that need to be confirmed by an epidemiologist, who determines if further investigation is needed. One limitation of these detection systems is an occasional lack of specificity, leading to false alarms that can overwhelm the epidemiologist with verification tasks [9, 10]. It is thus important to implement statistical methods that offer a good balance between sensitivity and specificity in order to detect a large majority of outbreaks without generating too many false positive alarms.

In the literature, a broad range of statistical methods has been proposed to detect outbreaks from surveillance data. The main statistical approaches have been reviewed by Shmueli et al. [11] and Unkel et al. [12]. By restricting these reviews to the methods that allow temporal detection of outbreaks without integrating the spatial distribution of cases, the general principle is to identify a time interval in which the observed number of cases of an event under surveillance (i.e. the number of reported cases) is significantly higher than expected. This identification is mainly based on a two-step process: First, an expected number of cases of the event of interest for the current time unit (generally a week or a day) is estimated and then compared to the observed value by a statistical test. A statistical alarm is triggered if the observed value is significantly different from the expected value. The main difference between statistical methods lies in how the expected value is estimated, which is most often done using statistical process control or regression techniques or combination of both [12].

A major constraint to the practical implementation of these methods is their capacity to be run on an increasing number of time series, provided by multiple sources of information, and centralized in large databases [3, 13, 14]. Monitoring a large number of polymorphic time series requires flexible statistical methods to deal with several well-known characteristics observed in time series: the frequency and variance of the number of cases, secular trend and one or more seasonality terms [14]. Even if some authors proposed to classify time series into a small number of categories and sought suitable algorithms for each category, in this automated and prospective framework, statistical methods cannot easily be fine tuned by choosing the most appropriate parameters adapted to each time series in an operational way, as explained by Farrington et al. [15].

A key question for public health practitioners is what method(s) can be adopted to detect the effects of unusual events on the data. Some authors have proposed a systematic assessment of the performances of certain methods in order to choose one reference algorithm [16–20]. They assessed these methods on a real dataset [16, 21], a simulated dataset [18–20, 22, 23] or on real time series for which simulated outbreaks were added [24, 25]. Simulating data offers the advantage of knowing the exact occurrence of the simulated outbreaks and their characteristics (amplitude, etc.). For example, Lotze et al. developed a simulated dataset of time series and outbreak signatures [26]. In the same way, Noufaily et al. [9] proposed a thorough simulation study to improve the Farrington algorithm [15]. Guillou et al. [27] compared the performance of their own algorithm to that of the improved Farrington, using the same simulated dataset. This dataset was also used by Salmon et al. to assess their method [28].

To our knowledge, no study has been proposed to thoroughly evaluate and compare the performance of a broad range of methods on a large simulated dataset.

The objective of this paper is to evaluate the performance of 21 statistical methods applied to large simulated datasets for outbreak detection in weekly health surveillance. The simulated dataset is presented in Section 2. The 21 evaluated methods and performance measures are described in Section 3. Evaluations and comparisons are presented in Section 4. A discussion follows in the last section.

## Materials

We simulated data following the approach proposed by Noufaily et al. [9].

First, simulated baseline data (i.e. time series of counts in the absence of outbreaks) were generated from a negative binomial model of mean *μ* and variance *ϕμ*, *ϕ* being the dispersion parameter ≥1. The mean at time *t*, *μ*(*t*), depends on a trend and seasonality modeled using Fourier terms:

$$\begin{array}{c} \log\left(\mu_{t}\right)=\theta +\beta t+\sum_{j=1}^{m}(\gamma_{1}\cos(\frac{2\pi jt}{52})+\gamma_{2}\sin(\frac{2\pi jt}{52})). \end{array}$$ (1)

Time series were simulated from 42 parameter combinations (called scenarios and presented in Table 1 in [9]) with different values taken by *θ*, *β*, *γ*_1_, *γ*_2_, *m* and *ϕ*, respectively associated with the baseline frequency of counts, trend, seasonality (no seasonality: *m* = 0, annual seasonality: *m* = 1, biannual seasonality: *m* = 2) and the dispersion parameter. For each scenario, 100 replicates of the baseline data (time series with 624 weeks) were generated. We thus obtained 42 × 100 = 4200 simulated time series. The last 49 weeks of each time series were named current weeks. The evaluated algorithms were run on these most recent 49 weeks. Performance measures described below were computed based on detection during these 49 weeks.

Secondly, for each time series, five outbreaks were simulated. Four outbreaks were generated in baseline weeks. Each outbreak started at a randomly drawn week and we generated the outbreak size (i.e. the number of outbreak cases) as Poisson with mean equal to a constant *k*_1_ times the standard deviation of the counts observed at the starting week. The fifth outbreak was generated in the current weeks in the same manner, using another constant noted *k*_2_. We chose the values of *k*_1_ to be 0, 2, 3, 5 and 10 in baseline weeks and *k*_2_ from 1 to 10 in current weeks as in [9].

Finally, outbreak cases were randomly distributed according to a lognormal distribution with mean 0 and standard deviation 0.5.

A total of 231,000 time series were generated from the 42 scenarios: 21,000 time series during the first step of simulation process (42 × 100 duplicates × 5 values for *k*_1_), and 210,000 time series during the second step of simulation process (21,000 × 10 values for *k*_2_), leading to a large simulated dataset including a great variety of time series, as observed in real surveillance data. At the end of the simulation process, 10,290,000 current weeks were generated, among which 6.2% were classified as outbreak weeks as they were included in an outbreak.

## Methods

### Statistical methods

We studied 21 statistical methods, 19 of which were implemented in the R package surveillance [29, 30]:

- the CDC algorithm [31].
- the RKI 1, 2 and 3 algorithms [29],
- the Bayes 1, 2 and 3 algorithms [29],
- CUSUM variants: original CUSUM [29, 32], a Rossi approximate CUSUM [32], a CUSUM algorithm for which the expected values are estimated by a GLM model [29], a mixed Rossi approximate CUSUM GLM algorithm [29],
- the original Farrington algorithm [15] and the improved Farrington algorithm [9],
- a count data regression chart (GLRNB) [29, 33] and a Poisson regression chart (GLR Poisson) [29, 34],
- the OutbreakP method [35],
- EARS C1, C2 and C3 algorithms [19, 36]

For all simulated time series, we used the tuning parameters recommended by their authors for each algorithm when available and proposed by default in the package surveillance. The commands used from the R package surveillance and the control tuning parameters chosen for these 19 algorithms are presented in Table 1.

**Table 1:** Commands, control tuning parameters and references of 19 algorithms implemented in the R package surveillance.

Method	Command	Control parameters	References
Improved Farrington	farringtonFlexible()	*b* = 5, *w* = 3, reweight = TRUE, weightsTreshold = 2.58, thresholdMethod = “nbPlugin”, *α*^1^	[9]
Original Farrington	algo.farrington()	*b* = 5, *w* = 3, reweight = TRUE, *α*^1^	[15]
CDC (historical limits)	algo.cdc()	*m* = 2, *b* = 4, *α*^1^	[31]
CUSUM	algo.cusum()	*k* = 1.04, *h* = 2.26, m = NULL, *α*^1^	[29, 32]
CUSUM Rossi	algo.cusum()	*k* = 1.04, *h* = 2.26, m = NULL, trans = “rossi”, *α*^1^	[29, 32]
CUSUM GLM	algo.cusum()	*k* = 1.04, *h* = 2.26, m = “glm”, *α*^1^	[29, 32]
CUSUM GLM Rossi	algo.cusum()	*k* = 1.04, *h* = 2.26, m = “glm”, trans = “rossi”, *α*^1^	[29, 32]
Bayes 1	algo.bayes1()	*α* = 0.05 (Package value)	[29]
Bayes 2	algo.bayes2()	*α* = 0.05 (Package value)	[29]
Bayes 3	algo.bayes3()	*α* = 0.05 (Package value)	[29]
RKI 1	algo.rki1()	-	[29]
RKI 2	algo.rki2()	-	[29]
RKI 3	algo.rki3()	-	[29]
GLR Negative Binomial	algo.glrnb()	ARL = 5, dir = “inc”	[29, 33]
GLR Poisson	algo.glrpois()	ARL = 5, dir = “inc”	[29, 34]
EARS C1	earsC()	method = “C1”, *α*^1^	[19, 36]
EARS C2	earsC()	method = “C2”, *α*^1^	[19, 36]
EARS C3	earsC()	method = “C3”, *α*^1^	[19, 36]
OutbreakP	algo.outbreakP()	*K* = 100, ret = c(“value”)	[35]

^1^ *α* = 0.001, 0.01 or 0.05

We also proposed two additional methods not implemented in the package surveillance:

- a periodic Poisson regression where *μ*(*t*) is defined as in Eq (1). The threshold is the 1 − *α* quantile of a Poisson distribution with mean equal to the predicted value at week *t*.
- a periodic negative binomial regression, also defined as in Eq (1), where the threshold is the 1 − *α* quantile of a negative binomial distribution with mean equal to the predicted value at week *t* and a dispersion parameter estimated by the model.

These last two models were run on all the historical data. An alarm was triggered if the observed number of cases was greater than the upper limit of the prediction interval. These two methods are basic periodic regressions. The R code of these two algorithms is presented in the S24 Appendix.

We evaluated the performances of the methods with three different *α* values: *α* = 0.001, *α* = 0.01 and *α* = 0.05.

### Performance measures

We considered eight measures to assess the performance of the methods:

- Measure 1 is false positive rate (FPR). For each method and each scenario, we calculated the FPR defined as the proportion of weeks corresponding to an alarm in the absence of an outbreak, as in [9]. Nominal FPRs were 0.0005 for analyses with *α* = 0.001, 0.005 for analyses with *α* = 0.01 or 0.025 for analyses with *α* = 0.05.
- Measure 2 is probability of detection (POD). For each scenario and for each current week period, if an alarm is generated at least once between the start and the end of an outbreak, the outbreak is considered to be detected [9]. POD is an event-based sensitivity (i.e. the entire outbreak interval is counted as a single observation for the sensitivity measurement) and is thus the proportion of outbreaks detected in 100 replicates.
- Measure 3 is probability of detection during the first week (POD1week), which makes it possible to evaluate the methods’ ability to enable early control measures.
- Measure 4 is observation-based sensitivity (Se): Outbreak weeks associated with an alarm were defined as True Positive (*TP*), non-outbreak weeks without alarm as True Negative (*TN*), outbreak weeks without alarm as False Negative (*FN*) and non-outbreak weeks with alarm as False Positive (*FP*). Thus, *Se* = *TP*/(*TP*+*FN*).
- Measure 5 is specificity (Sp) defined as *Sp* = *TN*/(*TN*+*FP*). Unlike FPR which was calculated on current weeks without any simulated outbreak, specificity was calculated on the entire number of current weeks out of the 210 000 time series including current outbreaks.
- Measure 6 is positive predictive value (PPV) defined as: *PPV* = *TP*/(*TP*+*FP*).
- Measure 7 is negative predictive value (NPV) defined as: *NPV* = *TN*/(*TN*+*FN*).
- Measure 8 is *F*_1_-measure defined as the harmonic mean of the sensitivity and the PPV: *F*_1_ = 2 × (*Se* × *PPV*)/(*Se* + *PPV*). *F*_1_-measure assumes values in the interval [0, 1] [37].

In the result section, we proposed to calculate averaged performance measures, i.e. to calculate FPR on the overall 21,000 time series without outbreak during the current weeks, and to calculate the other performance measures on the overall 210,000 time series with simulated outbreaks during the current weeks.

FPR was estimated prior to the simulation of current outbreaks, i.e. among the 49 current weeks for 21,000 (5 × 4,200) time series. Other indicators (POD, POD1week, Se, Sp, PPV, NPV) were estimated once outbreaks had been simulated, i.e. on the current weeks of all the time series (210,000 time series).

For each *α* value, we proposed ROC curve-like representation of these results with four plots representing sensitivity according to 1-specificity, POD and POD1week as functions of FPR, and sensitivity according to PPV.

### Factors associated with the performance measures

To identify the factors associated with the performance measures for *α* = 0.01 and assess the strength of associations, multivariate Poisson regression models [38] were run, as in Barboza et al. [39] or Buckeridge et al. [40]. A set of covariates corresponding to the characteristics of the simulated time series was included: trend (yes/no), seasonality (no/annual/biannual), the baseline frequency coefficient *θ*, the dispersion coefficient *ϕ* and *k*_1_ representing the amplitude and duration of past outbreaks. The last three covariates and *k*_2_ were treated as continuous and modeled using fractional polynomials. The statistical methods were introduced as covariates to estimate performance ratios, i.e. the ratios of performances of two methods, adjusted for the characteristics of the time series represented by the other covariates.

Adjusted FPR, POD, POD1week, sensitivity, and specificity ratios were estimated with the improved Farrington algorithm as reference. 95% confidence intervals were calculated with robust estimation of standard errors. For each continuous covariate modeled by fractional polynomials, ratios were presented for each value [41].

The simulation study, the implementation of the detection methods, and the estimations of performance were carried out using R (version 3.2.2), in particular using the package surveillance. Poisson regression models used to identify the factors associated with the performance measures and to assess the strength of associations were run using Stata 14.

## Results

### Averaged performances of the methods

In this section, we present the averaged performances of each evaluated method, i.e. the performances irrespective of the scenario and of the characteristics of the time series. Table 2 presents averaged FPR, specificity, POD, POD1week, sensitivity, negative predictive value, positive predictive value and *F*_1_-measure for all 42 scenarios and all past and current outbreak amplitude and duration and for *α* = 0.01. Overall, FPR ranged from 0.7% to 59.9% and POD from 43.3% to 88.7%. Methods with the highest specificity, such as the improved Farrington method or the periodic negative binomial regression, presented a POD lower than 45% and a sensitivity lower than 21%. Averaged measures for *α* = 0.001 and *α* = 0.05 are presented in S1 Table and S2 Table. RKI 1-3, GLR Negative Binomial, GLR Poisson, Bayes 1-3 and OutbreakP algorithms’ performances do not vary with *α* values (see Table 1). Their performances are only reported in Table 2. For each method, a radar chart presenting the measures 1-7 for *α* = 0.01 is proposed in the S23 Appendix.

**Table 2:** FPR, specificity, POD, POD1week, sensitivity, NPV, PPV and *F*_1_-measure for all 21 evaluated methods (for past outbreak constant *k*_1_ = 0, 2, 3, 5, 10 and current outbreak *k*_2_ = 1 to 10 for POD and sensitivity). *α* = 0.01 for Improved Farrington, Original Farrington, Periodic Poisson GLM and Neg Binomial GLM, CDC and EARS C1-C3. *α* = 0.05 for Bayes 1-3.

Method	FPR	Specificity	POD	POD1week	Sensitivity	NPV	PPV	*F*_1_-measure
Improved Farrington	1.0%	99.0%	43.3%	34.0%	20.5%	95.0%	58.3%	0.30
Original Farrington	2.3%	97.7%	56.9%	45.5%	29.0%	95.4%	45.0%	0.35
Periodic Poisson GLM	3.3%	96.8%	67.8%	56.6%	35.6%	95.8%	42.3%	0.39
Periodic Neg Binomial GLM	0.7%	99.4%	44.8%	36.3%	20.7%	95.0%	68.4%	0.32
CDC	3.6%	95.5%	45.0%	18.7%	34.2%	95.6%	33.2%	0.34
CUSUM	44.0%	52.7%	80.5%	70.5%	75.4%	97.0%	9.5%	0.17
CUSUM Rossi	39.5%	57.6%	77.0%	65.9%	71.8%	96.9%	10.1%	0.18
CUSUM GLM	44.2%	52.0%	84.4%	73.8%	79.5%	97.5%	9.9%	0.18
CUSUM GLM Rossi	39.9%	56.8%	81.1%	69.5%	76.1%	97.3%	10.4%	0.18
Bayes 1 (*α* = 0.05)	10.1%	90.5%	76.2%	66.2%	39.1%	95.7%	21.4%	0.28
Bayes 2 (*α* = 0.05)	9.4%	91.0%	80.8%	69.4%	45.7%	96.2%	25.0%	0.32
Bayes 3 (*α* = 0.05)	11.1%	88.9%	83.4%	71.9%	51.8%	96.5%	23.6%	0.32
RKI 1	8.3%	92.3%	67.8%	58.9%	30.4%	95.3%	20.6%	0.25
RKI 2	5.5%	94.7%	67.8%	57.8%	34.5%	95.6%	30.0%	0.32
RKI 3	7.0%	93.0%	71.3%	60.6%	41.8%	96.0%	28.3%	0.34
GLR Negative Binomial	4.3%	95.7%	50.8%	29.8%	21.6%	94.9%	24.9%	0.23
GLR Poisson	15.5%	84.5%	75.5%	60.3%	45.9%	95.9%	16.4%	0.24
EARS C1	6.9%	93.7%	66.3%	57.4%	25.6%	95.0%	21.2%	0.23
EARS C2	8.5%	92.4%	68.0%	57.1%	38.8%	95.8%	25.1%	0.31
EARS C3	7.4%	92.9%	54.2%	8.5%	35.3%	95.6%	24.7%	0.29
OutbreakP	59.9%	37.4%	70.4%	67.9%	66.1%	94.4%	6.5%	0.12

**Fig 1:**
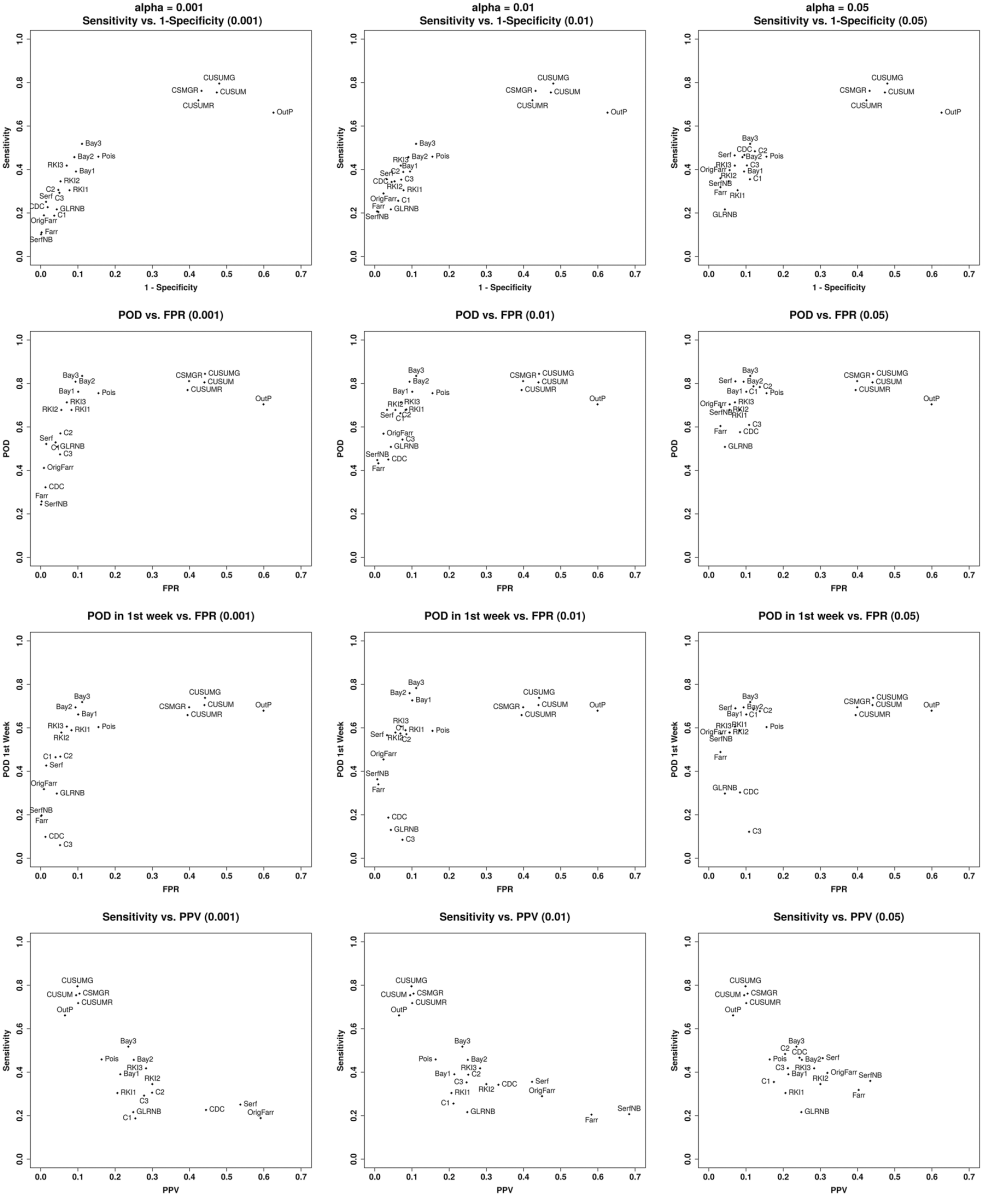
Sensitivity versus 1-specificity (line 1), POD versus FPR (line 2), POD1week versus FPR (line 3) and sensitivity versus PPV (line 4) for *α* = 0.001, 0.01 and 0.05 (columns 1-3). (Farr = Improved Farrington, OrigFarr = Original Farrington, Serf = periodic Poisson GLM, SerfNB = periodic Negative Binomial GLM, CDC = CDC algorithm, CUSUM = CUSUM, CUSUMR = CUSUM Rossi, CUSUMG = CUSUM GLM, CSMGR = CUSUM GLM Rossi, Bay1 = Bayes 1, Bay2 = Bayes 2, Bay3 = Bayes 3, RKI1 = RKI 1, RKI2 = RKI 2, RKI3 = RKI 3, Pois = GLR Poisson, GLRNB = GLR Negative Binomial, C1 = EARS C1, C2 = EARS C2, C3 = EARS C3, OutP = Outbreak P).

Fig 1 illustrates these results by plotting for the 21 methods the global results: sensitivity according to 1-specificity (line 1), POD according to FPR (line 2), POD1week according to FPR (line 3) and sensitivity according to PPV (line 4) for the 3 *α* values (columns 1-3). Two groups stand out from the rest. The first group consists of Bayes 1, 2 and 3. These methods present the best POD (around 0.8) and POD1week with a FPR around 10%. The second group consists of the 4 CUSUM methods: CUSUM, CUSUM Rossi, CUSUM GLM, and CUSUM GLM Rossi. For *α* = 0.01, these methods present the best sensitivity (around 0.80) but the lowest specificity (0.55) and the highest FPR (0.40). Note that while of the algorithm test statistics are based on the likelihood of single-week observations independent of recent ones, CuSUMs are not, and they may be important for applications where detection of gradual events rather than one-week spikes is especially critical. The OutbreakP method had the lowest specificity without having a better POD or POD1week than the first two groups. Finally, a third group consists of the other methods that had good specificity (over 0.9) but a lower sensitivity, POD and POD1week than the first two groups. All 21 methods presented a high negative predictive value, greater than 94%. The PPV of OutbreakP is very low (6.5%), while the Periodic Negative Binomial GLM method had the highest PPV (68.4%).

**Fig 2:**
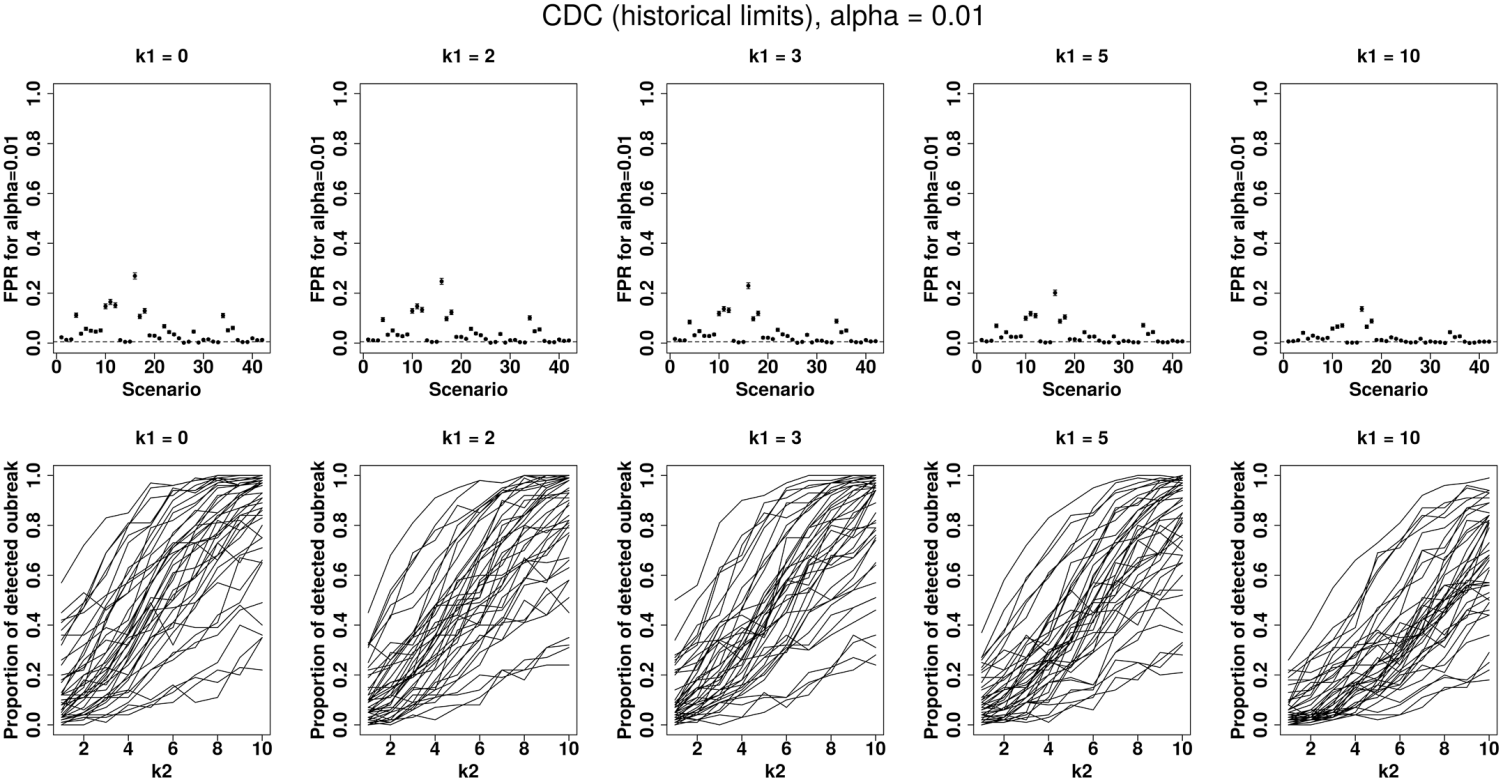
CDC algorithm performances for *α* = 0.01 by increasing past outbreak amplitude *k*_1_ = 0, 2, 3, 5 or 10 with (i) on the first row: false positive rate for 42 simulated scenarios, (ii) on the second row: probability of detection for 42 simulated scenarios (each curve corresponding to a scenario) by increasing current outbreak amplitude *k*_2_ = 1 to 10.

A first attempt to visualize certain differences is to plot POD and FPR according to the scenario and the *k*_1_ or *k*_2_ values. To illustrate this, Fig 2 shows the performances of the CDC method. The first row represents FPR for an increasing past outbreak constant *k*_1_ = 0, 2, 3, 5 and 10 according to the 42 scenarios. The second row shows POD according to *k*_2_ for the 42 scenarios (each curve corresponds to a simulated scenario) for an increasing past outbreak constant *k*_1_ = 0, 2, 3, 5 and 10. It clearly shows that performance depends on the scenario. The same plots with tables presenting numerical values for each method and different *α* values are presented in the S2 Appendix to S22 Appendix. To better compare the 21 methods, we presented on a single display in the S1 Appendix, their FPR according to the scenarios and their POD according to the *k*_2_ values for *k*_1_ = 5 and *α* = 0.01.

To better understand which characteristics are associated with each performance and to compare each method with the improved Farrington method, we present the results obtained from the multivariate Poisson regression models in the next section.

### Adjusted performance ratios and associated factors

Table 3 presents the adjusted performance ratios for performance measures 1 to 5 as described in the Methods’ section (*α* = 0.01 for Improved Farrington, Original Farrington, Periodic Poisson GLM and Neg Binomial GLM, CDC and EARS C1-C3. *α* = 0.05 for Bayes 1-3).

- Adjusted FPR ratios decreased when the amplitude and duration (driven by *k*_1_ in Eq (1)) of past outbreaks increased. It is indeed more difficult to detect an outbreak when past outbreaks have occurred, especially when these outbreaks are large and when the method does not under-weight their influence to estimate the expected number of cases. Adjusted FPR ratio was 2.75 times higher for time series with a secular trend than for the others. As we simulated time series with a non-negative trend (*β* ≥ 0 in Eq (1)), it was expected that FPR would decrease with a trend, especially for methods which do not integrate a trend in the estimation of the expected number of cases. In the same way, annual seasonality–and biannual seasonality to an even greater extent–and overdispersion increased FPR. We observed a nonlinear relation between FPR and baseline frequency: FPR ratio increased from the lowest frequencies to 12 cases per week, then decreased for the highest frequencies, with no clear explanation. Only periodic negative binomial GLM presented a FPR lower than improved Farrington FPR (FPR ratio = 0.71). Adjusted FPR ratios of OutbreakP and all CUSUM variants were higher than 40. Another group of methods all presented FPR ratios below 10: CDC, RKI variants, EARS methods, periodic Poisson GLM, original Farrington, Bayes 2 and GLR negative binomial. FPR ratios for other methods (Bayes 1 and 3, and GLR Poisson) were between 10 and 17.
- Adjusted specificity ratios were almost all equal to 1 as the amplitude and duration of past outbreaks had little influence on specificity. They were significantly lower for time series with a secular trend (adjusted specificity ratio = 0.84) or with annual or biannual seasonality (respective ratios: 0.99 and 0.98). Specificity decreased when dispersion increased but increased when the baseline frequency (*θ* in Eq (1)) increased. Only the periodic negative binomial GLM presented a specificity as good as that of the improved Farrington method (specificity ratio = 1.00).
- The adjusted POD ratios significantly decreased when past outbreak amplitude and duration (*k*_1_) increased, which is logical. They increased when current outbreak amplitude and duration (*k*_2_) increased, which is also normal. POD was higher for time series with secular trends which can be explained by the positive trend. POD decreased when there was an annual or a biannual seasonality (respective POD ratio = 0.97 and 0.92). Only the highest dispersion value (*θ* = 5) had an influence on POD (adjusted POD ratio = 1.09). Bayes 1, 2 and 3, CUSUM variants and the GLR Poisson method presented the highest POD ratios, from 1.75 (GLR Poisson) to 1.95 (CUSUM GLM). Any method was less able to detect an outbreak than the improved Farrington algorithm.
- POD1week presented results that were similar to those of POD. Adjusted POD1week ratios were significantly lower than those of POD for EARS C3 (0.25 versus 1.25), for CDC (0.55 versus 1.04) and for GLR negative binomial (1.17 versus 0.87). Other methods presented ratios for POD1week that were similar to or greater than those of POD.
- Finally, similar results were observed for sensitivity and for POD. Bayes 2 and 3 methods, OutbreakP, RKI 3, CUSUM variants and the GLR Poisson method presented the highest sensitivity ratios, from 2.04 (RKI 3) to 3.89 (CUSUM GLM). As observed in the POD model, any method was less able to detect an outbreak than the improved Farrington algorithm.

**Table 3:** Performance ratios with the improved Farrington method as reference, adjusted for past and current outbreaks (duration and amplitude), trend, seasonality, dispersion and baseline frequency (*α* = 0.01 for Improved Farrington, Original Farrington, Periodic Poisson GLM and Neg Binomial GLM, CDC and EARS C1-C3. *α* = 0.05 for Bayes 1-3).

Covariates	Categories/values	FPR ratio^⋆^ (CI 95%)	Specificity ratio^⋆^ (CI 95%)	POD ratio^⋆^ (CI 95%)	POD1week ratio^⋆^ (CI 95%)	Sensitivity ratio^⋆^ (CI 95%)
Methods	Improved Farrington	Ref (-)	Ref (-)	Ref (-)	Ref (-)	Ref (-)
	Original Farrington	2.43 (2.38 - 2.49)	0.99 (0.99 - 9.99)	1.32 (1.31 - 1.32)	1.34 (1.33 - 1.35)	1.42 (1.41 - 1.42)
	Periodic Poisson GLM	3.43 (3.35 - 3.50)	0.98 (0.98 - 0.98)	1.57 (1.56 - 1.58)	1.67 (1.66 - 1.68)	1.74 (1.73 - 1.75)
	Periodic Neg Binomial GLM	0.71 (0.68 - 0.73)	1.00 (1.00 - 1.00)	1.03 (1.03 - 1.04)	1.07 (1.06 - 1.08)	1.01 (1.00 - 1.02)
	CDC	3.79 (3.71 - 3.87)	0.96 (0.96 - 0.96)	1.04 (1.03 - 1.05)	0.55 (0.55 - 0.55)	1.67 (1.66 - 1.68)
	CUSUM	45.79 (44.90 - 46.70)	0.53 (0.53 - 0.53)	1.86 (1.85 - 1.87)	2.07 (2.06 - 2.08)	3.69 (3.67 - 3.71)
	CUSUM Rossi	41.08 (40.28 - 41.90)	0.58 (0.58 - 0.58)	1.78 (1.77 - 1.79)	1.94 (1.93 - 1.95	3.51 (3.49 - 3.53)
	CUSUM GLM	45.95 (45.06 - 46.87)	0.53 (0.52 - 0.53)	1.95 (1.94 - 1.96)	2.17 (2.16 - 2.18)	3.89 (3.87 - 3.91)
	CUSUM GLM Rossi	41.50 (40.69 - 42.32)	0.57 (0.57 - 0.57)	1.87 (1.87 - 1.88)	2.04 (2.03 - 2.05)	3.72 (3.70 - 3.74)
	Bayes 1	10.48 (10.27 - 10.70)	0.91 (0.91 - 0.91)	1.76 (1.75 - 1.77)	1.95 (1.93 - 1.96)	1.91 (1.90 - 1.92)
	Bayes 2	9.74 (9.54 - 9.94)	0.92 (0.92 - 0.92)	1.87 (1.86 - 1.88)	2.04 (2.03 - 2.05)	2.23 (2.22 - 2.24)
	Bayes 3	11.58 (11.35 - 11.82)	0.90 (0.90 - 0.90)	1.93 (1.92 - 1.94)	2.11 (2.10 - 2.13)	2.53 (2.52 - 2.55)
	RKI 1	8.60 (8.42 - 8.78)	0.93 (0.93 - 0.93)	1.57 (1.56 - 1.57)	1.73 (1.72 - 1.74)	1.49 (1.48 - 1.50)
	RKI 2	5.77 (5.65 - 5.89)	0.96 (0.96 - 0.96)	1.57 (1.56 - 1.58)	1.70 (1.69 - 1.71)	1.69 (1.68 - 1.70)
	RKI 3	7.30 (7.15 - 7.45)	0.94 (0.94 - 0.94)	1.65 (1.64 - 1.66)	1.78 (1.77 - 1.79)	2.04 (2.03 - 2.05)
	GLR Negative Binomial	4.49 (4.40 - 4.59)	0.97 (0.97 - 0.97)	1.17 (1.17 - 1.18)	0.87 (0.87 - 0.88)	1.06 (1.05 - 1.06)
	GLR Poisson	16.15 (15.83 - 16.47)	0.85 (0.85 - 0.85)	1.75 (1.74 - 1.75)	1.77 (1.76 - 1.78)	2.24 (2.23 - 2.25)
	EARS C1	7.16 (7.01 - 7.31)	0.95 (0.95 - 0.95)	1.54 (1.53 - 1.55)	1.69 (1.68 - 1.70)	1.25 (1.24 - 1.26)
	EARS C2	8.85 (8.67 - 9.04)	0.93 (0.93 - 0.93)	1.57 (1.56 - 1.58)	1.68 (1.67 - 1.69)	1.90 (1.89 - 1.91)
	EARS C3	7.74 (7.59 - 7.91)	0.94 (0.94 - 0.94)	1.25 (1.25 - 1.26)	0.25 (0.25 - 0.25)	1.73 (1.72 - 1.74)
	OutbreakP	62.32 (61.10 - 63.56)	0.38 (0.38 - 0.38)	1.63 (1.62 - 1.64)	2.00 (1.98 - 2.01)	3.23 (3.21 - 3.25)
*k*_1_	0	Ref (-)	Ref (-)	Ref (-)	Ref (-)	Ref (-)
	2	0.99 (0.98 - 0.99)	1.00 (1.00-1.00)	0.99 (0.99 - 0.99)	0.99 (0.99 - 0.99)	0.99 (0.99-0.99)
	3	0.98 (0.98 - 0.99)	1.00 (1.00-1.00)	0.98 (0.98 - 0.98)	0.98 (0.98 - 0.98)	0.98 (0.98-0.98)
	5	0.98 (0.97 - 0.98)	1.01 (1.01-1.01)	0.97 (0.97 - 0.97)	0.97 (0.97 - 0.97)	0.96 (0.96-0.97)
	10	0.96 (0.96 - 0.96)	1.01 (1.01-1.01)	0.94 (0.94 - 0.94)	0.93 (0.93 - 0.94)	0.93 (0.93-0.93)
*k*_2_	1	- -	Ref (-)	Ref (-)	Ref (-)	Ref (-)
	2	- -	1.00 (1.00 - 1.00)	1.32 (1.32 - 1.32)	1.30 (1.30 - 1.30)	1.23 (1.23 - 1.23)
	3	- -	1.00 (1.00 - 1.00)	1.63 (1.63 - 1.64)	1.64 (1.64 - 1.64)	1.47 (1.47 - 1.48)
	4	- -	1.00 (1.00 - 1.00)	1.93 (1.93 - 1.94)	2.01 (2.00 - 2.01)	1.73 (1.73 - 1.73)
	5	- -	1.00 (0.99 - 1.00)	2.22 (2.21 - 2.22)	2.39 (2.38 - 2.40)	1.99 (1.98 - 1.99)
	6	- -	0.99 (0.99 - 0.99)	2.47 (2.47 - 2.48)	2.76 (2.75 - 2.77)	2.23 (2.22 - 2.24)
	7	- -	0.99 (0.99 - 0.99)	2.69 (2.68 - 2.70)	3.10 (3.09 - 3.11)	2.44 (2.44 - 2.45)
	8	- -	0.99 (0.99 - 0.99)	2.85 (2.84 - 2.86)	3.37 (3.36 - 3.39)	2.62 (2.61 - 2.63)
	9	- -	0.99 (0.99 - 0.99)	2.95 (2.94 - 2.95)	3.57 (3.56 - 3.58)	2.75 (2.74 - 2.76)
	10	- -	0.99 (0.99 - 0.99)	2.96 (2.95 - 2.97)	3.67 (3.65 - 3.68)	2.82 (2.81 - 2.83)
Trend	No (*β* = 0)	Ref (-)	Ref (-)	Ref (-)	Ref (-)	Ref (-)
	Yes (*β* ≠ 0)	2.75 (2.74 - 2.76)	0.84 (0.84 - 0.84)	1.17 (1.16 - 1.17)	1.28 (1.28 - 1.28)	1.20 (1.20 - 1.20)
Seasonality (*m*)	No (*m* = 0)	Ref (-)	Ref (-)	Ref (-)	Ref (-)	Ref (-)
	Annual (*m* = 1)	1.06 (1.06 - 1.06)	0.99 (0.99 - 0.99)	0.97 (0.97 - 0.97)	0.98 (0.98 - 0.98)	0.97 (0.97 - 0.97)
	Biannual (*m* = 2)	1.13 (1.12 - 1.13)	0.98 (0.98 - 0.98)	0.92 (0.92 - 0.92)	0.93 (0.93 - 0.93)	0.92 (0.92 - 0.92)
Dispersion (*ϕ*)	1	Ref (-)	Ref (-)	Ref (-)	Ref (-)	Ref (-)
	1.1	1.02 (1.02 - 1.02)	1.00 (1.00 - 1.00)	1.00 (1.00 - 1.00)	1.00 (1.00 - 1.00)	1.00 (1.00 - 1.00)
	1.2	1.04 (1.04 - 1.04)	1.00 (1.00 - 1.00)	1.00 (1.00 - 1.00)	1.00 (1.00 - 1.00)	0.99 (0.99 - 0.99)
	1.5	1.07 (1.06 - 1.07)	0.99 (0.99 - 0.99)	0.99 (0.99 - 1.00)	1.00 (1.00 - 1.00)	0.98 (0.98 - 0.98)
	2	1.08 (1.08 - 1.08)	0.99 (0.99 - 0.99)	0.99 (0.99 - 0.99)	1.00 (1.00 - 1.00)	0.98 (0.97 - 0.98)
	3	1.08 (1.08 - 1.08)	0.98 (0.98 - 0.98)	0.98 (0.98 - 0.98)	1.01 (1.01 - 1.01)	0.98 (0.98 - 0.99)
	5	1.07 (1.07 - 1.08)	0.97 (0.97 - 0.97)	1.09 (1.09 - 1.09)	1.16 (1.16 - 1.17)	1.11 (1.11 - 1.11)
Frequency (*θ*)	-2 (0, 14 cases)	Ref (-)	Ref (-)	Ref (-)	Ref (-)	Ref (-)
	0.1 (1.1 cases)	1.14 (1.14 - 1.14)	0.99 (0.99 - 0.99)	1.01 (0.93 - 0.94)	1.03 (1.03 - 1.03)	0.95 (0.94 - 0.95)
	0.5 (1.65 cases)	1.18 (1.18 - 1.19)	0.98 (0.98 - 0.98)	1.01 (1.01 - 1.01)	1.04 (1.04 - 1.04)	0.94 (0.94 - 0.94)
	1.5 (4.48 cases)	1.27 (1.25 - 1.28)	0.97 (0.97 - 0.98)	1.02 (1.02 - 1.03)	1.07 (1.07 - 1.08)	0.92 (0.92 - 0.93)
	2.5 (12.18 cases)	1.22 (1.19 - 1.26)	0.98 (0.98 - 0.98)	1.03 (1.03 - 1.04)	1.10 (1.10 - 1.10)	0.90 (0.89 - 0.90)
	3.75 (42.52 cases)	0.88 (0.84 - 0.93)	1.02 (1.02 - 1.02)	1.04 (1.04 - 1.04)	1.10 (1.10 - 1.10)	0.84 (0.84 - 0.84)
	5 (148.41 cases)	0.38 (0.34 - 0.42)	1.13 (1.13 - 1.13)	1.03 (1.03 - 1.03)	1.04 (1.04 - 1.04)	0.77 (0.77 - 0.77)

^⋆^ Each ratio was statistically significant with *p* ≤ 10*e* − 3.

Estimation from the multivariate regression models to explain PPV and NPV are presented in S3 Table.

## Discussion

We presented a systematic assessment of the performance of 21 outbreak detection algorithms using a simulated dataset. One advantage of a simulation study for outbreak detection methods benchmarking is the a priori knowledge of the occurrence of outbreaks, which enables the developpment of a real “gold standard”. Some authors have already proposed that simulation studies be used to assess outbreak detection methods [18, 19, 23], and others have suggested adding simulated outbreaks to real surveillance data baselines [16, 24, 25], but without proposing a systematic assessment of the performance of a broad range of outbreak detection methods. Choi et al. [20] proposed such a study design based on the daily simulation method proposed by Hutwagner et al. [18] but do not study the influence of past outbreaks or time series characteristics (frequency, variance, secular trends, seasonalities, etc.), on methods performance.

The simulated dataset we used to perform our study is large enough to include the considerable diversity of time series observed in real surveillance systems. We also simulated a high diversity of outbreaks in terms of amplitude and duration. In our opinion, this simulated dataset presents a high representativeness of real weekly surveillance data. To extend our results to daily surveillance data, it should be necessary to perform a similar study with daily surveillance data. These characteristics of the simulated dataset enabled us to propose simple intrinsic performance indicator estimations such as FPR and POD and sensitivity and specificity to compare the performance of the evaluated methods. Furthermore, this allows us to compare our results to other studies based on the same dataset. Negative predictive value and positive predictive value are proposed as operational indicators for decision making when an alarm is triggered, or not triggered, by an algorithm. A benefit of the addition of outbreaks to the baseline weeks is that outlier removal strategies considered by many authors may be objectively tested and evaluated. One limitation in the simulation process was the fact that only increasing secular trends were used. Increasing secular trends would facilitate outbreak detection, while decreasing trends would hamper it. Furthermore, our study was designed based on weekly surveillance, while syndromic surveillance systems are most often daily systems. In daily surveillance time series, other seasonalities such as the “day of the week” effect need to be taken into account, which is not the case in our study.

The performance of the evaluated methods was only considered from a general perspective, in order to detect outbreaks in a large number of polymorphic weekly-based time series. In a pragmatic approach, it seems very difficult to adapt the tuning parameters of these methods for every time series. In France, public health agencies, such as the French National Public Health Agency (Santé publique France), the French Agency for Food, Environmental and Occupational Health Safety (Anses) and the French Armed Forces Center for Epidemiology and Public Health (CESPA) have deployed computer-supported outbreak detection systems in traditional or syndromic surveillance contexts [42–45]. They monitor a broad range of time series on a daily or weekly basis without, however, having rigorously evaluated the algorithms implemented. In the same way, the performance of the methods varied according to different baseline profiles depending on trend, seasonality, baseline frequency and overdispersion. Even if similar meta-models were already proposed by Buckeridge et al. for example [40], an original approach was to compare performance indicators adjusted for these parameters in a regression model. As expected, the adjusted performance of the 21 methods was penalized by increasing amplitude and duration in past outbreaks and by annual or biannual seasonality. Conversely, performance was better for increasing amplitude and duration in current outbreaks to be detected. More generally, the methods’ performance was highly dependent on simulation tuning parameters.

We proposed various measures to monitor the performance of outbreak detection methods. False positive rate (FPR) and probability of detection (POD) were proposed by Noufaily et al. [9]. We proposed an observation-based sensitivity measure and an event based sensitivity (POD). The concept of sensitivity based on alerting in each observation period is not applicable in some applications because signals of interest are intermittent and multimodal and may even be interpreted as multiple events. Many of the algorithms are based on the likelihood of single-week observations independent of recent ones, but CUSUMs are not, and the large sensitivity advantage in the CUSUMs methods, diminished for POD and POD1week, may be a result of the way the outbreak effects are modeled. By contrast, the implementation of the POD measure is uniformly applicable. Public health response to an outbreak depends on its early detection. In the POD definition, an outbreak was considered to be detected even if the first statistical alarm was issued during its last week. With the aim of estimating early detection performance, we also proposed POD during the first week, which cannot be considered alone, because even if it is done belatedly, an outbreak needs to be detected by the methods. While POD1week was an indicator of a method’s ability to detect an outbreak early, we did not propose any measure of timeliness like Salmon et al. [28] or Jiang et al. [45]. This topic could be further explored in another study. To give some insight on the speed of detection, we calculated it for the Improved Farrington algorithm and the CUSUM GLM Rossi algorithm. On average, on the overall dataset, it took 1.23 weeks for the Improved Farrincton method to detect an outbreak or 1.16 weeks for the CUSUM GLM Rossi method.

No method presented outbreak detection performances sufficient enough to provide reliable monitoring for a large surveillance system. Methods which provide high specificity or FPR, such as the improved Farrington or CDC algorithms, are not sensitive enough to detect the majority of outbreaks. These two algorithms could be implemented in systems that monitor health events to detect the largest outbreaks with the highest specificity.

Conversely, methods with the highest sensitivity and able to detect the majority of outbreaks–Bayes 3 or CUSUM GLM Rossi for example–produced an excessive number of false alarms, which could saturate a surveillance system and overhelm an epidemiologist in charge of outbreak investigations. As a screening test in clinical activity, the aim of an early outbreak detection method is to identify the largest possible number of outbreaks without producing too many false alarms.

The performances presented in this paper should be interpreted with caution as they depend both on tuning parameters and on the current implementation of the methods in the R packages. Packages evolve with time and their default parameters may also change. So this work based on R available packages, may be viewed as a starting point for researchers to enhance the comparison of methods and/or to optimize the tuning according to their data. Since no single algorithm presented sufficient performance for all scenarios, combinations of methods must be investigated to achieve predefined minimum performance. Other performance criteria should be proposed in order to improve the choice of algorithms to be implemented in surveillance systems. Therefore, we suggest that a study of the detection period between the first week of an outbreak and the first triggered alarm be conducted.

## Supporting information

S1 Appendix Comparison of the 21 evaluated methods (*α* = 0.01 for Improved Farrington, Original Farrington, Periodic Poisson GLM and Neg Binomial GLM, CDC and EARS C1-C3, *α* = 0.05 for Bayes 1-3, k1 = 5. (PDF)

S2 Appendix Overall performances of Improved Farrington algorithm (*α* = 0.001, 0.01 and 0.05). (PDF)

S3 Appendix Overall performances of Original Farrington algorithm (*α* = 0.001, 0.01 and 0.05). (PDF)

S4 Appendix Overall performances of Periodic Poisson GLM algorithm (*α* = 0.001, 0.01 and 0.05). (PDF)

S5 Appendix Overall performances of Periodic Negative Binomial GLM algorithm (*α* = 0.001, 0.01 and 0.05). (PDF)

S6 Appendix Overall performances of CDC algorithm (*α* = 0.001, 0.01 and 0.05). (PDF)

S7 Appendix Overall performances of CUSUM algorithm (*α* = 0.001, 0.01 and 0.05). (PDF)

S8 Appendix Overall performances of CUSUM Rossi algorithm (*α* = 0.001, 0.01 and 0.05). (PDF)

S9 Appendix Overall performances of CUSUM GLM algorithm (*α* = 0.001, 0.01 and 0.05). (PDF)

S10 Appendix Overall performances of CUSUM GLM Rossi algorithm (*α* = 0.001, 0.01 and 0.05). (PDF)

S11 Appendix Overall performances of Bayes 1 algorithm (*α* = 0.05). (PDF)

S12 Appendix Overall performances of Bayes 2 algorithm (*α* = 0.05). (PDF)

S13 Appendix Overall performances of Bayes 3 algorithm (*α* = 0.05). (PDF)

S14 Appendix Overall performances of RKI 1 algorithm. (PDF)

S15 Appendix Overall performances of RKI 2 algorithm. (PDF)

S16 Appendix Overall performances of RKI 3 algorithm. (PDF)

S17 Appendix Overall performances of GLR Negative Binomial algorithm. (PDF)

S18 Appendix Overall performances of GLR Poisson algorithm. (PDF)

S19 Appendix Overall performances of EARS C1 algorithm (*α* = 0.001, 0.01 and 0.05). (PDF)

S20 Appendix Overall performances of EARS C2 algorithm (*α* = 0.001, 0.01 and 0.05). (PDF)

S21 Appendix Overall performances of EARS C3 algorithm (*α* = 0.001, 0.01 and 0.05). (PDF)

S22 Appendix Overall performances of OutbreakP algorithm. (PDF)

S23 Appendix Radar charts of performances indicators: POD1week, POD, PPV, NPV, 1-FPR, Sp and Se for all 21 methods (*α* = 0.01 for Improved Farrington, Original Farrington, Periodic Poisson GLM and Neg Binomial GLM, CDC and EARS C1-C3. *α* = 0.05 for Bayes 1-3). (PDF)

S24 Appendix R code of periodic Poisson GLM algorithm and periodic negative binomial GLM algorithm. (PDF)

S1 Table FPR, specificity, POD, POD1week, sensitivity, negative predictive value, positive predictive value and *F*_1_-measure for 12 evaluated methods and *α* = 0.001 (for past outbreak constant *k*_1_ = 0, 2, 3, 5, 10 and current outbreak *k*_2_ = 1 to 10 for POD and sensitivity). (PDF)

S2 Table FPR, specificity, POD, POD1week, sensitivity, negative predictive value, positive predictive value and *F*_1_-measure for 15 evaluated methods and *α* = 0.05 (for past outbreak constant *k*_1_ = 0, 2, 3, 5, 10 and current outbreak *k*_2_ = 1 to 10 for POD and sensitivity). (PDF)

S3 Table Other performance ratios, adjusted on past and current outbreak duration and amplitude, trend, seasonality, dispersion and baseline frequency (*α* = 0.01 for Improved Farrington, Original Farrington, Periodic Poisson GLM and Neg Binomial GLM, CDC and EARS C1-C3. *α* = 0.05 for Bayes 1-3). (PDF)
