# Supplementary material for: Evaluation and comparison of statistical methods for early temporal detection of outbreaks: A simulation-based study
Source: PLoS One. 2017 Jul 17;12(7):e0181227. doi: 10.1371/journal.pone.0181227 (PMC5513450; doi:10.1371/journal.pone.0181227)

Evaluation and Comparison of Statistical Methods for Early Temporal Detection of Outbreaks: a Simulation-Based Study

Appendix S1: Comparison of the 21 evaluated methods ($\alpha = 0.01$ for Improved Farrington, Original Farrington, Periodic Poisson GLM and Neg Binomial GLM, CDC and EARS C1-C3, $\alpha = 0.05$ for Bayes 1-3, k1 = 5)

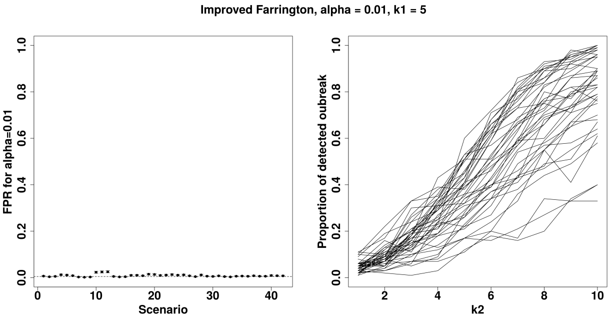

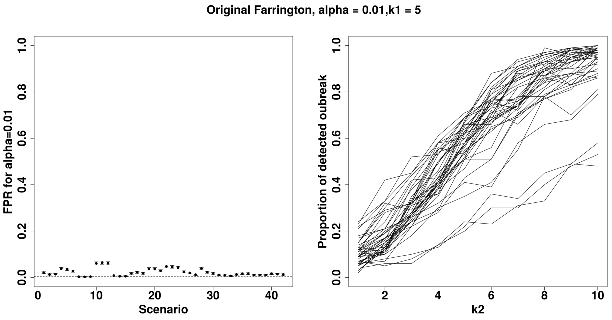

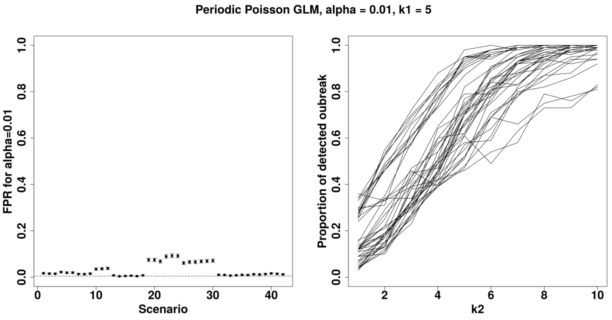

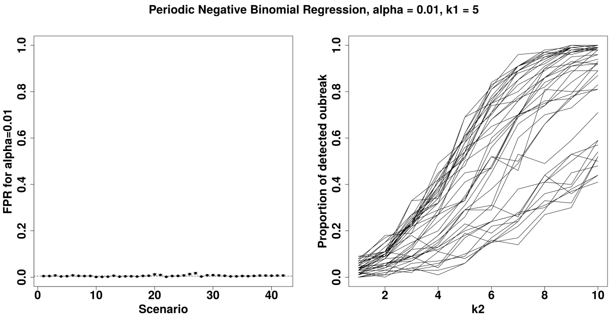

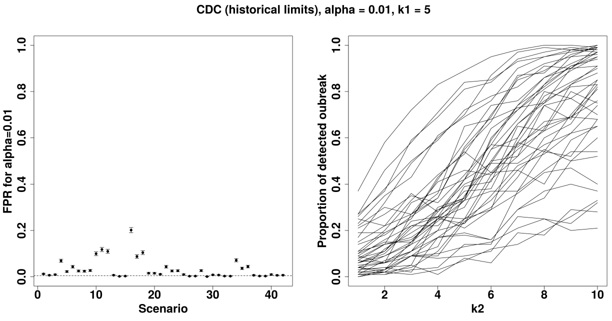

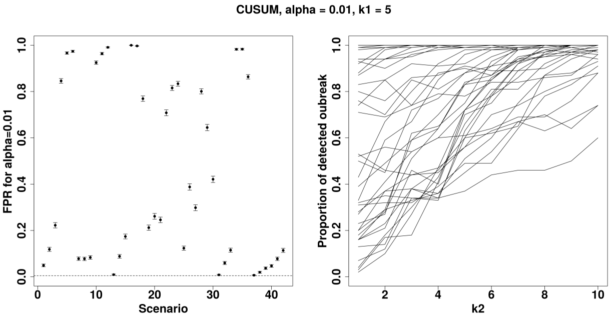

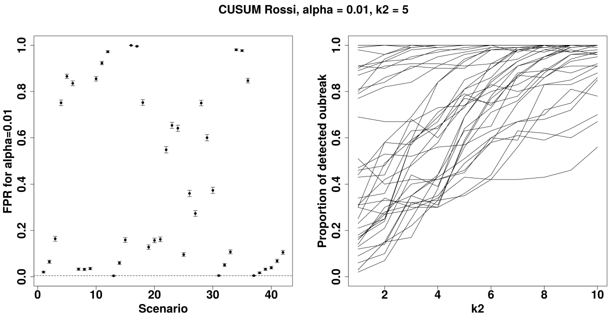

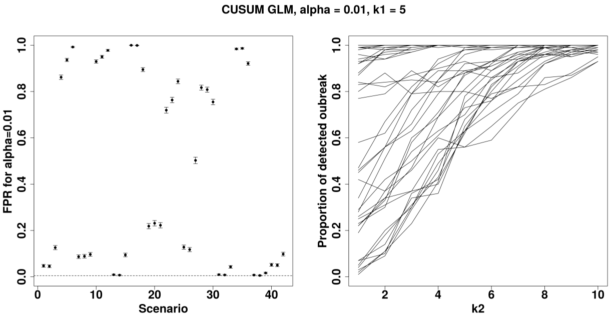

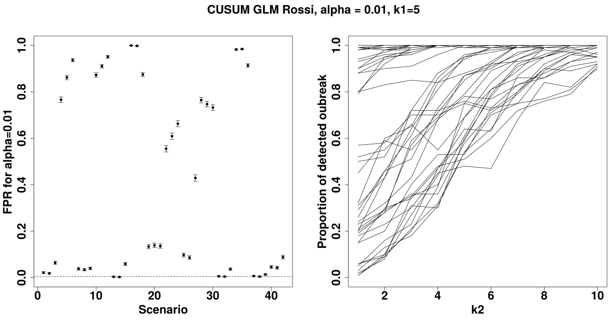

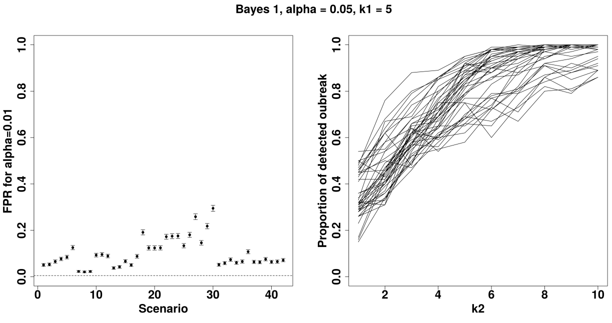

Bayes 2, alpha = 0.05, k1 = 5

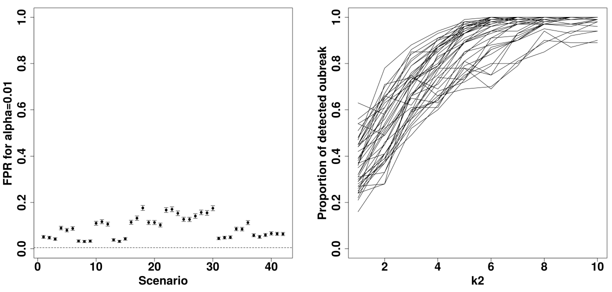

Bayes 3, alpha = 0.05, k1 = 5

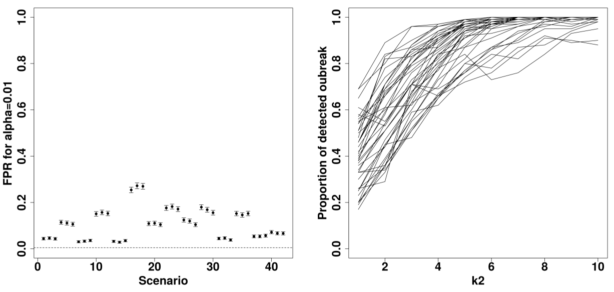

RKI 1, k1 = 5

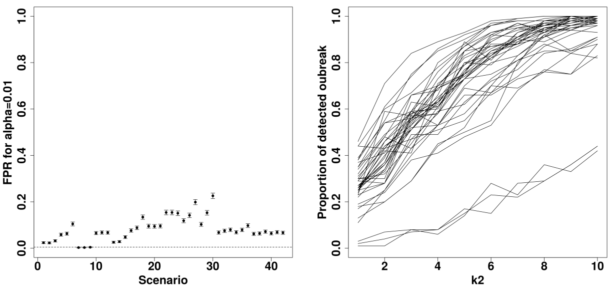

RKI 2, k1 = 5

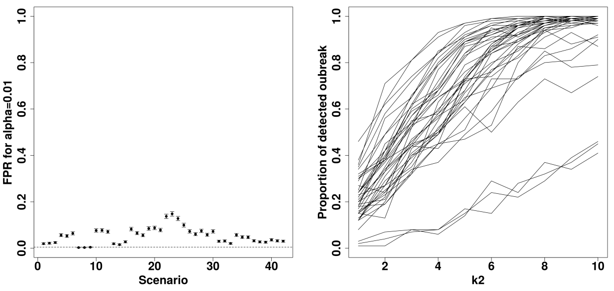

RKI 3, k1 = 5

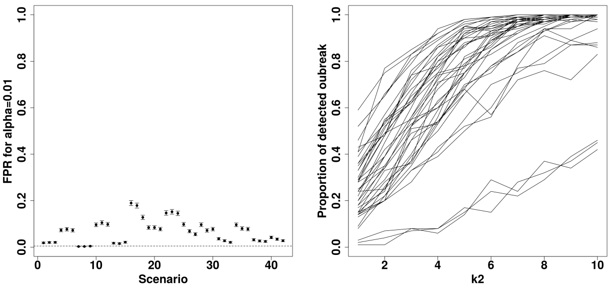

GLR Negative Binomiale, k1 = 5

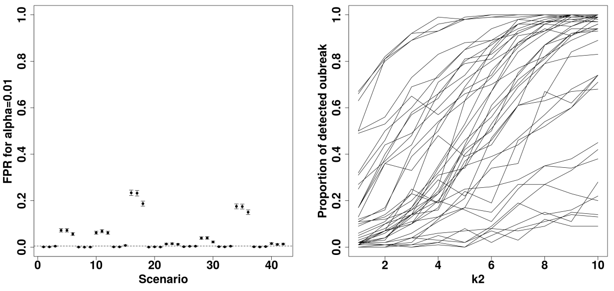

GLR Poisson, k1 = 5

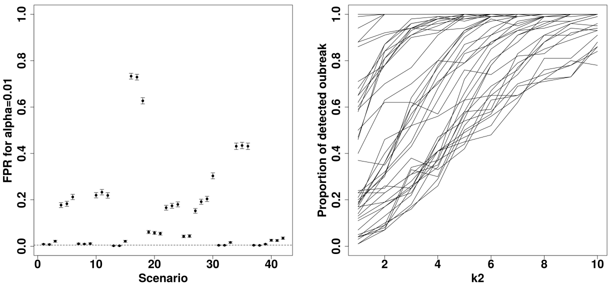

EARS C1, alpha = 0.01, k1 = 5

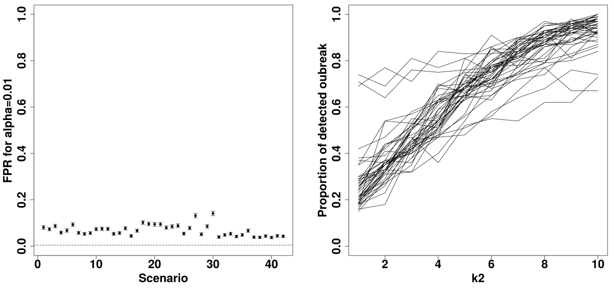

EARS C2, alpha = 0.01, k1 = 5

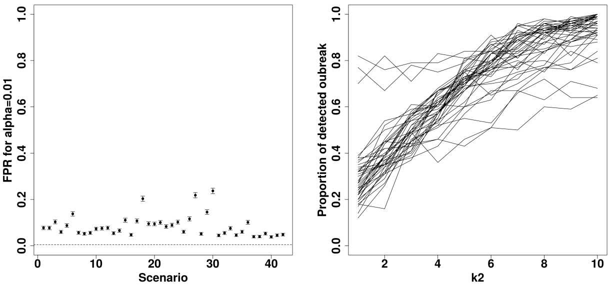

EARS C3, alpha = 0.01, k1 = 5

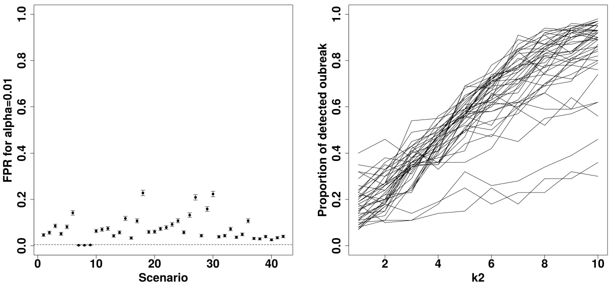

OutbreakP, k1 = 5

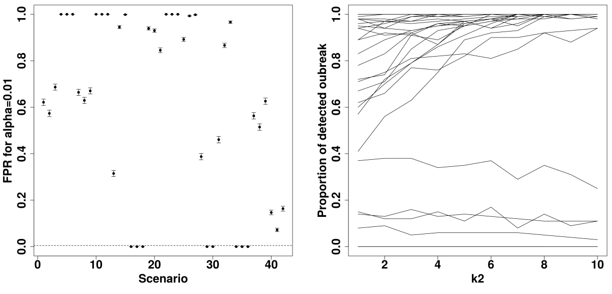

Supplement: S1 Appendix — (PDF) [file pone.0181227.s001.pdf]
