# Supplementary material for: Evaluation and comparison of statistical methods for early temporal detection of outbreaks: A simulation-based study
Source: PLoS One. 2017 Jul 17;12(7):e0181227. doi: 10.1371/journal.pone.0181227 (PMC5513450; doi:10.1371/journal.pone.0181227)
Supplement: S2 Appendix — (PDF) [file pone.0181227.s002.pdf]

Evaluation and Comparison of Statistical Methods for Early Temporal Detection of Outbreaks: a Simulation-Based Study

Appendix S2: Overall performances of Improved Farrington algorithm ($\alpha = 0.001, 0.01$ and 0.05)

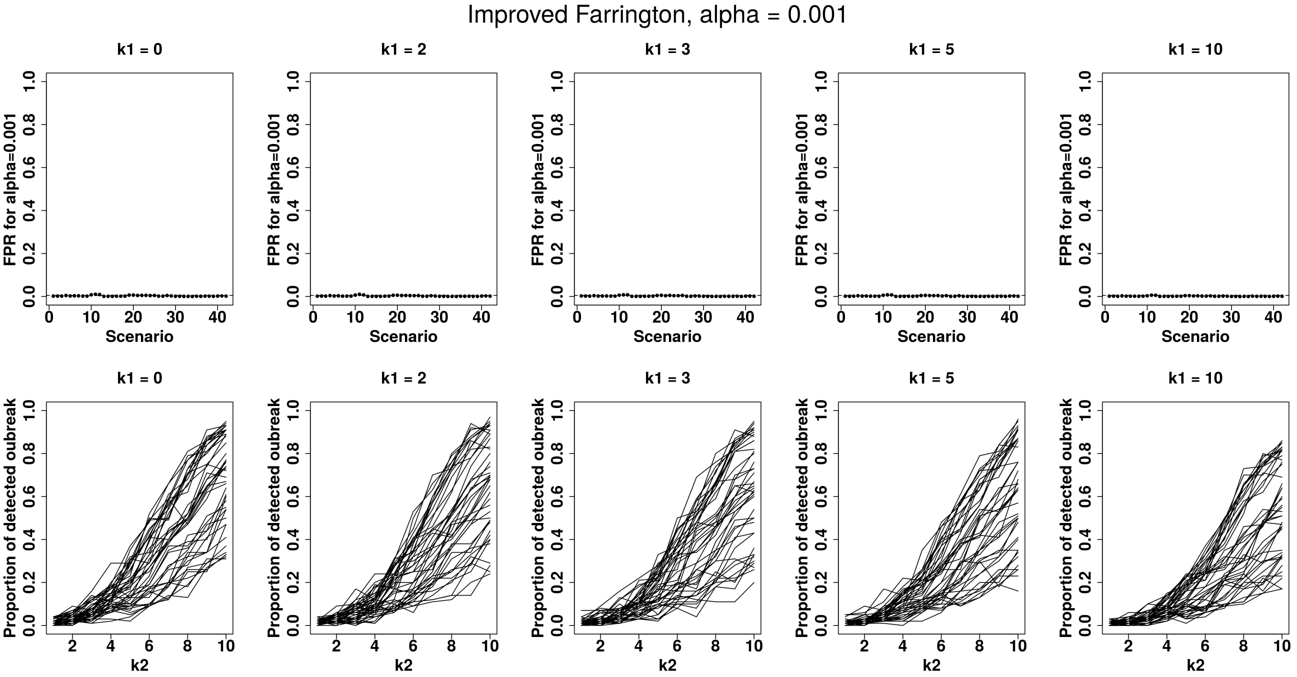

Figure 1: Improved Farrington algorithm performances for $\alpha = 0.001$ by increasing past outbreak amplitude $k_1 = 0$, 2, 3, 5 or 10 with (i) on the first row: false positive rate for 42 simulated scenarios, (ii) on the second row: probability of detection for 42 simulated scenarios (each curve corresponding to a scenario) by increasing current outbreak amplitude $k_2 = 1$ to 10.

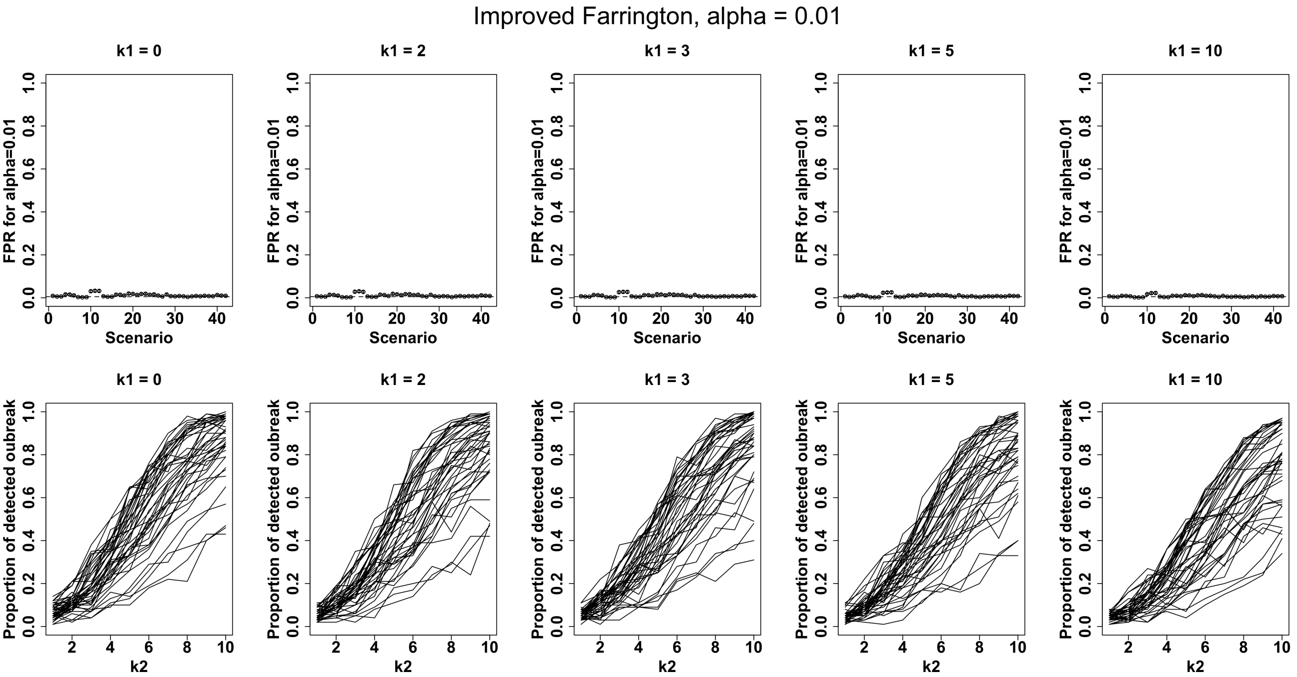

Figure 2: Improved Farrington algorithm performances for $\alpha = 0.01$ by increasing past outbreak amplitude $k_1 = 0$, 2, 3, 5 or 10 with (i) on the first row: false positive rate for 42 simulated scenarios, (ii) on the second row: probability of detection for 42 simulated scenarios (each curve corresponding to a scenario) by increasing current outbreak amplitude $k_2 = 1$ to 10.

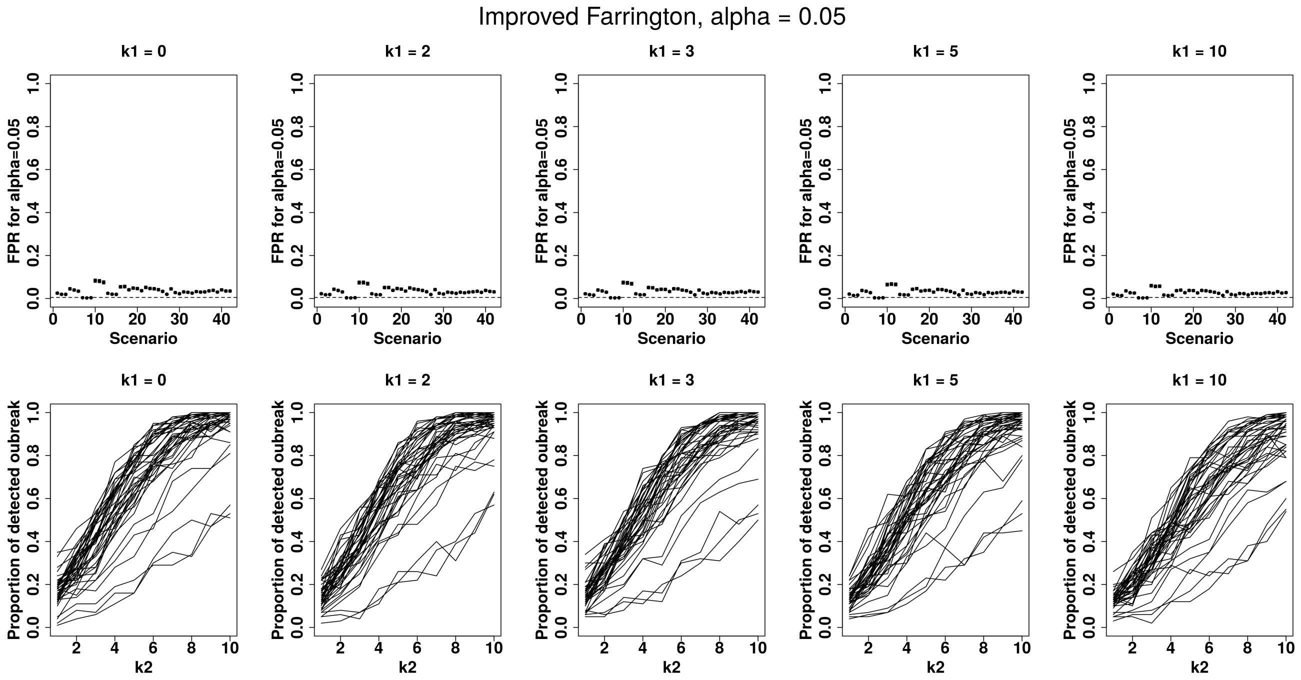

Figure 3: Improved Farrington algorithm performances for $\alpha = 0.05$ by increasing past outbreak amplitude $k_1 = 0, 2, 3, 5$ or 10 with (i) on the first row: false positive rate for 42 simulated scenarios, (ii) on the second row: probability of detection for 42 simulated scenarios (each curve corresponding to a scenario) by increasing current outbreak amplitude $k_2 = 1$ to 10.

Overall performances of Improved Farrington algorithm ($\alpha = 0.001$)

	FPR k1=0	FPR k1=2	FPR k1=3	FPR k1=5	FPR k1=10
1	0.00	0.00	0.00	0.00	0.00
2	0.00	0.00	0.00	0.00	0.00
3	0.00	0.00	0.00	0.00	0.00
4	0.00	0.00	0.00	0.00	0.00
5	0.00	0.00	0.00	0.00	0.00
6	0.00	0.00	0.00	0.00	0.00
7	0.00	0.00	0.00	0.00	0.00
8	0.00	0.00	0.00	0.00	0.00
9	0.00	0.00	0.00	0.00	0.00
10	0.01	0.01	0.01	0.01	0.00
11	0.01	0.01	0.01	0.01	0.01
12	0.01	0.01	0.01	0.01	0.01
13	0.00	0.00	0.00	0.00	0.00
14	0.00	0.00	0.00	0.00	0.00
15	0.00	0.00	0.00	0.00	0.00
16	0.00	0.00	0.00	0.00	0.00
17	0.00	0.00	0.00	0.00	0.00
18	0.00	0.00	0.00	0.00	0.00
19	0.01	0.01	0.00	0.00	0.00
20	0.01	0.01	0.01	0.01	0.00
21	0.00	0.00	0.00	0.00	0.00
22	0.01	0.00	0.01	0.00	0.00
23	0.01	0.00	0.00	0.00	0.00
24	0.00	0.00	0.00	0.00	0.00
25	0.00	0.00	0.00	0.00	0.00
26	0.00	0.00	0.00	0.00	0.00
27	0.00	0.00	0.00	0.00	0.00
28	0.00	0.00	0.00	0.00	0.00
29	0.00	0.00	0.00	0.00	0.00
30	0.00	0.00	0.00	0.00	0.00
31	0.00	0.00	0.00	0.00	0.00
32	0.00	0.00	0.00	0.00	0.00
33	0.00	0.00	0.00	0.00	0.00
34	0.00	0.00	0.00	0.00	0.00
35	0.00	0.00	0.00	0.00	0.00
36	0.00	0.00	0.00	0.00	0.00
37	0.00	0.00	0.00	0.00	0.00
38	0.00	0.00	0.00	0.00	0.00
39	0.00	0.00	0.00	0.00	0.00
40	0.00	0.00	0.00	0.00	0.00
41	0.00	0.00	0.00	0.00	0.00
42	0.00	0.00	0.00	0.00	0.00

Table 1: FPR according to each scenario and each k1 value, alpha = 0.001

	k2=1	k2=2	k2=3	k2=4	k2=5	k2=6	k2=7	k2=8	k2=9	k2=10
1	0.04	0.05	0.02	0.10	0.10	0.17	0.27	0.35	0.51	0.55
2	0.02	0.05	0.05	0.08	0.13	0.13	0.28	0.32	0.47	0.50
3	0.01	0.00	0.05	0.08	0.09	0.15	0.26	0.28	0.41	0.47
4	0.03	0.09	0.07	0.15	0.20	0.33	0.43	0.50	0.64	0.77
5	0.04	0.04	0.12	0.19	0.13	0.23	0.45	0.52	0.75	0.72
6	0.01	0.02	0.05	0.15	0.10	0.21	0.37	0.37	0.48	0.58
7	0.01	0.04	0.05	0.11	0.10	0.09	0.20	0.18	0.31	0.31
8	0.00	0.02	0.03	0.09	0.06	0.09	0.14	0.13	0.27	0.32
9	0.01	0.03	0.01	0.07	0.05	0.09	0.20	0.22	0.26	0.33
10	0.04	0.07	0.12	0.17	0.32	0.34	0.59	0.47	0.62	0.66
11	0.02	0.06	0.16	0.29	0.29	0.41	0.40	0.56	0.64	0.76
12	0.04	0.06	0.11	0.17	0.21	0.36	0.44	0.49	0.65	0.67
13	0.00	0.02	0.02	0.12	0.18	0.27	0.39	0.48	0.63	0.74
14	0.04	0.00	0.08	0.06	0.15	0.27	0.40	0.52	0.68	0.73
15	0.02	0.04	0.04	0.07	0.12	0.31	0.39	0.50	0.65	0.74
16	0.01	0.06	0.13	0.15	0.25	0.44	0.56	0.70	0.81	0.91
17	0.00	0.01	0.09	0.15	0.29	0.41	0.50	0.73	0.80	0.91
18	0.02	0.03	0.08	0.12	0.21	0.25	0.39	0.47	0.59	0.77
19	0.04	0.02	0.03	0.07	0.08	0.10	0.19	0.26	0.26	0.33
20	0.02	0.03	0.03	0.06	0.09	0.16	0.12	0.27	0.29	0.37
21	0.01	0.04	0.06	0.10	0.09	0.15	0.18	0.32	0.34	0.41
22	0.03	0.06	0.04	0.11	0.16	0.19	0.35	0.39	0.40	0.61
23	0.01	0.02	0.03	0.09	0.07	0.18	0.20	0.30	0.34	0.60
24	0.02	0.05	0.02	0.09	0.09	0.10	0.18	0.34	0.34	0.54
25	0.03	0.07	0.05	0.15	0.18	0.27	0.38	0.50	0.71	0.69
26	0.01	0.02	0.04	0.13	0.13	0.19	0.34	0.36	0.44	0.54
27	0.01	0.01	0.03	0.03	0.02	0.09	0.13	0.19	0.29	0.34
28	0.01	0.04	0.08	0.12	0.22	0.29	0.44	0.50	0.68	0.80
29	0.01	0.01	0.05	0.09	0.14	0.17	0.20	0.42	0.44	0.64
30	0.00	0.02	0.01	0.02	0.04	0.10	0.12	0.25	0.25	0.47
31	0.02	0.04	0.05	0.13	0.19	0.39	0.59	0.79	0.81	0.88
32	0.00	0.00	0.09	0.14	0.17	0.40	0.57	0.65	0.84	0.91
33	0.00	0.01	0.05	0.13	0.19	0.33	0.52	0.66	0.74	0.89
34	0.00	0.02	0.12	0.12	0.35	0.49	0.59	0.62	0.87	0.94
35	0.00	0.02	0.06	0.18	0.28	0.45	0.54	0.70	0.88	0.91
36	0.01	0.01	0.08	0.10	0.31	0.29	0.55	0.71	0.75	0.85
37	0.00	0.03	0.06	0.20	0.30	0.41	0.61	0.68	0.86	0.91
38	0.01	0.02	0.09	0.17	0.26	0.42	0.58	0.77	0.86	0.89
39	0.00	0.05	0.14	0.11	0.30	0.49	0.50	0.70	0.91	0.93
40	0.02	0.05	0.12	0.18	0.23	0.52	0.66	0.81	0.86	0.95
41	0.02	0.02	0.07	0.19	0.37	0.48	0.66	0.77	0.84	0.93
42	0.01	0.02	0.10	0.12	0.28	0.50	0.49	0.73	0.86	0.93

Table 2: POD according to each scenario and each k2 value, k1 = 0, alpha = 0.001

	k2=1	k2=2	k2=3	k2=4	k2=5	k2=6	k2=7	k2=8	k2=9	k2=10
1	0.01	0.05	0.06	0.05	0.17	0.15	0.25	0.47	0.49	0.50
2	0.01	0.06	0.05	0.08	0.16	0.16	0.19	0.34	0.34	0.49
3	0.00	0.02	0.02	0.10	0.06	0.17	0.23	0.25	0.33	0.40
4	0.00	0.06	0.10	0.09	0.29	0.30	0.36	0.52	0.63	0.70
5	0.02	0.05	0.03	0.19	0.17	0.32	0.37	0.48	0.60	0.68
6	0.01	0.01	0.08	0.08	0.15	0.17	0.36	0.40	0.52	0.56
7	0.02	0.02	0.01	0.05	0.07	0.11	0.12	0.14	0.29	0.25
8	0.03	0.03	0.03	0.01	0.09	0.11	0.12	0.14	0.14	0.28
9	0.00	0.02	0.01	0.02	0.12	0.09	0.11	0.12	0.20	0.24
10	0.02	0.04	0.10	0.20	0.24	0.40	0.44	0.52	0.68	0.71
11	0.04	0.05	0.17	0.12	0.31	0.34	0.43	0.41	0.58	0.69
12	0.02	0.09	0.10	0.15	0.18	0.35	0.45	0.42	0.52	0.68
13	0.01	0.01	0.03	0.10	0.23	0.25	0.41	0.54	0.67	0.74
14	0.02	0.01	0.04	0.06	0.17	0.20	0.38	0.51	0.63	0.76
15	0.01	0.01	0.01	0.12	0.22	0.25	0.26	0.48	0.52	0.65
16	0.01	0.04	0.12	0.15	0.24	0.41	0.46	0.62	0.86	0.82
17	0.02	0.02	0.12	0.24	0.24	0.32	0.50	0.69	0.82	0.83
18	0.00	0.04	0.03	0.08	0.20	0.20	0.30	0.42	0.53	0.70
19	0.03	0.02	0.05	0.04	0.06	0.14	0.20	0.26	0.28	0.44
20	0.03	0.05	0.06	0.06	0.08	0.13	0.22	0.22	0.29	0.42
21	0.02	0.00	0.02	0.09	0.07	0.09	0.14	0.21	0.25	0.40
22	0.01	0.05	0.03	0.04	0.07	0.20	0.28	0.36	0.47	0.53
23	0.03	0.04	0.07	0.12	0.10	0.06	0.20	0.38	0.38	0.48
24	0.02	0.02	0.07	0.07	0.09	0.11	0.20	0.25	0.37	0.49
25	0.00	0.02	0.03	0.07	0.22	0.25	0.33	0.44	0.64	0.69
26	0.01	0.02	0.05	0.08	0.11	0.14	0.29	0.37	0.49	0.59
27	0.00	0.02	0.00	0.07	0.08	0.15	0.16	0.20	0.32	0.29
28	0.00	0.02	0.09	0.06	0.23	0.28	0.36	0.53	0.68	0.80
29	0.02	0.01	0.02	0.01	0.10	0.15	0.23	0.36	0.47	0.62
30	0.00	0.02	0.00	0.04	0.07	0.12	0.18	0.31	0.36	0.38
31	0.03	0.03	0.03	0.13	0.23	0.40	0.57	0.72	0.83	0.94
32	0.00	0.05	0.10	0.12	0.27	0.39	0.51	0.63	0.78	0.91
33	0.00	0.00	0.10	0.08	0.21	0.28	0.45	0.52	0.74	0.87
34	0.01	0.04	0.07	0.18	0.23	0.34	0.60	0.76	0.91	0.93
35	0.01	0.05	0.06	0.09	0.28	0.38	0.60	0.73	0.94	0.89
36	0.00	0.04	0.06	0.10	0.12	0.38	0.57	0.74	0.78	0.89
37	0.02	0.01	0.07	0.13	0.29	0.40	0.54	0.72	0.83	0.97
38	0.00	0.04	0.09	0.18	0.18	0.50	0.66	0.76	0.87	0.94
39	0.01	0.04	0.08	0.11	0.27	0.43	0.58	0.80	0.88	0.95
40	0.00	0.05	0.08	0.13	0.30	0.53	0.63	0.79	0.87	0.93
41	0.02	0.03	0.09	0.14	0.31	0.45	0.70	0.73	0.87	0.93
42	0.04	0.01	0.03	0.17	0.28	0.35	0.57	0.74	0.92	0.91

Table 3: POD according to each scenario and each k2 value, k1 = 2, alpha = 0.001

	k2=1	k2=2	k2=3	k2=4	k2=5	k2=6	k2=7	k2=8	k2=9	k2=10
1	0.01	0.04	0.01	0.09	0.12	0.17	0.20	0.35	0.40	0.54
2	0.01	0.03	0.01	0.07	0.10	0.13	0.26	0.23	0.41	0.43
3	0.00	0.02	0.01	0.09	0.12	0.17	0.22	0.30	0.31	0.27
4	0.02	0.04	0.08	0.10	0.21	0.33	0.48	0.46	0.60	0.71
5	0.02	0.00	0.08	0.10	0.17	0.16	0.44	0.48	0.53	0.60
6	0.00	0.02	0.04	0.13	0.05	0.17	0.26	0.32	0.49	0.50
7	0.01	0.03	0.03	0.05	0.10	0.08	0.04	0.18	0.24	0.30
8	0.04	0.01	0.02	0.07	0.08	0.14	0.12	0.11	0.11	0.20
9	0.03	0.01	0.03	0.06	0.03	0.10	0.10	0.18	0.17	0.33
10	0.04	0.08	0.07	0.14	0.29	0.34	0.42	0.50	0.65	0.66
11	0.02	0.09	0.16	0.22	0.28	0.36	0.45	0.51	0.60	0.76
12	0.07	0.07	0.08	0.12	0.25	0.33	0.34	0.42	0.60	0.65
13	0.00	0.01	0.06	0.08	0.19	0.21	0.39	0.46	0.68	0.73
14	0.00	0.00	0.05	0.04	0.18	0.19	0.46	0.52	0.65	0.62
15	0.00	0.03	0.03	0.10	0.12	0.29	0.35	0.46	0.55	0.62
16	0.00	0.03	0.12	0.18	0.24	0.26	0.55	0.66	0.81	0.82
17	0.01	0.03	0.12	0.19	0.21	0.33	0.48	0.62	0.73	0.80
18	0.01	0.00	0.04	0.11	0.14	0.31	0.35	0.45	0.56	0.67
19	0.02	0.03	0.10	0.04	0.13	0.12	0.14	0.20	0.21	0.29
20	0.03	0.06	0.02	0.04	0.09	0.10	0.08	0.22	0.30	0.33
21	0.01	0.01	0.03	0.03	0.02	0.11	0.11	0.21	0.28	0.32
22	0.01	0.07	0.04	0.09	0.11	0.19	0.23	0.30	0.45	0.50
23	0.02	0.01	0.05	0.05	0.14	0.20	0.19	0.33	0.46	0.48
24	0.01	0.00	0.01	0.08	0.12	0.16	0.16	0.20	0.36	0.43
25	0.00	0.02	0.04	0.13	0.16	0.25	0.29	0.45	0.58	0.64
26	0.02	0.01	0.01	0.03	0.09	0.20	0.17	0.39	0.51	0.63
27	0.00	0.01	0.02	0.03	0.05	0.13	0.14	0.25	0.20	0.36
28	0.02	0.01	0.08	0.11	0.11	0.32	0.40	0.48	0.70	0.73
29	0.01	0.00	0.05	0.05	0.09	0.11	0.29	0.35	0.53	0.61
30	0.01	0.02	0.03	0.03	0.03	0.08	0.16	0.22	0.22	0.26
31	0.00	0.03	0.05	0.11	0.23	0.50	0.53	0.64	0.81	0.86
32	0.00	0.01	0.04	0.11	0.20	0.32	0.51	0.64	0.83	0.94
33	0.01	0.00	0.05	0.15	0.23	0.36	0.52	0.59	0.81	0.86
34	0.00	0.07	0.07	0.09	0.20	0.45	0.53	0.67	0.85	0.90
35	0.00	0.04	0.08	0.10	0.33	0.37	0.58	0.65	0.86	0.91
36	0.01	0.02	0.07	0.10	0.16	0.38	0.46	0.67	0.68	0.81
37	0.02	0.03	0.13	0.10	0.28	0.41	0.59	0.71	0.91	0.88
38	0.01	0.01	0.03	0.16	0.27	0.45	0.51	0.72	0.84	0.95
39	0.01	0.07	0.07	0.11	0.26	0.40	0.48	0.66	0.87	0.92
40	0.02	0.02	0.06	0.15	0.26	0.44	0.55	0.80	0.86	0.91
41	0.01	0.00	0.05	0.21	0.22	0.38	0.58	0.74	0.82	0.89
42	0.00	0.06	0.06	0.14	0.22	0.46	0.69	0.76	0.82	0.92

Table 4: POD according to each scenario and each k2 value, k1 = 3, alpha = 0.001

	k2=1	k2=2	k2=3	k2=4	k2=5	k2=6	k2=7	k2=8	k2=9	k2=10
1	0.01	0.01	0.03	0.09	0.12	0.14	0.30	0.29	0.32	0.50
2	0.00	0.01	0.03	0.09	0.11	0.12	0.21	0.30	0.35	0.35
3	0.02	0.01	0.02	0.04	0.10	0.13	0.19	0.24	0.30	0.40
4	0.00	0.03	0.04	0.16	0.19	0.24	0.39	0.54	0.53	0.64
5	0.02	0.03	0.07	0.12	0.15	0.26	0.26	0.51	0.54	0.63
6	0.00	0.03	0.04	0.08	0.10	0.17	0.29	0.33	0.43	0.50
7	0.02	0.03	0.03	0.06	0.08	0.10	0.10	0.13	0.23	0.23
8	0.01	0.01	0.01	0.00	0.06	0.12	0.09	0.13	0.19	0.26
9	0.01	0.02	0.02	0.05	0.06	0.15	0.09	0.15	0.19	0.16
10	0.05	0.05	0.17	0.14	0.20	0.29	0.40	0.43	0.53	0.63
11	0.05	0.06	0.11	0.22	0.21	0.27	0.36	0.51	0.54	0.57
12	0.04	0.09	0.07	0.15	0.21	0.28	0.38	0.42	0.41	0.68
13	0.00	0.02	0.09	0.11	0.14	0.28	0.31	0.50	0.64	0.66
14	0.01	0.03	0.05	0.06	0.19	0.25	0.38	0.52	0.62	0.76
15	0.02	0.02	0.04	0.10	0.12	0.15	0.21	0.37	0.53	0.72
16	0.02	0.03	0.05	0.14	0.23	0.37	0.45	0.68	0.74	0.87
17	0.00	0.02	0.08	0.15	0.25	0.40	0.45	0.56	0.76	0.87
18	0.00	0.02	0.05	0.06	0.11	0.20	0.37	0.42	0.52	0.63
19	0.02	0.03	0.03	0.03	0.08	0.13	0.12	0.20	0.20	0.35
20	0.01	0.00	0.04	0.07	0.09	0.08	0.11	0.17	0.26	0.26
21	0.03	0.01	0.02	0.03	0.08	0.07	0.13	0.16	0.24	0.32
22	0.01	0.05	0.02	0.06	0.08	0.16	0.22	0.31	0.34	0.48
23	0.03	0.01	0.04	0.03	0.09	0.16	0.20	0.35	0.44	0.51
24	0.01	0.03	0.04	0.08	0.09	0.15	0.16	0.24	0.32	0.41
25	0.03	0.01	0.06	0.07	0.12	0.20	0.28	0.49	0.58	0.66
26	0.01	0.03	0.03	0.04	0.10	0.10	0.25	0.35	0.41	0.50
27	0.00	0.02	0.02	0.02	0.08	0.08	0.16	0.30	0.18	0.28
28	0.01	0.04	0.05	0.12	0.12	0.27	0.38	0.41	0.61	0.76
29	0.01	0.03	0.02	0.02	0.07	0.13	0.29	0.35	0.48	0.52
30	0.00	0.01	0.02	0.02	0.02	0.06	0.16	0.17	0.23	0.34
31	0.02	0.01	0.02	0.13	0.18	0.30	0.52	0.65	0.78	0.87
32	0.01	0.01	0.04	0.15	0.25	0.39	0.44	0.56	0.80	0.91
33	0.00	0.03	0.06	0.11	0.16	0.34	0.50	0.58	0.74	0.76
34	0.00	0.01	0.04	0.12	0.17	0.37	0.58	0.72	0.79	0.87
35	0.01	0.01	0.06	0.08	0.23	0.46	0.53	0.63	0.84	0.83
36	0.00	0.02	0.07	0.15	0.20	0.26	0.43	0.66	0.69	0.86
37	0.02	0.00	0.09	0.18	0.26	0.40	0.57	0.72	0.73	0.91
38	0.02	0.05	0.06	0.09	0.35	0.46	0.58	0.70	0.84	0.95
39	0.02	0.02	0.08	0.11	0.22	0.41	0.50	0.68	0.74	0.92
40	0.01	0.03	0.07	0.17	0.19	0.43	0.61	0.79	0.81	0.93
41	0.00	0.02	0.07	0.14	0.26	0.48	0.56	0.73	0.80	0.91
42	0.00	0.00	0.06	0.16	0.23	0.32	0.58	0.63	0.78	0.96

Table 5: POD according to each scenario and each k2 value, k1 = 5, alpha = 0.001

	k2=1	k2=2	k2=3	k2=4	k2=5	k2=6	k2=7	k2=8	k2=9	k2=10
1	0.02	0.01	0.03	0.07	0.12	0.12	0.21	0.22	0.30	0.31
2	0.00	0.03	0.04	0.06	0.09	0.14	0.19	0.22	0.30	0.32
3	0.01	0.00	0.01	0.03	0.07	0.09	0.12	0.20	0.29	0.34
4	0.00	0.02	0.10	0.09	0.21	0.24	0.31	0.42	0.54	0.53
5	0.01	0.04	0.03	0.09	0.13	0.24	0.28	0.30	0.48	0.64
6	0.00	0.00	0.04	0.04	0.10	0.18	0.23	0.27	0.39	0.46
7	0.03	0.02	0.03	0.06	0.04	0.06	0.09	0.10	0.15	0.22
8	0.00	0.02	0.00	0.05	0.05	0.08	0.10	0.11	0.20	0.33
9	0.03	0.01	0.01	0.07	0.04	0.09	0.09	0.13	0.15	0.17
10	0.03	0.04	0.07	0.09	0.23	0.25	0.27	0.46	0.47	0.55
11	0.00	0.03	0.10	0.14	0.19	0.21	0.35	0.39	0.48	0.47
12	0.01	0.06	0.06	0.12	0.18	0.25	0.32	0.31	0.47	0.56
13	0.00	0.00	0.03	0.05	0.12	0.24	0.26	0.37	0.46	0.64
14	0.00	0.01	0.03	0.05	0.16	0.18	0.31	0.43	0.51	0.59
15	0.00	0.00	0.03	0.05	0.08	0.15	0.22	0.31	0.47	0.60
16	0.00	0.02	0.04	0.08	0.16	0.29	0.38	0.55	0.65	0.82
17	0.00	0.00	0.03	0.10	0.23	0.33	0.45	0.58	0.62	0.76
18	0.01	0.00	0.00	0.02	0.11	0.16	0.21	0.36	0.48	0.51
19	0.03	0.00	0.01	0.02	0.06	0.12	0.09	0.17	0.23	0.23
20	0.00	0.01	0.00	0.05	0.09	0.06	0.19	0.17	0.19	0.32
21	0.01	0.01	0.01	0.04	0.06	0.09	0.13	0.24	0.16	0.28
22	0.02	0.02	0.03	0.09	0.03	0.14	0.22	0.25	0.36	0.45
23	0.00	0.01	0.04	0.08	0.09	0.07	0.14	0.22	0.34	0.35
24	0.00	0.00	0.06	0.03	0.09	0.08	0.21	0.18	0.25	0.32
25	0.00	0.02	0.04	0.08	0.10	0.08	0.21	0.24	0.49	0.51
26	0.00	0.01	0.02	0.07	0.03	0.06	0.21	0.33	0.29	0.48
27	0.01	0.00	0.01	0.07	0.03	0.04	0.10	0.11	0.22	0.17
28	0.02	0.01	0.04	0.07	0.11	0.24	0.29	0.41	0.46	0.66
29	0.00	0.01	0.02	0.05	0.07	0.07	0.24	0.30	0.34	0.46
30	0.01	0.01	0.00	0.04	0.01	0.06	0.08	0.20	0.20	0.25
31	0.01	0.01	0.05	0.09	0.20	0.30	0.40	0.65	0.76	0.83
32	0.02	0.00	0.06	0.08	0.19	0.27	0.48	0.60	0.74	0.79
33	0.00	0.04	0.02	0.10	0.16	0.24	0.41	0.56	0.71	0.69
34	0.00	0.02	0.03	0.12	0.24	0.24	0.50	0.63	0.74	0.83
35	0.01	0.03	0.02	0.05	0.17	0.30	0.36	0.52	0.80	0.77
36	0.02	0.03	0.07	0.08	0.18	0.26	0.41	0.55	0.67	0.75
37	0.00	0.01	0.04	0.14	0.22	0.37	0.49	0.65	0.75	0.82
38	0.00	0.01	0.07	0.07	0.18	0.33	0.49	0.70	0.70	0.82
39	0.00	0.03	0.04	0.15	0.19	0.35	0.50	0.61	0.78	0.85
40	0.01	0.01	0.06	0.14	0.23	0.25	0.46	0.65	0.79	0.81
41	0.03	0.01	0.02	0.13	0.23	0.21	0.46	0.73	0.74	0.85
42	0.01	0.02	0.04	0.13	0.23	0.31	0.44	0.66	0.75	0.86

Table 6: POD according to each scenario and each k2 value, k1 = 10, alpha = 0.001

Overall performances of Improved Farrington algorithm ($\alpha = 0.01$)

	FPR k1=0	FPR k1=2	FPR k1=3	FPR k1=5	FPR k1=10
1	0.01	0.01	0.01	0.01	0.01
2	0.01	0.01	0.01	0.00	0.00
3	0.01	0.01	0.00	0.01	0.00
4	0.02	0.01	0.01	0.01	0.01
5	0.02	0.01	0.01	0.01	0.01
6	0.01	0.01	0.01	0.01	0.01
7	0.00	0.00	0.00	0.00	0.00
8	0.00	0.00	0.00	0.00	0.00
9	0.00	0.00	0.00	0.00	0.00
10	0.03	0.03	0.03	0.02	0.02
11	0.03	0.03	0.03	0.02	0.02
12	0.03	0.03	0.03	0.03	0.02
13	0.01	0.01	0.01	0.01	0.01
14	0.00	0.00	0.00	0.00	0.00
15	0.00	0.00	0.00	0.00	0.00
16	0.01	0.01	0.01	0.01	0.01
17	0.01	0.01	0.01	0.01	0.01
18	0.01	0.01	0.01	0.01	0.01
19	0.02	0.02	0.02	0.01	0.01
20	0.02	0.02	0.02	0.01	0.01
21	0.01	0.01	0.01	0.01	0.01
22	0.02	0.02	0.02	0.01	0.01
23	0.02	0.02	0.01	0.01	0.01
24	0.01	0.01	0.01	0.01	0.01
25	0.02	0.01	0.01	0.01	0.01
26	0.01	0.01	0.01	0.01	0.01
27	0.01	0.01	0.01	0.00	0.00
28	0.02	0.01	0.01	0.01	0.01
29	0.01	0.01	0.01	0.01	0.00
30	0.01	0.01	0.01	0.01	0.00
31	0.01	0.01	0.01	0.01	0.01
32	0.01	0.01	0.01	0.00	0.00
33	0.00	0.00	0.00	0.00	0.00
34	0.01	0.01	0.01	0.01	0.00
35	0.01	0.01	0.01	0.01	0.01
36	0.01	0.01	0.01	0.01	0.00
37	0.01	0.01	0.01	0.01	0.01
38	0.01	0.01	0.01	0.01	0.01
39	0.01	0.01	0.01	0.01	0.01
40	0.01	0.01	0.01	0.01	0.01
41	0.01	0.01	0.01	0.01	0.01
42	0.01	0.01	0.01	0.01	0.01

Table 7: FPR according to each scenario and each k1 value, alpha = 0.01

	k2=1	k2=2	k2=3	k2=4	k2=5	k2=6	k2=7	k2=8	k2=9	k2=10
1	0.10	0.13	0.17	0.29	0.33	0.38	0.55	0.69	0.78	0.82
2	0.05	0.10	0.10	0.30	0.33	0.47	0.57	0.65	0.72	0.84
3	0.06	0.03	0.09	0.18	0.26	0.32	0.47	0.54	0.67	0.73
4	0.09	0.21	0.21	0.35	0.42	0.58	0.75	0.83	0.82	0.92
5	0.08	0.08	0.27	0.39	0.45	0.49	0.67	0.76	0.96	0.91
6	0.10	0.10	0.13	0.31	0.33	0.49	0.57	0.59	0.77	0.79
7	0.03	0.07	0.07	0.16	0.14	0.19	0.24	0.31	0.43	0.43
8	0.01	0.03	0.04	0.10	0.10	0.18	0.22	0.21	0.40	0.46
9	0.01	0.06	0.04	0.11	0.13	0.23	0.34	0.37	0.40	0.47
10	0.12	0.21	0.30	0.40	0.59	0.62	0.80	0.78	0.81	0.84
11	0.12	0.19	0.38	0.50	0.65	0.64	0.75	0.77	0.85	0.96
12	0.14	0.19	0.35	0.36	0.51	0.61	0.71	0.78	0.81	0.87
13	0.02	0.10	0.11	0.27	0.43	0.53	0.67	0.76	0.90	0.90
14	0.08	0.10	0.18	0.22	0.42	0.59	0.68	0.78	0.86	0.91
15	0.09	0.11	0.12	0.19	0.34	0.54	0.63	0.72	0.84	0.91
16	0.09	0.21	0.25	0.36	0.56	0.75	0.80	0.90	0.95	0.98
17	0.08	0.14	0.33	0.41	0.53	0.73	0.86	0.89	0.91	0.96
18	0.12	0.14	0.20	0.35	0.39	0.47	0.66	0.73	0.80	0.88
19	0.09	0.08	0.16	0.21	0.29	0.36	0.49	0.66	0.69	0.70
20	0.06	0.08	0.12	0.24	0.26	0.48	0.52	0.57	0.64	0.74
21	0.04	0.12	0.14	0.23	0.32	0.37	0.50	0.61	0.68	0.79
22	0.04	0.10	0.16	0.29	0.36	0.41	0.64	0.77	0.75	0.85
23	0.05	0.07	0.18	0.22	0.37	0.46	0.59	0.71	0.73	0.86
24	0.08	0.16	0.10	0.24	0.37	0.38	0.48	0.67	0.80	0.85
25	0.07	0.12	0.17	0.32	0.45	0.62	0.76	0.79	0.95	0.93
26	0.03	0.09	0.18	0.25	0.37	0.44	0.57	0.73	0.78	0.84
27	0.05	0.02	0.07	0.13	0.16	0.26	0.37	0.49	0.54	0.57
28	0.10	0.12	0.16	0.28	0.40	0.66	0.76	0.80	0.90	0.97
29	0.05	0.10	0.07	0.26	0.33	0.50	0.58	0.75	0.86	0.88
30	0.02	0.09	0.08	0.09	0.18	0.29	0.30	0.44	0.53	0.65
31	0.07	0.11	0.19	0.31	0.51	0.72	0.84	0.94	0.95	0.97
32	0.08	0.07	0.29	0.38	0.48	0.70	0.78	0.92	0.95	0.99
33	0.04	0.08	0.19	0.31	0.48	0.58	0.80	0.88	0.93	0.98
34	0.02	0.11	0.25	0.37	0.64	0.70	0.80	0.90	0.97	0.98
35	0.08	0.08	0.19	0.41	0.45	0.72	0.81	0.93	0.97	0.98
36	0.06	0.13	0.20	0.30	0.65	0.67	0.81	0.92	0.93	0.97
37	0.04	0.11	0.19	0.45	0.50	0.64	0.84	0.90	0.97	1.00
38	0.05	0.12	0.30	0.41	0.57	0.68	0.88	0.98	0.95	0.98
39	0.04	0.14	0.34	0.35	0.55	0.75	0.72	0.91	0.99	0.97
40	0.11	0.14	0.31	0.50	0.57	0.76	0.90	0.96	0.97	1.00
41	0.11	0.11	0.25	0.40	0.64	0.73	0.85	0.95	0.96	0.99
42	0.06	0.10	0.24	0.34	0.53	0.72	0.83	0.92	0.99	0.98

Table 8: POD according to each scenario and each k2 value, k1 = 0, alpha = 0.01

	k2=1	k2=2	k2=3	k2=4	k2=5	k2=6	k2=7	k2=8	k2=9	k2=10
1	0.03	0.10	0.15	0.17	0.36	0.43	0.48	0.65	0.71	0.78
2	0.03	0.15	0.11	0.18	0.30	0.35	0.44	0.59	0.62	0.73
3	0.03	0.06	0.10	0.21	0.21	0.35	0.52	0.51	0.66	0.76
4	0.06	0.15	0.20	0.29	0.50	0.48	0.68	0.78	0.83	0.94
5	0.07	0.14	0.13	0.35	0.49	0.52	0.63	0.85	0.88	0.91
6	0.02	0.10	0.14	0.30	0.29	0.34	0.50	0.64	0.72	0.72
7	0.03	0.04	0.02	0.08	0.11	0.14	0.24	0.29	0.42	0.42
8	0.03	0.05	0.05	0.04	0.18	0.20	0.22	0.30	0.24	0.48
9	0.02	0.02	0.05	0.09	0.15	0.17	0.28	0.25	0.36	0.47
10	0.07	0.18	0.29	0.39	0.54	0.61	0.66	0.78	0.87	0.86
11	0.09	0.17	0.35	0.37	0.50	0.61	0.69	0.81	0.82	0.84
12	0.09	0.19	0.19	0.39	0.47	0.63	0.64	0.77	0.79	0.90
13	0.05	0.06	0.16	0.31	0.44	0.51	0.66	0.78	0.89	0.91
14	0.04	0.08	0.16	0.22	0.30	0.47	0.72	0.76	0.86	0.90
15	0.06	0.09	0.14	0.31	0.38	0.54	0.60	0.74	0.77	0.86
16	0.10	0.16	0.28	0.39	0.58	0.69	0.77	0.88	0.95	0.93
17	0.04	0.12	0.30	0.50	0.57	0.63	0.79	0.87	0.95	0.95
18	0.04	0.09	0.10	0.25	0.41	0.46	0.59	0.75	0.73	0.91
19	0.10	0.07	0.14	0.20	0.32	0.37	0.42	0.62	0.65	0.83
20	0.07	0.10	0.17	0.25	0.27	0.36	0.58	0.44	0.62	0.72
21	0.04	0.09	0.06	0.21	0.19	0.29	0.42	0.62	0.66	0.72
22	0.05	0.09	0.16	0.20	0.37	0.49	0.63	0.73	0.84	0.80
23	0.09	0.13	0.19	0.32	0.29	0.37	0.54	0.69	0.74	0.81
24	0.08	0.06	0.19	0.27	0.32	0.33	0.50	0.60	0.69	0.82
25	0.06	0.11	0.14	0.36	0.47	0.54	0.60	0.82	0.90	0.93
26	0.06	0.09	0.15	0.24	0.34	0.43	0.59	0.65	0.82	0.90
27	0.03	0.05	0.06	0.12	0.24	0.39	0.39	0.41	0.56	0.49
28	0.04	0.14	0.21	0.24	0.46	0.69	0.73	0.76	0.88	0.95
29	0.05	0.06	0.10	0.14	0.35	0.38	0.58	0.70	0.81	0.85
30	0.03	0.04	0.07	0.16	0.22	0.28	0.40	0.55	0.59	0.59
31	0.09	0.10	0.22	0.37	0.45	0.70	0.81	0.89	0.94	0.98
32	0.04	0.08	0.24	0.30	0.49	0.70	0.82	0.87	0.88	0.98
33	0.02	0.06	0.19	0.28	0.56	0.59	0.81	0.85	0.92	0.97
34	0.06	0.11	0.21	0.37	0.50	0.72	0.81	0.95	0.96	0.99
35	0.04	0.10	0.19	0.29	0.45	0.65	0.82	0.92	0.97	0.99
36	0.03	0.09	0.18	0.26	0.36	0.63	0.82	0.93	0.94	0.96
37	0.11	0.12	0.18	0.34	0.66	0.67	0.83	0.93	0.95	0.98
38	0.03	0.17	0.26	0.46	0.49	0.82	0.84	0.95	0.97	1.00
39	0.03	0.14	0.19	0.38	0.56	0.73	0.84	0.90	0.99	0.99
40	0.04	0.19	0.29	0.42	0.56	0.77	0.91	0.96	0.98	0.99
41	0.03	0.04	0.30	0.38	0.56	0.79	0.90	0.93	0.99	0.99
42	0.10	0.08	0.19	0.38	0.54	0.71	0.87	0.93	0.97	0.99

Table 9: POD according to each scenario and each k2 value, k1 = 2, alpha = 0.01

	k2=1	k2=2	k2=3	k2=4	k2=5	k2=6	k2=7	k2=8	k2=9	k2=10
1	0.06	0.07	0.12	0.24	0.36	0.42	0.52	0.64	0.71	0.80
2	0.06	0.11	0.11	0.22	0.25	0.33	0.52	0.52	0.70	0.67
3	0.01	0.08	0.14	0.26	0.27	0.35	0.47	0.59	0.57	0.69
4	0.05	0.12	0.23	0.29	0.44	0.60	0.70	0.71	0.83	0.90
5	0.08	0.08	0.24	0.32	0.36	0.45	0.67	0.84	0.83	0.88
6	0.07	0.12	0.15	0.29	0.25	0.43	0.50	0.62	0.78	0.79
7	0.04	0.05	0.09	0.09	0.14	0.17	0.23	0.32	0.38	0.40
8	0.04	0.03	0.03	0.09	0.09	0.22	0.25	0.21	0.29	0.31
9	0.05	0.01	0.09	0.09	0.08	0.21	0.24	0.34	0.36	0.48
10	0.07	0.15	0.29	0.41	0.48	0.64	0.71	0.75	0.85	0.87
11	0.11	0.22	0.29	0.45	0.55	0.60	0.70	0.74	0.84	0.92
12	0.11	0.15	0.25	0.33	0.55	0.61	0.59	0.72	0.82	0.84
13	0.05	0.10	0.21	0.22	0.42	0.53	0.70	0.72	0.84	0.91
14	0.08	0.03	0.20	0.21	0.38	0.51	0.66	0.73	0.87	0.84
15	0.03	0.09	0.14	0.23	0.25	0.46	0.54	0.74	0.80	0.88
16	0.06	0.11	0.29	0.42	0.45	0.58	0.71	0.90	0.92	0.99
17	0.07	0.12	0.38	0.40	0.51	0.67	0.73	0.87	0.92	0.97
18	0.03	0.05	0.17	0.29	0.30	0.55	0.64	0.68	0.77	0.84
19	0.06	0.14	0.20	0.15	0.28	0.33	0.41	0.53	0.51	0.72
20	0.07	0.14	0.16	0.11	0.33	0.34	0.48	0.54	0.70	0.80
21	0.03	0.07	0.12	0.09	0.15	0.33	0.43	0.55	0.64	0.68
22	0.06	0.17	0.16	0.29	0.26	0.49	0.55	0.67	0.75	0.86
23	0.09	0.05	0.16	0.18	0.31	0.43	0.56	0.71	0.78	0.81
24	0.04	0.07	0.12	0.28	0.37	0.45	0.57	0.62	0.77	0.79
25	0.05	0.15	0.15	0.29	0.39	0.57	0.67	0.76	0.86	0.93
26	0.04	0.10	0.10	0.19	0.35	0.43	0.46	0.72	0.77	0.90
27	0.03	0.04	0.08	0.11	0.16	0.28	0.30	0.46	0.45	0.64
28	0.06	0.12	0.19	0.27	0.33	0.70	0.70	0.80	0.92	0.94
29	0.03	0.04	0.14	0.20	0.29	0.39	0.55	0.76	0.85	0.87
30	0.05	0.04	0.10	0.10	0.12	0.23	0.38	0.42	0.53	0.49
31	0.07	0.13	0.19	0.28	0.48	0.79	0.75	0.85	0.94	1.00
32	0.03	0.11	0.12	0.27	0.57	0.64	0.77	0.87	0.90	1.00
33	0.07	0.11	0.19	0.33	0.42	0.64	0.78	0.89	0.95	0.97
34	0.06	0.13	0.17	0.31	0.46	0.68	0.80	0.87	0.96	1.00
35	0.03	0.14	0.19	0.32	0.57	0.62	0.85	0.90	0.94	0.99
36	0.04	0.05	0.24	0.25	0.44	0.61	0.73	0.91	0.91	0.98
37	0.06	0.14	0.30	0.38	0.58	0.73	0.86	0.91	0.97	0.99
38	0.08	0.07	0.20	0.37	0.53	0.73	0.80	0.94	0.97	1.00
39	0.08	0.16	0.24	0.41	0.51	0.69	0.79	0.92	0.97	1.00
40	0.08	0.16	0.18	0.36	0.52	0.71	0.80	0.93	0.99	0.99
41	0.05	0.11	0.23	0.41	0.43	0.69	0.80	0.91	0.96	0.99
42	0.03	0.16	0.24	0.39	0.53	0.75	0.86	0.97	0.95	1.00

Table 10: POD according to each scenario and each k2 value, k1 = 3, alpha = 0.01

	k2=1	k2=2	k2=3	k2=4	k2=5	k2=6	k2=7	k2=8	k2=9	k2=10
1	0.05	0.06	0.12	0.24	0.31	0.34	0.54	0.58	0.65	0.77
2	0.01	0.05	0.14	0.22	0.27	0.37	0.41	0.55	0.65	0.70
3	0.06	0.07	0.15	0.18	0.21	0.31	0.49	0.57	0.58	0.64
4	0.03	0.13	0.14	0.33	0.39	0.50	0.58	0.76	0.78	0.86
5	0.05	0.12	0.21	0.25	0.39	0.48	0.57	0.71	0.79	0.82
6	0.04	0.09	0.15	0.19	0.34	0.40	0.46	0.55	0.67	0.78
7	0.02	0.03	0.07	0.08	0.17	0.16	0.21	0.27	0.33	0.40
8	0.03	0.02	0.01	0.03	0.12	0.18	0.16	0.20	0.34	0.40
9	0.02	0.03	0.07	0.07	0.11	0.20	0.17	0.34	0.33	0.33
10	0.11	0.17	0.33	0.35	0.51	0.51	0.64	0.77	0.82	0.83
11	0.09	0.19	0.24	0.36	0.53	0.57	0.66	0.77	0.82	0.76
12	0.10	0.22	0.33	0.39	0.38	0.52	0.69	0.75	0.71	0.89
13	0.04	0.13	0.20	0.31	0.36	0.53	0.67	0.73	0.86	0.87
14	0.02	0.07	0.19	0.21	0.41	0.46	0.63	0.83	0.87	0.89
15	0.04	0.08	0.13	0.31	0.32	0.39	0.55	0.66	0.79	0.89
16	0.11	0.10	0.18	0.34	0.50	0.63	0.73	0.89	0.90	0.96
17	0.05	0.09	0.26	0.34	0.53	0.64	0.73	0.75	0.93	0.95
18	0.02	0.09	0.19	0.21	0.36	0.39	0.60	0.70	0.74	0.87
19	0.06	0.09	0.10	0.14	0.22	0.34	0.38	0.49	0.51	0.67
20	0.04	0.03	0.06	0.24	0.22	0.27	0.44	0.48	0.67	0.68
21	0.05	0.06	0.07	0.10	0.23	0.34	0.43	0.47	0.55	0.62
22	0.03	0.07	0.09	0.18	0.26	0.43	0.59	0.60	0.74	0.79
23	0.06	0.08	0.17	0.19	0.29	0.40	0.43	0.68	0.73	0.79
24	0.05	0.10	0.13	0.23	0.26	0.37	0.43	0.59	0.71	0.75
25	0.06	0.11	0.17	0.25	0.32	0.47	0.59	0.80	0.77	0.90
26	0.02	0.11	0.12	0.19	0.34	0.42	0.57	0.69	0.73	0.82
27	0.02	0.04	0.10	0.12	0.17	0.24	0.33	0.55	0.41	0.61
28	0.06	0.16	0.11	0.27	0.35	0.50	0.66	0.72	0.86	0.95
29	0.03	0.08	0.05	0.09	0.28	0.43	0.59	0.66	0.80	0.83
30	0.02	0.04	0.16	0.13	0.17	0.20	0.36	0.44	0.49	0.58
31	0.06	0.05	0.20	0.26	0.41	0.59	0.80	0.90	0.92	0.98
32	0.02	0.10	0.16	0.32	0.51	0.66	0.74	0.84	0.96	0.99
33	0.01	0.12	0.21	0.29	0.40	0.59	0.78	0.86	0.92	0.90
34	0.03	0.07	0.16	0.30	0.43	0.61	0.86	0.90	0.95	0.98
35	0.06	0.08	0.19	0.29	0.41	0.68	0.80	0.84	0.98	0.96
36	0.04	0.07	0.18	0.32	0.45	0.62	0.68	0.82	0.89	0.96
37	0.05	0.05	0.20	0.34	0.43	0.71	0.77	0.91	0.93	0.99
38	0.04	0.14	0.20	0.23	0.60	0.72	0.84	0.90	0.95	1.00
39	0.05	0.07	0.24	0.29	0.51	0.68	0.81	0.92	0.95	0.98
40	0.10	0.09	0.30	0.31	0.46	0.62	0.82	0.93	0.94	1.00
41	0.05	0.09	0.27	0.38	0.45	0.66	0.76	0.89	0.96	0.99
42	0.08	0.05	0.18	0.43	0.51	0.64	0.81	0.86	0.97	1.00

Table 11: POD according to each scenario and each k2 value, k1 = 5, alpha = 0.01, alpha = 0.01

	k2=1	k2=2	k2=3	k2=4	k2=5	k2=6	k2=7	k2=8	k2=9	k2=10
1	0.08	0.03	0.09	0.26	0.26	0.34	0.46	0.39	0.57	0.59
2	0.05	0.07	0.10	0.15	0.28	0.23	0.34	0.47	0.57	0.59
3	0.03	0.06	0.10	0.09	0.18	0.24	0.33	0.46	0.57	0.58
4	0.04	0.07	0.21	0.28	0.39	0.51	0.53	0.66	0.73	0.77
5	0.04	0.11	0.15	0.24	0.37	0.40	0.49	0.53	0.64	0.81
6	0.06	0.03	0.17	0.10	0.22	0.38	0.42	0.50	0.59	0.77
7	0.04	0.03	0.05	0.07	0.04	0.10	0.15	0.19	0.24	0.41
8	0.01	0.03	0.02	0.09	0.15	0.18	0.20	0.23	0.32	0.45
9	0.05	0.03	0.02	0.08	0.08	0.13	0.17	0.22	0.25	0.34
10	0.07	0.16	0.21	0.28	0.40	0.49	0.53	0.64	0.71	0.71
11	0.05	0.18	0.27	0.24	0.40	0.39	0.59	0.62	0.74	0.76
12	0.06	0.16	0.22	0.26	0.48	0.51	0.52	0.66	0.76	0.77
13	0.03	0.04	0.16	0.24	0.31	0.51	0.53	0.65	0.71	0.87
14	0.03	0.04	0.16	0.16	0.36	0.42	0.56	0.72	0.81	0.85
15	0.04	0.02	0.11	0.15	0.27	0.33	0.52	0.53	0.68	0.76
16	0.03	0.08	0.24	0.24	0.47	0.60	0.67	0.82	0.83	0.95
17	0.06	0.09	0.12	0.28	0.38	0.57	0.74	0.73	0.84	0.90
18	0.07	0.09	0.13	0.17	0.27	0.36	0.43	0.65	0.72	0.73
19	0.06	0.08	0.06	0.09	0.17	0.35	0.28	0.48	0.54	0.57
20	0.02	0.05	0.07	0.14	0.20	0.28	0.34	0.48	0.50	0.51
21	0.03	0.06	0.12	0.18	0.18	0.26	0.34	0.48	0.43	0.56
22	0.03	0.10	0.13	0.17	0.19	0.34	0.44	0.57	0.63	0.68
23	0.03	0.05	0.15	0.24	0.20	0.30	0.42	0.51	0.64	0.70
24	0.05	0.06	0.16	0.13	0.26	0.34	0.55	0.45	0.61	0.56
25	0.05	0.04	0.12	0.23	0.30	0.38	0.55	0.59	0.72	0.78
26	0.04	0.07	0.08	0.22	0.17	0.30	0.50	0.65	0.72	0.81
27	0.01	0.02	0.06	0.13	0.16	0.18	0.29	0.37	0.46	0.43
28	0.05	0.06	0.09	0.16	0.32	0.52	0.57	0.73	0.74	0.80
29	0.04	0.03	0.12	0.22	0.31	0.35	0.54	0.59	0.62	0.81
30	0.04	0.07	0.02	0.11	0.07	0.21	0.23	0.42	0.48	0.46
31	0.05	0.08	0.16	0.26	0.44	0.50	0.65	0.87	0.93	0.95
32	0.06	0.13	0.12	0.24	0.41	0.58	0.70	0.82	0.94	0.95
33	0.04	0.08	0.09	0.21	0.39	0.49	0.63	0.75	0.86	0.92
34	0.01	0.04	0.16	0.27	0.48	0.56	0.68	0.81	0.94	0.95
35	0.05	0.10	0.16	0.27	0.41	0.64	0.64	0.76	0.90	0.96
36	0.08	0.07	0.19	0.24	0.45	0.51	0.67	0.77	0.87	0.92
37	0.02	0.05	0.13	0.30	0.46	0.60	0.73	0.85	0.85	0.95
38	0.04	0.11	0.22	0.29	0.41	0.63	0.73	0.88	0.88	0.94
39	0.03	0.11	0.16	0.28	0.45	0.61	0.76	0.85	0.92	0.96
40	0.04	0.11	0.16	0.36	0.45	0.56	0.71	0.83	0.92	0.97
41	0.04	0.07	0.15	0.36	0.50	0.51	0.71	0.87	0.91	0.97
42	0.03	0.11	0.19	0.36	0.46	0.64	0.73	0.86	0.93	0.97

Table 12: POD according to each scenario and each k2 value, k1 = 10, alpha = 0.01

Overall performances of Improved Farrington algorithm ($\alpha = 0.05$)

	FPR k1=0	FPR k1=2	FPR k1=3	FPR k1=5	FPR k1=10
1	0.02	0.02	0.02	0.02	0.02
2	0.02	0.02	0.02	0.01	0.01
3	0.02	0.02	0.02	0.02	0.01
4	0.04	0.04	0.04	0.04	0.04
5	0.04	0.04	0.04	0.03	0.03
6	0.03	0.03	0.03	0.03	0.03
7	0.00	0.00	0.00	0.00	0.00
8	0.00	0.00	0.00	0.00	0.00
9	0.00	0.00	0.00	0.00	0.00
10	0.08	0.07	0.07	0.06	0.06
11	0.08	0.07	0.07	0.07	0.06
12	0.08	0.07	0.07	0.07	0.06
13	0.02	0.02	0.02	0.02	0.02
14	0.02	0.02	0.02	0.02	0.01
15	0.02	0.02	0.02	0.02	0.02
16	0.05	0.05	0.05	0.04	0.04
17	0.06	0.05	0.05	0.05	0.04
18	0.04	0.04	0.04	0.03	0.03
19	0.05	0.05	0.04	0.04	0.04
20	0.05	0.04	0.04	0.04	0.04
21	0.04	0.03	0.03	0.03	0.03
22	0.05	0.05	0.05	0.04	0.04
23	0.05	0.04	0.04	0.04	0.04
24	0.05	0.04	0.04	0.04	0.03
25	0.04	0.04	0.04	0.03	0.03
26	0.03	0.03	0.03	0.03	0.02
27	0.02	0.02	0.02	0.02	0.01
28	0.04	0.04	0.04	0.04	0.03
29	0.03	0.02	0.03	0.02	0.02
30	0.02	0.02	0.02	0.02	0.02
31	0.03	0.03	0.03	0.03	0.02
32	0.03	0.03	0.03	0.02	0.02
33	0.03	0.02	0.02	0.02	0.02
34	0.03	0.03	0.03	0.03	0.02
35	0.03	0.03	0.03	0.02	0.02
36	0.03	0.03	0.03	0.03	0.02
37	0.04	0.03	0.03	0.03	0.03
38	0.04	0.03	0.03	0.03	0.03
39	0.03	0.03	0.03	0.02	0.02
40	0.04	0.04	0.04	0.03	0.03
41	0.03	0.03	0.03	0.03	0.03
42	0.03	0.03	0.03	0.03	0.03

Table 13: FPR according to each scenario and each k1 value, alpha = 0.05

	k2=1	k2=2	k2=3	k2=4	k2=5	k2=6	k2=7	k2=8	k2=9	k2=10
1	0.19	0.26	0.33	0.45	0.62	0.68	0.77	0.88	0.98	0.95
2	0.18	0.15	0.26	0.52	0.56	0.69	0.77	0.88	0.89	0.95
3	0.16	0.21	0.22	0.35	0.48	0.53	0.72	0.82	0.88	0.86
4	0.19	0.33	0.41	0.55	0.66	0.77	0.86	0.96	0.91	0.99
5	0.22	0.22	0.50	0.58	0.67	0.71	0.84	0.93	0.99	0.98
6	0.22	0.27	0.28	0.48	0.58	0.75	0.76	0.79	0.95	0.91
7	0.05	0.11	0.11	0.19	0.22	0.29	0.29	0.34	0.53	0.51
8	0.01	0.04	0.06	0.11	0.16	0.30	0.35	0.33	0.47	0.57
9	0.02	0.08	0.07	0.16	0.16	0.32	0.44	0.50	0.47	0.53
10	0.33	0.46	0.53	0.69	0.77	0.77	0.90	0.91	0.96	0.91
11	0.28	0.34	0.56	0.72	0.80	0.80	0.87	0.95	0.94	0.98
12	0.35	0.37	0.54	0.64	0.80	0.88	0.92	0.94	0.88	0.97
13	0.10	0.25	0.35	0.57	0.72	0.78	0.84	0.96	0.98	0.99
14	0.18	0.24	0.41	0.49	0.60	0.79	0.89	0.88	0.94	0.98
15	0.21	0.22	0.28	0.44	0.53	0.73	0.83	0.89	0.94	0.99
16	0.26	0.39	0.51	0.65	0.77	0.95	0.95	0.99	1.00	1.00
17	0.22	0.28	0.52	0.63	0.81	0.86	0.96	0.98	0.97	1.00
18	0.24	0.27	0.34	0.52	0.70	0.68	0.89	0.87	0.95	1.00
19	0.17	0.20	0.43	0.53	0.63	0.76	0.79	0.93	0.95	0.97
20	0.17	0.23	0.34	0.47	0.63	0.74	0.84	0.89	0.89	0.94
21	0.12	0.22	0.30	0.43	0.57	0.76	0.74	0.86	0.96	0.97
22	0.19	0.28	0.43	0.52	0.63	0.76	0.84	0.90	0.94	0.98
23	0.18	0.25	0.44	0.46	0.65	0.76	0.88	0.93	0.92	0.98
24	0.20	0.32	0.41	0.56	0.65	0.71	0.84	0.90	0.93	0.98
25	0.20	0.34	0.49	0.63	0.74	0.81	0.93	0.92	0.98	0.99
26	0.13	0.18	0.37	0.50	0.69	0.76	0.89	0.92	0.94	0.96
27	0.13	0.14	0.22	0.31	0.43	0.46	0.68	0.74	0.74	0.81
28	0.18	0.38	0.48	0.64	0.73	0.82	0.92	0.96	0.98	0.99
29	0.13	0.24	0.25	0.48	0.61	0.76	0.80	0.95	0.98	1.00
30	0.04	0.18	0.17	0.28	0.33	0.48	0.54	0.64	0.74	0.85
31	0.13	0.34	0.39	0.61	0.74	0.94	0.95	0.98	0.99	1.00
32	0.15	0.31	0.48	0.65	0.77	0.91	0.94	0.99	0.98	1.00
33	0.15	0.26	0.42	0.62	0.75	0.88	0.95	0.98	1.00	1.00
34	0.14	0.28	0.51	0.62	0.85	0.92	0.93	0.99	0.99	1.00
35	0.21	0.30	0.41	0.69	0.72	0.90	0.93	1.00	1.00	0.99
36	0.14	0.30	0.40	0.56	0.84	0.86	0.95	0.99	1.00	1.00
37	0.11	0.32	0.40	0.67	0.78	0.88	0.95	0.96	0.99	1.00
38	0.20	0.27	0.48	0.64	0.78	0.90	0.98	0.99	1.00	1.00
39	0.12	0.33	0.52	0.72	0.78	0.90	0.91	0.98	0.99	1.00
40	0.19	0.35	0.48	0.77	0.84	0.94	0.97	0.99	0.99	1.00
41	0.20	0.34	0.43	0.63	0.83	0.89	0.95	1.00	1.00	1.00
42	0.19	0.34	0.48	0.55	0.76	0.91	0.98	0.97	1.00	1.00

Table 14: POD according to each scenario and each k2 value, k1 = 0, alpha = 0.05

	k2=1	k2=2	k2=3	k2=4	k2=5	k2=6	k2=7	k2=8	k2=9	k2=10
1	0.11	0.24	0.34	0.42	0.63	0.64	0.79	0.80	0.83	0.94
2	0.11	0.27	0.30	0.41	0.59	0.71	0.71	0.81	0.78	0.91
3	0.09	0.18	0.25	0.39	0.43	0.61	0.76	0.73	0.86	0.91
4	0.22	0.31	0.37	0.50	0.69	0.73	0.88	0.93	0.95	0.99
5	0.18	0.26	0.36	0.57	0.70	0.79	0.86	0.93	0.96	0.97
6	0.08	0.28	0.37	0.48	0.54	0.64	0.70	0.88	0.90	0.88
7	0.05	0.06	0.04	0.18	0.21	0.22	0.36	0.40	0.53	0.57
8	0.07	0.08	0.07	0.11	0.26	0.26	0.24	0.39	0.44	0.62
9	0.02	0.03	0.07	0.13	0.21	0.31	0.40	0.31	0.46	0.63
10	0.27	0.46	0.56	0.65	0.73	0.83	0.84	0.92	0.92	0.95
11	0.24	0.41	0.56	0.56	0.76	0.83	0.86	0.94	0.94	0.93
12	0.23	0.31	0.43	0.57	0.75	0.81	0.87	0.96	0.94	0.96
13	0.15	0.22	0.37	0.55	0.74	0.69	0.91	0.92	0.95	0.96
14	0.11	0.19	0.36	0.49	0.52	0.76	0.86	0.91	0.95	0.98
15	0.16	0.20	0.31	0.52	0.71	0.75	0.80	0.95	0.94	0.95
16	0.22	0.37	0.47	0.61	0.80	0.91	0.94	0.96	0.99	0.99
17	0.22	0.29	0.50	0.65	0.79	0.88	0.95	0.95	1.00	0.99
18	0.11	0.21	0.32	0.45	0.60	0.73	0.82	0.88	0.93	0.99
19	0.18	0.24	0.39	0.48	0.65	0.75	0.81	0.90	0.92	0.98
20	0.15	0.26	0.35	0.57	0.64	0.71	0.89	0.85	0.90	0.97
21	0.08	0.15	0.27	0.46	0.54	0.70	0.83	0.88	0.89	0.94
22	0.12	0.22	0.40	0.55	0.59	0.78	0.82	0.95	0.96	0.94
23	0.14	0.27	0.39	0.65	0.66	0.76	0.85	0.96	0.91	0.93
24	0.17	0.23	0.40	0.55	0.66	0.73	0.83	0.91	0.93	0.94
25	0.13	0.29	0.37	0.68	0.75	0.79	0.88	0.99	0.99	0.98
26	0.10	0.29	0.37	0.45	0.70	0.73	0.86	0.87	0.95	0.97
27	0.05	0.12	0.19	0.40	0.44	0.54	0.65	0.71	0.77	0.75
28	0.16	0.33	0.41	0.56	0.73	0.87	0.93	0.96	0.98	0.98
29	0.11	0.16	0.27	0.41	0.61	0.64	0.86	0.90	0.96	0.99
30	0.07	0.14	0.20	0.34	0.48	0.48	0.57	0.75	0.73	0.78
31	0.18	0.26	0.47	0.59	0.73	0.87	0.94	0.98	0.99	1.00
32	0.14	0.32	0.49	0.61	0.77	0.85	0.94	0.99	0.97	0.99
33	0.08	0.23	0.35	0.58	0.77	0.75	0.92	0.96	0.98	1.00
34	0.20	0.27	0.44	0.67	0.81	0.93	0.92	0.98	1.00	1.00
35	0.17	0.26	0.35	0.57	0.76	0.83	0.94	1.00	0.99	1.00
36	0.13	0.22	0.39	0.56	0.64	0.82	0.97	0.98	0.99	1.00
37	0.22	0.28	0.50	0.56	0.83	0.89	0.94	0.98	1.00	0.98
38	0.12	0.30	0.54	0.67	0.74	0.95	0.97	0.99	1.00	1.00
39	0.12	0.41	0.43	0.66	0.86	0.89	0.97	1.00	1.00	1.00
40	0.17	0.43	0.46	0.69	0.85	0.92	0.98	0.98	0.98	1.00
41	0.22	0.18	0.54	0.65	0.79	0.96	0.97	0.99	1.00	1.00
42	0.17	0.29	0.46	0.61	0.74	0.89	0.98	0.98	1.00	1.00

Table 15: POD according to each scenario and each k2 value, k1 = 2, alpha = 0.05

	k2=1	k2=2	k2=3	k2=4	k2=5	k2=6	k2=7	k2=8	k2=9	k2=10
1	0.15	0.21	0.31	0.44	0.64	0.65	0.75	0.86	0.88	0.90
2	0.13	0.26	0.36	0.40	0.54	0.64	0.76	0.79	0.85	0.88
3	0.06	0.19	0.24	0.37	0.50	0.59	0.73	0.83	0.85	0.91
4	0.16	0.29	0.37	0.53	0.68	0.81	0.84	0.87	0.96	0.95
5	0.14	0.22	0.44	0.56	0.64	0.71	0.83	0.93	0.95	0.94
6	0.19	0.27	0.32	0.50	0.49	0.66	0.80	0.80	0.90	0.91
7	0.06	0.07	0.12	0.11	0.20	0.24	0.30	0.40	0.50	0.53
8	0.08	0.06	0.08	0.17	0.16	0.30	0.32	0.31	0.40	0.50
9	0.05	0.05	0.14	0.13	0.12	0.31	0.35	0.54	0.48	0.57
10	0.28	0.37	0.49	0.64	0.72	0.82	0.89	0.89	0.91	0.91
11	0.34	0.41	0.47	0.74	0.76	0.81	0.82	0.87	0.94	0.97
12	0.27	0.34	0.48	0.58	0.76	0.81	0.79	0.91	0.92	0.92
13	0.17	0.29	0.34	0.49	0.70	0.71	0.87	0.92	0.96	0.99
14	0.19	0.17	0.38	0.47	0.70	0.75	0.86	0.94	0.94	0.96
15	0.07	0.17	0.42	0.43	0.52	0.73	0.83	0.86	0.95	0.93
16	0.17	0.33	0.50	0.72	0.75	0.87	0.92	0.97	0.99	1.00
17	0.30	0.30	0.50	0.68	0.77	0.81	0.95	0.98	0.99	0.98
18	0.12	0.20	0.39	0.51	0.62	0.77	0.85	0.86	0.93	0.97
19	0.12	0.29	0.36	0.49	0.57	0.69	0.78	0.85	0.84	0.96
20	0.15	0.29	0.32	0.45	0.67	0.72	0.84	0.90	0.97	0.98
21	0.11	0.28	0.33	0.44	0.48	0.66	0.78	0.89	0.91	0.97
22	0.14	0.33	0.39	0.56	0.58	0.79	0.79	0.90	0.93	0.98
23	0.18	0.25	0.36	0.47	0.70	0.78	0.87	0.94	0.93	0.97
24	0.12	0.20	0.38	0.48	0.73	0.72	0.83	0.91	0.95	0.93
25	0.10	0.27	0.36	0.59	0.61	0.83	0.93	0.97	0.96	0.99
26	0.13	0.20	0.29	0.39	0.61	0.75	0.79	0.93	0.92	0.97
27	0.07	0.13	0.19	0.32	0.33	0.52	0.61	0.69	0.73	0.83
28	0.21	0.26	0.45	0.54	0.65	0.85	0.87	0.96	0.99	1.00
29	0.14	0.19	0.37	0.42	0.62	0.71	0.80	0.93	0.98	0.98
30	0.12	0.18	0.20	0.32	0.29	0.45	0.58	0.63	0.67	0.69
31	0.12	0.28	0.42	0.53	0.69	0.92	0.94	0.97	0.98	1.00
32	0.10	0.29	0.42	0.59	0.75	0.82	0.92	0.99	0.99	1.00
33	0.19	0.23	0.40	0.57	0.71	0.89	0.95	0.97	1.00	1.00
34	0.14	0.26	0.47	0.54	0.73	0.88	0.95	0.96	1.00	1.00
35	0.19	0.27	0.43	0.62	0.74	0.82	0.95	0.96	0.98	1.00
36	0.11	0.23	0.41	0.56	0.73	0.85	0.95	0.96	0.96	1.00
37	0.21	0.36	0.54	0.58	0.80	0.89	0.95	0.98	0.98	1.00
38	0.17	0.24	0.39	0.61	0.79	0.92	0.90	0.99	0.99	1.00
39	0.16	0.30	0.51	0.64	0.79	0.93	0.94	0.98	1.00	1.00
40	0.17	0.39	0.41	0.65	0.75	0.87	0.94	1.00	1.00	1.00
41	0.07	0.28	0.41	0.66	0.71	0.90	0.93	0.98	0.99	1.00
42	0.16	0.38	0.46	0.61	0.78	0.86	0.95	1.00	1.00	1.00

Table 16: POD according to each scenario and each k2 value, k1 = 3, alpha = 0.05

	k2=1	k2=2	k2=3	k2=4	k2=5	k2=6	k2=7	k2=8	k2=9	k2=10
1	0.18	0.19	0.30	0.44	0.51	0.56	0.79	0.85	0.89	0.93
2	0.12	0.18	0.27	0.34	0.48	0.61	0.65	0.82	0.84	0.88
3	0.11	0.17	0.27	0.35	0.52	0.54	0.71	0.79	0.82	0.87
4	0.22	0.28	0.32	0.55	0.67	0.70	0.83	0.91	0.97	0.97
5	0.15	0.22	0.44	0.44	0.72	0.71	0.78	0.89	0.92	0.92
6	0.11	0.21	0.33	0.49	0.51	0.66	0.70	0.79	0.85	0.89
7	0.05	0.05	0.07	0.16	0.23	0.22	0.32	0.35	0.47	0.59
8	0.04	0.06	0.07	0.11	0.17	0.28	0.29	0.42	0.47	0.53
9	0.06	0.07	0.09	0.14	0.19	0.37	0.29	0.44	0.44	0.45
10	0.22	0.38	0.62	0.61	0.77	0.76	0.82	0.90	0.92	0.93
11	0.24	0.46	0.49	0.68	0.77	0.78	0.78	0.89	0.94	0.92
12	0.24	0.42	0.53	0.62	0.64	0.79	0.88	0.90	0.86	0.96
13	0.14	0.27	0.39	0.55	0.63	0.78	0.79	0.93	0.95	0.97
14	0.09	0.22	0.40	0.49	0.65	0.71	0.86	0.89	0.96	0.97
15	0.11	0.17	0.37	0.49	0.59	0.70	0.80	0.85	0.90	0.97
16	0.20	0.30	0.43	0.65	0.83	0.89	0.85	0.96	0.97	0.98
17	0.17	0.27	0.43	0.53	0.78	0.83	0.88	0.91	0.98	0.98
18	0.13	0.17	0.33	0.44	0.65	0.63	0.86	0.85	0.90	0.96
19	0.12	0.28	0.31	0.38	0.50	0.68	0.76	0.77	0.90	0.89
20	0.12	0.15	0.28	0.52	0.53	0.71	0.82	0.82	0.93	0.97
21	0.11	0.16	0.23	0.38	0.63	0.67	0.75	0.79	0.89	0.84
22	0.15	0.24	0.25	0.52	0.58	0.67	0.85	0.85	0.94	0.94
23	0.15	0.29	0.41	0.51	0.58	0.71	0.75	0.91	0.92	0.97
24	0.13	0.21	0.34	0.50	0.60	0.67	0.77	0.84	0.92	0.95
25	0.13	0.29	0.34	0.54	0.58	0.73	0.78	0.99	0.95	0.97
26	0.07	0.24	0.34	0.52	0.60	0.71	0.75	0.92	0.96	0.97
27	0.07	0.12	0.15	0.33	0.38	0.46	0.66	0.78	0.68	0.80
28	0.13	0.29	0.35	0.47	0.64	0.76	0.89	0.89	0.97	1.00
29	0.18	0.20	0.19	0.30	0.55	0.65	0.86	0.93	0.94	0.96
30	0.08	0.15	0.29	0.30	0.44	0.37	0.52	0.63	0.65	0.78
31	0.14	0.25	0.39	0.50	0.63	0.83	0.92	0.98	0.99	1.00
32	0.13	0.32	0.52	0.60	0.76	0.82	0.92	0.96	0.99	0.99
33	0.09	0.22	0.43	0.57	0.77	0.84	0.90	0.94	0.97	0.99
34	0.11	0.22	0.37	0.55	0.71	0.81	0.95	0.97	0.97	0.99
35	0.13	0.33	0.38	0.59	0.72	0.90	0.93	0.97	0.99	0.99
36	0.08	0.21	0.32	0.55	0.73	0.82	0.85	0.95	0.96	1.00
37	0.11	0.23	0.47	0.62	0.74	0.91	0.93	0.99	0.95	0.99
38	0.13	0.27	0.37	0.57	0.83	0.85	0.97	0.99	1.00	1.00
39	0.15	0.19	0.47	0.52	0.74	0.89	0.92	0.98	0.99	1.00
40	0.18	0.27	0.50	0.68	0.77	0.86	0.94	0.96	0.99	1.00
41	0.16	0.22	0.48	0.66	0.68	0.86	0.96	0.96	0.98	1.00
42	0.16	0.23	0.36	0.64	0.77	0.82	0.96	0.96	1.00	1.00

Table 17: POD according to each scenario and each k2 value, k1 = 5, alpha = 0.05

	k2=1	k2=2	k2=3	k2=4	k2=5	k2=6	k2=7	k2=8	k2=9	k2=10
1	0.20	0.22	0.27	0.49	0.44	0.57	0.65	0.71	0.75	0.82
2	0.15	0.20	0.27	0.28	0.49	0.51	0.65	0.76	0.81	0.79
3	0.11	0.14	0.25	0.31	0.37	0.41	0.59	0.67	0.76	0.84
4	0.11	0.17	0.36	0.46	0.52	0.69	0.80	0.84	0.93	0.92
5	0.14	0.24	0.30	0.41	0.56	0.70	0.73	0.78	0.84	0.94
6	0.13	0.10	0.36	0.34	0.45	0.54	0.68	0.67	0.79	0.95
7	0.07	0.07	0.08	0.12	0.12	0.18	0.28	0.31	0.45	0.55
8	0.03	0.06	0.06	0.14	0.27	0.24	0.32	0.31	0.48	0.60
9	0.05	0.05	0.02	0.12	0.17	0.26	0.25	0.38	0.40	0.54
10	0.15	0.29	0.42	0.52	0.61	0.77	0.74	0.78	0.89	0.85
11	0.17	0.35	0.44	0.47	0.59	0.68	0.77	0.83	0.88	0.91
12	0.26	0.32	0.44	0.50	0.69	0.75	0.72	0.89	0.90	0.94
13	0.11	0.21	0.33	0.45	0.56	0.71	0.78	0.84	0.88	0.97
14	0.13	0.14	0.38	0.41	0.59	0.65	0.76	0.86	0.93	0.96
15	0.10	0.11	0.30	0.32	0.53	0.60	0.75	0.73	0.88	0.91
16	0.17	0.25	0.43	0.43	0.71	0.77	0.87	0.95	0.97	0.98
17	0.15	0.26	0.32	0.42	0.66	0.83	0.89	0.89	0.94	0.99
18	0.16	0.17	0.25	0.38	0.49	0.62	0.73	0.87	0.88	0.91
19	0.11	0.23	0.21	0.31	0.49	0.61	0.62	0.72	0.84	0.82
20	0.14	0.19	0.20	0.41	0.46	0.57	0.65	0.76	0.78	0.85
21	0.12	0.14	0.29	0.40	0.42	0.53	0.65	0.75	0.80	0.85
22	0.15	0.25	0.30	0.41	0.49	0.62	0.74	0.77	0.89	0.89
23	0.14	0.11	0.36	0.47	0.48	0.60	0.72	0.82	0.87	0.89
24	0.13	0.16	0.33	0.49	0.46	0.61	0.77	0.74	0.90	0.79
25	0.09	0.20	0.34	0.48	0.65	0.62	0.84	0.84	0.92	0.93
26	0.12	0.17	0.27	0.44	0.46	0.68	0.75	0.79	0.93	0.92
27	0.05	0.08	0.12	0.27	0.32	0.35	0.52	0.64	0.63	0.68
28	0.15	0.17	0.29	0.45	0.61	0.66	0.76	0.92	0.92	0.96
29	0.10	0.15	0.29	0.49	0.64	0.67	0.76	0.83	0.86	0.97
30	0.06	0.14	0.16	0.29	0.25	0.35	0.47	0.58	0.62	0.68
31	0.13	0.25	0.41	0.48	0.70	0.77	0.87	0.94	0.98	0.99
32	0.12	0.27	0.39	0.45	0.60	0.85	0.91	0.94	0.99	0.99
33	0.14	0.17	0.26	0.48	0.62	0.75	0.81	0.92	0.96	0.96
34	0.10	0.25	0.27	0.51	0.73	0.81	0.91	0.91	0.98	1.00
35	0.13	0.25	0.33	0.48	0.68	0.83	0.79	0.91	0.94	0.98
36	0.14	0.19	0.37	0.41	0.68	0.75	0.87	0.95	0.95	0.98
37	0.12	0.20	0.40	0.53	0.73	0.79	0.92	0.94	0.98	1.00
38	0.19	0.22	0.51	0.60	0.73	0.85	0.96	0.98	0.97	0.99
39	0.12	0.23	0.38	0.49	0.66	0.80	0.91	0.97	0.98	0.98
40	0.14	0.23	0.32	0.62	0.72	0.82	0.88	0.95	0.99	1.00
41	0.14	0.19	0.31	0.56	0.79	0.79	0.87	0.93	0.98	0.99
42	0.13	0.29	0.38	0.65	0.74	0.84	0.93	0.96	0.97	0.99

Table 18: POD according to each scenario and each k2 value, k1 = 10, alpha = 0.05
