# Supplementary material for: Evaluation and comparison of statistical methods for early temporal detection of outbreaks: A simulation-based study
Source: PLoS One. 2017 Jul 17;12(7):e0181227. doi: 10.1371/journal.pone.0181227 (PMC5513450; doi:10.1371/journal.pone.0181227)
Supplement: S3 Appendix — (PDF) [file pone.0181227.s003.pdf]

Evaluation and Comparison of Statistical Methods for Early Temporal Detection of Outbreaks: a Simulation-Based Study

Appendix S3: Overall performances of Original Farrington algorithm ($\alpha = 0.001, 0.01$ and 0.05)

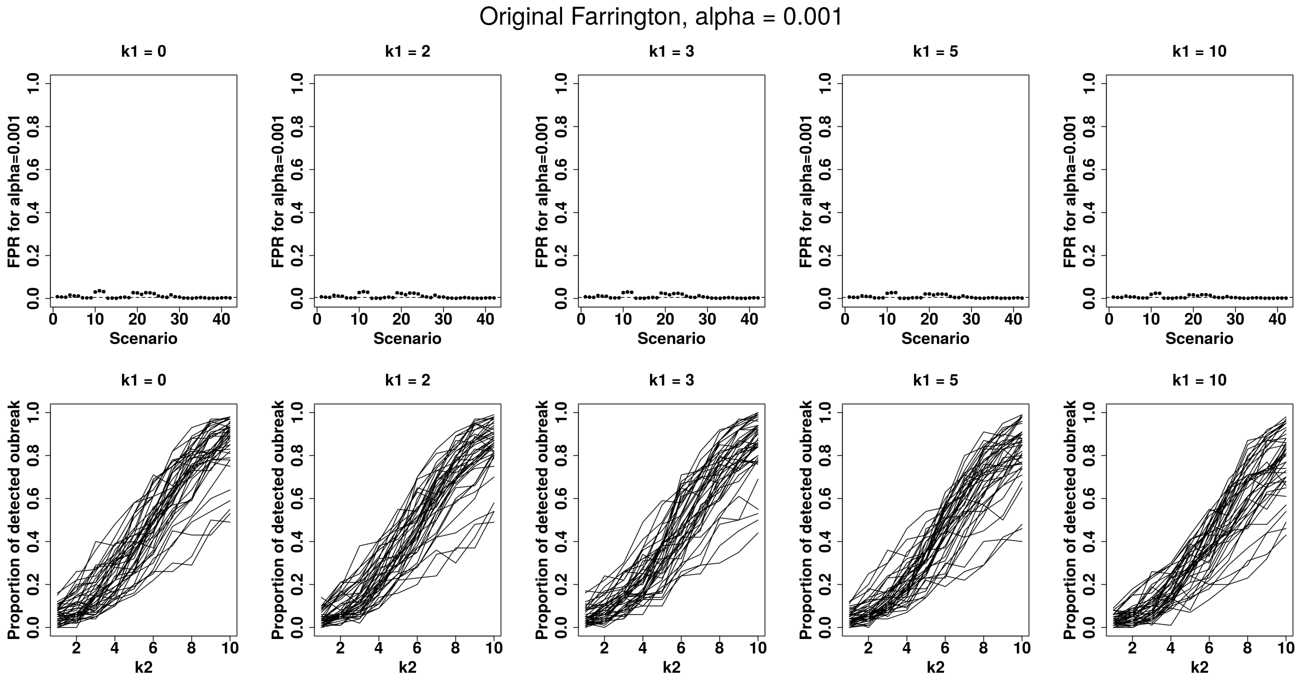

Figure 1: Original Farrington algorithm performances for $\alpha = 0.001$ by increasing past outbreak amplitude $k_1 = 0$, 2, 3, 5 or 10 with (i) on the first row: false positive rate for 42 simulated scenarios, (ii) on the second row: probability of detection for 42 simulated scenarios (each curve corresponding to a scenario) by increasing current outbreak amplitude $k_2 = 1$ to 10.

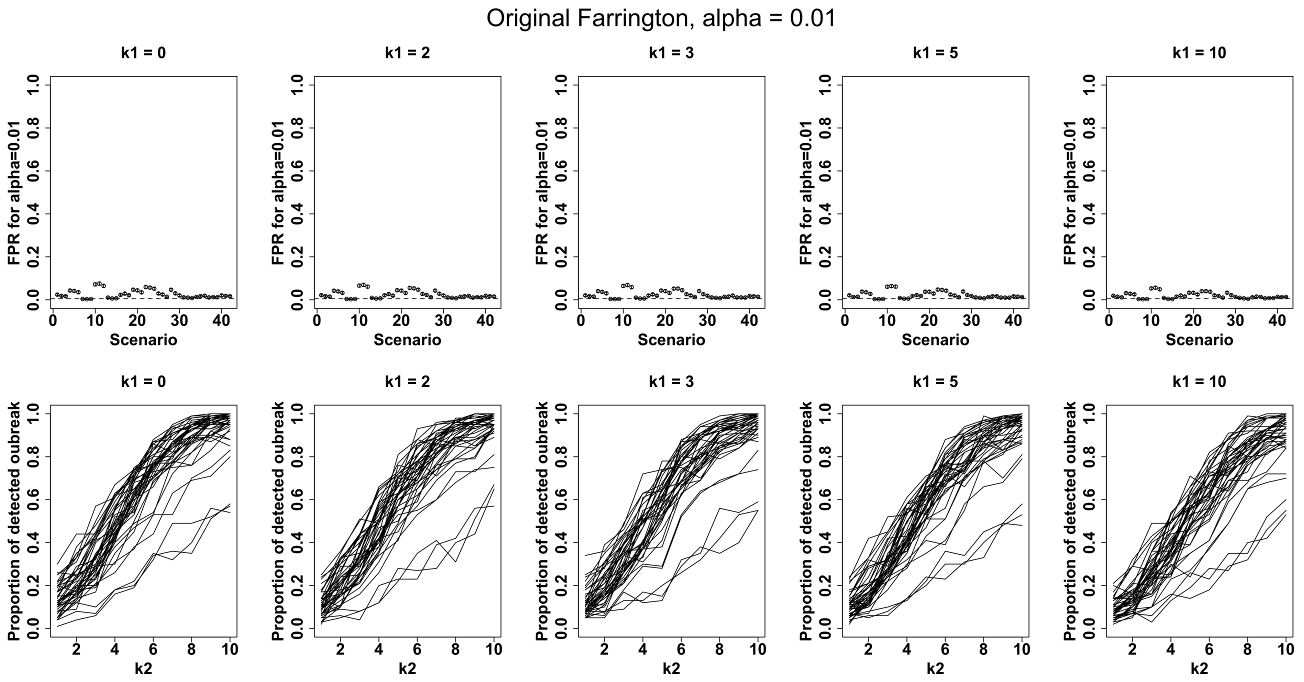

Figure 2: Original Farrington algorithm performances for $\alpha = 0.01$ by increasing past outbreak amplitude $k_1 = 0$, 2, 3, 5 or 10 with (i) on the first row: false positive rate for 42 simulated scenarios, (ii) on the second row: probability of detection for 42 simulated scenarios (each curve corresponding to a scenario) by increasing current outbreak amplitude $k_2 = 1$ to 10.

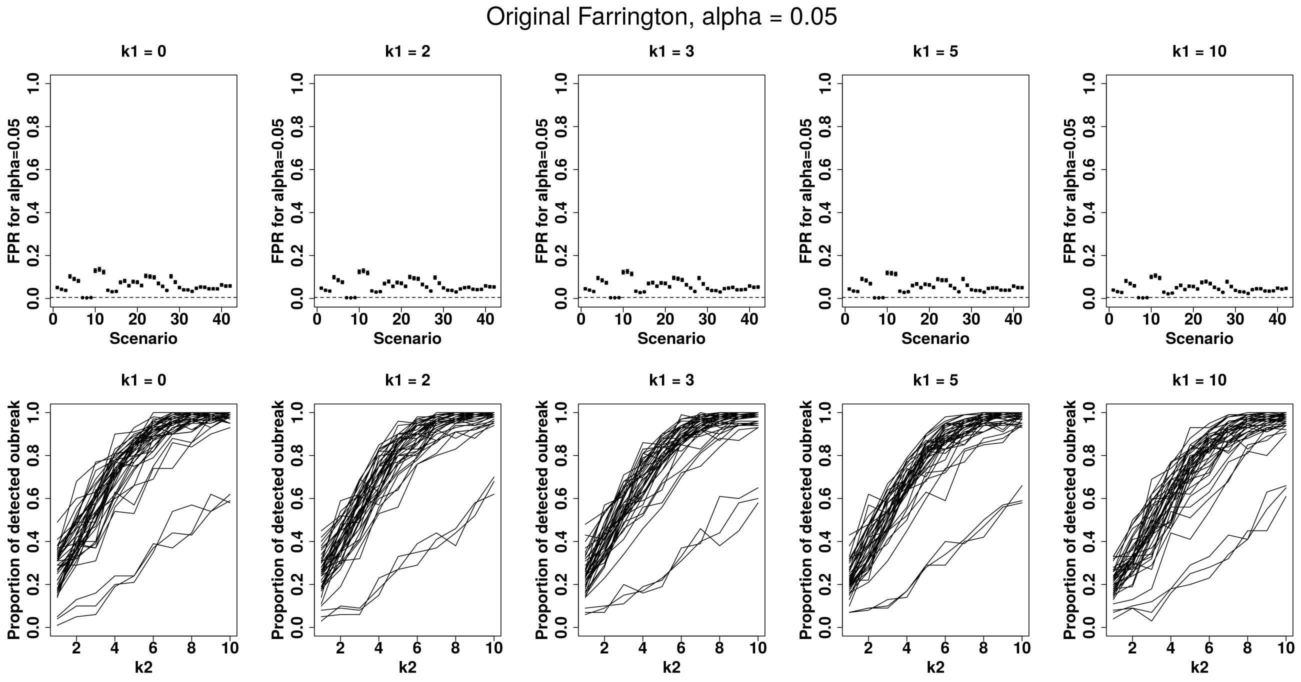

Figure 3: Original Farrington algorithm performances for $\alpha = 0.05$ by increasing past outbreak amplitude k_1 = 0, 2, 3, 5 or 10 with (i) on the first row: false positive rate for 42 simulated scenarios, (ii) on the second row: probability of detection for 42 simulated scenarios (each curve corresponding to a scenario) by increasing current outbreak amplitude $k_2 = 1$ to 10.

Overall performances of Original Farrington algorithm ($\alpha = 0.001$)

	FPR k1=0	FPR k1=2	FPR k1=3	FPR k1=5	FPR k1=10
1	0.01	0.01	0.01	0.01	0.01
2	0.01	0.01	0.01	0.01	0.00
3	0.01	0.01	0.01	0.01	0.01
4	0.02	0.01	0.01	0.01	0.01
5	0.01	0.01	0.01	0.01	0.01
6	0.01	0.01	0.01	0.01	0.01
7	0.00	0.00	0.00	0.00	0.00
8	0.00	0.00	0.00	0.00	0.00
9	0.00	0.00	0.00	0.00	0.00
10	0.03	0.03	0.03	0.02	0.02
11	0.04	0.03	0.03	0.03	0.02
12	0.03	0.03	0.03	0.03	0.02
13	0.00	0.00	0.00	0.00	0.00
14	0.00	0.00	0.00	0.00	0.00
15	0.00	0.00	0.00	0.00	0.00
16	0.00	0.00	0.00	0.00	0.00
17	0.01	0.01	0.01	0.00	0.00
18	0.00	0.00	0.00	0.00	0.00
19	0.03	0.03	0.03	0.02	0.02
20	0.03	0.02	0.02	0.02	0.02
21	0.02	0.02	0.02	0.02	0.01
22	0.03	0.03	0.02	0.02	0.02
23	0.03	0.02	0.02	0.02	0.02
24	0.02	0.02	0.02	0.02	0.01
25	0.01	0.01	0.01	0.01	0.01
26	0.01	0.01	0.01	0.01	0.00
27	0.01	0.00	0.00	0.00	0.00
28	0.02	0.02	0.01	0.01	0.01
29	0.01	0.01	0.01	0.01	0.00
30	0.01	0.01	0.01	0.00	0.00
31	0.00	0.00	0.00	0.00	0.00
32	0.00	0.00	0.00	0.00	0.00
33	0.00	0.00	0.00	0.00	0.00
34	0.00	0.00	0.00	0.00	0.00
35	0.00	0.00	0.00	0.00	0.00
36	0.00	0.00	0.00	0.00	0.00
37	0.00	0.00	0.00	0.00	0.00
38	0.00	0.00	0.00	0.00	0.00
39	0.00	0.00	0.00	0.00	0.00
40	0.00	0.00	0.00	0.00	0.00
41	0.00	0.00	0.00	0.00	0.00
42	0.00	0.00	0.00	0.00	0.00

Table 1: FPR according to each scenario and each k1 value, alpha = 0.001

	k2=1	k2=2	k2=3	k2=4	k2=5	k2=6	k2=7	k2=8	k2=9	k2=10
1	0.11	0.14	0.18	0.28	0.27	0.41	0.51	0.73	0.82	0.85
2	0.08	0.12	0.09	0.29	0.37	0.42	0.55	0.64	0.78	0.85
3	0.07	0.09	0.10	0.19	0.25	0.35	0.49	0.60	0.77	0.75
4	0.08	0.20	0.21	0.39	0.40	0.54	0.72	0.79	0.79	0.90
5	0.08	0.11	0.26	0.39	0.43	0.44	0.62	0.74	0.90	0.91
6	0.09	0.10	0.15	0.32	0.34	0.50	0.57	0.59	0.78	0.79
7	0.05	0.09	0.08	0.17	0.19	0.26	0.26	0.33	0.50	0.49
8	0.01	0.04	0.05	0.11	0.15	0.22	0.30	0.29	0.45	0.55
9	0.03	0.07	0.07	0.13	0.16	0.27	0.45	0.43	0.43	0.53
10	0.15	0.26	0.27	0.42	0.58	0.64	0.78	0.75	0.83	0.81
11	0.16	0.22	0.36	0.48	0.58	0.61	0.65	0.70	0.80	0.88
12	0.12	0.17	0.40	0.38	0.53	0.71	0.66	0.75	0.78	0.78
13	0.00	0.04	0.04	0.16	0.26	0.36	0.54	0.60	0.82	0.83
14	0.06	0.03	0.11	0.10	0.27	0.34	0.52	0.60	0.74	0.82
15	0.05	0.06	0.06	0.13	0.20	0.41	0.53	0.69	0.77	0.87
16	0.02	0.07	0.14	0.17	0.30	0.45	0.62	0.75	0.83	0.92
17	0.03	0.04	0.16	0.22	0.35	0.50	0.61	0.76	0.80	0.93
18	0.06	0.05	0.14	0.19	0.25	0.30	0.47	0.52	0.65	0.78
19	0.11	0.11	0.29	0.30	0.40	0.52	0.57	0.79	0.81	0.91
20	0.08	0.11	0.17	0.31	0.35	0.52	0.65	0.73	0.73	0.89
21	0.08	0.15	0.20	0.30	0.38	0.46	0.58	0.74	0.83	0.93
22	0.11	0.16	0.27	0.34	0.51	0.61	0.78	0.80	0.83	0.92
23	0.10	0.17	0.28	0.29	0.53	0.67	0.77	0.82	0.81	0.96
24	0.09	0.19	0.21	0.33	0.45	0.49	0.72	0.81	0.89	0.89
25	0.03	0.15	0.15	0.36	0.46	0.55	0.69	0.80	0.96	0.93
26	0.02	0.06	0.16	0.22	0.34	0.49	0.59	0.72	0.81	0.89
27	0.08	0.02	0.05	0.10	0.20	0.25	0.39	0.49	0.54	0.59
28	0.08	0.22	0.24	0.34	0.45	0.69	0.75	0.81	0.90	0.95
29	0.06	0.12	0.10	0.31	0.43	0.48	0.63	0.78	0.91	0.93
30	0.01	0.11	0.07	0.14	0.18	0.32	0.38	0.51	0.60	0.64
31	0.04	0.07	0.06	0.17	0.29	0.49	0.65	0.88	0.88	0.94
32	0.00	0.00	0.15	0.22	0.25	0.53	0.64	0.80	0.93	0.97
33	0.00	0.03	0.06	0.17	0.29	0.43	0.73	0.83	0.81	0.96
34	0.00	0.03	0.15	0.13	0.36	0.45	0.60	0.71	0.90	0.95
35	0.06	0.05	0.08	0.24	0.26	0.53	0.63	0.78	0.89	0.93
36	0.04	0.05	0.11	0.15	0.40	0.37	0.63	0.74	0.78	0.92
37	0.00	0.07	0.09	0.29	0.32	0.52	0.75	0.85	0.95	0.98
38	0.02	0.02	0.14	0.22	0.38	0.54	0.73	0.89	0.94	0.97
39	0.01	0.05	0.20	0.16	0.38	0.56	0.62	0.84	0.97	0.97
40	0.06	0.08	0.23	0.32	0.39	0.61	0.82	0.93	0.96	0.98
41	0.07	0.06	0.12	0.22	0.53	0.61	0.82	0.88	0.92	0.98
42	0.02	0.02	0.16	0.26	0.43	0.68	0.75	0.84	0.96	0.98

Table 2: POD according to each scenario and each k2 value, k1 = 0, alpha = 0.001

	k2=1	k2=2	k2=3	k2=4	k2=5	k2=6	k2=7	k2=8	k2=9	k2=10
1	0.04	0.10	0.14	0.20	0.36	0.47	0.54	0.66	0.75	0.80
2	0.03	0.17	0.11	0.18	0.33	0.35	0.50	0.65	0.66	0.79
3	0.03	0.07	0.11	0.24	0.29	0.38	0.54	0.58	0.67	0.81
4	0.05	0.16	0.22	0.33	0.46	0.49	0.68	0.75	0.76	0.85
5	0.09	0.15	0.16	0.38	0.41	0.52	0.56	0.73	0.83	0.82
6	0.03	0.11	0.16	0.32	0.33	0.42	0.57	0.69	0.74	0.75
7	0.05	0.05	0.02	0.14	0.16	0.20	0.34	0.36	0.48	0.49
8	0.05	0.06	0.06	0.09	0.22	0.23	0.24	0.37	0.37	0.54
9	0.02	0.04	0.05	0.09	0.18	0.29	0.36	0.30	0.42	0.58
10	0.09	0.19	0.28	0.41	0.49	0.63	0.59	0.70	0.78	0.86
11	0.09	0.18	0.38	0.40	0.55	0.54	0.73	0.72	0.77	0.81
12	0.10	0.21	0.21	0.40	0.46	0.65	0.62	0.69	0.78	0.85
13	0.01	0.03	0.06	0.19	0.29	0.35	0.54	0.68	0.82	0.87
14	0.02	0.01	0.08	0.11	0.23	0.30	0.52	0.69	0.77	0.86
15	0.00	0.02	0.04	0.19	0.29	0.37	0.44	0.60	0.67	0.82
16	0.04	0.05	0.12	0.21	0.30	0.44	0.50	0.69	0.89	0.82
17	0.02	0.06	0.20	0.30	0.34	0.41	0.58	0.79	0.81	0.87
18	0.01	0.05	0.04	0.16	0.25	0.28	0.42	0.51	0.60	0.80
19	0.14	0.08	0.20	0.25	0.45	0.51	0.59	0.78	0.78	0.89
20	0.09	0.13	0.21	0.29	0.40	0.49	0.70	0.67	0.76	0.84
21	0.04	0.11	0.12	0.28	0.29	0.45	0.59	0.74	0.79	0.86
22	0.08	0.14	0.23	0.37	0.48	0.64	0.75	0.84	0.90	0.87
23	0.08	0.21	0.25	0.37	0.48	0.55	0.69	0.87	0.86	0.91
24	0.10	0.16	0.28	0.36	0.43	0.55	0.66	0.78	0.85	0.89
25	0.03	0.07	0.15	0.28	0.43	0.56	0.61	0.79	0.95	0.90
26	0.06	0.06	0.07	0.20	0.30	0.50	0.60	0.69	0.82	0.88
27	0.02	0.05	0.05	0.11	0.22	0.37	0.42	0.44	0.50	0.54
28	0.07	0.15	0.22	0.32	0.53	0.69	0.73	0.77	0.87	0.94
29	0.05	0.04	0.13	0.17	0.33	0.44	0.65	0.75	0.86	0.90
30	0.03	0.06	0.06	0.21	0.23	0.28	0.43	0.59	0.63	0.70
31	0.02	0.05	0.07	0.25	0.26	0.44	0.69	0.82	0.88	0.98
32	0.00	0.07	0.14	0.22	0.34	0.47	0.69	0.82	0.87	0.96
33	0.01	0.01	0.10	0.15	0.34	0.41	0.68	0.77	0.87	0.93
34	0.05	0.06	0.10	0.17	0.25	0.40	0.62	0.79	0.92	0.95
35	0.02	0.07	0.08	0.15	0.33	0.46	0.63	0.74	0.95	0.92
36	0.01	0.05	0.07	0.12	0.22	0.44	0.63	0.80	0.87	0.91
37	0.03	0.05	0.09	0.21	0.45	0.50	0.70	0.89	0.92	0.97
38	0.00	0.06	0.11	0.27	0.32	0.60	0.74	0.83	0.96	0.99
39	0.00	0.05	0.10	0.19	0.39	0.54	0.70	0.89	0.94	0.98
40	0.02	0.08	0.11	0.28	0.47	0.70	0.83	0.91	0.95	0.98
41	0.02	0.04	0.16	0.24	0.34	0.70	0.81	0.83	0.97	0.97
42	0.05	0.04	0.16	0.29	0.43	0.58	0.77	0.85	0.95	0.98

Table 3: POD according to each scenario and each k2 value, k1 = 2, alpha = 0.001

	k2=1	k2=2	k2=3	k2=4	k2=5	k2=6	k2=7	k2=8	k2=9	k2=10
1	0.03	0.10	0.12	0.25	0.39	0.46	0.55	0.65	0.75	0.85
2	0.04	0.10	0.16	0.23	0.32	0.32	0.54	0.59	0.71	0.77
3	0.04	0.07	0.14	0.23	0.27	0.42	0.48	0.65	0.68	0.79
4	0.07	0.16	0.18	0.27	0.48	0.63	0.70	0.67	0.81	0.90
5	0.08	0.10	0.26	0.32	0.38	0.39	0.63	0.80	0.74	0.80
6	0.09	0.11	0.16	0.34	0.29	0.47	0.54	0.60	0.78	0.77
7	0.05	0.06	0.08	0.10	0.19	0.24	0.25	0.38	0.45	0.50
8	0.05	0.06	0.05	0.13	0.13	0.24	0.29	0.30	0.35	0.44
9	0.05	0.03	0.14	0.10	0.10	0.26	0.29	0.51	0.50	0.53
10	0.12	0.15	0.29	0.41	0.49	0.65	0.74	0.77	0.81	0.84
11	0.16	0.22	0.30	0.49	0.59	0.58	0.70	0.71	0.78	0.86
12	0.17	0.16	0.22	0.37	0.57	0.65	0.61	0.76	0.73	0.80
13	0.01	0.03	0.09	0.13	0.31	0.37	0.54	0.59	0.75	0.86
14	0.02	0.00	0.06	0.06	0.21	0.29	0.57	0.63	0.78	0.76
15	0.00	0.03	0.04	0.13	0.17	0.35	0.46	0.61	0.77	0.77
16	0.02	0.10	0.19	0.27	0.25	0.35	0.48	0.71	0.83	0.84
17	0.04	0.08	0.18	0.25	0.23	0.38	0.51	0.67	0.77	0.84
18	0.01	0.02	0.09	0.15	0.16	0.42	0.42	0.51	0.64	0.78
19	0.08	0.19	0.26	0.25	0.36	0.45	0.52	0.69	0.73	0.88
20	0.10	0.17	0.19	0.20	0.39	0.47	0.63	0.73	0.82	0.87
21	0.05	0.13	0.17	0.14	0.29	0.39	0.56	0.67	0.81	0.86
22	0.09	0.24	0.22	0.36	0.41	0.68	0.74	0.82	0.84	0.94
23	0.11	0.10	0.26	0.29	0.48	0.52	0.72	0.85	0.84	0.92
24	0.08	0.11	0.16	0.37	0.45	0.57	0.72	0.75	0.87	0.85
25	0.03	0.11	0.15	0.28	0.39	0.53	0.72	0.81	0.85	0.93
26	0.06	0.08	0.07	0.15	0.31	0.39	0.45	0.79	0.78	0.92
27	0.03	0.02	0.08	0.14	0.14	0.30	0.34	0.48	0.50	0.69
28	0.08	0.13	0.25	0.32	0.36	0.71	0.73	0.86	0.92	0.92
29	0.03	0.06	0.12	0.23	0.39	0.44	0.61	0.77	0.86	0.94
30	0.05	0.04	0.14	0.15	0.17	0.28	0.39	0.53	0.61	0.55
31	0.02	0.04	0.06	0.14	0.28	0.61	0.64	0.74	0.92	0.97
32	0.02	0.03	0.04	0.17	0.35	0.44	0.64	0.82	0.92	1.00
33	0.03	0.01	0.09	0.18	0.30	0.51	0.65	0.79	0.92	0.94
34	0.00	0.09	0.11	0.16	0.22	0.48	0.60	0.70	0.86	0.93
35	0.02	0.05	0.14	0.16	0.40	0.43	0.64	0.77	0.88	0.94
36	0.02	0.02	0.10	0.15	0.26	0.41	0.53	0.73	0.77	0.91
37	0.02	0.04	0.14	0.21	0.38	0.59	0.78	0.87	0.95	0.99
38	0.01	0.02	0.10	0.17	0.38	0.67	0.69	0.86	0.92	0.99
39	0.01	0.07	0.15	0.16	0.35	0.52	0.66	0.87	0.97	0.96
40	0.03	0.06	0.15	0.25	0.39	0.59	0.71	0.91	0.96	0.99
41	0.03	0.05	0.13	0.31	0.32	0.62	0.72	0.87	0.96	1.00
42	0.02	0.09	0.15	0.22	0.39	0.65	0.82	0.92	0.94	0.98

Table 4: POD according to each scenario and each k2 value, k1 = 3, alpha = 0.001

	k2=1	k2=2	k2=3	k2=4	k2=5	k2=6	k2=7	k2=8	k2=9	k2=10
1	0.06	0.05	0.12	0.22	0.31	0.38	0.55	0.65	0.70	0.80
2	0.03	0.06	0.13	0.20	0.26	0.45	0.42	0.63	0.70	0.74
3	0.06	0.08	0.15	0.12	0.27	0.33	0.51	0.58	0.66	0.71
4	0.10	0.12	0.14	0.35	0.42	0.44	0.58	0.74	0.76	0.82
5	0.05	0.10	0.23	0.24	0.40	0.50	0.60	0.72	0.74	0.77
6	0.01	0.08	0.15	0.25	0.30	0.43	0.54	0.54	0.68	0.81
7	0.04	0.04	0.07	0.11	0.22	0.19	0.26	0.29	0.39	0.48
8	0.03	0.04	0.04	0.07	0.14	0.24	0.22	0.29	0.42	0.46
9	0.06	0.06	0.08	0.10	0.15	0.30	0.28	0.40	0.41	0.40
10	0.12	0.20	0.36	0.33	0.49	0.54	0.67	0.72	0.77	0.79
11	0.12	0.17	0.30	0.46	0.54	0.56	0.62	0.76	0.75	0.76
12	0.11	0.25	0.30	0.41	0.43	0.60	0.71	0.68	0.70	0.85
13	0.03	0.05	0.14	0.14	0.17	0.36	0.48	0.62	0.79	0.80
14	0.02	0.04	0.12	0.09	0.28	0.31	0.49	0.66	0.76	0.85
15	0.01	0.03	0.06	0.18	0.18	0.29	0.39	0.52	0.70	0.87
16	0.06	0.05	0.06	0.21	0.27	0.42	0.52	0.69	0.78	0.90
17	0.00	0.02	0.12	0.19	0.32	0.47	0.53	0.61	0.82	0.91
18	0.01	0.07	0.07	0.08	0.23	0.25	0.45	0.54	0.60	0.74
19	0.06	0.14	0.17	0.22	0.31	0.51	0.52	0.63	0.73	0.77
20	0.09	0.07	0.16	0.30	0.35	0.45	0.64	0.69	0.83	0.76
21	0.06	0.11	0.17	0.17	0.40	0.47	0.60	0.57	0.66	0.75
22	0.08	0.13	0.13	0.27	0.43	0.52	0.68	0.74	0.85	0.91
23	0.07	0.14	0.24	0.28	0.44	0.57	0.61	0.84	0.85	0.91
24	0.09	0.12	0.23	0.32	0.35	0.48	0.60	0.73	0.83	0.85
25	0.05	0.09	0.15	0.24	0.31	0.50	0.57	0.83	0.83	0.87
26	0.02	0.06	0.11	0.17	0.25	0.45	0.55	0.71	0.73	0.88
27	0.02	0.04	0.09	0.12	0.14	0.24	0.38	0.57	0.50	0.65
28	0.05	0.18	0.16	0.26	0.35	0.56	0.72	0.77	0.86	0.99
29	0.07	0.10	0.05	0.14	0.35	0.52	0.68	0.81	0.87	0.88
30	0.02	0.04	0.18	0.22	0.23	0.23	0.42	0.45	0.52	0.68
31	0.02	0.01	0.11	0.17	0.27	0.43	0.69	0.77	0.84	0.91
32	0.00	0.04	0.08	0.19	0.35	0.55	0.64	0.74	0.92	0.96
33	0.00	0.06	0.10	0.16	0.24	0.45	0.63	0.80	0.90	0.88
34	0.01	0.03	0.09	0.18	0.24	0.43	0.62	0.78	0.84	0.90
35	0.04	0.05	0.10	0.19	0.31	0.58	0.66	0.71	0.85	0.86
36	0.04	0.06	0.09	0.18	0.36	0.37	0.55	0.69	0.78	0.90
37	0.03	0.00	0.10	0.24	0.32	0.57	0.73	0.87	0.85	0.98
38	0.02	0.08	0.08	0.15	0.46	0.50	0.70	0.83	0.95	0.99
39	0.04	0.03	0.08	0.15	0.36	0.50	0.66	0.82	0.93	0.95
40	0.04	0.09	0.15	0.27	0.31	0.55	0.77	0.91	0.89	0.98
41	0.02	0.06	0.15	0.25	0.37	0.63	0.71	0.85	0.91	0.98
42	0.00	0.01	0.11	0.27	0.35	0.58	0.80	0.84	0.91	0.98

Table 5: POD according to each scenario and each k2 value, k1 = 5, alpha = 0.001

	k2=1	k2=2	k2=3	k2=4	k2=5	k2=6	k2=7	k2=8	k2=9	k2=10
1	0.08	0.07	0.14	0.23	0.26	0.37	0.45	0.46	0.58	0.70
2	0.06	0.07	0.14	0.12	0.28	0.34	0.43	0.52	0.60	0.70
3	0.03	0.07	0.10	0.15	0.21	0.23	0.39	0.48	0.63	0.65
4	0.04	0.05	0.26	0.25	0.38	0.54	0.57	0.68	0.74	0.74
5	0.04	0.10	0.13	0.23	0.36	0.44	0.48	0.56	0.68	0.84
6	0.08	0.05	0.19	0.13	0.25	0.43	0.43	0.56	0.68	0.81
7	0.05	0.06	0.10	0.09	0.07	0.13	0.20	0.23	0.32	0.49
8	0.02	0.05	0.03	0.12	0.24	0.20	0.24	0.29	0.44	0.54
9	0.05	0.07	0.01	0.10	0.13	0.20	0.20	0.31	0.34	0.43
10	0.09	0.18	0.24	0.30	0.43	0.55	0.52	0.68	0.77	0.66
11	0.08	0.15	0.24	0.29	0.41	0.44	0.60	0.65	0.74	0.80
12	0.06	0.16	0.23	0.29	0.40	0.52	0.58	0.70	0.74	0.81
13	0.02	0.00	0.07	0.13	0.18	0.35	0.46	0.53	0.66	0.77
14	0.00	0.01	0.02	0.11	0.24	0.27	0.44	0.59	0.73	0.77
15	0.00	0.00	0.04	0.08	0.17	0.25	0.42	0.47	0.66	0.73
16	0.02	0.01	0.06	0.13	0.25	0.38	0.44	0.61	0.70	0.86
17	0.02	0.03	0.06	0.15	0.21	0.44	0.50	0.61	0.70	0.83
18	0.02	0.02	0.02	0.01	0.15	0.22	0.31	0.50	0.62	0.61
19	0.06	0.09	0.10	0.15	0.28	0.38	0.40	0.53	0.65	0.70
20	0.03	0.09	0.11	0.21	0.26	0.31	0.43	0.61	0.67	0.68
21	0.04	0.09	0.11	0.23	0.28	0.35	0.47	0.60	0.55	0.68
22	0.06	0.15	0.16	0.25	0.27	0.48	0.65	0.71	0.73	0.80
23	0.06	0.08	0.18	0.29	0.33	0.46	0.62	0.71	0.72	0.83
24	0.08	0.07	0.20	0.25	0.35	0.39	0.65	0.68	0.72	0.72
25	0.02	0.05	0.13	0.20	0.29	0.37	0.48	0.67	0.83	0.85
26	0.04	0.03	0.06	0.24	0.13	0.28	0.54	0.70	0.65	0.83
27	0.02	0.02	0.03	0.11	0.14	0.18	0.29	0.37	0.44	0.46
28	0.05	0.05	0.12	0.18	0.41	0.45	0.64	0.74	0.87	0.88
29	0.02	0.03	0.13	0.23	0.37	0.44	0.62	0.71	0.72	0.85
30	0.02	0.07	0.06	0.14	0.08	0.25	0.34	0.48	0.48	0.57
31	0.02	0.02	0.04	0.09	0.30	0.39	0.56	0.80	0.88	0.93
32	0.02	0.01	0.05	0.11	0.26	0.41	0.60	0.77	0.89	0.96
33	0.01	0.03	0.03	0.13	0.29	0.36	0.54	0.75	0.82	0.92
34	0.01	0.04	0.04	0.15	0.34	0.30	0.53	0.70	0.85	0.90
35	0.03	0.02	0.08	0.15	0.28	0.48	0.44	0.58	0.87	0.86
36	0.03	0.05	0.13	0.15	0.28	0.35	0.54	0.69	0.74	0.88
37	0.01	0.01	0.05	0.16	0.35	0.49	0.69	0.78	0.84	0.97
38	0.01	0.05	0.13	0.16	0.27	0.49	0.62	0.83	0.87	0.95
39	0.00	0.03	0.07	0.19	0.28	0.46	0.58	0.79	0.92	0.96
40	0.01	0.03	0.10	0.21	0.36	0.45	0.66	0.76	0.90	0.95
41	0.03	0.05	0.07	0.24	0.39	0.33	0.63	0.85	0.91	0.98
42	0.02	0.06	0.08	0.25	0.31	0.54	0.65	0.87	0.89	0.95

Table 6: POD according to each scenario and each k2 value, k1 = 10, alpha = 0.001

Overall performances of Original Farrington algorithm ($\alpha = 0.01$)

	FPR k1=0	FPR k1=2	FPR k1=3	FPR k1=5	FPR k1=10
1	0.02	0.02	0.02	0.02	0.02
2	0.02	0.02	0.02	0.01	0.01
3	0.02	0.01	0.01	0.01	0.01
4	0.04	0.04	0.04	0.04	0.03
5	0.04	0.04	0.04	0.04	0.03
6	0.04	0.03	0.03	0.03	0.03
7	0.00	0.00	0.00	0.00	0.00
8	0.00	0.00	0.00	0.00	0.00
9	0.00	0.00	0.00	0.00	0.00
10	0.07	0.07	0.06	0.06	0.05
11	0.08	0.07	0.07	0.06	0.06
12	0.06	0.06	0.06	0.06	0.05
13	0.01	0.01	0.01	0.01	0.01
14	0.01	0.01	0.01	0.01	0.00
15	0.01	0.01	0.01	0.01	0.00
16	0.02	0.02	0.02	0.02	0.01
17	0.03	0.03	0.03	0.02	0.02
18	0.02	0.02	0.02	0.02	0.02
19	0.05	0.04	0.04	0.04	0.03
20	0.04	0.04	0.04	0.04	0.03
21	0.03	0.03	0.03	0.03	0.02
22	0.06	0.06	0.05	0.05	0.04
23	0.06	0.05	0.05	0.05	0.04
24	0.05	0.05	0.04	0.04	0.04
25	0.03	0.03	0.03	0.02	0.02
26	0.02	0.02	0.02	0.02	0.02
27	0.01	0.01	0.01	0.01	0.01
28	0.05	0.04	0.04	0.04	0.03
29	0.03	0.03	0.03	0.02	0.02
30	0.02	0.02	0.02	0.02	0.01
31	0.01	0.01	0.01	0.01	0.01
32	0.01	0.01	0.01	0.01	0.01
33	0.01	0.01	0.01	0.01	0.00
34	0.01	0.01	0.01	0.01	0.01
35	0.02	0.02	0.02	0.02	0.01
36	0.02	0.02	0.02	0.02	0.01
37	0.01	0.01	0.01	0.01	0.01
38	0.01	0.01	0.01	0.01	0.01
39	0.01	0.01	0.01	0.01	0.01
40	0.02	0.02	0.02	0.02	0.01
41	0.02	0.02	0.02	0.01	0.01
42	0.02	0.01	0.01	0.01	0.01

Table 7: FPR according to each scenario and each k1 value, alpha = 0.01

	k2=1	k2=2	k2=3	k2=4	k2=5	k2=6	k2=7	k2=8	k2=9	k2=10
1	0.22	0.25	0.27	0.41	0.61	0.70	0.78	0.86	0.96	0.95
2	0.20	0.17	0.20	0.50	0.60	0.66	0.81	0.90	0.90	0.95
3	0.17	0.22	0.23	0.40	0.49	0.54	0.72	0.82	0.89	0.85
4	0.19	0.35	0.42	0.53	0.57	0.75	0.84	0.89	0.89	0.97
5	0.20	0.23	0.44	0.62	0.65	0.70	0.81	0.88	0.97	0.95
6	0.26	0.25	0.30	0.52	0.54	0.73	0.75	0.76	0.92	0.88
7	0.05	0.12	0.10	0.18	0.22	0.35	0.32	0.40	0.56	0.54
8	0.01	0.04	0.06	0.16	0.19	0.34	0.36	0.35	0.51	0.58
9	0.04	0.08	0.07	0.18	0.20	0.32	0.49	0.49	0.52	0.57
10	0.30	0.44	0.44	0.61	0.72	0.77	0.86	0.86	0.90	0.88
11	0.25	0.31	0.50	0.67	0.74	0.76	0.82	0.87	0.90	0.94
12	0.25	0.33	0.57	0.61	0.74	0.80	0.80	0.89	0.87	0.92
13	0.04	0.14	0.17	0.43	0.56	0.71	0.78	0.87	0.96	0.98
14	0.11	0.15	0.29	0.30	0.50	0.68	0.77	0.82	0.92	0.96
15	0.12	0.18	0.19	0.35	0.45	0.69	0.76	0.86	0.90	0.97
16	0.12	0.20	0.28	0.43	0.61	0.78	0.84	0.93	0.96	0.98
17	0.09	0.16	0.34	0.45	0.57	0.77	0.85	0.93	0.93	0.98
18	0.13	0.18	0.25	0.38	0.47	0.53	0.78	0.76	0.83	0.93
19	0.20	0.23	0.41	0.46	0.62	0.74	0.87	0.89	0.96	0.99
20	0.17	0.23	0.33	0.49	0.61	0.75	0.84	0.90	0.95	0.95
21	0.12	0.25	0.29	0.44	0.59	0.77	0.77	0.88	0.98	0.98
22	0.20	0.27	0.44	0.55	0.75	0.81	0.85	0.95	0.96	0.95
23	0.22	0.35	0.43	0.59	0.72	0.83	0.90	0.95	0.95	0.99
24	0.21	0.33	0.45	0.63	0.69	0.75	0.87	0.93	0.95	0.97
25	0.17	0.27	0.42	0.57	0.69	0.83	0.94	0.92	0.98	0.98
26	0.11	0.18	0.33	0.43	0.64	0.75	0.86	0.93	0.93	0.96
27	0.11	0.12	0.21	0.26	0.38	0.44	0.63	0.69	0.71	0.80
28	0.17	0.38	0.48	0.62	0.73	0.87	0.91	0.94	0.97	1.00
29	0.15	0.27	0.33	0.55	0.64	0.77	0.79	0.97	0.98	0.98
30	0.05	0.20	0.20	0.31	0.37	0.53	0.53	0.70	0.75	0.83
31	0.10	0.15	0.24	0.42	0.62	0.80	0.93	0.96	0.98	1.00
32	0.08	0.09	0.34	0.47	0.55	0.86	0.82	0.95	0.98	1.00
33	0.05	0.13	0.26	0.47	0.63	0.80	0.91	0.94	0.98	1.00
34	0.08	0.14	0.30	0.42	0.70	0.73	0.87	0.92	0.99	0.99
35	0.15	0.16	0.23	0.45	0.53	0.76	0.85	0.95	0.97	0.99
36	0.12	0.19	0.27	0.40	0.68	0.71	0.87	0.94	0.98	0.99
37	0.07	0.17	0.27	0.56	0.65	0.76	0.90	0.95	0.99	0.99
38	0.07	0.17	0.35	0.46	0.71	0.83	0.95	0.99	1.00	1.00
39	0.05	0.17	0.40	0.48	0.68	0.87	0.88	0.98	0.99	1.00
40	0.11	0.24	0.41	0.63	0.70	0.88	0.94	0.98	0.99	1.00
41	0.14	0.29	0.32	0.56	0.74	0.84	0.92	0.99	0.99	0.99
42	0.10	0.21	0.34	0.50	0.72	0.86	0.91	0.95	1.00	1.00

Table 8: POD according to each scenario and each k2 value, k1 = 0, alpha = 0.01

	k2=1	k2=2	k2=3	k2=4	k2=5	k2=6	k2=7	k2=8	k2=9	k2=10
1	0.13	0.22	0.33	0.38	0.60	0.65	0.74	0.85	0.84	0.92
2	0.08	0.29	0.29	0.40	0.60	0.68	0.74	0.79	0.79	0.95
3	0.09	0.22	0.29	0.42	0.45	0.61	0.76	0.79	0.84	0.93
4	0.24	0.30	0.44	0.51	0.63	0.69	0.84	0.89	0.89	0.95
5	0.23	0.32	0.40	0.55	0.66	0.72	0.80	0.90	0.95	0.95
6	0.10	0.33	0.42	0.50	0.57	0.62	0.72	0.89	0.85	0.89
7	0.05	0.06	0.04	0.20	0.23	0.23	0.39	0.42	0.56	0.57
8	0.07	0.09	0.08	0.12	0.28	0.27	0.28	0.40	0.44	0.65
9	0.03	0.05	0.08	0.12	0.22	0.35	0.41	0.31	0.51	0.67
10	0.25	0.36	0.50	0.62	0.70	0.78	0.71	0.80	0.87	0.93
11	0.17	0.35	0.51	0.49	0.71	0.73	0.86	0.85	0.88	0.93
12	0.18	0.30	0.38	0.57	0.68	0.77	0.82	0.89	0.85	0.93
13	0.06	0.10	0.25	0.45	0.55	0.59	0.82	0.86	0.94	0.95
14	0.05	0.11	0.24	0.34	0.41	0.65	0.77	0.88	0.94	0.95
15	0.11	0.15	0.24	0.36	0.63	0.68	0.76	0.87	0.88	0.91
16	0.12	0.20	0.33	0.42	0.62	0.71	0.81	0.90	0.97	0.95
17	0.10	0.21	0.31	0.51	0.63	0.76	0.86	0.91	0.97	0.97
18	0.07	0.13	0.19	0.31	0.48	0.57	0.69	0.77	0.80	0.95
19	0.17	0.18	0.39	0.56	0.67	0.74	0.81	0.93	0.96	0.98
20	0.14	0.25	0.34	0.59	0.63	0.81	0.88	0.86	0.92	0.97
21	0.07	0.16	0.28	0.47	0.50	0.67	0.82	0.89	0.91	0.95
22	0.14	0.26	0.44	0.65	0.72	0.81	0.90	0.96	0.96	0.93
23	0.14	0.32	0.43	0.66	0.73	0.88	0.85	0.97	0.93	0.97
24	0.21	0.27	0.43	0.64	0.72	0.78	0.87	0.93	0.93	0.97
25	0.14	0.24	0.30	0.58	0.72	0.78	0.87	0.97	1.00	1.00
26	0.11	0.24	0.31	0.40	0.62	0.78	0.84	0.87	0.95	0.98
27	0.07	0.11	0.16	0.26	0.42	0.53	0.60	0.68	0.74	0.75
28	0.17	0.33	0.35	0.59	0.77	0.86	0.89	0.93	0.96	0.99
29	0.14	0.16	0.34	0.41	0.63	0.71	0.91	0.92	0.96	0.98
30	0.10	0.16	0.21	0.42	0.53	0.55	0.60	0.73	0.73	0.81
31	0.09	0.15	0.25	0.49	0.62	0.85	0.89	0.96	0.95	1.00
32	0.02	0.20	0.32	0.40	0.57	0.77	0.89	0.95	0.95	0.99
33	0.03	0.10	0.26	0.38	0.70	0.69	0.89	0.94	0.97	0.99
34	0.09	0.17	0.26	0.42	0.57	0.74	0.83	0.98	0.98	0.99
35	0.09	0.18	0.21	0.35	0.57	0.71	0.89	0.93	0.97	0.99
36	0.07	0.15	0.22	0.36	0.49	0.71	0.87	0.94	0.97	0.98
37	0.13	0.20	0.31	0.44	0.76	0.74	0.89	0.96	0.99	0.98
38	0.03	0.16	0.35	0.55	0.62	0.86	0.92	0.97	1.00	1.00
39	0.06	0.24	0.25	0.51	0.70	0.84	0.92	0.94	1.00	1.00
40	0.10	0.32	0.44	0.49	0.81	0.88	0.96	0.97	0.98	1.00
41	0.14	0.10	0.40	0.53	0.66	0.93	0.95	0.96	1.00	1.00
42	0.12	0.20	0.34	0.51	0.67	0.80	0.95	0.97	1.00	0.99

Table 9: POD according to each scenario and each k2 value, k1 = 2, alpha = 0.01

	k2=1	k2=2	k2=3	k2=4	k2=5	k2=6	k2=7	k2=8	k2=9	k2=10
1	0.15	0.21	0.30	0.47	0.65	0.68	0.75	0.87	0.86	0.89
2	0.12	0.25	0.34	0.40	0.53	0.62	0.82	0.79	0.84	0.91
3	0.09	0.16	0.24	0.37	0.46	0.62	0.75	0.83	0.86	0.93
4	0.19	0.29	0.39	0.52	0.62	0.78	0.84	0.87	0.92	0.93
5	0.20	0.31	0.44	0.49	0.60	0.68	0.76	0.90	0.91	0.95
6	0.18	0.25	0.35	0.51	0.51	0.66	0.76	0.84	0.89	0.87
7	0.05	0.08	0.13	0.13	0.20	0.26	0.32	0.42	0.53	0.55
8	0.08	0.08	0.09	0.17	0.15	0.29	0.38	0.35	0.40	0.55
9	0.05	0.05	0.17	0.12	0.13	0.32	0.35	0.56	0.54	0.59
10	0.24	0.28	0.49	0.63	0.64	0.78	0.88	0.86	0.91	0.90
11	0.34	0.36	0.48	0.72	0.74	0.76	0.81	0.84	0.87	0.96
12	0.25	0.30	0.43	0.53	0.74	0.75	0.70	0.83	0.86	0.89
13	0.08	0.14	0.22	0.37	0.56	0.65	0.75	0.85	0.91	0.97
14	0.13	0.07	0.29	0.37	0.52	0.66	0.74	0.82	0.90	0.93
15	0.05	0.11	0.34	0.31	0.41	0.67	0.69	0.85	0.87	0.94
16	0.09	0.17	0.29	0.51	0.53	0.66	0.74	0.92	0.95	0.99
17	0.11	0.19	0.37	0.50	0.57	0.69	0.77	0.89	0.94	0.98
18	0.08	0.10	0.26	0.37	0.38	0.63	0.69	0.81	0.84	0.93
19	0.10	0.31	0.39	0.50	0.55	0.72	0.80	0.85	0.88	0.96
20	0.14	0.29	0.35	0.39	0.61	0.74	0.80	0.91	0.95	0.99
21	0.08	0.26	0.36	0.37	0.51	0.66	0.79	0.89	0.94	0.96
22	0.10	0.39	0.46	0.63	0.67	0.81	0.90	0.95	0.93	0.98
23	0.18	0.24	0.42	0.52	0.78	0.78	0.90	0.92	0.93	0.97
24	0.13	0.21	0.39	0.60	0.73	0.77	0.89	0.93	0.95	0.94
25	0.11	0.23	0.27	0.61	0.65	0.80	0.91	0.95	0.96	0.99
26	0.11	0.18	0.26	0.41	0.53	0.73	0.81	0.92	0.96	0.99
27	0.07	0.06	0.18	0.29	0.28	0.52	0.63	0.69	0.72	0.83
28	0.22	0.35	0.44	0.58	0.66	0.88	0.91	0.95	0.98	1.00
29	0.16	0.19	0.40	0.49	0.68	0.80	0.79	0.92	0.97	0.99
30	0.12	0.15	0.23	0.30	0.29	0.53	0.64	0.68	0.72	0.74
31	0.07	0.17	0.21	0.40	0.57	0.87	0.88	0.96	0.98	1.00
32	0.05	0.15	0.26	0.38	0.62	0.77	0.88	0.96	0.97	1.00
33	0.07	0.16	0.26	0.40	0.57	0.83	0.90	0.97	1.00	0.99
34	0.08	0.14	0.21	0.34	0.50	0.72	0.83	0.91	0.96	0.99
35	0.08	0.18	0.27	0.40	0.65	0.68	0.89	0.92	0.94	1.00
36	0.11	0.11	0.30	0.33	0.58	0.68	0.84	0.94	0.92	0.99
37	0.08	0.17	0.34	0.47	0.71	0.84	0.94	0.94	0.96	1.00
38	0.08	0.12	0.26	0.52	0.61	0.85	0.90	0.96	0.99	1.00
39	0.09	0.18	0.37	0.50	0.64	0.80	0.88	0.96	0.99	1.00
40	0.11	0.23	0.36	0.53	0.73	0.85	0.91	0.97	1.00	1.00
41	0.06	0.15	0.31	0.55	0.59	0.85	0.94	0.97	0.99	1.00
42	0.07	0.24	0.40	0.53	0.73	0.87	0.95	0.99	1.00	1.00

Table 10: POD according to each scenario and each k2 value, k1 = 3, alpha = 0.01

	k2=1	k2=2	k2=3	k2=4	k2=5	k2=6	k2=7	k2=8	k2=9	k2=10
1	0.14	0.19	0.28	0.43	0.51	0.56	0.78	0.90	0.90	0.92
2	0.12	0.17	0.27	0.38	0.47	0.65	0.69	0.79	0.83	0.87
3	0.09	0.15	0.24	0.29	0.51	0.51	0.70	0.77	0.82	0.86
4	0.22	0.29	0.32	0.55	0.62	0.68	0.74	0.87	0.93	0.95
5	0.13	0.24	0.42	0.43	0.66	0.69	0.74	0.85	0.88	0.89
6	0.11	0.19	0.33	0.56	0.53	0.72	0.66	0.78	0.85	0.90
7	0.07	0.05	0.08	0.14	0.24	0.23	0.31	0.33	0.49	0.58
8	0.04	0.06	0.06	0.14	0.20	0.30	0.30	0.40	0.48	0.53
9	0.06	0.08	0.10	0.13	0.22	0.36	0.34	0.45	0.49	0.48
10	0.21	0.33	0.52	0.53	0.66	0.66	0.82	0.86	0.83	0.88
11	0.24	0.33	0.45	0.61	0.71	0.76	0.72	0.86	0.88	0.86
12	0.23	0.42	0.45	0.58	0.60	0.76	0.77	0.90	0.83	0.95
13	0.06	0.13	0.28	0.40	0.51	0.67	0.75	0.82	0.93	0.95
14	0.03	0.13	0.24	0.35	0.55	0.63	0.73	0.89	0.93	0.96
15	0.09	0.10	0.27	0.38	0.54	0.64	0.70	0.81	0.88	0.99
16	0.13	0.11	0.25	0.40	0.58	0.71	0.74	0.92	0.93	0.97
17	0.10	0.11	0.33	0.41	0.58	0.68	0.76	0.84	0.95	0.98
18	0.03	0.12	0.22	0.28	0.43	0.51	0.71	0.77	0.81	0.93
19	0.11	0.28	0.32	0.43	0.56	0.77	0.76	0.84	0.87	0.95
20	0.12	0.19	0.28	0.45	0.57	0.70	0.88	0.87	0.95	0.94
21	0.10	0.17	0.27	0.40	0.57	0.71	0.78	0.78	0.87	0.93
22	0.15	0.29	0.32	0.53	0.69	0.74	0.82	0.86	0.99	0.96
23	0.15	0.30	0.44	0.55	0.69	0.77	0.83	0.89	0.93	0.99
24	0.18	0.21	0.41	0.58	0.64	0.73	0.85	0.91	0.98	0.98
25	0.09	0.20	0.27	0.50	0.60	0.75	0.73	0.99	0.96	0.98
26	0.08	0.15	0.32	0.41	0.63	0.73	0.76	0.90	0.95	0.97
27	0.07	0.10	0.18	0.30	0.35	0.41	0.55	0.78	0.70	0.81
28	0.15	0.32	0.29	0.50	0.63	0.79	0.86	0.89	0.96	1.00
29	0.17	0.21	0.26	0.42	0.58	0.75	0.89	0.93	0.98	0.95
30	0.06	0.14	0.26	0.32	0.41	0.39	0.58	0.66	0.68	0.79
31	0.11	0.12	0.34	0.36	0.52	0.70	0.89	0.92	0.98	0.99
32	0.05	0.11	0.31	0.50	0.62	0.76	0.86	0.93	0.98	0.99
33	0.02	0.15	0.26	0.41	0.65	0.77	0.87	0.94	0.99	0.98
34	0.06	0.07	0.22	0.39	0.54	0.68	0.90	0.93	0.94	0.98
35	0.09	0.17	0.22	0.41	0.51	0.81	0.85	0.90	0.97	0.97
36	0.05	0.12	0.27	0.41	0.51	0.67	0.74	0.87	0.91	0.99
37	0.07	0.11	0.35	0.46	0.67	0.88	0.91	0.97	0.95	0.99
38	0.05	0.17	0.24	0.38	0.68	0.84	0.94	0.97	0.99	1.00
39	0.09	0.11	0.31	0.36	0.62	0.82	0.91	0.96	0.98	1.00
40	0.12	0.21	0.38	0.49	0.67	0.79	0.91	0.96	0.98	1.00
41	0.09	0.16	0.39	0.54	0.58	0.81	0.90	0.95	0.98	1.00
42	0.12	0.13	0.33	0.58	0.69	0.84	0.93	0.96	0.99	1.00

Table 11: POD according to each scenario and each k2 value, k1 = 5, alpha = 0.01

	k2=1	k2=2	k2=3	k2=4	k2=5	k2=6	k2=7	k2=8	k2=9	k2=10
1	0.20	0.16	0.30	0.44	0.46	0.56	0.69	0.69	0.73	0.84
2	0.13	0.18	0.24	0.32	0.48	0.55	0.62	0.77	0.85	0.86
3	0.11	0.14	0.22	0.29	0.35	0.41	0.59	0.68	0.82	0.87
4	0.13	0.20	0.40	0.46	0.60	0.71	0.74	0.86	0.90	0.88
5	0.11	0.22	0.32	0.39	0.56	0.68	0.73	0.79	0.84	0.94
6	0.14	0.14	0.36	0.36	0.47	0.60	0.66	0.78	0.86	0.91
7	0.06	0.07	0.10	0.16	0.14	0.18	0.29	0.32	0.46	0.55
8	0.04	0.07	0.06	0.15	0.26	0.23	0.35	0.35	0.52	0.60
9	0.06	0.08	0.03	0.13	0.19	0.28	0.27	0.40	0.42	0.53
10	0.17	0.29	0.49	0.49	0.59	0.71	0.75	0.81	0.89	0.84
11	0.14	0.32	0.43	0.50	0.57	0.63	0.74	0.78	0.87	0.87
12	0.21	0.29	0.41	0.50	0.60	0.73	0.71	0.87	0.88	0.90
13	0.05	0.08	0.23	0.34	0.48	0.58	0.71	0.81	0.92	0.98
14	0.03	0.05	0.26	0.25	0.49	0.53	0.72	0.80	0.92	0.93
15	0.05	0.07	0.23	0.24	0.45	0.51	0.69	0.73	0.89	0.90
16	0.04	0.10	0.24	0.24	0.52	0.64	0.76	0.83	0.88	0.98
17	0.08	0.14	0.16	0.27	0.48	0.62	0.78	0.83	0.88	0.96
18	0.08	0.12	0.15	0.26	0.35	0.41	0.61	0.76	0.82	0.85
19	0.10	0.21	0.20	0.32	0.51	0.67	0.64	0.76	0.83	0.88
20	0.10	0.18	0.16	0.39	0.44	0.50	0.65	0.81	0.91	0.91
21	0.09	0.11	0.28	0.34	0.39	0.57	0.66	0.80	0.81	0.88
22	0.15	0.29	0.27	0.41	0.52	0.72	0.78	0.85	0.91	0.93
23	0.11	0.14	0.36	0.45	0.57	0.63	0.82	0.87	0.91	0.93
24	0.14	0.15	0.36	0.50	0.56	0.60	0.85	0.80	0.89	0.89
25	0.07	0.16	0.26	0.42	0.53	0.56	0.76	0.85	0.93	0.98
26	0.14	0.17	0.25	0.40	0.39	0.55	0.77	0.87	0.93	0.95
27	0.02	0.06	0.10	0.23	0.27	0.37	0.49	0.65	0.68	0.70
28	0.14	0.16	0.31	0.45	0.62	0.67	0.83	0.94	0.94	0.94
29	0.09	0.12	0.36	0.53	0.66	0.74	0.80	0.84	0.88	0.96
30	0.07	0.13	0.16	0.30	0.24	0.38	0.49	0.65	0.72	0.72
31	0.10	0.12	0.27	0.36	0.55	0.63	0.84	0.97	1.00	1.00
32	0.08	0.16	0.25	0.31	0.47	0.78	0.84	0.96	0.99	0.99
33	0.05	0.09	0.10	0.33	0.51	0.68	0.80	0.89	0.93	0.97
34	0.03	0.10	0.18	0.37	0.57	0.62	0.80	0.87	0.98	1.00
35	0.07	0.10	0.22	0.35	0.58	0.71	0.72	0.84	0.93	0.99
36	0.09	0.13	0.29	0.36	0.56	0.61	0.80	0.88	0.93	0.96
37	0.04	0.07	0.21	0.36	0.64	0.77	0.91	0.92	0.92	1.00
38	0.06	0.14	0.34	0.40	0.55	0.74	0.89	0.94	0.98	0.99
39	0.03	0.11	0.25	0.37	0.62	0.75	0.89	0.97	0.98	1.00
40	0.08	0.16	0.27	0.54	0.63	0.82	0.88	0.91	0.98	1.00
41	0.07	0.16	0.20	0.50	0.71	0.65	0.86	0.95	0.98	0.99
42	0.06	0.16	0.26	0.53	0.66	0.80	0.88	0.99	0.98	0.98

Table 12: POD according to each scenario and each k2 value, k1 = 10, alpha = 0.01

Overall performances of Original Farrington algorithm ($\alpha = 0.05$)

	FPR k1=0	FPR k1=2	FPR k1=3	FPR k1=5	FPR k1=10
1	0.05	0.05	0.05	0.04	0.04
2	0.04	0.04	0.04	0.03	0.03
3	0.04	0.03	0.03	0.03	0.03
4	0.10	0.10	0.10	0.09	0.08
5	0.09	0.09	0.08	0.08	0.07
6	0.08	0.08	0.07	0.07	0.06
7	0.00	0.00	0.00	0.00	0.00
8	0.00	0.00	0.00	0.00	0.00
9	0.00	0.00	0.00	0.00	0.00
10	0.13	0.12	0.12	0.12	0.10
11	0.14	0.13	0.13	0.12	0.11
12	0.12	0.12	0.11	0.11	0.10
13	0.04	0.04	0.03	0.03	0.03
14	0.03	0.03	0.03	0.03	0.02
15	0.03	0.03	0.03	0.03	0.03
16	0.08	0.07	0.07	0.06	0.05
17	0.08	0.08	0.07	0.07	0.06
18	0.06	0.06	0.06	0.05	0.04
19	0.08	0.07	0.07	0.07	0.06
20	0.08	0.07	0.07	0.06	0.06
21	0.06	0.06	0.05	0.05	0.04
22	0.11	0.10	0.10	0.09	0.07
23	0.10	0.09	0.09	0.09	0.08
24	0.10	0.09	0.09	0.08	0.07
25	0.07	0.07	0.06	0.06	0.05
26	0.06	0.05	0.05	0.05	0.04
27	0.04	0.03	0.03	0.03	0.03
28	0.10	0.10	0.10	0.09	0.08
29	0.08	0.07	0.07	0.06	0.06
30	0.05	0.05	0.05	0.04	0.04
31	0.04	0.04	0.04	0.04	0.03
32	0.04	0.04	0.04	0.04	0.03
33	0.03	0.03	0.03	0.03	0.02
34	0.05	0.04	0.05	0.05	0.04
35	0.05	0.05	0.05	0.05	0.05
36	0.05	0.05	0.05	0.05	0.05
37	0.05	0.04	0.04	0.04	0.03
38	0.05	0.04	0.04	0.04	0.03
39	0.04	0.04	0.04	0.04	0.04
40	0.06	0.06	0.06	0.06	0.05
41	0.06	0.05	0.05	0.05	0.04
42	0.06	0.05	0.05	0.05	0.05

Table 13: FPR according to each scenario and each k1 value, alpha = 0.05

	k2=1	k2=2	k2=3	k2=4	k2=5	k2=6	k2=7	k2=8	k2=9	k2=10
1	0.38	0.46	0.52	0.62	0.79	0.84	0.93	0.95	0.99	0.97
2	0.31	0.32	0.40	0.77	0.75	0.79	0.90	0.96	0.95	0.98
3	0.27	0.38	0.37	0.63	0.57	0.75	0.88	0.86	0.98	0.95
4	0.35	0.51	0.58	0.71	0.80	0.87	0.95	0.97	0.96	0.99
5	0.37	0.39	0.63	0.78	0.80	0.82	0.93	0.96	0.98	1.00
6	0.41	0.49	0.49	0.73	0.77	0.89	0.90	0.91	0.99	0.95
7	0.05	0.13	0.16	0.24	0.24	0.39	0.37	0.44	0.62	0.58
8	0.01	0.05	0.06	0.19	0.24	0.37	0.44	0.43	0.54	0.62
9	0.04	0.10	0.10	0.20	0.21	0.35	0.54	0.57	0.54	0.59
10	0.49	0.60	0.63	0.81	0.82	0.89	0.90	0.91	0.97	0.95
11	0.38	0.48	0.62	0.74	0.85	0.88	0.91	0.97	0.93	0.99
12	0.31	0.53	0.69	0.80	0.87	0.94	0.90	0.95	0.95	0.99
13	0.18	0.34	0.49	0.71	0.80	0.89	0.90	0.97	0.99	1.00
14	0.29	0.29	0.54	0.60	0.66	0.88	0.91	0.96	0.97	0.98
15	0.26	0.40	0.40	0.58	0.73	0.84	0.95	0.96	0.97	1.00
16	0.32	0.43	0.54	0.66	0.79	0.96	0.94	0.99	1.00	0.99
17	0.25	0.36	0.57	0.70	0.83	0.90	0.96	0.99	0.98	1.00
18	0.27	0.33	0.39	0.61	0.74	0.82	0.96	0.92	0.97	1.00
19	0.27	0.36	0.58	0.70	0.84	0.92	0.96	0.99	1.00	0.99
20	0.31	0.39	0.52	0.72	0.84	0.91	0.95	0.98	0.99	1.00
21	0.17	0.35	0.46	0.61	0.77	0.87	0.91	0.96	1.00	1.00
22	0.34	0.50	0.77	0.79	0.88	0.91	0.96	0.99	1.00	0.99
23	0.38	0.56	0.67	0.78	0.85	0.91	0.98	0.96	0.97	0.99
24	0.33	0.51	0.73	0.78	0.86	0.89	0.96	0.97	0.96	1.00
25	0.33	0.47	0.66	0.78	0.86	0.95	0.99	0.99	0.99	1.00
26	0.22	0.36	0.64	0.69	0.84	0.93	0.95	1.00	0.97	0.99
27	0.15	0.29	0.31	0.56	0.66	0.69	0.86	0.84	0.90	0.93
28	0.36	0.68	0.76	0.81	0.90	0.93	0.99	0.99	1.00	1.00
29	0.33	0.43	0.63	0.73	0.85	0.89	0.92	1.00	1.00	1.00
30	0.14	0.39	0.37	0.54	0.53	0.74	0.74	0.86	0.93	0.98
31	0.18	0.42	0.47	0.69	0.85	1.00	1.00	0.99	0.99	1.00
32	0.22	0.37	0.61	0.72	0.82	0.97	0.97	1.00	1.00	1.00
33	0.17	0.31	0.50	0.76	0.87	0.94	0.97	0.99	1.00	1.00
34	0.18	0.33	0.53	0.67	0.86	0.92	0.94	0.99	0.99	1.00
35	0.29	0.36	0.49	0.74	0.79	0.93	0.95	1.00	1.00	0.99
36	0.20	0.37	0.49	0.69	0.88	0.89	0.97	0.99	1.00	1.00
37	0.14	0.44	0.60	0.77	0.86	0.94	0.99	1.00	0.99	1.00
38	0.27	0.34	0.62	0.77	0.85	0.94	1.00	1.00	1.00	1.00
39	0.16	0.39	0.58	0.81	0.93	0.98	0.97	1.00	0.99	1.00
40	0.32	0.50	0.60	0.90	0.91	0.94	0.98	1.00	1.00	1.00
41	0.34	0.49	0.62	0.83	0.89	0.94	0.98	1.00	1.00	1.00
42	0.31	0.46	0.60	0.71	0.88	0.96	1.00	0.97	1.00	1.00

Table 14: POD according to each scenario and each k2 value, k1 = 0, alpha = 0.05

	k2=1	k2=2	k2=3	k2=4	k2=5	k2=6	k2=7	k2=8	k2=9	k2=10
1	0.30	0.42	0.61	0.57	0.76	0.86	0.93	0.97	0.94	0.96
2	0.26	0.45	0.51	0.63	0.83	0.83	0.87	0.91	0.92	0.95
3	0.22	0.35	0.46	0.60	0.64	0.80	0.92	0.88	0.93	0.95
4	0.42	0.51	0.62	0.75	0.77	0.85	0.96	0.96	0.98	0.99
5	0.36	0.46	0.57	0.70	0.82	0.88	0.93	0.96	0.99	0.98
6	0.26	0.48	0.60	0.66	0.76	0.81	0.86	0.94	0.94	0.99
7	0.05	0.06	0.06	0.23	0.27	0.29	0.41	0.46	0.58	0.62
8	0.08	0.09	0.08	0.15	0.33	0.35	0.37	0.44	0.52	0.68
9	0.03	0.10	0.09	0.18	0.27	0.39	0.44	0.38	0.57	0.70
10	0.40	0.51	0.66	0.80	0.81	0.90	0.87	0.91	0.92	0.97
11	0.38	0.50	0.69	0.63	0.80	0.82	0.90	0.93	0.95	0.98
12	0.45	0.52	0.51	0.69	0.80	0.86	0.91	0.97	0.96	0.99
13	0.19	0.35	0.53	0.70	0.80	0.80	0.95	0.97	1.00	0.99
14	0.19	0.29	0.55	0.61	0.72	0.79	0.92	0.96	0.99	0.99
15	0.24	0.30	0.41	0.65	0.88	0.90	0.91	0.97	0.97	0.98
16	0.28	0.40	0.55	0.63	0.82	0.91	0.94	0.98	0.99	0.99
17	0.31	0.33	0.61	0.70	0.86	0.90	0.96	0.95	1.00	0.99
18	0.21	0.29	0.40	0.54	0.71	0.78	0.87	0.95	0.96	1.00
19	0.25	0.42	0.58	0.77	0.87	0.96	0.98	0.97	1.00	0.99
20	0.24	0.36	0.54	0.75	0.82	0.94	0.96	0.95	0.98	0.99
21	0.18	0.27	0.44	0.63	0.77	0.82	0.95	0.97	0.99	0.99
22	0.31	0.46	0.64	0.82	0.88	0.94	0.95	0.97	0.98	0.99
23	0.34	0.46	0.68	0.85	0.90	0.97	0.96	0.99	0.99	1.00
24	0.37	0.45	0.69	0.82	0.82	0.91	0.97	0.96	0.98	1.00
25	0.25	0.39	0.53	0.83	0.88	0.88	0.96	1.00	1.00	1.00
26	0.19	0.41	0.54	0.65	0.86	0.91	0.98	0.97	1.00	1.00
27	0.10	0.19	0.37	0.53	0.56	0.76	0.80	0.83	0.90	0.94
28	0.32	0.54	0.62	0.83	0.88	0.94	0.99	1.00	0.99	0.99
29	0.28	0.41	0.51	0.71	0.86	0.90	0.97	0.99	0.98	1.00
30	0.17	0.31	0.32	0.56	0.65	0.76	0.81	0.91	0.88	0.96
31	0.27	0.31	0.50	0.71	0.84	0.95	0.98	1.00	0.99	1.00
32	0.18	0.40	0.59	0.72	0.86	0.91	0.96	0.99	0.98	0.99
33	0.11	0.35	0.52	0.71	0.90	0.90	0.96	0.99	1.00	1.00
34	0.24	0.36	0.46	0.72	0.85	0.94	0.94	0.98	1.00	1.00
35	0.25	0.35	0.41	0.62	0.82	0.86	0.97	0.99	0.99	1.00
36	0.17	0.31	0.49	0.62	0.73	0.88	0.97	0.99	1.00	1.00
37	0.26	0.40	0.60	0.75	0.94	0.95	0.99	1.00	1.00	1.00
38	0.18	0.35	0.63	0.75	0.85	0.97	0.99	1.00	1.00	1.00
39	0.14	0.48	0.49	0.79	0.90	0.92	1.00	1.00	1.00	1.00
40	0.30	0.59	0.62	0.83	0.96	0.95	0.99	0.99	1.00	1.00
41	0.27	0.35	0.62	0.83	0.87	0.98	0.98	1.00	1.00	1.00
42	0.28	0.45	0.60	0.77	0.86	0.95	0.99	0.99	1.00	1.00

Table 15: POD according to each scenario and each k2 value, k1 = 2, alpha = 0.05

	k2=1	k2=2	k2=3	k2=4	k2=5	k2=6	k2=7	k2=8	k2=9	k2=10
1	0.29	0.38	0.52	0.66	0.84	0.82	0.90	0.96	0.94	0.96
2	0.27	0.45	0.52	0.57	0.77	0.85	0.92	0.87	0.95	0.94
3	0.22	0.32	0.46	0.52	0.67	0.73	0.82	0.90	0.94	0.95
4	0.37	0.43	0.52	0.70	0.81	0.88	0.92	0.94	0.96	0.96
5	0.33	0.54	0.63	0.71	0.76	0.89	0.91	0.99	0.98	0.99
6	0.32	0.41	0.56	0.63	0.73	0.85	0.92	0.95	0.95	0.95
7	0.06	0.09	0.15	0.17	0.23	0.31	0.40	0.44	0.58	0.60
8	0.09	0.10	0.11	0.19	0.22	0.33	0.46	0.38	0.45	0.58
9	0.07	0.07	0.20	0.16	0.19	0.37	0.39	0.61	0.60	0.65
10	0.40	0.53	0.65	0.71	0.78	0.91	0.98	0.93	0.94	0.94
11	0.48	0.54	0.64	0.83	0.84	0.88	0.89	0.92	0.98	0.98
12	0.43	0.41	0.62	0.72	0.84	0.85	0.83	0.92	0.94	0.96
13	0.22	0.35	0.45	0.61	0.76	0.85	0.94	0.94	0.98	0.99
14	0.26	0.30	0.51	0.62	0.77	0.87	0.92	0.98	0.97	0.98
15	0.15	0.28	0.57	0.59	0.64	0.86	0.92	0.96	0.99	0.98
16	0.21	0.35	0.54	0.81	0.78	0.88	0.95	0.97	0.99	1.00
17	0.31	0.37	0.52	0.66	0.79	0.86	0.93	0.99	0.99	0.99
18	0.16	0.29	0.49	0.53	0.68	0.80	0.92	0.90	0.98	1.00
19	0.24	0.45	0.58	0.69	0.76	0.88	0.94	0.97	0.99	1.00
20	0.18	0.44	0.52	0.61	0.78	0.89	0.97	0.95	0.97	1.00
21	0.19	0.40	0.58	0.63	0.73	0.89	0.90	0.98	0.99	0.98
22	0.23	0.49	0.65	0.86	0.85	0.94	0.96	0.96	0.98	0.98
23	0.26	0.49	0.68	0.74	0.88	0.88	0.99	0.98	0.97	0.98
24	0.26	0.45	0.61	0.78	0.87	0.94	0.97	0.98	0.97	0.99
25	0.23	0.40	0.60	0.79	0.83	0.94	0.98	1.00	0.99	1.00
26	0.22	0.33	0.48	0.65	0.81	0.90	0.95	0.99	1.00	1.00
27	0.14	0.23	0.34	0.46	0.56	0.70	0.81	0.88	0.87	0.93
28	0.34	0.47	0.71	0.76	0.89	0.96	0.96	0.99	0.99	1.00
29	0.32	0.43	0.66	0.73	0.86	0.93	0.93	1.00	1.00	1.00
30	0.20	0.33	0.41	0.56	0.58	0.72	0.75	0.84	0.92	0.93
31	0.23	0.42	0.51	0.65	0.79	0.99	0.97	1.00	0.99	1.00
32	0.14	0.35	0.54	0.68	0.80	0.86	0.94	0.99	1.00	1.00
33	0.24	0.32	0.51	0.75	0.78	0.92	0.98	0.99	1.00	1.00
34	0.17	0.29	0.54	0.62	0.78	0.91	0.96	0.96	1.00	1.00
35	0.25	0.39	0.55	0.66	0.85	0.88	0.97	0.97	0.99	1.00
36	0.20	0.34	0.48	0.65	0.81	0.88	0.97	0.98	0.98	1.00
37	0.26	0.46	0.68	0.70	0.83	0.93	1.00	0.98	1.00	1.00
38	0.25	0.36	0.53	0.77	0.83	0.93	0.96	0.98	1.00	1.00
39	0.16	0.40	0.61	0.75	0.88	0.94	0.98	0.99	1.00	1.00
40	0.35	0.54	0.62	0.81	0.90	0.97	0.99	1.00	1.00	1.00
41	0.24	0.39	0.52	0.81	0.85	0.97	0.98	0.99	0.99	1.00
42	0.27	0.57	0.61	0.75	0.90	0.93	0.99	1.00	1.00	1.00

Table 16: POD according to each scenario and each k2 value, k1 = 3, alpha = 0.05

	k2=1	k2=2	k2=3	k2=4	k2=5	k2=6	k2=7	k2=8	k2=9	k2=10
1	0.32	0.37	0.55	0.63	0.74	0.78	0.96	0.98	0.97	0.95
2	0.20	0.28	0.51	0.64	0.68	0.83	0.87	0.91	0.95	0.94
3	0.19	0.32	0.39	0.55	0.69	0.72	0.83	0.86	0.88	0.94
4	0.43	0.48	0.54	0.69	0.83	0.83	0.92	0.96	0.98	0.98
5	0.30	0.44	0.58	0.65	0.84	0.83	0.91	0.94	0.95	0.98
6	0.28	0.39	0.52	0.74	0.70	0.87	0.89	0.91	0.96	0.98
7	0.07	0.09	0.09	0.17	0.29	0.29	0.40	0.42	0.54	0.66
8	0.07	0.09	0.10	0.17	0.28	0.34	0.41	0.50	0.57	0.59
9	0.07	0.08	0.13	0.14	0.29	0.40	0.39	0.47	0.56	0.58
10	0.32	0.47	0.64	0.69	0.82	0.81	0.89	0.95	0.95	0.93
11	0.38	0.52	0.61	0.73	0.79	0.83	0.85	0.94	0.94	0.95
12	0.33	0.62	0.58	0.67	0.78	0.89	0.90	0.95	0.91	0.99
13	0.19	0.38	0.46	0.64	0.75	0.87	0.89	0.97	0.98	0.99
14	0.18	0.32	0.51	0.63	0.76	0.86	0.90	0.97	0.99	0.98
15	0.16	0.26	0.51	0.65	0.73	0.87	0.90	0.97	0.92	1.00
16	0.24	0.31	0.47	0.70	0.86	0.87	0.85	0.97	0.99	0.98
17	0.20	0.31	0.49	0.62	0.82	0.87	0.93	0.96	0.99	1.00
18	0.19	0.26	0.41	0.50	0.72	0.73	0.91	0.91	0.95	0.98
19	0.20	0.42	0.54	0.67	0.75	0.88	0.90	0.95	0.96	0.98
20	0.24	0.34	0.50	0.69	0.82	0.85	0.97	0.96	0.98	0.97
21	0.21	0.27	0.44	0.65	0.84	0.90	0.94	0.95	0.96	0.97
22	0.26	0.57	0.53	0.78	0.87	0.86	0.93	0.95	1.00	1.00
23	0.31	0.48	0.67	0.76	0.89	0.95	0.93	0.98	0.99	1.00
24	0.31	0.47	0.62	0.77	0.83	0.90	0.93	0.97	0.99	1.00
25	0.22	0.46	0.54	0.71	0.83	0.88	0.93	1.00	0.99	1.00
26	0.21	0.34	0.50	0.71	0.79	0.90	0.93	0.97	1.00	0.98
27	0.16	0.21	0.31	0.45	0.60	0.74	0.77	0.88	0.90	0.94
28	0.26	0.48	0.62	0.69	0.82	0.92	0.94	0.96	1.00	1.00
29	0.27	0.42	0.53	0.71	0.79	0.91	0.97	0.98	1.00	0.99
30	0.15	0.32	0.45	0.52	0.63	0.59	0.82	0.85	0.86	0.97
31	0.20	0.38	0.52	0.63	0.78	0.93	0.97	0.99	1.00	1.00
32	0.23	0.39	0.60	0.70	0.81	0.89	0.95	0.98	1.00	1.00
33	0.10	0.32	0.51	0.65	0.86	0.90	0.96	0.98	1.00	1.00
34	0.19	0.29	0.45	0.56	0.81	0.86	0.96	0.97	0.99	1.00
35	0.22	0.42	0.45	0.66	0.78	0.94	0.97	0.99	0.99	1.00
36	0.13	0.33	0.46	0.64	0.82	0.86	0.89	0.95	0.99	1.00
37	0.17	0.32	0.62	0.67	0.85	0.95	0.99	1.00	0.97	1.00
38	0.19	0.38	0.45	0.71	0.90	0.98	0.99	1.00	1.00	1.00
39	0.22	0.22	0.58	0.65	0.80	0.95	0.95	0.99	1.00	1.00
40	0.26	0.45	0.65	0.79	0.90	0.96	0.99	1.00	1.00	1.00
41	0.25	0.35	0.62	0.75	0.83	0.94	0.95	1.00	1.00	1.00
42	0.21	0.43	0.59	0.78	0.86	0.95	0.99	0.99	1.00	1.00

Table 17: POD according to each scenario and each k2 value, k1 = 5, alpha = 0.05

	k2=1	k2=2	k2=3	k2=4	k2=5	k2=6	k2=7	k2=8	k2=9	k2=10
1	0.33	0.33	0.42	0.61	0.69	0.72	0.91	0.83	0.89	0.91
2	0.23	0.30	0.50	0.46	0.71	0.77	0.86	0.88	0.92	0.94
3	0.20	0.25	0.39	0.56	0.56	0.63	0.77	0.81	0.93	0.94
4	0.32	0.36	0.56	0.62	0.74	0.84	0.89	0.96	0.99	0.99
5	0.23	0.41	0.43	0.57	0.71	0.88	0.86	0.91	0.92	0.98
6	0.28	0.33	0.60	0.60	0.71	0.75	0.86	0.97	0.96	0.97
7	0.07	0.10	0.12	0.18	0.20	0.23	0.34	0.41	0.55	0.65
8	0.04	0.09	0.07	0.18	0.29	0.33	0.43	0.41	0.63	0.66
9	0.08	0.09	0.03	0.16	0.25	0.28	0.32	0.45	0.45	0.61
10	0.33	0.48	0.59	0.65	0.78	0.86	0.81	0.89	0.93	0.92
11	0.26	0.50	0.59	0.69	0.67	0.79	0.85	0.91	0.96	0.96
12	0.33	0.48	0.62	0.71	0.77	0.84	0.82	0.95	0.91	0.96
13	0.14	0.31	0.47	0.59	0.75	0.82	0.89	0.94	0.98	1.00
14	0.18	0.19	0.43	0.50	0.79	0.83	0.92	0.93	0.97	0.97
15	0.15	0.20	0.45	0.49	0.67	0.84	0.87	0.91	0.96	0.97
16	0.18	0.31	0.47	0.56	0.80	0.82	0.92	0.97	0.98	0.98
17	0.21	0.33	0.34	0.49	0.75	0.89	0.91	0.95	0.97	0.99
18	0.20	0.24	0.29	0.46	0.61	0.67	0.79	0.90	0.94	0.95
19	0.16	0.34	0.37	0.48	0.71	0.83	0.84	0.89	0.95	0.99
20	0.19	0.29	0.33	0.58	0.65	0.71	0.87	0.93	0.95	0.97
21	0.16	0.25	0.43	0.63	0.62	0.74	0.86	0.93	0.91	0.95
22	0.31	0.46	0.52	0.64	0.76	0.87	0.91	0.94	0.94	0.98
23	0.27	0.31	0.69	0.74	0.83	0.84	0.92	0.96	0.97	0.96
24	0.23	0.33	0.53	0.69	0.72	0.84	0.94	0.93	0.97	0.95
25	0.20	0.30	0.46	0.66	0.78	0.89	0.92	0.97	0.97	1.00
26	0.22	0.31	0.44	0.60	0.72	0.86	0.93	0.96	0.99	0.99
27	0.11	0.13	0.18	0.44	0.41	0.54	0.69	0.82	0.80	0.90
28	0.26	0.43	0.57	0.63	0.85	0.81	0.94	0.99	0.99	0.98
29	0.24	0.33	0.62	0.77	0.81	0.89	0.91	0.96	0.96	0.98
30	0.24	0.30	0.27	0.54	0.51	0.60	0.68	0.85	0.87	0.94
31	0.19	0.29	0.51	0.65	0.82	0.90	0.93	0.99	1.00	1.00
32	0.17	0.34	0.48	0.65	0.68	0.92	0.95	0.99	1.00	1.00
33	0.16	0.26	0.32	0.63	0.75	0.84	0.96	0.97	1.00	1.00
34	0.13	0.27	0.35	0.56	0.77	0.88	0.96	0.97	1.00	1.00
35	0.26	0.32	0.45	0.61	0.79	0.86	0.89	0.98	0.98	1.00
36	0.27	0.27	0.46	0.49	0.74	0.79	0.93	0.99	0.98	0.99
37	0.22	0.31	0.45	0.69	0.83	0.88	0.95	0.98	1.00	1.00
38	0.22	0.28	0.59	0.71	0.83	0.92	0.98	0.99	1.00	1.00
39	0.15	0.34	0.45	0.60	0.85	0.91	0.99	0.99	1.00	1.00
40	0.22	0.37	0.48	0.77	0.83	0.91	0.98	0.99	1.00	1.00
41	0.24	0.38	0.49	0.68	0.93	0.93	0.98	1.00	1.00	1.00
42	0.23	0.42	0.55	0.76	0.88	0.95	0.99	0.99	1.00	0.99

Table 18: POD according to each scenario and each k2 value, k1 = 10, alpha = 0.05
