# Supplementary material for: Evaluation and comparison of statistical methods for early temporal detection of outbreaks: A simulation-based study
Source: PLoS One. 2017 Jul 17;12(7):e0181227. doi: 10.1371/journal.pone.0181227 (PMC5513450; doi:10.1371/journal.pone.0181227)
Supplement: S4 Appendix — (PDF) [file pone.0181227.s004.pdf]

Evaluation and Comparison of Statistical Methods for Early Temporal Detection of Outbreaks: a Simulation-Based Study

Appendix S4: Overall performances of Periodic Poisson GLM algorithm ($\alpha = 0.001, 0.01$ and 0.05)

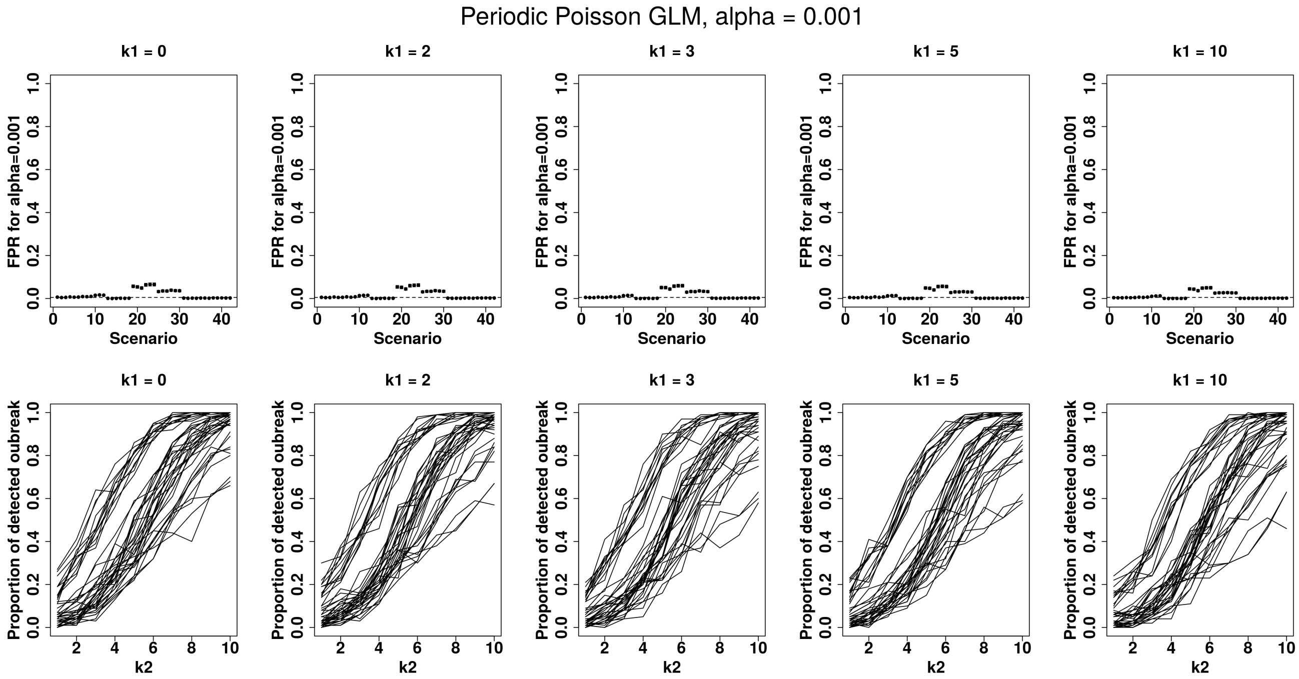

Figure 1: Periodic Poisson GLM algorithm performances for $\alpha = 0.001$ by increasing past outbreak amplitude k_1 = 0, 2, 3, 5 or 10 with (i) on the first row: false positive rate for 42 simulated scenarios, (ii) on the second row: probability of detection for 42 simulated scenarios (each curve corresponding to a scenario) by increasing current outbreak amplitude $k_2 = 1$ to 10.

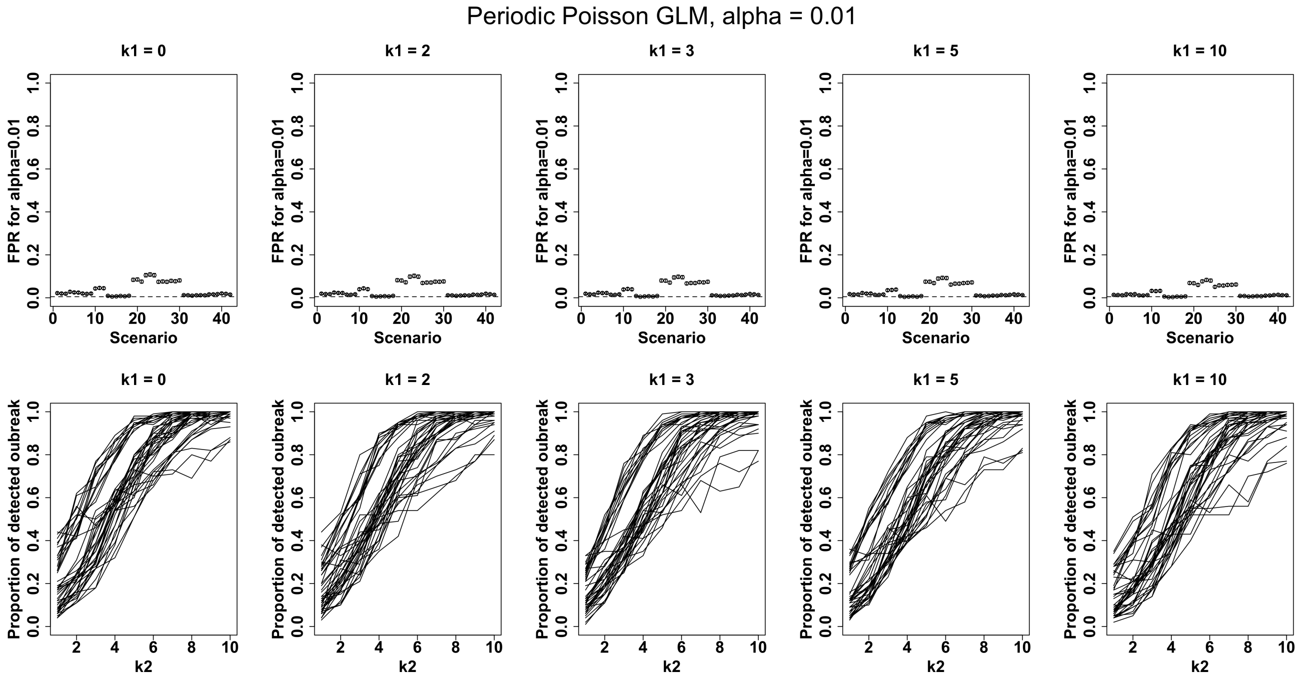

Figure 2: Periodic Poisson GLM algorithm performances for $\alpha = 0.01$ by increasing past outbreak amplitude k_1 = 0, 2, 3, 5 or 10 with (i) on the first row: false positive rate for 42 simulated scenarios, (ii) on the second row: probability of detection for 42 simulated scenarios (each curve corresponding to a scenario) by increasing current outbreak amplitude $k_2 = 1$ to 10.

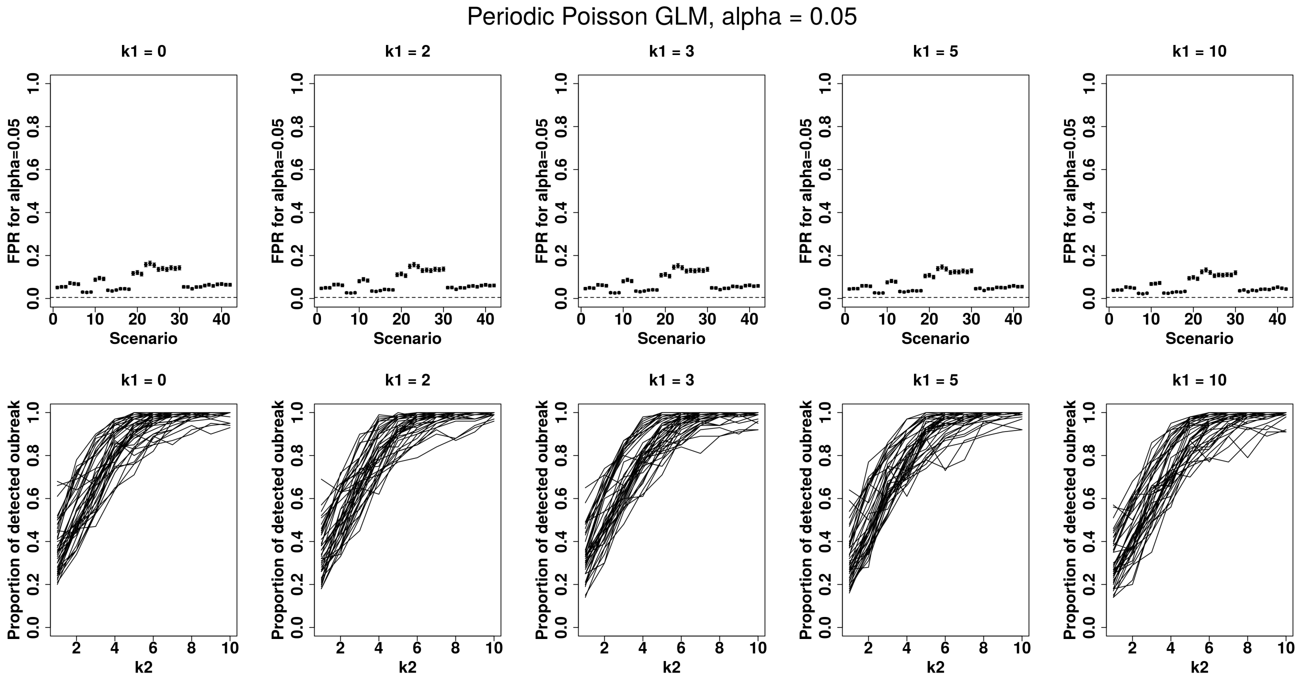

Figure 3: Periodic Poisson GLM algorithm performances for $\alpha = 0.05$ by increasing past outbreak amplitude $k_1 = 0$, 2, 3, 5 or 10 with (i) on the first row: false positive rate for 42 simulated scenarios, (ii) on the second row: probability of detection for 42 simulated scenarios (each curve corresponding to a scenario) by increasing current outbreak amplitude $k_2 = 1$ to 10.

Overall performances of Periodic Poisson GLM algorithm ($\alpha = 0.001$)

	FPR k1=0	FPR k1=2	FPR k1=3	FPR k1=5	FPR k1=10
1	0.01	0.01	0.01	0.00	0.00
2	0.00	0.00	0.00	0.00	0.00
3	0.01	0.00	0.00	0.00	0.00
4	0.01	0.01	0.01	0.01	0.00
5	0.01	0.00	0.00	0.00	0.00
6	0.01	0.01	0.01	0.00	0.00
7	0.01	0.01	0.01	0.01	0.01
8	0.01	0.01	0.01	0.01	0.01
9	0.01	0.01	0.01	0.01	0.01
10	0.01	0.01	0.01	0.01	0.01
11	0.02	0.01	0.01	0.01	0.01
12	0.02	0.01	0.01	0.01	0.01
13	0.00	0.00	0.00	0.00	0.00
14	0.00	0.00	0.00	0.00	0.00
15	0.00	0.00	0.00	0.00	0.00
16	0.00	0.00	0.00	0.00	0.00
17	0.00	0.00	0.00	0.00	0.00
18	0.00	0.00	0.00	0.00	0.00
19	0.06	0.05	0.05	0.05	0.04
20	0.05	0.05	0.05	0.05	0.04
21	0.05	0.04	0.04	0.04	0.04
22	0.06	0.06	0.06	0.06	0.05
23	0.07	0.06	0.06	0.06	0.05
24	0.07	0.06	0.06	0.06	0.05
25	0.03	0.03	0.03	0.03	0.03
26	0.04	0.03	0.03	0.03	0.03
27	0.03	0.03	0.03	0.03	0.03
28	0.04	0.04	0.04	0.03	0.03
29	0.04	0.03	0.03	0.03	0.03
30	0.04	0.03	0.03	0.03	0.03
31	0.00	0.00	0.00	0.00	0.00
32	0.00	0.00	0.00	0.00	0.00
33	0.00	0.00	0.00	0.00	0.00
34	0.00	0.00	0.00	0.00	0.00
35	0.00	0.00	0.00	0.00	0.00
36	0.00	0.00	0.00	0.00	0.00
37	0.00	0.00	0.00	0.00	0.00
38	0.00	0.00	0.00	0.00	0.00
39	0.00	0.00	0.00	0.00	0.00
40	0.00	0.00	0.00	0.00	0.00
41	0.00	0.00	0.00	0.00	0.00
42	0.00	0.00	0.00	0.00	0.00

Table 1: FPR according to each scenario and each k1 value, alpha = 0.001

	k2=1	k2=2	k2=3	k2=4	k2=5	k2=6	k2=7	k2=8	k2=9	k2=10
1	0.06	0.12	0.09	0.18	0.29	0.40	0.54	0.71	0.84	0.81
2	0.06	0.08	0.10	0.25	0.38	0.45	0.55	0.68	0.78	0.91
3	0.07	0.07	0.11	0.23	0.30	0.38	0.51	0.69	0.81	0.83
4	0.06	0.08	0.09	0.26	0.40	0.53	0.71	0.80	0.85	0.97
5	0.05	0.06	0.15	0.39	0.34	0.50	0.61	0.83	0.89	0.95
6	0.05	0.10	0.16	0.32	0.41	0.58	0.72	0.80	0.98	0.94
7	0.07	0.17	0.23	0.29	0.33	0.37	0.43	0.53	0.61	0.68
8	0.12	0.13	0.19	0.28	0.30	0.45	0.44	0.40	0.62	0.66
9	0.09	0.14	0.18	0.26	0.28	0.41	0.54	0.60	0.61	0.70
10	0.11	0.12	0.20	0.39	0.59	0.64	0.82	0.83	0.91	0.95
11	0.06	0.12	0.30	0.43	0.58	0.71	0.81	0.85	0.92	0.97
12	0.04	0.13	0.29	0.32	0.48	0.71	0.77	0.84	0.94	0.94
13	0.00	0.04	0.03	0.13	0.24	0.32	0.53	0.58	0.80	0.84
14	0.04	0.04	0.06	0.14	0.23	0.32	0.50	0.63	0.75	0.80
15	0.04	0.04	0.06	0.15	0.22	0.41	0.53	0.69	0.79	0.89
16	0.02	0.05	0.08	0.16	0.24	0.52	0.67	0.84	0.92	0.97
17	0.01	0.02	0.04	0.20	0.36	0.49	0.64	0.81	0.89	0.97
18	0.01	0.06	0.07	0.16	0.30	0.49	0.69	0.76	0.89	0.95
19	0.19	0.25	0.44	0.61	0.82	0.91	0.95	0.97	1.00	0.99
20	0.19	0.29	0.44	0.59	0.71	0.88	0.96	0.96	1.00	1.00
21	0.15	0.32	0.41	0.56	0.76	0.88	0.96	0.98	1.00	1.00
22	0.24	0.34	0.59	0.70	0.81	0.95	0.99	1.00	1.00	1.00
23	0.27	0.41	0.64	0.63	0.86	0.91	0.99	1.00	1.00	1.00
24	0.26	0.38	0.58	0.76	0.84	0.89	1.00	1.00	0.99	1.00
25	0.15	0.27	0.46	0.64	0.75	0.92	0.98	0.99	1.00	1.00
26	0.15	0.24	0.52	0.63	0.79	0.91	0.92	0.99	1.00	1.00
27	0.14	0.33	0.37	0.61	0.82	0.87	0.98	0.98	1.00	1.00
28	0.11	0.36	0.46	0.69	0.81	0.94	0.98	1.00	0.98	1.00
29	0.19	0.34	0.45	0.67	0.81	0.91	0.96	0.99	1.00	1.00
30	0.13	0.33	0.39	0.67	0.73	0.95	0.95	0.99	1.00	1.00
31	0.03	0.04	0.10	0.18	0.38	0.52	0.71	0.93	0.90	0.95
32	0.01	0.01	0.16	0.26	0.30	0.59	0.66	0.86	0.95	1.00
33	0.00	0.04	0.11	0.20	0.31	0.53	0.78	0.87	0.92	0.98
34	0.00	0.03	0.14	0.25	0.52	0.62	0.73	0.80	0.93	0.99
35	0.01	0.05	0.08	0.27	0.34	0.59	0.72	0.89	0.93	0.99
36	0.03	0.01	0.13	0.22	0.50	0.52	0.79	0.90	0.94	0.96
37	0.00	0.08	0.11	0.32	0.39	0.60	0.86	0.92	0.98	0.98
38	0.03	0.02	0.16	0.23	0.46	0.69	0.86	0.94	0.96	0.99
39	0.03	0.06	0.20	0.20	0.52	0.63	0.76	0.90	0.97	1.00
40	0.05	0.07	0.21	0.30	0.42	0.63	0.84	0.95	0.98	0.99
41	0.05	0.04	0.16	0.26	0.51	0.67	0.86	0.93	0.97	0.99
42	0.03	0.04	0.14	0.23	0.46	0.69	0.84	0.86	0.98	0.98

Table 2: POD according to each scenario and each k2 value, k1 = 0, alpha = 0.001

	k2=1	k2=2	k2=3	k2=4	k2=5	k2=6	k2=7	k2=8	k2=9	k2=10
1	0.03	0.07	0.13	0.17	0.31	0.40	0.51	0.68	0.77	0.77
2	0.03	0.12	0.10	0.17	0.36	0.34	0.49	0.69	0.68	0.82
3	0.03	0.07	0.12	0.20	0.29	0.41	0.59	0.65	0.69	0.86
4	0.02	0.12	0.15	0.22	0.44	0.53	0.68	0.83	0.91	0.94
5	0.06	0.11	0.12	0.27	0.42	0.56	0.72	0.81	0.92	0.93
6	0.02	0.10	0.14	0.29	0.37	0.59	0.69	0.87	0.93	0.90
7	0.10	0.16	0.15	0.25	0.36	0.30	0.40	0.52	0.59	0.57
8	0.09	0.13	0.14	0.18	0.31	0.35	0.38	0.46	0.56	0.67
9	0.08	0.08	0.14	0.20	0.30	0.33	0.43	0.45	0.56	0.67
10	0.08	0.13	0.19	0.34	0.49	0.67	0.75	0.82	0.95	0.97
11	0.05	0.08	0.26	0.29	0.57	0.60	0.75	0.90	0.92	0.95
12	0.06	0.18	0.16	0.29	0.54	0.63	0.82	0.81	0.89	0.97
13	0.01	0.04	0.03	0.11	0.26	0.31	0.54	0.66	0.80	0.84
14	0.01	0.01	0.06	0.12	0.22	0.31	0.52	0.65	0.82	0.88
15	0.01	0.03	0.06	0.17	0.36	0.38	0.50	0.63	0.69	0.84
16	0.01	0.01	0.08	0.11	0.26	0.51	0.68	0.72	0.96	0.94
17	0.00	0.02	0.06	0.18	0.27	0.45	0.70	0.83	0.93	0.92
18	0.01	0.09	0.04	0.13	0.32	0.40	0.55	0.79	0.86	0.98
19	0.20	0.30	0.43	0.66	0.82	0.87	0.94	0.99	0.99	1.00
20	0.16	0.27	0.45	0.56	0.69	0.91	0.94	0.96	1.00	1.00
21	0.14	0.21	0.39	0.55	0.76	0.86	0.97	0.97	0.99	1.00
22	0.22	0.32	0.57	0.75	0.83	0.98	0.99	1.00	1.00	1.00
23	0.20	0.38	0.51	0.71	0.88	0.97	0.99	1.00	1.00	1.00
24	0.30	0.33	0.63	0.71	0.87	0.91	0.95	1.00	1.00	1.00
25	0.10	0.25	0.40	0.69	0.85	0.88	0.94	0.98	1.00	1.00
26	0.14	0.37	0.43	0.46	0.76	0.93	0.99	0.98	1.00	1.00
27	0.19	0.22	0.38	0.67	0.78	0.92	0.94	0.99	1.00	1.00
28	0.15	0.33	0.42	0.59	0.84	0.91	0.99	1.00	1.00	1.00
29	0.19	0.23	0.45	0.52	0.78	0.87	0.99	0.99	1.00	1.00
30	0.15	0.25	0.44	0.67	0.82	0.88	0.95	1.00	1.00	1.00
31	0.03	0.04	0.08	0.26	0.30	0.55	0.78	0.85	0.93	0.99
32	0.00	0.08	0.16	0.21	0.32	0.53	0.76	0.84	0.89	0.98
33	0.01	0.03	0.11	0.17	0.43	0.47	0.72	0.90	0.94	0.97
34	0.02	0.05	0.10	0.20	0.37	0.57	0.79	0.89	0.96	0.98
35	0.00	0.07	0.09	0.15	0.34	0.56	0.77	0.85	0.99	0.96
36	0.03	0.06	0.10	0.24	0.23	0.63	0.79	0.94	0.94	0.98
37	0.03	0.05	0.14	0.26	0.56	0.57	0.73	0.89	0.97	0.98
38	0.00	0.03	0.13	0.25	0.38	0.70	0.81	0.91	0.98	0.98
39	0.03	0.06	0.15	0.21	0.45	0.66	0.78	0.93	0.95	0.99
40	0.01	0.06	0.11	0.29	0.50	0.71	0.86	0.92	0.98	1.00
41	0.02	0.03	0.17	0.32	0.39	0.72	0.85	0.91	0.99	1.00
42	0.05	0.04	0.13	0.26	0.48	0.62	0.88	0.91	0.98	0.99

Table 3: POD according to each scenario and each k2 value, k1 = 2, alpha = 0.001

	k2=1	k2=2	k2=3	k2=4	k2=5	k2=6	k2=7	k2=8	k2=9	k2=10
1	0.03	0.07	0.08	0.24	0.33	0.38	0.58	0.67	0.77	0.82
2	0.03	0.09	0.12	0.23	0.31	0.42	0.59	0.58	0.71	0.75
3	0.02	0.09	0.12	0.23	0.31	0.45	0.64	0.77	0.71	0.84
4	0.05	0.10	0.14	0.22	0.38	0.58	0.66	0.79	0.93	0.94
5	0.02	0.10	0.16	0.33	0.41	0.50	0.68	0.91	0.86	0.96
6	0.07	0.11	0.21	0.27	0.39	0.53	0.73	0.74	0.87	0.93
7	0.08	0.11	0.15	0.18	0.25	0.35	0.37	0.48	0.52	0.60
8	0.09	0.11	0.11	0.21	0.22	0.34	0.44	0.37	0.43	0.58
9	0.06	0.15	0.13	0.21	0.23	0.39	0.35	0.54	0.52	0.63
10	0.05	0.12	0.20	0.36	0.45	0.72	0.78	0.82	0.90	0.97
11	0.07	0.11	0.23	0.38	0.61	0.58	0.75	0.89	0.90	0.97
12	0.08	0.18	0.23	0.32	0.49	0.66	0.67	0.85	0.91	0.94
13	0.00	0.03	0.08	0.09	0.30	0.34	0.57	0.57	0.80	0.89
14	0.01	0.00	0.04	0.05	0.20	0.26	0.55	0.65	0.76	0.78
15	0.00	0.03	0.06	0.12	0.16	0.33	0.42	0.66	0.73	0.81
16	0.00	0.03	0.09	0.22	0.22	0.34	0.61	0.77	0.91	0.87
17	0.01	0.00	0.09	0.12	0.25	0.46	0.60	0.77	0.88	0.91
18	0.00	0.03	0.04	0.23	0.22	0.45	0.66	0.74	0.91	0.91
19	0.19	0.32	0.42	0.59	0.76	0.86	0.95	0.97	0.99	1.00
20	0.16	0.33	0.39	0.47	0.74	0.82	0.96	1.00	0.99	1.00
21	0.14	0.32	0.43	0.56	0.74	0.89	0.85	0.99	1.00	1.00
22	0.14	0.41	0.49	0.68	0.86	0.93	0.99	0.99	1.00	1.00
23	0.21	0.29	0.57	0.64	0.90	0.91	0.97	1.00	1.00	1.00
24	0.15	0.27	0.47	0.76	0.87	0.97	0.97	1.00	1.00	1.00
25	0.13	0.25	0.39	0.61	0.81	0.90	0.98	0.99	0.99	1.00
26	0.15	0.29	0.42	0.54	0.77	0.92	0.94	0.98	0.99	1.00
27	0.12	0.17	0.40	0.61	0.75	0.91	0.97	1.00	0.99	1.00
28	0.16	0.31	0.44	0.60	0.75	0.94	0.97	0.99	1.00	1.00
29	0.17	0.22	0.53	0.65	0.84	0.89	0.96	1.00	1.00	1.00
30	0.12	0.29	0.40	0.57	0.83	0.86	0.98	0.99	0.99	1.00
31	0.00	0.07	0.08	0.14	0.33	0.68	0.71	0.82	0.96	0.98
32	0.02	0.02	0.05	0.16	0.42	0.58	0.73	0.84	0.93	1.00
33	0.02	0.01	0.09	0.25	0.32	0.62	0.74	0.85	0.95	0.97
34	0.00	0.04	0.08	0.17	0.33	0.64	0.71	0.84	0.96	1.00
35	0.00	0.04	0.10	0.21	0.42	0.56	0.75	0.87	0.94	1.00
36	0.01	0.06	0.16	0.20	0.40	0.59	0.75	0.89	0.93	0.96
37	0.03	0.05	0.12	0.20	0.46	0.66	0.80	0.89	0.96	1.00
38	0.01	0.02	0.14	0.21	0.47	0.76	0.81	0.88	0.96	1.00
39	0.02	0.08	0.16	0.23	0.38	0.62	0.79	0.93	1.00	0.99
40	0.04	0.05	0.13	0.24	0.38	0.67	0.75	0.94	0.97	1.00
41	0.04	0.04	0.14	0.31	0.38	0.68	0.82	0.89	0.93	1.00
42	0.02	0.06	0.16	0.27	0.50	0.66	0.83	0.93	0.97	0.98

Table 4: POD according to each scenario and each k2 value, k1 = 3, alpha = 0.001

	k2=1	k2=2	k2=3	k2=4	k2=5	k2=6	k2=7	k2=8	k2=9	k2=10
1	0.02	0.06	0.08	0.21	0.29	0.32	0.61	0.72	0.74	0.83
2	0.02	0.03	0.10	0.21	0.28	0.43	0.51	0.64	0.75	0.77
3	0.06	0.11	0.15	0.21	0.29	0.39	0.59	0.66	0.71	0.78
4	0.04	0.08	0.10	0.24	0.39	0.52	0.65	0.79	0.88	0.95
5	0.04	0.06	0.21	0.20	0.42	0.55	0.52	0.84	0.89	0.93
6	0.02	0.06	0.14	0.26	0.29	0.55	0.72	0.76	0.86	0.95
7	0.05	0.07	0.13	0.19	0.30	0.27	0.45	0.46	0.51	0.62
8	0.05	0.09	0.11	0.15	0.22	0.37	0.39	0.44	0.56	0.59
9	0.09	0.12	0.17	0.15	0.23	0.40	0.40	0.56	0.51	0.58
10	0.06	0.11	0.24	0.32	0.45	0.56	0.77	0.85	0.91	0.92
11	0.08	0.13	0.21	0.39	0.54	0.56	0.78	0.93	0.94	0.95
12	0.07	0.18	0.21	0.34	0.43	0.67	0.73	0.93	0.87	0.97
13	0.01	0.03	0.09	0.10	0.15	0.33	0.45	0.70	0.78	0.82
14	0.01	0.01	0.06	0.09	0.26	0.29	0.45	0.67	0.76	0.85
15	0.02	0.04	0.08	0.16	0.19	0.27	0.47	0.54	0.75	0.87
16	0.00	0.04	0.07	0.14	0.26	0.49	0.55	0.75	0.91	0.95
17	0.00	0.03	0.09	0.17	0.31	0.44	0.60	0.76	0.91	0.95
18	0.00	0.02	0.09	0.12	0.31	0.42	0.66	0.75	0.86	0.89
19	0.14	0.41	0.38	0.63	0.73	0.91	0.93	0.99	0.99	0.99
20	0.17	0.19	0.41	0.65	0.78	0.85	0.98	0.98	1.00	1.00
21	0.16	0.24	0.38	0.54	0.81	0.90	0.95	0.97	1.00	1.00
22	0.22	0.33	0.42	0.65	0.85	0.93	0.99	1.00	1.00	1.00
23	0.21	0.32	0.50	0.70	0.82	0.89	0.99	0.99	1.00	1.00
24	0.23	0.33	0.45	0.70	0.85	0.96	0.95	1.00	1.00	1.00
25	0.11	0.27	0.41	0.62	0.72	0.82	0.93	1.00	0.99	1.00
26	0.13	0.23	0.44	0.62	0.82	0.89	0.94	0.99	1.00	1.00
27	0.16	0.34	0.38	0.57	0.82	0.87	0.97	0.99	0.98	1.00
28	0.12	0.37	0.38	0.58	0.71	0.86	0.97	0.98	0.99	1.00
29	0.23	0.21	0.38	0.56	0.74	0.91	0.99	1.00	1.00	1.00
30	0.17	0.33	0.45	0.68	0.78	0.87	0.97	0.97	0.99	1.00
31	0.04	0.01	0.11	0.15	0.31	0.48	0.75	0.83	0.94	0.95
32	0.00	0.07	0.08	0.22	0.39	0.58	0.75	0.81	0.94	0.96
33	0.00	0.06	0.11	0.19	0.33	0.49	0.69	0.85	0.93	0.94
34	0.00	0.02	0.05	0.18	0.32	0.52	0.78	0.84	0.96	0.96
35	0.02	0.00	0.09	0.12	0.38	0.60	0.78	0.83	0.95	0.94
36	0.02	0.04	0.14	0.21	0.36	0.49	0.70	0.85	0.93	1.00
37	0.03	0.03	0.10	0.27	0.41	0.68	0.84	0.92	0.92	0.99
38	0.01	0.07	0.09	0.17	0.51	0.67	0.85	0.91	0.99	1.00
39	0.04	0.04	0.12	0.19	0.39	0.56	0.78	0.90	0.97	1.00
40	0.03	0.09	0.17	0.27	0.36	0.64	0.84	0.89	0.94	1.00
41	0.02	0.04	0.17	0.26	0.38	0.61	0.81	0.91	0.97	0.99
42	0.00	0.02	0.11	0.27	0.40	0.59	0.82	0.91	0.97	1.00

Table 5: POD according to each scenario and each k2 value, k1 = 5, alpha = 0.001

	k2=1	k2=2	k2=3	k2=4	k2=5	k2=6	k2=7	k2=8	k2=9	k2=10
1	0.06	0.04	0.11	0.25	0.22	0.37	0.51	0.50	0.64	0.78
2	0.02	0.06	0.09	0.12	0.30	0.39	0.47	0.59	0.70	0.80
3	0.06	0.08	0.10	0.19	0.26	0.34	0.45	0.57	0.71	0.77
4	0.01	0.08	0.13	0.24	0.29	0.57	0.69	0.79	0.92	0.91
5	0.02	0.10	0.08	0.19	0.31	0.53	0.60	0.71	0.84	0.91
6	0.02	0.05	0.17	0.22	0.30	0.52	0.74	0.76	0.74	0.93
7	0.07	0.06	0.12	0.20	0.23	0.23	0.30	0.34	0.47	0.63
8	0.05	0.06	0.06	0.17	0.35	0.29	0.30	0.34	0.46	0.63
9	0.08	0.05	0.10	0.19	0.23	0.28	0.30	0.45	0.51	0.46
10	0.07	0.05	0.18	0.20	0.43	0.59	0.66	0.79	0.89	0.90
11	0.05	0.08	0.19	0.27	0.42	0.55	0.69	0.81	0.85	0.90
12	0.06	0.11	0.19	0.27	0.46	0.54	0.70	0.81	0.96	0.96
13	0.02	0.00	0.04	0.07	0.18	0.39	0.45	0.57	0.72	0.80
14	0.00	0.02	0.04	0.04	0.25	0.26	0.44	0.61	0.70	0.76
15	0.00	0.00	0.05	0.10	0.11	0.30	0.47	0.56	0.70	0.75
16	0.00	0.01	0.06	0.09	0.22	0.33	0.59	0.77	0.91	0.95
17	0.01	0.00	0.05	0.13	0.30	0.51	0.71	0.70	0.81	0.91
18	0.00	0.02	0.02	0.09	0.18	0.41	0.57	0.69	0.78	0.88
19	0.10	0.26	0.24	0.40	0.70	0.82	0.87	0.94	1.00	1.00
20	0.15	0.22	0.24	0.43	0.66	0.76	0.86	0.98	0.97	0.99
21	0.14	0.26	0.34	0.58	0.65	0.82	0.90	0.95	0.97	1.00
22	0.24	0.31	0.37	0.61	0.76	0.92	0.97	0.99	0.99	1.00
23	0.17	0.16	0.50	0.69	0.78	0.90	0.97	0.97	1.00	1.00
24	0.16	0.23	0.40	0.62	0.76	0.87	0.99	0.99	1.00	1.00
25	0.09	0.19	0.38	0.53	0.75	0.82	0.93	1.00	0.98	1.00
26	0.19	0.27	0.37	0.56	0.68	0.85	0.96	0.98	0.99	1.00
27	0.16	0.18	0.26	0.56	0.66	0.75	0.93	0.97	0.99	0.99
28	0.11	0.21	0.37	0.53	0.77	0.86	0.95	0.99	1.00	1.00
29	0.14	0.22	0.43	0.66	0.82	0.86	0.97	0.98	1.00	1.00
30	0.22	0.26	0.33	0.54	0.75	0.84	0.95	0.99	1.00	1.00
31	0.01	0.02	0.11	0.14	0.35	0.44	0.68	0.87	0.93	0.96
32	0.03	0.03	0.08	0.13	0.30	0.46	0.69	0.92	0.99	0.98
33	0.01	0.02	0.06	0.14	0.28	0.55	0.59	0.80	0.89	0.98
34	0.00	0.03	0.07	0.15	0.39	0.47	0.68	0.77	0.95	0.95
35	0.01	0.03	0.07	0.13	0.34	0.57	0.65	0.85	0.95	0.96
36	0.03	0.01	0.12	0.23	0.45	0.47	0.68	0.85	0.92	0.98
37	0.00	0.01	0.06	0.17	0.39	0.59	0.80	0.88	0.93	1.00
38	0.01	0.03	0.17	0.23	0.33	0.56	0.79	0.92	0.97	0.99
39	0.01	0.03	0.07	0.20	0.34	0.61	0.73	0.92	0.99	0.99
40	0.02	0.02	0.10	0.22	0.41	0.52	0.74	0.83	0.93	1.00
41	0.03	0.03	0.06	0.24	0.43	0.44	0.79	0.90	0.94	1.00
42	0.02	0.06	0.08	0.31	0.35	0.66	0.74	0.94	0.98	0.99

Table 6: POD according to each scenario and each k2 value, k1 = 10, alpha = 0.001

Overall performances of Periodic Poisson GLM algorithm ($\alpha = 0.01$)

	FPR k1=0	FPR k1=2	FPR k1=3	FPR k1=5	FPR k1=10
1	0.02	0.02	0.02	0.02	0.01
2	0.02	0.02	0.02	0.02	0.01
3	0.02	0.02	0.02	0.02	0.01
4	0.03	0.02	0.02	0.02	0.02
5	0.02	0.02	0.02	0.02	0.02
6	0.02	0.02	0.02	0.02	0.02
7	0.02	0.01	0.01	0.01	0.01
8	0.02	0.01	0.01	0.01	0.01
9	0.02	0.02	0.02	0.02	0.01
10	0.04	0.04	0.04	0.04	0.03
11	0.05	0.04	0.04	0.04	0.03
12	0.04	0.04	0.04	0.04	0.03
13	0.01	0.01	0.01	0.01	0.01
14	0.01	0.01	0.00	0.00	0.00
15	0.01	0.01	0.01	0.01	0.00
16	0.01	0.01	0.01	0.01	0.01
17	0.01	0.01	0.01	0.00	0.00
18	0.01	0.01	0.01	0.01	0.01
19	0.08	0.08	0.08	0.07	0.07
20	0.09	0.08	0.08	0.07	0.07
21	0.08	0.07	0.07	0.07	0.06
22	0.11	0.10	0.09	0.09	0.08
23	0.11	0.10	0.10	0.09	0.08
24	0.11	0.10	0.10	0.09	0.08
25	0.07	0.07	0.07	0.06	0.05
26	0.08	0.07	0.07	0.07	0.06
27	0.07	0.07	0.07	0.07	0.06
28	0.08	0.07	0.07	0.07	0.06
29	0.08	0.07	0.07	0.07	0.06
30	0.08	0.08	0.07	0.07	0.06
31	0.01	0.01	0.01	0.01	0.01
32	0.01	0.01	0.01	0.01	0.01
33	0.01	0.01	0.01	0.01	0.01
34	0.01	0.01	0.01	0.01	0.01
35	0.01	0.01	0.01	0.01	0.01
36	0.01	0.01	0.01	0.01	0.01
37	0.02	0.01	0.01	0.01	0.01
38	0.02	0.01	0.01	0.01	0.01
39	0.02	0.02	0.01	0.01	0.01
40	0.02	0.02	0.02	0.02	0.01
41	0.02	0.02	0.02	0.01	0.01
42	0.01	0.01	0.01	0.01	0.01

Table 7: FPR according to each scenario and each k1 value, alpha = 0.01

	k2=1	k2=2	k2=3	k2=4	k2=5	k2=6	k2=7	k2=8	k2=9	k2=10
1	0.15	0.23	0.28	0.39	0.62	0.66	0.81	0.91	0.96	0.95
2	0.17	0.22	0.29	0.60	0.66	0.68	0.80	0.89	0.93	0.99
3	0.18	0.23	0.31	0.54	0.56	0.65	0.78	0.87	0.92	0.93
4	0.21	0.26	0.35	0.59	0.72	0.81	0.92	0.99	0.94	0.99
5	0.19	0.24	0.45	0.60	0.68	0.82	0.87	0.98	0.99	1.00
6	0.16	0.24	0.35	0.55	0.70	0.86	0.90	0.94	1.00	1.00
7	0.43	0.52	0.50	0.57	0.73	0.70	0.71	0.80	0.77	0.87
8	0.44	0.42	0.46	0.64	0.61	0.72	0.73	0.69	0.82	0.86
9	0.38	0.56	0.47	0.57	0.63	0.73	0.81	0.83	0.82	0.88
10	0.18	0.29	0.39	0.61	0.78	0.80	0.89	0.96	0.98	0.99
11	0.17	0.31	0.54	0.60	0.79	0.87	0.95	0.97	0.96	1.00
12	0.18	0.21	0.51	0.58	0.75	0.85	0.96	0.97	0.97	1.00
13	0.05	0.11	0.18	0.44	0.55	0.72	0.80	0.90	0.98	0.97
14	0.09	0.13	0.27	0.32	0.51	0.68	0.78	0.87	0.93	0.98
15	0.11	0.16	0.18	0.35	0.51	0.70	0.81	0.89	0.96	0.98
16	0.07	0.20	0.27	0.42	0.66	0.85	0.89	0.99	0.99	1.00
17	0.04	0.12	0.31	0.47	0.69	0.79	0.90	0.99	0.98	1.00
18	0.06	0.11	0.22	0.38	0.67	0.77	0.93	0.94	0.99	1.00
19	0.25	0.44	0.64	0.77	0.95	0.95	0.99	1.00	1.00	1.00
20	0.33	0.47	0.60	0.79	0.94	0.96	1.00	0.99	1.00	1.00
21	0.26	0.45	0.65	0.77	0.92	0.96	0.99	1.00	1.00	1.00
22	0.33	0.51	0.77	0.86	0.97	0.97	1.00	1.00	1.00	1.00
23	0.39	0.50	0.73	0.83	0.98	0.98	1.00	1.00	1.00	1.00
24	0.41	0.55	0.76	0.89	0.96	0.98	1.00	1.00	1.00	1.00
25	0.32	0.44	0.72	0.81	0.91	0.98	1.00	1.00	1.00	1.00
26	0.31	0.42	0.74	0.81	0.94	0.98	0.97	1.00	1.00	1.00
27	0.26	0.51	0.58	0.78	0.95	0.94	1.00	1.00	1.00	1.00
28	0.28	0.62	0.70	0.87	0.94	0.99	1.00	1.00	1.00	1.00
29	0.37	0.41	0.71	0.82	0.94	0.96	0.99	1.00	1.00	1.00
30	0.26	0.61	0.65	0.86	0.93	0.98	0.98	1.00	1.00	1.00
31	0.08	0.17	0.27	0.47	0.74	0.90	0.96	0.99	0.98	1.00
32	0.06	0.17	0.37	0.54	0.63	0.88	0.88	0.98	0.98	1.00
33	0.07	0.14	0.27	0.52	0.69	0.83	0.93	0.95	0.99	1.00
34	0.04	0.17	0.38	0.54	0.80	0.90	0.90	0.98	0.98	1.00
35	0.08	0.15	0.30	0.54	0.60	0.88	0.91	0.99	1.00	1.00
36	0.08	0.20	0.30	0.54	0.80	0.86	0.97	1.00	1.00	1.00
37	0.08	0.25	0.39	0.64	0.75	0.81	0.95	0.99	0.99	1.00
38	0.12	0.15	0.45	0.58	0.79	0.87	0.99	1.00	1.00	1.00
39	0.10	0.21	0.48	0.56	0.75	0.92	0.97	0.99	0.99	1.00
40	0.12	0.24	0.43	0.63	0.70	0.93	0.95	1.00	0.99	1.00
41	0.13	0.24	0.35	0.57	0.80	0.88	0.96	1.00	1.00	1.00
42	0.06	0.22	0.32	0.54	0.76	0.91	0.99	0.99	1.00	1.00

Table 8: POD according to each scenario and each k2 value, k1 = 0, alpha = 0.01

	k2=1	k2=2	k2=3	k2=4	k2=5	k2=6	k2=7	k2=8	k2=9	k2=10
1	0.08	0.17	0.33	0.40	0.58	0.63	0.75	0.83	0.85	0.91
2	0.11	0.27	0.24	0.39	0.63	0.71	0.76	0.83	0.88	0.95
3	0.11	0.23	0.36	0.48	0.49	0.69	0.86	0.88	0.93	0.95
4	0.14	0.23	0.37	0.57	0.71	0.74	0.89	0.98	0.99	0.99
5	0.16	0.25	0.39	0.52	0.67	0.82	0.89	0.96	0.97	0.98
6	0.06	0.25	0.42	0.54	0.71	0.79	0.89	0.97	0.98	0.99
7	0.27	0.37	0.42	0.47	0.61	0.62	0.65	0.68	0.80	0.80
8	0.38	0.33	0.39	0.51	0.54	0.54	0.62	0.70	0.77	0.87
9	0.30	0.28	0.44	0.45	0.57	0.66	0.70	0.73	0.77	0.89
10	0.19	0.30	0.49	0.57	0.70	0.84	0.90	0.95	0.97	1.00
11	0.20	0.30	0.52	0.52	0.79	0.81	0.93	0.98	0.98	0.99
12	0.17	0.29	0.35	0.55	0.79	0.84	0.94	0.98	0.94	1.00
13	0.08	0.10	0.25	0.48	0.60	0.61	0.87	0.87	0.94	0.96
14	0.06	0.11	0.25	0.35	0.42	0.68	0.75	0.86	0.96	0.96
15	0.13	0.15	0.23	0.43	0.68	0.70	0.77	0.90	0.92	0.94
16	0.08	0.16	0.21	0.43	0.70	0.82	0.89	0.95	0.99	0.99
17	0.07	0.10	0.29	0.56	0.63	0.80	0.90	0.96	1.00	0.98
18	0.04	0.17	0.31	0.36	0.60	0.70	0.87	0.93	0.98	1.00
19	0.29	0.46	0.61	0.82	0.94	0.98	0.99	1.00	1.00	1.00
20	0.24	0.38	0.61	0.83	0.90	0.98	0.99	1.00	1.00	1.00
21	0.24	0.36	0.60	0.78	0.88	0.97	1.00	0.99	1.00	1.00
22	0.33	0.50	0.71	0.88	0.92	0.99	1.00	1.00	1.00	1.00
23	0.37	0.49	0.69	0.89	0.95	1.00	1.00	1.00	1.00	1.00
24	0.44	0.53	0.80	0.85	0.94	0.98	0.97	1.00	1.00	1.00
25	0.28	0.41	0.55	0.89	0.94	0.94	0.98	1.00	1.00	1.00
26	0.26	0.49	0.71	0.75	0.95	0.98	1.00	1.00	1.00	1.00
27	0.26	0.38	0.60	0.82	0.93	0.99	0.98	1.00	1.00	1.00
28	0.28	0.50	0.61	0.86	0.95	0.97	1.00	1.00	1.00	1.00
29	0.31	0.52	0.59	0.77	0.94	0.96	1.00	1.00	1.00	1.00
30	0.20	0.45	0.62	0.90	0.93	0.98	0.99	1.00	1.00	1.00
31	0.10	0.19	0.26	0.53	0.71	0.87	0.94	0.99	0.98	1.00
32	0.07	0.22	0.38	0.48	0.66	0.83	0.95	0.96	0.98	0.99
33	0.03	0.12	0.33	0.50	0.77	0.78	0.93	0.98	1.00	1.00
34	0.09	0.18	0.34	0.54	0.69	0.84	0.94	0.98	1.00	1.00
35	0.08	0.18	0.31	0.40	0.67	0.81	0.97	1.00	1.00	1.00
36	0.12	0.20	0.35	0.51	0.61	0.90	0.96	0.97	1.00	0.99
37	0.13	0.21	0.38	0.54	0.80	0.85	0.90	0.98	1.00	1.00
38	0.04	0.21	0.37	0.60	0.73	0.91	0.97	1.00	1.00	1.00
39	0.06	0.31	0.29	0.59	0.77	0.92	0.97	0.97	1.00	1.00
40	0.09	0.36	0.40	0.61	0.83	0.90	0.98	0.98	0.98	1.00
41	0.14	0.16	0.46	0.62	0.70	0.96	0.96	0.98	1.00	1.00
42	0.12	0.22	0.40	0.57	0.74	0.87	0.97	0.99	1.00	1.00

Table 9: POD according to each scenario and each k2 value, k1 = 2, alpha = 0.01

	k2=1	k2=2	k2=3	k2=4	k2=5	k2=6	k2=7	k2=8	k2=9	k2=10
1	0.12	0.19	0.28	0.45	0.66	0.61	0.77	0.92	0.89	0.90
2	0.11	0.26	0.31	0.41	0.59	0.67	0.86	0.83	0.88	0.92
3	0.13	0.19	0.37	0.46	0.60	0.73	0.83	0.90	0.95	0.94
4	0.13	0.25	0.36	0.49	0.62	0.82	0.92	0.92	0.97	0.98
5	0.14	0.24	0.37	0.56	0.65	0.76	0.87	0.98	0.97	1.00
6	0.17	0.31	0.37	0.54	0.62	0.84	0.91	0.93	0.96	0.98
7	0.27	0.28	0.42	0.41	0.47	0.59	0.70	0.76	0.72	0.77
8	0.33	0.35	0.35	0.47	0.52	0.54	0.68	0.63	0.65	0.82
9	0.33	0.40	0.45	0.47	0.54	0.66	0.53	0.79	0.82	0.82
10	0.19	0.22	0.41	0.59	0.74	0.91	0.94	0.93	0.97	1.00
11	0.13	0.26	0.37	0.62	0.73	0.83	0.89	0.97	1.00	0.99
12	0.21	0.28	0.44	0.59	0.76	0.84	0.88	0.98	0.97	0.98
13	0.04	0.12	0.26	0.39	0.54	0.64	0.80	0.89	0.93	1.00
14	0.09	0.11	0.27	0.33	0.54	0.66	0.77	0.85	0.92	0.94
15	0.05	0.15	0.32	0.35	0.46	0.67	0.72	0.89	0.93	0.94
16	0.02	0.11	0.33	0.54	0.53	0.70	0.83	0.91	0.98	0.99
17	0.06	0.16	0.35	0.44	0.62	0.71	0.82	0.92	0.98	1.00
18	0.01	0.12	0.26	0.52	0.56	0.80	0.89	0.95	0.99	0.99
19	0.23	0.45	0.65	0.79	0.86	0.95	0.99	0.99	1.00	1.00
20	0.24	0.47	0.60	0.78	0.94	0.94	1.00	1.00	1.00	1.00
21	0.22	0.41	0.59	0.77	0.91	0.97	0.98	1.00	1.00	1.00
22	0.27	0.50	0.71	0.87	0.96	1.00	1.00	1.00	1.00	1.00
23	0.26	0.46	0.76	0.82	0.94	0.98	0.98	1.00	1.00	1.00
24	0.25	0.46	0.74	0.88	0.99	1.00	1.00	1.00	1.00	1.00
25	0.21	0.44	0.66	0.89	0.93	0.97	0.99	1.00	1.00	1.00
26	0.26	0.40	0.64	0.79	0.92	0.98	0.99	1.00	1.00	1.00
27	0.29	0.36	0.59	0.80	0.92	0.97	0.99	1.00	1.00	1.00
28	0.30	0.48	0.70	0.81	0.95	0.99	0.99	1.00	1.00	1.00
29	0.29	0.46	0.68	0.80	0.94	0.97	0.99	1.00	1.00	1.00
30	0.26	0.52	0.65	0.83	0.89	0.97	1.00	0.99	1.00	1.00
31	0.09	0.19	0.25	0.46	0.62	0.93	0.93	1.00	0.99	1.00
32	0.08	0.18	0.30	0.48	0.69	0.81	0.93	0.99	0.99	1.00
33	0.11	0.18	0.32	0.49	0.62	0.91	0.95	0.97	1.00	0.99
34	0.08	0.20	0.31	0.51	0.73	0.91	0.91	0.98	1.00	1.00
35	0.06	0.22	0.31	0.47	0.73	0.77	0.95	0.96	1.00	1.00
36	0.08	0.15	0.32	0.46	0.69	0.90	0.96	0.99	0.98	1.00
37	0.08	0.19	0.43	0.57	0.77	0.92	0.98	0.98	0.98	1.00
38	0.10	0.15	0.26	0.63	0.73	0.91	0.95	0.96	1.00	1.00
39	0.12	0.22	0.37	0.60	0.75	0.86	0.93	0.98	1.00	1.00
40	0.12	0.24	0.40	0.60	0.73	0.89	0.96	0.99	1.00	1.00
41	0.07	0.18	0.36	0.58	0.67	0.87	0.96	0.99	1.00	1.00
42	0.10	0.29	0.39	0.57	0.81	0.93	0.97	1.00	1.00	0.99

Table 10: POD according to each scenario and each k2 value, k1 = 3, alpha = 0.01

	k2=1	k2=2	k2=3	k2=4	k2=5	k2=6	k2=7	k2=8	k2=9	k2=10
1	0.12	0.19	0.30	0.39	0.48	0.61	0.82	0.94	0.92	0.94
2	0.12	0.18	0.31	0.39	0.52	0.71	0.78	0.87	0.88	0.97
3	0.13	0.22	0.29	0.49	0.58	0.59	0.80	0.82	0.86	0.92
4	0.16	0.21	0.31	0.45	0.66	0.75	0.86	0.96	0.99	1.00
5	0.12	0.22	0.45	0.47	0.70	0.80	0.89	0.95	0.97	0.98
6	0.07	0.18	0.33	0.60	0.62	0.86	0.88	0.94	0.98	1.00
7	0.26	0.34	0.34	0.49	0.61	0.49	0.63	0.73	0.73	0.83
8	0.29	0.33	0.39	0.42	0.46	0.54	0.58	0.79	0.76	0.82
9	0.36	0.33	0.46	0.39	0.47	0.69	0.66	0.75	0.78	0.81
10	0.12	0.25	0.47	0.54	0.75	0.85	0.92	0.93	0.98	1.00
11	0.12	0.29	0.43	0.65	0.79	0.79	0.92	0.97	0.99	0.99
12	0.15	0.34	0.41	0.60	0.74	0.84	0.93	0.97	0.96	0.99
13	0.05	0.15	0.25	0.42	0.52	0.69	0.81	0.87	0.94	0.94
14	0.03	0.15	0.27	0.39	0.57	0.64	0.78	0.90	0.95	0.96
15	0.09	0.10	0.27	0.44	0.55	0.69	0.79	0.85	0.93	0.99
16	0.07	0.13	0.23	0.46	0.67	0.81	0.83	0.92	0.98	0.98
17	0.04	0.11	0.31	0.44	0.70	0.84	0.83	0.93	1.00	1.00
18	0.04	0.12	0.26	0.40	0.51	0.78	0.88	0.90	0.99	0.99
19	0.25	0.48	0.57	0.80	0.91	0.97	0.98	1.00	1.00	1.00
20	0.30	0.31	0.63	0.80	0.90	0.96	0.99	0.99	1.00	1.00
21	0.24	0.35	0.58	0.76	0.95	0.95	0.99	1.00	1.00	1.00
22	0.26	0.55	0.70	0.82	0.92	0.98	0.99	1.00	1.00	1.00
23	0.29	0.53	0.72	0.88	0.95	0.98	1.00	1.00	1.00	1.00
24	0.34	0.52	0.69	0.81	0.98	1.00	0.98	1.00	1.00	1.00
25	0.28	0.46	0.60	0.78	0.90	0.96	0.99	1.00	1.00	1.00
26	0.27	0.47	0.66	0.77	0.94	0.98	1.00	1.00	1.00	1.00
27	0.34	0.46	0.58	0.74	0.95	0.96	0.99	1.00	1.00	1.00
28	0.28	0.49	0.61	0.73	0.87	0.96	0.99	1.00	1.00	1.00
29	0.33	0.46	0.61	0.74	0.95	0.94	1.00	1.00	1.00	1.00
30	0.27	0.53	0.67	0.83	0.93	0.98	1.00	1.00	1.00	1.00
31	0.09	0.13	0.38	0.40	0.61	0.80	0.93	0.95	1.00	1.00
32	0.06	0.17	0.33	0.51	0.62	0.80	0.93	0.96	0.99	1.00
33	0.03	0.19	0.30	0.46	0.77	0.82	0.87	0.95	1.00	0.99
34	0.04	0.14	0.33	0.42	0.67	0.76	0.93	0.98	1.00	1.00
35	0.09	0.17	0.33	0.45	0.70	0.90	0.96	0.99	1.00	1.00
36	0.04	0.11	0.27	0.54	0.72	0.78	0.92	0.95	1.00	1.00
37	0.12	0.15	0.38	0.57	0.73	0.89	0.96	1.00	0.97	0.99
38	0.04	0.18	0.31	0.51	0.82	0.90	0.99	0.99	1.00	1.00
39	0.11	0.13	0.40	0.50	0.70	0.90	0.96	1.00	1.00	1.00
40	0.12	0.27	0.39	0.49	0.71	0.81	0.93	0.99	1.00	1.00
41	0.14	0.18	0.43	0.58	0.65	0.85	0.95	0.99	0.99	1.00
42	0.11	0.19	0.32	0.64	0.71	0.91	0.95	0.98	1.00	1.00

Table 11: POD according to each scenario and each k2 value, k1 = 5, alpha = 0.01

	k2=1	k2=2	k2=3	k2=4	k2=5	k2=6	k2=7	k2=8	k2=9	k2=10
1	0.17	0.21	0.22	0.43	0.43	0.62	0.80	0.77	0.85	0.88
2	0.06	0.17	0.30	0.34	0.53	0.73	0.76	0.81	0.89	0.94
3	0.15	0.18	0.28	0.46	0.52	0.61	0.73	0.86	0.88	0.95
4	0.05	0.18	0.28	0.48	0.55	0.81	0.85	0.95	0.96	0.98
5	0.07	0.23	0.26	0.38	0.61	0.81	0.88	0.92	0.97	0.99
6	0.09	0.15	0.39	0.47	0.56	0.72	0.94	0.95	0.95	0.99
7	0.23	0.22	0.35	0.42	0.55	0.55	0.56	0.56	0.76	0.77
8	0.18	0.31	0.30	0.43	0.59	0.53	0.66	0.58	0.77	0.84
9	0.26	0.21	0.29	0.43	0.52	0.52	0.52	0.70	0.72	0.76
10	0.14	0.21	0.45	0.42	0.68	0.81	0.85	0.92	0.96	0.99
11	0.15	0.16	0.40	0.47	0.66	0.74	0.88	0.89	0.95	0.99
12	0.13	0.18	0.36	0.43	0.71	0.79	0.84	0.92	1.00	0.98
13	0.04	0.07	0.21	0.34	0.50	0.66	0.72	0.83	0.93	0.99
14	0.02	0.05	0.18	0.28	0.54	0.60	0.73	0.87	0.93	0.94
15	0.05	0.06	0.17	0.28	0.40	0.57	0.74	0.82	0.93	0.91
16	0.04	0.10	0.25	0.36	0.55	0.65	0.83	0.94	0.98	1.00
17	0.08	0.11	0.17	0.36	0.58	0.78	0.88	0.90	0.96	0.99
18	0.04	0.09	0.14	0.34	0.56	0.71	0.80	0.95	0.96	0.97
19	0.17	0.40	0.48	0.60	0.91	0.95	0.97	0.98	1.00	1.00
20	0.29	0.39	0.43	0.73	0.85	0.92	0.99	1.00	1.00	1.00
21	0.25	0.40	0.49	0.76	0.84	0.93	0.98	0.99	0.98	1.00
22	0.34	0.49	0.55	0.77	0.91	0.99	0.99	1.00	0.99	1.00
23	0.28	0.34	0.71	0.83	0.92	0.98	1.00	0.99	1.00	1.00
24	0.29	0.42	0.56	0.75	0.91	0.97	0.99	1.00	1.00	1.00
25	0.28	0.38	0.57	0.78	0.89	0.95	0.98	1.00	1.00	1.00
26	0.25	0.41	0.54	0.73	0.87	0.94	0.99	0.98	1.00	1.00
27	0.28	0.31	0.47	0.81	0.80	0.91	0.98	0.99	1.00	1.00
28	0.19	0.40	0.61	0.77	0.92	0.96	0.99	1.00	1.00	1.00
29	0.24	0.39	0.67	0.82	0.94	0.93	1.00	1.00	1.00	1.00
30	0.35	0.51	0.57	0.83	0.93	0.95	0.98	0.99	1.00	1.00
31	0.08	0.16	0.31	0.47	0.67	0.75	0.87	0.98	1.00	1.00
32	0.07	0.17	0.26	0.38	0.51	0.80	0.91	0.98	1.00	1.00
33	0.05	0.09	0.14	0.43	0.63	0.79	0.89	0.98	1.00	1.00
34	0.04	0.12	0.18	0.40	0.68	0.75	0.91	0.95	1.00	1.00
35	0.09	0.15	0.19	0.37	0.55	0.79	0.91	0.97	1.00	1.00
36	0.10	0.10	0.30	0.45	0.74	0.79	0.93	0.97	1.00	1.00
37	0.07	0.11	0.29	0.45	0.74	0.84	0.94	0.96	0.98	1.00
38	0.07	0.20	0.32	0.52	0.66	0.82	0.97	0.97	1.00	1.00
39	0.08	0.18	0.30	0.52	0.74	0.86	0.93	1.00	1.00	1.00
40	0.08	0.15	0.29	0.54	0.75	0.78	0.96	0.95	0.99	1.00
41	0.10	0.18	0.27	0.53	0.75	0.75	0.96	0.97	0.99	1.00
42	0.06	0.20	0.30	0.59	0.69	0.88	0.95	1.00	1.00	1.00

Table 12: POD according to each scenario and each k2 value, k1 = 10, alpha = 0.01

Overall performances of Periodic Poisson GLM algorithm ($\alpha = 0.05$)

	FPR k1=0	FPR k1=2	FPR k1=3	FPR k1=5	FPR k1=10
1	0.05	0.05	0.05	0.04	0.04
2	0.05	0.05	0.05	0.05	0.04
3	0.05	0.05	0.05	0.05	0.04
4	0.07	0.06	0.06	0.06	0.05
5	0.07	0.06	0.06	0.06	0.05
6	0.07	0.06	0.06	0.06	0.05
7	0.03	0.03	0.03	0.03	0.02
8	0.03	0.03	0.03	0.02	0.02
9	0.03	0.03	0.03	0.03	0.02
10	0.09	0.08	0.08	0.08	0.07
11	0.09	0.09	0.09	0.08	0.07
12	0.09	0.08	0.08	0.08	0.07
13	0.04	0.03	0.03	0.03	0.03
14	0.03	0.03	0.03	0.03	0.02
15	0.04	0.04	0.03	0.03	0.03
16	0.05	0.04	0.04	0.04	0.03
17	0.05	0.04	0.04	0.04	0.03
18	0.04	0.04	0.04	0.04	0.03
19	0.12	0.11	0.11	0.10	0.09
20	0.12	0.11	0.11	0.11	0.10
21	0.11	0.11	0.10	0.10	0.09
22	0.16	0.15	0.15	0.14	0.12
23	0.16	0.16	0.15	0.15	0.13
24	0.15	0.15	0.14	0.14	0.12
25	0.14	0.13	0.13	0.12	0.11
26	0.14	0.13	0.13	0.12	0.11
27	0.14	0.13	0.13	0.12	0.11
28	0.14	0.14	0.13	0.13	0.11
29	0.14	0.13	0.13	0.12	0.11
30	0.14	0.14	0.14	0.13	0.12
31	0.05	0.05	0.05	0.05	0.04
32	0.05	0.05	0.05	0.05	0.04
33	0.05	0.04	0.04	0.04	0.03
34	0.05	0.05	0.05	0.04	0.04
35	0.05	0.05	0.05	0.04	0.04
36	0.06	0.06	0.06	0.05	0.04
37	0.06	0.06	0.06	0.05	0.04
38	0.06	0.05	0.05	0.05	0.04
39	0.07	0.06	0.06	0.06	0.05
40	0.07	0.06	0.06	0.06	0.05
41	0.06	0.06	0.06	0.05	0.05
42	0.06	0.06	0.06	0.06	0.04

Table 13: FPR according to each scenario and each k1 value, alpha = 0.05

	k2=1	k2=2	k2=3	k2=4	k2=5	k2=6	k2=7	k2=8	k2=9	k2=10
1	0.36	0.47	0.55	0.64	0.83	0.90	0.94	0.97	0.99	0.98
2	0.32	0.45	0.53	0.86	0.83	0.85	0.92	0.98	0.98	1.00
3	0.45	0.44	0.56	0.75	0.76	0.82	0.96	0.96	0.97	0.95
4	0.28	0.48	0.64	0.79	0.88	0.94	0.95	1.00	1.00	1.00
5	0.36	0.47	0.67	0.84	0.85	0.95	0.96	0.99	0.99	1.00
6	0.41	0.48	0.61	0.81	0.89	0.95	0.96	1.00	1.00	1.00
7	0.66	0.69	0.74	0.77	0.90	0.86	0.90	0.94	0.90	0.93
8	0.68	0.64	0.70	0.87	0.80	0.85	0.87	0.90	0.95	0.94
9	0.61	0.72	0.66	0.74	0.77	0.89	0.85	0.92	0.94	0.95
10	0.35	0.54	0.64	0.79	0.88	0.92	0.96	0.98	1.00	1.00
11	0.40	0.44	0.74	0.88	0.95	0.95	1.00	1.00	0.97	1.00
12	0.35	0.46	0.69	0.79	0.93	0.98	0.99	0.99	0.98	1.00
13	0.21	0.34	0.53	0.72	0.84	0.90	0.92	0.97	1.00	1.00
14	0.28	0.36	0.53	0.65	0.71	0.89	0.92	0.98	0.98	1.00
15	0.26	0.46	0.47	0.64	0.76	0.88	0.98	0.98	1.00	1.00
16	0.27	0.47	0.58	0.74	0.83	0.98	0.98	1.00	1.00	1.00
17	0.23	0.36	0.54	0.81	0.90	0.96	0.99	1.00	0.99	1.00
18	0.24	0.38	0.59	0.76	0.92	0.94	0.99	0.99	1.00	1.00
19	0.34	0.59	0.79	0.91	0.97	1.00	1.00	1.00	1.00	1.00
20	0.48	0.62	0.82	0.92	0.98	1.00	1.00	1.00	1.00	1.00
21	0.37	0.58	0.82	0.91	0.95	1.00	0.99	1.00	1.00	1.00
22	0.42	0.71	0.89	0.94	1.00	1.00	1.00	1.00	1.00	1.00
23	0.48	0.67	0.85	0.97	0.98	1.00	1.00	1.00	1.00	1.00
24	0.52	0.70	0.89	0.97	0.99	0.99	1.00	1.00	1.00	1.00
25	0.51	0.72	0.86	0.94	0.97	0.99	1.00	1.00	1.00	1.00
26	0.50	0.65	0.88	0.91	1.00	0.98	1.00	1.00	1.00	1.00
27	0.44	0.75	0.80	0.90	0.98	1.00	1.00	1.00	1.00	1.00
28	0.46	0.78	0.90	0.96	1.00	0.99	1.00	1.00	1.00	1.00
29	0.50	0.60	0.83	0.95	0.98	0.99	1.00	1.00	1.00	1.00
30	0.41	0.73	0.81	0.96	0.99	0.99	1.00	1.00	1.00	1.00
31	0.22	0.49	0.54	0.77	0.91	1.00	0.99	1.00	1.00	1.00
32	0.24	0.46	0.68	0.79	0.90	0.99	0.99	1.00	1.00	1.00
33	0.26	0.36	0.63	0.82	0.94	0.97	0.98	1.00	1.00	1.00
34	0.25	0.50	0.64	0.79	0.95	1.00	1.00	1.00	1.00	1.00
35	0.30	0.45	0.68	0.85	0.93	0.99	0.98	1.00	1.00	1.00
36	0.20	0.42	0.63	0.80	0.95	0.98	0.99	1.00	1.00	1.00
37	0.24	0.55	0.68	0.84	0.91	0.96	0.99	1.00	1.00	1.00
38	0.35	0.43	0.66	0.84	0.91	0.95	1.00	1.00	1.00	1.00
39	0.30	0.54	0.70	0.90	0.97	1.00	1.00	1.00	1.00	1.00
40	0.35	0.59	0.69	0.95	0.91	0.97	0.99	1.00	1.00	1.00
41	0.38	0.49	0.63	0.89	0.97	0.96	0.98	1.00	1.00	1.00
42	0.33	0.51	0.72	0.81	0.94	0.98	1.00	1.00	1.00	1.00

Table 14: POD according to each scenario and each k2 value, k1 = 0, alpha = 0.05

	k2=1	k2=2	k2=3	k2=4	k2=5	k2=6	k2=7	k2=8	k2=9	k2=10
1	0.32	0.44	0.65	0.62	0.82	0.85	0.93	0.98	0.97	0.99
2	0.36	0.48	0.55	0.70	0.87	0.90	0.93	0.96	0.96	0.99
3	0.28	0.41	0.63	0.73	0.78	0.90	0.95	0.97	0.97	0.99
4	0.38	0.49	0.61	0.79	0.90	0.94	0.96	1.00	1.00	1.00
5	0.34	0.49	0.67	0.79	0.90	0.95	0.96	0.99	0.99	0.99
6	0.21	0.55	0.63	0.79	0.88	0.92	0.96	0.99	1.00	1.00
7	0.57	0.67	0.72	0.78	0.82	0.85	0.90	0.88	0.93	0.96
8	0.69	0.63	0.68	0.71	0.77	0.79	0.84	0.88	0.94	0.97
9	0.51	0.59	0.68	0.73	0.79	0.86	0.92	0.87	0.91	0.99
10	0.36	0.51	0.71	0.84	0.84	0.97	0.98	0.98	1.00	1.00
11	0.36	0.50	0.74	0.75	0.94	0.93	0.98	1.00	1.00	1.00
12	0.26	0.55	0.71	0.77	0.96	0.97	0.98	0.99	1.00	1.00
13	0.23	0.33	0.51	0.72	0.83	0.88	0.98	0.97	0.99	1.00
14	0.18	0.36	0.56	0.66	0.78	0.86	0.95	0.98	1.00	0.99
15	0.26	0.37	0.45	0.69	0.89	0.91	0.96	0.99	0.97	1.00
16	0.26	0.37	0.52	0.70	0.92	0.96	0.98	1.00	1.00	1.00
17	0.18	0.38	0.59	0.83	0.90	0.96	0.99	0.99	1.00	1.00
18	0.23	0.41	0.59	0.71	0.82	0.93	0.98	0.99	1.00	1.00
19	0.43	0.62	0.73	0.91	0.98	1.00	1.00	1.00	1.00	1.00
20	0.39	0.62	0.79	0.98	0.98	1.00	1.00	1.00	1.00	1.00
21	0.41	0.51	0.79	0.90	0.97	1.00	1.00	1.00	1.00	1.00
22	0.45	0.66	0.85	0.99	0.98	0.99	1.00	1.00	1.00	1.00
23	0.52	0.62	0.88	0.96	0.99	1.00	1.00	1.00	1.00	1.00
24	0.54	0.71	0.90	0.92	0.98	1.00	1.00	1.00	1.00	1.00
25	0.48	0.65	0.76	0.94	1.00	0.98	1.00	1.00	1.00	1.00
26	0.43	0.63	0.86	0.88	0.98	1.00	1.00	1.00	1.00	1.00
27	0.44	0.66	0.76	0.90	0.97	1.00	1.00	1.00	1.00	1.00
28	0.45	0.66	0.79	0.97	0.98	1.00	1.00	1.00	1.00	1.00
29	0.46	0.66	0.73	0.93	0.98	1.00	1.00	1.00	1.00	1.00
30	0.38	0.72	0.79	0.96	0.97	0.99	1.00	1.00	1.00	1.00
31	0.31	0.37	0.58	0.80	0.92	0.98	1.00	1.00	1.00	1.00
32	0.26	0.43	0.66	0.82	0.90	0.98	0.98	0.99	0.99	0.99
33	0.19	0.47	0.60	0.83	0.91	0.94	0.99	1.00	1.00	1.00
34	0.30	0.49	0.64	0.83	0.91	0.97	0.99	1.00	1.00	1.00
35	0.26	0.43	0.52	0.74	0.93	0.98	0.99	1.00	1.00	1.00
36	0.27	0.37	0.73	0.79	0.92	0.96	1.00	1.00	1.00	1.00
37	0.29	0.45	0.66	0.85	0.97	0.98	1.00	1.00	1.00	1.00
38	0.20	0.47	0.71	0.86	0.94	1.00	0.99	1.00	1.00	1.00
39	0.22	0.51	0.64	0.90	0.94	0.98	1.00	1.00	1.00	1.00
40	0.33	0.63	0.65	0.90	0.99	0.99	0.99	0.99	1.00	1.00
41	0.32	0.34	0.69	0.85	0.91	0.99	0.99	1.00	1.00	1.00
42	0.34	0.49	0.69	0.82	0.89	0.99	0.99	1.00	1.00	1.00

Table 15: POD according to each scenario and each k2 value, k1 = 2, alpha = 0.05

	k2=1	k2=2	k2=3	k2=4	k2=5	k2=6	k2=7	k2=8	k2=9	k2=10
1	0.34	0.39	0.57	0.67	0.87	0.85	0.94	0.98	0.95	0.97
2	0.34	0.54	0.60	0.61	0.78	0.92	0.96	0.94	0.99	0.95
3	0.33	0.44	0.55	0.69	0.83	0.88	0.94	0.94	0.96	0.99
4	0.30	0.45	0.55	0.76	0.85	0.97	0.97	0.98	1.00	0.99
5	0.33	0.46	0.61	0.80	0.86	0.90	0.95	0.99	1.00	1.00
6	0.33	0.49	0.62	0.75	0.79	0.95	0.99	0.99	1.00	1.00
7	0.49	0.54	0.70	0.72	0.82	0.85	0.92	0.93	0.90	0.96
8	0.58	0.74	0.80	0.77	0.75	0.87	0.89	0.89	0.92	0.92
9	0.65	0.71	0.66	0.79	0.81	0.84	0.81	0.89	0.91	0.92
10	0.33	0.49	0.64	0.83	0.89	0.97	0.98	1.00	0.98	1.00
11	0.33	0.47	0.64	0.83	0.92	0.95	0.96	0.98	1.00	1.00
12	0.35	0.47	0.66	0.77	0.92	0.90	0.97	1.00	1.00	1.00
13	0.21	0.37	0.48	0.64	0.78	0.87	0.95	0.94	0.99	1.00
14	0.25	0.30	0.54	0.68	0.84	0.87	0.94	0.98	0.98	0.99
15	0.20	0.34	0.56	0.62	0.71	0.89	0.95	0.98	0.99	0.99
16	0.15	0.30	0.64	0.82	0.82	0.93	0.97	0.98	1.00	1.00
17	0.19	0.51	0.66	0.75	0.88	0.95	0.97	0.99	0.99	1.00
18	0.14	0.37	0.57	0.74	0.87	0.98	0.99	1.00	1.00	1.00
19	0.36	0.62	0.83	0.90	0.98	1.00	1.00	1.00	1.00	1.00
20	0.36	0.64	0.79	0.89	0.97	0.98	1.00	1.00	1.00	1.00
21	0.33	0.62	0.82	0.94	0.98	1.00	0.99	1.00	1.00	1.00
22	0.43	0.63	0.85	0.97	1.00	1.00	1.00	1.00	1.00	1.00
23	0.40	0.69	0.87	0.93	1.00	1.00	1.00	1.00	1.00	1.00
24	0.44	0.66	0.85	0.96	1.00	1.00	1.00	1.00	1.00	1.00
25	0.35	0.62	0.84	0.95	0.99	0.99	1.00	1.00	1.00	1.00
26	0.49	0.65	0.87	0.91	0.97	1.00	1.00	1.00	1.00	1.00
27	0.48	0.61	0.81	0.91	0.96	1.00	1.00	1.00	1.00	1.00
28	0.46	0.71	0.83	0.92	1.00	1.00	1.00	1.00	1.00	1.00
29	0.48	0.67	0.84	0.92	0.99	0.99	1.00	1.00	1.00	1.00
30	0.49	0.69	0.84	0.98	0.99	0.98	1.00	1.00	1.00	1.00
31	0.27	0.45	0.63	0.77	0.87	1.00	0.97	1.00	1.00	1.00
32	0.21	0.46	0.59	0.78	0.84	0.94	0.99	1.00	1.00	1.00
33	0.31	0.44	0.59	0.80	0.88	0.93	0.99	1.00	1.00	1.00
34	0.20	0.40	0.58	0.76	0.92	0.98	0.98	1.00	1.00	1.00
35	0.30	0.41	0.66	0.78	0.94	0.95	1.00	1.00	1.00	1.00
36	0.25	0.42	0.68	0.82	0.94	0.97	1.00	1.00	0.99	1.00
37	0.28	0.49	0.77	0.81	0.85	0.96	1.00	1.00	1.00	1.00
38	0.32	0.47	0.60	0.80	0.90	0.98	0.99	1.00	1.00	1.00
39	0.25	0.45	0.71	0.83	0.95	0.97	0.99	1.00	1.00	1.00
40	0.35	0.62	0.70	0.86	0.91	0.99	1.00	1.00	1.00	1.00
41	0.29	0.48	0.62	0.81	0.94	0.98	0.98	1.00	1.00	1.00
42	0.30	0.60	0.65	0.81	0.95	0.99	1.00	1.00	1.00	1.00

Table 16: POD according to each scenario and each k2 value, k1 = 3, alpha = 0.05

	k2=1	k2=2	k2=3	k2=4	k2=5	k2=6	k2=7	k2=8	k2=9	k2=10
1	0.32	0.43	0.63	0.69	0.74	0.83	0.95	1.00	0.98	0.99
2	0.31	0.34	0.60	0.70	0.76	0.88	0.90	0.95	0.97	0.98
3	0.34	0.53	0.55	0.73	0.82	0.87	0.89	0.94	0.98	0.99
4	0.30	0.45	0.59	0.73	0.88	0.95	0.94	1.00	1.00	1.00
5	0.25	0.45	0.67	0.69	0.91	0.93	0.98	0.98	1.00	1.00
6	0.28	0.41	0.60	0.79	0.83	0.95	0.95	0.99	0.99	1.00
7	0.54	0.67	0.60	0.80	0.88	0.73	0.87	0.90	0.94	0.97
8	0.64	0.58	0.75	0.73	0.78	0.74	0.78	0.96	0.94	0.92
9	0.59	0.50	0.75	0.61	0.79	0.84	0.86	0.89	0.91	0.92
10	0.23	0.40	0.68	0.73	0.92	0.94	0.96	0.99	1.00	1.00
11	0.34	0.44	0.65	0.84	0.92	0.95	0.98	0.98	1.00	0.99
12	0.29	0.53	0.70	0.83	0.90	0.98	0.97	1.00	0.99	1.00
13	0.22	0.38	0.53	0.72	0.78	0.88	0.92	0.98	1.00	0.99
14	0.20	0.37	0.51	0.67	0.80	0.89	0.94	0.97	1.00	0.99
15	0.20	0.31	0.58	0.66	0.78	0.90	0.94	0.96	0.97	1.00
16	0.26	0.35	0.52	0.78	0.91	0.95	0.95	0.99	0.99	1.00
17	0.18	0.33	0.61	0.67	0.93	0.97	0.98	1.00	1.00	1.00
18	0.17	0.35	0.53	0.75	0.82	0.91	0.99	0.97	1.00	1.00
19	0.33	0.69	0.74	0.89	0.98	0.99	0.99	1.00	1.00	1.00
20	0.40	0.55	0.78	0.90	0.97	1.00	0.99	1.00	1.00	1.00
21	0.37	0.46	0.70	0.92	0.98	0.97	1.00	1.00	1.00	1.00
22	0.38	0.77	0.85	0.92	1.00	0.99	1.00	1.00	1.00	1.00
23	0.39	0.64	0.85	0.92	0.99	0.99	1.00	1.00	1.00	1.00
24	0.51	0.67	0.83	0.92	1.00	1.00	1.00	1.00	1.00	1.00
25	0.42	0.66	0.81	0.90	0.98	1.00	0.99	1.00	1.00	1.00
26	0.38	0.59	0.83	0.97	1.00	1.00	1.00	1.00	1.00	1.00
27	0.48	0.70	0.79	0.89	0.98	1.00	1.00	1.00	1.00	1.00
28	0.38	0.66	0.83	0.88	0.93	0.98	1.00	1.00	1.00	1.00
29	0.47	0.66	0.80	0.90	0.97	0.97	1.00	1.00	1.00	1.00
30	0.37	0.67	0.86	0.97	0.98	0.99	1.00	1.00	1.00	1.00
31	0.26	0.43	0.64	0.70	0.88	0.96	1.00	1.00	1.00	1.00
32	0.27	0.45	0.62	0.72	0.87	0.92	0.97	1.00	1.00	1.00
33	0.18	0.36	0.60	0.76	0.93	0.94	0.97	0.99	1.00	1.00
34	0.16	0.37	0.59	0.74	0.96	0.95	0.98	0.99	1.00	1.00
35	0.23	0.48	0.63	0.71	0.89	1.00	0.99	1.00	1.00	1.00
36	0.21	0.45	0.60	0.78	0.92	0.98	0.96	0.99	1.00	1.00
37	0.26	0.35	0.72	0.80	0.93	0.99	0.99	1.00	0.99	1.00
38	0.19	0.45	0.61	0.77	0.98	0.99	1.00	1.00	1.00	1.00
39	0.27	0.28	0.76	0.84	0.87	0.99	0.99	1.00	1.00	1.00
40	0.27	0.48	0.72	0.79	0.91	0.98	1.00	1.00	1.00	1.00
41	0.27	0.37	0.66	0.81	0.92	0.97	1.00	1.00	1.00	1.00
42	0.29	0.40	0.64	0.85	0.95	0.99	1.00	1.00	1.00	1.00

Table 17: POD according to each scenario and each k2 value, k1 = 5, alpha = 0.05

	k2=1	k2=2	k2=3	k2=4	k2=5	k2=6	k2=7	k2=8	k2=9	k2=10
1	0.35	0.37	0.44	0.66	0.76	0.83	0.95	0.93	0.92	0.98
2	0.30	0.38	0.58	0.61	0.80	0.86	0.94	0.92	0.95	0.99
3	0.39	0.36	0.51	0.72	0.70	0.81	0.89	0.98	0.99	0.99
4	0.21	0.40	0.54	0.71	0.79	0.94	0.98	1.00	0.99	1.00
5	0.20	0.43	0.53	0.65	0.88	0.95	0.97	0.97	0.99	0.99
6	0.17	0.36	0.64	0.71	0.85	0.91	0.98	1.00	0.99	1.00
7	0.56	0.54	0.65	0.70	0.75	0.79	0.77	0.86	0.94	0.91
8	0.46	0.62	0.62	0.67	0.87	0.77	0.89	0.79	0.91	0.91
9	0.57	0.50	0.66	0.73	0.77	0.78	0.84	0.93	0.89	0.92
10	0.25	0.37	0.66	0.66	0.88	0.94	0.98	0.95	0.98	1.00
11	0.28	0.47	0.63	0.69	0.83	0.87	0.96	0.99	0.98	0.99
12	0.27	0.42	0.62	0.66	0.87	0.92	0.93	0.99	1.00	1.00
13	0.15	0.29	0.44	0.60	0.77	0.87	0.92	0.95	0.98	1.00
14	0.18	0.20	0.49	0.56	0.82	0.83	0.93	0.98	0.97	1.00
15	0.14	0.22	0.49	0.58	0.73	0.86	0.90	0.96	0.97	1.00
16	0.14	0.35	0.54	0.68	0.84	0.84	0.94	1.00	0.99	1.00
17	0.26	0.38	0.49	0.64	0.79	0.95	0.98	0.99	1.00	1.00
18	0.18	0.29	0.35	0.61	0.80	0.89	0.94	0.99	0.99	1.00
19	0.26	0.49	0.70	0.82	0.98	0.99	1.00	1.00	1.00	1.00
20	0.38	0.49	0.70	0.87	0.97	0.98	1.00	1.00	1.00	1.00
21	0.35	0.56	0.77	0.83	0.96	1.00	1.00	1.00	1.00	1.00
22	0.42	0.61	0.73	0.89	0.98	1.00	1.00	1.00	1.00	1.00
23	0.42	0.54	0.83	0.93	0.97	1.00	1.00	1.00	1.00	1.00
24	0.40	0.62	0.77	0.89	0.96	1.00	1.00	1.00	1.00	1.00
25	0.45	0.59	0.74	0.84	0.97	0.99	1.00	1.00	1.00	1.00
26	0.44	0.58	0.72	0.86	0.95	0.99	0.99	1.00	1.00	1.00
27	0.36	0.57	0.69	0.92	0.94	0.97	1.00	1.00	1.00	1.00
28	0.36	0.58	0.78	0.90	0.97	1.00	0.99	1.00	1.00	1.00
29	0.39	0.60	0.86	0.92	0.97	0.98	1.00	1.00	1.00	1.00
30	0.51	0.68	0.78	0.95	0.98	0.98	1.00	1.00	1.00	1.00
31	0.27	0.34	0.59	0.74	0.87	0.95	0.96	0.99	1.00	1.00
32	0.18	0.42	0.53	0.74	0.81	0.96	0.99	0.99	1.00	1.00
33	0.22	0.34	0.47	0.79	0.85	0.92	0.98	0.98	1.00	1.00
34	0.25	0.30	0.50	0.69	0.92	0.96	0.99	1.00	1.00	1.00
35	0.21	0.38	0.55	0.70	0.87	0.97	0.97	1.00	1.00	1.00
36	0.27	0.31	0.64	0.74	0.87	0.96	0.99	1.00	1.00	1.00
37	0.24	0.41	0.60	0.82	0.95	0.94	0.98	0.99	1.00	1.00
38	0.25	0.37	0.72	0.84	0.90	0.96	0.99	0.99	1.00	1.00
39	0.24	0.43	0.54	0.72	0.94	0.97	1.00	1.00	1.00	1.00
40	0.23	0.42	0.60	0.85	0.94	0.94	0.99	1.00	1.00	1.00
41	0.25	0.41	0.51	0.74	0.96	0.97	0.99	1.00	1.00	1.00
42	0.29	0.46	0.56	0.85	0.93	0.99	1.00	1.00	1.00	1.00

Table 18: POD according to each scenario and each k2 value, k1 = 10, alpha = 0.05
