# Supplementary material for: Evaluation and comparison of statistical methods for early temporal detection of outbreaks: A simulation-based study
Source: PLoS One. 2017 Jul 17;12(7):e0181227. doi: 10.1371/journal.pone.0181227 (PMC5513450; doi:10.1371/journal.pone.0181227)
Supplement: S5 Appendix — (PDF) [file pone.0181227.s005.pdf]

Evaluation and Comparison of Statistical Methods for Early Temporal Detection of Outbreaks: a Simulation-Based Study

Appendix S5: Overall performances of Periodic Negative Binomial GLM algorithm ($\alpha = 0.001, 0.01$ and 0.05)

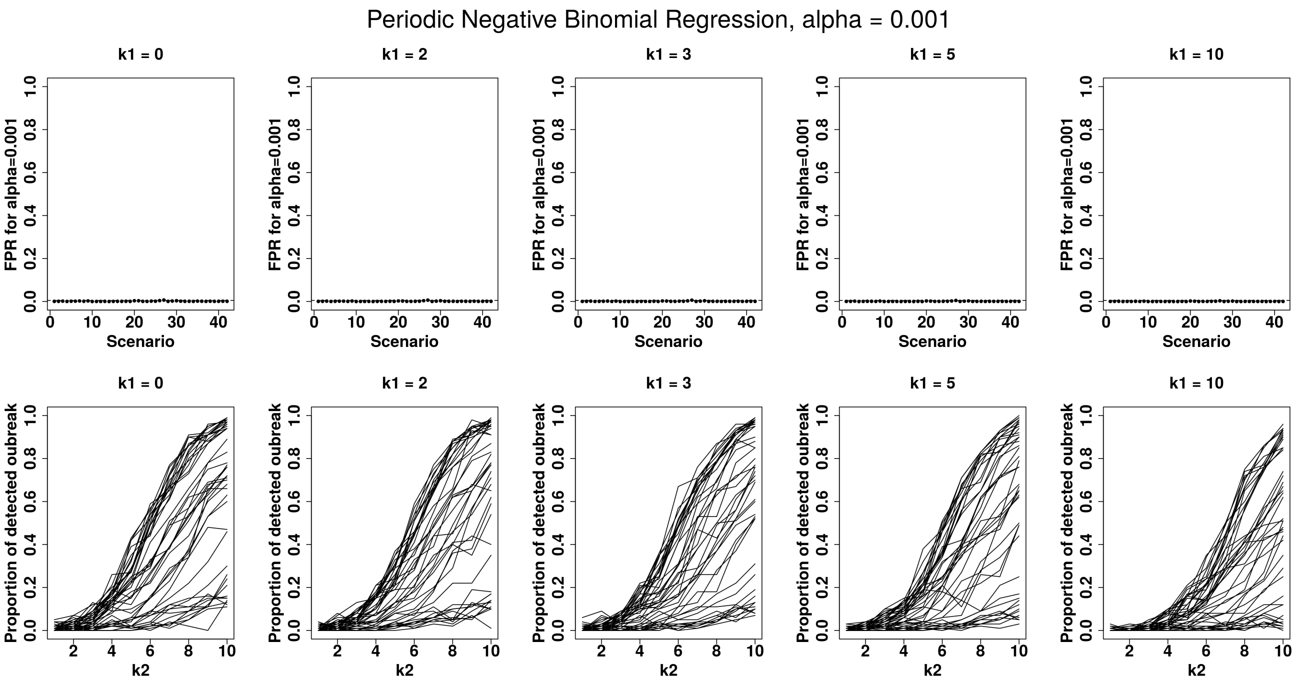

Figure 1: Periodic Negative Binomial GLM algorithm performances for $\alpha = 0.001$ by increasing past outbreak amplitude $k_1 = 0$, 2, 3, 5 or 10 with (i) on the first row: false positive rate for 42 simulated scenarios, (ii) on the second row: probability of detection for 42 simulated scenarios (each curve corresponding to a scenario) by increasing current outbreak amplitude $k_2 = 1$ to 10.

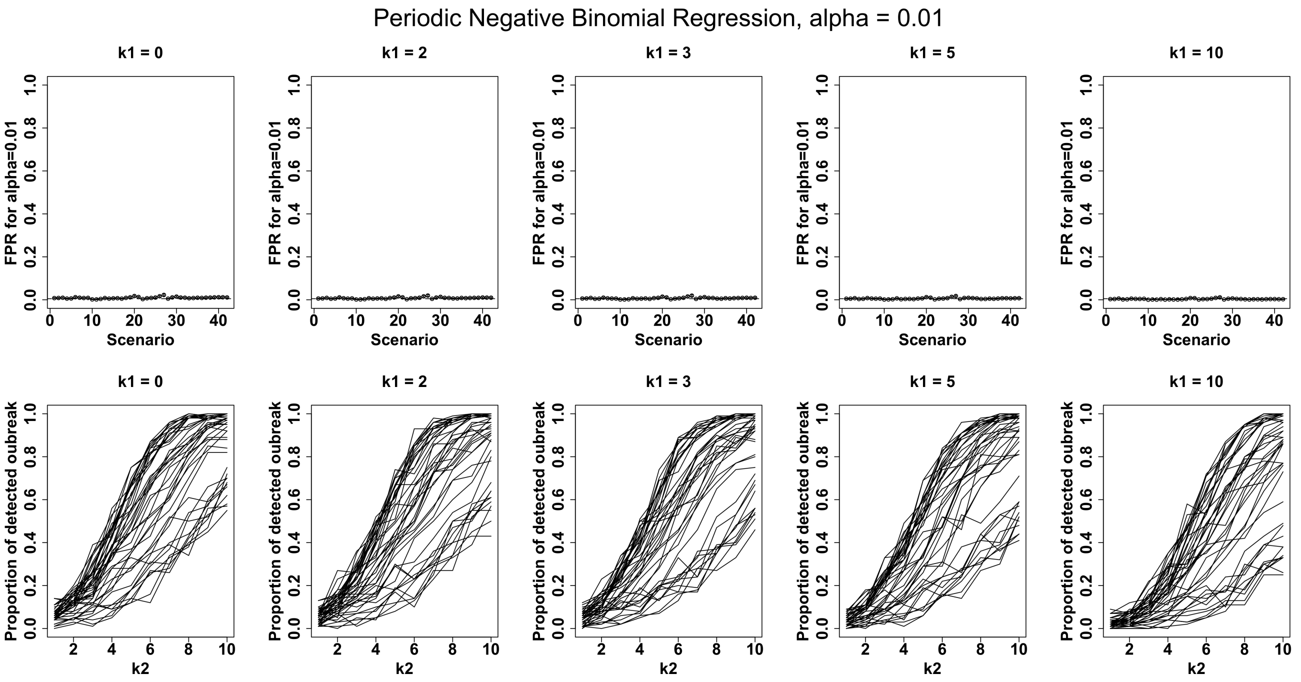

Figure 2: Periodic Negative Binomial GLM algorithm performances for $\alpha = 0.01$ by increasing past outbreak amplitude $k_1 = 0$, 2, 3, 5 or 10 with (i) on the first row: false positive rate for 42 simulated scenarios, (ii) on the second row: probability of detection for 42 simulated scenarios (each curve corresponding to a scenario) by increasing current outbreak amplitude $k_2 = 1$ to 10.

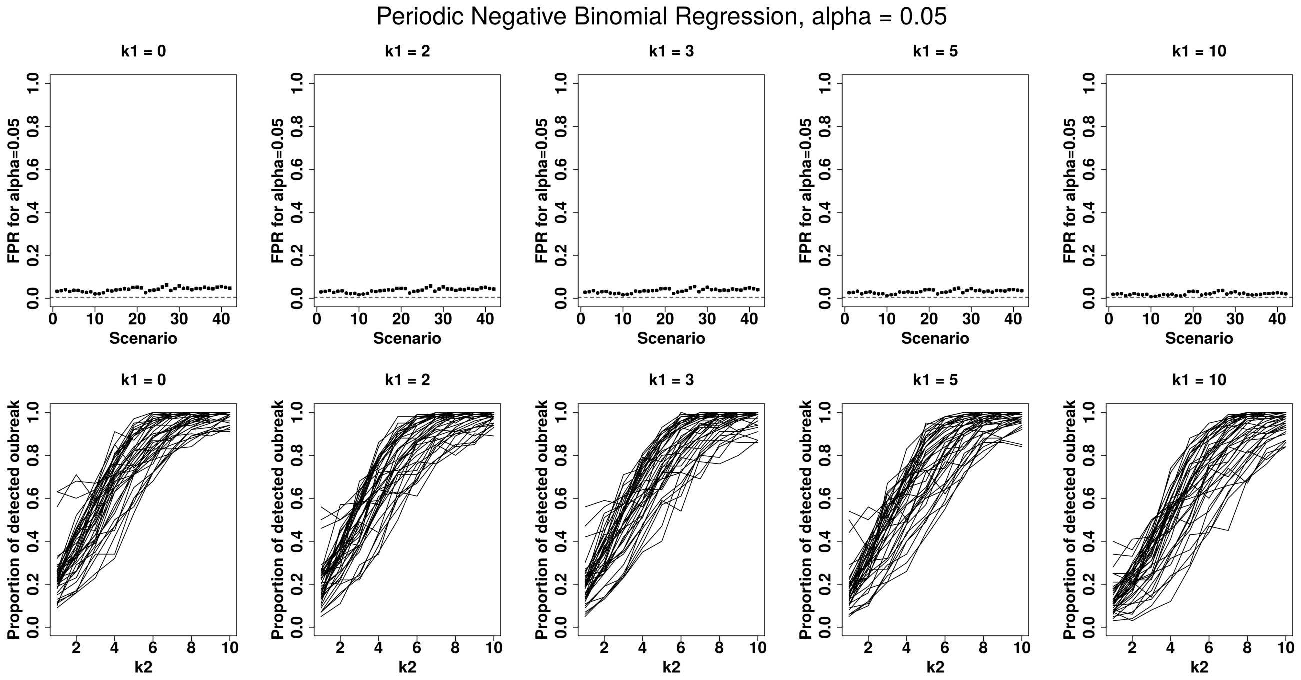

Figure 3: Periodic Negative Binomial GLM algorithm performances for $\alpha = 0.05$ by increasing past outbreak amplitude $k_1 = 0$, 2, 3, 5 or 10 with (i) on the first row: false positive rate for 42 simulated scenarios, (ii) on the second row: probability of detection for 42 simulated scenarios (each curve corresponding to a scenario) by increasing current outbreak amplitude $k_2 = 1$ to 10.

Overall performances of Periodic Negative Binomial GLM algorithm ($\alpha = 0.001$)

	FPR k1=0	FPR k1=2	FPR k1=3	FPR k1=5	FPR k1=10
1	0.00	0.00	0.00	0.00	0.00
2	0.00	0.00	0.00	0.00	0.00
3	0.00	0.00	0.00	0.00	0.00
4	0.00	0.00	0.00	0.00	0.00
5	0.00	0.00	0.00	0.00	0.00
6	0.00	0.00	0.00	0.00	0.00
7	0.00	0.00	0.00	0.00	0.00
8	0.00	0.00	0.00	0.00	0.00
9	0.00	0.00	0.00	0.00	0.00
10	0.00	0.00	0.00	0.00	0.00
11	0.00	0.00	0.00	0.00	0.00
12	0.00	0.00	0.00	0.00	0.00
13	0.00	0.00	0.00	0.00	0.00
14	0.00	0.00	0.00	0.00	0.00
15	0.00	0.00	0.00	0.00	0.00
16	0.00	0.00	0.00	0.00	0.00
17	0.00	0.00	0.00	0.00	0.00
18	0.00	0.00	0.00	0.00	0.00
19	0.00	0.00	0.00	0.00	0.00
20	0.00	0.00	0.00	0.00	0.00
21	0.00	0.00	0.00	0.00	0.00
22	0.00	0.00	0.00	0.00	0.00
23	0.00	0.00	0.00	0.00	0.00
24	0.00	0.00	0.00	0.00	0.00
25	0.00	0.00	0.00	0.00	0.00
26	0.00	0.00	0.00	0.00	0.00
27	0.01	0.01	0.01	0.00	0.00
28	0.00	0.00	0.00	0.00	0.00
29	0.00	0.00	0.00	0.00	0.00
30	0.00	0.00	0.00	0.00	0.00
31	0.00	0.00	0.00	0.00	0.00
32	0.00	0.00	0.00	0.00	0.00
33	0.00	0.00	0.00	0.00	0.00
34	0.00	0.00	0.00	0.00	0.00
35	0.00	0.00	0.00	0.00	0.00
36	0.00	0.00	0.00	0.00	0.00
37	0.00	0.00	0.00	0.00	0.00
38	0.00	0.00	0.00	0.00	0.00
39	0.00	0.00	0.00	0.00	0.00
40	0.00	0.00	0.00	0.00	0.00
41	0.00	0.00	0.00	0.00	0.00
42	0.00	0.00	0.00	0.00	0.00

Table 1: FPR according to each scenario and each k1 value, alpha = 0.001

	k2=1	k2=2	k2=3	k2=4	k2=5	k2=6	k2=7	k2=8	k2=9	k2=10
1	0.00	0.01	0.01	0.02	0.05	0.08	0.19	0.34	0.48	0.47
2	0.02	0.02	0.02	0.09	0.10	0.15	0.27	0.37	0.53	0.63
3	0.04	0.01	0.03	0.11	0.18	0.22	0.36	0.44	0.60	0.68
4	0.00	0.01	0.01	0.03	0.04	0.16	0.26	0.36	0.53	0.60
5	0.01	0.00	0.04	0.11	0.08	0.15	0.29	0.34	0.66	0.66
6	0.01	0.03	0.04	0.19	0.15	0.27	0.39	0.51	0.67	0.76
7	0.01	0.02	0.01	0.07	0.02	0.07	0.10	0.11	0.11	0.24
8	0.01	0.03	0.01	0.03	0.02	0.09	0.08	0.09	0.17	0.12
9	0.00	0.01	0.00	0.03	0.06	0.07	0.12	0.11	0.15	0.15
10	0.00	0.00	0.00	0.00	0.00	0.01	0.03	0.05	0.10	0.11
11	0.00	0.00	0.01	0.02	0.01	0.03	0.01	0.05	0.12	0.21
12	0.01	0.00	0.00	0.02	0.01	0.02	0.06	0.09	0.14	0.16
13	0.00	0.04	0.03	0.13	0.23	0.29	0.51	0.57	0.78	0.83
14	0.04	0.04	0.05	0.12	0.22	0.31	0.49	0.63	0.75	0.78
15	0.04	0.04	0.06	0.15	0.20	0.41	0.53	0.66	0.78	0.89
16	0.02	0.05	0.08	0.13	0.22	0.49	0.61	0.82	0.91	0.95
17	0.00	0.02	0.04	0.18	0.35	0.45	0.62	0.80	0.89	0.96
18	0.01	0.05	0.07	0.15	0.24	0.49	0.66	0.73	0.87	0.94
19	0.00	0.01	0.00	0.00	0.00	0.02	0.03	0.07	0.11	0.13
20	0.00	0.00	0.02	0.02	0.02	0.04	0.12	0.15	0.16	0.15
21	0.01	0.01	0.01	0.04	0.05	0.09	0.15	0.22	0.32	0.46
22	0.00	0.00	0.00	0.00	0.02	0.00	0.04	0.02	0.00	0.14
23	0.00	0.00	0.00	0.00	0.02	0.04	0.02	0.09	0.11	0.26
24	0.01	0.02	0.00	0.05	0.01	0.03	0.10	0.14	0.21	0.30
25	0.02	0.03	0.04	0.08	0.16	0.23	0.39	0.53	0.68	0.70
26	0.01	0.01	0.07	0.14	0.25	0.30	0.49	0.53	0.64	0.77
27	0.05	0.07	0.12	0.14	0.23	0.32	0.47	0.62	0.65	0.76
28	0.00	0.00	0.03	0.03	0.06	0.15	0.23	0.38	0.61	0.72
29	0.01	0.02	0.02	0.14	0.12	0.26	0.39	0.54	0.63	0.72
30	0.02	0.06	0.03	0.12	0.15	0.33	0.38	0.57	0.69	0.71
31	0.01	0.04	0.06	0.13	0.28	0.49	0.68	0.90	0.88	0.94
32	0.00	0.01	0.11	0.20	0.27	0.54	0.63	0.84	0.95	0.98
33	0.00	0.04	0.09	0.16	0.29	0.46	0.70	0.79	0.87	0.97
34	0.00	0.02	0.09	0.18	0.42	0.57	0.67	0.74	0.91	0.99
35	0.01	0.02	0.05	0.18	0.28	0.54	0.67	0.87	0.91	0.98
36	0.03	0.00	0.08	0.18	0.39	0.44	0.72	0.86	0.91	0.94
37	0.00	0.07	0.05	0.26	0.27	0.50	0.76	0.86	0.95	0.98
38	0.01	0.02	0.09	0.16	0.33	0.55	0.72	0.90	0.91	0.97
39	0.01	0.03	0.13	0.09	0.42	0.52	0.66	0.83	0.95	0.98
40	0.01	0.04	0.09	0.17	0.28	0.52	0.73	0.91	0.92	0.99
41	0.02	0.03	0.09	0.21	0.43	0.56	0.77	0.86	0.94	0.99
42	0.01	0.01	0.07	0.17	0.32	0.59	0.65	0.77	0.96	0.97

Table 2: POD according to each scenario and each k2 value, k1 = 0, alpha = 0.001

	k2=1	k2=2	k2=3	k2=4	k2=5	k2=6	k2=7	k2=8	k2=9	k2=10
1	0.00	0.00	0.04	0.02	0.08	0.07	0.18	0.37	0.44	0.40
2	0.00	0.05	0.03	0.05	0.13	0.18	0.27	0.40	0.35	0.54
3	0.03	0.03	0.08	0.07	0.13	0.24	0.33	0.46	0.54	0.68
4	0.00	0.00	0.00	0.03	0.08	0.10	0.19	0.36	0.38	0.59
5	0.00	0.02	0.04	0.04	0.13	0.15	0.29	0.41	0.42	0.62
6	0.01	0.02	0.06	0.17	0.19	0.20	0.44	0.56	0.68	0.65
7	0.04	0.00	0.01	0.01	0.03	0.03	0.06	0.12	0.12	0.13
8	0.03	0.01	0.01	0.01	0.02	0.04	0.06	0.05	0.06	0.10
9	0.00	0.00	0.02	0.01	0.04	0.08	0.09	0.04	0.13	0.10
10	0.01	0.00	0.00	0.00	0.04	0.01	0.05	0.03	0.09	0.14
11	0.00	0.00	0.01	0.00	0.01	0.04	0.04	0.05	0.12	0.10
12	0.00	0.00	0.00	0.00	0.02	0.03	0.08	0.10	0.11	0.17
13	0.01	0.04	0.03	0.08	0.24	0.29	0.51	0.65	0.79	0.83
14	0.01	0.01	0.06	0.10	0.21	0.28	0.49	0.64	0.81	0.87
15	0.01	0.02	0.06	0.15	0.33	0.38	0.48	0.62	0.67	0.82
16	0.01	0.01	0.04	0.10	0.23	0.45	0.58	0.66	0.95	0.91
17	0.00	0.02	0.06	0.16	0.26	0.41	0.66	0.79	0.93	0.91
18	0.01	0.08	0.03	0.11	0.29	0.35	0.55	0.76	0.83	0.98
19	0.00	0.01	0.00	0.00	0.01	0.04	0.06	0.09	0.06	0.11
20	0.03	0.03	0.02	0.01	0.02	0.06	0.11	0.05	0.19	0.18
21	0.00	0.01	0.03	0.04	0.05	0.09	0.13	0.22	0.22	0.35
22	0.01	0.00	0.00	0.00	0.00	0.01	0.03	0.02	0.07	0.01
23	0.00	0.00	0.00	0.02	0.02	0.02	0.05	0.06	0.05	0.14
24	0.01	0.00	0.01	0.01	0.03	0.05	0.06	0.10	0.11	0.17
25	0.00	0.01	0.02	0.04	0.10	0.19	0.30	0.49	0.65	0.78
26	0.02	0.04	0.06	0.07	0.16	0.31	0.44	0.45	0.65	0.77
27	0.02	0.06	0.06	0.12	0.22	0.37	0.48	0.62	0.65	0.77
28	0.00	0.00	0.00	0.03	0.09	0.14	0.20	0.29	0.56	0.74
29	0.03	0.01	0.02	0.08	0.11	0.21	0.35	0.40	0.57	0.71
30	0.02	0.04	0.04	0.15	0.19	0.33	0.39	0.63	0.67	0.78
31	0.02	0.04	0.05	0.21	0.24	0.51	0.69	0.78	0.89	0.98
32	0.00	0.07	0.13	0.15	0.26	0.44	0.67	0.76	0.85	0.96
33	0.00	0.03	0.09	0.15	0.35	0.45	0.64	0.87	0.90	0.94
34	0.01	0.03	0.09	0.16	0.31	0.44	0.70	0.82	0.92	0.95
35	0.00	0.04	0.05	0.10	0.25	0.46	0.65	0.82	0.98	0.95
36	0.00	0.02	0.08	0.17	0.21	0.51	0.67	0.89	0.91	0.98
37	0.02	0.02	0.10	0.16	0.37	0.46	0.64	0.82	0.92	0.97
38	0.00	0.03	0.08	0.18	0.30	0.59	0.75	0.85	0.94	0.98
39	0.00	0.04	0.09	0.15	0.31	0.54	0.65	0.88	0.94	0.97
40	0.00	0.05	0.04	0.17	0.31	0.54	0.77	0.88	0.91	0.99
41	0.00	0.01	0.04	0.21	0.29	0.57	0.72	0.88	0.95	0.95
42	0.04	0.01	0.09	0.18	0.35	0.52	0.73	0.85	0.95	0.98

Table 3: POD according to each scenario and each k2 value, k1 = 2, alpha = 0.001

	k2=1	k2=2	k2=3	k2=4	k2=5	k2=6	k2=7	k2=8	k2=9	k2=10
1	0.01	0.02	0.02	0.02	0.07	0.11	0.13	0.28	0.37	0.52
2	0.01	0.03	0.02	0.12	0.10	0.16	0.26	0.26	0.42	0.52
3	0.00	0.07	0.04	0.09	0.13	0.23	0.38	0.50	0.51	0.60
4	0.00	0.00	0.00	0.01	0.05	0.08	0.18	0.18	0.40	0.53
5	0.00	0.00	0.04	0.06	0.10	0.13	0.29	0.37	0.49	0.54
6	0.02	0.04	0.09	0.12	0.13	0.16	0.47	0.43	0.56	0.76
7	0.02	0.00	0.01	0.00	0.03	0.03	0.03	0.04	0.12	0.08
8	0.00	0.02	0.02	0.01	0.00	0.03	0.04	0.04	0.09	0.16
9	0.02	0.02	0.01	0.07	0.02	0.05	0.05	0.09	0.06	0.13
10	0.00	0.00	0.00	0.00	0.00	0.03	0.02	0.06	0.10	0.13
11	0.00	0.01	0.00	0.00	0.01	0.01	0.04	0.07	0.04	0.10
12	0.00	0.00	0.00	0.01	0.03	0.02	0.01	0.04	0.10	0.11
13	0.00	0.03	0.07	0.08	0.23	0.31	0.53	0.53	0.78	0.85
14	0.01	0.00	0.03	0.05	0.18	0.22	0.52	0.63	0.72	0.76
15	0.00	0.02	0.05	0.12	0.16	0.31	0.40	0.65	0.71	0.80
16	0.00	0.03	0.07	0.17	0.17	0.30	0.59	0.66	0.89	0.85
17	0.01	0.00	0.08	0.11	0.20	0.41	0.52	0.70	0.84	0.88
18	0.00	0.03	0.04	0.21	0.20	0.41	0.62	0.69	0.87	0.90
19	0.00	0.01	0.02	0.00	0.02	0.02	0.02	0.05	0.07	0.10
20	0.02	0.02	0.00	0.03	0.09	0.05	0.08	0.13	0.17	0.26
21	0.01	0.00	0.02	0.00	0.04	0.07	0.10	0.17	0.22	0.31
22	0.00	0.00	0.00	0.00	0.02	0.00	0.01	0.03	0.05	0.07
23	0.00	0.00	0.00	0.01	0.04	0.00	0.04	0.07	0.11	0.08
24	0.01	0.00	0.00	0.03	0.04	0.07	0.11	0.11	0.12	0.19
25	0.00	0.00	0.03	0.04	0.12	0.17	0.25	0.42	0.61	0.72
26	0.06	0.09	0.03	0.11	0.14	0.27	0.29	0.49	0.56	0.74
27	0.04	0.02	0.06	0.15	0.18	0.30	0.47	0.65	0.67	0.77
28	0.00	0.00	0.00	0.02	0.01	0.11	0.24	0.37	0.52	0.61
29	0.01	0.02	0.06	0.10	0.11	0.21	0.26	0.40	0.52	0.69
30	0.03	0.02	0.06	0.10	0.13	0.26	0.38	0.50	0.63	0.70
31	0.00	0.06	0.05	0.10	0.28	0.57	0.66	0.78	0.93	0.96
32	0.01	0.01	0.04	0.13	0.35	0.48	0.70	0.79	0.91	0.99
33	0.01	0.01	0.07	0.22	0.25	0.52	0.68	0.78	0.92	0.95
34	0.00	0.01	0.05	0.14	0.25	0.49	0.59	0.79	0.93	0.99
35	0.00	0.03	0.05	0.17	0.35	0.45	0.67	0.75	0.89	0.98
36	0.00	0.02	0.09	0.12	0.28	0.43	0.66	0.79	0.88	0.95
37	0.01	0.03	0.08	0.14	0.29	0.49	0.71	0.83	0.93	0.98
38	0.01	0.00	0.05	0.12	0.34	0.67	0.71	0.81	0.92	0.97
39	0.01	0.05	0.07	0.19	0.31	0.49	0.65	0.86	0.96	0.96
40	0.01	0.03	0.07	0.18	0.27	0.51	0.69	0.84	0.94	0.97
41	0.01	0.02	0.08	0.20	0.24	0.48	0.63	0.79	0.87	0.98
42	0.00	0.03	0.04	0.17	0.36	0.55	0.76	0.87	0.91	0.98

Table 4: POD according to each scenario and each k2 value, k1 = 3, alpha = 0.001

	k2=1	k2=2	k2=3	k2=4	k2=5	k2=6	k2=7	k2=8	k2=9	k2=10
1	0.00	0.00	0.00	0.06	0.11	0.09	0.18	0.22	0.28	0.49
2	0.00	0.01	0.03	0.06	0.04	0.21	0.11	0.28	0.34	0.44
3	0.03	0.02	0.08	0.08	0.13	0.21	0.38	0.44	0.47	0.67
4	0.00	0.00	0.00	0.02	0.04	0.06	0.07	0.26	0.25	0.49
5	0.01	0.01	0.02	0.04	0.06	0.18	0.14	0.32	0.40	0.50
6	0.01	0.01	0.04	0.08	0.15	0.25	0.36	0.45	0.54	0.65
7	0.01	0.01	0.01	0.04	0.02	0.02	0.02	0.05	0.08	0.06
8	0.01	0.01	0.01	0.01	0.02	0.05	0.01	0.05	0.05	0.10
9	0.00	0.01	0.02	0.01	0.04	0.03	0.03	0.06	0.09	0.05
10	0.00	0.00	0.00	0.01	0.00	0.01	0.00	0.03	0.06	0.05
11	0.00	0.00	0.00	0.00	0.00	0.02	0.02	0.07	0.07	0.14
12	0.00	0.01	0.00	0.00	0.01	0.00	0.03	0.03	0.03	0.07
13	0.01	0.02	0.08	0.09	0.11	0.31	0.40	0.62	0.71	0.76
14	0.00	0.01	0.03	0.07	0.21	0.24	0.41	0.61	0.71	0.81
15	0.01	0.04	0.05	0.14	0.16	0.23	0.39	0.53	0.72	0.85
16	0.00	0.03	0.04	0.09	0.18	0.39	0.42	0.57	0.80	0.90
17	0.00	0.02	0.06	0.13	0.22	0.37	0.54	0.65	0.84	0.90
18	0.00	0.02	0.07	0.10	0.21	0.34	0.56	0.65	0.81	0.86
19	0.00	0.00	0.00	0.00	0.01	0.00	0.02	0.03	0.05	0.09
20	0.00	0.00	0.03	0.02	0.01	0.03	0.05	0.09	0.12	0.15
21	0.02	0.00	0.02	0.03	0.04	0.06	0.14	0.09	0.21	0.25
22	0.00	0.00	0.00	0.00	0.00	0.01	0.01	0.02	0.01	0.03
23	0.01	0.00	0.00	0.01	0.01	0.01	0.03	0.05	0.08	0.12
24	0.00	0.01	0.01	0.00	0.03	0.02	0.05	0.09	0.15	0.17
25	0.01	0.00	0.03	0.04	0.07	0.15	0.27	0.46	0.59	0.68
26	0.01	0.04	0.01	0.06	0.19	0.25	0.34	0.51	0.53	0.62
27	0.03	0.04	0.05	0.09	0.16	0.32	0.38	0.62	0.72	0.76
28	0.00	0.00	0.00	0.04	0.03	0.09	0.23	0.27	0.51	0.63
29	0.00	0.02	0.02	0.04	0.10	0.21	0.35	0.42	0.49	0.65
30	0.01	0.03	0.09	0.11	0.19	0.18	0.40	0.44	0.52	0.79
31	0.01	0.01	0.08	0.09	0.22	0.35	0.60	0.77	0.84	0.94
32	0.00	0.01	0.06	0.14	0.32	0.46	0.61	0.71	0.90	0.95
33	0.00	0.04	0.08	0.13	0.22	0.36	0.61	0.81	0.86	0.88
34	0.00	0.00	0.04	0.10	0.21	0.37	0.68	0.79	0.89	0.91
35	0.01	0.00	0.04	0.07	0.17	0.46	0.66	0.69	0.90	0.92
36	0.01	0.01	0.08	0.12	0.24	0.35	0.55	0.82	0.83	0.98
37	0.02	0.00	0.02	0.11	0.25	0.51	0.66	0.82	0.83	0.97
38	0.01	0.04	0.04	0.10	0.38	0.47	0.67	0.83	0.93	0.99
39	0.02	0.02	0.06	0.07	0.27	0.42	0.60	0.77	0.93	1.00
40	0.01	0.02	0.10	0.10	0.22	0.48	0.71	0.83	0.92	0.99
41	0.00	0.02	0.10	0.15	0.18	0.50	0.64	0.82	0.90	0.98
42	0.00	0.01	0.05	0.13	0.27	0.46	0.71	0.83	0.92	0.96

Table 5: POD according to each scenario and each k2 value, k1 = 5, alpha = 0.001

	k2=1	k2=2	k2=3	k2=4	k2=5	k2=6	k2=7	k2=8	k2=9	k2=10
1	0.00	0.00	0.01	0.01	0.01	0.05	0.08	0.12	0.18	0.29
2	0.00	0.01	0.02	0.02	0.09	0.08	0.08	0.17	0.23	0.35
3	0.02	0.01	0.01	0.05	0.07	0.11	0.16	0.30	0.35	0.42
4	0.00	0.00	0.01	0.01	0.04	0.06	0.08	0.08	0.19	0.25
5	0.00	0.01	0.00	0.03	0.02	0.07	0.14	0.18	0.24	0.44
6	0.01	0.02	0.04	0.03	0.08	0.14	0.18	0.23	0.38	0.48
7	0.01	0.00	0.02	0.01	0.01	0.01	0.02	0.00	0.04	0.02
8	0.00	0.00	0.00	0.00	0.01	0.01	0.04	0.03	0.08	0.05
9	0.00	0.01	0.00	0.03	0.00	0.01	0.03	0.06	0.07	0.03
10	0.00	0.00	0.00	0.00	0.00	0.00	0.00	0.01	0.03	0.02
11	0.00	0.00	0.00	0.00	0.00	0.00	0.01	0.01	0.02	0.01
12	0.00	0.00	0.01	0.00	0.01	0.01	0.00	0.06	0.01	0.07
13	0.01	0.00	0.02	0.05	0.05	0.22	0.29	0.41	0.52	0.67
14	0.00	0.01	0.01	0.04	0.16	0.14	0.28	0.37	0.57	0.65
15	0.00	0.00	0.02	0.05	0.07	0.19	0.34	0.43	0.52	0.64
16	0.00	0.00	0.00	0.04	0.05	0.13	0.26	0.42	0.58	0.74
17	0.00	0.00	0.02	0.05	0.08	0.16	0.25	0.35	0.51	0.69
18	0.00	0.00	0.01	0.02	0.09	0.15	0.29	0.39	0.55	0.72
19	0.00	0.00	0.00	0.00	0.00	0.02	0.00	0.02	0.03	0.04
20	0.00	0.00	0.00	0.01	0.01	0.01	0.02	0.07	0.12	0.12
21	0.00	0.00	0.01	0.01	0.02	0.02	0.02	0.05	0.10	0.16
22	0.00	0.00	0.00	0.02	0.00	0.01	0.01	0.00	0.04	0.00
23	0.00	0.00	0.00	0.03	0.02	0.00	0.01	0.03	0.06	0.03
24	0.00	0.00	0.01	0.00	0.04	0.04	0.03	0.02	0.08	0.12
25	0.00	0.01	0.01	0.04	0.03	0.10	0.13	0.19	0.43	0.46
26	0.02	0.00	0.05	0.08	0.06	0.14	0.31	0.41	0.42	0.47
27	0.03	0.00	0.04	0.10	0.12	0.22	0.38	0.44	0.49	0.51
28	0.00	0.00	0.00	0.02	0.01	0.08	0.02	0.16	0.27	0.51
29	0.00	0.00	0.02	0.02	0.07	0.12	0.29	0.31	0.44	0.52
30	0.01	0.03	0.02	0.09	0.06	0.15	0.25	0.36	0.45	0.62
31	0.00	0.01	0.02	0.05	0.19	0.24	0.41	0.74	0.77	0.90
32	0.01	0.00	0.04	0.09	0.16	0.21	0.41	0.71	0.81	0.85
33	0.01	0.00	0.02	0.03	0.12	0.21	0.33	0.65	0.75	0.89
34	0.00	0.01	0.02	0.02	0.15	0.14	0.38	0.56	0.75	0.85
35	0.00	0.00	0.02	0.02	0.16	0.24	0.29	0.57	0.76	0.84
36	0.00	0.00	0.03	0.03	0.17	0.19	0.43	0.58	0.74	0.91
37	0.00	0.00	0.01	0.08	0.16	0.35	0.44	0.66	0.78	0.93
38	0.01	0.02	0.02	0.07	0.09	0.34	0.50	0.72	0.76	0.94
39	0.00	0.01	0.01	0.09	0.21	0.29	0.49	0.75	0.86	0.94
40	0.00	0.00	0.02	0.07	0.20	0.25	0.50	0.60	0.76	0.92
41	0.00	0.01	0.02	0.10	0.16	0.21	0.44	0.67	0.86	0.93
42	0.00	0.01	0.02	0.09	0.10	0.28	0.45	0.70	0.85	0.96

Table 6: POD according to each scenario and each k2 value, k1 = 10, alpha = 0.001

Overall performances of Periodic Negative Binomial GLM algorithm ($\alpha = 0.01$)

	FPR k1=0	FPR k1=2	FPR k1=3	FPR k1=5	FPR k1=10
1	0.01	0.01	0.01	0.00	0.00
2	0.01	0.01	0.01	0.01	0.00
3	0.01	0.01	0.01	0.01	0.01
4	0.01	0.00	0.00	0.00	0.00
5	0.01	0.01	0.01	0.00	0.00
6	0.01	0.01	0.01	0.01	0.01
7	0.01	0.01	0.01	0.01	0.00
8	0.01	0.01	0.01	0.01	0.00
9	0.01	0.01	0.01	0.01	0.00
10	0.00	0.00	0.00	0.00	0.00
11	0.00	0.00	0.00	0.00	0.00
12	0.00	0.00	0.00	0.00	0.00
13	0.01	0.01	0.01	0.01	0.00
14	0.01	0.00	0.00	0.00	0.00
15	0.01	0.01	0.01	0.00	0.00
16	0.01	0.01	0.01	0.00	0.00
17	0.01	0.00	0.00	0.00	0.00
18	0.01	0.01	0.01	0.01	0.00
19	0.01	0.01	0.01	0.01	0.00
20	0.02	0.02	0.01	0.01	0.01
21	0.01	0.01	0.01	0.01	0.01
22	0.00	0.00	0.00	0.00	0.00
23	0.01	0.01	0.01	0.01	0.00
24	0.01	0.01	0.01	0.01	0.00
25	0.01	0.01	0.01	0.01	0.01
26	0.02	0.02	0.02	0.01	0.01
27	0.02	0.02	0.02	0.02	0.01
28	0.01	0.00	0.00	0.00	0.00
29	0.01	0.01	0.01	0.01	0.00
30	0.01	0.01	0.01	0.01	0.01
31	0.01	0.01	0.01	0.01	0.00
32	0.01	0.01	0.01	0.01	0.00
33	0.01	0.01	0.01	0.00	0.00
34	0.01	0.01	0.00	0.00	0.00
35	0.01	0.01	0.01	0.01	0.00
36	0.01	0.01	0.01	0.00	0.00
37	0.01	0.01	0.01	0.01	0.00
38	0.01	0.01	0.01	0.01	0.00
39	0.01	0.01	0.01	0.01	0.00
40	0.01	0.01	0.01	0.01	0.00
41	0.01	0.01	0.01	0.01	0.00
42	0.01	0.01	0.01	0.01	0.00

Table 7: FPR according to each scenario and each k1 value, alpha = 0.01

	k2=1	k2=2	k2=3	k2=4	k2=5	k2=6	k2=7	k2=8	k2=9	k2=10
1	0.07	0.12	0.12	0.18	0.35	0.47	0.55	0.70	0.85	0.84
2	0.10	0.13	0.15	0.34	0.45	0.52	0.60	0.76	0.87	0.94
3	0.11	0.15	0.21	0.34	0.45	0.53	0.61	0.82	0.88	0.88
4	0.02	0.05	0.07	0.22	0.35	0.48	0.62	0.75	0.83	0.92
5	0.05	0.06	0.14	0.35	0.37	0.43	0.64	0.79	0.90	0.96
6	0.08	0.11	0.22	0.38	0.51	0.68	0.78	0.83	0.95	0.95
7	0.11	0.17	0.20	0.29	0.33	0.30	0.40	0.44	0.53	0.58
8	0.14	0.12	0.17	0.28	0.28	0.35	0.39	0.34	0.56	0.57
9	0.14	0.13	0.16	0.23	0.27	0.38	0.52	0.50	0.52	0.62
10	0.01	0.03	0.01	0.08	0.12	0.16	0.34	0.39	0.45	0.55
11	0.00	0.02	0.06	0.11	0.14	0.26	0.28	0.43	0.58	0.66
12	0.01	0.05	0.04	0.06	0.18	0.25	0.33	0.38	0.60	0.70
13	0.05	0.10	0.16	0.44	0.55	0.71	0.78	0.87	0.98	0.97
14	0.08	0.13	0.26	0.32	0.48	0.67	0.76	0.86	0.93	0.98
15	0.11	0.15	0.18	0.35	0.49	0.70	0.81	0.89	0.95	0.98
16	0.07	0.19	0.26	0.37	0.61	0.82	0.88	0.98	0.99	1.00
17	0.04	0.11	0.31	0.46	0.68	0.78	0.90	0.99	0.98	1.00
18	0.06	0.10	0.21	0.38	0.63	0.76	0.93	0.94	0.98	0.99
19	0.04	0.08	0.12	0.09	0.18	0.31	0.30	0.52	0.66	0.70
20	0.07	0.07	0.08	0.17	0.20	0.34	0.51	0.61	0.59	0.67
21	0.04	0.12	0.12	0.19	0.29	0.37	0.48	0.66	0.82	0.82
22	0.02	0.05	0.02	0.05	0.14	0.12	0.30	0.40	0.47	0.72
23	0.04	0.04	0.11	0.09	0.12	0.27	0.26	0.42	0.51	0.68
24	0.05	0.04	0.10	0.11	0.18	0.29	0.37	0.54	0.57	0.75
25	0.05	0.12	0.16	0.36	0.45	0.66	0.81	0.92	0.98	0.99
26	0.08	0.08	0.23	0.32	0.56	0.65	0.83	0.86	0.96	0.95
27	0.11	0.18	0.25	0.38	0.54	0.69	0.79	0.87	0.94	0.92
28	0.04	0.12	0.11	0.28	0.36	0.54	0.75	0.81	0.94	0.98
29	0.06	0.13	0.14	0.35	0.40	0.60	0.70	0.84	0.93	0.97
30	0.08	0.16	0.19	0.39	0.45	0.62	0.66	0.81	0.89	0.89
31	0.06	0.17	0.24	0.43	0.70	0.85	0.95	0.99	0.97	1.00
32	0.05	0.12	0.29	0.47	0.58	0.84	0.87	0.98	0.98	1.00
33	0.05	0.12	0.23	0.47	0.67	0.77	0.91	0.93	0.99	1.00
34	0.04	0.13	0.35	0.47	0.75	0.82	0.86	0.95	0.98	1.00
35	0.05	0.10	0.27	0.49	0.55	0.81	0.90	0.98	0.99	1.00
36	0.05	0.16	0.26	0.52	0.75	0.81	0.96	1.00	0.99	1.00
37	0.07	0.17	0.26	0.56	0.66	0.77	0.93	0.98	0.99	0.98
38	0.08	0.11	0.34	0.47	0.66	0.84	0.95	0.99	1.00	1.00
39	0.06	0.15	0.39	0.43	0.67	0.87	0.92	0.99	0.99	1.00
40	0.09	0.20	0.35	0.53	0.62	0.84	0.92	0.99	0.99	1.00
41	0.11	0.15	0.30	0.45	0.68	0.86	0.94	1.00	0.99	1.00
42	0.06	0.13	0.26	0.42	0.68	0.87	0.96	0.98	1.00	1.00

Table 8: POD according to each scenario and each k2 value, k1 = 0, alpha = 0.01

	k2=1	k2=2	k2=3	k2=4	k2=5	k2=6	k2=7	k2=8	k2=9	k2=10
1	0.04	0.08	0.15	0.21	0.34	0.43	0.52	0.69	0.76	0.78
2	0.03	0.14	0.15	0.28	0.41	0.46	0.57	0.69	0.71	0.88
3	0.08	0.14	0.24	0.32	0.41	0.50	0.73	0.74	0.86	0.91
4	0.02	0.10	0.12	0.17	0.37	0.45	0.56	0.74	0.82	0.90
5	0.06	0.09	0.14	0.28	0.38	0.49	0.68	0.77	0.88	0.95
6	0.02	0.13	0.21	0.36	0.43	0.50	0.72	0.87	0.93	0.87
7	0.13	0.16	0.16	0.16	0.29	0.23	0.31	0.40	0.43	0.43
8	0.08	0.12	0.10	0.14	0.30	0.23	0.29	0.33	0.39	0.57
9	0.08	0.07	0.13	0.20	0.18	0.25	0.30	0.34	0.45	0.50
10	0.01	0.01	0.02	0.09	0.17	0.10	0.27	0.27	0.52	0.59
11	0.02	0.00	0.05	0.04	0.19	0.14	0.29	0.44	0.55	0.55
12	0.02	0.07	0.01	0.05	0.11	0.22	0.32	0.47	0.50	0.64
13	0.07	0.09	0.25	0.45	0.58	0.57	0.86	0.86	0.94	0.96
14	0.06	0.10	0.24	0.32	0.42	0.66	0.75	0.85	0.96	0.96
15	0.13	0.15	0.23	0.41	0.67	0.67	0.77	0.90	0.92	0.93
16	0.07	0.13	0.20	0.39	0.63	0.81	0.89	0.92	0.99	0.99
17	0.07	0.10	0.28	0.52	0.61	0.80	0.89	0.96	1.00	0.98
18	0.04	0.16	0.28	0.33	0.57	0.69	0.85	0.92	0.98	1.00
19	0.06	0.03	0.07	0.08	0.18	0.29	0.34	0.50	0.55	0.68
20	0.06	0.08	0.13	0.15	0.29	0.31	0.42	0.54	0.59	0.80
21	0.02	0.07	0.08	0.17	0.22	0.42	0.50	0.60	0.68	0.83
22	0.01	0.03	0.04	0.05	0.08	0.13	0.23	0.34	0.58	0.61
23	0.02	0.06	0.07	0.10	0.12	0.14	0.25	0.50	0.51	0.64
24	0.04	0.04	0.13	0.12	0.18	0.23	0.36	0.40	0.55	0.61
25	0.01	0.07	0.17	0.31	0.45	0.57	0.73	0.91	1.00	0.98
26	0.08	0.18	0.20	0.26	0.45	0.58	0.77	0.79	0.95	0.98
27	0.09	0.12	0.18	0.41	0.50	0.66	0.79	0.88	0.90	0.94
28	0.01	0.09	0.19	0.21	0.40	0.60	0.67	0.79	0.89	0.98
29	0.08	0.11	0.15	0.24	0.38	0.52	0.73	0.76	0.87	0.92
30	0.07	0.14	0.18	0.40	0.49	0.61	0.72	0.84	0.82	0.91
31	0.08	0.14	0.23	0.48	0.66	0.85	0.91	0.97	0.98	0.99
32	0.05	0.18	0.36	0.44	0.62	0.79	0.93	0.95	0.98	0.99
33	0.03	0.11	0.30	0.43	0.70	0.75	0.90	0.98	0.99	1.00
34	0.07	0.13	0.23	0.46	0.66	0.82	0.93	0.98	0.99	1.00
35	0.07	0.14	0.27	0.34	0.64	0.75	0.94	0.98	1.00	1.00
36	0.09	0.16	0.29	0.48	0.51	0.85	0.94	0.96	0.99	0.99
37	0.07	0.15	0.28	0.42	0.74	0.72	0.87	0.98	1.00	1.00
38	0.02	0.14	0.31	0.49	0.61	0.86	0.96	0.98	1.00	0.99
39	0.05	0.23	0.27	0.49	0.70	0.85	0.96	0.96	1.00	1.00
40	0.06	0.27	0.26	0.46	0.71	0.86	0.98	0.97	0.98	1.00
41	0.10	0.05	0.35	0.50	0.59	0.93	0.93	0.97	1.00	1.00
42	0.10	0.15	0.27	0.46	0.67	0.83	0.96	0.99	1.00	0.99

Table 9: POD according to each scenario and each k2 value, k1 = 2, alpha = 0.01

	k2=1	k2=2	k2=3	k2=4	k2=5	k2=6	k2=7	k2=8	k2=9	k2=10
1	0.05	0.08	0.10	0.23	0.32	0.39	0.58	0.68	0.77	0.81
2	0.07	0.13	0.15	0.27	0.38	0.46	0.59	0.71	0.77	0.80
3	0.11	0.15	0.22	0.32	0.50	0.53	0.73	0.82	0.83	0.92
4	0.02	0.05	0.14	0.18	0.28	0.43	0.57	0.74	0.89	0.87
5	0.05	0.07	0.15	0.25	0.37	0.45	0.63	0.81	0.87	0.92
6	0.12	0.16	0.20	0.31	0.38	0.58	0.74	0.79	0.86	0.95
7	0.07	0.06	0.12	0.13	0.20	0.22	0.31	0.38	0.39	0.51
8	0.08	0.08	0.09	0.16	0.13	0.25	0.28	0.27	0.33	0.46
9	0.06	0.14	0.09	0.13	0.18	0.33	0.24	0.39	0.40	0.54
10	0.01	0.00	0.02	0.06	0.07	0.13	0.28	0.35	0.48	0.56
11	0.01	0.03	0.02	0.06	0.14	0.17	0.17	0.37	0.43	0.56
12	0.01	0.03	0.04	0.08	0.18	0.20	0.19	0.35	0.48	0.53
13	0.04	0.11	0.24	0.37	0.53	0.63	0.80	0.87	0.91	0.98
14	0.09	0.11	0.27	0.32	0.54	0.64	0.76	0.83	0.91	0.93
15	0.05	0.14	0.30	0.34	0.45	0.66	0.71	0.88	0.93	0.94
16	0.02	0.10	0.29	0.50	0.50	0.66	0.82	0.91	0.98	0.98
17	0.05	0.13	0.34	0.40	0.57	0.69	0.81	0.91	0.97	1.00
18	0.00	0.11	0.23	0.48	0.53	0.79	0.89	0.94	0.99	0.99
19	0.01	0.08	0.09	0.08	0.20	0.23	0.24	0.47	0.53	0.69
20	0.04	0.10	0.09	0.12	0.22	0.24	0.33	0.52	0.59	0.72
21	0.03	0.06	0.13	0.10	0.19	0.35	0.46	0.65	0.74	0.75
22	0.00	0.06	0.02	0.05	0.08	0.16	0.18	0.29	0.41	0.56
23	0.03	0.02	0.06	0.07	0.20	0.17	0.36	0.37	0.47	0.64
24	0.01	0.02	0.07	0.19	0.13	0.23	0.34	0.35	0.53	0.67
25	0.03	0.09	0.16	0.26	0.40	0.58	0.81	0.88	0.91	0.99
26	0.08	0.15	0.19	0.25	0.51	0.56	0.62	0.85	0.84	0.97
27	0.07	0.09	0.29	0.41	0.50	0.61	0.81	0.90	0.86	0.94
28	0.01	0.04	0.17	0.16	0.29	0.56	0.74	0.88	0.95	0.97
29	0.05	0.08	0.16	0.31	0.44	0.57	0.60	0.76	0.83	0.94
30	0.06	0.17	0.20	0.29	0.42	0.60	0.70	0.80	0.90	0.88
31	0.06	0.14	0.24	0.39	0.60	0.88	0.89	0.98	0.98	1.00
32	0.06	0.15	0.26	0.43	0.65	0.78	0.91	0.97	0.99	1.00
33	0.10	0.12	0.25	0.46	0.55	0.89	0.94	0.96	1.00	0.99
34	0.06	0.15	0.26	0.36	0.62	0.81	0.88	0.95	0.99	1.00
35	0.06	0.17	0.23	0.40	0.69	0.73	0.90	0.95	0.99	1.00
36	0.04	0.10	0.26	0.38	0.64	0.82	0.94	0.99	0.97	1.00
37	0.04	0.13	0.34	0.43	0.70	0.86	0.95	0.97	0.98	1.00
38	0.06	0.07	0.21	0.51	0.63	0.87	0.93	0.94	1.00	1.00
39	0.09	0.17	0.32	0.47	0.64	0.79	0.91	0.97	1.00	1.00
40	0.08	0.10	0.27	0.44	0.65	0.89	0.89	0.99	0.99	1.00
41	0.05	0.10	0.30	0.50	0.56	0.83	0.93	0.97	1.00	1.00
42	0.07	0.23	0.29	0.42	0.74	0.86	0.96	0.98	0.99	0.99

Table 10: POD according to each scenario and each k2 value, k1 = 3, alpha = 0.01

	k2=1	k2=2	k2=3	k2=4	k2=5	k2=6	k2=7	k2=8	k2=9	k2=10
1	0.01	0.06	0.09	0.18	0.29	0.31	0.60	0.70	0.73	0.83
2	0.05	0.05	0.14	0.24	0.34	0.52	0.50	0.66	0.78	0.81
3	0.08	0.17	0.22	0.27	0.41	0.47	0.69	0.76	0.77	0.87
4	0.03	0.03	0.07	0.15	0.29	0.38	0.49	0.66	0.77	0.89
5	0.03	0.09	0.15	0.20	0.35	0.52	0.46	0.81	0.80	0.81
6	0.03	0.05	0.13	0.29	0.33	0.60	0.70	0.74	0.84	0.92
7	0.04	0.06	0.08	0.13	0.23	0.19	0.28	0.30	0.38	0.44
8	0.04	0.08	0.09	0.12	0.18	0.24	0.27	0.31	0.37	0.41
9	0.09	0.11	0.18	0.11	0.21	0.31	0.26	0.44	0.36	0.44
10	0.00	0.01	0.03	0.03	0.06	0.17	0.25	0.36	0.39	0.54
11	0.00	0.02	0.04	0.06	0.10	0.15	0.17	0.29	0.45	0.48
12	0.00	0.03	0.02	0.11	0.08	0.15	0.22	0.33	0.32	0.52
13	0.05	0.11	0.23	0.34	0.52	0.68	0.78	0.86	0.93	0.92
14	0.03	0.10	0.24	0.37	0.56	0.62	0.76	0.86	0.95	0.96
15	0.09	0.09	0.25	0.41	0.52	0.68	0.77	0.82	0.92	0.99
16	0.04	0.10	0.22	0.39	0.54	0.74	0.76	0.90	0.98	0.98
17	0.01	0.10	0.24	0.41	0.61	0.75	0.80	0.91	0.97	0.98
18	0.03	0.09	0.24	0.39	0.48	0.71	0.83	0.89	0.97	0.99
19	0.02	0.00	0.06	0.07	0.09	0.22	0.24	0.41	0.40	0.59
20	0.02	0.01	0.12	0.13	0.20	0.24	0.38	0.41	0.50	0.59
21	0.04	0.06	0.07	0.14	0.29	0.29	0.53	0.49	0.59	0.71
22	0.00	0.02	0.04	0.01	0.06	0.16	0.14	0.27	0.30	0.52
23	0.02	0.05	0.03	0.05	0.19	0.15	0.24	0.41	0.53	0.50
24	0.01	0.04	0.09	0.04	0.17	0.24	0.27	0.44	0.53	0.57
25	0.04	0.04	0.12	0.27	0.33	0.56	0.69	0.90	0.92	0.96
26	0.04	0.10	0.24	0.29	0.50	0.58	0.70	0.86	0.91	0.92
27	0.07	0.18	0.19	0.27	0.54	0.65	0.70	0.84	0.89	0.89
28	0.02	0.05	0.09	0.16	0.26	0.45	0.66	0.74	0.88	0.98
29	0.05	0.11	0.09	0.18	0.31	0.48	0.70	0.80	0.91	0.94
30	0.06	0.10	0.25	0.32	0.45	0.47	0.70	0.75	0.79	0.93
31	0.07	0.09	0.33	0.34	0.52	0.75	0.89	0.94	1.00	0.99
32	0.05	0.15	0.23	0.44	0.60	0.77	0.88	0.92	0.99	1.00
33	0.03	0.13	0.23	0.38	0.69	0.75	0.85	0.93	0.99	0.98
34	0.01	0.08	0.23	0.34	0.58	0.71	0.89	0.96	0.98	0.99
35	0.04	0.08	0.21	0.32	0.62	0.83	0.89	0.94	0.99	1.00
36	0.04	0.08	0.19	0.42	0.61	0.70	0.85	0.95	0.97	1.00
37	0.06	0.11	0.26	0.45	0.54	0.84	0.91	0.97	0.96	0.99
38	0.03	0.10	0.22	0.36	0.69	0.82	0.96	0.97	1.00	1.00
39	0.08	0.11	0.27	0.32	0.62	0.81	0.89	0.97	1.00	1.00
40	0.06	0.15	0.27	0.35	0.58	0.78	0.91	0.95	0.99	1.00
41	0.06	0.11	0.33	0.42	0.51	0.76	0.91	0.96	0.99	1.00
42	0.03	0.10	0.22	0.49	0.59	0.81	0.91	0.98	0.99	1.00

Table 11: POD according to each scenario and each k2 value, k1 = 5, alpha = 0.01

	k2=1	k2=2	k2=3	k2=4	k2=5	k2=6	k2=7	k2=8	k2=9	k2=10
1	0.04	0.03	0.10	0.21	0.18	0.24	0.41	0.42	0.57	0.73
2	0.02	0.06	0.13	0.11	0.32	0.35	0.46	0.59	0.71	0.77
3	0.07	0.07	0.14	0.29	0.28	0.36	0.44	0.61	0.74	0.87
4	0.00	0.01	0.06	0.09	0.17	0.26	0.37	0.48	0.64	0.76
5	0.03	0.05	0.07	0.13	0.17	0.33	0.38	0.47	0.68	0.77
6	0.03	0.04	0.16	0.16	0.26	0.49	0.48	0.66	0.68	0.86
7	0.04	0.04	0.08	0.08	0.15	0.11	0.20	0.18	0.31	0.33
8	0.04	0.03	0.03	0.07	0.15	0.14	0.18	0.16	0.30	0.33
9	0.05	0.04	0.07	0.13	0.16	0.20	0.10	0.30	0.27	0.33
10	0.00	0.00	0.00	0.01	0.02	0.05	0.08	0.19	0.30	0.26
11	0.00	0.00	0.00	0.04	0.03	0.06	0.13	0.13	0.27	0.34
12	0.01	0.01	0.02	0.04	0.08	0.09	0.14	0.22	0.27	0.38
13	0.03	0.01	0.15	0.23	0.35	0.55	0.64	0.75	0.88	0.92
14	0.00	0.04	0.11	0.16	0.43	0.48	0.63	0.80	0.86	0.89
15	0.01	0.04	0.11	0.24	0.29	0.47	0.66	0.74	0.90	0.89
16	0.01	0.01	0.06	0.15	0.27	0.40	0.65	0.76	0.94	0.97
17	0.02	0.02	0.08	0.19	0.39	0.53	0.74	0.77	0.89	0.94
18	0.02	0.04	0.06	0.17	0.34	0.49	0.61	0.80	0.84	0.92
19	0.01	0.03	0.01	0.00	0.10	0.18	0.17	0.28	0.39	0.43
20	0.02	0.02	0.04	0.04	0.10	0.16	0.17	0.36	0.43	0.49
21	0.04	0.02	0.05	0.12	0.15	0.23	0.33	0.45	0.54	0.59
22	0.02	0.01	0.00	0.04	0.03	0.05	0.11	0.11	0.25	0.25
23	0.00	0.01	0.04	0.06	0.06	0.08	0.11	0.27	0.37	0.48
24	0.01	0.01	0.04	0.08	0.09	0.13	0.24	0.25	0.39	0.38
25	0.01	0.09	0.09	0.17	0.27	0.39	0.56	0.75	0.90	0.96
26	0.07	0.12	0.14	0.30	0.30	0.46	0.58	0.79	0.79	0.86
27	0.07	0.08	0.10	0.29	0.30	0.55	0.64	0.75	0.78	0.77
28	0.02	0.02	0.03	0.06	0.25	0.39	0.41	0.64	0.80	0.88
29	0.02	0.04	0.11	0.18	0.34	0.41	0.63	0.66	0.75	0.88
30	0.09	0.07	0.07	0.21	0.26	0.39	0.55	0.69	0.76	0.86
31	0.05	0.07	0.22	0.28	0.47	0.64	0.82	0.95	0.98	1.00
32	0.05	0.08	0.15	0.20	0.42	0.65	0.83	0.95	1.00	1.00
33	0.01	0.04	0.09	0.19	0.43	0.68	0.76	0.88	0.96	1.00
34	0.00	0.04	0.09	0.22	0.48	0.60	0.76	0.86	0.98	0.96
35	0.01	0.07	0.11	0.18	0.38	0.62	0.73	0.90	0.98	0.97
36	0.03	0.03	0.18	0.25	0.53	0.54	0.74	0.92	0.96	0.99
37	0.01	0.04	0.15	0.27	0.49	0.71	0.87	0.95	0.96	1.00
38	0.01	0.07	0.21	0.35	0.47	0.63	0.86	0.94	1.00	0.99
39	0.02	0.06	0.14	0.30	0.44	0.71	0.80	0.95	1.00	1.00
40	0.02	0.05	0.11	0.33	0.47	0.57	0.78	0.86	0.97	1.00
41	0.03	0.07	0.11	0.26	0.58	0.53	0.86	0.94	0.96	1.00
42	0.02	0.08	0.12	0.35	0.46	0.72	0.81	0.95	0.99	1.00

Table 12: POD according to each scenario and each k2 value, k1 = 10, alpha = 0.01

Overall performances of Periodic Negative Binomial GLM algorithm ($\alpha = 0.05$)

	FPR k1=0	FPR k1=2	FPR k1=3	FPR k1=5	FPR k1=10
1	0.03	0.03	0.03	0.03	0.02
2	0.04	0.03	0.03	0.03	0.02
3	0.04	0.04	0.04	0.03	0.02
4	0.03	0.03	0.02	0.02	0.01
5	0.04	0.03	0.03	0.03	0.02
6	0.04	0.03	0.03	0.03	0.02
7	0.03	0.02	0.02	0.02	0.02
8	0.03	0.02	0.02	0.02	0.02
9	0.03	0.02	0.02	0.02	0.02
10	0.02	0.02	0.02	0.01	0.01
11	0.02	0.02	0.02	0.02	0.01
12	0.03	0.02	0.02	0.02	0.01
13	0.04	0.03	0.03	0.03	0.02
14	0.03	0.03	0.03	0.03	0.02
15	0.04	0.03	0.03	0.03	0.02
16	0.04	0.04	0.03	0.03	0.01
17	0.04	0.04	0.04	0.03	0.01
18	0.04	0.04	0.04	0.03	0.02
19	0.05	0.05	0.04	0.04	0.03
20	0.05	0.05	0.05	0.04	0.03
21	0.05	0.04	0.04	0.04	0.03
22	0.03	0.02	0.02	0.02	0.01
23	0.04	0.03	0.03	0.03	0.02
24	0.04	0.03	0.03	0.03	0.02
25	0.04	0.04	0.04	0.03	0.03
26	0.05	0.05	0.05	0.04	0.03
27	0.06	0.06	0.05	0.05	0.04
28	0.04	0.03	0.03	0.03	0.02
29	0.04	0.04	0.04	0.04	0.03
30	0.06	0.05	0.05	0.04	0.03
31	0.05	0.04	0.04	0.04	0.02
32	0.05	0.04	0.04	0.04	0.02
33	0.04	0.04	0.04	0.03	0.02
34	0.05	0.04	0.04	0.03	0.02
35	0.04	0.04	0.04	0.03	0.02
36	0.05	0.05	0.04	0.04	0.02
37	0.05	0.04	0.04	0.04	0.02
38	0.04	0.04	0.04	0.03	0.02
39	0.05	0.05	0.05	0.04	0.02
40	0.05	0.05	0.05	0.04	0.03
41	0.05	0.05	0.04	0.04	0.02
42	0.05	0.04	0.04	0.03	0.02

Table 13: FPR according to each scenario and each k1 value, alpha = 0.05

	k2=1	k2=2	k2=3	k2=4	k2=5	k2=6	k2=7	k2=8	k2=9	k2=10
1	0.26	0.32	0.43	0.45	0.71	0.79	0.88	0.93	0.96	0.96
2	0.23	0.30	0.41	0.76	0.75	0.78	0.91	0.96	0.95	0.99
3	0.33	0.39	0.50	0.68	0.71	0.76	0.93	0.94	0.97	0.95
4	0.23	0.26	0.38	0.63	0.71	0.83	0.92	0.99	0.95	0.99
5	0.16	0.26	0.46	0.70	0.75	0.84	0.90	0.97	0.99	1.00
6	0.29	0.32	0.49	0.65	0.78	0.89	0.93	0.95	1.00	0.99
7	0.63	0.68	0.67	0.71	0.83	0.82	0.89	0.90	0.89	0.93
8	0.63	0.60	0.64	0.85	0.74	0.83	0.83	0.84	0.91	0.92
9	0.56	0.71	0.59	0.71	0.73	0.81	0.85	0.88	0.91	0.91
10	0.12	0.16	0.23	0.42	0.60	0.68	0.81	0.87	0.92	0.94
11	0.09	0.16	0.37	0.47	0.65	0.72	0.81	0.89	0.92	0.99
12	0.11	0.17	0.32	0.47	0.58	0.78	0.81	0.89	0.91	0.94
13	0.20	0.34	0.51	0.72	0.84	0.90	0.92	0.97	1.00	1.00
14	0.27	0.34	0.53	0.65	0.71	0.89	0.92	0.98	0.98	1.00
15	0.26	0.46	0.45	0.63	0.76	0.88	0.97	0.98	1.00	1.00
16	0.24	0.44	0.58	0.70	0.83	0.98	0.97	1.00	1.00	1.00
17	0.20	0.33	0.54	0.81	0.90	0.95	0.99	1.00	0.99	1.00
18	0.24	0.38	0.58	0.75	0.92	0.93	0.99	0.99	1.00	1.00
19	0.19	0.23	0.41	0.53	0.72	0.86	0.88	0.96	0.99	0.99
20	0.20	0.26	0.40	0.55	0.73	0.80	0.92	0.95	0.98	1.00
21	0.18	0.35	0.39	0.57	0.71	0.79	0.93	0.95	0.99	0.99
22	0.13	0.17	0.25	0.32	0.53	0.72	0.88	0.93	0.98	1.00
23	0.15	0.21	0.34	0.34	0.60	0.72	0.83	0.92	0.95	0.98
24	0.11	0.29	0.35	0.50	0.56	0.72	0.82	0.92	0.93	0.98
25	0.21	0.31	0.54	0.71	0.82	0.93	1.00	1.00	1.00	1.00
26	0.21	0.33	0.59	0.65	0.82	0.94	0.94	1.00	1.00	1.00
27	0.23	0.41	0.48	0.69	0.79	0.88	0.94	0.98	0.99	1.00
28	0.10	0.33	0.43	0.67	0.80	0.91	0.98	0.99	0.98	1.00
29	0.25	0.34	0.41	0.65	0.82	0.84	0.93	0.98	1.00	1.00
30	0.18	0.45	0.48	0.71	0.76	0.87	0.87	0.97	0.96	0.96
31	0.21	0.43	0.52	0.74	0.89	1.00	0.99	1.00	1.00	1.00
32	0.22	0.43	0.65	0.77	0.89	0.99	0.99	1.00	1.00	1.00
33	0.21	0.35	0.59	0.80	0.92	0.96	0.98	1.00	1.00	1.00
34	0.22	0.46	0.57	0.77	0.94	1.00	1.00	1.00	1.00	1.00
35	0.28	0.42	0.61	0.80	0.89	0.99	0.98	1.00	1.00	1.00
36	0.18	0.38	0.60	0.74	0.93	0.97	0.99	1.00	1.00	1.00
37	0.21	0.46	0.63	0.79	0.90	0.95	0.99	1.00	1.00	1.00
38	0.26	0.36	0.64	0.79	0.89	0.94	1.00	1.00	1.00	1.00
39	0.25	0.48	0.68	0.85	0.97	1.00	1.00	1.00	1.00	1.00
40	0.26	0.52	0.64	0.91	0.87	0.97	0.99	1.00	1.00	1.00
41	0.32	0.42	0.55	0.80	0.95	0.96	0.98	1.00	1.00	1.00
42	0.26	0.45	0.62	0.76	0.89	0.96	1.00	1.00	1.00	1.00

Table 14: POD according to each scenario and each k2 value, k1 = 0, alpha = 0.05

	k2=1	k2=2	k2=3	k2=4	k2=5	k2=6	k2=7	k2=8	k2=9	k2=10
1	0.18	0.26	0.49	0.44	0.71	0.71	0.85	0.92	0.89	0.94
2	0.25	0.40	0.38	0.56	0.77	0.85	0.87	0.90	0.91	0.98
3	0.24	0.32	0.51	0.64	0.68	0.86	0.92	0.91	0.95	0.99
4	0.14	0.23	0.38	0.55	0.72	0.73	0.89	0.97	0.99	0.99
5	0.23	0.32	0.47	0.59	0.77	0.83	0.93	0.96	0.99	0.98
6	0.13	0.34	0.51	0.64	0.76	0.86	0.93	0.99	0.99	0.99
7	0.50	0.55	0.64	0.72	0.73	0.77	0.76	0.82	0.90	0.89
8	0.56	0.50	0.56	0.64	0.62	0.67	0.78	0.85	0.86	0.93
9	0.46	0.50	0.58	0.59	0.72	0.80	0.85	0.80	0.87	0.95
10	0.07	0.16	0.23	0.35	0.50	0.71	0.78	0.84	0.94	0.97
11	0.05	0.11	0.29	0.34	0.63	0.61	0.76	0.89	0.93	0.97
12	0.09	0.22	0.22	0.38	0.60	0.68	0.88	0.84	0.85	0.98
13	0.23	0.30	0.50	0.70	0.83	0.88	0.98	0.97	0.99	1.00
14	0.17	0.35	0.56	0.65	0.76	0.86	0.95	0.98	1.00	0.99
15	0.26	0.36	0.45	0.69	0.88	0.91	0.96	0.99	0.97	1.00
16	0.24	0.34	0.50	0.66	0.91	0.95	0.98	1.00	1.00	1.00
17	0.17	0.37	0.57	0.80	0.88	0.95	0.99	0.99	1.00	1.00
18	0.22	0.39	0.58	0.69	0.82	0.92	0.97	0.99	1.00	1.00
19	0.19	0.26	0.35	0.57	0.72	0.82	0.89	0.97	0.98	0.98
20	0.16	0.22	0.41	0.61	0.63	0.86	0.91	0.94	0.97	1.00
21	0.13	0.25	0.38	0.55	0.68	0.77	0.89	0.96	0.99	0.99
22	0.07	0.15	0.25	0.43	0.55	0.69	0.84	0.93	0.98	0.99
23	0.07	0.20	0.24	0.44	0.60	0.76	0.82	0.91	0.93	0.95
24	0.21	0.20	0.37	0.50	0.61	0.69	0.84	0.90	0.93	0.94
25	0.10	0.28	0.40	0.76	0.88	0.88	0.95	0.99	1.00	1.00
26	0.20	0.40	0.59	0.57	0.79	0.94	0.99	0.98	1.00	1.00
27	0.23	0.33	0.44	0.73	0.78	0.87	0.94	0.98	0.97	1.00
28	0.14	0.33	0.38	0.57	0.83	0.90	0.97	1.00	1.00	1.00
29	0.16	0.31	0.38	0.59	0.77	0.88	0.98	0.95	1.00	1.00
30	0.14	0.35	0.42	0.70	0.74	0.87	0.89	0.92	0.96	0.98
31	0.29	0.36	0.56	0.79	0.91	0.98	0.98	1.00	1.00	1.00
32	0.22	0.40	0.63	0.79	0.89	0.97	0.98	0.99	0.99	0.99
33	0.15	0.45	0.55	0.78	0.91	0.94	0.99	1.00	1.00	1.00
34	0.26	0.44	0.58	0.80	0.88	0.94	0.98	1.00	1.00	1.00
35	0.24	0.40	0.50	0.68	0.88	0.97	0.99	1.00	1.00	1.00
36	0.23	0.31	0.68	0.74	0.90	0.94	1.00	1.00	1.00	1.00
37	0.25	0.37	0.63	0.77	0.95	0.95	0.98	1.00	1.00	1.00
38	0.11	0.38	0.61	0.80	0.92	0.99	0.99	1.00	1.00	1.00
39	0.15	0.46	0.59	0.86	0.93	0.97	1.00	1.00	1.00	1.00
40	0.26	0.57	0.58	0.84	0.98	0.98	0.99	0.99	1.00	1.00
41	0.26	0.28	0.65	0.79	0.87	0.99	0.99	0.99	1.00	1.00
42	0.27	0.39	0.63	0.76	0.86	0.99	0.99	1.00	1.00	1.00

Table 15: POD according to each scenario and each k2 value, k1 = 2, alpha = 0.05

	k2=1	k2=2	k2=3	k2=4	k2=5	k2=6	k2=7	k2=8	k2=9	k2=10
1	0.21	0.25	0.36	0.55	0.72	0.72	0.85	0.96	0.91	0.93
2	0.25	0.43	0.46	0.52	0.72	0.83	0.95	0.92	0.97	0.94
3	0.27	0.34	0.48	0.59	0.76	0.84	0.88	0.92	0.96	0.99
4	0.16	0.28	0.36	0.52	0.66	0.86	0.93	0.93	0.97	0.99
5	0.16	0.27	0.41	0.58	0.65	0.81	0.89	0.97	0.98	1.00
6	0.21	0.35	0.48	0.62	0.69	0.85	0.94	0.95	1.00	0.99
7	0.42	0.45	0.61	0.56	0.75	0.75	0.87	0.87	0.86	0.86
8	0.47	0.56	0.61	0.64	0.64	0.79	0.77	0.76	0.80	0.87
9	0.56	0.59	0.57	0.68	0.69	0.72	0.69	0.85	0.90	0.87
10	0.05	0.14	0.23	0.37	0.47	0.73	0.80	0.79	0.91	0.96
11	0.06	0.14	0.25	0.42	0.59	0.54	0.79	0.89	0.90	0.98
12	0.10	0.19	0.29	0.46	0.58	0.65	0.72	0.84	0.88	0.91
13	0.20	0.36	0.46	0.62	0.78	0.87	0.94	0.94	0.99	1.00
14	0.25	0.29	0.53	0.68	0.82	0.86	0.94	0.98	0.98	0.99
15	0.19	0.30	0.56	0.62	0.71	0.89	0.94	0.98	0.99	0.99
16	0.12	0.29	0.59	0.79	0.77	0.92	0.96	0.96	1.00	1.00
17	0.19	0.47	0.66	0.72	0.88	0.93	0.97	0.98	0.99	1.00
18	0.13	0.36	0.53	0.74	0.86	0.98	0.99	1.00	1.00	1.00
19	0.14	0.28	0.34	0.47	0.67	0.78	0.88	0.93	0.98	1.00
20	0.14	0.29	0.35	0.47	0.67	0.80	0.89	0.99	0.96	1.00
21	0.15	0.29	0.40	0.55	0.74	0.84	0.83	0.94	0.98	0.99
22	0.09	0.19	0.23	0.35	0.40	0.65	0.81	0.89	0.97	0.97
23	0.11	0.14	0.24	0.39	0.57	0.61	0.82	0.91	0.91	0.96
24	0.07	0.13	0.23	0.48	0.60	0.67	0.80	0.87	0.92	0.94
25	0.13	0.28	0.45	0.68	0.86	0.93	0.98	0.99	0.99	1.00
26	0.24	0.26	0.49	0.59	0.87	0.93	0.90	0.99	0.99	1.00
27	0.21	0.34	0.54	0.72	0.81	0.88	0.96	1.00	0.99	1.00
28	0.14	0.25	0.42	0.51	0.75	0.93	0.97	0.99	1.00	1.00
29	0.19	0.25	0.49	0.66	0.80	0.81	0.92	0.98	1.00	1.00
30	0.15	0.38	0.45	0.59	0.71	0.83	0.94	0.97	0.99	1.00
31	0.23	0.41	0.55	0.70	0.86	1.00	0.97	1.00	1.00	1.00
32	0.19	0.41	0.55	0.71	0.83	0.94	0.98	1.00	1.00	1.00
33	0.26	0.39	0.55	0.76	0.87	0.93	0.99	1.00	1.00	1.00
34	0.18	0.37	0.54	0.75	0.88	0.98	0.98	1.00	1.00	1.00
35	0.22	0.36	0.61	0.72	0.91	0.95	1.00	1.00	1.00	1.00
36	0.20	0.40	0.62	0.76	0.91	0.96	1.00	0.99	0.99	1.00
37	0.25	0.44	0.71	0.74	0.83	0.96	0.99	0.99	1.00	1.00
38	0.19	0.37	0.54	0.78	0.88	0.95	0.98	0.98	1.00	1.00
39	0.22	0.39	0.67	0.80	0.90	0.96	0.99	1.00	1.00	1.00
40	0.30	0.53	0.57	0.81	0.91	0.98	0.99	0.99	1.00	1.00
41	0.20	0.32	0.54	0.77	0.87	0.96	0.98	1.00	1.00	1.00
42	0.21	0.51	0.57	0.77	0.93	0.99	0.99	1.00	1.00	1.00

Table 16: POD according to each scenario and each k2 value, k1 = 3, alpha = 0.05

	k2=1	k2=2	k2=3	k2=4	k2=5	k2=6	k2=7	k2=8	k2=9	k2=10
1	0.17	0.27	0.42	0.52	0.60	0.67	0.90	0.96	0.94	0.96
2	0.21	0.23	0.47	0.57	0.65	0.83	0.84	0.94	0.95	0.98
3	0.29	0.34	0.49	0.65	0.73	0.74	0.86	0.90	0.94	0.98
4	0.17	0.23	0.31	0.43	0.61	0.73	0.84	0.96	0.99	1.00
5	0.14	0.23	0.47	0.49	0.72	0.79	0.92	0.96	0.97	0.98
6	0.11	0.24	0.43	0.65	0.68	0.86	0.90	0.97	0.97	1.00
7	0.44	0.56	0.51	0.71	0.73	0.62	0.75	0.84	0.88	0.94
8	0.54	0.50	0.59	0.62	0.60	0.64	0.70	0.86	0.86	0.84
9	0.50	0.36	0.62	0.50	0.62	0.77	0.75	0.82	0.87	0.85
10	0.05	0.12	0.24	0.30	0.42	0.55	0.72	0.79	0.88	0.92
11	0.06	0.10	0.26	0.40	0.51	0.57	0.70	0.87	0.93	0.95
12	0.08	0.18	0.21	0.40	0.49	0.67	0.76	0.91	0.88	0.93
13	0.19	0.35	0.48	0.67	0.78	0.87	0.91	0.98	1.00	0.99
14	0.18	0.34	0.48	0.64	0.77	0.88	0.94	0.97	1.00	0.99
15	0.20	0.30	0.57	0.65	0.76	0.89	0.94	0.96	0.97	1.00
16	0.20	0.30	0.46	0.73	0.89	0.93	0.94	0.98	0.99	0.99
17	0.13	0.28	0.52	0.62	0.88	0.95	0.97	0.99	1.00	1.00
18	0.16	0.31	0.46	0.69	0.80	0.87	0.97	0.96	1.00	1.00
19	0.10	0.27	0.27	0.48	0.57	0.80	0.83	0.94	0.96	0.98
20	0.11	0.16	0.37	0.57	0.63	0.75	0.92	0.94	0.99	0.99
21	0.17	0.25	0.32	0.48	0.72	0.78	0.92	0.91	0.93	0.96
22	0.06	0.12	0.21	0.26	0.44	0.59	0.81	0.86	0.96	0.96
23	0.09	0.11	0.26	0.44	0.52	0.66	0.79	0.90	0.93	0.97
24	0.13	0.19	0.29	0.34	0.50	0.64	0.77	0.88	0.88	0.94
25	0.13	0.30	0.43	0.65	0.81	0.88	0.94	1.00	0.99	1.00
26	0.12	0.36	0.48	0.58	0.82	0.89	0.94	0.97	1.00	0.99
27	0.27	0.41	0.46	0.61	0.78	0.84	0.90	0.95	0.98	0.95
28	0.10	0.31	0.32	0.51	0.64	0.82	0.96	0.98	0.98	1.00
29	0.21	0.30	0.39	0.50	0.63	0.83	0.96	0.99	1.00	1.00
30	0.15	0.35	0.55	0.70	0.74	0.80	0.89	0.94	0.96	0.97
31	0.20	0.39	0.56	0.65	0.84	0.92	0.99	0.99	1.00	1.00
32	0.22	0.41	0.59	0.69	0.84	0.91	0.96	0.99	1.00	1.00
33	0.13	0.33	0.53	0.70	0.91	0.94	0.97	0.99	1.00	1.00
34	0.15	0.32	0.56	0.64	0.95	0.94	0.98	0.99	1.00	1.00
35	0.18	0.39	0.56	0.65	0.89	0.98	0.98	1.00	1.00	1.00
36	0.14	0.41	0.53	0.72	0.86	0.94	0.96	0.99	1.00	1.00
37	0.20	0.29	0.62	0.71	0.91	0.97	0.99	1.00	0.99	1.00
38	0.15	0.38	0.50	0.74	0.94	0.98	1.00	0.99	1.00	1.00
39	0.19	0.23	0.67	0.75	0.86	0.97	0.99	1.00	1.00	1.00
40	0.23	0.39	0.63	0.71	0.84	0.94	0.99	0.99	1.00	1.00
41	0.20	0.29	0.59	0.75	0.86	0.95	0.99	1.00	0.99	1.00
42	0.20	0.30	0.54	0.83	0.92	0.98	1.00	1.00	1.00	1.00

Table 17: POD according to each scenario and each k2 value, k1 = 5, alpha = 0.05

	k2=1	k2=2	k2=3	k2=4	k2=5	k2=6	k2=7	k2=8	k2=9	k2=10
1	0.21	0.21	0.25	0.52	0.48	0.66	0.84	0.81	0.86	0.91
2	0.18	0.26	0.37	0.45	0.65	0.77	0.84	0.88	0.91	0.96
3	0.25	0.21	0.35	0.58	0.61	0.69	0.77	0.90	0.98	0.98
4	0.04	0.12	0.23	0.36	0.47	0.75	0.77	0.89	0.95	0.98
5	0.07	0.18	0.29	0.34	0.51	0.76	0.81	0.87	0.95	0.97
6	0.08	0.16	0.40	0.45	0.56	0.72	0.85	0.95	0.93	0.98
7	0.34	0.33	0.50	0.57	0.60	0.63	0.66	0.68	0.81	0.84
8	0.28	0.41	0.42	0.55	0.67	0.60	0.74	0.67	0.83	0.87
9	0.40	0.36	0.50	0.54	0.57	0.65	0.72	0.82	0.78	0.84
10	0.07	0.03	0.08	0.12	0.28	0.47	0.45	0.69	0.76	0.86
11	0.03	0.04	0.10	0.22	0.33	0.44	0.60	0.69	0.79	0.87
12	0.05	0.07	0.15	0.22	0.43	0.44	0.63	0.74	0.86	0.91
13	0.11	0.23	0.36	0.54	0.67	0.83	0.86	0.90	0.98	1.00
14	0.12	0.17	0.42	0.52	0.76	0.78	0.89	0.97	0.97	0.98
15	0.11	0.19	0.39	0.50	0.68	0.80	0.88	0.96	0.97	0.99
16	0.06	0.23	0.31	0.49	0.73	0.80	0.91	0.97	0.99	1.00
17	0.12	0.19	0.29	0.51	0.69	0.87	0.93	0.95	1.00	0.99
18	0.10	0.22	0.22	0.49	0.68	0.82	0.88	0.97	0.97	0.98
19	0.07	0.21	0.14	0.34	0.52	0.74	0.70	0.82	0.90	0.95
20	0.10	0.11	0.17	0.35	0.45	0.65	0.75	0.86	0.93	0.95
21	0.13	0.21	0.24	0.45	0.55	0.68	0.81	0.87	0.88	0.94
22	0.07	0.11	0.13	0.24	0.29	0.56	0.68	0.77	0.87	0.90
23	0.08	0.06	0.20	0.32	0.36	0.48	0.62	0.78	0.90	0.94
24	0.07	0.14	0.25	0.29	0.48	0.54	0.68	0.79	0.90	0.92
25	0.09	0.19	0.36	0.56	0.76	0.81	0.92	1.00	0.98	1.00
26	0.17	0.29	0.39	0.57	0.64	0.85	0.88	0.98	0.99	1.00
27	0.19	0.24	0.31	0.55	0.62	0.74	0.85	0.92	0.93	0.97
28	0.08	0.15	0.24	0.41	0.69	0.77	0.94	0.98	0.98	1.00
29	0.12	0.19	0.36	0.55	0.64	0.81	0.94	0.92	0.96	0.99
30	0.25	0.25	0.33	0.60	0.64	0.72	0.84	0.91	0.90	0.93
31	0.17	0.26	0.49	0.59	0.81	0.92	0.94	0.99	1.00	1.00
32	0.12	0.30	0.44	0.56	0.74	0.91	0.97	0.99	1.00	1.00
33	0.18	0.23	0.28	0.60	0.79	0.91	0.96	0.98	1.00	1.00
34	0.11	0.20	0.34	0.55	0.81	0.87	0.95	0.99	1.00	1.00
35	0.12	0.26	0.39	0.46	0.67	0.89	0.94	1.00	1.00	1.00
36	0.11	0.19	0.43	0.59	0.81	0.87	0.96	1.00	1.00	1.00
37	0.11	0.25	0.41	0.64	0.83	0.89	0.97	0.99	0.99	1.00
38	0.13	0.27	0.46	0.66	0.78	0.91	0.99	0.99	1.00	1.00
39	0.12	0.29	0.42	0.63	0.81	0.89	0.98	1.00	1.00	1.00
40	0.13	0.24	0.44	0.72	0.85	0.92	0.98	0.98	1.00	1.00
41	0.16	0.27	0.40	0.63	0.88	0.92	0.98	0.99	0.99	1.00
42	0.15	0.29	0.41	0.74	0.85	0.95	0.97	1.00	1.00	1.00

Table 18: POD according to each scenario and each k2 value, k1 = 10, alpha = 0.05
