# Supplementary material for: Evaluation and comparison of statistical methods for early temporal detection of outbreaks: A simulation-based study
Source: PLoS One. 2017 Jul 17;12(7):e0181227. doi: 10.1371/journal.pone.0181227 (PMC5513450; doi:10.1371/journal.pone.0181227)
Supplement: S6 Appendix — (PDF) [file pone.0181227.s006.pdf]

Evaluation and Comparison of Statistical Methods for Early Temporal Detection of Outbreaks: a Simulation-Based Study

Appendix S6: Overall performances of CDC algorithm ($\alpha = 0.001, 0.01$ and 0.05)

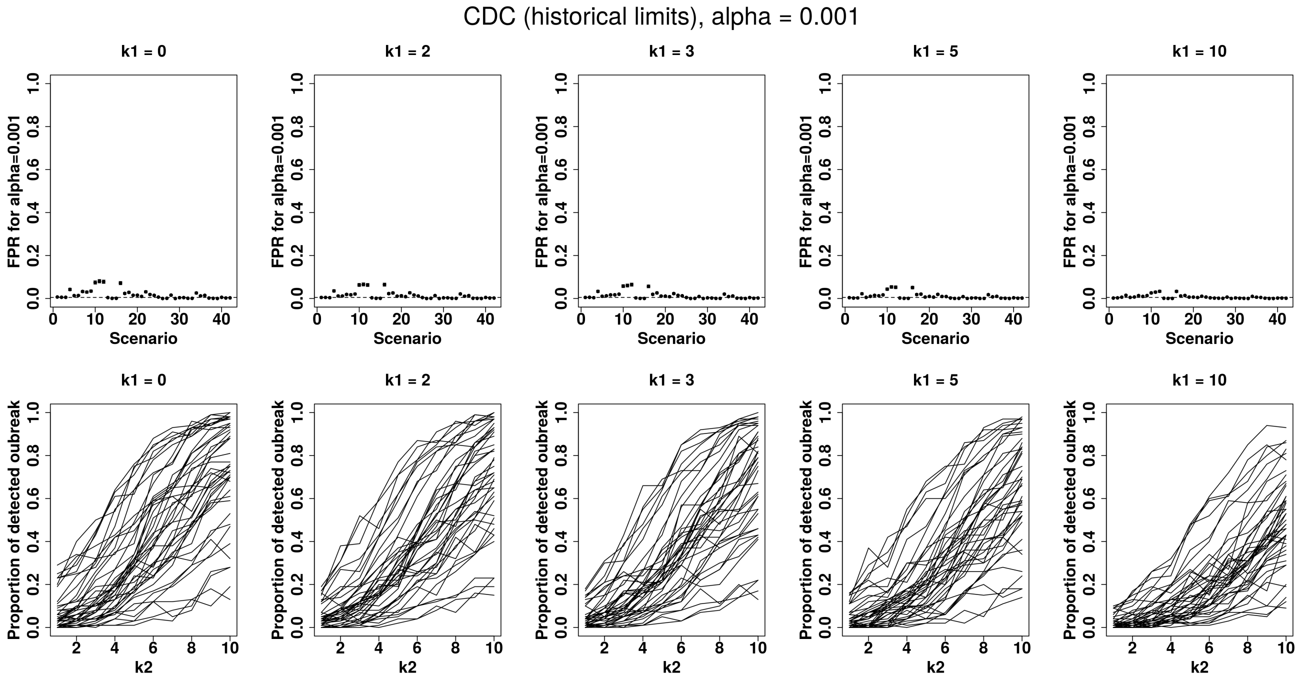

Figure 1: CDC algorithm performances for $\alpha = 0.001$ by increasing past outbreak amplitude $k_1 = 0$, 2, 3, 5 or 10 with (i) on the first row: false positive rate for 42 simulated scenarios, (ii) on the second row: probability of detection for 42 simulated scenarios (each curve corresponding to a scenario) by increasing current outbreak amplitude $k_2 = 1$ to 10.

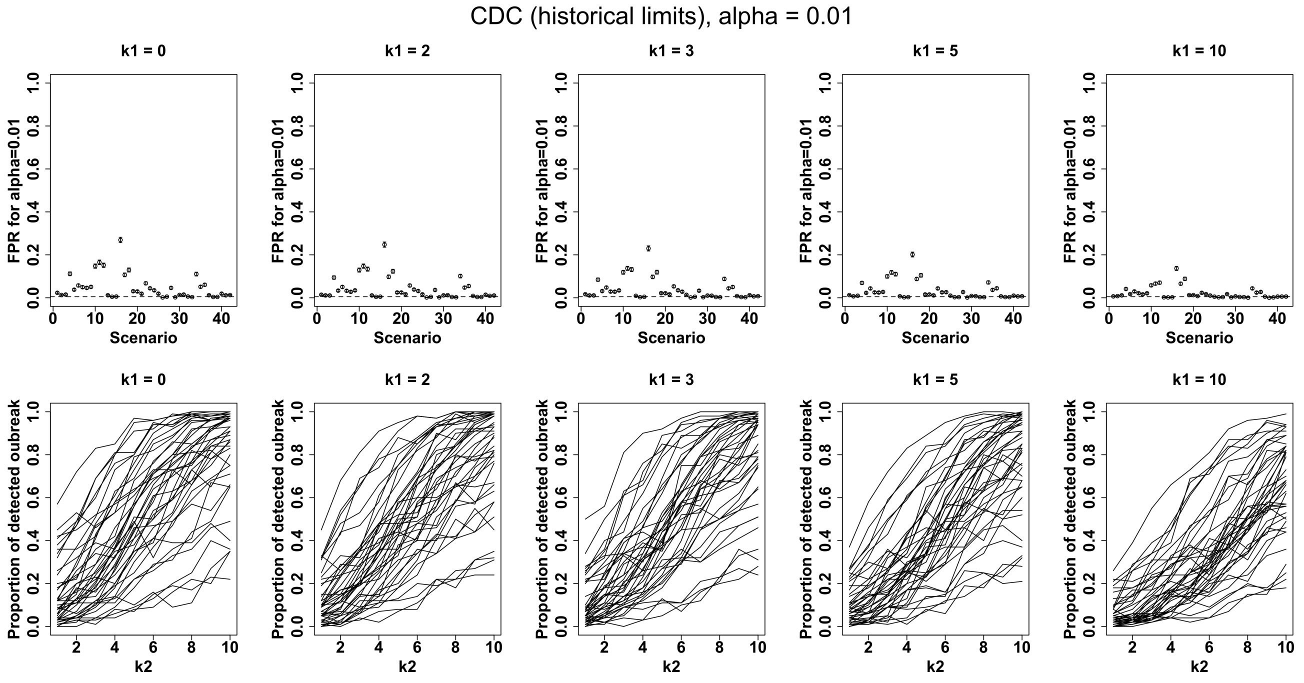

Figure 2: CDC algorithm performances for $\alpha = 0.01$ by increasing past outbreak amplitude $k_1 = 0$, 2, 3, 5 or 10 with (i) on the first row: false positive rate for 42 simulated scenarios, (ii) on the second row: probability of detection for 42 simulated scenarios (each curve corresponding to a scenario) by increasing current outbreak amplitude $k_2 = 1$ to 10.

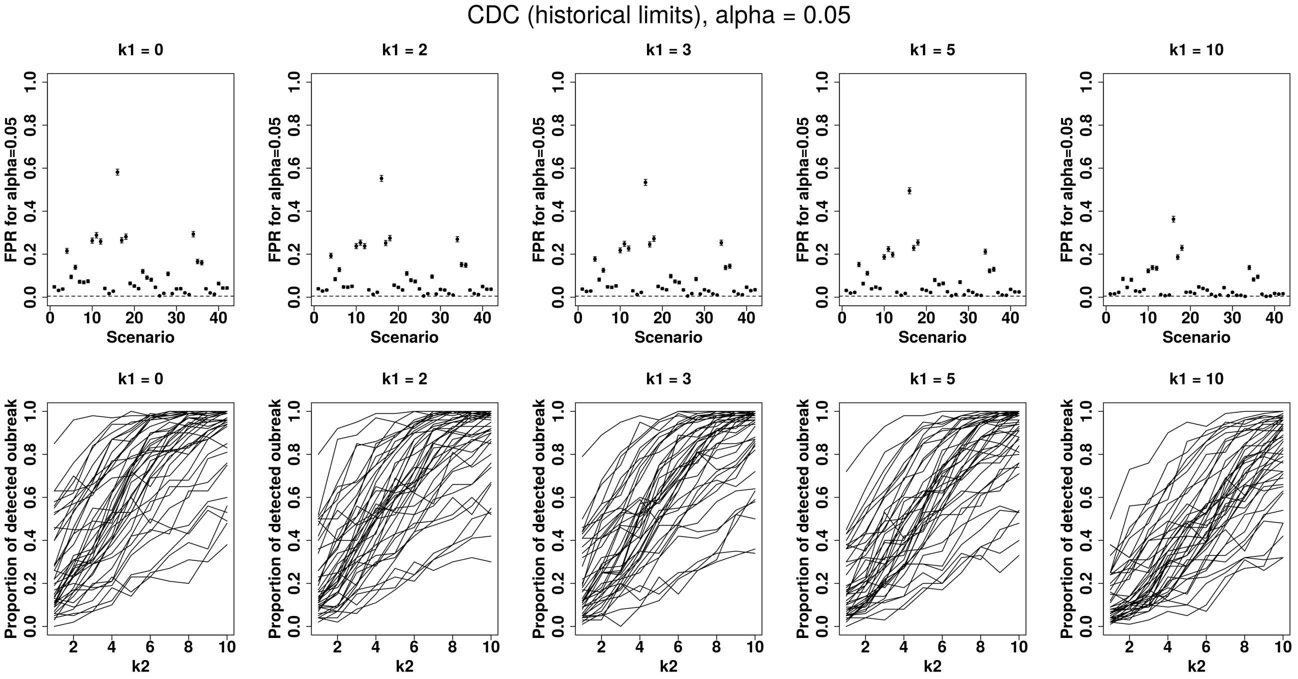

Figure 3: CDC algorithm performances for $\alpha = 0.05$ by increasing past outbreak amplitude $k_1 = 0$, 2, 3, 5 or 10 with (i) on the first row: false positive rate for 42 simulated scenarios, (ii) on the second row: probability of detection for 42 simulated scenarios (each curve corresponding to a scenario) by increasing current outbreak amplitude $k_2 = 1$ to 10.

Overall performances of CDC algorithm ($\alpha = 0.001$)

	FPR k1=0	FPR k1=2	FPR k1=3	FPR k1=5	FPR k1=10
1	0.01	0.00	0.00	0.00	0.00
2	0.01	0.00	0.01	0.00	0.00
3	0.01	0.00	0.00	0.00	0.01
4	0.04	0.04	0.03	0.02	0.01
5	0.01	0.01	0.01	0.01	0.01
6	0.01	0.01	0.01	0.01	0.01
7	0.03	0.02	0.02	0.01	0.01
8	0.03	0.02	0.02	0.01	0.01
9	0.03	0.02	0.02	0.02	0.01
10	0.07	0.06	0.06	0.04	0.03
11	0.08	0.07	0.06	0.05	0.03
12	0.08	0.06	0.06	0.05	0.03
13	0.00	0.00	0.00	0.00	0.00
14	0.00	0.00	0.00	0.00	0.00
15	0.00	0.00	0.00	0.00	0.00
16	0.07	0.06	0.06	0.05	0.03
17	0.02	0.02	0.02	0.02	0.01
18	0.03	0.03	0.03	0.02	0.01
19	0.02	0.01	0.01	0.01	0.01
20	0.02	0.01	0.01	0.01	0.01
21	0.01	0.01	0.01	0.01	0.00
22	0.03	0.03	0.02	0.02	0.01
23	0.02	0.02	0.01	0.01	0.01
24	0.01	0.01	0.01	0.01	0.00
25	0.01	0.00	0.00	0.00	0.00
26	0.00	0.00	0.00	0.00	0.00
27	0.00	0.00	0.00	0.00	0.00
28	0.02	0.01	0.01	0.01	0.01
29	0.00	0.00	0.00	0.00	0.00
30	0.00	0.00	0.00	0.00	0.00
31	0.00	0.00	0.00	0.00	0.00
32	0.00	0.00	0.00	0.00	0.00
33	0.00	0.00	0.00	0.00	0.00
34	0.03	0.02	0.02	0.02	0.01
35	0.01	0.01	0.01	0.01	0.01
36	0.01	0.01	0.01	0.01	0.00
37	0.00	0.00	0.00	0.00	0.00
38	0.00	0.00	0.00	0.00	0.00
39	0.00	0.00	0.00	0.00	0.00
40	0.01	0.00	0.00	0.00	0.00
41	0.00	0.00	0.00	0.00	0.00
42	0.00	0.00	0.00	0.00	0.00

Table 1: FPR according to each scenario and each k1 value, alpha = 0.001

	k2=1	k2=2	k2=3	k2=4	k2=5	k2=6	k2=7	k2=8	k2=9	k2=10
1	0.03	0.06	0.07	0.20	0.32	0.38	0.51	0.74	0.77	0.77
2	0.01	0.06	0.08	0.09	0.19	0.24	0.35	0.39	0.62	0.71
3	0.06	0.04	0.04	0.07	0.14	0.17	0.21	0.29	0.33	0.39
4	0.12	0.24	0.31	0.55	0.64	0.78	0.85	0.89	0.97	0.98
5	0.05	0.04	0.13	0.19	0.21	0.29	0.41	0.47	0.64	0.70
6	0.03	0.10	0.10	0.13	0.23	0.30	0.33	0.31	0.41	0.32
7	0.23	0.33	0.38	0.43	0.52	0.59	0.61	0.63	0.74	0.72
8	0.23	0.25	0.28	0.42	0.45	0.57	0.61	0.54	0.71	0.75
9	0.29	0.34	0.32	0.42	0.48	0.59	0.65	0.66	0.72	0.68
10	0.25	0.29	0.43	0.63	0.71	0.85	0.89	0.93	0.97	0.97
11	0.23	0.26	0.44	0.64	0.76	0.84	0.91	0.89	0.95	0.98
12	0.19	0.31	0.45	0.61	0.62	0.82	0.87	0.93	0.92	0.95
13	0.01	0.03	0.07	0.17	0.27	0.37	0.58	0.61	0.80	0.88
14	0.01	0.01	0.05	0.08	0.17	0.24	0.34	0.48	0.61	0.70
15	0.01	0.00	0.02	0.07	0.08	0.16	0.19	0.26	0.34	0.47
16	0.20	0.40	0.50	0.54	0.74	0.88	0.93	0.94	0.99	1.00
17	0.08	0.08	0.10	0.17	0.32	0.44	0.49	0.51	0.68	0.76
18	0.10	0.12	0.19	0.20	0.29	0.24	0.32	0.35	0.43	0.53
19	0.10	0.12	0.14	0.19	0.28	0.51	0.57	0.65	0.84	0.88
20	0.00	0.04	0.11	0.13	0.29	0.41	0.49	0.62	0.66	0.75
21	0.01	0.02	0.07	0.15	0.16	0.27	0.32	0.46	0.59	0.61
22	0.09	0.13	0.26	0.37	0.64	0.72	0.76	0.93	0.94	0.98
23	0.08	0.09	0.22	0.27	0.43	0.61	0.67	0.73	0.80	0.89
24	0.04	0.10	0.10	0.17	0.29	0.38	0.38	0.46	0.58	0.59
25	0.04	0.06	0.11	0.19	0.34	0.54	0.66	0.82	0.93	0.93
26	0.00	0.00	0.01	0.01	0.01	0.04	0.03	0.13	0.10	0.19
27	0.01	0.01	0.01	0.04	0.03	0.12	0.12	0.13	0.18	0.13
28	0.06	0.15	0.32	0.34	0.49	0.67	0.80	0.96	0.94	1.00
29	0.00	0.00	0.01	0.01	0.03	0.05	0.08	0.10	0.26	0.28
30	0.00	0.01	0.03	0.07	0.05	0.12	0.05	0.08	0.24	0.28
31	0.03	0.03	0.12	0.23	0.30	0.60	0.66	0.81	0.88	0.95
32	0.01	0.01	0.03	0.10	0.17	0.30	0.40	0.66	0.65	0.73
33	0.00	0.01	0.00	0.04	0.11	0.12	0.20	0.29	0.45	0.48
34	0.11	0.19	0.30	0.47	0.75	0.79	0.88	0.90	0.99	0.98
35	0.06	0.09	0.13	0.27	0.34	0.58	0.64	0.81	0.87	0.92
36	0.03	0.08	0.17	0.17	0.32	0.28	0.45	0.48	0.55	0.64
37	0.01	0.03	0.08	0.21	0.32	0.47	0.71	0.77	0.87	0.94
38	0.00	0.01	0.03	0.13	0.18	0.39	0.54	0.65	0.79	0.81
39	0.00	0.03	0.04	0.06	0.15	0.20	0.29	0.49	0.56	0.70
40	0.03	0.09	0.24	0.35	0.39	0.71	0.77	0.92	0.95	0.97
41	0.02	0.10	0.10	0.15	0.31	0.43	0.64	0.76	0.82	0.91
42	0.03	0.04	0.04	0.09	0.24	0.23	0.40	0.52	0.67	0.73

Table 2: POD according to each scenario and each k2 value, k1 = 0, alpha = 0.001

	k2=1	k2=2	k2=3	k2=4	k2=5	k2=6	k2=7	k2=8	k2=9	k2=10
1	0.04	0.08	0.12	0.16	0.29	0.39	0.41	0.67	0.66	0.72
2	0.03	0.09	0.03	0.14	0.27	0.25	0.36	0.46	0.53	0.61
3	0.04	0.04	0.02	0.05	0.11	0.22	0.23	0.29	0.34	0.46
4	0.12	0.27	0.26	0.42	0.62	0.75	0.82	0.86	0.96	1.00
5	0.04	0.09	0.08	0.20	0.19	0.36	0.44	0.55	0.58	0.65
6	0.03	0.05	0.12	0.15	0.22	0.25	0.35	0.32	0.48	0.43
7	0.13	0.14	0.20	0.33	0.42	0.39	0.53	0.58	0.64	0.65
8	0.16	0.10	0.18	0.28	0.37	0.40	0.45	0.55	0.61	0.71
9	0.12	0.19	0.19	0.28	0.35	0.38	0.49	0.50	0.64	0.68
10	0.19	0.31	0.49	0.59	0.71	0.84	0.87	0.86	0.99	0.98
11	0.17	0.31	0.52	0.46	0.71	0.78	0.91	0.95	0.96	0.98
12	0.17	0.28	0.32	0.43	0.59	0.77	0.84	0.85	0.89	0.97
13	0.01	0.04	0.08	0.16	0.29	0.40	0.57	0.66	0.80	0.82
14	0.02	0.00	0.01	0.07	0.07	0.17	0.29	0.37	0.51	0.63
15	0.00	0.00	0.02	0.07	0.14	0.19	0.15	0.31	0.35	0.40
16	0.15	0.38	0.39	0.60	0.77	0.87	0.86	0.93	0.99	0.98
17	0.05	0.06	0.12	0.22	0.22	0.38	0.41	0.52	0.68	0.78
18	0.06	0.11	0.16	0.18	0.29	0.26	0.25	0.42	0.35	0.51
19	0.05	0.08	0.12	0.25	0.33	0.40	0.52	0.67	0.76	0.83
20	0.03	0.09	0.11	0.10	0.18	0.40	0.50	0.47	0.70	0.75
21	0.03	0.03	0.08	0.08	0.13	0.21	0.25	0.38	0.45	0.59
22	0.07	0.17	0.23	0.42	0.51	0.67	0.82	0.89	0.92	0.97
23	0.05	0.08	0.19	0.23	0.35	0.48	0.55	0.75	0.77	0.83
24	0.07	0.04	0.12	0.15	0.24	0.31	0.37	0.49	0.50	0.48
25	0.03	0.07	0.10	0.17	0.31	0.40	0.52	0.68	0.84	0.92
26	0.00	0.00	0.01	0.00	0.02	0.04	0.10	0.07	0.16	0.15
27	0.00	0.00	0.02	0.04	0.05	0.10	0.12	0.15	0.19	0.19
28	0.04	0.09	0.19	0.36	0.49	0.68	0.73	0.89	0.96	0.97
29	0.01	0.00	0.00	0.01	0.04	0.04	0.08	0.13	0.14	0.23
30	0.00	0.01	0.07	0.05	0.06	0.10	0.13	0.14	0.23	0.23
31	0.03	0.03	0.11	0.14	0.26	0.46	0.65	0.78	0.84	0.93
32	0.00	0.02	0.04	0.07	0.13	0.21	0.30	0.49	0.60	0.79
33	0.00	0.01	0.03	0.02	0.08	0.10	0.20	0.26	0.34	0.43
34	0.11	0.17	0.29	0.49	0.62	0.78	0.85	0.96	0.94	1.00
35	0.04	0.07	0.14	0.15	0.32	0.45	0.62	0.74	0.86	0.89
36	0.04	0.04	0.12	0.15	0.26	0.30	0.34	0.45	0.54	0.52
37	0.02	0.05	0.09	0.23	0.25	0.44	0.64	0.67	0.86	0.93
38	0.00	0.00	0.04	0.14	0.21	0.36	0.47	0.66	0.80	0.91
39	0.00	0.00	0.01	0.05	0.14	0.26	0.28	0.44	0.51	0.69
40	0.01	0.08	0.11	0.25	0.43	0.61	0.75	0.85	0.84	0.95
41	0.03	0.04	0.12	0.19	0.20	0.46	0.60	0.64	0.82	0.85
42	0.02	0.03	0.06	0.15	0.24	0.24	0.30	0.53	0.68	0.72

Table 3: POD according to each scenario and each k2 value, k1 = 2, alpha = 0.001

	k2=1	k2=2	k2=3	k2=4	k2=5	k2=6	k2=7	k2=8	k2=9	k2=10
1	0.05	0.07	0.05	0.14	0.27	0.39	0.41	0.60	0.66	0.79
2	0.01	0.02	0.05	0.09	0.11	0.14	0.26	0.40	0.43	0.55
3	0.02	0.04	0.05	0.07	0.13	0.23	0.19	0.20	0.31	0.41
4	0.13	0.15	0.30	0.41	0.61	0.72	0.81	0.85	0.94	0.94
5	0.04	0.05	0.08	0.18	0.22	0.29	0.45	0.54	0.55	0.63
6	0.04	0.07	0.10	0.16	0.18	0.29	0.22	0.27	0.41	0.43
7	0.11	0.18	0.26	0.20	0.28	0.36	0.55	0.48	0.62	0.75
8	0.10	0.19	0.24	0.27	0.31	0.44	0.46	0.45	0.49	0.61
9	0.14	0.21	0.24	0.24	0.34	0.42	0.47	0.59	0.66	0.67
10	0.15	0.21	0.39	0.48	0.64	0.85	0.90	0.91	0.95	0.98
11	0.18	0.31	0.38	0.55	0.65	0.82	0.86	0.92	0.95	0.98
12	0.17	0.30	0.31	0.56	0.69	0.73	0.73	0.88	0.94	0.96
13	0.01	0.01	0.05	0.13	0.21	0.34	0.51	0.58	0.81	0.87
14	0.01	0.00	0.01	0.02	0.10	0.12	0.31	0.37	0.42	0.53
15	0.01	0.02	0.00	0.06	0.09	0.15	0.25	0.30	0.33	0.42
16	0.14	0.24	0.43	0.66	0.66	0.85	0.92	0.93	0.96	0.98
17	0.06	0.10	0.15	0.27	0.27	0.32	0.41	0.55	0.54	0.73
18	0.10	0.05	0.16	0.16	0.26	0.38	0.43	0.37	0.42	0.46
19	0.05	0.07	0.16	0.14	0.24	0.46	0.48	0.65	0.68	0.88
20	0.02	0.09	0.09	0.16	0.21	0.34	0.52	0.55	0.69	0.79
21	0.00	0.02	0.09	0.07	0.11	0.18	0.26	0.40	0.46	0.61
22	0.04	0.15	0.20	0.32	0.43	0.68	0.77	0.82	0.95	0.95
23	0.02	0.09	0.22	0.21	0.36	0.43	0.57	0.61	0.89	0.81
24	0.02	0.04	0.11	0.21	0.21	0.31	0.35	0.39	0.43	0.46
25	0.01	0.07	0.08	0.19	0.27	0.37	0.56	0.72	0.78	0.91
26	0.00	0.00	0.00	0.01	0.02	0.03	0.07	0.08	0.14	0.22
27	0.01	0.00	0.01	0.01	0.06	0.08	0.11	0.12	0.14	0.17
28	0.04	0.09	0.15	0.26	0.46	0.61	0.73	0.90	0.97	0.97
29	0.02	0.00	0.01	0.01	0.05	0.03	0.08	0.10	0.19	0.22
30	0.02	0.00	0.05	0.08	0.07	0.08	0.18	0.11	0.20	0.13
31	0.04	0.03	0.07	0.19	0.22	0.57	0.57	0.71	0.77	0.91
32	0.00	0.01	0.03	0.05	0.11	0.20	0.42	0.42	0.60	0.82
33	0.00	0.01	0.02	0.04	0.09	0.12	0.22	0.27	0.38	0.43
34	0.04	0.13	0.30	0.46	0.57	0.76	0.85	0.92	0.97	1.00
35	0.03	0.07	0.12	0.19	0.37	0.47	0.61	0.69	0.81	0.84
36	0.03	0.07	0.17	0.17	0.22	0.37	0.37	0.49	0.53	0.55
37	0.01	0.04	0.17	0.18	0.22	0.37	0.56	0.73	0.87	0.94
38	0.00	0.01	0.03	0.10	0.17	0.33	0.46	0.56	0.67	0.82
39	0.00	0.01	0.03	0.07	0.15	0.19	0.28	0.42	0.54	0.62
40	0.02	0.05	0.09	0.19	0.29	0.56	0.68	0.78	0.92	0.95
41	0.00	0.02	0.06	0.14	0.24	0.33	0.56	0.64	0.79	0.89
42	0.00	0.05	0.07	0.08	0.25	0.29	0.44	0.52	0.62	0.77

Table 4: POD according to each scenario and each k2 value, k1 = 3, alpha = 0.001

	k2=1	k2=2	k2=3	k2=4	k2=5	k2=6	k2=7	k2=8	k2=9	k2=10
1	0.02	0.05	0.09	0.18	0.22	0.20	0.44	0.56	0.60	0.73
2	0.02	0.00	0.08	0.09	0.11	0.20	0.30	0.35	0.45	0.52
3	0.01	0.00	0.06	0.10	0.07	0.06	0.15	0.26	0.28	0.26
4	0.11	0.19	0.19	0.41	0.47	0.62	0.74	0.84	0.92	0.98
5	0.03	0.01	0.11	0.16	0.20	0.26	0.31	0.46	0.53	0.54
6	0.07	0.09	0.13	0.17	0.14	0.23	0.31	0.32	0.42	0.34
7	0.11	0.15	0.14	0.30	0.31	0.33	0.40	0.50	0.59	0.66
8	0.16	0.22	0.16	0.30	0.29	0.36	0.45	0.47	0.55	0.58
9	0.15	0.19	0.23	0.21	0.37	0.42	0.41	0.58	0.55	0.57
10	0.11	0.25	0.31	0.55	0.66	0.74	0.84	0.89	0.95	0.97
11	0.16	0.23	0.42	0.47	0.63	0.67	0.86	0.87	0.94	0.95
12	0.15	0.29	0.35	0.48	0.60	0.60	0.71	0.80	0.86	0.97
13	0.00	0.07	0.08	0.13	0.18	0.31	0.36	0.58	0.71	0.75
14	0.00	0.00	0.03	0.05	0.09	0.18	0.24	0.39	0.44	0.59
15	0.01	0.01	0.02	0.04	0.10	0.06	0.16	0.25	0.33	0.41
16	0.12	0.37	0.28	0.52	0.68	0.75	0.76	0.93	0.97	0.97
17	0.03	0.08	0.12	0.11	0.25	0.36	0.43	0.43	0.59	0.70
18	0.06	0.12	0.13	0.14	0.26	0.29	0.36	0.36	0.38	0.49
19	0.04	0.07	0.10	0.14	0.19	0.34	0.42	0.58	0.66	0.69
20	0.01	0.01	0.08	0.09	0.18	0.23	0.39	0.41	0.57	0.59
21	0.01	0.03	0.04	0.12	0.14	0.22	0.35	0.30	0.36	0.52
22	0.04	0.09	0.17	0.24	0.37	0.53	0.73	0.78	0.90	0.93
23	0.06	0.05	0.12	0.11	0.22	0.39	0.50	0.68	0.81	0.83
24	0.03	0.07	0.09	0.14	0.22	0.27	0.26	0.41	0.42	0.52
25	0.02	0.04	0.09	0.10	0.17	0.43	0.49	0.71	0.78	0.88
26	0.00	0.00	0.00	0.02	0.04	0.04	0.02	0.07	0.11	0.14
27	0.00	0.02	0.03	0.01	0.02	0.07	0.17	0.20	0.14	0.18
28	0.06	0.05	0.10	0.24	0.35	0.46	0.72	0.78	0.90	0.91
29	0.00	0.00	0.01	0.00	0.02	0.02	0.08	0.05	0.18	0.18
30	0.01	0.00	0.03	0.08	0.04	0.13	0.16	0.15	0.16	0.24
31	0.02	0.05	0.07	0.06	0.14	0.33	0.54	0.62	0.68	0.84
32	0.01	0.00	0.03	0.10	0.09	0.14	0.29	0.30	0.53	0.67
33	0.00	0.01	0.01	0.03	0.04	0.06	0.17	0.24	0.34	0.36
34	0.07	0.14	0.17	0.27	0.45	0.63	0.83	0.92	0.92	0.93
35	0.03	0.07	0.11	0.18	0.24	0.45	0.51	0.55	0.66	0.86
36	0.03	0.04	0.13	0.18	0.20	0.26	0.29	0.41	0.43	0.54
37	0.01	0.01	0.01	0.12	0.21	0.33	0.51	0.61	0.74	0.84
38	0.00	0.00	0.03	0.04	0.12	0.25	0.42	0.57	0.66	0.82
39	0.01	0.01	0.00	0.04	0.07	0.16	0.26	0.26	0.42	0.61
40	0.03	0.00	0.10	0.15	0.28	0.48	0.61	0.71	0.89	0.90
41	0.00	0.01	0.06	0.09	0.18	0.31	0.49	0.64	0.65	0.81
42	0.01	0.00	0.00	0.10	0.10	0.13	0.36	0.50	0.50	0.69

Table 5: POD according to each scenario and each k2 value, k1 = 5, alpha = 0.001

	k2=1	k2=2	k2=3	k2=4	k2=5	k2=6	k2=7	k2=8	k2=9	k2=10
1	0.02	0.01	0.03	0.10	0.09	0.13	0.24	0.31	0.38	0.48
2	0.01	0.03	0.02	0.04	0.06	0.10	0.18	0.20	0.31	0.34
3	0.03	0.00	0.02	0.03	0.04	0.13	0.16	0.18	0.26	0.29
4	0.01	0.06	0.17	0.20	0.31	0.40	0.59	0.58	0.69	0.83
5	0.01	0.03	0.07	0.05	0.11	0.21	0.30	0.35	0.37	0.58
6	0.03	0.02	0.08	0.13	0.12	0.20	0.29	0.21	0.32	0.38
7	0.09	0.15	0.19	0.16	0.24	0.15	0.32	0.31	0.43	0.41
8	0.05	0.07	0.10	0.20	0.27	0.21	0.34	0.39	0.41	0.53
9	0.10	0.13	0.12	0.19	0.26	0.22	0.20	0.37	0.38	0.46
10	0.04	0.17	0.23	0.24	0.47	0.59	0.61	0.78	0.85	0.78
11	0.09	0.12	0.26	0.29	0.48	0.52	0.62	0.72	0.80	0.87
12	0.07	0.10	0.18	0.29	0.48	0.60	0.62	0.68	0.84	0.86
13	0.00	0.03	0.00	0.06	0.11	0.17	0.24	0.33	0.42	0.59
14	0.00	0.01	0.00	0.01	0.07	0.06	0.16	0.17	0.33	0.43
15	0.00	0.00	0.02	0.02	0.05	0.07	0.08	0.11	0.23	0.29
16	0.06	0.16	0.26	0.32	0.45	0.61	0.72	0.85	0.94	0.93
17	0.04	0.04	0.12	0.11	0.20	0.31	0.41	0.44	0.52	0.59
18	0.09	0.06	0.07	0.13	0.18	0.24	0.23	0.29	0.41	0.39
19	0.01	0.02	0.03	0.10	0.17	0.22	0.22	0.29	0.41	0.50
20	0.03	0.02	0.00	0.04	0.08	0.10	0.21	0.30	0.43	0.46
21	0.02	0.01	0.03	0.05	0.06	0.08	0.14	0.19	0.31	0.36
22	0.03	0.06	0.03	0.14	0.20	0.24	0.44	0.52	0.68	0.73
23	0.01	0.03	0.06	0.13	0.18	0.30	0.24	0.36	0.54	0.67
24	0.01	0.00	0.06	0.10	0.14	0.18	0.34	0.25	0.35	0.43
25	0.00	0.03	0.01	0.06	0.07	0.19	0.20	0.27	0.45	0.61
26	0.00	0.00	0.01	0.00	0.01	0.01	0.03	0.06	0.05	0.14
27	0.00	0.00	0.01	0.03	0.01	0.03	0.07	0.07	0.06	0.12
28	0.01	0.04	0.08	0.07	0.25	0.34	0.27	0.58	0.59	0.71
29	0.00	0.01	0.02	0.02	0.02	0.02	0.05	0.08	0.10	0.09
30	0.00	0.01	0.03	0.06	0.02	0.04	0.07	0.15	0.10	0.20
31	0.01	0.01	0.03	0.06	0.15	0.13	0.20	0.42	0.51	0.66
32	0.00	0.00	0.02	0.02	0.05	0.05	0.09	0.25	0.29	0.31
33	0.00	0.00	0.00	0.03	0.02	0.02	0.08	0.14	0.21	0.19
34	0.02	0.08	0.10	0.19	0.36	0.42	0.56	0.64	0.76	0.81
35	0.00	0.02	0.06	0.08	0.19	0.28	0.36	0.49	0.61	0.68
36	0.01	0.03	0.11	0.13	0.14	0.16	0.31	0.31	0.39	0.48
37	0.00	0.02	0.04	0.06	0.04	0.18	0.19	0.30	0.46	0.55
38	0.00	0.01	0.00	0.03	0.06	0.13	0.20	0.31	0.37	0.53
39	0.00	0.00	0.00	0.03	0.04	0.11	0.11	0.22	0.17	0.43
40	0.00	0.01	0.04	0.08	0.11	0.20	0.25	0.41	0.54	0.55
41	0.01	0.00	0.00	0.06	0.11	0.10	0.23	0.31	0.41	0.61
42	0.00	0.02	0.03	0.03	0.09	0.10	0.16	0.22	0.36	0.43

Table 6: POD according to each scenario and each k2 value, k1 = 10, alpha = 0.001

Overall performances of CDC algorithm ($\alpha = 0.01$)

	FPR k1=0	FPR k1=2	FPR k1=3	FPR k1=5	FPR k1=10
1	0.02	0.01	0.02	0.01	0.01
2	0.01	0.01	0.01	0.01	0.01
3	0.01	0.01	0.01	0.01	0.01
4	0.11	0.09	0.08	0.07	0.04
5	0.04	0.03	0.03	0.02	0.02
6	0.06	0.05	0.05	0.04	0.03
7	0.05	0.03	0.03	0.03	0.02
8	0.05	0.03	0.03	0.02	0.02
9	0.05	0.03	0.03	0.03	0.02
10	0.15	0.13	0.12	0.10	0.06
11	0.17	0.15	0.14	0.12	0.07
12	0.15	0.13	0.13	0.11	0.07
13	0.01	0.01	0.01	0.01	0.00
14	0.00	0.00	0.00	0.00	0.00
15	0.01	0.01	0.00	0.00	0.00
16	0.27	0.25	0.23	0.20	0.14
17	0.11	0.10	0.10	0.09	0.07
18	0.13	0.12	0.12	0.10	0.09
19	0.03	0.02	0.02	0.02	0.01
20	0.03	0.02	0.02	0.02	0.01
21	0.02	0.02	0.02	0.01	0.01
22	0.07	0.06	0.05	0.04	0.02
23	0.04	0.04	0.03	0.03	0.02
24	0.03	0.03	0.03	0.03	0.01
25	0.02	0.02	0.01	0.01	0.01
26	0.00	0.00	0.00	0.00	0.00
27	0.01	0.00	0.00	0.00	0.00
28	0.05	0.04	0.03	0.03	0.02
29	0.00	0.00	0.00	0.00	0.00
30	0.01	0.01	0.01	0.01	0.01
31	0.01	0.01	0.01	0.01	0.00
32	0.01	0.00	0.00	0.00	0.00
33	0.00	0.00	0.00	0.00	0.00
34	0.11	0.10	0.09	0.07	0.04
35	0.05	0.05	0.04	0.04	0.02
36	0.06	0.05	0.05	0.04	0.03
37	0.01	0.01	0.01	0.01	0.01
38	0.00	0.00	0.00	0.00	0.00
39	0.00	0.00	0.00	0.00	0.00
40	0.02	0.01	0.01	0.01	0.01
41	0.01	0.01	0.01	0.01	0.01
42	0.01	0.01	0.01	0.01	0.01

Table 7: FPR according to each scenario and each k1 value, alpha = 0.01

	k2=1	k2=2	k2=3	k2=4	k2=5	k2=6	k2=7	k2=8	k2=9	k2=10
1	0.06	0.13	0.18	0.33	0.41	0.60	0.71	0.84	0.87	0.89
2	0.08	0.11	0.17	0.24	0.35	0.39	0.63	0.68	0.75	0.87
3	0.10	0.11	0.11	0.11	0.22	0.26	0.33	0.36	0.46	0.49
4	0.24	0.37	0.51	0.73	0.80	0.94	0.94	0.98	0.99	0.99
5	0.12	0.12	0.24	0.35	0.38	0.52	0.59	0.68	0.84	0.87
6	0.12	0.20	0.23	0.20	0.35	0.41	0.39	0.39	0.48	0.40
7	0.42	0.45	0.52	0.57	0.63	0.73	0.73	0.76	0.84	0.84
8	0.36	0.36	0.42	0.58	0.64	0.69	0.73	0.66	0.81	0.91
9	0.45	0.53	0.46	0.53	0.61	0.72	0.80	0.81	0.82	0.75
10	0.38	0.47	0.69	0.81	0.81	0.94	0.94	1.00	1.00	1.00
11	0.41	0.49	0.69	0.80	0.91	0.92	0.99	0.98	0.96	0.98
12	0.34	0.47	0.62	0.76	0.80	0.95	0.93	0.99	0.96	0.97
13	0.03	0.10	0.20	0.37	0.48	0.64	0.70	0.77	0.92	0.98
14	0.02	0.05	0.12	0.14	0.30	0.41	0.52	0.66	0.78	0.83
15	0.02	0.04	0.04	0.09	0.18	0.29	0.31	0.44	0.49	0.65
16	0.57	0.72	0.83	0.85	0.97	0.96	0.98	1.00	1.00	1.00
17	0.18	0.24	0.29	0.46	0.57	0.68	0.75	0.83	0.91	0.97
18	0.26	0.32	0.39	0.36	0.49	0.32	0.49	0.57	0.54	0.66
19	0.12	0.16	0.22	0.32	0.48	0.66	0.76	0.83	0.93	0.96
20	0.03	0.07	0.19	0.28	0.44	0.61	0.63	0.82	0.78	0.86
21	0.09	0.06	0.12	0.19	0.25	0.37	0.47	0.63	0.69	0.75
22	0.17	0.28	0.47	0.53	0.79	0.82	0.88	0.97	0.98	0.99
23	0.18	0.23	0.36	0.42	0.68	0.76	0.86	0.85	0.92	0.97
24	0.05	0.18	0.16	0.29	0.46	0.47	0.49	0.59	0.68	0.71
25	0.08	0.17	0.29	0.40	0.56	0.72	0.80	0.89	0.97	0.98
26	0.00	0.00	0.04	0.04	0.08	0.09	0.16	0.22	0.20	0.35
27	0.01	0.03	0.01	0.08	0.07	0.15	0.19	0.17	0.23	0.22
28	0.12	0.30	0.48	0.61	0.76	0.90	0.96	0.99	0.98	1.00
29	0.01	0.03	0.03	0.06	0.09	0.13	0.20	0.25	0.40	0.36
30	0.04	0.04	0.07	0.14	0.10	0.16	0.09	0.11	0.27	0.35
31	0.08	0.11	0.21	0.37	0.58	0.77	0.85	0.95	0.96	0.99
32	0.03	0.04	0.09	0.18	0.36	0.54	0.59	0.77	0.85	0.93
33	0.01	0.02	0.11	0.13	0.22	0.28	0.36	0.41	0.67	0.65
34	0.32	0.49	0.60	0.80	0.95	0.96	0.98	0.99	1.00	1.00
35	0.20	0.22	0.41	0.63	0.69	0.85	0.89	0.96	0.96	0.99
36	0.13	0.26	0.34	0.32	0.51	0.50	0.60	0.73	0.65	0.80
37	0.03	0.13	0.26	0.39	0.56	0.71	0.78	0.91	0.96	0.98
38	0.02	0.05	0.10	0.27	0.45	0.58	0.78	0.85	0.92	0.93
39	0.00	0.05	0.10	0.20	0.33	0.46	0.53	0.73	0.81	0.91
40	0.08	0.15	0.43	0.58	0.73	0.87	0.92	0.97	0.98	1.00
41	0.06	0.20	0.25	0.36	0.56	0.71	0.80	0.92	0.93	0.97
42	0.04	0.13	0.14	0.26	0.41	0.50	0.73	0.75	0.87	0.91

Table 8: POD according to each scenario and each k2 value, k1 = 0, alpha = 0.01

	k2=1	k2=2	k2=3	k2=4	k2=5	k2=6	k2=7	k2=8	k2=9	k2=10
1	0.10	0.15	0.19	0.26	0.48	0.49	0.56	0.80	0.77	0.84
2	0.05	0.12	0.09	0.23	0.42	0.45	0.46	0.58	0.70	0.82
3	0.06	0.07	0.06	0.11	0.24	0.36	0.34	0.41	0.44	0.58
4	0.25	0.44	0.47	0.58	0.80	0.87	0.93	0.97	0.98	1.00
5	0.10	0.17	0.19	0.36	0.36	0.57	0.64	0.79	0.79	0.82
6	0.12	0.17	0.20	0.22	0.31	0.34	0.43	0.41	0.55	0.45
7	0.23	0.33	0.32	0.47	0.52	0.53	0.69	0.74	0.78	0.79
8	0.33	0.26	0.35	0.39	0.46	0.56	0.59	0.65	0.68	0.80
9	0.19	0.28	0.37	0.48	0.48	0.60	0.60	0.66	0.70	0.77
10	0.32	0.54	0.66	0.78	0.82	0.88	0.95	0.97	1.00	1.00
11	0.32	0.43	0.69	0.74	0.88	0.85	0.94	1.00	0.98	0.99
12	0.32	0.48	0.53	0.66	0.75	0.87	0.94	0.94	0.96	0.99
13	0.02	0.09	0.20	0.33	0.50	0.51	0.80	0.84	0.92	0.93
14	0.03	0.01	0.06	0.17	0.20	0.31	0.51	0.58	0.74	0.81
15	0.00	0.01	0.08	0.14	0.18	0.29	0.34	0.50	0.44	0.51
16	0.45	0.68	0.81	0.91	0.95	0.98	0.97	1.00	1.00	1.00
17	0.20	0.23	0.35	0.48	0.55	0.69	0.74	0.84	0.91	0.94
18	0.22	0.24	0.27	0.41	0.44	0.40	0.40	0.56	0.58	0.66
19	0.09	0.14	0.23	0.33	0.47	0.51	0.75	0.80	0.89	0.95
20	0.07	0.12	0.21	0.27	0.33	0.58	0.66	0.70	0.83	0.93
21	0.07	0.05	0.14	0.15	0.25	0.34	0.42	0.59	0.57	0.76
22	0.14	0.26	0.37	0.54	0.67	0.83	0.93	0.97	0.98	1.00
23	0.09	0.19	0.31	0.38	0.57	0.66	0.77	0.90	0.91	0.91
24	0.10	0.11	0.21	0.28	0.36	0.48	0.47	0.67	0.58	0.63
25	0.05	0.14	0.20	0.35	0.58	0.60	0.73	0.85	0.95	0.99
26	0.01	0.00	0.04	0.02	0.07	0.11	0.18	0.26	0.27	0.31
27	0.00	0.03	0.09	0.11	0.13	0.20	0.19	0.22	0.24	0.24
28	0.09	0.23	0.35	0.61	0.75	0.85	0.93	0.95	1.00	0.99
29	0.01	0.02	0.03	0.07	0.08	0.08	0.22	0.22	0.31	0.35
30	0.05	0.02	0.12	0.12	0.12	0.14	0.22	0.18	0.31	0.32
31	0.05	0.08	0.25	0.36	0.51	0.69	0.84	0.92	0.92	0.98
32	0.05	0.06	0.15	0.14	0.38	0.41	0.56	0.72	0.80	0.88
33	0.00	0.05	0.08	0.09	0.18	0.24	0.41	0.42	0.52	0.58
34	0.31	0.52	0.65	0.80	0.91	0.98	0.97	1.00	0.99	1.00
35	0.10	0.24	0.36	0.44	0.60	0.72	0.89	0.95	0.97	0.99
36	0.15	0.15	0.29	0.35	0.43	0.48	0.54	0.66	0.65	0.67
37	0.05	0.09	0.21	0.48	0.59	0.71	0.82	0.84	0.94	0.98
38	0.02	0.05	0.14	0.30	0.42	0.66	0.73	0.82	0.94	0.98
39	0.02	0.01	0.07	0.18	0.29	0.42	0.59	0.65	0.75	0.89
40	0.12	0.29	0.28	0.46	0.73	0.82	0.88	0.99	0.95	0.99
41	0.07	0.12	0.27	0.41	0.44	0.68	0.90	0.87	0.98	0.99
42	0.03	0.06	0.16	0.25	0.35	0.40	0.67	0.77	0.85	0.90

Table 9: POD according to each scenario and each k2 value, k1 = 2, alpha = 0.01

	k2=1	k2=2	k2=3	k2=4	k2=5	k2=6	k2=7	k2=8	k2=9	k2=10
1	0.09	0.08	0.12	0.27	0.41	0.56	0.59	0.82	0.83	0.94
2	0.04	0.09	0.12	0.18	0.27	0.29	0.45	0.55	0.62	0.74
3	0.05	0.11	0.10	0.14	0.23	0.31	0.30	0.36	0.41	0.46
4	0.24	0.35	0.49	0.62	0.79	0.88	0.93	0.93	0.98	0.96
5	0.07	0.14	0.18	0.31	0.38	0.45	0.65	0.78	0.75	0.82
6	0.09	0.18	0.19	0.26	0.28	0.36	0.30	0.38	0.47	0.51
7	0.20	0.30	0.41	0.44	0.39	0.58	0.68	0.64	0.71	0.85
8	0.21	0.28	0.34	0.40	0.45	0.58	0.61	0.63	0.67	0.76
9	0.28	0.33	0.34	0.37	0.46	0.58	0.56	0.71	0.79	0.79
10	0.27	0.36	0.63	0.68	0.89	0.91	0.96	0.97	0.98	1.00
11	0.34	0.50	0.61	0.76	0.82	0.94	0.95	0.97	0.99	0.99
12	0.25	0.40	0.44	0.72	0.85	0.83	0.88	0.97	0.97	0.98
13	0.05	0.04	0.21	0.24	0.44	0.54	0.70	0.81	0.89	0.96
14	0.03	0.02	0.06	0.16	0.31	0.32	0.48	0.68	0.67	0.79
15	0.02	0.04	0.05	0.13	0.21	0.32	0.32	0.44	0.47	0.51
16	0.50	0.56	0.81	0.90	0.92	0.97	1.00	1.00	1.00	0.99
17	0.21	0.22	0.39	0.46	0.49	0.62	0.72	0.86	0.90	0.94
18	0.22	0.25	0.33	0.26	0.44	0.50	0.55	0.50	0.56	0.64
19	0.08	0.18	0.24	0.32	0.46	0.63	0.67	0.80	0.78	0.96
20	0.05	0.15	0.14	0.21	0.33	0.47	0.66	0.72	0.82	0.94
21	0.02	0.07	0.14	0.16	0.20	0.30	0.45	0.50	0.59	0.75
22	0.10	0.24	0.35	0.55	0.64	0.77	0.87	0.94	0.96	0.97
23	0.05	0.14	0.34	0.30	0.51	0.62	0.79	0.82	0.94	0.95
24	0.08	0.15	0.16	0.31	0.37	0.45	0.46	0.50	0.54	0.65
25	0.05	0.09	0.17	0.34	0.53	0.65	0.79	0.89	0.96	0.95
26	0.00	0.02	0.00	0.03	0.05	0.10	0.12	0.23	0.33	0.36
27	0.01	0.01	0.09	0.08	0.11	0.11	0.17	0.20	0.22	0.28
28	0.08	0.21	0.39	0.46	0.69	0.89	0.89	0.97	1.00	1.00
29	0.04	0.02	0.04	0.03	0.13	0.07	0.16	0.21	0.36	0.31
30	0.04	0.05	0.09	0.11	0.12	0.11	0.21	0.23	0.27	0.24
31	0.07	0.14	0.22	0.37	0.36	0.75	0.75	0.88	0.94	0.98
32	0.00	0.05	0.11	0.15	0.24	0.40	0.64	0.67	0.76	0.94
33	0.01	0.04	0.09	0.12	0.18	0.30	0.37	0.48	0.54	0.57
34	0.26	0.32	0.61	0.66	0.81	0.95	0.98	0.98	1.00	1.00
35	0.07	0.25	0.41	0.49	0.66	0.76	0.89	0.93	0.98	1.00
36	0.14	0.18	0.30	0.39	0.35	0.52	0.53	0.67	0.69	0.78
37	0.07	0.11	0.29	0.34	0.51	0.65	0.79	0.87	0.92	0.99
38	0.02	0.06	0.10	0.23	0.40	0.57	0.73	0.82	0.86	0.97
39	0.01	0.06	0.14	0.18	0.27	0.34	0.54	0.66	0.78	0.81
40	0.05	0.19	0.24	0.47	0.62	0.80	0.86	0.95	0.98	0.99
41	0.02	0.10	0.20	0.32	0.38	0.66	0.80	0.92	0.96	0.97
42	0.03	0.09	0.12	0.18	0.37	0.45	0.64	0.70	0.85	0.89

Table 10: POD according to each scenario and each k2 value, k1 = 3, alpha = 0.01

	k2=1	k2=2	k2=3	k2=4	k2=5	k2=6	k2=7	k2=8	k2=9	k2=10
1	0.07	0.14	0.16	0.25	0.37	0.38	0.60	0.80	0.77	0.85
2	0.05	0.06	0.14	0.20	0.23	0.35	0.48	0.53	0.62	0.65
3	0.04	0.06	0.11	0.17	0.18	0.16	0.29	0.37	0.40	0.37
4	0.23	0.35	0.43	0.61	0.69	0.76	0.90	0.95	0.97	0.99
5	0.06	0.10	0.21	0.29	0.33	0.50	0.52	0.74	0.77	0.70
6	0.09	0.16	0.22	0.26	0.21	0.29	0.36	0.45	0.47	0.40
7	0.19	0.19	0.25	0.43	0.54	0.45	0.57	0.65	0.69	0.81
8	0.22	0.30	0.27	0.41	0.46	0.44	0.55	0.63	0.69	0.76
9	0.21	0.27	0.35	0.32	0.44	0.57	0.56	0.67	0.69	0.68
10	0.27	0.46	0.58	0.71	0.84	0.85	0.93	0.95	0.98	1.00
11	0.27	0.37	0.59	0.69	0.81	0.84	0.94	0.97	0.99	0.98
12	0.26	0.38	0.50	0.59	0.72	0.78	0.82	0.91	0.90	0.99
13	0.02	0.12	0.19	0.26	0.34	0.55	0.60	0.78	0.84	0.90
14	0.00	0.03	0.09	0.09	0.24	0.30	0.43	0.60	0.66	0.82
15	0.03	0.04	0.02	0.17	0.19	0.16	0.29	0.34	0.50	0.52
16	0.37	0.58	0.72	0.83	0.89	0.95	0.98	1.00	1.00	0.99
17	0.15	0.22	0.27	0.36	0.65	0.68	0.73	0.75	0.83	0.91
18	0.25	0.22	0.36	0.28	0.40	0.45	0.46	0.55	0.50	0.60
19	0.08	0.14	0.17	0.25	0.30	0.49	0.66	0.76	0.82	0.83
20	0.04	0.03	0.11	0.20	0.39	0.41	0.65	0.63	0.80	0.75
21	0.07	0.06	0.09	0.17	0.25	0.33	0.46	0.40	0.59	0.65
22	0.11	0.20	0.33	0.44	0.53	0.70	0.85	0.89	0.96	0.99
23	0.06	0.11	0.24	0.26	0.42	0.57	0.74	0.86	0.90	0.97
24	0.10	0.15	0.15	0.24	0.33	0.37	0.37	0.54	0.52	0.68
25	0.06	0.09	0.15	0.29	0.34	0.55	0.68	0.83	0.95	0.96
26	0.00	0.02	0.01	0.03	0.07	0.12	0.14	0.20	0.26	0.33
27	0.01	0.02	0.09	0.08	0.09	0.11	0.26	0.25	0.20	0.21
28	0.14	0.18	0.26	0.34	0.55	0.67	0.86	0.92	0.95	1.00
29	0.01	0.01	0.05	0.01	0.08	0.06	0.19	0.18	0.29	0.28
30	0.04	0.02	0.09	0.10	0.13	0.15	0.21	0.25	0.23	0.32
31	0.08	0.11	0.22	0.22	0.33	0.60	0.72	0.83	0.91	0.95
32	0.01	0.07	0.11	0.25	0.23	0.42	0.52	0.64	0.83	0.88
33	0.01	0.01	0.03	0.04	0.14	0.14	0.37	0.46	0.54	0.54
34	0.17	0.32	0.49	0.60	0.70	0.87	0.97	0.99	0.98	0.98
35	0.10	0.19	0.25	0.42	0.58	0.74	0.76	0.86	0.92	0.97
36	0.11	0.14	0.32	0.37	0.42	0.49	0.49	0.57	0.62	0.74
37	0.02	0.03	0.15	0.28	0.42	0.64	0.72	0.81	0.89	0.95
38	0.00	0.03	0.09	0.13	0.35	0.50	0.66	0.75	0.90	0.91
39	0.02	0.02	0.04	0.11	0.25	0.29	0.58	0.54	0.70	0.83
40	0.09	0.06	0.27	0.34	0.56	0.69	0.87	0.88	0.98	0.97
41	0.04	0.09	0.14	0.30	0.34	0.59	0.74	0.82	0.91	0.94
42	0.03	0.04	0.05	0.26	0.25	0.44	0.58	0.74	0.68	0.85

Table 11: POD according to each scenario and each k2 value, k1 = 5, alpha = 0.01

	k2=1	k2=2	k2=3	k2=4	k2=5	k2=6	k2=7	k2=8	k2=9	k2=10
1	0.08	0.03	0.10	0.16	0.19	0.29	0.37	0.44	0.55	0.63
2	0.05	0.04	0.05	0.07	0.14	0.22	0.29	0.35	0.44	0.46
3	0.03	0.03	0.03	0.09	0.11	0.20	0.22	0.24	0.33	0.36
4	0.10	0.12	0.28	0.37	0.46	0.58	0.77	0.79	0.86	0.93
5	0.02	0.04	0.14	0.16	0.25	0.29	0.47	0.56	0.53	0.81
6	0.05	0.08	0.17	0.21	0.18	0.26	0.37	0.31	0.46	0.44
7	0.18	0.18	0.23	0.24	0.32	0.24	0.42	0.47	0.56	0.57
8	0.09	0.17	0.17	0.33	0.38	0.31	0.51	0.50	0.57	0.67
9	0.16	0.18	0.22	0.28	0.37	0.35	0.36	0.50	0.54	0.56
10	0.09	0.29	0.34	0.39	0.69	0.71	0.83	0.89	0.91	0.91
11	0.21	0.28	0.39	0.45	0.67	0.74	0.84	0.83	0.90	0.94
12	0.19	0.26	0.34	0.48	0.62	0.73	0.79	0.85	0.96	0.94
13	0.04	0.06	0.03	0.16	0.18	0.32	0.37	0.52	0.62	0.73
14	0.02	0.02	0.02	0.05	0.18	0.16	0.30	0.34	0.56	0.56
15	0.02	0.02	0.05	0.04	0.09	0.15	0.24	0.21	0.36	0.49
16	0.26	0.39	0.55	0.66	0.73	0.81	0.92	0.96	0.97	0.99
17	0.13	0.14	0.31	0.31	0.47	0.52	0.70	0.66	0.78	0.84
18	0.22	0.21	0.25	0.32	0.33	0.37	0.48	0.45	0.56	0.50
19	0.03	0.06	0.06	0.14	0.25	0.41	0.38	0.46	0.65	0.70
20	0.04	0.04	0.06	0.14	0.14	0.24	0.35	0.44	0.61	0.68
21	0.03	0.03	0.06	0.07	0.14	0.16	0.27	0.34	0.46	0.51
22	0.06	0.15	0.19	0.29	0.33	0.44	0.70	0.71	0.84	0.89
23	0.02	0.06	0.14	0.22	0.35	0.44	0.50	0.64	0.77	0.82
24	0.02	0.04	0.08	0.24	0.24	0.27	0.43	0.36	0.49	0.53
25	0.01	0.03	0.05	0.15	0.13	0.37	0.42	0.51	0.70	0.84
26	0.00	0.00	0.02	0.04	0.02	0.04	0.07	0.15	0.15	0.29
27	0.00	0.02	0.04	0.04	0.04	0.04	0.11	0.14	0.17	0.18
28	0.06	0.10	0.15	0.24	0.44	0.54	0.59	0.75	0.87	0.85
29	0.00	0.01	0.03	0.07	0.05	0.08	0.14	0.20	0.17	0.22
30	0.02	0.02	0.06	0.17	0.12	0.09	0.15	0.19	0.18	0.25
31	0.04	0.03	0.13	0.17	0.21	0.27	0.38	0.67	0.74	0.84
32	0.00	0.01	0.04	0.07	0.13	0.18	0.29	0.46	0.58	0.57
33	0.00	0.03	0.03	0.08	0.16	0.14	0.18	0.27	0.40	0.44
34	0.08	0.26	0.30	0.45	0.61	0.73	0.87	0.87	0.95	0.93
35	0.06	0.11	0.24	0.27	0.39	0.52	0.65	0.69	0.87	0.89
36	0.10	0.14	0.20	0.29	0.32	0.33	0.44	0.56	0.57	0.68
37	0.01	0.02	0.09	0.18	0.25	0.33	0.37	0.51	0.65	0.82
38	0.00	0.02	0.03	0.13	0.18	0.29	0.43	0.50	0.60	0.79
39	0.00	0.01	0.03	0.09	0.11	0.21	0.26	0.38	0.43	0.62
40	0.03	0.05	0.12	0.18	0.25	0.30	0.48	0.61	0.82	0.81
41	0.02	0.02	0.08	0.15	0.27	0.21	0.39	0.60	0.66	0.81
42	0.02	0.03	0.05	0.12	0.16	0.27	0.33	0.46	0.53	0.66

Table 12: POD according to each scenario and each k2 value, k1 = 10, alpha = 0.01

Overall performances of CDC algorithm ($\alpha = 0.05$)

	FPR k1=0	FPR k1=2	FPR k1=3	FPR k1=5	FPR k1=10
1	0.05	0.04	0.04	0.03	0.02
2	0.03	0.03	0.03	0.02	0.02
3	0.04	0.03	0.03	0.02	0.02
4	0.22	0.19	0.18	0.15	0.09
5	0.09	0.08	0.08	0.06	0.05
6	0.14	0.13	0.13	0.11	0.08
7	0.07	0.05	0.05	0.04	0.03
8	0.07	0.05	0.05	0.05	0.03
9	0.07	0.05	0.05	0.04	0.04
10	0.26	0.24	0.22	0.19	0.12
11	0.29	0.25	0.25	0.22	0.14
12	0.26	0.24	0.23	0.20	0.13
13	0.04	0.03	0.03	0.02	0.01
14	0.02	0.01	0.01	0.01	0.01
15	0.03	0.02	0.02	0.02	0.01
16	0.58	0.55	0.53	0.49	0.36
17	0.26	0.25	0.25	0.23	0.19
18	0.28	0.27	0.27	0.25	0.23
19	0.06	0.06	0.05	0.04	0.02
20	0.05	0.05	0.04	0.03	0.02
21	0.04	0.03	0.03	0.02	0.02
22	0.12	0.11	0.10	0.08	0.05
23	0.09	0.08	0.07	0.06	0.04
24	0.08	0.07	0.07	0.06	0.03
25	0.05	0.04	0.03	0.03	0.01
26	0.01	0.01	0.01	0.01	0.00
27	0.02	0.02	0.02	0.01	0.01
28	0.11	0.10	0.08	0.07	0.04
29	0.02	0.02	0.02	0.01	0.01
30	0.04	0.04	0.03	0.03	0.02
31	0.04	0.03	0.03	0.02	0.01
32	0.02	0.02	0.02	0.01	0.01
33	0.01	0.01	0.01	0.01	0.00
34	0.29	0.27	0.25	0.21	0.14
35	0.17	0.15	0.14	0.12	0.08
36	0.16	0.15	0.14	0.13	0.09
37	0.04	0.03	0.03	0.02	0.01
38	0.02	0.02	0.01	0.01	0.00
39	0.01	0.01	0.01	0.01	0.01
40	0.06	0.05	0.05	0.04	0.02
41	0.04	0.04	0.03	0.02	0.01
42	0.04	0.04	0.03	0.02	0.02

Table 13: FPR according to each scenario and each k1 value, alpha = 0.05

	k2=1	k2=2	k2=3	k2=4	k2=5	k2=6	k2=7	k2=8	k2=9	k2=10
1	0.19	0.31	0.34	0.47	0.60	0.74	0.86	0.94	0.95	0.96
2	0.14	0.23	0.29	0.38	0.50	0.54	0.75	0.84	0.87	0.94
3	0.17	0.20	0.18	0.19	0.33	0.40	0.45	0.47	0.58	0.60
4	0.40	0.59	0.68	0.87	0.91	0.98	0.98	1.00	1.00	1.00
5	0.26	0.23	0.37	0.53	0.53	0.70	0.82	0.83	0.91	0.95
6	0.22	0.31	0.29	0.32	0.38	0.50	0.46	0.43	0.54	0.49
7	0.63	0.63	0.66	0.73	0.79	0.82	0.81	0.84	0.91	0.94
8	0.53	0.57	0.61	0.77	0.78	0.81	0.82	0.80	0.91	0.94
9	0.56	0.70	0.62	0.69	0.70	0.79	0.82	0.90	0.88	0.83
10	0.55	0.67	0.83	0.94	0.94	0.98	0.96	1.00	1.00	1.00
11	0.58	0.62	0.84	0.90	0.95	0.96	1.00	0.99	0.98	1.00
12	0.52	0.59	0.75	0.87	0.86	0.97	0.99	1.00	0.98	1.00
13	0.09	0.24	0.32	0.58	0.74	0.83	0.84	0.92	0.97	0.99
14	0.09	0.16	0.29	0.34	0.45	0.69	0.70	0.80	0.89	0.96
15	0.11	0.17	0.17	0.21	0.35	0.45	0.43	0.63	0.63	0.75
16	0.85	0.96	0.98	0.97	0.98	0.98	1.00	1.00	1.00	1.00
17	0.45	0.55	0.69	0.76	0.85	0.96	0.97	0.99	0.98	1.00
18	0.46	0.45	0.45	0.50	0.60	0.48	0.65	0.65	0.67	0.76
19	0.20	0.24	0.37	0.52	0.71	0.84	0.89	0.94	1.00	0.99
20	0.10	0.23	0.31	0.41	0.56	0.77	0.81	0.93	0.92	0.97
21	0.13	0.11	0.19	0.27	0.42	0.58	0.61	0.72	0.87	0.94
22	0.28	0.43	0.63	0.74	0.87	0.93	0.96	0.99	0.99	1.00
23	0.29	0.38	0.55	0.54	0.74	0.90	0.91	0.97	0.95	1.00
24	0.11	0.27	0.30	0.46	0.53	0.59	0.68	0.68	0.80	0.85
25	0.14	0.26	0.46	0.62	0.82	0.89	0.95	0.93	0.98	1.00
26	0.00	0.02	0.07	0.10	0.23	0.23	0.33	0.39	0.36	0.56
27	0.04	0.06	0.07	0.18	0.14	0.25	0.27	0.31	0.30	0.38
28	0.28	0.54	0.63	0.81	0.90	0.97	0.98	0.99	0.99	1.00
29	0.06	0.05	0.09	0.12	0.24	0.26	0.35	0.44	0.56	0.53
30	0.12	0.09	0.12	0.23	0.16	0.23	0.21	0.20	0.34	0.48
31	0.15	0.30	0.41	0.53	0.72	0.90	0.92	0.97	0.97	1.00
32	0.06	0.14	0.25	0.40	0.61	0.73	0.87	0.95	0.93	0.96
33	0.05	0.08	0.20	0.21	0.42	0.44	0.55	0.60	0.78	0.81
34	0.63	0.76	0.86	0.95	1.00	0.98	1.00	1.00	1.00	1.00
35	0.40	0.53	0.67	0.87	0.92	0.99	0.98	1.00	1.00	1.00
36	0.26	0.43	0.49	0.48	0.72	0.65	0.79	0.85	0.87	0.93
37	0.10	0.28	0.50	0.63	0.75	0.85	0.90	0.98	0.99	1.00
38	0.07	0.18	0.35	0.50	0.69	0.80	0.91	0.97	0.96	0.96
39	0.03	0.16	0.24	0.40	0.53	0.69	0.78	0.88	0.95	0.96
40	0.22	0.46	0.63	0.76	0.86	0.96	0.97	0.99	1.00	1.00
41	0.13	0.33	0.36	0.62	0.74	0.87	0.89	0.99	0.98	1.00
42	0.11	0.28	0.31	0.42	0.67	0.73	0.90	0.90	0.97	1.00

Table 14: POD according to each scenario and each k2 value, k1 = 0, alpha = 0.05

	k2=1	k2=2	k2=3	k2=4	k2=5	k2=6	k2=7	k2=8	k2=9	k2=10
1	0.16	0.25	0.38	0.43	0.69	0.62	0.79	0.90	0.88	0.92
2	0.12	0.25	0.19	0.40	0.55	0.56	0.63	0.73	0.79	0.90
3	0.08	0.16	0.10	0.19	0.31	0.44	0.47	0.50	0.56	0.67
4	0.42	0.62	0.68	0.83	0.88	0.96	0.99	1.00	0.99	1.00
5	0.21	0.34	0.37	0.55	0.54	0.72	0.85	0.93	0.93	0.93
6	0.23	0.25	0.30	0.28	0.41	0.48	0.49	0.52	0.60	0.52
7	0.40	0.54	0.47	0.65	0.62	0.65	0.78	0.83	0.86	0.90
8	0.49	0.40	0.48	0.53	0.65	0.67	0.73	0.83	0.81	0.87
9	0.36	0.40	0.51	0.63	0.63	0.74	0.73	0.80	0.87	0.85
10	0.47	0.67	0.84	0.86	0.90	0.93	0.99	0.98	1.00	1.00
11	0.50	0.59	0.85	0.83	0.95	0.92	0.98	1.00	1.00	0.99
12	0.51	0.65	0.67	0.75	0.89	0.95	0.96	0.96	1.00	1.00
13	0.07	0.20	0.35	0.49	0.63	0.75	0.92	0.90	0.98	0.97
14	0.10	0.09	0.27	0.33	0.34	0.52	0.68	0.78	0.86	0.92
15	0.04	0.09	0.16	0.29	0.34	0.42	0.49	0.60	0.52	0.66
16	0.80	0.92	0.95	0.99	0.99	1.00	1.00	1.00	1.00	1.00
17	0.50	0.50	0.61	0.79	0.83	0.91	0.96	0.96	0.99	0.99
18	0.36	0.40	0.40	0.57	0.53	0.52	0.56	0.66	0.66	0.76
19	0.14	0.21	0.38	0.50	0.59	0.72	0.92	0.92	0.99	0.98
20	0.12	0.23	0.32	0.50	0.52	0.66	0.83	0.84	0.92	0.99
21	0.09	0.09	0.25	0.29	0.43	0.46	0.61	0.72	0.75	0.86
22	0.26	0.36	0.59	0.72	0.82	0.94	0.97	1.00	0.99	1.00
23	0.17	0.30	0.43	0.59	0.74	0.87	0.93	0.95	0.97	0.96
24	0.16	0.18	0.30	0.38	0.43	0.62	0.57	0.81	0.74	0.80
25	0.13	0.29	0.36	0.50	0.76	0.80	0.86	0.92	1.00	1.00
26	0.04	0.02	0.08	0.11	0.17	0.24	0.34	0.38	0.42	0.55
27	0.04	0.04	0.15	0.17	0.18	0.30	0.26	0.30	0.32	0.30
28	0.21	0.41	0.58	0.85	0.89	0.95	0.98	1.00	1.00	0.99
29	0.02	0.07	0.06	0.15	0.27	0.24	0.31	0.38	0.50	0.53
30	0.09	0.07	0.17	0.19	0.23	0.21	0.31	0.35	0.41	0.42
31	0.12	0.20	0.42	0.54	0.68	0.87	0.93	0.95	0.95	1.00
32	0.09	0.15	0.32	0.36	0.60	0.67	0.78	0.91	0.89	0.95
33	0.05	0.10	0.20	0.30	0.32	0.44	0.59	0.62	0.66	0.74
34	0.52	0.87	0.89	0.97	0.95	1.00	0.98	1.00	1.00	1.00
35	0.34	0.57	0.66	0.75	0.82	0.93	1.00	0.99	1.00	1.00
36	0.26	0.37	0.56	0.51	0.61	0.63	0.74	0.87	0.89	0.91
37	0.11	0.25	0.43	0.67	0.80	0.90	0.91	0.95	0.97	0.99
38	0.10	0.15	0.36	0.52	0.64	0.83	0.91	0.97	0.98	0.99
39	0.06	0.07	0.19	0.36	0.50	0.64	0.85	0.83	0.92	0.99
40	0.19	0.45	0.51	0.70	0.86	0.90	0.95	1.00	0.96	0.99
41	0.13	0.21	0.47	0.51	0.63	0.84	0.94	0.96	1.00	1.00
42	0.11	0.16	0.28	0.44	0.59	0.68	0.86	0.91	0.96	0.98

Table 15: POD according to each scenario and each k2 value, k1 = 2, alpha = 0.05

	k2=1	k2=2	k2=3	k2=4	k2=5	k2=6	k2=7	k2=8	k2=9	k2=10
1	0.17	0.16	0.25	0.52	0.62	0.74	0.76	0.91	0.90	0.96
2	0.10	0.20	0.22	0.31	0.47	0.47	0.63	0.72	0.77	0.87
3	0.06	0.17	0.20	0.16	0.32	0.42	0.41	0.44	0.54	0.58
4	0.44	0.57	0.61	0.81	0.90	0.94	0.98	0.98	0.99	0.99
5	0.18	0.32	0.34	0.49	0.60	0.70	0.83	0.91	0.93	0.94
6	0.22	0.27	0.28	0.35	0.34	0.47	0.44	0.45	0.52	0.59
7	0.37	0.45	0.53	0.60	0.57	0.69	0.81	0.78	0.81	0.93
8	0.34	0.42	0.53	0.52	0.58	0.70	0.81	0.73	0.74	0.82
9	0.41	0.52	0.55	0.51	0.58	0.68	0.70	0.82	0.84	0.88
10	0.44	0.57	0.79	0.87	0.95	0.98	0.96	1.00	0.99	1.00
11	0.50	0.66	0.75	0.95	0.91	0.99	0.96	1.00	1.00	0.99
12	0.43	0.54	0.65	0.83	0.90	0.94	0.95	0.98	0.98	0.99
13	0.12	0.18	0.35	0.42	0.69	0.74	0.79	0.95	0.95	0.99
14	0.05	0.11	0.18	0.32	0.51	0.54	0.67	0.85	0.85	0.87
15	0.05	0.05	0.15	0.27	0.32	0.46	0.48	0.58	0.63	0.64
16	0.79	0.89	0.95	0.98	0.96	1.00	1.00	1.00	1.00	1.00
17	0.46	0.50	0.69	0.78	0.81	0.87	0.91	0.96	1.00	0.98
18	0.41	0.41	0.46	0.41	0.50	0.59	0.65	0.68	0.65	0.72
19	0.11	0.29	0.34	0.46	0.60	0.72	0.86	0.91	0.96	0.96
20	0.11	0.25	0.24	0.36	0.52	0.66	0.79	0.89	0.93	0.99
21	0.04	0.13	0.17	0.29	0.40	0.48	0.60	0.70	0.75	0.86
22	0.20	0.41	0.52	0.74	0.85	0.93	0.96	0.97	0.98	0.98
23	0.11	0.25	0.47	0.46	0.75	0.81	0.92	0.95	0.97	0.99
24	0.12	0.25	0.25	0.40	0.54	0.55	0.57	0.66	0.69	0.85
25	0.12	0.16	0.34	0.57	0.73	0.80	0.87	0.98	0.99	0.99
26	0.01	0.06	0.00	0.09	0.12	0.22	0.27	0.35	0.48	0.63
27	0.03	0.03	0.20	0.12	0.18	0.25	0.26	0.30	0.33	0.36
28	0.19	0.49	0.56	0.68	0.84	0.95	0.99	1.00	1.00	1.00
29	0.04	0.05	0.08	0.09	0.23	0.15	0.26	0.31	0.52	0.50
30	0.09	0.12	0.14	0.19	0.15	0.23	0.30	0.34	0.35	0.34
31	0.08	0.24	0.38	0.57	0.59	0.89	0.88	0.96	0.96	1.00
32	0.04	0.07	0.20	0.35	0.52	0.63	0.82	0.92	0.92	0.99
33	0.02	0.09	0.22	0.25	0.37	0.51	0.57	0.63	0.73	0.84
34	0.44	0.68	0.81	0.88	0.96	1.00	0.99	1.00	1.00	1.00
35	0.28	0.51	0.64	0.76	0.88	0.92	0.98	0.99	1.00	1.00
36	0.32	0.37	0.51	0.59	0.48	0.67	0.77	0.85	0.89	0.95
37	0.16	0.28	0.48	0.53	0.75	0.86	0.92	0.93	0.99	1.00
38	0.09	0.14	0.26	0.53	0.62	0.77	0.87	0.91	0.97	0.99
39	0.05	0.15	0.23	0.32	0.48	0.63	0.81	0.83	0.95	0.96
40	0.20	0.41	0.48	0.71	0.85	0.92	0.98	0.97	0.99	1.00
41	0.13	0.27	0.35	0.52	0.71	0.87	0.97	0.99	0.98	0.98
42	0.11	0.25	0.26	0.40	0.64	0.74	0.86	0.89	0.97	0.97

Table 16: POD according to each scenario and each k2 value, k1 = 3, alpha = 0.05

	k2=1	k2=2	k2=3	k2=4	k2=5	k2=6	k2=7	k2=8	k2=9	k2=10
1	0.16	0.22	0.32	0.33	0.53	0.54	0.73	0.88	0.90	0.91
2	0.10	0.14	0.20	0.28	0.37	0.54	0.61	0.71	0.79	0.80
3	0.07	0.13	0.19	0.29	0.29	0.26	0.38	0.45	0.54	0.53
4	0.36	0.52	0.61	0.76	0.85	0.87	0.97	0.97	0.98	1.00
5	0.18	0.26	0.39	0.38	0.51	0.71	0.77	0.88	0.91	0.88
6	0.16	0.31	0.29	0.35	0.36	0.39	0.47	0.52	0.56	0.53
7	0.30	0.32	0.31	0.58	0.63	0.63	0.69	0.81	0.79	0.87
8	0.38	0.43	0.44	0.52	0.63	0.62	0.72	0.76	0.76	0.88
9	0.36	0.39	0.49	0.50	0.60	0.66	0.70	0.80	0.85	0.81
10	0.43	0.58	0.73	0.85	0.95	0.94	0.98	0.98	1.00	1.00
11	0.43	0.52	0.70	0.84	0.92	0.93	0.98	1.00	1.00	1.00
12	0.40	0.55	0.63	0.73	0.87	0.91	0.92	0.95	0.93	1.00
13	0.05	0.19	0.34	0.49	0.61	0.72	0.77	0.88	0.94	0.95
14	0.02	0.11	0.22	0.27	0.48	0.55	0.69	0.82	0.84	0.92
15	0.05	0.08	0.13	0.29	0.29	0.28	0.43	0.61	0.65	0.71
16	0.72	0.84	0.93	0.98	0.98	1.00	1.00	1.00	1.00	1.00
17	0.36	0.44	0.60	0.68	0.85	0.92	0.92	0.94	0.99	0.98
18	0.38	0.35	0.53	0.41	0.50	0.55	0.63	0.62	0.63	0.76
19	0.12	0.19	0.29	0.39	0.45	0.66	0.84	0.87	0.92	0.94
20	0.09	0.09	0.21	0.37	0.49	0.60	0.83	0.81	0.93	0.94
21	0.11	0.14	0.14	0.24	0.38	0.49	0.57	0.63	0.70	0.76
22	0.14	0.37	0.49	0.69	0.72	0.87	0.93	0.98	1.00	1.00
23	0.15	0.22	0.43	0.49	0.67	0.74	0.86	0.95	0.98	0.99
24	0.17	0.21	0.18	0.28	0.47	0.52	0.52	0.63	0.71	0.84
25	0.10	0.21	0.27	0.47	0.60	0.74	0.82	0.95	0.96	0.99
26	0.00	0.04	0.03	0.10	0.18	0.17	0.25	0.37	0.41	0.54
27	0.05	0.06	0.12	0.14	0.20	0.20	0.32	0.33	0.25	0.33
28	0.24	0.36	0.45	0.63	0.77	0.85	0.92	0.96	0.96	1.00
29	0.04	0.04	0.08	0.08	0.16	0.24	0.34	0.31	0.45	0.48
30	0.08	0.09	0.20	0.18	0.25	0.20	0.29	0.37	0.34	0.40
31	0.15	0.19	0.40	0.40	0.56	0.73	0.84	0.95	0.95	0.98
32	0.05	0.11	0.20	0.42	0.44	0.60	0.74	0.82	0.96	0.96
33	0.03	0.11	0.11	0.14	0.33	0.35	0.55	0.56	0.73	0.74
34	0.45	0.64	0.81	0.86	0.93	0.95	0.98	1.00	0.99	1.00
35	0.30	0.43	0.61	0.72	0.89	0.98	0.96	0.99	0.99	1.00
36	0.28	0.33	0.46	0.60	0.60	0.70	0.68	0.77	0.80	0.91
37	0.06	0.12	0.34	0.47	0.60	0.82	0.85	0.95	0.95	0.99
38	0.03	0.10	0.23	0.31	0.54	0.75	0.81	0.93	0.98	0.98
39	0.05	0.07	0.22	0.27	0.39	0.57	0.70	0.77	0.84	0.93
40	0.19	0.23	0.54	0.61	0.78	0.86	0.96	0.97	1.00	0.99
41	0.15	0.16	0.28	0.52	0.55	0.79	0.93	0.96	0.99	0.99
42	0.08	0.11	0.27	0.36	0.55	0.69	0.81	0.87	0.85	0.96

Table 17: POD according to each scenario and each k2 value, k1 = 5, alpha = 0.05

	k2=1	k2=2	k2=3	k2=4	k2=5	k2=6	k2=7	k2=8	k2=9	k2=10
1	0.16	0.11	0.15	0.29	0.33	0.43	0.60	0.58	0.71	0.82
2	0.06	0.10	0.14	0.14	0.28	0.39	0.49	0.53	0.67	0.69
3	0.08	0.10	0.13	0.18	0.15	0.31	0.30	0.38	0.49	0.48
4	0.20	0.29	0.42	0.57	0.62	0.73	0.87	0.91	0.96	0.97
5	0.09	0.14	0.26	0.35	0.46	0.53	0.69	0.76	0.79	0.93
6	0.15	0.14	0.20	0.28	0.28	0.38	0.45	0.35	0.52	0.54
7	0.25	0.25	0.40	0.41	0.42	0.41	0.57	0.60	0.73	0.72
8	0.17	0.31	0.27	0.46	0.57	0.52	0.69	0.61	0.70	0.77
9	0.24	0.31	0.31	0.38	0.49	0.47	0.52	0.65	0.67	0.72
10	0.25	0.46	0.55	0.59	0.86	0.89	0.93	0.93	0.95	0.99
11	0.33	0.43	0.59	0.65	0.78	0.86	0.92	0.96	0.97	0.98
12	0.25	0.41	0.48	0.66	0.75	0.86	0.95	0.94	0.98	0.97
13	0.06	0.11	0.10	0.30	0.33	0.49	0.54	0.73	0.80	0.87
14	0.04	0.03	0.13	0.19	0.31	0.31	0.47	0.60	0.79	0.76
15	0.04	0.05	0.17	0.12	0.16	0.33	0.33	0.40	0.51	0.62
16	0.50	0.73	0.76	0.90	0.95	0.93	0.99	1.00	1.00	1.00
17	0.38	0.32	0.50	0.65	0.72	0.84	0.90	0.91	0.92	0.97
18	0.38	0.32	0.42	0.49	0.46	0.46	0.60	0.66	0.63	0.65
19	0.05	0.10	0.12	0.26	0.43	0.57	0.60	0.69	0.85	0.88
20	0.04	0.07	0.10	0.31	0.28	0.36	0.48	0.66	0.74	0.86
21	0.07	0.08	0.09	0.15	0.22	0.26	0.38	0.44	0.61	0.71
22	0.11	0.24	0.32	0.44	0.55	0.67	0.86	0.89	0.94	0.94
23	0.06	0.14	0.24	0.38	0.50	0.68	0.74	0.85	0.91	0.91
24	0.08	0.08	0.21	0.35	0.37	0.39	0.56	0.55	0.65	0.66
25	0.06	0.10	0.16	0.29	0.45	0.56	0.67	0.79	0.81	0.94
26	0.02	0.01	0.03	0.07	0.05	0.10	0.23	0.27	0.28	0.48
27	0.02	0.05	0.10	0.09	0.10	0.07	0.19	0.31	0.26	0.32
28	0.12	0.27	0.25	0.48	0.64	0.69	0.83	0.92	0.91	0.96
29	0.02	0.07	0.10	0.12	0.10	0.20	0.25	0.32	0.33	0.42
30	0.04	0.10	0.09	0.23	0.22	0.23	0.27	0.32	0.31	0.32
31	0.06	0.12	0.23	0.30	0.42	0.41	0.64	0.82	0.88	0.98
32	0.02	0.05	0.12	0.16	0.27	0.40	0.53	0.71	0.81	0.80
33	0.01	0.06	0.07	0.21	0.24	0.27	0.38	0.52	0.54	0.63
34	0.25	0.56	0.57	0.76	0.81	0.91	0.94	0.97	0.99	1.00
35	0.19	0.22	0.45	0.53	0.65	0.80	0.81	0.96	0.98	0.97
36	0.24	0.27	0.40	0.44	0.48	0.53	0.61	0.71	0.75	0.83
37	0.02	0.09	0.19	0.35	0.46	0.57	0.60	0.78	0.82	0.92
38	0.02	0.07	0.10	0.20	0.36	0.50	0.68	0.84	0.84	0.94
39	0.02	0.04	0.07	0.14	0.29	0.44	0.47	0.61	0.74	0.79
40	0.09	0.12	0.21	0.40	0.52	0.55	0.77	0.84	0.95	0.96
41	0.04	0.10	0.17	0.31	0.48	0.39	0.67	0.78	0.87	0.96
42	0.03	0.07	0.15	0.33	0.35	0.53	0.62	0.77	0.82	0.89

Table 18: POD according to each scenario and each k2 value, k1 = 10, alpha = 0.05
