# Supplementary material for: Evaluation and comparison of statistical methods for early temporal detection of outbreaks: A simulation-based study
Source: PLoS One. 2017 Jul 17;12(7):e0181227. doi: 10.1371/journal.pone.0181227 (PMC5513450; doi:10.1371/journal.pone.0181227)
Supplement: S7 Appendix — (PDF) [file pone.0181227.s007.pdf]

Evaluation and Comparison of Statistical Methods for Early Temporal Detection of Outbreaks: a Simulation-Based Study

Appendix S7: Overall performances of CUSUM algorithm ($\alpha = 0.001, 0.01$ and 0.05)

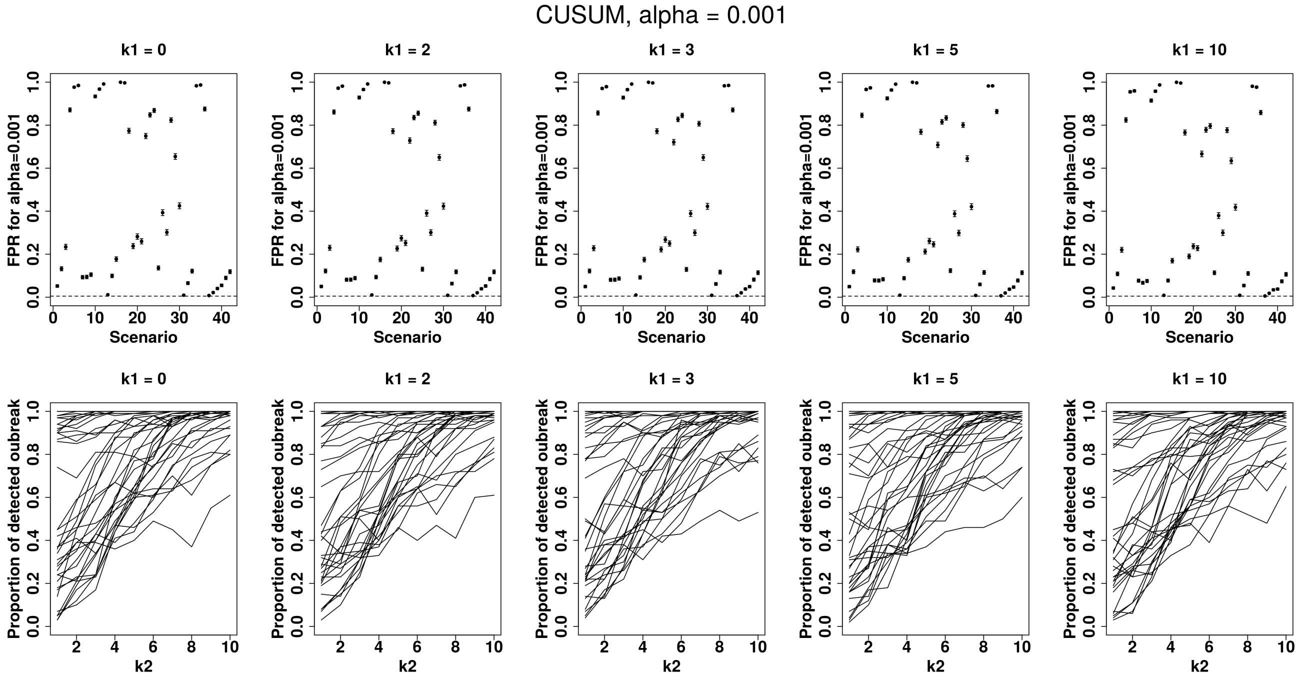

Figure 1: CUSUM algorithm performances for $\alpha = 0.001$ by increasing past outbreak amplitude $k_1 = 0$, 2, 3, 5 or 10 with (i) on the first row: false positive rate for 42 simulated scenarios, (ii) on the second row: probability of detection for 42 simulated scenarios (each curve corresponding to a scenario) by increasing current outbreak amplitude $k_2 = 1$ to 10.

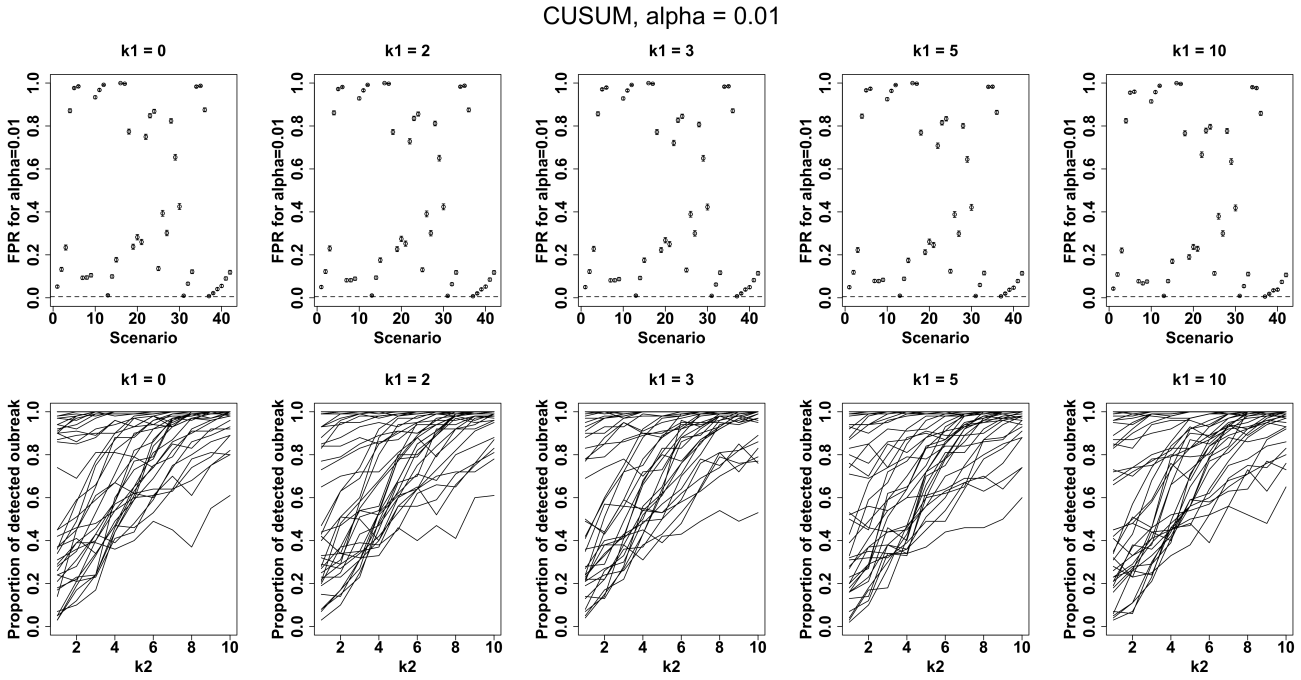

Figure 2: CUSUM algorithm performances for $\alpha = 0.01$ by increasing past outbreak amplitude $k_1 = 0$, 2, 3, 5 or 10 with (i) on the first row: false positive rate for 42 simulated scenarios, (ii) on the second row: probability of detection for 42 simulated scenarios (each curve corresponding to a scenario) by increasing current outbreak amplitude $k_2 = 1$ to 10.

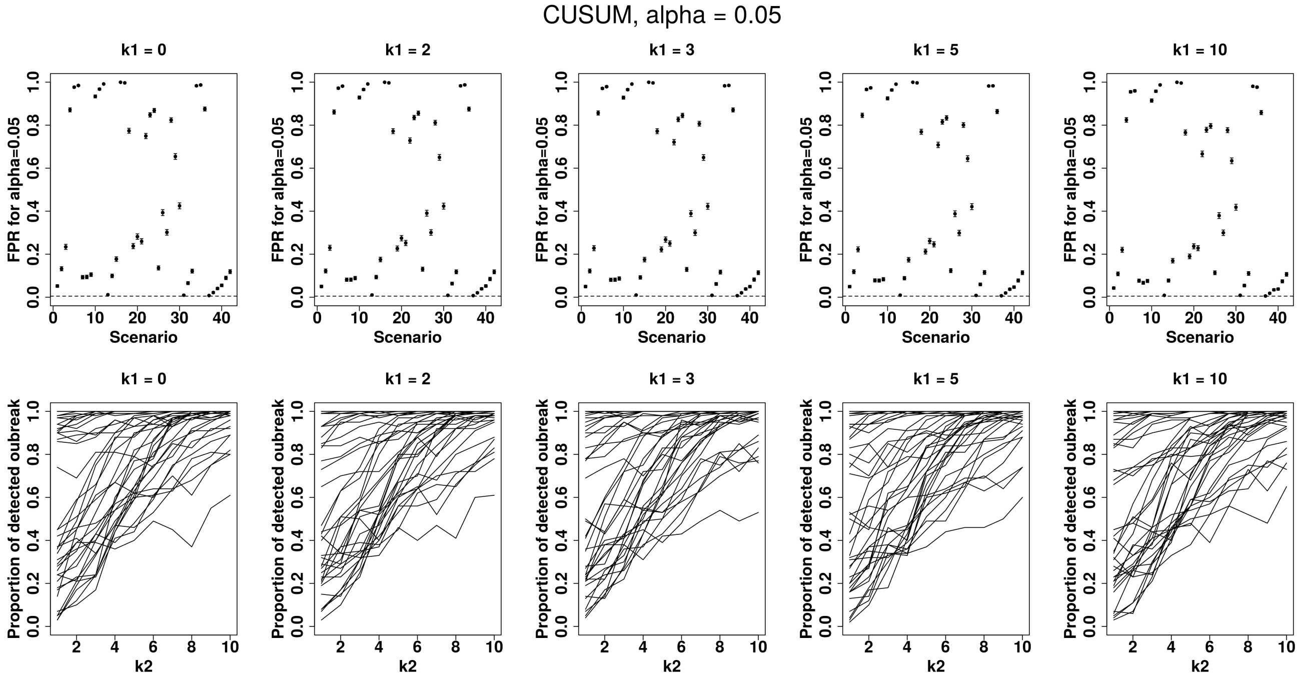

Figure 3: CUSUM algorithm performances for $\alpha = 0.05$ by increasing past outbreak amplitude $k_1 = 0$, 2, 3, 5 or 10 with (i) on the first row: false positive rate for 42 simulated scenarios, (ii) on the second row: probability of detection for 42 simulated scenarios (each curve corresponding to a scenario) by increasing current outbreak amplitude $k_2 = 1$ to 10.

Overall performances of CUSUM algorithm ($\alpha = 0.001$)

	FPR k1=0	FPR k1=2	FPR k1=3	FPR k1=5	FPR k1=10
1	0.05	0.05	0.05	0.05	0.04
2	0.13	0.12	0.12	0.12	0.11
3	0.23	0.23	0.23	0.22	0.22
4	0.87	0.86	0.86	0.85	0.82
5	0.98	0.97	0.97	0.97	0.96
6	0.98	0.98	0.98	0.97	0.96
7	0.09	0.08	0.08	0.08	0.08
8	0.09	0.08	0.08	0.08	0.07
9	0.10	0.09	0.09	0.08	0.08
10	0.93	0.93	0.93	0.92	0.91
11	0.97	0.97	0.97	0.96	0.96
12	0.99	0.99	0.99	0.99	0.99
13	0.01	0.01	0.01	0.01	0.01
14	0.10	0.09	0.09	0.09	0.08
15	0.18	0.18	0.17	0.17	0.17
16	1.00	1.00	1.00	1.00	1.00
17	1.00	1.00	1.00	1.00	1.00
18	0.77	0.77	0.77	0.77	0.77
19	0.24	0.23	0.22	0.21	0.19
20	0.28	0.27	0.27	0.26	0.24
21	0.26	0.25	0.25	0.25	0.23
22	0.75	0.73	0.72	0.71	0.67
23	0.85	0.84	0.83	0.82	0.78
24	0.87	0.86	0.85	0.83	0.80
25	0.14	0.13	0.13	0.12	0.11
26	0.39	0.39	0.39	0.39	0.38
27	0.30	0.30	0.30	0.30	0.30
28	0.82	0.81	0.81	0.80	0.78
29	0.65	0.65	0.65	0.64	0.63
30	0.42	0.42	0.42	0.42	0.42
31	0.01	0.01	0.01	0.01	0.01
32	0.07	0.06	0.06	0.06	0.05
33	0.12	0.12	0.12	0.12	0.11
34	0.98	0.98	0.98	0.98	0.98
35	0.99	0.99	0.98	0.98	0.98
36	0.88	0.87	0.87	0.86	0.86
37	0.01	0.01	0.01	0.01	0.01
38	0.02	0.02	0.02	0.02	0.02
39	0.04	0.04	0.04	0.04	0.03
40	0.06	0.05	0.05	0.05	0.04
41	0.09	0.08	0.08	0.08	0.07
42	0.12	0.12	0.11	0.11	0.11

Table 1: FPR according to each scenario and each k1 value, alpha = 0.001

	k2=1	k2=2	k2=3	k2=4	k2=5	k2=6	k2=7	k2=8	k2=9	k2=10
1	0.29	0.39	0.46	0.58	0.77	0.79	0.97	0.96	1.00	0.98
2	0.31	0.36	0.40	0.57	0.62	0.60	0.74	0.86	0.89	0.93
3	0.37	0.41	0.34	0.47	0.46	0.53	0.64	0.68	0.75	0.82
4	0.94	0.98	1.00	1.00	1.00	1.00	1.00	1.00	1.00	1.00
5	0.98	0.99	0.98	1.00	1.00	1.00	1.00	1.00	1.00	1.00
6	0.98	1.00	1.00	0.98	0.99	0.99	1.00	1.00	1.00	1.00
7	0.90	0.86	0.92	0.94	0.97	0.94	0.93	0.97	0.97	0.99
8	0.90	0.91	0.89	0.96	0.93	0.98	0.93	0.97	0.96	0.98
9	0.87	0.89	0.90	0.90	0.93	0.98	0.95	0.99	0.97	1.00
10	0.97	0.98	1.00	1.00	1.00	1.00	1.00	1.00	1.00	1.00
11	1.00	1.00	1.00	1.00	1.00	1.00	1.00	1.00	1.00	1.00
12	1.00	1.00	1.00	1.00	1.00	1.00	1.00	1.00	1.00	1.00
13	0.05	0.12	0.24	0.46	0.63	0.75	0.79	0.92	0.96	0.98
14	0.24	0.28	0.43	0.39	0.48	0.59	0.75	0.80	0.86	0.89
15	0.24	0.21	0.23	0.41	0.44	0.61	0.63	0.74	0.79	0.89
16	1.00	1.00	1.00	1.00	1.00	1.00	1.00	1.00	1.00	1.00
17	1.00	1.00	1.00	1.00	1.00	1.00	1.00	1.00	1.00	1.00
18	0.86	0.85	0.88	0.96	0.93	0.94	0.99	0.99	1.00	1.00
19	0.36	0.58	0.77	0.90	0.98	0.99	1.00	1.00	1.00	1.00
20	0.45	0.59	0.66	0.82	0.89	0.96	0.98	0.98	1.00	1.00
21	0.37	0.45	0.61	0.67	0.79	0.92	0.94	0.99	0.99	0.99
22	0.90	0.98	1.00	1.00	1.00	1.00	1.00	1.00	1.00	1.00
23	0.94	0.92	0.97	1.00	1.00	1.00	1.00	1.00	1.00	1.00
24	0.91	0.97	0.99	0.99	0.99	1.00	1.00	1.00	1.00	1.00
25	0.34	0.49	0.75	0.87	0.97	0.98	1.00	1.00	1.00	1.00
26	0.45	0.51	0.59	0.67	0.62	0.64	0.64	0.78	0.81	0.80
27	0.26	0.39	0.39	0.36	0.40	0.49	0.45	0.37	0.55	0.61
28	0.97	0.96	1.00	1.00	1.00	1.00	1.00	1.00	1.00	1.00
29	0.74	0.69	0.81	0.81	0.79	0.74	0.85	0.83	0.92	0.92
30	0.42	0.46	0.48	0.54	0.60	0.61	0.70	0.61	0.75	0.80
31	0.07	0.10	0.17	0.44	0.61	0.87	0.96	0.99	0.99	1.00
32	0.18	0.22	0.34	0.47	0.65	0.74	0.86	0.90	0.95	1.00
33	0.17	0.23	0.24	0.44	0.59	0.69	0.82	0.91	0.91	0.96
34	1.00	1.00	1.00	1.00	1.00	1.00	1.00	1.00	1.00	1.00
35	1.00	1.00	1.00	1.00	1.00	1.00	1.00	1.00	1.00	1.00
36	0.92	0.94	0.99	0.99	0.99	1.00	1.00	1.00	1.00	1.00
37	0.03	0.17	0.24	0.60	0.73	0.78	0.96	0.99	0.99	1.00
38	0.03	0.19	0.33	0.49	0.59	0.84	0.93	1.00	1.00	1.00
39	0.05	0.21	0.40	0.54	0.65	0.81	0.87	0.97	0.99	1.00
40	0.14	0.45	0.65	0.88	0.88	0.96	0.99	1.00	1.00	1.00
41	0.28	0.35	0.53	0.71	0.90	0.92	0.97	0.99	0.99	1.00
42	0.21	0.31	0.43	0.58	0.82	0.86	0.97	0.98	1.00	1.00

Table 2: POD according to each scenario and each k2 value, k1 = 0, alpha = 0.001

	k2=1	k2=2	k2=3	k2=4	k2=5	k2=6	k2=7	k2=8	k2=9	k2=10
1	0.20	0.37	0.54	0.56	0.80	0.80	0.89	0.97	0.95	0.96
2	0.33	0.34	0.35	0.53	0.62	0.71	0.68	0.80	0.84	0.88
3	0.27	0.37	0.39	0.38	0.45	0.62	0.66	0.65	0.76	0.81
4	0.99	1.00	1.00	1.00	1.00	1.00	1.00	1.00	1.00	1.00
5	0.99	1.00	1.00	0.99	1.00	1.00	1.00	1.00	1.00	1.00
6	0.99	0.99	1.00	1.00	0.98	1.00	1.00	0.99	1.00	1.00
7	0.77	0.79	0.85	0.89	0.91	0.90	0.96	0.96	0.98	0.97
8	0.84	0.84	0.88	0.85	0.91	0.90	0.94	0.94	0.98	0.98
9	0.73	0.78	0.83	0.86	0.87	0.94	0.94	0.98	0.96	0.99
10	0.99	1.00	1.00	1.00	1.00	1.00	1.00	1.00	1.00	1.00
11	0.99	0.99	1.00	1.00	1.00	1.00	1.00	1.00	1.00	1.00
12	1.00	1.00	1.00	1.00	1.00	1.00	1.00	1.00	1.00	1.00
13	0.03	0.10	0.25	0.45	0.67	0.70	0.92	0.92	0.98	0.98
14	0.29	0.27	0.34	0.42	0.53	0.64	0.73	0.82	0.91	0.95
15	0.32	0.29	0.35	0.37	0.56	0.56	0.59	0.71	0.76	0.87
16	1.00	1.00	1.00	1.00	1.00	1.00	1.00	1.00	1.00	1.00
17	1.00	1.00	1.00	1.00	1.00	1.00	1.00	1.00	1.00	1.00
18	0.85	0.88	0.93	0.95	0.93	0.98	0.99	0.98	1.00	1.00
19	0.43	0.63	0.74	0.92	0.98	0.99	1.00	1.00	1.00	1.00
20	0.47	0.63	0.69	0.85	0.90	0.95	0.96	1.00	1.00	1.00
21	0.42	0.34	0.55	0.67	0.77	0.88	0.94	0.98	0.99	0.99
22	0.88	0.96	0.99	1.00	1.00	1.00	1.00	1.00	1.00	1.00
23	0.93	0.94	0.98	0.98	0.99	1.00	1.00	1.00	1.00	1.00
24	0.83	0.95	0.95	0.98	0.99	0.99	1.00	1.00	1.00	1.00
25	0.28	0.51	0.60	0.88	0.97	0.97	1.00	1.00	1.00	1.00
26	0.44	0.47	0.54	0.52	0.61	0.64	0.70	0.72	0.72	0.78
27	0.42	0.34	0.32	0.33	0.46	0.40	0.47	0.41	0.60	0.61
28	0.93	0.97	0.99	1.00	1.00	1.00	1.00	1.00	1.00	1.00
29	0.65	0.70	0.72	0.72	0.81	0.88	0.79	0.92	0.92	0.96
30	0.40	0.51	0.50	0.40	0.60	0.65	0.52	0.66	0.75	0.83
31	0.08	0.15	0.23	0.49	0.68	0.85	0.91	0.99	0.99	1.00
32	0.19	0.25	0.43	0.47	0.60	0.75	0.84	0.92	0.94	0.99
33	0.23	0.23	0.31	0.40	0.56	0.64	0.80	0.88	0.97	0.98
34	1.00	1.00	1.00	1.00	1.00	1.00	1.00	1.00	1.00	1.00
35	0.99	1.00	1.00	1.00	1.00	1.00	1.00	1.00	1.00	1.00
36	0.93	0.99	0.99	0.97	0.99	0.99	1.00	1.00	1.00	1.00
37	0.07	0.17	0.31	0.53	0.78	0.79	0.90	0.99	0.99	1.00
38	0.08	0.17	0.27	0.53	0.68	0.85	0.91	0.98	1.00	1.00
39	0.15	0.14	0.31	0.48	0.63	0.82	0.89	0.99	0.99	1.00
40	0.21	0.48	0.58	0.80	0.94	0.95	0.99	1.00	0.99	1.00
41	0.22	0.29	0.58	0.68	0.79	0.95	0.99	0.99	1.00	1.00
42	0.25	0.31	0.51	0.54	0.78	0.89	0.97	0.98	1.00	1.00

Table 3: POD according to each scenario and each k2 value, k1 = 2, alpha = 0.001

	k2=1	k2=2	k2=3	k2=4	k2=5	k2=6	k2=7	k2=8	k2=9	k2=10
1	0.28	0.29	0.49	0.67	0.83	0.83	0.92	0.98	0.95	0.97
2	0.24	0.33	0.43	0.48	0.57	0.64	0.78	0.75	0.82	0.86
3	0.23	0.34	0.42	0.45	0.39	0.48	0.65	0.81	0.72	0.83
4	0.97	0.97	0.98	1.00	1.00	1.00	1.00	1.00	1.00	1.00
5	1.00	1.00	0.99	1.00	1.00	1.00	1.00	1.00	1.00	1.00
6	1.00	1.00	0.97	1.00	1.00	1.00	1.00	1.00	1.00	1.00
7	0.78	0.79	0.87	0.90	0.90	0.95	0.95	0.98	0.95	0.99
8	0.77	0.88	0.95	0.90	0.88	0.94	0.94	0.96	0.98	0.96
9	0.78	0.85	0.86	0.89	0.91	0.92	0.95	0.95	0.94	0.97
10	0.98	1.00	1.00	1.00	1.00	1.00	1.00	1.00	1.00	1.00
11	1.00	1.00	1.00	1.00	1.00	1.00	1.00	1.00	1.00	1.00
12	1.00	1.00	1.00	1.00	1.00	1.00	1.00	1.00	1.00	1.00
13	0.04	0.15	0.27	0.43	0.64	0.76	0.87	0.93	0.99	1.00
14	0.22	0.22	0.36	0.37	0.50	0.59	0.76	0.81	0.82	0.89
15	0.19	0.24	0.26	0.38	0.43	0.53	0.64	0.79	0.76	0.77
16	1.00	1.00	1.00	1.00	1.00	1.00	1.00	1.00	1.00	1.00
17	1.00	1.00	1.00	1.00	1.00	1.00	1.00	1.00	1.00	1.00
18	0.83	0.85	0.95	0.91	0.95	0.97	0.99	1.00	1.00	1.00
19	0.41	0.60	0.75	0.92	0.98	0.98	1.00	0.99	1.00	1.00
20	0.42	0.59	0.72	0.75	0.89	0.93	0.99	1.00	1.00	1.00
21	0.35	0.57	0.56	0.72	0.73	0.88	0.89	0.98	0.98	1.00
22	0.91	0.97	0.99	1.00	1.00	1.00	1.00	1.00	1.00	1.00
23	0.90	0.90	0.97	0.98	0.98	1.00	1.00	1.00	1.00	1.00
24	0.88	0.93	0.93	0.99	0.98	0.99	1.00	1.00	1.00	1.00
25	0.24	0.51	0.72	0.88	0.97	0.98	1.00	1.00	1.00	1.00
26	0.50	0.43	0.56	0.55	0.53	0.62	0.69	0.74	0.85	0.76
27	0.36	0.38	0.38	0.31	0.42	0.43	0.49	0.54	0.49	0.53
28	0.95	0.98	0.98	1.00	1.00	1.00	1.00	1.00	1.00	1.00
29	0.69	0.74	0.78	0.70	0.80	0.77	0.87	0.92	0.97	0.95
30	0.49	0.44	0.59	0.54	0.53	0.58	0.63	0.70	0.75	0.79
31	0.05	0.17	0.22	0.43	0.62	0.96	0.93	0.99	0.99	1.00
32	0.13	0.22	0.38	0.54	0.62	0.71	0.86	0.92	0.97	1.00
33	0.21	0.27	0.30	0.49	0.50	0.70	0.80	0.94	0.96	0.99
34	0.99	1.00	1.00	1.00	1.00	1.00	1.00	1.00	1.00	1.00
35	1.00	0.99	1.00	1.00	1.00	1.00	1.00	1.00	1.00	1.00
36	0.93	0.97	0.99	1.00	1.00	1.00	1.00	1.00	1.00	1.00
37	0.07	0.14	0.41	0.50	0.71	0.88	0.93	0.98	0.98	1.00
38	0.14	0.13	0.28	0.56	0.65	0.86	0.90	0.93	0.99	1.00
39	0.08	0.26	0.33	0.52	0.61	0.76	0.85	0.96	1.00	1.00
40	0.21	0.44	0.59	0.78	0.88	0.99	0.99	0.99	1.00	1.00
41	0.22	0.38	0.41	0.68	0.81	0.92	0.98	0.99	1.00	1.00
42	0.27	0.40	0.42	0.66	0.78	0.89	0.99	0.99	1.00	1.00

Table 4: POD according to each scenario and each k2 value, k1 = 3, alpha = 0.001

	k2=1	k2=2	k2=3	k2=4	k2=5	k2=6	k2=7	k2=8	k2=9	k2=10
1	0.27	0.41	0.54	0.60	0.72	0.78	0.92	1.00	0.99	0.97
2	0.20	0.37	0.44	0.47	0.56	0.70	0.74	0.80	0.85	0.88
3	0.28	0.37	0.38	0.36	0.45	0.54	0.67	0.70	0.64	0.74
4	0.94	0.94	0.99	1.00	1.00	1.00	1.00	1.00	1.00	1.00
5	1.00	1.00	1.00	1.00	1.00	1.00	1.00	1.00	1.00	1.00
6	0.98	0.99	1.00	1.00	0.99	0.99	1.00	1.00	1.00	1.00
7	0.74	0.85	0.74	0.88	0.90	0.87	0.94	0.95	0.98	0.99
8	0.80	0.76	0.86	0.87	0.91	0.89	0.89	0.96	0.97	0.96
9	0.76	0.70	0.85	0.81	0.88	0.91	0.93	0.95	0.96	0.94
10	0.99	0.99	0.99	1.00	1.00	1.00	1.00	1.00	1.00	1.00
11	1.00	1.00	1.00	1.00	1.00	1.00	1.00	1.00	1.00	1.00
12	1.00	1.00	1.00	1.00	1.00	1.00	1.00	1.00	1.00	1.00
13	0.04	0.18	0.30	0.43	0.66	0.81	0.81	0.92	0.97	0.98
14	0.16	0.23	0.38	0.46	0.60	0.62	0.65	0.87	0.88	0.93
15	0.31	0.32	0.32	0.39	0.49	0.49	0.64	0.78	0.83	0.91
16	1.00	1.00	1.00	1.00	1.00	1.00	1.00	1.00	1.00	1.00
17	1.00	1.00	1.00	1.00	1.00	1.00	1.00	1.00	1.00	1.00
18	0.83	0.85	0.92	0.91	0.92	0.98	0.98	0.99	1.00	1.00
19	0.43	0.67	0.80	0.92	0.98	0.98	0.99	1.00	1.00	1.00
20	0.50	0.45	0.72	0.77	0.88	0.95	0.99	0.99	1.00	1.00
21	0.39	0.49	0.59	0.64	0.83	0.85	0.95	0.97	1.00	1.00
22	0.88	0.98	1.00	1.00	1.00	1.00	1.00	1.00	1.00	1.00
23	0.87	0.94	0.97	1.00	0.99	0.99	0.99	1.00	1.00	1.00
24	0.94	0.94	0.95	0.99	0.99	1.00	0.98	0.99	1.00	1.00
25	0.33	0.59	0.67	0.85	0.96	0.99	0.99	1.00	1.00	1.00
26	0.52	0.56	0.54	0.60	0.61	0.64	0.69	0.69	0.76	0.88
27	0.32	0.35	0.34	0.34	0.37	0.44	0.46	0.46	0.50	0.60
28	0.92	0.99	1.00	0.99	1.00	1.00	1.00	1.00	1.00	1.00
29	0.71	0.69	0.74	0.79	0.78	0.84	0.84	0.86	0.87	0.96
30	0.53	0.46	0.44	0.45	0.56	0.63	0.67	0.63	0.68	0.74
31	0.07	0.12	0.34	0.33	0.63	0.79	0.92	0.96	1.00	1.00
32	0.16	0.21	0.46	0.40	0.70	0.75	0.89	0.90	0.96	0.98
33	0.16	0.29	0.33	0.36	0.51	0.60	0.75	0.87	0.94	0.98
34	0.99	1.00	1.00	1.00	1.00	1.00	1.00	1.00	1.00	1.00
35	0.99	1.00	1.00	1.00	1.00	1.00	1.00	1.00	1.00	1.00
36	0.93	0.90	0.96	0.98	0.99	1.00	1.00	1.00	1.00	1.00
37	0.02	0.10	0.27	0.47	0.72	0.85	0.94	0.98	0.97	1.00
38	0.03	0.17	0.18	0.41	0.72	0.86	0.93	0.98	1.00	1.00
39	0.13	0.14	0.38	0.34	0.60	0.78	0.86	0.97	0.99	1.00
40	0.20	0.29	0.59	0.70	0.83	0.91	0.99	0.99	1.00	1.00
41	0.23	0.27	0.62	0.65	0.75	0.93	0.98	1.00	1.00	1.00
42	0.18	0.28	0.51	0.64	0.84	0.87	0.98	0.99	1.00	1.00

Table 5: POD according to each scenario and each k2 value, k1 = 5, alpha = 0.001

	k2=1	k2=2	k2=3	k2=4	k2=5	k2=6	k2=7	k2=8	k2=9	k2=10
1	0.23	0.29	0.41	0.66	0.73	0.81	0.94	0.92	0.96	0.98
2	0.19	0.38	0.36	0.45	0.56	0.60	0.68	0.71	0.87	0.92
3	0.32	0.23	0.44	0.45	0.50	0.52	0.56	0.73	0.63	0.76
4	0.96	0.97	0.99	0.99	1.00	1.00	1.00	1.00	1.00	1.00
5	0.98	0.98	1.00	1.00	1.00	1.00	0.99	1.00	1.00	1.00
6	0.96	0.99	0.98	0.97	0.97	0.99	1.00	1.00	1.00	1.00
7	0.68	0.74	0.80	0.83	0.83	0.87	0.92	0.93	0.96	0.95
8	0.66	0.72	0.77	0.82	0.93	0.88	0.94	0.89	0.95	0.93
9	0.73	0.70	0.78	0.79	0.90	0.89	0.93	0.95	0.99	0.97
10	0.99	1.00	1.00	1.00	1.00	1.00	1.00	1.00	1.00	1.00
11	1.00	1.00	1.00	1.00	1.00	1.00	1.00	1.00	1.00	1.00
12	0.99	1.00	1.00	1.00	1.00	1.00	1.00	1.00	1.00	1.00
13	0.05	0.11	0.21	0.41	0.56	0.77	0.81	0.90	0.97	1.00
14	0.23	0.26	0.33	0.42	0.59	0.62	0.75	0.78	0.84	0.86
15	0.28	0.23	0.25	0.33	0.38	0.52	0.69	0.75	0.74	0.83
16	1.00	1.00	1.00	1.00	1.00	1.00	1.00	1.00	1.00	1.00
17	1.00	1.00	1.00	1.00	1.00	1.00	1.00	1.00	1.00	1.00
18	0.85	0.83	0.94	0.96	0.96	0.93	0.97	1.00	1.00	1.00
19	0.34	0.57	0.74	0.86	0.97	0.99	1.00	1.00	1.00	1.00
20	0.45	0.48	0.58	0.73	0.92	0.94	0.99	0.98	0.99	0.99
21	0.42	0.50	0.59	0.76	0.68	0.84	0.89	0.92	0.95	0.99
22	0.84	0.92	0.99	0.99	1.00	1.00	1.00	1.00	1.00	1.00
23	0.83	0.90	0.95	0.98	1.00	1.00	1.00	1.00	1.00	1.00
24	0.87	0.87	0.90	0.92	0.97	0.99	0.99	1.00	1.00	1.00
25	0.31	0.43	0.68	0.84	0.92	0.96	0.99	1.00	1.00	1.00
26	0.35	0.51	0.46	0.52	0.54	0.57	0.65	0.71	0.76	0.80
27	0.41	0.33	0.41	0.45	0.48	0.39	0.56	0.52	0.48	0.65
28	0.89	0.95	0.99	0.98	1.00	1.00	1.00	1.00	1.00	1.00
29	0.72	0.74	0.75	0.80	0.79	0.80	0.88	0.86	0.88	0.93
30	0.45	0.48	0.44	0.40	0.59	0.58	0.63	0.64	0.77	0.73
31	0.07	0.06	0.29	0.39	0.71	0.84	0.91	0.98	1.00	1.00
32	0.18	0.27	0.39	0.48	0.56	0.73	0.83	0.92	0.98	0.98
33	0.21	0.28	0.29	0.46	0.47	0.64	0.77	0.88	0.94	0.97
34	1.00	1.00	1.00	1.00	1.00	1.00	1.00	1.00	1.00	1.00
35	1.00	0.99	1.00	1.00	1.00	1.00	1.00	1.00	1.00	1.00
36	0.95	0.94	0.99	0.99	1.00	1.00	1.00	1.00	1.00	1.00
37	0.03	0.07	0.23	0.49	0.70	0.87	0.95	0.97	0.98	1.00
38	0.04	0.11	0.30	0.44	0.65	0.78	0.94	0.99	1.00	1.00
39	0.06	0.26	0.24	0.46	0.56	0.79	0.84	1.00	1.00	1.00
40	0.16	0.26	0.46	0.79	0.83	0.93	0.99	0.99	1.00	1.00
41	0.26	0.33	0.38	0.66	0.87	0.85	0.93	1.00	0.99	1.00
42	0.21	0.37	0.46	0.74	0.83	0.89	0.97	0.99	1.00	1.00

Table 6: POD according to each scenario and each k2 value, k1 = 10, alpha = 0.001

Overall performances of CUSUM algorithm ($\alpha = 0.01$)

	FPR k1=0	FPR k1=2	FPR k1=3	FPR k1=5	FPR k1=10
1	0.05	0.05	0.05	0.05	0.04
2	0.13	0.12	0.12	0.12	0.11
3	0.23	0.23	0.23	0.22	0.22
4	0.87	0.86	0.86	0.85	0.82
5	0.98	0.97	0.97	0.97	0.96
6	0.98	0.98	0.98	0.97	0.96
7	0.09	0.08	0.08	0.08	0.08
8	0.09	0.08	0.08	0.08	0.07
9	0.10	0.09	0.09	0.08	0.08
10	0.93	0.93	0.93	0.92	0.91
11	0.97	0.97	0.97	0.96	0.96
12	0.99	0.99	0.99	0.99	0.99
13	0.01	0.01	0.01	0.01	0.01
14	0.10	0.09	0.09	0.09	0.08
15	0.18	0.18	0.17	0.17	0.17
16	1.00	1.00	1.00	1.00	1.00
17	1.00	1.00	1.00	1.00	1.00
18	0.77	0.77	0.77	0.77	0.77
19	0.24	0.23	0.22	0.21	0.19
20	0.28	0.27	0.27	0.26	0.24
21	0.26	0.25	0.25	0.25	0.23
22	0.75	0.73	0.72	0.71	0.67
23	0.85	0.84	0.83	0.82	0.78
24	0.87	0.86	0.85	0.83	0.80
25	0.14	0.13	0.13	0.12	0.11
26	0.39	0.39	0.39	0.39	0.38
27	0.30	0.30	0.30	0.30	0.30
28	0.82	0.81	0.81	0.80	0.78
29	0.65	0.65	0.65	0.64	0.63
30	0.42	0.42	0.42	0.42	0.42
31	0.01	0.01	0.01	0.01	0.01
32	0.07	0.06	0.06	0.06	0.05
33	0.12	0.12	0.12	0.12	0.11
34	0.98	0.98	0.98	0.98	0.98
35	0.99	0.99	0.98	0.98	0.98
36	0.88	0.87	0.87	0.86	0.86
37	0.01	0.01	0.01	0.01	0.01
38	0.02	0.02	0.02	0.02	0.02
39	0.04	0.04	0.04	0.04	0.03
40	0.06	0.05	0.05	0.05	0.04
41	0.09	0.08	0.08	0.08	0.07
42	0.12	0.12	0.11	0.11	0.11

Table 7: FPR according to each scenario and each k1 value, alpha = 0.01

	k2=1	k2=2	k2=3	k2=4	k2=5	k2=6	k2=7	k2=8	k2=9	k2=10
1	0.29	0.39	0.46	0.58	0.77	0.79	0.97	0.96	1.00	0.98
2	0.31	0.36	0.40	0.57	0.62	0.60	0.74	0.86	0.89	0.93
3	0.37	0.41	0.34	0.47	0.46	0.53	0.64	0.68	0.75	0.82
4	0.94	0.98	1.00	1.00	1.00	1.00	1.00	1.00	1.00	1.00
5	0.98	0.99	0.98	1.00	1.00	1.00	1.00	1.00	1.00	1.00
6	0.98	1.00	1.00	0.98	0.99	0.99	1.00	1.00	1.00	1.00
7	0.90	0.86	0.92	0.94	0.97	0.94	0.93	0.97	0.97	0.99
8	0.90	0.91	0.89	0.96	0.93	0.98	0.93	0.97	0.96	0.98
9	0.87	0.89	0.90	0.90	0.93	0.98	0.95	0.99	0.97	1.00
10	0.97	0.98	1.00	1.00	1.00	1.00	1.00	1.00	1.00	1.00
11	1.00	1.00	1.00	1.00	1.00	1.00	1.00	1.00	1.00	1.00
12	1.00	1.00	1.00	1.00	1.00	1.00	1.00	1.00	1.00	1.00
13	0.05	0.12	0.24	0.46	0.63	0.75	0.79	0.92	0.96	0.98
14	0.24	0.28	0.43	0.39	0.48	0.59	0.75	0.80	0.86	0.89
15	0.24	0.21	0.23	0.41	0.44	0.61	0.63	0.74	0.79	0.89
16	1.00	1.00	1.00	1.00	1.00	1.00	1.00	1.00	1.00	1.00
17	1.00	1.00	1.00	1.00	1.00	1.00	1.00	1.00	1.00	1.00
18	0.86	0.85	0.88	0.96	0.93	0.94	0.99	0.99	1.00	1.00
19	0.36	0.58	0.77	0.90	0.98	0.99	1.00	1.00	1.00	1.00
20	0.45	0.59	0.66	0.82	0.89	0.96	0.98	0.98	1.00	1.00
21	0.37	0.45	0.61	0.67	0.79	0.92	0.94	0.99	0.99	0.99
22	0.90	0.98	1.00	1.00	1.00	1.00	1.00	1.00	1.00	1.00
23	0.94	0.92	0.97	1.00	1.00	1.00	1.00	1.00	1.00	1.00
24	0.91	0.97	0.99	0.99	0.99	1.00	1.00	1.00	1.00	1.00
25	0.34	0.49	0.75	0.87	0.97	0.98	1.00	1.00	1.00	1.00
26	0.45	0.51	0.59	0.67	0.62	0.64	0.64	0.78	0.81	0.80
27	0.26	0.39	0.39	0.36	0.40	0.49	0.45	0.37	0.55	0.61
28	0.97	0.96	1.00	1.00	1.00	1.00	1.00	1.00	1.00	1.00
29	0.74	0.69	0.81	0.81	0.79	0.74	0.85	0.83	0.92	0.92
30	0.42	0.46	0.48	0.54	0.60	0.61	0.70	0.61	0.75	0.80
31	0.07	0.10	0.17	0.44	0.61	0.87	0.96	0.99	0.99	1.00
32	0.18	0.22	0.34	0.47	0.65	0.74	0.86	0.90	0.95	1.00
33	0.17	0.23	0.24	0.44	0.59	0.69	0.82	0.91	0.91	0.96
34	1.00	1.00	1.00	1.00	1.00	1.00	1.00	1.00	1.00	1.00
35	1.00	1.00	1.00	1.00	1.00	1.00	1.00	1.00	1.00	1.00
36	0.92	0.94	0.99	0.99	0.99	1.00	1.00	1.00	1.00	1.00
37	0.03	0.17	0.24	0.60	0.73	0.78	0.96	0.99	0.99	1.00
38	0.03	0.19	0.33	0.49	0.59	0.84	0.93	1.00	1.00	1.00
39	0.05	0.21	0.40	0.54	0.65	0.81	0.87	0.97	0.99	1.00
40	0.14	0.45	0.65	0.88	0.88	0.96	0.99	1.00	1.00	1.00
41	0.28	0.35	0.53	0.71	0.90	0.92	0.97	0.99	0.99	1.00
42	0.21	0.31	0.43	0.58	0.82	0.86	0.97	0.98	1.00	1.00

Table 8: POD according to each scenario and each k2 value, k1 = 0, alpha = 0.01

	k2=1	k2=2	k2=3	k2=4	k2=5	k2=6	k2=7	k2=8	k2=9	k2=10
1	0.20	0.37	0.54	0.56	0.80	0.80	0.89	0.97	0.95	0.96
2	0.33	0.34	0.35	0.53	0.62	0.71	0.68	0.80	0.84	0.88
3	0.27	0.37	0.39	0.38	0.45	0.62	0.66	0.65	0.76	0.81
4	0.99	1.00	1.00	1.00	1.00	1.00	1.00	1.00	1.00	1.00
5	0.99	1.00	1.00	0.99	1.00	1.00	1.00	1.00	1.00	1.00
6	0.99	0.99	1.00	1.00	0.98	1.00	1.00	0.99	1.00	1.00
7	0.77	0.79	0.85	0.89	0.91	0.90	0.96	0.96	0.98	0.97
8	0.84	0.84	0.88	0.85	0.91	0.90	0.94	0.94	0.98	0.98
9	0.73	0.78	0.83	0.86	0.87	0.94	0.94	0.98	0.96	0.99
10	0.99	1.00	1.00	1.00	1.00	1.00	1.00	1.00	1.00	1.00
11	0.99	0.99	1.00	1.00	1.00	1.00	1.00	1.00	1.00	1.00
12	1.00	1.00	1.00	1.00	1.00	1.00	1.00	1.00	1.00	1.00
13	0.03	0.10	0.25	0.45	0.67	0.70	0.92	0.92	0.98	0.98
14	0.29	0.27	0.34	0.42	0.53	0.64	0.73	0.82	0.91	0.95
15	0.32	0.29	0.35	0.37	0.56	0.56	0.59	0.71	0.76	0.87
16	1.00	1.00	1.00	1.00	1.00	1.00	1.00	1.00	1.00	1.00
17	1.00	1.00	1.00	1.00	1.00	1.00	1.00	1.00	1.00	1.00
18	0.85	0.88	0.93	0.95	0.93	0.98	0.99	0.98	1.00	1.00
19	0.43	0.63	0.74	0.92	0.98	0.99	1.00	1.00	1.00	1.00
20	0.47	0.63	0.69	0.85	0.90	0.95	0.96	1.00	1.00	1.00
21	0.42	0.34	0.55	0.67	0.77	0.88	0.94	0.98	0.99	0.99
22	0.88	0.96	0.99	1.00	1.00	1.00	1.00	1.00	1.00	1.00
23	0.93	0.94	0.98	0.98	0.99	1.00	1.00	1.00	1.00	1.00
24	0.83	0.95	0.95	0.98	0.99	0.99	1.00	1.00	1.00	1.00
25	0.28	0.51	0.60	0.88	0.97	0.97	1.00	1.00	1.00	1.00
26	0.44	0.47	0.54	0.52	0.61	0.64	0.70	0.72	0.72	0.78
27	0.42	0.34	0.32	0.33	0.46	0.40	0.47	0.41	0.60	0.61
28	0.93	0.97	0.99	1.00	1.00	1.00	1.00	1.00	1.00	1.00
29	0.65	0.70	0.72	0.72	0.81	0.88	0.79	0.92	0.92	0.96
30	0.40	0.51	0.50	0.40	0.60	0.65	0.52	0.66	0.75	0.83
31	0.08	0.15	0.23	0.49	0.68	0.85	0.91	0.99	0.99	1.00
32	0.19	0.25	0.43	0.47	0.60	0.75	0.84	0.92	0.94	0.99
33	0.23	0.23	0.31	0.40	0.56	0.64	0.80	0.88	0.97	0.98
34	1.00	1.00	1.00	1.00	1.00	1.00	1.00	1.00	1.00	1.00
35	0.99	1.00	1.00	1.00	1.00	1.00	1.00	1.00	1.00	1.00
36	0.93	0.99	0.99	0.97	0.99	0.99	1.00	1.00	1.00	1.00
37	0.07	0.17	0.31	0.53	0.78	0.79	0.90	0.99	0.99	1.00
38	0.08	0.17	0.27	0.53	0.68	0.85	0.91	0.98	1.00	1.00
39	0.15	0.14	0.31	0.48	0.63	0.82	0.89	0.99	0.99	1.00
40	0.21	0.48	0.58	0.80	0.94	0.95	0.99	1.00	0.99	1.00
41	0.22	0.29	0.58	0.68	0.79	0.95	0.99	0.99	1.00	1.00
42	0.25	0.31	0.51	0.54	0.78	0.89	0.97	0.98	1.00	1.00

Table 9: POD according to each scenario and each k2 value, k1 = 2, alpha = 0.01

	k2=1	k2=2	k2=3	k2=4	k2=5	k2=6	k2=7	k2=8	k2=9	k2=10
1	0.28	0.29	0.49	0.67	0.83	0.83	0.92	0.98	0.95	0.97
2	0.24	0.33	0.43	0.48	0.57	0.64	0.78	0.75	0.82	0.86
3	0.23	0.34	0.42	0.45	0.39	0.48	0.65	0.81	0.72	0.83
4	0.97	0.97	0.98	1.00	1.00	1.00	1.00	1.00	1.00	1.00
5	1.00	1.00	0.99	1.00	1.00	1.00	1.00	1.00	1.00	1.00
6	1.00	1.00	0.97	1.00	1.00	1.00	1.00	1.00	1.00	1.00
7	0.78	0.79	0.87	0.90	0.90	0.95	0.95	0.98	0.95	0.99
8	0.77	0.88	0.95	0.90	0.88	0.94	0.94	0.96	0.98	0.96
9	0.78	0.85	0.86	0.89	0.91	0.92	0.95	0.95	0.94	0.97
10	0.98	1.00	1.00	1.00	1.00	1.00	1.00	1.00	1.00	1.00
11	1.00	1.00	1.00	1.00	1.00	1.00	1.00	1.00	1.00	1.00
12	1.00	1.00	1.00	1.00	1.00	1.00	1.00	1.00	1.00	1.00
13	0.04	0.15	0.27	0.43	0.64	0.76	0.87	0.93	0.99	1.00
14	0.22	0.22	0.36	0.37	0.50	0.59	0.76	0.81	0.82	0.89
15	0.19	0.24	0.26	0.38	0.43	0.53	0.64	0.79	0.76	0.77
16	1.00	1.00	1.00	1.00	1.00	1.00	1.00	1.00	1.00	1.00
17	1.00	1.00	1.00	1.00	1.00	1.00	1.00	1.00	1.00	1.00
18	0.83	0.85	0.95	0.91	0.95	0.97	0.99	1.00	1.00	1.00
19	0.41	0.60	0.75	0.92	0.98	0.98	1.00	0.99	1.00	1.00
20	0.42	0.59	0.72	0.75	0.89	0.93	0.99	1.00	1.00	1.00
21	0.35	0.57	0.56	0.72	0.73	0.88	0.89	0.98	0.98	1.00
22	0.91	0.97	0.99	1.00	1.00	1.00	1.00	1.00	1.00	1.00
23	0.90	0.90	0.97	0.98	0.98	1.00	1.00	1.00	1.00	1.00
24	0.88	0.93	0.93	0.99	0.98	0.99	1.00	1.00	1.00	1.00
25	0.24	0.51	0.72	0.88	0.97	0.98	1.00	1.00	1.00	1.00
26	0.50	0.43	0.56	0.55	0.53	0.62	0.69	0.74	0.85	0.76
27	0.36	0.38	0.38	0.31	0.42	0.43	0.49	0.54	0.49	0.53
28	0.95	0.98	0.98	1.00	1.00	1.00	1.00	1.00	1.00	1.00
29	0.69	0.74	0.78	0.70	0.80	0.77	0.87	0.92	0.97	0.95
30	0.49	0.44	0.59	0.54	0.53	0.58	0.63	0.70	0.75	0.79
31	0.05	0.17	0.22	0.43	0.62	0.96	0.93	0.99	0.99	1.00
32	0.13	0.22	0.38	0.54	0.62	0.71	0.86	0.92	0.97	1.00
33	0.21	0.27	0.30	0.49	0.50	0.70	0.80	0.94	0.96	0.99
34	0.99	1.00	1.00	1.00	1.00	1.00	1.00	1.00	1.00	1.00
35	1.00	0.99	1.00	1.00	1.00	1.00	1.00	1.00	1.00	1.00
36	0.93	0.97	0.99	1.00	1.00	1.00	1.00	1.00	1.00	1.00
37	0.07	0.14	0.41	0.50	0.71	0.88	0.93	0.98	0.98	1.00
38	0.14	0.13	0.28	0.56	0.65	0.86	0.90	0.93	0.99	1.00
39	0.08	0.26	0.33	0.52	0.61	0.76	0.85	0.96	1.00	1.00
40	0.21	0.44	0.59	0.78	0.88	0.99	0.99	0.99	1.00	1.00
41	0.22	0.38	0.41	0.68	0.81	0.92	0.98	0.99	1.00	1.00
42	0.27	0.40	0.42	0.66	0.78	0.89	0.99	0.99	1.00	1.00

Table 10: POD according to each scenario and each k2 value, k1 = 3, alpha = 0.01

	k2=1	k2=2	k2=3	k2=4	k2=5	k2=6	k2=7	k2=8	k2=9	k2=10
1	0.27	0.41	0.54	0.60	0.72	0.78	0.92	1.00	0.99	0.97
2	0.20	0.37	0.44	0.47	0.56	0.70	0.74	0.80	0.85	0.88
3	0.28	0.37	0.38	0.36	0.45	0.54	0.67	0.70	0.64	0.74
4	0.94	0.94	0.99	1.00	1.00	1.00	1.00	1.00	1.00	1.00
5	1.00	1.00	1.00	1.00	1.00	1.00	1.00	1.00	1.00	1.00
6	0.98	0.99	1.00	1.00	0.99	0.99	1.00	1.00	1.00	1.00
7	0.74	0.85	0.74	0.88	0.90	0.87	0.94	0.95	0.98	0.99
8	0.80	0.76	0.86	0.87	0.91	0.89	0.89	0.96	0.97	0.96
9	0.76	0.70	0.85	0.81	0.88	0.91	0.93	0.95	0.96	0.94
10	0.99	0.99	0.99	1.00	1.00	1.00	1.00	1.00	1.00	1.00
11	1.00	1.00	1.00	1.00	1.00	1.00	1.00	1.00	1.00	1.00
12	1.00	1.00	1.00	1.00	1.00	1.00	1.00	1.00	1.00	1.00
13	0.04	0.18	0.30	0.43	0.66	0.81	0.81	0.92	0.97	0.98
14	0.16	0.23	0.38	0.46	0.60	0.62	0.65	0.87	0.88	0.93
15	0.31	0.32	0.32	0.39	0.49	0.49	0.64	0.78	0.83	0.91
16	1.00	1.00	1.00	1.00	1.00	1.00	1.00	1.00	1.00	1.00
17	1.00	1.00	1.00	1.00	1.00	1.00	1.00	1.00	1.00	1.00
18	0.83	0.85	0.92	0.91	0.92	0.98	0.98	0.99	1.00	1.00
19	0.43	0.67	0.80	0.92	0.98	0.98	0.99	1.00	1.00	1.00
20	0.50	0.45	0.72	0.77	0.88	0.95	0.99	0.99	1.00	1.00
21	0.39	0.49	0.59	0.64	0.83	0.85	0.95	0.97	1.00	1.00
22	0.88	0.98	1.00	1.00	1.00	1.00	1.00	1.00	1.00	1.00
23	0.87	0.94	0.97	1.00	0.99	0.99	0.99	1.00	1.00	1.00
24	0.94	0.94	0.95	0.99	0.99	1.00	0.98	0.99	1.00	1.00
25	0.33	0.59	0.67	0.85	0.96	0.99	0.99	1.00	1.00	1.00
26	0.52	0.56	0.54	0.60	0.61	0.64	0.69	0.69	0.76	0.88
27	0.32	0.35	0.34	0.34	0.37	0.44	0.46	0.46	0.50	0.60
28	0.92	0.99	1.00	0.99	1.00	1.00	1.00	1.00	1.00	1.00
29	0.71	0.69	0.74	0.79	0.78	0.84	0.84	0.86	0.87	0.96
30	0.53	0.46	0.44	0.45	0.56	0.63	0.67	0.63	0.68	0.74
31	0.07	0.12	0.34	0.33	0.63	0.79	0.92	0.96	1.00	1.00
32	0.16	0.21	0.46	0.40	0.70	0.75	0.89	0.90	0.96	0.98
33	0.16	0.29	0.33	0.36	0.51	0.60	0.75	0.87	0.94	0.98
34	0.99	1.00	1.00	1.00	1.00	1.00	1.00	1.00	1.00	1.00
35	0.99	1.00	1.00	1.00	1.00	1.00	1.00	1.00	1.00	1.00
36	0.93	0.90	0.96	0.98	0.99	1.00	1.00	1.00	1.00	1.00
37	0.02	0.10	0.27	0.47	0.72	0.85	0.94	0.98	0.97	1.00
38	0.03	0.17	0.18	0.41	0.72	0.86	0.93	0.98	1.00	1.00
39	0.13	0.14	0.38	0.34	0.60	0.78	0.86	0.97	0.99	1.00
40	0.20	0.29	0.59	0.70	0.83	0.91	0.99	0.99	1.00	1.00
41	0.23	0.27	0.62	0.65	0.75	0.93	0.98	1.00	1.00	1.00
42	0.18	0.28	0.51	0.64	0.84	0.87	0.98	0.99	1.00	1.00

Table 11: POD according to each scenario and each k2 value, k1 = 5, alpha = 0.01

	k2=1	k2=2	k2=3	k2=4	k2=5	k2=6	k2=7	k2=8	k2=9	k2=10
1	0.23	0.29	0.41	0.66	0.73	0.81	0.94	0.92	0.96	0.98
2	0.19	0.38	0.36	0.45	0.56	0.60	0.68	0.71	0.87	0.92
3	0.32	0.23	0.44	0.45	0.50	0.52	0.56	0.73	0.63	0.76
4	0.96	0.97	0.99	0.99	1.00	1.00	1.00	1.00	1.00	1.00
5	0.98	0.98	1.00	1.00	1.00	1.00	0.99	1.00	1.00	1.00
6	0.96	0.99	0.98	0.97	0.97	0.99	1.00	1.00	1.00	1.00
7	0.68	0.74	0.80	0.83	0.83	0.87	0.92	0.93	0.96	0.95
8	0.66	0.72	0.77	0.82	0.93	0.88	0.94	0.89	0.95	0.93
9	0.73	0.70	0.78	0.79	0.90	0.89	0.93	0.95	0.99	0.97
10	0.99	1.00	1.00	1.00	1.00	1.00	1.00	1.00	1.00	1.00
11	1.00	1.00	1.00	1.00	1.00	1.00	1.00	1.00	1.00	1.00
12	0.99	1.00	1.00	1.00	1.00	1.00	1.00	1.00	1.00	1.00
13	0.05	0.11	0.21	0.41	0.56	0.77	0.81	0.90	0.97	1.00
14	0.23	0.26	0.33	0.42	0.59	0.62	0.75	0.78	0.84	0.86
15	0.28	0.23	0.25	0.33	0.38	0.52	0.69	0.75	0.74	0.83
16	1.00	1.00	1.00	1.00	1.00	1.00	1.00	1.00	1.00	1.00
17	1.00	1.00	1.00	1.00	1.00	1.00	1.00	1.00	1.00	1.00
18	0.85	0.83	0.94	0.96	0.96	0.93	0.97	1.00	1.00	1.00
19	0.34	0.57	0.74	0.86	0.97	0.99	1.00	1.00	1.00	1.00
20	0.45	0.48	0.58	0.73	0.92	0.94	0.99	0.98	0.99	0.99
21	0.42	0.50	0.59	0.76	0.68	0.84	0.89	0.92	0.95	0.99
22	0.84	0.92	0.99	0.99	1.00	1.00	1.00	1.00	1.00	1.00
23	0.83	0.90	0.95	0.98	1.00	1.00	1.00	1.00	1.00	1.00
24	0.87	0.87	0.90	0.92	0.97	0.99	0.99	1.00	1.00	1.00
25	0.31	0.43	0.68	0.84	0.92	0.96	0.99	1.00	1.00	1.00
26	0.35	0.51	0.46	0.52	0.54	0.57	0.65	0.71	0.76	0.80
27	0.41	0.33	0.41	0.45	0.48	0.39	0.56	0.52	0.48	0.65
28	0.89	0.95	0.99	0.98	1.00	1.00	1.00	1.00	1.00	1.00
29	0.72	0.74	0.75	0.80	0.79	0.80	0.88	0.86	0.88	0.93
30	0.45	0.48	0.44	0.40	0.59	0.58	0.63	0.64	0.77	0.73
31	0.07	0.06	0.29	0.39	0.71	0.84	0.91	0.98	1.00	1.00
32	0.18	0.27	0.39	0.48	0.56	0.73	0.83	0.92	0.98	0.98
33	0.21	0.28	0.29	0.46	0.47	0.64	0.77	0.88	0.94	0.97
34	1.00	1.00	1.00	1.00	1.00	1.00	1.00	1.00	1.00	1.00
35	1.00	0.99	1.00	1.00	1.00	1.00	1.00	1.00	1.00	1.00
36	0.95	0.94	0.99	0.99	1.00	1.00	1.00	1.00	1.00	1.00
37	0.03	0.07	0.23	0.49	0.70	0.87	0.95	0.97	0.98	1.00
38	0.04	0.11	0.30	0.44	0.65	0.78	0.94	0.99	1.00	1.00
39	0.06	0.26	0.24	0.46	0.56	0.79	0.84	1.00	1.00	1.00
40	0.16	0.26	0.46	0.79	0.83	0.93	0.99	0.99	1.00	1.00
41	0.26	0.33	0.38	0.66	0.87	0.85	0.93	1.00	0.99	1.00
42	0.21	0.37	0.46	0.74	0.83	0.89	0.97	0.99	1.00	1.00

Table 12: POD according to each scenario and each k2 value, k1 = 10, alpha = 0.01

Overall performances of CUSUM algorithm ($\alpha = 0.05$)

	FPR k1=0	FPR k1=2	FPR k1=3	FPR k1=5	FPR k1=10
1	0.05	0.05	0.05	0.05	0.04
2	0.13	0.12	0.12	0.12	0.11
3	0.23	0.23	0.23	0.22	0.22
4	0.87	0.86	0.86	0.85	0.82
5	0.98	0.97	0.97	0.97	0.96
6	0.98	0.98	0.98	0.97	0.96
7	0.09	0.08	0.08	0.08	0.08
8	0.09	0.08	0.08	0.08	0.07
9	0.10	0.09	0.09	0.08	0.08
10	0.93	0.93	0.93	0.92	0.91
11	0.97	0.97	0.97	0.96	0.96
12	0.99	0.99	0.99	0.99	0.99
13	0.01	0.01	0.01	0.01	0.01
14	0.10	0.09	0.09	0.09	0.08
15	0.18	0.18	0.17	0.17	0.17
16	1.00	1.00	1.00	1.00	1.00
17	1.00	1.00	1.00	1.00	1.00
18	0.77	0.77	0.77	0.77	0.77
19	0.24	0.23	0.22	0.21	0.19
20	0.28	0.27	0.27	0.26	0.24
21	0.26	0.25	0.25	0.25	0.23
22	0.75	0.73	0.72	0.71	0.67
23	0.85	0.84	0.83	0.82	0.78
24	0.87	0.86	0.85	0.83	0.80
25	0.14	0.13	0.13	0.12	0.11
26	0.39	0.39	0.39	0.39	0.38
27	0.30	0.30	0.30	0.30	0.30
28	0.82	0.81	0.81	0.80	0.78
29	0.65	0.65	0.65	0.64	0.63
30	0.42	0.42	0.42	0.42	0.42
31	0.01	0.01	0.01	0.01	0.01
32	0.07	0.06	0.06	0.06	0.05
33	0.12	0.12	0.12	0.12	0.11
34	0.98	0.98	0.98	0.98	0.98
35	0.99	0.99	0.98	0.98	0.98
36	0.88	0.87	0.87	0.86	0.86
37	0.01	0.01	0.01	0.01	0.01
38	0.02	0.02	0.02	0.02	0.02
39	0.04	0.04	0.04	0.04	0.03
40	0.06	0.05	0.05	0.05	0.04
41	0.09	0.08	0.08	0.08	0.07
42	0.12	0.12	0.11	0.11	0.11

Table 13: FPR according to each scenario and each k1 value, alpha = 0.05

	k2=1	k2=2	k2=3	k2=4	k2=5	k2=6	k2=7	k2=8	k2=9	k2=10
1	0.29	0.39	0.46	0.58	0.77	0.79	0.97	0.96	1.00	0.98
2	0.31	0.36	0.40	0.57	0.62	0.60	0.74	0.86	0.89	0.93
3	0.37	0.41	0.34	0.47	0.46	0.53	0.64	0.68	0.75	0.82
4	0.94	0.98	1.00	1.00	1.00	1.00	1.00	1.00	1.00	1.00
5	0.98	0.99	0.98	1.00	1.00	1.00	1.00	1.00	1.00	1.00
6	0.98	1.00	1.00	0.98	0.99	0.99	1.00	1.00	1.00	1.00
7	0.90	0.86	0.92	0.94	0.97	0.94	0.93	0.97	0.97	0.99
8	0.90	0.91	0.89	0.96	0.93	0.98	0.93	0.97	0.96	0.98
9	0.87	0.89	0.90	0.90	0.93	0.98	0.95	0.99	0.97	1.00
10	0.97	0.98	1.00	1.00	1.00	1.00	1.00	1.00	1.00	1.00
11	1.00	1.00	1.00	1.00	1.00	1.00	1.00	1.00	1.00	1.00
12	1.00	1.00	1.00	1.00	1.00	1.00	1.00	1.00	1.00	1.00
13	0.05	0.12	0.24	0.46	0.63	0.75	0.79	0.92	0.96	0.98
14	0.24	0.28	0.43	0.39	0.48	0.59	0.75	0.80	0.86	0.89
15	0.24	0.21	0.23	0.41	0.44	0.61	0.63	0.74	0.79	0.89
16	1.00	1.00	1.00	1.00	1.00	1.00	1.00	1.00	1.00	1.00
17	1.00	1.00	1.00	1.00	1.00	1.00	1.00	1.00	1.00	1.00
18	0.86	0.85	0.88	0.96	0.93	0.94	0.99	0.99	1.00	1.00
19	0.36	0.58	0.77	0.90	0.98	0.99	1.00	1.00	1.00	1.00
20	0.45	0.59	0.66	0.82	0.89	0.96	0.98	0.98	1.00	1.00
21	0.37	0.45	0.61	0.67	0.79	0.92	0.94	0.99	0.99	0.99
22	0.90	0.98	1.00	1.00	1.00	1.00	1.00	1.00	1.00	1.00
23	0.94	0.92	0.97	1.00	1.00	1.00	1.00	1.00	1.00	1.00
24	0.91	0.97	0.99	0.99	0.99	1.00	1.00	1.00	1.00	1.00
25	0.34	0.49	0.75	0.87	0.97	0.98	1.00	1.00	1.00	1.00
26	0.45	0.51	0.59	0.67	0.62	0.64	0.64	0.78	0.81	0.80
27	0.26	0.39	0.39	0.36	0.40	0.49	0.45	0.37	0.55	0.61
28	0.97	0.96	1.00	1.00	1.00	1.00	1.00	1.00	1.00	1.00
29	0.74	0.69	0.81	0.81	0.79	0.74	0.85	0.83	0.92	0.92
30	0.42	0.46	0.48	0.54	0.60	0.61	0.70	0.61	0.75	0.80
31	0.07	0.10	0.17	0.44	0.61	0.87	0.96	0.99	0.99	1.00
32	0.18	0.22	0.34	0.47	0.65	0.74	0.86	0.90	0.95	1.00
33	0.17	0.23	0.24	0.44	0.59	0.69	0.82	0.91	0.91	0.96
34	1.00	1.00	1.00	1.00	1.00	1.00	1.00	1.00	1.00	1.00
35	1.00	1.00	1.00	1.00	1.00	1.00	1.00	1.00	1.00	1.00
36	0.92	0.94	0.99	0.99	0.99	1.00	1.00	1.00	1.00	1.00
37	0.03	0.17	0.24	0.60	0.73	0.78	0.96	0.99	0.99	1.00
38	0.03	0.19	0.33	0.49	0.59	0.84	0.93	1.00	1.00	1.00
39	0.05	0.21	0.40	0.54	0.65	0.81	0.87	0.97	0.99	1.00
40	0.14	0.45	0.65	0.88	0.88	0.96	0.99	1.00	1.00	1.00
41	0.28	0.35	0.53	0.71	0.90	0.92	0.97	0.99	0.99	1.00
42	0.21	0.31	0.43	0.58	0.82	0.86	0.97	0.98	1.00	1.00

Table 14: POD according to each scenario and each k2 value, k1 = 0, alpha = 0.05

	k2=1	k2=2	k2=3	k2=4	k2=5	k2=6	k2=7	k2=8	k2=9	k2=10
1	0.20	0.37	0.54	0.56	0.80	0.80	0.89	0.97	0.95	0.96
2	0.33	0.34	0.35	0.53	0.62	0.71	0.68	0.80	0.84	0.88
3	0.27	0.37	0.39	0.38	0.45	0.62	0.66	0.65	0.76	0.81
4	0.99	1.00	1.00	1.00	1.00	1.00	1.00	1.00	1.00	1.00
5	0.99	1.00	1.00	0.99	1.00	1.00	1.00	1.00	1.00	1.00
6	0.99	0.99	1.00	1.00	0.98	1.00	1.00	0.99	1.00	1.00
7	0.77	0.79	0.85	0.89	0.91	0.90	0.96	0.96	0.98	0.97
8	0.84	0.84	0.88	0.85	0.91	0.90	0.94	0.94	0.98	0.98
9	0.73	0.78	0.83	0.86	0.87	0.94	0.94	0.98	0.96	0.99
10	0.99	1.00	1.00	1.00	1.00	1.00	1.00	1.00	1.00	1.00
11	0.99	0.99	1.00	1.00	1.00	1.00	1.00	1.00	1.00	1.00
12	1.00	1.00	1.00	1.00	1.00	1.00	1.00	1.00	1.00	1.00
13	0.03	0.10	0.25	0.45	0.67	0.70	0.92	0.92	0.98	0.98
14	0.29	0.27	0.34	0.42	0.53	0.64	0.73	0.82	0.91	0.95
15	0.32	0.29	0.35	0.37	0.56	0.56	0.59	0.71	0.76	0.87
16	1.00	1.00	1.00	1.00	1.00	1.00	1.00	1.00	1.00	1.00
17	1.00	1.00	1.00	1.00	1.00	1.00	1.00	1.00	1.00	1.00
18	0.85	0.88	0.93	0.95	0.93	0.98	0.99	0.98	1.00	1.00
19	0.43	0.63	0.74	0.92	0.98	0.99	1.00	1.00	1.00	1.00
20	0.47	0.63	0.69	0.85	0.90	0.95	0.96	1.00	1.00	1.00
21	0.42	0.34	0.55	0.67	0.77	0.88	0.94	0.98	0.99	0.99
22	0.88	0.96	0.99	1.00	1.00	1.00	1.00	1.00	1.00	1.00
23	0.93	0.94	0.98	0.98	0.99	1.00	1.00	1.00	1.00	1.00
24	0.83	0.95	0.95	0.98	0.99	0.99	1.00	1.00	1.00	1.00
25	0.28	0.51	0.60	0.88	0.97	0.97	1.00	1.00	1.00	1.00
26	0.44	0.47	0.54	0.52	0.61	0.64	0.70	0.72	0.72	0.78
27	0.42	0.34	0.32	0.33	0.46	0.40	0.47	0.41	0.60	0.61
28	0.93	0.97	0.99	1.00	1.00	1.00	1.00	1.00	1.00	1.00
29	0.65	0.70	0.72	0.72	0.81	0.88	0.79	0.92	0.92	0.96
30	0.40	0.51	0.50	0.40	0.60	0.65	0.52	0.66	0.75	0.83
31	0.08	0.15	0.23	0.49	0.68	0.85	0.91	0.99	0.99	1.00
32	0.19	0.25	0.43	0.47	0.60	0.75	0.84	0.92	0.94	0.99
33	0.23	0.23	0.31	0.40	0.56	0.64	0.80	0.88	0.97	0.98
34	1.00	1.00	1.00	1.00	1.00	1.00	1.00	1.00	1.00	1.00
35	0.99	1.00	1.00	1.00	1.00	1.00	1.00	1.00	1.00	1.00
36	0.93	0.99	0.99	0.97	0.99	0.99	1.00	1.00	1.00	1.00
37	0.07	0.17	0.31	0.53	0.78	0.79	0.90	0.99	0.99	1.00
38	0.08	0.17	0.27	0.53	0.68	0.85	0.91	0.98	1.00	1.00
39	0.15	0.14	0.31	0.48	0.63	0.82	0.89	0.99	0.99	1.00
40	0.21	0.48	0.58	0.80	0.94	0.95	0.99	1.00	0.99	1.00
41	0.22	0.29	0.58	0.68	0.79	0.95	0.99	0.99	1.00	1.00
42	0.25	0.31	0.51	0.54	0.78	0.89	0.97	0.98	1.00	1.00

Table 15: POD according to each scenario and each k2 value, k1 = 2, alpha = 0.05

	k2=1	k2=2	k2=3	k2=4	k2=5	k2=6	k2=7	k2=8	k2=9	k2=10
1	0.28	0.29	0.49	0.67	0.83	0.83	0.92	0.98	0.95	0.97
2	0.24	0.33	0.43	0.48	0.57	0.64	0.78	0.75	0.82	0.86
3	0.23	0.34	0.42	0.45	0.39	0.48	0.65	0.81	0.72	0.83
4	0.97	0.97	0.98	1.00	1.00	1.00	1.00	1.00	1.00	1.00
5	1.00	1.00	0.99	1.00	1.00	1.00	1.00	1.00	1.00	1.00
6	1.00	1.00	0.97	1.00	1.00	1.00	1.00	1.00	1.00	1.00
7	0.78	0.79	0.87	0.90	0.90	0.95	0.95	0.98	0.95	0.99
8	0.77	0.88	0.95	0.90	0.88	0.94	0.94	0.96	0.98	0.96
9	0.78	0.85	0.86	0.89	0.91	0.92	0.95	0.95	0.94	0.97
10	0.98	1.00	1.00	1.00	1.00	1.00	1.00	1.00	1.00	1.00
11	1.00	1.00	1.00	1.00	1.00	1.00	1.00	1.00	1.00	1.00
12	1.00	1.00	1.00	1.00	1.00	1.00	1.00	1.00	1.00	1.00
13	0.04	0.15	0.27	0.43	0.64	0.76	0.87	0.93	0.99	1.00
14	0.22	0.22	0.36	0.37	0.50	0.59	0.76	0.81	0.82	0.89
15	0.19	0.24	0.26	0.38	0.43	0.53	0.64	0.79	0.76	0.77
16	1.00	1.00	1.00	1.00	1.00	1.00	1.00	1.00	1.00	1.00
17	1.00	1.00	1.00	1.00	1.00	1.00	1.00	1.00	1.00	1.00
18	0.83	0.85	0.95	0.91	0.95	0.97	0.99	1.00	1.00	1.00
19	0.41	0.60	0.75	0.92	0.98	0.98	1.00	0.99	1.00	1.00
20	0.42	0.59	0.72	0.75	0.89	0.93	0.99	1.00	1.00	1.00
21	0.35	0.57	0.56	0.72	0.73	0.88	0.89	0.98	0.98	1.00
22	0.91	0.97	0.99	1.00	1.00	1.00	1.00	1.00	1.00	1.00
23	0.90	0.90	0.97	0.98	0.98	1.00	1.00	1.00	1.00	1.00
24	0.88	0.93	0.93	0.99	0.98	0.99	1.00	1.00	1.00	1.00
25	0.24	0.51	0.72	0.88	0.97	0.98	1.00	1.00	1.00	1.00
26	0.50	0.43	0.56	0.55	0.53	0.62	0.69	0.74	0.85	0.76
27	0.36	0.38	0.38	0.31	0.42	0.43	0.49	0.54	0.49	0.53
28	0.95	0.98	0.98	1.00	1.00	1.00	1.00	1.00	1.00	1.00
29	0.69	0.74	0.78	0.70	0.80	0.77	0.87	0.92	0.97	0.95
30	0.49	0.44	0.59	0.54	0.53	0.58	0.63	0.70	0.75	0.79
31	0.05	0.17	0.22	0.43	0.62	0.96	0.93	0.99	0.99	1.00
32	0.13	0.22	0.38	0.54	0.62	0.71	0.86	0.92	0.97	1.00
33	0.21	0.27	0.30	0.49	0.50	0.70	0.80	0.94	0.96	0.99
34	0.99	1.00	1.00	1.00	1.00	1.00	1.00	1.00	1.00	1.00
35	1.00	0.99	1.00	1.00	1.00	1.00	1.00	1.00	1.00	1.00
36	0.93	0.97	0.99	1.00	1.00	1.00	1.00	1.00	1.00	1.00
37	0.07	0.14	0.41	0.50	0.71	0.88	0.93	0.98	0.98	1.00
38	0.14	0.13	0.28	0.56	0.65	0.86	0.90	0.93	0.99	1.00
39	0.08	0.26	0.33	0.52	0.61	0.76	0.85	0.96	1.00	1.00
40	0.21	0.44	0.59	0.78	0.88	0.99	0.99	0.99	1.00	1.00
41	0.22	0.38	0.41	0.68	0.81	0.92	0.98	0.99	1.00	1.00
42	0.27	0.40	0.42	0.66	0.78	0.89	0.99	0.99	1.00	1.00

Table 16: POD according to each scenario and each k2 value, k1 = 3, alpha = 0.05

	k2=1	k2=2	k2=3	k2=4	k2=5	k2=6	k2=7	k2=8	k2=9	k2=10
1	0.27	0.41	0.54	0.60	0.72	0.78	0.92	1.00	0.99	0.97
2	0.20	0.37	0.44	0.47	0.56	0.70	0.74	0.80	0.85	0.88
3	0.28	0.37	0.38	0.36	0.45	0.54	0.67	0.70	0.64	0.74
4	0.94	0.94	0.99	1.00	1.00	1.00	1.00	1.00	1.00	1.00
5	1.00	1.00	1.00	1.00	1.00	1.00	1.00	1.00	1.00	1.00
6	0.98	0.99	1.00	1.00	0.99	0.99	1.00	1.00	1.00	1.00
7	0.74	0.85	0.74	0.88	0.90	0.87	0.94	0.95	0.98	0.99
8	0.80	0.76	0.86	0.87	0.91	0.89	0.89	0.96	0.97	0.96
9	0.76	0.70	0.85	0.81	0.88	0.91	0.93	0.95	0.96	0.94
10	0.99	0.99	0.99	1.00	1.00	1.00	1.00	1.00	1.00	1.00
11	1.00	1.00	1.00	1.00	1.00	1.00	1.00	1.00	1.00	1.00
12	1.00	1.00	1.00	1.00	1.00	1.00	1.00	1.00	1.00	1.00
13	0.04	0.18	0.30	0.43	0.66	0.81	0.81	0.92	0.97	0.98
14	0.16	0.23	0.38	0.46	0.60	0.62	0.65	0.87	0.88	0.93
15	0.31	0.32	0.32	0.39	0.49	0.49	0.64	0.78	0.83	0.91
16	1.00	1.00	1.00	1.00	1.00	1.00	1.00	1.00	1.00	1.00
17	1.00	1.00	1.00	1.00	1.00	1.00	1.00	1.00	1.00	1.00
18	0.83	0.85	0.92	0.91	0.92	0.98	0.98	0.99	1.00	1.00
19	0.43	0.67	0.80	0.92	0.98	0.98	0.99	1.00	1.00	1.00
20	0.50	0.45	0.72	0.77	0.88	0.95	0.99	0.99	1.00	1.00
21	0.39	0.49	0.59	0.64	0.83	0.85	0.95	0.97	1.00	1.00
22	0.88	0.98	1.00	1.00	1.00	1.00	1.00	1.00	1.00	1.00
23	0.87	0.94	0.97	1.00	0.99	0.99	0.99	1.00	1.00	1.00
24	0.94	0.94	0.95	0.99	0.99	1.00	0.98	0.99	1.00	1.00
25	0.33	0.59	0.67	0.85	0.96	0.99	0.99	1.00	1.00	1.00
26	0.52	0.56	0.54	0.60	0.61	0.64	0.69	0.69	0.76	0.88
27	0.32	0.35	0.34	0.34	0.37	0.44	0.46	0.46	0.50	0.60
28	0.92	0.99	1.00	0.99	1.00	1.00	1.00	1.00	1.00	1.00
29	0.71	0.69	0.74	0.79	0.78	0.84	0.84	0.86	0.87	0.96
30	0.53	0.46	0.44	0.45	0.56	0.63	0.67	0.63	0.68	0.74
31	0.07	0.12	0.34	0.33	0.63	0.79	0.92	0.96	1.00	1.00
32	0.16	0.21	0.46	0.40	0.70	0.75	0.89	0.90	0.96	0.98
33	0.16	0.29	0.33	0.36	0.51	0.60	0.75	0.87	0.94	0.98
34	0.99	1.00	1.00	1.00	1.00	1.00	1.00	1.00	1.00	1.00
35	0.99	1.00	1.00	1.00	1.00	1.00	1.00	1.00	1.00	1.00
36	0.93	0.90	0.96	0.98	0.99	1.00	1.00	1.00	1.00	1.00
37	0.02	0.10	0.27	0.47	0.72	0.85	0.94	0.98	0.97	1.00
38	0.03	0.17	0.18	0.41	0.72	0.86	0.93	0.98	1.00	1.00
39	0.13	0.14	0.38	0.34	0.60	0.78	0.86	0.97	0.99	1.00
40	0.20	0.29	0.59	0.70	0.83	0.91	0.99	0.99	1.00	1.00
41	0.23	0.27	0.62	0.65	0.75	0.93	0.98	1.00	1.00	1.00
42	0.18	0.28	0.51	0.64	0.84	0.87	0.98	0.99	1.00	1.00

Table 17: POD according to each scenario and each k2 value, k1 = 5, alpha = 0.05

	k2=1	k2=2	k2=3	k2=4	k2=5	k2=6	k2=7	k2=8	k2=9	k2=10
1	0.23	0.29	0.41	0.66	0.73	0.81	0.94	0.92	0.96	0.98
2	0.19	0.38	0.36	0.45	0.56	0.60	0.68	0.71	0.87	0.92
3	0.32	0.23	0.44	0.45	0.50	0.52	0.56	0.73	0.63	0.76
4	0.96	0.97	0.99	0.99	1.00	1.00	1.00	1.00	1.00	1.00
5	0.98	0.98	1.00	1.00	1.00	1.00	0.99	1.00	1.00	1.00
6	0.96	0.99	0.98	0.97	0.97	0.99	1.00	1.00	1.00	1.00
7	0.68	0.74	0.80	0.83	0.83	0.87	0.92	0.93	0.96	0.95
8	0.66	0.72	0.77	0.82	0.93	0.88	0.94	0.89	0.95	0.93
9	0.73	0.70	0.78	0.79	0.90	0.89	0.93	0.95	0.99	0.97
10	0.99	1.00	1.00	1.00	1.00	1.00	1.00	1.00	1.00	1.00
11	1.00	1.00	1.00	1.00	1.00	1.00	1.00	1.00	1.00	1.00
12	0.99	1.00	1.00	1.00	1.00	1.00	1.00	1.00	1.00	1.00
13	0.05	0.11	0.21	0.41	0.56	0.77	0.81	0.90	0.97	1.00
14	0.23	0.26	0.33	0.42	0.59	0.62	0.75	0.78	0.84	0.86
15	0.28	0.23	0.25	0.33	0.38	0.52	0.69	0.75	0.74	0.83
16	1.00	1.00	1.00	1.00	1.00	1.00	1.00	1.00	1.00	1.00
17	1.00	1.00	1.00	1.00	1.00	1.00	1.00	1.00	1.00	1.00
18	0.85	0.83	0.94	0.96	0.96	0.93	0.97	1.00	1.00	1.00
19	0.34	0.57	0.74	0.86	0.97	0.99	1.00	1.00	1.00	1.00
20	0.45	0.48	0.58	0.73	0.92	0.94	0.99	0.98	0.99	0.99
21	0.42	0.50	0.59	0.76	0.68	0.84	0.89	0.92	0.95	0.99
22	0.84	0.92	0.99	0.99	1.00	1.00	1.00	1.00	1.00	1.00
23	0.83	0.90	0.95	0.98	1.00	1.00	1.00	1.00	1.00	1.00
24	0.87	0.87	0.90	0.92	0.97	0.99	0.99	1.00	1.00	1.00
25	0.31	0.43	0.68	0.84	0.92	0.96	0.99	1.00	1.00	1.00
26	0.35	0.51	0.46	0.52	0.54	0.57	0.65	0.71	0.76	0.80
27	0.41	0.33	0.41	0.45	0.48	0.39	0.56	0.52	0.48	0.65
28	0.89	0.95	0.99	0.98	1.00	1.00	1.00	1.00	1.00	1.00
29	0.72	0.74	0.75	0.80	0.79	0.80	0.88	0.86	0.88	0.93
30	0.45	0.48	0.44	0.40	0.59	0.58	0.63	0.64	0.77	0.73
31	0.07	0.06	0.29	0.39	0.71	0.84	0.91	0.98	1.00	1.00
32	0.18	0.27	0.39	0.48	0.56	0.73	0.83	0.92	0.98	0.98
33	0.21	0.28	0.29	0.46	0.47	0.64	0.77	0.88	0.94	0.97
34	1.00	1.00	1.00	1.00	1.00	1.00	1.00	1.00	1.00	1.00
35	1.00	0.99	1.00	1.00	1.00	1.00	1.00	1.00	1.00	1.00
36	0.95	0.94	0.99	0.99	1.00	1.00	1.00	1.00	1.00	1.00
37	0.03	0.07	0.23	0.49	0.70	0.87	0.95	0.97	0.98	1.00
38	0.04	0.11	0.30	0.44	0.65	0.78	0.94	0.99	1.00	1.00
39	0.06	0.26	0.24	0.46	0.56	0.79	0.84	1.00	1.00	1.00
40	0.16	0.26	0.46	0.79	0.83	0.93	0.99	0.99	1.00	1.00
41	0.26	0.33	0.38	0.66	0.87	0.85	0.93	1.00	0.99	1.00
42	0.21	0.37	0.46	0.74	0.83	0.89	0.97	0.99	1.00	1.00

Table 18: POD according to each scenario and each k2 value, k1 = 10, alpha = 0.05
