# Supplementary material for: Evaluation and comparison of statistical methods for early temporal detection of outbreaks: A simulation-based study
Source: PLoS One. 2017 Jul 17;12(7):e0181227. doi: 10.1371/journal.pone.0181227 (PMC5513450; doi:10.1371/journal.pone.0181227)
Supplement: S8 Appendix — (PDF) [file pone.0181227.s008.pdf]

Evaluation and Comparison of Statistical Methods for Early Temporal Detection of Outbreaks: a Simulation-Based Study

Appendix S8: Overall performances of CUSUM Rossi algorithm ($\alpha = 0.001, 0.01$ and 0.05)

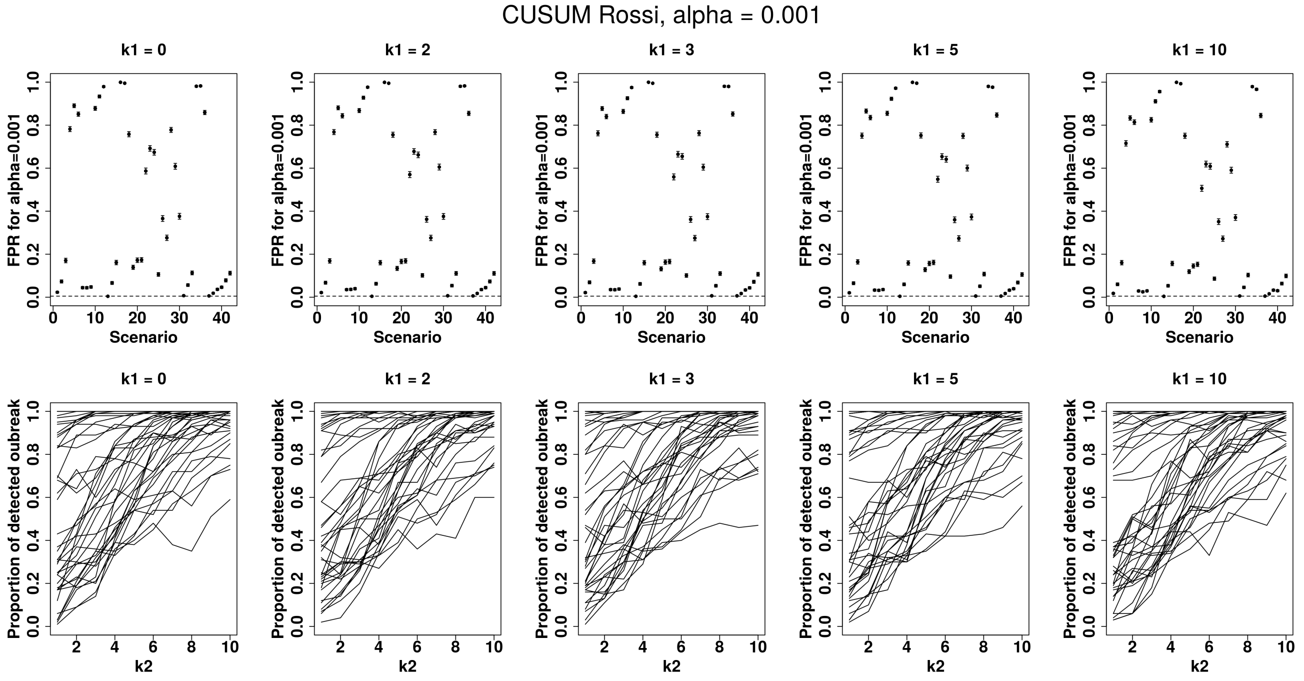

Figure 1: CUSUM Rossi algorithm performances for $\alpha = 0.001$ by increasing past outbreak amplitude $k_1 = 0, 2, 3, 5$ or 10 with (i) on the first row: false positive rate for 42 simulated scenarios, (ii) on the second row: probability of detection for 42 simulated scenarios (each curve corresponding to a scenario) by increasing current outbreak amplitude $k_2 = 1$ to 10.

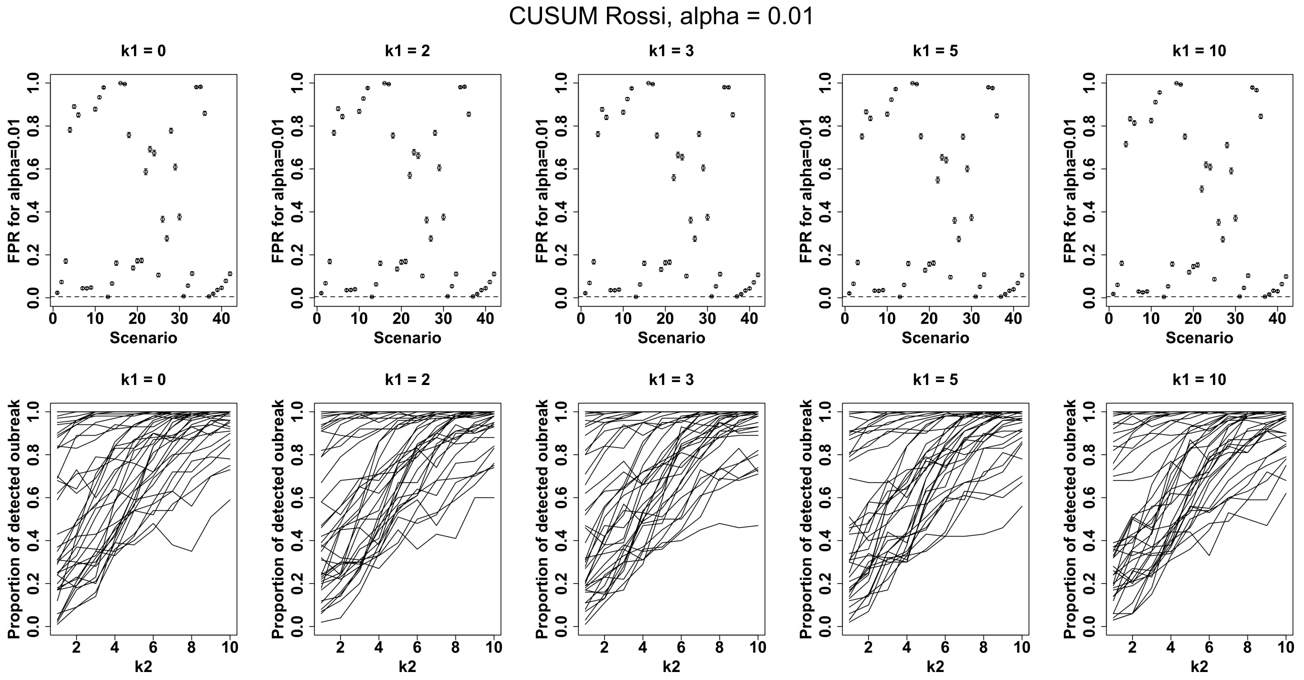

Figure 2: CUSUM Rossi algorithm performances for $\alpha = 0.01$ by increasing past outbreak amplitude $k_1 = 0, 2, 3, 5$ or 10 with (i) on the first row: false positive rate for 42 simulated scenarios, (ii) on the second row: probability of detection for 42 simulated scenarios (each curve corresponding to a scenario) by increasing current outbreak amplitude $k_2 = 1$ to 10.

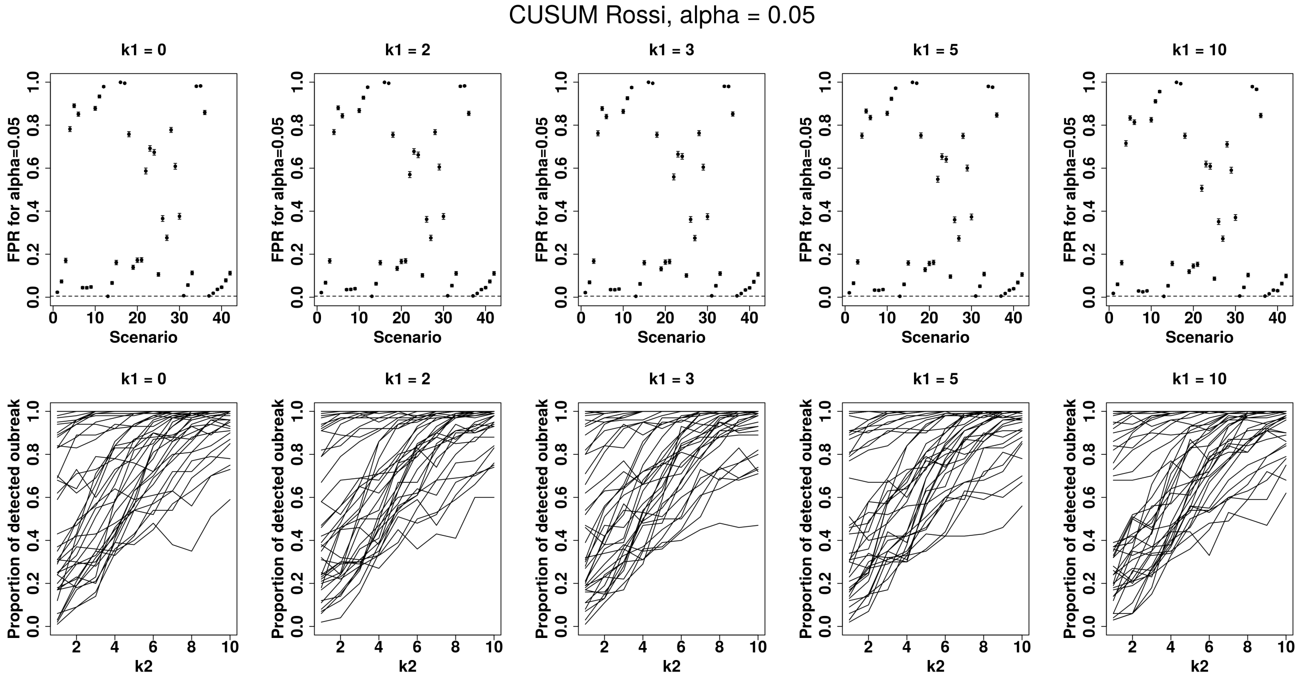

Figure 3: CUSUM Rossi algorithm performances for $\alpha = 0.05$ by increasing past outbreak amplitude $k_1 = 0, 2, 3, 5$ or 10 with (i) on the first row: false positive rate for 42 simulated scenarios, (ii) on the second row: probability of detection for 42 simulated scenarios (each curve corresponding to a scenario) by increasing current outbreak amplitude $k_2 = 1$ to 10.

Overall performances of CUSUM Rossi algorithm ($\alpha = 0.001$)

	FPR k1=0	FPR k1=2	FPR k1=3	FPR k1=5	FPR k1=10
1	0.02	0.02	0.02	0.02	0.02
2	0.07	0.07	0.07	0.07	0.06
3	0.17	0.17	0.17	0.16	0.16
4	0.78	0.77	0.76	0.75	0.72
5	0.89	0.88	0.88	0.87	0.83
6	0.85	0.84	0.84	0.84	0.81
7	0.04	0.04	0.04	0.03	0.03
8	0.04	0.04	0.03	0.03	0.03
9	0.05	0.04	0.04	0.04	0.03
10	0.88	0.87	0.86	0.86	0.83
11	0.93	0.93	0.93	0.92	0.91
12	0.98	0.98	0.98	0.97	0.96
13	0.00	0.00	0.00	0.00	0.00
14	0.07	0.06	0.06	0.06	0.05
15	0.16	0.16	0.16	0.16	0.16
16	1.00	1.00	1.00	1.00	1.00
17	1.00	0.99	0.99	1.00	0.99
18	0.76	0.76	0.76	0.75	0.75
19	0.14	0.13	0.13	0.13	0.12
20	0.17	0.17	0.16	0.16	0.15
21	0.17	0.17	0.17	0.16	0.15
22	0.59	0.57	0.56	0.55	0.51
23	0.69	0.68	0.66	0.65	0.62
24	0.67	0.66	0.65	0.64	0.61
25	0.11	0.10	0.10	0.10	0.09
26	0.37	0.36	0.36	0.36	0.35
27	0.28	0.28	0.27	0.27	0.27
28	0.78	0.77	0.76	0.75	0.71
29	0.61	0.61	0.60	0.60	0.59
30	0.38	0.38	0.37	0.37	0.37
31	0.01	0.01	0.01	0.01	0.01
32	0.06	0.05	0.05	0.05	0.05
33	0.11	0.11	0.11	0.11	0.10
34	0.98	0.98	0.98	0.98	0.98
35	0.98	0.98	0.98	0.98	0.97
36	0.86	0.85	0.85	0.85	0.84
37	0.01	0.01	0.01	0.01	0.01
38	0.02	0.02	0.02	0.02	0.02
39	0.04	0.04	0.03	0.03	0.03
40	0.05	0.04	0.04	0.04	0.03
41	0.08	0.07	0.07	0.07	0.06
42	0.11	0.11	0.11	0.11	0.10

Table 1: FPR according to each scenario and each k1 value, alpha = 0.001

	k2=1	k2=2	k2=3	k2=4	k2=5	k2=6	k2=7	k2=8	k2=9	k2=10
1	0.17	0.29	0.30	0.43	0.63	0.70	0.83	0.94	0.97	0.96
2	0.25	0.29	0.33	0.45	0.54	0.52	0.69	0.81	0.84	0.90
3	0.31	0.30	0.28	0.39	0.38	0.45	0.57	0.63	0.70	0.75
4	0.90	0.96	0.99	1.00	1.00	1.00	1.00	1.00	1.00	1.00
5	0.95	0.96	0.98	0.98	0.98	1.00	0.99	0.99	1.00	1.00
6	0.88	0.94	0.94	0.92	0.93	0.98	0.97	0.96	0.98	0.98
7	0.62	0.71	0.73	0.78	0.87	0.88	0.89	0.93	0.89	0.96
8	0.70	0.62	0.69	0.83	0.83	0.90	0.88	0.87	0.96	0.93
9	0.59	0.73	0.60	0.73	0.78	0.90	0.86	0.93	0.94	0.92
10	0.97	0.98	1.00	1.00	1.00	1.00	1.00	1.00	1.00	1.00
11	0.98	1.00	1.00	1.00	1.00	1.00	1.00	1.00	1.00	1.00
12	1.00	1.00	1.00	1.00	1.00	1.00	1.00	1.00	1.00	1.00
13	0.01	0.09	0.16	0.34	0.54	0.69	0.77	0.86	0.96	0.96
14	0.18	0.22	0.35	0.33	0.42	0.58	0.72	0.72	0.81	0.87
15	0.24	0.18	0.21	0.36	0.43	0.54	0.54	0.69	0.76	0.85
16	1.00	1.00	1.00	1.00	1.00	1.00	1.00	1.00	1.00	1.00
17	1.00	1.00	1.00	1.00	1.00	1.00	1.00	1.00	1.00	1.00
18	0.84	0.83	0.87	0.94	0.93	0.93	0.98	0.99	0.99	1.00
19	0.29	0.49	0.66	0.86	0.94	0.98	1.00	1.00	1.00	1.00
20	0.35	0.49	0.56	0.71	0.83	0.95	0.98	0.97	1.00	1.00
21	0.30	0.38	0.55	0.58	0.74	0.87	0.88	0.96	0.98	0.99
22	0.83	0.96	1.00	1.00	1.00	1.00	1.00	1.00	1.00	1.00
23	0.83	0.88	0.95	0.97	0.99	0.99	1.00	1.00	1.00	1.00
24	0.69	0.89	0.89	0.92	0.99	0.99	0.99	0.98	1.00	1.00
25	0.31	0.43	0.71	0.84	0.94	0.97	1.00	1.00	1.00	1.00
26	0.43	0.47	0.57	0.64	0.59	0.61	0.63	0.77	0.79	0.78
27	0.24	0.37	0.36	0.35	0.40	0.48	0.38	0.35	0.51	0.59
28	0.94	0.95	1.00	1.00	1.00	1.00	1.00	1.00	1.00	1.00
29	0.68	0.64	0.79	0.78	0.76	0.72	0.80	0.81	0.90	0.91
30	0.37	0.42	0.43	0.48	0.54	0.57	0.63	0.56	0.70	0.73
31	0.06	0.09	0.14	0.41	0.59	0.84	0.93	0.99	0.97	1.00
32	0.17	0.19	0.32	0.42	0.63	0.72	0.86	0.90	0.94	1.00
33	0.17	0.23	0.20	0.39	0.57	0.66	0.79	0.88	0.90	0.95
34	1.00	1.00	1.00	1.00	1.00	1.00	1.00	1.00	1.00	1.00
35	1.00	1.00	1.00	1.00	1.00	1.00	1.00	1.00	1.00	1.00
36	0.89	0.94	0.99	0.99	0.99	1.00	1.00	1.00	1.00	1.00
37	0.02	0.17	0.23	0.59	0.68	0.78	0.95	0.99	0.99	1.00
38	0.03	0.19	0.29	0.46	0.56	0.84	0.93	1.00	1.00	1.00
39	0.03	0.21	0.35	0.53	0.64	0.81	0.87	0.97	0.99	1.00
40	0.12	0.42	0.62	0.84	0.86	0.96	0.99	1.00	1.00	1.00
41	0.25	0.33	0.51	0.66	0.87	0.91	0.97	0.99	0.99	1.00
42	0.20	0.30	0.42	0.58	0.82	0.85	0.97	0.98	1.00	1.00

Table 2: POD according to each scenario and each k2 value, k1 = 0, alpha = 0.001

	k2=1	k2=2	k2=3	k2=4	k2=5	k2=6	k2=7	k2=8	k2=9	k2=10
1	0.11	0.21	0.36	0.41	0.67	0.67	0.82	0.94	0.90	0.94
2	0.24	0.30	0.29	0.45	0.54	0.64	0.61	0.76	0.77	0.84
3	0.23	0.30	0.30	0.27	0.37	0.50	0.53	0.59	0.66	0.76
4	0.92	0.97	0.97	1.00	1.00	1.00	1.00	1.00	1.00	1.00
5	0.94	0.97	0.97	0.97	1.00	0.97	0.99	1.00	1.00	1.00
6	0.91	0.89	0.88	0.92	0.92	0.98	0.98	0.97	0.98	1.00
7	0.47	0.56	0.69	0.71	0.72	0.79	0.90	0.87	0.94	0.93
8	0.58	0.52	0.65	0.62	0.72	0.81	0.84	0.88	0.92	0.95
9	0.46	0.56	0.65	0.71	0.77	0.84	0.88	0.85	0.86	0.95
10	0.97	0.99	1.00	1.00	1.00	1.00	1.00	1.00	1.00	1.00
11	0.98	0.99	1.00	0.99	1.00	1.00	1.00	1.00	1.00	1.00
12	1.00	1.00	1.00	1.00	1.00	1.00	1.00	1.00	1.00	1.00
13	0.02	0.04	0.16	0.34	0.56	0.61	0.85	0.91	0.91	0.98
14	0.25	0.22	0.28	0.33	0.39	0.55	0.68	0.80	0.88	0.88
15	0.31	0.29	0.30	0.35	0.51	0.48	0.52	0.67	0.71	0.83
16	1.00	1.00	1.00	1.00	1.00	1.00	1.00	1.00	1.00	1.00
17	1.00	1.00	1.00	1.00	1.00	1.00	1.00	1.00	1.00	1.00
18	0.82	0.84	0.92	0.92	0.92	0.96	0.97	0.97	1.00	1.00
19	0.35	0.48	0.66	0.86	0.94	0.98	1.00	1.00	1.00	1.00
20	0.39	0.50	0.60	0.73	0.81	0.92	0.96	0.99	1.00	0.99
21	0.32	0.24	0.46	0.59	0.74	0.82	0.93	0.98	0.97	0.99
22	0.79	0.94	0.98	1.00	1.00	1.00	1.00	1.00	1.00	1.00
23	0.79	0.87	0.93	0.97	0.97	1.00	1.00	1.00	1.00	1.00
24	0.67	0.79	0.86	0.91	0.97	0.97	0.99	1.00	1.00	1.00
25	0.24	0.46	0.55	0.86	0.96	0.96	1.00	1.00	1.00	1.00
26	0.42	0.46	0.50	0.50	0.60	0.62	0.68	0.69	0.70	0.75
27	0.38	0.32	0.31	0.33	0.45	0.36	0.43	0.41	0.60	0.60
28	0.91	0.96	0.99	1.00	1.00	1.00	1.00	1.00	1.00	1.00
29	0.58	0.68	0.69	0.68	0.80	0.83	0.74	0.90	0.90	0.93
30	0.37	0.45	0.46	0.38	0.53	0.59	0.47	0.61	0.71	0.74
31	0.07	0.12	0.20	0.38	0.63	0.84	0.91	0.98	0.98	0.99
32	0.17	0.24	0.38	0.47	0.57	0.73	0.83	0.91	0.93	0.98
33	0.20	0.23	0.31	0.39	0.55	0.61	0.78	0.86	0.95	0.98
34	1.00	1.00	1.00	1.00	1.00	1.00	1.00	1.00	1.00	1.00
35	0.99	1.00	1.00	1.00	1.00	1.00	1.00	1.00	1.00	1.00
36	0.93	0.99	0.99	0.95	0.99	0.98	1.00	1.00	1.00	1.00
37	0.06	0.15	0.26	0.49	0.76	0.79	0.90	0.97	0.99	1.00
38	0.08	0.16	0.27	0.52	0.65	0.85	0.90	0.98	1.00	1.00
39	0.12	0.14	0.28	0.46	0.63	0.81	0.88	0.99	0.99	1.00
40	0.18	0.45	0.53	0.78	0.94	0.95	0.99	1.00	0.99	1.00
41	0.21	0.25	0.54	0.66	0.78	0.93	0.99	0.98	1.00	1.00
42	0.22	0.29	0.50	0.54	0.76	0.89	0.97	0.97	1.00	1.00

Table 3: POD according to each scenario and each k2 value, k1 = 2, alpha = 0.001

	k2=1	k2=2	k2=3	k2=4	k2=5	k2=6	k2=7	k2=8	k2=9	k2=10
1	0.16	0.16	0.26	0.53	0.69	0.71	0.84	0.95	0.93	0.95
2	0.18	0.26	0.33	0.37	0.45	0.51	0.73	0.68	0.76	0.80
3	0.19	0.29	0.38	0.35	0.36	0.44	0.51	0.69	0.68	0.71
4	0.96	0.95	0.97	1.00	1.00	1.00	1.00	1.00	1.00	1.00
5	0.89	0.95	0.96	0.96	1.00	1.00	1.00	0.99	1.00	1.00
6	0.82	0.91	0.93	0.95	0.92	0.96	0.94	0.93	0.98	1.00
7	0.39	0.56	0.65	0.72	0.77	0.82	0.89	0.95	0.90	0.91
8	0.52	0.64	0.72	0.66	0.69	0.84	0.88	0.88	0.89	0.89
9	0.54	0.63	0.64	0.72	0.75	0.81	0.78	0.91	0.91	0.93
10	0.94	0.99	1.00	1.00	1.00	1.00	1.00	1.00	1.00	1.00
11	0.98	0.99	1.00	1.00	1.00	1.00	1.00	1.00	1.00	1.00
12	1.00	1.00	1.00	1.00	1.00	1.00	1.00	1.00	1.00	1.00
13	0.01	0.11	0.20	0.29	0.57	0.64	0.77	0.84	0.96	0.98
14	0.17	0.15	0.30	0.31	0.41	0.54	0.69	0.73	0.76	0.82
15	0.17	0.23	0.22	0.36	0.37	0.45	0.60	0.74	0.69	0.74
16	1.00	1.00	1.00	1.00	1.00	1.00	1.00	1.00	1.00	1.00
17	1.00	1.00	1.00	1.00	1.00	1.00	1.00	1.00	1.00	1.00
18	0.80	0.84	0.94	0.89	0.94	0.97	0.99	0.99	1.00	0.99
19	0.31	0.55	0.66	0.86	0.95	0.95	1.00	0.99	1.00	1.00
20	0.28	0.51	0.61	0.66	0.81	0.90	0.99	0.99	1.00	1.00
21	0.30	0.47	0.46	0.57	0.65	0.83	0.86	0.97	0.98	0.99
22	0.80	0.94	0.98	1.00	1.00	1.00	1.00	1.00	1.00	1.00
23	0.66	0.81	0.92	0.95	0.98	1.00	1.00	1.00	1.00	1.00
24	0.71	0.84	0.83	0.95	0.94	0.97	0.98	1.00	1.00	1.00
25	0.21	0.41	0.64	0.84	0.97	0.97	1.00	1.00	1.00	1.00
26	0.47	0.42	0.54	0.53	0.51	0.60	0.66	0.74	0.83	0.72
27	0.32	0.34	0.36	0.31	0.39	0.40	0.45	0.48	0.46	0.47
28	0.93	0.97	0.97	1.00	1.00	1.00	1.00	1.00	1.00	1.00
29	0.62	0.69	0.77	0.66	0.76	0.74	0.83	0.88	0.92	0.93
30	0.46	0.38	0.57	0.48	0.50	0.50	0.61	0.64	0.69	0.73
31	0.03	0.15	0.17	0.35	0.59	0.93	0.90	0.98	0.99	1.00
32	0.11	0.19	0.37	0.49	0.55	0.67	0.83	0.91	0.95	0.99
33	0.19	0.23	0.30	0.47	0.48	0.68	0.79	0.89	0.94	0.98
34	0.99	1.00	1.00	1.00	1.00	1.00	1.00	1.00	1.00	1.00
35	1.00	0.99	1.00	1.00	1.00	1.00	1.00	1.00	1.00	1.00
36	0.93	0.97	0.99	1.00	1.00	1.00	1.00	1.00	1.00	1.00
37	0.07	0.13	0.35	0.49	0.71	0.87	0.93	0.98	0.98	1.00
38	0.11	0.13	0.28	0.54	0.63	0.85	0.89	0.93	0.99	1.00
39	0.08	0.24	0.30	0.50	0.61	0.76	0.84	0.96	1.00	1.00
40	0.20	0.42	0.52	0.75	0.88	0.98	0.99	0.99	1.00	1.00
41	0.21	0.36	0.41	0.68	0.78	0.90	0.96	0.99	0.99	1.00
42	0.27	0.38	0.39	0.63	0.75	0.89	0.99	0.99	1.00	1.00

Table 4: POD according to each scenario and each k2 value, k1 = 3, alpha = 0.001

	k2=1	k2=2	k2=3	k2=4	k2=5	k2=6	k2=7	k2=8	k2=9	k2=10
1	0.14	0.30	0.35	0.44	0.63	0.65	0.87	0.95	0.98	0.96
2	0.16	0.27	0.40	0.42	0.45	0.60	0.67	0.70	0.81	0.78
3	0.23	0.34	0.30	0.30	0.36	0.44	0.63	0.62	0.60	0.67
4	0.89	0.93	0.98	0.99	1.00	1.00	1.00	1.00	1.00	1.00
5	0.91	0.93	0.94	0.98	0.98	1.00	0.97	1.00	0.99	1.00
6	0.87	0.91	0.92	0.92	0.91	0.92	0.94	1.00	0.97	0.98
7	0.44	0.58	0.57	0.71	0.79	0.74	0.87	0.86	0.93	0.95
8	0.46	0.48	0.63	0.64	0.77	0.75	0.78	0.90	0.88	0.92
9	0.43	0.44	0.68	0.60	0.74	0.80	0.83	0.89	0.91	0.91
10	0.98	0.96	0.99	1.00	1.00	1.00	1.00	1.00	1.00	1.00
11	0.99	1.00	0.99	1.00	1.00	1.00	1.00	1.00	1.00	1.00
12	1.00	1.00	1.00	1.00	1.00	1.00	1.00	1.00	1.00	1.00
13	0.03	0.15	0.22	0.33	0.56	0.71	0.76	0.88	0.96	0.97
14	0.09	0.15	0.35	0.43	0.51	0.60	0.59	0.83	0.83	0.88
15	0.30	0.27	0.29	0.34	0.43	0.42	0.56	0.72	0.75	0.86
16	1.00	1.00	1.00	1.00	1.00	1.00	1.00	1.00	1.00	1.00
17	1.00	1.00	1.00	1.00	1.00	1.00	1.00	1.00	1.00	1.00
18	0.81	0.84	0.91	0.90	0.90	0.98	0.98	0.97	1.00	1.00
19	0.30	0.58	0.69	0.84	0.94	0.98	0.99	0.99	1.00	1.00
20	0.34	0.36	0.59	0.67	0.85	0.90	0.98	0.99	1.00	0.99
21	0.31	0.42	0.48	0.58	0.78	0.82	0.91	0.94	0.99	1.00
22	0.80	0.94	0.99	1.00	1.00	1.00	1.00	1.00	1.00	1.00
23	0.79	0.86	0.90	0.95	0.97	0.99	0.99	1.00	1.00	1.00
24	0.77	0.82	0.84	0.91	0.96	0.98	0.98	0.99	1.00	1.00
25	0.25	0.49	0.59	0.84	0.93	0.99	0.99	1.00	1.00	1.00
26	0.47	0.53	0.52	0.56	0.57	0.63	0.67	0.68	0.72	0.85
27	0.31	0.33	0.32	0.33	0.35	0.42	0.42	0.43	0.46	0.56
28	0.90	0.96	1.00	0.99	1.00	1.00	1.00	1.00	1.00	1.00
29	0.69	0.67	0.67	0.73	0.74	0.83	0.80	0.83	0.85	0.91
30	0.51	0.40	0.42	0.42	0.46	0.58	0.61	0.59	0.64	0.70
31	0.06	0.10	0.32	0.30	0.59	0.78	0.91	0.95	1.00	1.00
32	0.13	0.21	0.44	0.39	0.64	0.73	0.88	0.89	0.96	0.96
33	0.16	0.28	0.31	0.35	0.49	0.58	0.75	0.86	0.94	0.97
34	0.99	1.00	1.00	1.00	1.00	1.00	1.00	1.00	1.00	1.00
35	0.99	1.00	1.00	1.00	1.00	1.00	1.00	1.00	1.00	1.00
36	0.91	0.90	0.95	0.97	0.99	1.00	1.00	1.00	1.00	1.00
37	0.02	0.07	0.25	0.44	0.69	0.84	0.93	0.98	0.97	1.00
38	0.03	0.15	0.17	0.38	0.69	0.85	0.93	0.98	1.00	1.00
39	0.12	0.14	0.35	0.31	0.58	0.77	0.86	0.95	0.99	1.00
40	0.17	0.24	0.58	0.69	0.81	0.90	0.99	0.99	1.00	1.00
41	0.21	0.25	0.58	0.65	0.73	0.92	0.98	1.00	1.00	1.00
42	0.18	0.25	0.49	0.63	0.81	0.86	0.98	0.99	1.00	1.00

Table 5: POD according to each scenario and each k2 value, k1 = 5, alpha = 0.001

	k2=1	k2=2	k2=3	k2=4	k2=5	k2=6	k2=7	k2=8	k2=9	k2=10
1	0.18	0.21	0.28	0.45	0.59	0.72	0.87	0.87	0.91	0.96
2	0.12	0.35	0.33	0.34	0.50	0.54	0.59	0.66	0.79	0.85
3	0.26	0.20	0.34	0.35	0.42	0.48	0.49	0.59	0.59	0.75
4	0.90	0.92	0.96	0.99	1.00	0.99	1.00	1.00	1.00	1.00
5	0.92	0.98	0.93	0.96	0.97	1.00	0.99	0.99	1.00	1.00
6	0.82	0.88	0.90	0.89	0.90	0.92	0.96	0.96	0.94	0.98
7	0.35	0.52	0.55	0.64	0.67	0.77	0.81	0.81	0.91	0.90
8	0.33	0.51	0.46	0.58	0.87	0.77	0.84	0.81	0.92	0.90
9	0.37	0.39	0.56	0.57	0.67	0.70	0.78	0.86	0.91	0.86
10	0.94	0.99	0.99	1.00	1.00	1.00	1.00	1.00	1.00	1.00
11	0.99	0.99	1.00	0.99	1.00	1.00	1.00	1.00	1.00	1.00
12	0.98	1.00	1.00	1.00	1.00	1.00	1.00	1.00	1.00	1.00
13	0.03	0.06	0.15	0.33	0.47	0.67	0.77	0.86	0.94	0.98
14	0.17	0.19	0.25	0.37	0.56	0.55	0.68	0.75	0.79	0.84
15	0.28	0.21	0.24	0.31	0.36	0.45	0.60	0.63	0.68	0.78
16	1.00	1.00	1.00	1.00	1.00	1.00	1.00	1.00	1.00	1.00
17	1.00	1.00	1.00	1.00	1.00	1.00	1.00	1.00	1.00	1.00
18	0.84	0.83	0.91	0.96	0.94	0.91	0.97	0.98	1.00	0.99
19	0.20	0.50	0.60	0.74	0.93	0.97	0.99	1.00	1.00	1.00
20	0.37	0.43	0.48	0.67	0.85	0.90	0.98	0.97	0.97	0.99
21	0.32	0.41	0.50	0.62	0.61	0.81	0.86	0.91	0.92	0.98
22	0.76	0.90	0.97	0.99	1.00	1.00	1.00	1.00	1.00	1.00
23	0.73	0.82	0.87	0.94	0.99	1.00	0.99	1.00	1.00	1.00
24	0.70	0.72	0.81	0.86	0.93	0.94	0.99	1.00	1.00	1.00
25	0.24	0.36	0.66	0.82	0.91	0.95	0.99	1.00	0.99	1.00
26	0.32	0.50	0.45	0.47	0.54	0.54	0.63	0.70	0.75	0.79
27	0.36	0.33	0.37	0.44	0.44	0.33	0.53	0.50	0.47	0.62
28	0.86	0.91	0.98	0.98	1.00	1.00	1.00	1.00	1.00	1.00
29	0.68	0.68	0.70	0.77	0.69	0.72	0.85	0.84	0.87	0.89
30	0.39	0.42	0.37	0.38	0.56	0.53	0.60	0.59	0.72	0.68
31	0.06	0.06	0.27	0.38	0.65	0.78	0.89	0.98	1.00	1.00
32	0.14	0.22	0.35	0.46	0.53	0.69	0.77	0.92	0.95	0.97
33	0.20	0.27	0.26	0.40	0.46	0.61	0.73	0.85	0.93	0.97
34	1.00	1.00	1.00	1.00	1.00	1.00	1.00	1.00	1.00	1.00
35	1.00	0.99	1.00	1.00	1.00	1.00	1.00	1.00	1.00	1.00
36	0.95	0.94	0.98	0.99	1.00	1.00	1.00	1.00	1.00	1.00
37	0.03	0.06	0.18	0.48	0.65	0.85	0.94	0.97	0.98	1.00
38	0.04	0.11	0.27	0.41	0.63	0.74	0.93	0.98	1.00	1.00
39	0.06	0.26	0.23	0.44	0.55	0.79	0.83	1.00	0.99	1.00
40	0.14	0.25	0.44	0.76	0.82	0.93	0.99	0.99	1.00	1.00
41	0.24	0.31	0.35	0.64	0.83	0.84	0.93	0.99	0.99	1.00
42	0.19	0.36	0.43	0.71	0.83	0.89	0.96	0.99	1.00	1.00

Table 6: POD according to each scenario and each k2 value, k1 = 10, alpha = 0.001

Overall performances of CUSUM Rossi algorithm ($\alpha = 0.01$)

	FPR k1=0	FPR k1=2	FPR k1=3	FPR k1=5	FPR k1=10
1	0.02	0.02	0.02	0.02	0.02
2	0.07	0.07	0.07	0.07	0.06
3	0.17	0.17	0.17	0.16	0.16
4	0.78	0.77	0.76	0.75	0.72
5	0.89	0.88	0.88	0.87	0.83
6	0.85	0.84	0.84	0.84	0.81
7	0.04	0.04	0.04	0.03	0.03
8	0.04	0.04	0.03	0.03	0.03
9	0.05	0.04	0.04	0.04	0.03
10	0.88	0.87	0.86	0.86	0.83
11	0.93	0.93	0.93	0.92	0.91
12	0.98	0.98	0.98	0.97	0.96
13	0.00	0.00	0.00	0.00	0.00
14	0.07	0.06	0.06	0.06	0.05
15	0.16	0.16	0.16	0.16	0.16
16	1.00	1.00	1.00	1.00	1.00
17	1.00	0.99	0.99	1.00	0.99
18	0.76	0.76	0.76	0.75	0.75
19	0.14	0.13	0.13	0.13	0.12
20	0.17	0.17	0.16	0.16	0.15
21	0.17	0.17	0.17	0.16	0.15
22	0.59	0.57	0.56	0.55	0.51
23	0.69	0.68	0.66	0.65	0.62
24	0.67	0.66	0.65	0.64	0.61
25	0.11	0.10	0.10	0.10	0.09
26	0.37	0.36	0.36	0.36	0.35
27	0.28	0.28	0.27	0.27	0.27
28	0.78	0.77	0.76	0.75	0.71
29	0.61	0.61	0.60	0.60	0.59
30	0.38	0.38	0.37	0.37	0.37
31	0.01	0.01	0.01	0.01	0.01
32	0.06	0.05	0.05	0.05	0.05
33	0.11	0.11	0.11	0.11	0.10
34	0.98	0.98	0.98	0.98	0.98
35	0.98	0.98	0.98	0.98	0.97
36	0.86	0.85	0.85	0.85	0.84
37	0.01	0.01	0.01	0.01	0.01
38	0.02	0.02	0.02	0.02	0.02
39	0.04	0.04	0.03	0.03	0.03
40	0.05	0.04	0.04	0.04	0.03
41	0.08	0.07	0.07	0.07	0.06
42	0.11	0.11	0.11	0.11	0.10

Table 7: FPR according to each scenario and each k1 value, alpha = 0.01

	k2=1	k2=2	k2=3	k2=4	k2=5	k2=6	k2=7	k2=8	k2=9	k2=10
1	0.17	0.29	0.30	0.43	0.63	0.70	0.83	0.94	0.97	0.96
2	0.25	0.29	0.33	0.45	0.54	0.52	0.69	0.81	0.84	0.90
3	0.31	0.30	0.28	0.39	0.38	0.45	0.57	0.63	0.70	0.75
4	0.90	0.96	0.99	1.00	1.00	1.00	1.00	1.00	1.00	1.00
5	0.95	0.96	0.98	0.98	0.98	1.00	0.99	0.99	1.00	1.00
6	0.88	0.94	0.94	0.92	0.93	0.98	0.97	0.96	0.98	0.98
7	0.62	0.71	0.73	0.78	0.87	0.88	0.89	0.93	0.89	0.96
8	0.70	0.62	0.69	0.83	0.83	0.90	0.88	0.87	0.96	0.93
9	0.59	0.73	0.60	0.73	0.78	0.90	0.86	0.93	0.94	0.92
10	0.97	0.98	1.00	1.00	1.00	1.00	1.00	1.00	1.00	1.00
11	0.98	1.00	1.00	1.00	1.00	1.00	1.00	1.00	1.00	1.00
12	1.00	1.00	1.00	1.00	1.00	1.00	1.00	1.00	1.00	1.00
13	0.01	0.09	0.16	0.34	0.54	0.69	0.77	0.86	0.96	0.96
14	0.18	0.22	0.35	0.33	0.42	0.58	0.72	0.72	0.81	0.87
15	0.24	0.18	0.21	0.36	0.43	0.54	0.54	0.69	0.76	0.85
16	1.00	1.00	1.00	1.00	1.00	1.00	1.00	1.00	1.00	1.00
17	1.00	1.00	1.00	1.00	1.00	1.00	1.00	1.00	1.00	1.00
18	0.84	0.83	0.87	0.94	0.93	0.93	0.98	0.99	0.99	1.00
19	0.29	0.49	0.66	0.86	0.94	0.98	1.00	1.00	1.00	1.00
20	0.35	0.49	0.56	0.71	0.83	0.95	0.98	0.97	1.00	1.00
21	0.30	0.38	0.55	0.58	0.74	0.87	0.88	0.96	0.98	0.99
22	0.83	0.96	1.00	1.00	1.00	1.00	1.00	1.00	1.00	1.00
23	0.83	0.88	0.95	0.97	0.99	0.99	1.00	1.00	1.00	1.00
24	0.69	0.89	0.89	0.92	0.99	0.99	0.99	0.98	1.00	1.00
25	0.31	0.43	0.71	0.84	0.94	0.97	1.00	1.00	1.00	1.00
26	0.43	0.47	0.57	0.64	0.59	0.61	0.63	0.77	0.79	0.78
27	0.24	0.37	0.36	0.35	0.40	0.48	0.38	0.35	0.51	0.59
28	0.94	0.95	1.00	1.00	1.00	1.00	1.00	1.00	1.00	1.00
29	0.68	0.64	0.79	0.78	0.76	0.72	0.80	0.81	0.90	0.91
30	0.37	0.42	0.43	0.48	0.54	0.57	0.63	0.56	0.70	0.73
31	0.06	0.09	0.14	0.41	0.59	0.84	0.93	0.99	0.97	1.00
32	0.17	0.19	0.32	0.42	0.63	0.72	0.86	0.90	0.94	1.00
33	0.17	0.23	0.20	0.39	0.57	0.66	0.79	0.88	0.90	0.95
34	1.00	1.00	1.00	1.00	1.00	1.00	1.00	1.00	1.00	1.00
35	1.00	1.00	1.00	1.00	1.00	1.00	1.00	1.00	1.00	1.00
36	0.89	0.94	0.99	0.99	0.99	1.00	1.00	1.00	1.00	1.00
37	0.02	0.17	0.23	0.59	0.68	0.78	0.95	0.99	0.99	1.00
38	0.03	0.19	0.29	0.46	0.56	0.84	0.93	1.00	1.00	1.00
39	0.03	0.21	0.35	0.53	0.64	0.81	0.87	0.97	0.99	1.00
40	0.12	0.42	0.62	0.84	0.86	0.96	0.99	1.00	1.00	1.00
41	0.25	0.33	0.51	0.66	0.87	0.91	0.97	0.99	0.99	1.00
42	0.20	0.30	0.42	0.58	0.82	0.85	0.97	0.98	1.00	1.00

Table 8: POD according to each scenario and each k2 value, k1 = 0, alpha = 0.01

	k2=1	k2=2	k2=3	k2=4	k2=5	k2=6	k2=7	k2=8	k2=9	k2=10
1	0.11	0.21	0.36	0.41	0.67	0.67	0.82	0.94	0.90	0.94
2	0.24	0.30	0.29	0.45	0.54	0.64	0.61	0.76	0.77	0.84
3	0.23	0.30	0.30	0.27	0.37	0.50	0.53	0.59	0.66	0.76
4	0.92	0.97	0.97	1.00	1.00	1.00	1.00	1.00	1.00	1.00
5	0.94	0.97	0.97	0.97	1.00	0.97	0.99	1.00	1.00	1.00
6	0.91	0.89	0.88	0.92	0.92	0.98	0.98	0.97	0.98	1.00
7	0.47	0.56	0.69	0.71	0.72	0.79	0.90	0.87	0.94	0.93
8	0.58	0.52	0.65	0.62	0.72	0.81	0.84	0.88	0.92	0.95
9	0.46	0.56	0.65	0.71	0.77	0.84	0.88	0.85	0.86	0.95
10	0.97	0.99	1.00	1.00	1.00	1.00	1.00	1.00	1.00	1.00
11	0.98	0.99	1.00	0.99	1.00	1.00	1.00	1.00	1.00	1.00
12	1.00	1.00	1.00	1.00	1.00	1.00	1.00	1.00	1.00	1.00
13	0.02	0.04	0.16	0.34	0.56	0.61	0.85	0.91	0.91	0.98
14	0.25	0.22	0.28	0.33	0.39	0.55	0.68	0.80	0.88	0.88
15	0.31	0.29	0.30	0.35	0.51	0.48	0.52	0.67	0.71	0.83
16	1.00	1.00	1.00	1.00	1.00	1.00	1.00	1.00	1.00	1.00
17	1.00	1.00	1.00	1.00	1.00	1.00	1.00	1.00	1.00	1.00
18	0.82	0.84	0.92	0.92	0.92	0.96	0.97	0.97	1.00	1.00
19	0.35	0.48	0.66	0.86	0.94	0.98	1.00	1.00	1.00	1.00
20	0.39	0.50	0.60	0.73	0.81	0.92	0.96	0.99	1.00	0.99
21	0.32	0.24	0.46	0.59	0.74	0.82	0.93	0.98	0.97	0.99
22	0.79	0.94	0.98	1.00	1.00	1.00	1.00	1.00	1.00	1.00
23	0.79	0.87	0.93	0.97	0.97	1.00	1.00	1.00	1.00	1.00
24	0.67	0.79	0.86	0.91	0.97	0.97	0.99	1.00	1.00	1.00
25	0.24	0.46	0.55	0.86	0.96	0.96	1.00	1.00	1.00	1.00
26	0.42	0.46	0.50	0.50	0.60	0.62	0.68	0.69	0.70	0.75
27	0.38	0.32	0.31	0.33	0.45	0.36	0.43	0.41	0.60	0.60
28	0.91	0.96	0.99	1.00	1.00	1.00	1.00	1.00	1.00	1.00
29	0.58	0.68	0.69	0.68	0.80	0.83	0.74	0.90	0.90	0.93
30	0.37	0.45	0.46	0.38	0.53	0.59	0.47	0.61	0.71	0.74
31	0.07	0.12	0.20	0.38	0.63	0.84	0.91	0.98	0.98	0.99
32	0.17	0.24	0.38	0.47	0.57	0.73	0.83	0.91	0.93	0.98
33	0.20	0.23	0.31	0.39	0.55	0.61	0.78	0.86	0.95	0.98
34	1.00	1.00	1.00	1.00	1.00	1.00	1.00	1.00	1.00	1.00
35	0.99	1.00	1.00	1.00	1.00	1.00	1.00	1.00	1.00	1.00
36	0.93	0.99	0.99	0.95	0.99	0.98	1.00	1.00	1.00	1.00
37	0.06	0.15	0.26	0.49	0.76	0.79	0.90	0.97	0.99	1.00
38	0.08	0.16	0.27	0.52	0.65	0.85	0.90	0.98	1.00	1.00
39	0.12	0.14	0.28	0.46	0.63	0.81	0.88	0.99	0.99	1.00
40	0.18	0.45	0.53	0.78	0.94	0.95	0.99	1.00	0.99	1.00
41	0.21	0.25	0.54	0.66	0.78	0.93	0.99	0.98	1.00	1.00
42	0.22	0.29	0.50	0.54	0.76	0.89	0.97	0.97	1.00	1.00

Table 9: POD according to each scenario and each k2 value, k1 = 2, alpha = 0.01

	k2=1	k2=2	k2=3	k2=4	k2=5	k2=6	k2=7	k2=8	k2=9	k2=10
1	0.16	0.16	0.26	0.53	0.69	0.71	0.84	0.95	0.93	0.95
2	0.18	0.26	0.33	0.37	0.45	0.51	0.73	0.68	0.76	0.80
3	0.19	0.29	0.38	0.35	0.36	0.44	0.51	0.69	0.68	0.71
4	0.96	0.95	0.97	1.00	1.00	1.00	1.00	1.00	1.00	1.00
5	0.89	0.95	0.96	0.96	1.00	1.00	1.00	0.99	1.00	1.00
6	0.82	0.91	0.93	0.95	0.92	0.96	0.94	0.93	0.98	1.00
7	0.39	0.56	0.65	0.72	0.77	0.82	0.89	0.95	0.90	0.91
8	0.52	0.64	0.72	0.66	0.69	0.84	0.88	0.88	0.89	0.89
9	0.54	0.63	0.64	0.72	0.75	0.81	0.78	0.91	0.91	0.93
10	0.94	0.99	1.00	1.00	1.00	1.00	1.00	1.00	1.00	1.00
11	0.98	0.99	1.00	1.00	1.00	1.00	1.00	1.00	1.00	1.00
12	1.00	1.00	1.00	1.00	1.00	1.00	1.00	1.00	1.00	1.00
13	0.01	0.11	0.20	0.29	0.57	0.64	0.77	0.84	0.96	0.98
14	0.17	0.15	0.30	0.31	0.41	0.54	0.69	0.73	0.76	0.82
15	0.17	0.23	0.22	0.36	0.37	0.45	0.60	0.74	0.69	0.74
16	1.00	1.00	1.00	1.00	1.00	1.00	1.00	1.00	1.00	1.00
17	1.00	1.00	1.00	1.00	1.00	1.00	1.00	1.00	1.00	1.00
18	0.80	0.84	0.94	0.89	0.94	0.97	0.99	0.99	1.00	0.99
19	0.31	0.55	0.66	0.86	0.95	0.95	1.00	0.99	1.00	1.00
20	0.28	0.51	0.61	0.66	0.81	0.90	0.99	0.99	1.00	1.00
21	0.30	0.47	0.46	0.57	0.65	0.83	0.86	0.97	0.98	0.99
22	0.80	0.94	0.98	1.00	1.00	1.00	1.00	1.00	1.00	1.00
23	0.66	0.81	0.92	0.95	0.98	1.00	1.00	1.00	1.00	1.00
24	0.71	0.84	0.83	0.95	0.94	0.97	0.98	1.00	1.00	1.00
25	0.21	0.41	0.64	0.84	0.97	0.97	1.00	1.00	1.00	1.00
26	0.47	0.42	0.54	0.53	0.51	0.60	0.66	0.74	0.83	0.72
27	0.32	0.34	0.36	0.31	0.39	0.40	0.45	0.48	0.46	0.47
28	0.93	0.97	0.97	1.00	1.00	1.00	1.00	1.00	1.00	1.00
29	0.62	0.69	0.77	0.66	0.76	0.74	0.83	0.88	0.92	0.93
30	0.46	0.38	0.57	0.48	0.50	0.50	0.61	0.64	0.69	0.73
31	0.03	0.15	0.17	0.35	0.59	0.93	0.90	0.98	0.99	1.00
32	0.11	0.19	0.37	0.49	0.55	0.67	0.83	0.91	0.95	0.99
33	0.19	0.23	0.30	0.47	0.48	0.68	0.79	0.89	0.94	0.98
34	0.99	1.00	1.00	1.00	1.00	1.00	1.00	1.00	1.00	1.00
35	1.00	0.99	1.00	1.00	1.00	1.00	1.00	1.00	1.00	1.00
36	0.93	0.97	0.99	1.00	1.00	1.00	1.00	1.00	1.00	1.00
37	0.07	0.13	0.35	0.49	0.71	0.87	0.93	0.98	0.98	1.00
38	0.11	0.13	0.28	0.54	0.63	0.85	0.89	0.93	0.99	1.00
39	0.08	0.24	0.30	0.50	0.61	0.76	0.84	0.96	1.00	1.00
40	0.20	0.42	0.52	0.75	0.88	0.98	0.99	0.99	1.00	1.00
41	0.21	0.36	0.41	0.68	0.78	0.90	0.96	0.99	0.99	1.00
42	0.27	0.38	0.39	0.63	0.75	0.89	0.99	0.99	1.00	1.00

Table 10: POD according to each scenario and each k2 value, k1 = 3, alpha = 0.01

	k2=1	k2=2	k2=3	k2=4	k2=5	k2=6	k2=7	k2=8	k2=9	k2=10
1	0.14	0.30	0.35	0.44	0.63	0.65	0.87	0.95	0.98	0.96
2	0.16	0.27	0.40	0.42	0.45	0.60	0.67	0.70	0.81	0.78
3	0.23	0.34	0.30	0.30	0.36	0.44	0.63	0.62	0.60	0.67
4	0.89	0.93	0.98	0.99	1.00	1.00	1.00	1.00	1.00	1.00
5	0.91	0.93	0.94	0.98	0.98	1.00	0.97	1.00	0.99	1.00
6	0.87	0.91	0.92	0.92	0.91	0.92	0.94	1.00	0.97	0.98
7	0.44	0.58	0.57	0.71	0.79	0.74	0.87	0.86	0.93	0.95
8	0.46	0.48	0.63	0.64	0.77	0.75	0.78	0.90	0.88	0.92
9	0.43	0.44	0.68	0.60	0.74	0.80	0.83	0.89	0.91	0.91
10	0.98	0.96	0.99	1.00	1.00	1.00	1.00	1.00	1.00	1.00
11	0.99	1.00	0.99	1.00	1.00	1.00	1.00	1.00	1.00	1.00
12	1.00	1.00	1.00	1.00	1.00	1.00	1.00	1.00	1.00	1.00
13	0.03	0.15	0.22	0.33	0.56	0.71	0.76	0.88	0.96	0.97
14	0.09	0.15	0.35	0.43	0.51	0.60	0.59	0.83	0.83	0.88
15	0.30	0.27	0.29	0.34	0.43	0.42	0.56	0.72	0.75	0.86
16	1.00	1.00	1.00	1.00	1.00	1.00	1.00	1.00	1.00	1.00
17	1.00	1.00	1.00	1.00	1.00	1.00	1.00	1.00	1.00	1.00
18	0.81	0.84	0.91	0.90	0.90	0.98	0.98	0.97	1.00	1.00
19	0.30	0.58	0.69	0.84	0.94	0.98	0.99	0.99	1.00	1.00
20	0.34	0.36	0.59	0.67	0.85	0.90	0.98	0.99	1.00	0.99
21	0.31	0.42	0.48	0.58	0.78	0.82	0.91	0.94	0.99	1.00
22	0.80	0.94	0.99	1.00	1.00	1.00	1.00	1.00	1.00	1.00
23	0.79	0.86	0.90	0.95	0.97	0.99	0.99	1.00	1.00	1.00
24	0.77	0.82	0.84	0.91	0.96	0.98	0.98	0.99	1.00	1.00
25	0.25	0.49	0.59	0.84	0.93	0.99	0.99	1.00	1.00	1.00
26	0.47	0.53	0.52	0.56	0.57	0.63	0.67	0.68	0.72	0.85
27	0.31	0.33	0.32	0.33	0.35	0.42	0.42	0.43	0.46	0.56
28	0.90	0.96	1.00	0.99	1.00	1.00	1.00	1.00	1.00	1.00
29	0.69	0.67	0.67	0.73	0.74	0.83	0.80	0.83	0.85	0.91
30	0.51	0.40	0.42	0.42	0.46	0.58	0.61	0.59	0.64	0.70
31	0.06	0.10	0.32	0.30	0.59	0.78	0.91	0.95	1.00	1.00
32	0.13	0.21	0.44	0.39	0.64	0.73	0.88	0.89	0.96	0.96
33	0.16	0.28	0.31	0.35	0.49	0.58	0.75	0.86	0.94	0.97
34	0.99	1.00	1.00	1.00	1.00	1.00	1.00	1.00	1.00	1.00
35	0.99	1.00	1.00	1.00	1.00	1.00	1.00	1.00	1.00	1.00
36	0.91	0.90	0.95	0.97	0.99	1.00	1.00	1.00	1.00	1.00
37	0.02	0.07	0.25	0.44	0.69	0.84	0.93	0.98	0.97	1.00
38	0.03	0.15	0.17	0.38	0.69	0.85	0.93	0.98	1.00	1.00
39	0.12	0.14	0.35	0.31	0.58	0.77	0.86	0.95	0.99	1.00
40	0.17	0.24	0.58	0.69	0.81	0.90	0.99	0.99	1.00	1.00
41	0.21	0.25	0.58	0.65	0.73	0.92	0.98	1.00	1.00	1.00
42	0.18	0.25	0.49	0.63	0.81	0.86	0.98	0.99	1.00	1.00

Table 11: POD according to each scenario and each k2 value, k1 = 5, alpha = 0.01

	k2=1	k2=2	k2=3	k2=4	k2=5	k2=6	k2=7	k2=8	k2=9	k2=10
1	0.18	0.21	0.28	0.45	0.59	0.72	0.87	0.87	0.91	0.96
2	0.12	0.35	0.33	0.34	0.50	0.54	0.59	0.66	0.79	0.85
3	0.26	0.20	0.34	0.35	0.42	0.48	0.49	0.59	0.59	0.75
4	0.90	0.92	0.96	0.99	1.00	0.99	1.00	1.00	1.00	1.00
5	0.92	0.98	0.93	0.96	0.97	1.00	0.99	0.99	1.00	1.00
6	0.82	0.88	0.90	0.89	0.90	0.92	0.96	0.96	0.94	0.98
7	0.35	0.52	0.55	0.64	0.67	0.77	0.81	0.81	0.91	0.90
8	0.33	0.51	0.46	0.58	0.87	0.77	0.84	0.81	0.92	0.90
9	0.37	0.39	0.56	0.57	0.67	0.70	0.78	0.86	0.91	0.86
10	0.94	0.99	0.99	1.00	1.00	1.00	1.00	1.00	1.00	1.00
11	0.99	0.99	1.00	0.99	1.00	1.00	1.00	1.00	1.00	1.00
12	0.98	1.00	1.00	1.00	1.00	1.00	1.00	1.00	1.00	1.00
13	0.03	0.06	0.15	0.33	0.47	0.67	0.77	0.86	0.94	0.98
14	0.17	0.19	0.25	0.37	0.56	0.55	0.68	0.75	0.79	0.84
15	0.28	0.21	0.24	0.31	0.36	0.45	0.60	0.63	0.68	0.78
16	1.00	1.00	1.00	1.00	1.00	1.00	1.00	1.00	1.00	1.00
17	1.00	1.00	1.00	1.00	1.00	1.00	1.00	1.00	1.00	1.00
18	0.84	0.83	0.91	0.96	0.94	0.91	0.97	0.98	1.00	0.99
19	0.20	0.50	0.60	0.74	0.93	0.97	0.99	1.00	1.00	1.00
20	0.37	0.43	0.48	0.67	0.85	0.90	0.98	0.97	0.97	0.99
21	0.32	0.41	0.50	0.62	0.61	0.81	0.86	0.91	0.92	0.98
22	0.76	0.90	0.97	0.99	1.00	1.00	1.00	1.00	1.00	1.00
23	0.73	0.82	0.87	0.94	0.99	1.00	0.99	1.00	1.00	1.00
24	0.70	0.72	0.81	0.86	0.93	0.94	0.99	1.00	1.00	1.00
25	0.24	0.36	0.66	0.82	0.91	0.95	0.99	1.00	0.99	1.00
26	0.32	0.50	0.45	0.47	0.54	0.54	0.63	0.70	0.75	0.79
27	0.36	0.33	0.37	0.44	0.44	0.33	0.53	0.50	0.47	0.62
28	0.86	0.91	0.98	0.98	1.00	1.00	1.00	1.00	1.00	1.00
29	0.68	0.68	0.70	0.77	0.69	0.72	0.85	0.84	0.87	0.89
30	0.39	0.42	0.37	0.38	0.56	0.53	0.60	0.59	0.72	0.68
31	0.06	0.06	0.27	0.38	0.65	0.78	0.89	0.98	1.00	1.00
32	0.14	0.22	0.35	0.46	0.53	0.69	0.77	0.92	0.95	0.97
33	0.20	0.27	0.26	0.40	0.46	0.61	0.73	0.85	0.93	0.97
34	1.00	1.00	1.00	1.00	1.00	1.00	1.00	1.00	1.00	1.00
35	1.00	0.99	1.00	1.00	1.00	1.00	1.00	1.00	1.00	1.00
36	0.95	0.94	0.98	0.99	1.00	1.00	1.00	1.00	1.00	1.00
37	0.03	0.06	0.18	0.48	0.65	0.85	0.94	0.97	0.98	1.00
38	0.04	0.11	0.27	0.41	0.63	0.74	0.93	0.98	1.00	1.00
39	0.06	0.26	0.23	0.44	0.55	0.79	0.83	1.00	0.99	1.00
40	0.14	0.25	0.44	0.76	0.82	0.93	0.99	0.99	1.00	1.00
41	0.24	0.31	0.35	0.64	0.83	0.84	0.93	0.99	0.99	1.00
42	0.19	0.36	0.43	0.71	0.83	0.89	0.96	0.99	1.00	1.00

Table 12: POD according to each scenario and each k2 value, k1 = 10, alpha = 0.01

Overall performances of CUSUM Rossi algorithm ($\alpha = 0.05$)

	FPR k1=0	FPR k1=2	FPR k1=3	FPR k1=5	FPR k1=10
1	0.02	0.02	0.02	0.02	0.02
2	0.07	0.07	0.07	0.07	0.06
3	0.17	0.17	0.17	0.16	0.16
4	0.78	0.77	0.76	0.75	0.72
5	0.89	0.88	0.88	0.87	0.83
6	0.85	0.84	0.84	0.84	0.81
7	0.04	0.04	0.04	0.03	0.03
8	0.04	0.04	0.03	0.03	0.03
9	0.05	0.04	0.04	0.04	0.03
10	0.88	0.87	0.86	0.86	0.83
11	0.93	0.93	0.93	0.92	0.91
12	0.98	0.98	0.98	0.97	0.96
13	0.00	0.00	0.00	0.00	0.00
14	0.07	0.06	0.06	0.06	0.05
15	0.16	0.16	0.16	0.16	0.16
16	1.00	1.00	1.00	1.00	1.00
17	1.00	0.99	0.99	1.00	0.99
18	0.76	0.76	0.76	0.75	0.75
19	0.14	0.13	0.13	0.13	0.12
20	0.17	0.17	0.16	0.16	0.15
21	0.17	0.17	0.17	0.16	0.15
22	0.59	0.57	0.56	0.55	0.51
23	0.69	0.68	0.66	0.65	0.62
24	0.67	0.66	0.65	0.64	0.61
25	0.11	0.10	0.10	0.10	0.09
26	0.37	0.36	0.36	0.36	0.35
27	0.28	0.28	0.27	0.27	0.27
28	0.78	0.77	0.76	0.75	0.71
29	0.61	0.61	0.60	0.60	0.59
30	0.38	0.38	0.37	0.37	0.37
31	0.01	0.01	0.01	0.01	0.01
32	0.06	0.05	0.05	0.05	0.05
33	0.11	0.11	0.11	0.11	0.10
34	0.98	0.98	0.98	0.98	0.98
35	0.98	0.98	0.98	0.98	0.97
36	0.86	0.85	0.85	0.85	0.84
37	0.01	0.01	0.01	0.01	0.01
38	0.02	0.02	0.02	0.02	0.02
39	0.04	0.04	0.03	0.03	0.03
40	0.05	0.04	0.04	0.04	0.03
41	0.08	0.07	0.07	0.07	0.06
42	0.11	0.11	0.11	0.11	0.10

Table 13: FPR according to each scenario and each k1 value, alpha = 0.05

	k2=1	k2=2	k2=3	k2=4	k2=5	k2=6	k2=7	k2=8	k2=9	k2=10
1	0.17	0.29	0.30	0.43	0.63	0.70	0.83	0.94	0.97	0.96
2	0.25	0.29	0.33	0.45	0.54	0.52	0.69	0.81	0.84	0.90
3	0.31	0.30	0.28	0.39	0.38	0.45	0.57	0.63	0.70	0.75
4	0.90	0.96	0.99	1.00	1.00	1.00	1.00	1.00	1.00	1.00
5	0.95	0.96	0.98	0.98	0.98	1.00	0.99	0.99	1.00	1.00
6	0.88	0.94	0.94	0.92	0.93	0.98	0.97	0.96	0.98	0.98
7	0.62	0.71	0.73	0.78	0.87	0.88	0.89	0.93	0.89	0.96
8	0.70	0.62	0.69	0.83	0.83	0.90	0.88	0.87	0.96	0.93
9	0.59	0.73	0.60	0.73	0.78	0.90	0.86	0.93	0.94	0.92
10	0.97	0.98	1.00	1.00	1.00	1.00	1.00	1.00	1.00	1.00
11	0.98	1.00	1.00	1.00	1.00	1.00	1.00	1.00	1.00	1.00
12	1.00	1.00	1.00	1.00	1.00	1.00	1.00	1.00	1.00	1.00
13	0.01	0.09	0.16	0.34	0.54	0.69	0.77	0.86	0.96	0.96
14	0.18	0.22	0.35	0.33	0.42	0.58	0.72	0.72	0.81	0.87
15	0.24	0.18	0.21	0.36	0.43	0.54	0.54	0.69	0.76	0.85
16	1.00	1.00	1.00	1.00	1.00	1.00	1.00	1.00	1.00	1.00
17	1.00	1.00	1.00	1.00	1.00	1.00	1.00	1.00	1.00	1.00
18	0.84	0.83	0.87	0.94	0.93	0.93	0.98	0.99	0.99	1.00
19	0.29	0.49	0.66	0.86	0.94	0.98	1.00	1.00	1.00	1.00
20	0.35	0.49	0.56	0.71	0.83	0.95	0.98	0.97	1.00	1.00
21	0.30	0.38	0.55	0.58	0.74	0.87	0.88	0.96	0.98	0.99
22	0.83	0.96	1.00	1.00	1.00	1.00	1.00	1.00	1.00	1.00
23	0.83	0.88	0.95	0.97	0.99	0.99	1.00	1.00	1.00	1.00
24	0.69	0.89	0.89	0.92	0.99	0.99	0.99	0.98	1.00	1.00
25	0.31	0.43	0.71	0.84	0.94	0.97	1.00	1.00	1.00	1.00
26	0.43	0.47	0.57	0.64	0.59	0.61	0.63	0.77	0.79	0.78
27	0.24	0.37	0.36	0.35	0.40	0.48	0.38	0.35	0.51	0.59
28	0.94	0.95	1.00	1.00	1.00	1.00	1.00	1.00	1.00	1.00
29	0.68	0.64	0.79	0.78	0.76	0.72	0.80	0.81	0.90	0.91
30	0.37	0.42	0.43	0.48	0.54	0.57	0.63	0.56	0.70	0.73
31	0.06	0.09	0.14	0.41	0.59	0.84	0.93	0.99	0.97	1.00
32	0.17	0.19	0.32	0.42	0.63	0.72	0.86	0.90	0.94	1.00
33	0.17	0.23	0.20	0.39	0.57	0.66	0.79	0.88	0.90	0.95
34	1.00	1.00	1.00	1.00	1.00	1.00	1.00	1.00	1.00	1.00
35	1.00	1.00	1.00	1.00	1.00	1.00	1.00	1.00	1.00	1.00
36	0.89	0.94	0.99	0.99	0.99	1.00	1.00	1.00	1.00	1.00
37	0.02	0.17	0.23	0.59	0.68	0.78	0.95	0.99	0.99	1.00
38	0.03	0.19	0.29	0.46	0.56	0.84	0.93	1.00	1.00	1.00
39	0.03	0.21	0.35	0.53	0.64	0.81	0.87	0.97	0.99	1.00
40	0.12	0.42	0.62	0.84	0.86	0.96	0.99	1.00	1.00	1.00
41	0.25	0.33	0.51	0.66	0.87	0.91	0.97	0.99	0.99	1.00
42	0.20	0.30	0.42	0.58	0.82	0.85	0.97	0.98	1.00	1.00

Table 14: POD according to each scenario and each k2 value, k1 = 0, alpha = 0.05

	k2=1	k2=2	k2=3	k2=4	k2=5	k2=6	k2=7	k2=8	k2=9	k2=10
1	0.11	0.21	0.36	0.41	0.67	0.67	0.82	0.94	0.90	0.94
2	0.24	0.30	0.29	0.45	0.54	0.64	0.61	0.76	0.77	0.84
3	0.23	0.30	0.30	0.27	0.37	0.50	0.53	0.59	0.66	0.76
4	0.92	0.97	0.97	1.00	1.00	1.00	1.00	1.00	1.00	1.00
5	0.94	0.97	0.97	0.97	1.00	0.97	0.99	1.00	1.00	1.00
6	0.91	0.89	0.88	0.92	0.92	0.98	0.98	0.97	0.98	1.00
7	0.47	0.56	0.69	0.71	0.72	0.79	0.90	0.87	0.94	0.93
8	0.58	0.52	0.65	0.62	0.72	0.81	0.84	0.88	0.92	0.95
9	0.46	0.56	0.65	0.71	0.77	0.84	0.88	0.85	0.86	0.95
10	0.97	0.99	1.00	1.00	1.00	1.00	1.00	1.00	1.00	1.00
11	0.98	0.99	1.00	0.99	1.00	1.00	1.00	1.00	1.00	1.00
12	1.00	1.00	1.00	1.00	1.00	1.00	1.00	1.00	1.00	1.00
13	0.02	0.04	0.16	0.34	0.56	0.61	0.85	0.91	0.91	0.98
14	0.25	0.22	0.28	0.33	0.39	0.55	0.68	0.80	0.88	0.88
15	0.31	0.29	0.30	0.35	0.51	0.48	0.52	0.67	0.71	0.83
16	1.00	1.00	1.00	1.00	1.00	1.00	1.00	1.00	1.00	1.00
17	1.00	1.00	1.00	1.00	1.00	1.00	1.00	1.00	1.00	1.00
18	0.82	0.84	0.92	0.92	0.92	0.96	0.97	0.97	1.00	1.00
19	0.35	0.48	0.66	0.86	0.94	0.98	1.00	1.00	1.00	1.00
20	0.39	0.50	0.60	0.73	0.81	0.92	0.96	0.99	1.00	0.99
21	0.32	0.24	0.46	0.59	0.74	0.82	0.93	0.98	0.97	0.99
22	0.79	0.94	0.98	1.00	1.00	1.00	1.00	1.00	1.00	1.00
23	0.79	0.87	0.93	0.97	0.97	1.00	1.00	1.00	1.00	1.00
24	0.67	0.79	0.86	0.91	0.97	0.97	0.99	1.00	1.00	1.00
25	0.24	0.46	0.55	0.86	0.96	0.96	1.00	1.00	1.00	1.00
26	0.42	0.46	0.50	0.50	0.60	0.62	0.68	0.69	0.70	0.75
27	0.38	0.32	0.31	0.33	0.45	0.36	0.43	0.41	0.60	0.60
28	0.91	0.96	0.99	1.00	1.00	1.00	1.00	1.00	1.00	1.00
29	0.58	0.68	0.69	0.68	0.80	0.83	0.74	0.90	0.90	0.93
30	0.37	0.45	0.46	0.38	0.53	0.59	0.47	0.61	0.71	0.74
31	0.07	0.12	0.20	0.38	0.63	0.84	0.91	0.98	0.98	0.99
32	0.17	0.24	0.38	0.47	0.57	0.73	0.83	0.91	0.93	0.98
33	0.20	0.23	0.31	0.39	0.55	0.61	0.78	0.86	0.95	0.98
34	1.00	1.00	1.00	1.00	1.00	1.00	1.00	1.00	1.00	1.00
35	0.99	1.00	1.00	1.00	1.00	1.00	1.00	1.00	1.00	1.00
36	0.93	0.99	0.99	0.95	0.99	0.98	1.00	1.00	1.00	1.00
37	0.06	0.15	0.26	0.49	0.76	0.79	0.90	0.97	0.99	1.00
38	0.08	0.16	0.27	0.52	0.65	0.85	0.90	0.98	1.00	1.00
39	0.12	0.14	0.28	0.46	0.63	0.81	0.88	0.99	0.99	1.00
40	0.18	0.45	0.53	0.78	0.94	0.95	0.99	1.00	0.99	1.00
41	0.21	0.25	0.54	0.66	0.78	0.93	0.99	0.98	1.00	1.00
42	0.22	0.29	0.50	0.54	0.76	0.89	0.97	0.97	1.00	1.00

Table 15: POD according to each scenario and each k2 value, k1 = 2, alpha = 0.05

	k2=1	k2=2	k2=3	k2=4	k2=5	k2=6	k2=7	k2=8	k2=9	k2=10
1	0.16	0.16	0.26	0.53	0.69	0.71	0.84	0.95	0.93	0.95
2	0.18	0.26	0.33	0.37	0.45	0.51	0.73	0.68	0.76	0.80
3	0.19	0.29	0.38	0.35	0.36	0.44	0.51	0.69	0.68	0.71
4	0.96	0.95	0.97	1.00	1.00	1.00	1.00	1.00	1.00	1.00
5	0.89	0.95	0.96	0.96	1.00	1.00	1.00	0.99	1.00	1.00
6	0.82	0.91	0.93	0.95	0.92	0.96	0.94	0.93	0.98	1.00
7	0.39	0.56	0.65	0.72	0.77	0.82	0.89	0.95	0.90	0.91
8	0.52	0.64	0.72	0.66	0.69	0.84	0.88	0.88	0.89	0.89
9	0.54	0.63	0.64	0.72	0.75	0.81	0.78	0.91	0.91	0.93
10	0.94	0.99	1.00	1.00	1.00	1.00	1.00	1.00	1.00	1.00
11	0.98	0.99	1.00	1.00	1.00	1.00	1.00	1.00	1.00	1.00
12	1.00	1.00	1.00	1.00	1.00	1.00	1.00	1.00	1.00	1.00
13	0.01	0.11	0.20	0.29	0.57	0.64	0.77	0.84	0.96	0.98
14	0.17	0.15	0.30	0.31	0.41	0.54	0.69	0.73	0.76	0.82
15	0.17	0.23	0.22	0.36	0.37	0.45	0.60	0.74	0.69	0.74
16	1.00	1.00	1.00	1.00	1.00	1.00	1.00	1.00	1.00	1.00
17	1.00	1.00	1.00	1.00	1.00	1.00	1.00	1.00	1.00	1.00
18	0.80	0.84	0.94	0.89	0.94	0.97	0.99	0.99	1.00	0.99
19	0.31	0.55	0.66	0.86	0.95	0.95	1.00	0.99	1.00	1.00
20	0.28	0.51	0.61	0.66	0.81	0.90	0.99	0.99	1.00	1.00
21	0.30	0.47	0.46	0.57	0.65	0.83	0.86	0.97	0.98	0.99
22	0.80	0.94	0.98	1.00	1.00	1.00	1.00	1.00	1.00	1.00
23	0.66	0.81	0.92	0.95	0.98	1.00	1.00	1.00	1.00	1.00
24	0.71	0.84	0.83	0.95	0.94	0.97	0.98	1.00	1.00	1.00
25	0.21	0.41	0.64	0.84	0.97	0.97	1.00	1.00	1.00	1.00
26	0.47	0.42	0.54	0.53	0.51	0.60	0.66	0.74	0.83	0.72
27	0.32	0.34	0.36	0.31	0.39	0.40	0.45	0.48	0.46	0.47
28	0.93	0.97	0.97	1.00	1.00	1.00	1.00	1.00	1.00	1.00
29	0.62	0.69	0.77	0.66	0.76	0.74	0.83	0.88	0.92	0.93
30	0.46	0.38	0.57	0.48	0.50	0.50	0.61	0.64	0.69	0.73
31	0.03	0.15	0.17	0.35	0.59	0.93	0.90	0.98	0.99	1.00
32	0.11	0.19	0.37	0.49	0.55	0.67	0.83	0.91	0.95	0.99
33	0.19	0.23	0.30	0.47	0.48	0.68	0.79	0.89	0.94	0.98
34	0.99	1.00	1.00	1.00	1.00	1.00	1.00	1.00	1.00	1.00
35	1.00	0.99	1.00	1.00	1.00	1.00	1.00	1.00	1.00	1.00
36	0.93	0.97	0.99	1.00	1.00	1.00	1.00	1.00	1.00	1.00
37	0.07	0.13	0.35	0.49	0.71	0.87	0.93	0.98	0.98	1.00
38	0.11	0.13	0.28	0.54	0.63	0.85	0.89	0.93	0.99	1.00
39	0.08	0.24	0.30	0.50	0.61	0.76	0.84	0.96	1.00	1.00
40	0.20	0.42	0.52	0.75	0.88	0.98	0.99	0.99	1.00	1.00
41	0.21	0.36	0.41	0.68	0.78	0.90	0.96	0.99	0.99	1.00
42	0.27	0.38	0.39	0.63	0.75	0.89	0.99	0.99	1.00	1.00

Table 16: POD according to each scenario and each k2 value, k1 = 3, alpha = 0.05

	k2=1	k2=2	k2=3	k2=4	k2=5	k2=6	k2=7	k2=8	k2=9	k2=10
1	0.14	0.30	0.35	0.44	0.63	0.65	0.87	0.95	0.98	0.96
2	0.16	0.27	0.40	0.42	0.45	0.60	0.67	0.70	0.81	0.78
3	0.23	0.34	0.30	0.30	0.36	0.44	0.63	0.62	0.60	0.67
4	0.89	0.93	0.98	0.99	1.00	1.00	1.00	1.00	1.00	1.00
5	0.91	0.93	0.94	0.98	0.98	1.00	0.97	1.00	0.99	1.00
6	0.87	0.91	0.92	0.92	0.91	0.92	0.94	1.00	0.97	0.98
7	0.44	0.58	0.57	0.71	0.79	0.74	0.87	0.86	0.93	0.95
8	0.46	0.48	0.63	0.64	0.77	0.75	0.78	0.90	0.88	0.92
9	0.43	0.44	0.68	0.60	0.74	0.80	0.83	0.89	0.91	0.91
10	0.98	0.96	0.99	1.00	1.00	1.00	1.00	1.00	1.00	1.00
11	0.99	1.00	0.99	1.00	1.00	1.00	1.00	1.00	1.00	1.00
12	1.00	1.00	1.00	1.00	1.00	1.00	1.00	1.00	1.00	1.00
13	0.03	0.15	0.22	0.33	0.56	0.71	0.76	0.88	0.96	0.97
14	0.09	0.15	0.35	0.43	0.51	0.60	0.59	0.83	0.83	0.88
15	0.30	0.27	0.29	0.34	0.43	0.42	0.56	0.72	0.75	0.86
16	1.00	1.00	1.00	1.00	1.00	1.00	1.00	1.00	1.00	1.00
17	1.00	1.00	1.00	1.00	1.00	1.00	1.00	1.00	1.00	1.00
18	0.81	0.84	0.91	0.90	0.90	0.98	0.98	0.97	1.00	1.00
19	0.30	0.58	0.69	0.84	0.94	0.98	0.99	0.99	1.00	1.00
20	0.34	0.36	0.59	0.67	0.85	0.90	0.98	0.99	1.00	0.99
21	0.31	0.42	0.48	0.58	0.78	0.82	0.91	0.94	0.99	1.00
22	0.80	0.94	0.99	1.00	1.00	1.00	1.00	1.00	1.00	1.00
23	0.79	0.86	0.90	0.95	0.97	0.99	0.99	1.00	1.00	1.00
24	0.77	0.82	0.84	0.91	0.96	0.98	0.98	0.99	1.00	1.00
25	0.25	0.49	0.59	0.84	0.93	0.99	0.99	1.00	1.00	1.00
26	0.47	0.53	0.52	0.56	0.57	0.63	0.67	0.68	0.72	0.85
27	0.31	0.33	0.32	0.33	0.35	0.42	0.42	0.43	0.46	0.56
28	0.90	0.96	1.00	0.99	1.00	1.00	1.00	1.00	1.00	1.00
29	0.69	0.67	0.67	0.73	0.74	0.83	0.80	0.83	0.85	0.91
30	0.51	0.40	0.42	0.42	0.46	0.58	0.61	0.59	0.64	0.70
31	0.06	0.10	0.32	0.30	0.59	0.78	0.91	0.95	1.00	1.00
32	0.13	0.21	0.44	0.39	0.64	0.73	0.88	0.89	0.96	0.96
33	0.16	0.28	0.31	0.35	0.49	0.58	0.75	0.86	0.94	0.97
34	0.99	1.00	1.00	1.00	1.00	1.00	1.00	1.00	1.00	1.00
35	0.99	1.00	1.00	1.00	1.00	1.00	1.00	1.00	1.00	1.00
36	0.91	0.90	0.95	0.97	0.99	1.00	1.00	1.00	1.00	1.00
37	0.02	0.07	0.25	0.44	0.69	0.84	0.93	0.98	0.97	1.00
38	0.03	0.15	0.17	0.38	0.69	0.85	0.93	0.98	1.00	1.00
39	0.12	0.14	0.35	0.31	0.58	0.77	0.86	0.95	0.99	1.00
40	0.17	0.24	0.58	0.69	0.81	0.90	0.99	0.99	1.00	1.00
41	0.21	0.25	0.58	0.65	0.73	0.92	0.98	1.00	1.00	1.00
42	0.18	0.25	0.49	0.63	0.81	0.86	0.98	0.99	1.00	1.00

Table 17: POD according to each scenario and each k2 value, k1 = 5, alpha = 0.05

	k2=1	k2=2	k2=3	k2=4	k2=5	k2=6	k2=7	k2=8	k2=9	k2=10
1	0.18	0.21	0.28	0.45	0.59	0.72	0.87	0.87	0.91	0.96
2	0.12	0.35	0.33	0.34	0.50	0.54	0.59	0.66	0.79	0.85
3	0.26	0.20	0.34	0.35	0.42	0.48	0.49	0.59	0.59	0.75
4	0.90	0.92	0.96	0.99	1.00	0.99	1.00	1.00	1.00	1.00
5	0.92	0.98	0.93	0.96	0.97	1.00	0.99	0.99	1.00	1.00
6	0.82	0.88	0.90	0.89	0.90	0.92	0.96	0.96	0.94	0.98
7	0.35	0.52	0.55	0.64	0.67	0.77	0.81	0.81	0.91	0.90
8	0.33	0.51	0.46	0.58	0.87	0.77	0.84	0.81	0.92	0.90
9	0.37	0.39	0.56	0.57	0.67	0.70	0.78	0.86	0.91	0.86
10	0.94	0.99	0.99	1.00	1.00	1.00	1.00	1.00	1.00	1.00
11	0.99	0.99	1.00	0.99	1.00	1.00	1.00	1.00	1.00	1.00
12	0.98	1.00	1.00	1.00	1.00	1.00	1.00	1.00	1.00	1.00
13	0.03	0.06	0.15	0.33	0.47	0.67	0.77	0.86	0.94	0.98
14	0.17	0.19	0.25	0.37	0.56	0.55	0.68	0.75	0.79	0.84
15	0.28	0.21	0.24	0.31	0.36	0.45	0.60	0.63	0.68	0.78
16	1.00	1.00	1.00	1.00	1.00	1.00	1.00	1.00	1.00	1.00
17	1.00	1.00	1.00	1.00	1.00	1.00	1.00	1.00	1.00	1.00
18	0.84	0.83	0.91	0.96	0.94	0.91	0.97	0.98	1.00	0.99
19	0.20	0.50	0.60	0.74	0.93	0.97	0.99	1.00	1.00	1.00
20	0.37	0.43	0.48	0.67	0.85	0.90	0.98	0.97	0.97	0.99
21	0.32	0.41	0.50	0.62	0.61	0.81	0.86	0.91	0.92	0.98
22	0.76	0.90	0.97	0.99	1.00	1.00	1.00	1.00	1.00	1.00
23	0.73	0.82	0.87	0.94	0.99	1.00	0.99	1.00	1.00	1.00
24	0.70	0.72	0.81	0.86	0.93	0.94	0.99	1.00	1.00	1.00
25	0.24	0.36	0.66	0.82	0.91	0.95	0.99	1.00	0.99	1.00
26	0.32	0.50	0.45	0.47	0.54	0.54	0.63	0.70	0.75	0.79
27	0.36	0.33	0.37	0.44	0.44	0.33	0.53	0.50	0.47	0.62
28	0.86	0.91	0.98	0.98	1.00	1.00	1.00	1.00	1.00	1.00
29	0.68	0.68	0.70	0.77	0.69	0.72	0.85	0.84	0.87	0.89
30	0.39	0.42	0.37	0.38	0.56	0.53	0.60	0.59	0.72	0.68
31	0.06	0.06	0.27	0.38	0.65	0.78	0.89	0.98	1.00	1.00
32	0.14	0.22	0.35	0.46	0.53	0.69	0.77	0.92	0.95	0.97
33	0.20	0.27	0.26	0.40	0.46	0.61	0.73	0.85	0.93	0.97
34	1.00	1.00	1.00	1.00	1.00	1.00	1.00	1.00	1.00	1.00
35	1.00	0.99	1.00	1.00	1.00	1.00	1.00	1.00	1.00	1.00
36	0.95	0.94	0.98	0.99	1.00	1.00	1.00	1.00	1.00	1.00
37	0.03	0.06	0.18	0.48	0.65	0.85	0.94	0.97	0.98	1.00
38	0.04	0.11	0.27	0.41	0.63	0.74	0.93	0.98	1.00	1.00
39	0.06	0.26	0.23	0.44	0.55	0.79	0.83	1.00	0.99	1.00
40	0.14	0.25	0.44	0.76	0.82	0.93	0.99	0.99	1.00	1.00
41	0.24	0.31	0.35	0.64	0.83	0.84	0.93	0.99	0.99	1.00
42	0.19	0.36	0.43	0.71	0.83	0.89	0.96	0.99	1.00	1.00

Table 18: POD according to each scenario and each k2 value, k1 = 10, alpha = 0.05
