# Supplementary material for: Evaluation and comparison of statistical methods for early temporal detection of outbreaks: A simulation-based study
Source: PLoS One. 2017 Jul 17;12(7):e0181227. doi: 10.1371/journal.pone.0181227 (PMC5513450; doi:10.1371/journal.pone.0181227)
Supplement: S9 Appendix — (PDF) [file pone.0181227.s009.pdf]

Evaluation and Comparison of Statistical Methods for Early Temporal Detection of Outbreaks: a Simulation-Based Study

Appendix S9: Overall performances of CUSUM GLM algorithm ($\alpha = 0.001, 0.01$ and 0.05)

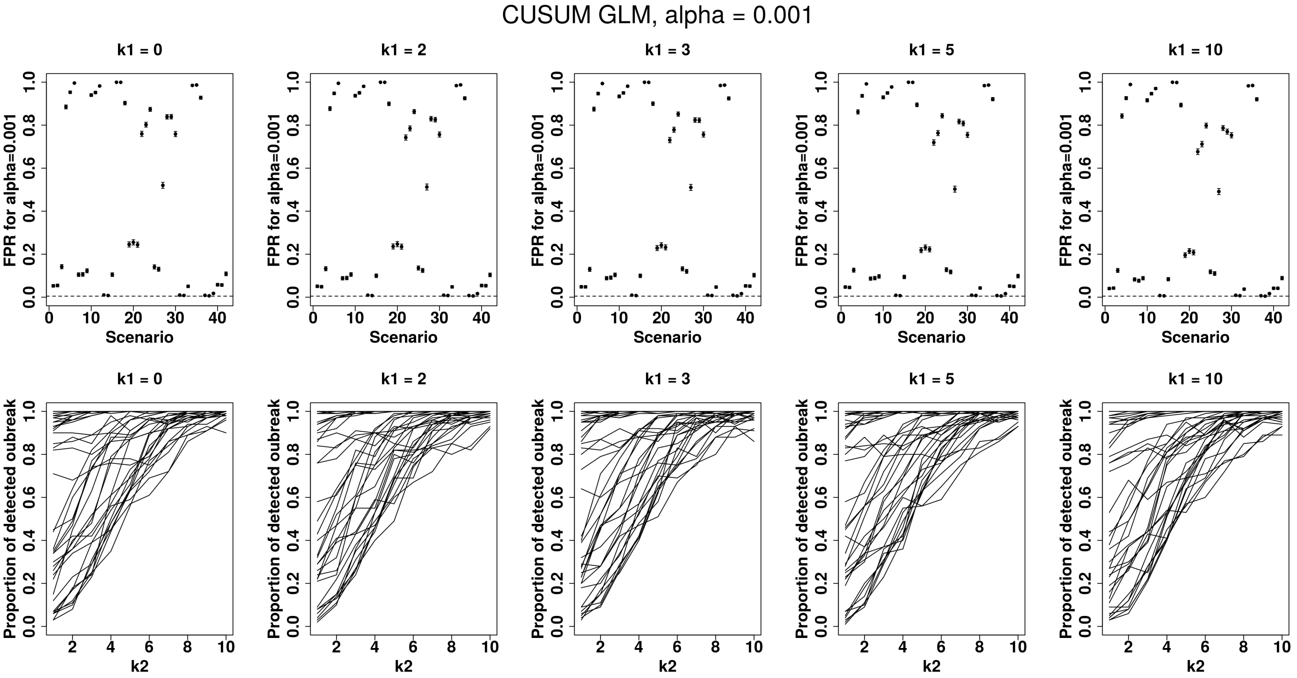

Figure 1: CUSUM GLM algorithm performances for $\alpha = 0.001$ by increasing past outbreak amplitude $k_1 = 0, 2, 3, 5$ or 10 with (i) on the first row: false positive rate for 42 simulated scenarios, (ii) on the second row: probability of detection for 42 simulated scenarios (each curve corresponding to a scenario) by increasing current outbreak amplitude $k_2 = 1$ to 10.

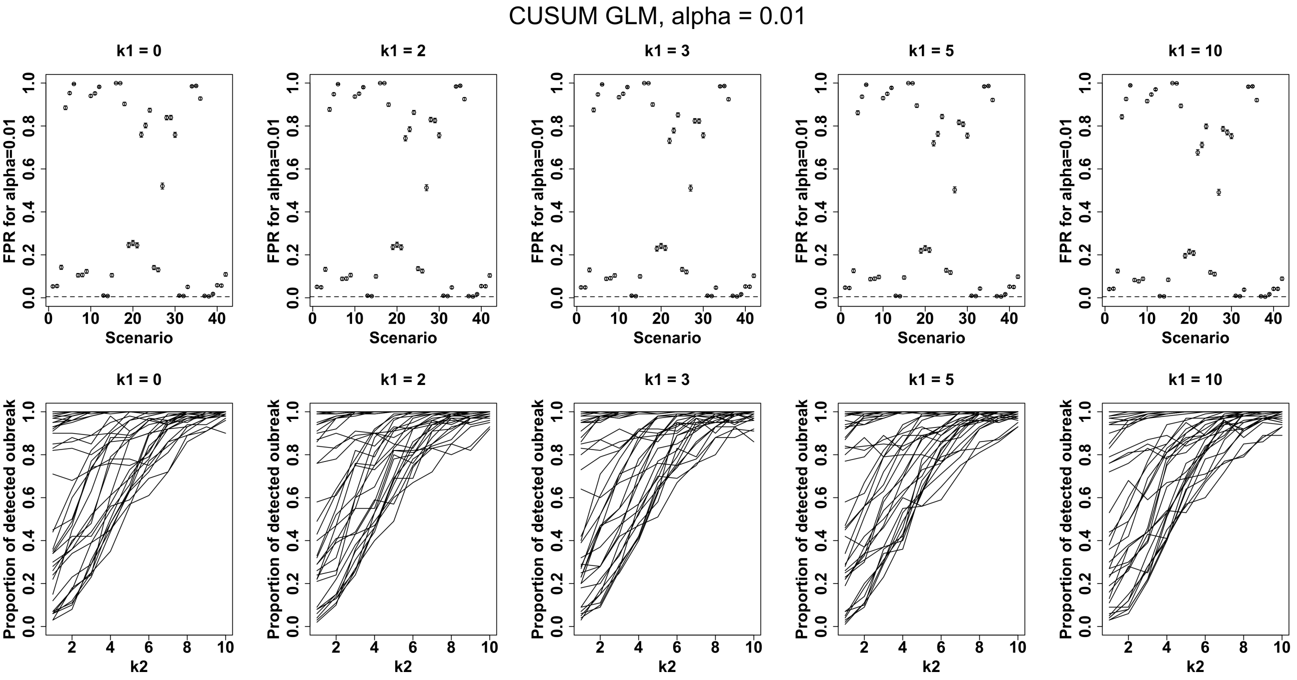

Figure 2: CUSUM GLM algorithm performances for $\alpha = 0.01$ by increasing past outbreak amplitude $k_1 = 0, 2, 3, 5$ or 10 with (i) on the first row: false positive rate for 42 simulated scenarios, (ii) on the second row: probability of detection for 42 simulated scenarios (each curve corresponding to a scenario) by increasing current outbreak amplitude $k_2 = 1$ to 10.

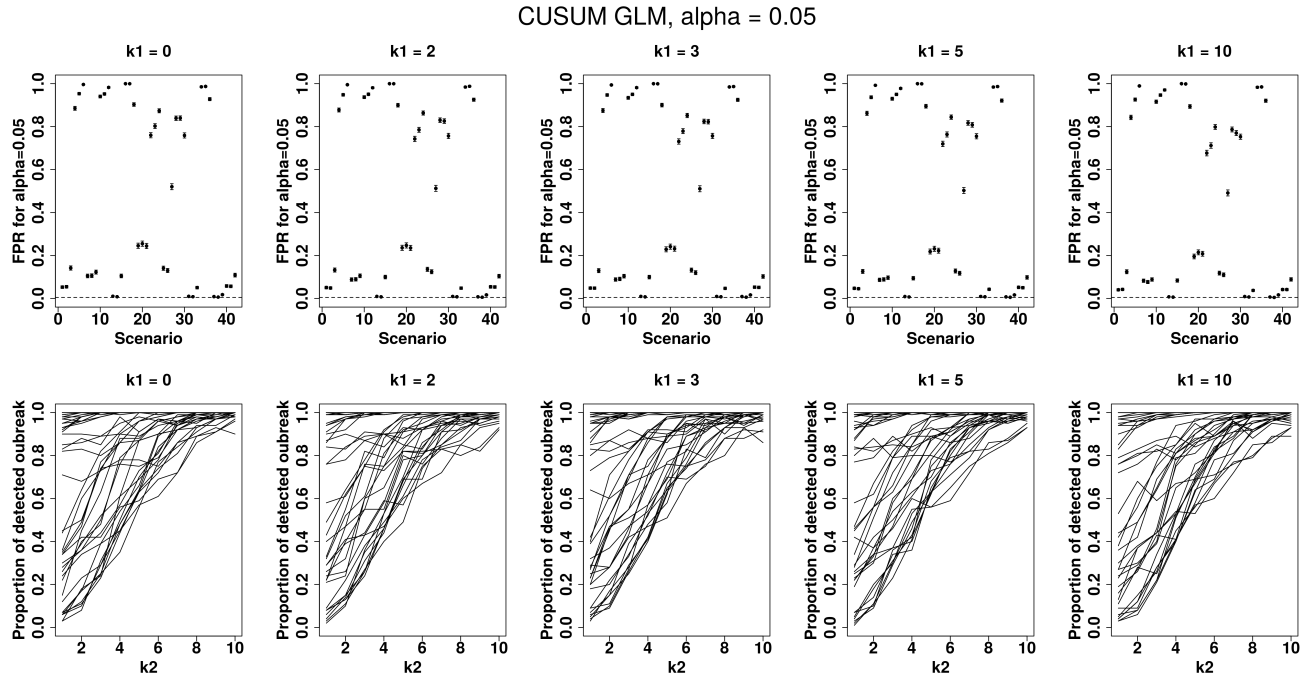

Figure 3: CUSUM GLM algorithm performances for $\alpha = 0.05$ by increasing past outbreak amplitude $k_1 = 0, 2, 3, 5$ or 10 with (i) on the first row: false positive rate for 42 simulated scenarios, (ii) on the second row: probability of detection for 42 simulated scenarios (each curve corresponding to a scenario) by increasing current outbreak amplitude $k_2 = 1$ to 10.

Overall performances of CUSUM GLM algorithm ($\alpha = 0.001$)

	FPR k1=0	FPR k1=2	FPR k1=3	FPR k1=5	FPR k1=10
1	0.05	0.05	0.05	0.05	0.04
2	0.05	0.05	0.05	0.05	0.04
3	0.14	0.13	0.13	0.13	0.12
4	0.88	0.88	0.87	0.86	0.84
5	0.95	0.95	0.95	0.94	0.93
6	1.00	0.99	0.99	0.99	0.99
7	0.10	0.09	0.09	0.09	0.08
8	0.11	0.09	0.09	0.09	0.08
9	0.12	0.11	0.10	0.10	0.09
10	0.94	0.94	0.93	0.93	0.92
11	0.95	0.95	0.95	0.95	0.95
12	0.98	0.98	0.98	0.98	0.97
13	0.01	0.01	0.01	0.01	0.01
14	0.01	0.01	0.01	0.01	0.01
15	0.10	0.10	0.10	0.09	0.08
16	1.00	1.00	1.00	1.00	1.00
17	1.00	1.00	1.00	1.00	1.00
18	0.90	0.90	0.90	0.89	0.89
19	0.25	0.24	0.23	0.22	0.20
20	0.26	0.25	0.24	0.23	0.21
21	0.24	0.24	0.23	0.22	0.21
22	0.76	0.74	0.73	0.72	0.68
23	0.80	0.78	0.78	0.76	0.71
24	0.87	0.86	0.85	0.84	0.80
25	0.14	0.14	0.13	0.13	0.12
26	0.13	0.12	0.12	0.12	0.11
27	0.52	0.51	0.51	0.50	0.49
28	0.84	0.83	0.82	0.82	0.79
29	0.84	0.83	0.82	0.81	0.77
30	0.76	0.76	0.76	0.75	0.75
31	0.01	0.01	0.01	0.01	0.01
32	0.01	0.01	0.01	0.01	0.01
33	0.05	0.05	0.05	0.04	0.04
34	0.98	0.98	0.98	0.98	0.98
35	0.99	0.99	0.99	0.99	0.98
36	0.93	0.93	0.92	0.92	0.92
37	0.01	0.01	0.01	0.01	0.01
38	0.01	0.01	0.01	0.01	0.00
39	0.02	0.02	0.02	0.02	0.02
40	0.06	0.05	0.05	0.05	0.04
41	0.06	0.05	0.05	0.05	0.04
42	0.11	0.10	0.10	0.10	0.09

Table 1: FPR according to each scenario and each k1 value, alpha = 0.001

	k2=1	k2=2	k2=3	k2=4	k2=5	k2=6	k2=7	k2=8	k2=9	k2=10
1	0.30	0.36	0.44	0.57	0.73	0.81	0.96	0.95	1.00	0.98
2	0.28	0.36	0.50	0.76	0.75	0.79	0.92	0.97	0.99	1.00
3	0.34	0.42	0.42	0.56	0.59	0.69	0.72	0.89	0.93	0.90
4	0.95	0.98	1.00	1.00	1.00	1.00	1.00	1.00	1.00	1.00
5	0.98	1.00	0.99	1.00	1.00	1.00	1.00	1.00	1.00	1.00
6	1.00	1.00	1.00	1.00	1.00	1.00	1.00	1.00	1.00	1.00
7	0.90	0.90	0.89	0.95	0.98	0.96	0.94	0.97	0.97	1.00
8	0.83	0.88	0.85	0.98	0.91	0.97	0.94	0.98	0.97	0.99
9	0.85	0.86	0.89	0.90	0.89	0.94	0.96	0.98	0.97	0.98
10	0.98	0.98	1.00	1.00	1.00	1.00	1.00	1.00	1.00	1.00
11	0.99	1.00	1.00	1.00	1.00	1.00	1.00	1.00	1.00	1.00
12	0.99	1.00	1.00	1.00	1.00	1.00	1.00	1.00	1.00	1.00
13	0.06	0.12	0.25	0.48	0.64	0.74	0.81	0.93	0.97	0.98
14	0.07	0.17	0.25	0.35	0.56	0.77	0.84	0.91	0.97	1.00
15	0.24	0.34	0.39	0.45	0.57	0.61	0.72	0.86	0.91	0.97
16	1.00	1.00	1.00	1.00	1.00	1.00	1.00	1.00	1.00	1.00
17	1.00	1.00	1.00	1.00	1.00	1.00	1.00	1.00	1.00	1.00
18	0.95	0.96	0.98	1.00	1.00	1.00	1.00	1.00	1.00	1.00
19	0.36	0.60	0.73	0.90	0.96	1.00	1.00	1.00	1.00	1.00
20	0.44	0.64	0.82	0.91	1.00	0.99	1.00	1.00	1.00	1.00
21	0.45	0.50	0.74	0.76	0.91	0.97	0.96	1.00	1.00	1.00
22	0.91	0.98	1.00	1.00	1.00	1.00	1.00	1.00	1.00	1.00
23	0.93	0.95	0.98	1.00	1.00	1.00	1.00	1.00	1.00	1.00
24	0.93	0.96	1.00	1.00	1.00	1.00	1.00	1.00	1.00	1.00
25	0.35	0.49	0.74	0.86	0.96	0.98	1.00	1.00	1.00	1.00
26	0.34	0.47	0.77	0.86	0.96	0.98	1.00	1.00	1.00	1.00
27	0.71	0.68	0.73	0.78	0.78	0.75	0.86	0.89	0.91	0.96
28	0.97	0.96	1.00	1.00	1.00	1.00	1.00	1.00	1.00	1.00
29	0.92	0.98	1.00	0.99	1.00	0.99	1.00	1.00	1.00	1.00
30	0.82	0.83	0.80	0.86	0.87	0.90	0.92	0.93	0.97	0.99
31	0.07	0.11	0.23	0.44	0.64	0.89	0.96	0.99	0.98	1.00
32	0.03	0.08	0.31	0.48	0.63	0.84	0.92	0.97	0.98	1.00
33	0.12	0.23	0.29	0.45	0.63	0.72	0.86	0.94	0.94	0.99
34	1.00	1.00	1.00	1.00	1.00	1.00	1.00	1.00	1.00	1.00
35	1.00	1.00	1.00	1.00	1.00	1.00	1.00	1.00	1.00	1.00
36	0.97	0.99	1.00	1.00	1.00	1.00	1.00	1.00	1.00	1.00
37	0.03	0.18	0.24	0.61	0.69	0.78	0.95	0.99	0.99	1.00
38	0.06	0.10	0.29	0.50	0.70	0.82	0.94	1.00	1.00	1.00
39	0.06	0.17	0.39	0.54	0.66	0.90	0.92	0.96	0.98	1.00
40	0.15	0.45	0.63	0.88	0.88	0.96	0.98	1.00	1.00	1.00
41	0.22	0.40	0.48	0.79	0.87	0.96	0.98	0.99	1.00	1.00
42	0.26	0.39	0.52	0.61	0.87	0.90	0.99	0.99	1.00	1.00

Table 2: POD according to each scenario and each k2 value, k1 = 0, alpha = 0.001

	k2=1	k2=2	k2=3	k2=4	k2=5	k2=6	k2=7	k2=8	k2=9	k2=10
1	0.22	0.40	0.55	0.55	0.80	0.76	0.88	0.97	0.94	0.96
2	0.29	0.41	0.43	0.58	0.82	0.81	0.85	0.93	0.95	0.98
3	0.29	0.39	0.44	0.59	0.57	0.75	0.83	0.80	0.84	0.93
4	0.99	0.99	1.00	1.00	1.00	1.00	1.00	1.00	1.00	1.00
5	0.99	1.00	1.00	1.00	1.00	1.00	1.00	1.00	1.00	1.00
6	1.00	1.00	1.00	1.00	1.00	1.00	1.00	1.00	1.00	1.00
7	0.76	0.78	0.87	0.90	0.91	0.93	0.95	0.96	0.95	0.98
8	0.87	0.90	0.87	0.84	0.93	0.92	0.94	0.95	0.98	0.99
9	0.76	0.84	0.89	0.90	0.88	0.93	0.96	0.99	0.97	0.99
10	1.00	1.00	1.00	1.00	1.00	1.00	1.00	1.00	1.00	1.00
11	0.99	0.99	1.00	0.99	1.00	1.00	1.00	1.00	1.00	1.00
12	1.00	1.00	1.00	1.00	1.00	1.00	1.00	1.00	1.00	1.00
13	0.03	0.11	0.26	0.46	0.69	0.69	0.91	0.92	0.97	0.99
14	0.08	0.13	0.27	0.40	0.49	0.74	0.85	0.91	0.97	0.99
15	0.24	0.26	0.43	0.47	0.60	0.67	0.72	0.85	0.82	0.92
16	1.00	1.00	1.00	1.00	1.00	1.00	1.00	1.00	1.00	1.00
17	1.00	1.00	1.00	1.00	1.00	1.00	1.00	1.00	1.00	1.00
18	0.95	0.98	0.99	1.00	1.00	1.00	1.00	1.00	1.00	1.00
19	0.43	0.62	0.75	0.91	0.99	0.99	1.00	1.00	1.00	1.00
20	0.49	0.64	0.78	0.92	0.98	1.00	1.00	1.00	1.00	1.00
21	0.40	0.47	0.76	0.75	0.91	0.98	0.99	0.99	0.99	1.00
22	0.86	0.97	1.00	1.00	1.00	1.00	1.00	1.00	1.00	1.00
23	0.90	0.92	1.00	1.00	1.00	1.00	1.00	1.00	1.00	1.00
24	0.91	0.97	0.98	1.00	1.00	1.00	1.00	1.00	1.00	1.00
25	0.32	0.52	0.61	0.88	0.97	0.98	1.00	1.00	1.00	1.00
26	0.33	0.55	0.82	0.79	0.96	1.00	1.00	1.00	1.00	1.00
27	0.58	0.61	0.75	0.73	0.82	0.82	0.83	0.85	0.93	0.95
28	0.94	0.97	0.99	1.00	1.00	1.00	1.00	1.00	1.00	1.00
29	0.94	0.98	0.99	0.99	1.00	1.00	1.00	1.00	1.00	1.00
30	0.84	0.83	0.91	0.90	0.85	0.83	0.96	0.94	0.98	0.99
31	0.08	0.13	0.24	0.46	0.68	0.86	0.92	0.99	1.00	1.00
32	0.04	0.14	0.31	0.44	0.69	0.80	0.94	0.97	0.97	0.98
33	0.09	0.22	0.38	0.49	0.61	0.78	0.84	0.87	0.97	0.97
34	1.00	1.00	1.00	1.00	1.00	1.00	1.00	1.00	1.00	1.00
35	1.00	1.00	1.00	1.00	1.00	1.00	1.00	1.00	1.00	1.00
36	1.00	0.99	1.00	1.00	1.00	1.00	1.00	1.00	1.00	1.00
37	0.08	0.15	0.26	0.53	0.78	0.80	0.91	0.98	0.99	1.00
38	0.02	0.10	0.36	0.53	0.70	0.92	0.97	0.98	0.99	1.00
39	0.06	0.15	0.35	0.46	0.66	0.84	0.94	0.98	0.98	1.00
40	0.22	0.47	0.55	0.81	0.92	0.95	0.99	1.00	0.99	1.00
41	0.21	0.24	0.60	0.75	0.84	0.97	0.99	0.98	1.00	1.00
42	0.26	0.32	0.60	0.65	0.83	0.92	0.98	1.00	1.00	1.00

Table 3: POD according to each scenario and each k2 value, k1 = 2, alpha = 0.001

	k2=1	k2=2	k2=3	k2=4	k2=5	k2=6	k2=7	k2=8	k2=9	k2=10
1	0.29	0.28	0.46	0.67	0.83	0.85	0.91	0.98	0.95	0.96
2	0.28	0.47	0.51	0.57	0.77	0.82	0.97	0.90	0.99	0.97
3	0.32	0.37	0.47	0.54	0.70	0.69	0.75	0.88	0.92	0.91
4	0.98	0.98	0.98	1.00	1.00	1.00	1.00	1.00	1.00	1.00
5	0.99	1.00	0.99	1.00	1.00	1.00	1.00	1.00	1.00	1.00
6	1.00	1.00	1.00	1.00	1.00	1.00	1.00	1.00	1.00	1.00
7	0.73	0.79	0.82	0.92	0.92	0.91	0.98	0.98	0.94	0.99
8	0.80	0.87	0.92	0.88	0.86	0.96	0.98	0.97	0.99	0.96
9	0.85	0.87	0.83	0.91	0.90	0.94	0.95	0.95	0.96	0.98
10	0.99	1.00	1.00	1.00	1.00	1.00	1.00	1.00	1.00	1.00
11	1.00	0.99	1.00	1.00	1.00	1.00	1.00	1.00	1.00	1.00
12	1.00	1.00	1.00	1.00	1.00	1.00	1.00	1.00	1.00	1.00
13	0.03	0.17	0.28	0.42	0.65	0.74	0.86	0.93	0.99	0.99
14	0.09	0.11	0.29	0.41	0.63	0.75	0.85	0.93	0.93	0.97
15	0.25	0.28	0.42	0.47	0.51	0.67	0.76	0.80	0.93	0.86
16	1.00	1.00	1.00	1.00	1.00	1.00	1.00	1.00	1.00	1.00
17	1.00	1.00	1.00	1.00	1.00	1.00	1.00	1.00	1.00	1.00
18	0.95	0.96	0.99	1.00	1.00	1.00	1.00	1.00	1.00	1.00
19	0.42	0.61	0.78	0.91	0.97	0.98	1.00	0.99	1.00	1.00
20	0.40	0.71	0.82	0.91	0.98	0.98	1.00	1.00	1.00	1.00
21	0.39	0.52	0.71	0.75	0.89	0.95	0.94	0.98	1.00	1.00
22	0.88	0.97	0.99	1.00	1.00	1.00	1.00	1.00	1.00	1.00
23	0.86	0.98	0.99	1.00	1.00	1.00	1.00	1.00	1.00	1.00
24	0.91	0.98	0.97	0.99	0.99	1.00	1.00	1.00	1.00	1.00
25	0.27	0.52	0.69	0.88	0.98	0.98	1.00	1.00	1.00	1.00
26	0.23	0.45	0.71	0.86	0.98	0.99	0.98	1.00	1.00	1.00
27	0.64	0.60	0.67	0.71	0.76	0.75	0.78	0.88	0.88	0.92
28	0.95	0.98	0.97	1.00	1.00	1.00	1.00	1.00	1.00	1.00
29	0.95	0.95	1.00	1.00	0.99	1.00	1.00	1.00	1.00	1.00
30	0.83	0.82	0.86	0.88	0.89	0.90	0.97	0.95	0.95	0.99
31	0.05	0.15	0.25	0.40	0.62	0.95	0.91	0.98	0.99	1.00
32	0.06	0.09	0.26	0.42	0.67	0.79	0.95	0.98	0.99	1.00
33	0.18	0.20	0.34	0.48	0.58	0.83	0.79	0.93	0.98	1.00
34	0.98	1.00	1.00	1.00	1.00	1.00	1.00	1.00	1.00	1.00
35	1.00	1.00	1.00	1.00	1.00	1.00	1.00	1.00	1.00	1.00
36	0.96	0.98	1.00	1.00	1.00	1.00	1.00	1.00	1.00	1.00
37	0.07	0.15	0.39	0.52	0.72	0.89	0.93	0.99	0.98	1.00
38	0.04	0.10	0.27	0.57	0.69	0.89	0.92	0.96	0.99	1.00
39	0.09	0.22	0.33	0.53	0.64	0.76	0.93	0.95	1.00	1.00
40	0.20	0.46	0.55	0.78	0.88	0.99	0.99	0.99	1.00	1.00
41	0.20	0.28	0.43	0.71	0.88	0.98	0.98	1.00	1.00	1.00
42	0.23	0.47	0.55	0.67	0.90	0.93	0.99	1.00	1.00	1.00

Table 4: POD according to each scenario and each k2 value, k1 = 3, alpha = 0.001

	k2=1	k2=2	k2=3	k2=4	k2=5	k2=6	k2=7	k2=8	k2=9	k2=10
1	0.29	0.42	0.50	0.61	0.73	0.76	0.90	0.99	0.99	0.97
2	0.19	0.37	0.54	0.63	0.70	0.86	0.88	0.96	0.95	0.98
3	0.42	0.37	0.46	0.60	0.56	0.65	0.75	0.81	0.86	0.93
4	0.96	0.94	0.99	1.00	1.00	1.00	1.00	1.00	1.00	1.00
5	0.99	0.98	1.00	1.00	1.00	1.00	1.00	1.00	1.00	1.00
6	0.98	1.00	1.00	1.00	1.00	1.00	1.00	1.00	1.00	1.00
7	0.80	0.88	0.79	0.89	0.89	0.86	0.93	0.95	0.98	1.00
8	0.84	0.82	0.87	0.90	0.93	0.89	0.95	0.99	0.95	0.98
9	0.77	0.79	0.89	0.82	0.89	0.91	0.96	0.96	0.96	0.97
10	0.99	0.99	0.99	1.00	1.00	1.00	1.00	1.00	1.00	1.00
11	0.99	1.00	1.00	1.00	1.00	1.00	1.00	1.00	1.00	1.00
12	1.00	1.00	1.00	1.00	1.00	1.00	1.00	1.00	1.00	1.00
13	0.04	0.18	0.31	0.43	0.68	0.79	0.82	0.92	0.97	0.98
14	0.02	0.11	0.30	0.49	0.63	0.76	0.85	0.93	0.98	0.96
15	0.25	0.31	0.37	0.55	0.56	0.59	0.72	0.86	0.87	0.93
16	1.00	1.00	1.00	1.00	1.00	1.00	1.00	1.00	1.00	1.00
17	1.00	1.00	1.00	1.00	1.00	1.00	1.00	1.00	1.00	1.00
18	0.94	0.96	0.97	1.00	1.00	1.00	1.00	1.00	1.00	1.00
19	0.47	0.67	0.82	0.92	0.98	0.98	0.99	0.99	1.00	1.00
20	0.45	0.56	0.79	0.94	0.98	0.99	0.99	1.00	1.00	1.00
21	0.46	0.56	0.63	0.79	0.92	0.93	0.97	0.99	1.00	1.00
22	0.87	0.98	1.00	1.00	1.00	1.00	1.00	1.00	1.00	1.00
23	0.88	0.98	1.00	1.00	1.00	0.99	1.00	1.00	1.00	1.00
24	0.93	0.94	0.97	1.00	1.00	1.00	1.00	1.00	1.00	1.00
25	0.34	0.56	0.66	0.85	0.96	0.99	0.99	1.00	1.00	1.00
26	0.28	0.51	0.72	0.85	0.99	0.99	0.99	1.00	1.00	1.00
27	0.58	0.62	0.79	0.80	0.80	0.77	0.82	0.83	0.88	0.95
28	0.92	0.99	1.00	0.99	1.00	1.00	1.00	1.00	1.00	1.00
29	0.92	0.97	0.98	1.00	1.00	1.00	1.00	1.00	1.00	1.00
30	0.83	0.84	0.85	0.84	0.88	0.93	0.94	0.96	0.98	0.99
31	0.07	0.14	0.34	0.36	0.65	0.80	0.93	0.96	1.00	1.00
32	0.05	0.20	0.30	0.53	0.63	0.82	0.93	0.97	1.00	1.00
33	0.22	0.34	0.37	0.40	0.63	0.71	0.79	0.92	0.96	0.99
34	0.99	1.00	1.00	1.00	1.00	1.00	1.00	1.00	1.00	1.00
35	0.98	1.00	1.00	1.00	1.00	1.00	1.00	1.00	1.00	1.00
36	0.99	0.98	0.98	1.00	0.99	1.00	1.00	1.00	1.00	1.00
37	0.03	0.09	0.29	0.46	0.75	0.85	0.94	0.99	0.97	1.00
38	0.01	0.11	0.23	0.41	0.78	0.89	0.97	0.99	1.00	1.00
39	0.07	0.10	0.36	0.42	0.62	0.83	0.91	1.00	0.99	1.00
40	0.22	0.33	0.60	0.69	0.81	0.92	1.00	0.99	1.00	1.00
41	0.23	0.30	0.58	0.71	0.87	0.93	1.00	1.00	1.00	1.00
42	0.26	0.34	0.47	0.69	0.88	0.91	0.96	1.00	1.00	1.00

Table 5: POD according to each scenario and each k2 value, k1 = 5, alpha = 0.001

	k2=1	k2=2	k2=3	k2=4	k2=5	k2=6	k2=7	k2=8	k2=9	k2=10
1	0.27	0.30	0.39	0.65	0.72	0.82	0.96	0.91	0.96	0.98
2	0.23	0.37	0.44	0.53	0.76	0.81	0.91	0.92	0.96	0.99
3	0.30	0.38	0.44	0.54	0.53	0.67	0.77	0.79	0.89	0.89
4	0.97	0.96	0.97	0.99	1.00	1.00	1.00	1.00	1.00	1.00
5	0.98	0.99	1.00	1.00	1.00	1.00	1.00	1.00	1.00	1.00
6	1.00	1.00	1.00	1.00	1.00	1.00	1.00	1.00	1.00	1.00
7	0.72	0.76	0.83	0.78	0.86	0.89	0.95	0.92	0.97	0.95
8	0.76	0.81	0.82	0.85	0.95	0.89	0.96	0.88	0.95	0.94
9	0.76	0.80	0.85	0.81	0.88	0.89	0.94	0.95	0.98	0.96
10	1.00	1.00	1.00	1.00	1.00	1.00	1.00	1.00	1.00	1.00
11	1.00	1.00	1.00	1.00	1.00	1.00	1.00	1.00	1.00	1.00
12	0.98	1.00	1.00	1.00	1.00	1.00	1.00	1.00	1.00	1.00
13	0.06	0.08	0.22	0.41	0.58	0.78	0.81	0.91	0.97	0.99
14	0.03	0.06	0.21	0.40	0.64	0.69	0.87	0.95	0.97	0.95
15	0.19	0.26	0.43	0.41	0.57	0.60	0.72	0.85	0.86	0.93
16	1.00	1.00	1.00	1.00	1.00	1.00	1.00	1.00	1.00	1.00
17	1.00	1.00	1.00	1.00	1.00	1.00	1.00	1.00	1.00	1.00
18	0.94	0.97	0.99	0.99	1.00	1.00	1.00	1.00	1.00	1.00
19	0.38	0.60	0.75	0.85	0.97	0.97	1.00	1.00	1.00	1.00
20	0.42	0.56	0.77	0.94	0.96	0.99	1.00	1.00	1.00	1.00
21	0.44	0.49	0.64	0.81	0.87	0.92	0.98	0.98	0.99	1.00
22	0.83	0.94	0.99	1.00	1.00	1.00	1.00	1.00	1.00	1.00
23	0.85	0.96	0.97	0.99	1.00	1.00	1.00	1.00	1.00	1.00
24	0.94	0.94	0.97	0.98	0.99	1.00	1.00	1.00	1.00	1.00
25	0.36	0.42	0.69	0.81	0.92	0.96	0.98	1.00	0.99	1.00
26	0.27	0.49	0.65	0.83	0.90	0.97	0.98	0.99	1.00	1.00
27	0.53	0.68	0.59	0.67	0.69	0.71	0.76	0.80	0.88	0.93
28	0.90	0.93	0.98	0.99	1.00	1.00	1.00	1.00	1.00	1.00
29	0.95	0.96	0.99	1.00	1.00	1.00	1.00	1.00	1.00	1.00
30	0.79	0.83	0.87	0.89	0.84	0.88	0.91	0.96	0.97	0.97
31	0.09	0.09	0.28	0.41	0.69	0.84	0.89	0.99	1.00	1.00
32	0.04	0.12	0.28	0.40	0.63	0.83	0.94	0.99	1.00	1.00
33	0.11	0.28	0.25	0.45	0.56	0.68	0.80	0.92	0.96	1.00
34	1.00	1.00	1.00	1.00	1.00	1.00	1.00	1.00	1.00	1.00
35	1.00	1.00	1.00	1.00	1.00	1.00	1.00	1.00	1.00	1.00
36	0.97	0.98	1.00	1.00	1.00	1.00	1.00	1.00	1.00	1.00
37	0.03	0.08	0.22	0.51	0.70	0.87	0.95	0.98	0.98	1.00
38	0.04	0.12	0.34	0.53	0.67	0.81	0.97	0.98	1.00	1.00
39	0.05	0.15	0.26	0.52	0.68	0.82	0.88	0.99	1.00	1.00
40	0.16	0.27	0.46	0.79	0.81	0.90	0.99	1.00	1.00	1.00
41	0.24	0.29	0.40	0.76	0.90	0.96	0.97	0.99	1.00	1.00
42	0.13	0.37	0.44	0.75	0.85	0.94	0.97	1.00	1.00	1.00

Table 6: POD according to each scenario and each k2 value, k1 = 10, alpha = 0.001

Overall performances of CUSUM GLM algorithm ($\alpha = 0.01$)

	FPR k1=0	FPR k1=2	FPR k1=3	FPR k1=5	FPR k1=10
1	0.05	0.05	0.05	0.05	0.04
2	0.05	0.05	0.05	0.05	0.04
3	0.14	0.13	0.13	0.13	0.12
4	0.88	0.88	0.87	0.86	0.84
5	0.95	0.95	0.95	0.94	0.93
6	1.00	0.99	0.99	0.99	0.99
7	0.10	0.09	0.09	0.09	0.08
8	0.11	0.09	0.09	0.09	0.08
9	0.12	0.11	0.10	0.10	0.09
10	0.94	0.94	0.93	0.93	0.92
11	0.95	0.95	0.95	0.95	0.95
12	0.98	0.98	0.98	0.98	0.97
13	0.01	0.01	0.01	0.01	0.01
14	0.01	0.01	0.01	0.01	0.01
15	0.10	0.10	0.10	0.09	0.08
16	1.00	1.00	1.00	1.00	1.00
17	1.00	1.00	1.00	1.00	1.00
18	0.90	0.90	0.90	0.89	0.89
19	0.25	0.24	0.23	0.22	0.20
20	0.26	0.25	0.24	0.23	0.21
21	0.24	0.24	0.23	0.22	0.21
22	0.76	0.74	0.73	0.72	0.68
23	0.80	0.78	0.78	0.76	0.71
24	0.87	0.86	0.85	0.84	0.80
25	0.14	0.14	0.13	0.13	0.12
26	0.13	0.12	0.12	0.12	0.11
27	0.52	0.51	0.51	0.50	0.49
28	0.84	0.83	0.82	0.82	0.79
29	0.84	0.83	0.82	0.81	0.77
30	0.76	0.76	0.76	0.75	0.75
31	0.01	0.01	0.01	0.01	0.01
32	0.01	0.01	0.01	0.01	0.01
33	0.05	0.05	0.05	0.04	0.04
34	0.98	0.98	0.98	0.98	0.98
35	0.99	0.99	0.99	0.99	0.98
36	0.93	0.93	0.92	0.92	0.92
37	0.01	0.01	0.01	0.01	0.01
38	0.01	0.01	0.01	0.01	0.00
39	0.02	0.02	0.02	0.02	0.02
40	0.06	0.05	0.05	0.05	0.04
41	0.06	0.05	0.05	0.05	0.04
42	0.11	0.10	0.10	0.10	0.09

Table 7: FPR according to each scenario and each k1 value, alpha = 0.01

	k2=1	k2=2	k2=3	k2=4	k2=5	k2=6	k2=7	k2=8	k2=9	k2=10
1	0.30	0.36	0.44	0.57	0.73	0.81	0.96	0.95	1.00	0.98
2	0.28	0.36	0.50	0.76	0.75	0.79	0.92	0.97	0.99	1.00
3	0.34	0.42	0.42	0.56	0.59	0.69	0.72	0.89	0.93	0.90
4	0.95	0.98	1.00	1.00	1.00	1.00	1.00	1.00	1.00	1.00
5	0.98	1.00	0.99	1.00	1.00	1.00	1.00	1.00	1.00	1.00
6	1.00	1.00	1.00	1.00	1.00	1.00	1.00	1.00	1.00	1.00
7	0.90	0.90	0.89	0.95	0.98	0.96	0.94	0.97	0.97	1.00
8	0.83	0.88	0.85	0.98	0.91	0.97	0.94	0.98	0.97	0.99
9	0.85	0.86	0.89	0.90	0.89	0.94	0.96	0.98	0.97	0.98
10	0.98	0.98	1.00	1.00	1.00	1.00	1.00	1.00	1.00	1.00
11	0.99	1.00	1.00	1.00	1.00	1.00	1.00	1.00	1.00	1.00
12	0.99	1.00	1.00	1.00	1.00	1.00	1.00	1.00	1.00	1.00
13	0.06	0.12	0.25	0.48	0.64	0.74	0.81	0.93	0.97	0.98
14	0.07	0.17	0.25	0.35	0.56	0.77	0.84	0.91	0.97	1.00
15	0.24	0.34	0.39	0.45	0.57	0.61	0.72	0.86	0.91	0.97
16	1.00	1.00	1.00	1.00	1.00	1.00	1.00	1.00	1.00	1.00
17	1.00	1.00	1.00	1.00	1.00	1.00	1.00	1.00	1.00	1.00
18	0.95	0.96	0.98	1.00	1.00	1.00	1.00	1.00	1.00	1.00
19	0.36	0.60	0.73	0.90	0.96	1.00	1.00	1.00	1.00	1.00
20	0.44	0.64	0.82	0.91	1.00	0.99	1.00	1.00	1.00	1.00
21	0.45	0.50	0.74	0.76	0.91	0.97	0.96	1.00	1.00	1.00
22	0.91	0.98	1.00	1.00	1.00	1.00	1.00	1.00	1.00	1.00
23	0.93	0.95	0.98	1.00	1.00	1.00	1.00	1.00	1.00	1.00
24	0.93	0.96	1.00	1.00	1.00	1.00	1.00	1.00	1.00	1.00
25	0.35	0.49	0.74	0.86	0.96	0.98	1.00	1.00	1.00	1.00
26	0.34	0.47	0.77	0.86	0.96	0.98	1.00	1.00	1.00	1.00
27	0.71	0.68	0.73	0.78	0.78	0.75	0.86	0.89	0.91	0.96
28	0.97	0.96	1.00	1.00	1.00	1.00	1.00	1.00	1.00	1.00
29	0.92	0.98	1.00	0.99	1.00	0.99	1.00	1.00	1.00	1.00
30	0.82	0.83	0.80	0.86	0.87	0.90	0.92	0.93	0.97	0.99
31	0.07	0.11	0.23	0.44	0.64	0.89	0.96	0.99	0.98	1.00
32	0.03	0.08	0.31	0.48	0.63	0.84	0.92	0.97	0.98	1.00
33	0.12	0.23	0.29	0.45	0.63	0.72	0.86	0.94	0.94	0.99
34	1.00	1.00	1.00	1.00	1.00	1.00	1.00	1.00	1.00	1.00
35	1.00	1.00	1.00	1.00	1.00	1.00	1.00	1.00	1.00	1.00
36	0.97	0.99	1.00	1.00	1.00	1.00	1.00	1.00	1.00	1.00
37	0.03	0.18	0.24	0.61	0.69	0.78	0.95	0.99	0.99	1.00
38	0.06	0.10	0.29	0.50	0.70	0.82	0.94	1.00	1.00	1.00
39	0.06	0.17	0.39	0.54	0.66	0.90	0.92	0.96	0.98	1.00
40	0.15	0.45	0.63	0.88	0.88	0.96	0.98	1.00	1.00	1.00
41	0.22	0.40	0.48	0.79	0.87	0.96	0.98	0.99	1.00	1.00
42	0.26	0.39	0.52	0.61	0.87	0.90	0.99	0.99	1.00	1.00

Table 8: POD according to each scenario and each k2 value, k1 = 0, alpha = 0.01

	k2=1	k2=2	k2=3	k2=4	k2=5	k2=6	k2=7	k2=8	k2=9	k2=10
1	0.22	0.40	0.55	0.55	0.80	0.76	0.88	0.97	0.94	0.96
2	0.29	0.41	0.43	0.58	0.82	0.81	0.85	0.93	0.95	0.98
3	0.29	0.39	0.44	0.59	0.57	0.75	0.83	0.80	0.84	0.93
4	0.99	0.99	1.00	1.00	1.00	1.00	1.00	1.00	1.00	1.00
5	0.99	1.00	1.00	1.00	1.00	1.00	1.00	1.00	1.00	1.00
6	1.00	1.00	1.00	1.00	1.00	1.00	1.00	1.00	1.00	1.00
7	0.76	0.78	0.87	0.90	0.91	0.93	0.95	0.96	0.95	0.98
8	0.87	0.90	0.87	0.84	0.93	0.92	0.94	0.95	0.98	0.99
9	0.76	0.84	0.89	0.90	0.88	0.93	0.96	0.99	0.97	0.99
10	1.00	1.00	1.00	1.00	1.00	1.00	1.00	1.00	1.00	1.00
11	0.99	0.99	1.00	0.99	1.00	1.00	1.00	1.00	1.00	1.00
12	1.00	1.00	1.00	1.00	1.00	1.00	1.00	1.00	1.00	1.00
13	0.03	0.11	0.26	0.46	0.69	0.69	0.91	0.92	0.97	0.99
14	0.08	0.13	0.27	0.40	0.49	0.74	0.85	0.91	0.97	0.99
15	0.24	0.26	0.43	0.47	0.60	0.67	0.72	0.85	0.82	0.92
16	1.00	1.00	1.00	1.00	1.00	1.00	1.00	1.00	1.00	1.00
17	1.00	1.00	1.00	1.00	1.00	1.00	1.00	1.00	1.00	1.00
18	0.95	0.98	0.99	1.00	1.00	1.00	1.00	1.00	1.00	1.00
19	0.43	0.62	0.75	0.91	0.99	0.99	1.00	1.00	1.00	1.00
20	0.49	0.64	0.78	0.92	0.98	1.00	1.00	1.00	1.00	1.00
21	0.40	0.47	0.76	0.75	0.91	0.98	0.99	0.99	0.99	1.00
22	0.86	0.97	1.00	1.00	1.00	1.00	1.00	1.00	1.00	1.00
23	0.90	0.92	1.00	1.00	1.00	1.00	1.00	1.00	1.00	1.00
24	0.91	0.97	0.98	1.00	1.00	1.00	1.00	1.00	1.00	1.00
25	0.32	0.52	0.61	0.88	0.97	0.98	1.00	1.00	1.00	1.00
26	0.33	0.55	0.82	0.79	0.96	1.00	1.00	1.00	1.00	1.00
27	0.58	0.61	0.75	0.73	0.82	0.82	0.83	0.85	0.93	0.95
28	0.94	0.97	0.99	1.00	1.00	1.00	1.00	1.00	1.00	1.00
29	0.94	0.98	0.99	0.99	1.00	1.00	1.00	1.00	1.00	1.00
30	0.84	0.83	0.91	0.90	0.85	0.83	0.96	0.94	0.98	0.99
31	0.08	0.13	0.24	0.46	0.68	0.86	0.92	0.99	1.00	1.00
32	0.04	0.14	0.31	0.44	0.69	0.80	0.94	0.97	0.97	0.98
33	0.09	0.22	0.38	0.49	0.61	0.78	0.84	0.87	0.97	0.97
34	1.00	1.00	1.00	1.00	1.00	1.00	1.00	1.00	1.00	1.00
35	1.00	1.00	1.00	1.00	1.00	1.00	1.00	1.00	1.00	1.00
36	1.00	0.99	1.00	1.00	1.00	1.00	1.00	1.00	1.00	1.00
37	0.08	0.15	0.26	0.53	0.78	0.80	0.91	0.98	0.99	1.00
38	0.02	0.10	0.36	0.53	0.70	0.92	0.97	0.98	0.99	1.00
39	0.06	0.15	0.35	0.46	0.66	0.84	0.94	0.98	0.98	1.00
40	0.22	0.47	0.55	0.81	0.92	0.95	0.99	1.00	0.99	1.00
41	0.21	0.24	0.60	0.75	0.84	0.97	0.99	0.98	1.00	1.00
42	0.26	0.32	0.60	0.65	0.83	0.92	0.98	1.00	1.00	1.00

Table 9: POD according to each scenario and each k2 value, k1 = 2, alpha = 0.01

	k2=1	k2=2	k2=3	k2=4	k2=5	k2=6	k2=7	k2=8	k2=9	k2=10
1	0.29	0.28	0.46	0.67	0.83	0.85	0.91	0.98	0.95	0.96
2	0.28	0.47	0.51	0.57	0.77	0.82	0.97	0.90	0.99	0.97
3	0.32	0.37	0.47	0.54	0.70	0.69	0.75	0.88	0.92	0.91
4	0.98	0.98	0.98	1.00	1.00	1.00	1.00	1.00	1.00	1.00
5	0.99	1.00	0.99	1.00	1.00	1.00	1.00	1.00	1.00	1.00
6	1.00	1.00	1.00	1.00	1.00	1.00	1.00	1.00	1.00	1.00
7	0.73	0.79	0.82	0.92	0.92	0.91	0.98	0.98	0.94	0.99
8	0.80	0.87	0.92	0.88	0.86	0.96	0.98	0.97	0.99	0.96
9	0.85	0.87	0.83	0.91	0.90	0.94	0.95	0.95	0.96	0.98
10	0.99	1.00	1.00	1.00	1.00	1.00	1.00	1.00	1.00	1.00
11	1.00	0.99	1.00	1.00	1.00	1.00	1.00	1.00	1.00	1.00
12	1.00	1.00	1.00	1.00	1.00	1.00	1.00	1.00	1.00	1.00
13	0.03	0.17	0.28	0.42	0.65	0.74	0.86	0.93	0.99	0.99
14	0.09	0.11	0.29	0.41	0.63	0.75	0.85	0.93	0.93	0.97
15	0.25	0.28	0.42	0.47	0.51	0.67	0.76	0.80	0.93	0.86
16	1.00	1.00	1.00	1.00	1.00	1.00	1.00	1.00	1.00	1.00
17	1.00	1.00	1.00	1.00	1.00	1.00	1.00	1.00	1.00	1.00
18	0.95	0.96	0.99	1.00	1.00	1.00	1.00	1.00	1.00	1.00
19	0.42	0.61	0.78	0.91	0.97	0.98	1.00	0.99	1.00	1.00
20	0.40	0.71	0.82	0.91	0.98	0.98	1.00	1.00	1.00	1.00
21	0.39	0.52	0.71	0.75	0.89	0.95	0.94	0.98	1.00	1.00
22	0.88	0.97	0.99	1.00	1.00	1.00	1.00	1.00	1.00	1.00
23	0.86	0.98	0.99	1.00	1.00	1.00	1.00	1.00	1.00	1.00
24	0.91	0.98	0.97	0.99	0.99	1.00	1.00	1.00	1.00	1.00
25	0.27	0.52	0.69	0.88	0.98	0.98	1.00	1.00	1.00	1.00
26	0.23	0.45	0.71	0.86	0.98	0.99	0.98	1.00	1.00	1.00
27	0.64	0.60	0.67	0.71	0.76	0.75	0.78	0.88	0.88	0.92
28	0.95	0.98	0.97	1.00	1.00	1.00	1.00	1.00	1.00	1.00
29	0.95	0.95	1.00	1.00	0.99	1.00	1.00	1.00	1.00	1.00
30	0.83	0.82	0.86	0.88	0.89	0.90	0.97	0.95	0.95	0.99
31	0.05	0.15	0.25	0.40	0.62	0.95	0.91	0.98	0.99	1.00
32	0.06	0.09	0.26	0.42	0.67	0.79	0.95	0.98	0.99	1.00
33	0.18	0.20	0.34	0.48	0.58	0.83	0.79	0.93	0.98	1.00
34	0.98	1.00	1.00	1.00	1.00	1.00	1.00	1.00	1.00	1.00
35	1.00	1.00	1.00	1.00	1.00	1.00	1.00	1.00	1.00	1.00
36	0.96	0.98	1.00	1.00	1.00	1.00	1.00	1.00	1.00	1.00
37	0.07	0.15	0.39	0.52	0.72	0.89	0.93	0.99	0.98	1.00
38	0.04	0.10	0.27	0.57	0.69	0.89	0.92	0.96	0.99	1.00
39	0.09	0.22	0.33	0.53	0.64	0.76	0.93	0.95	1.00	1.00
40	0.20	0.46	0.55	0.78	0.88	0.99	0.99	0.99	1.00	1.00
41	0.20	0.28	0.43	0.71	0.88	0.98	0.98	1.00	1.00	1.00
42	0.23	0.47	0.55	0.67	0.90	0.93	0.99	1.00	1.00	1.00

Table 10: POD according to each scenario and each k2 value, k1 = 3, alpha = 0.01

	k2=1	k2=2	k2=3	k2=4	k2=5	k2=6	k2=7	k2=8	k2=9	k2=10
1	0.29	0.42	0.50	0.61	0.73	0.76	0.90	0.99	0.99	0.97
2	0.19	0.37	0.54	0.63	0.70	0.86	0.88	0.96	0.95	0.98
3	0.42	0.37	0.46	0.60	0.56	0.65	0.75	0.81	0.86	0.93
4	0.96	0.94	0.99	1.00	1.00	1.00	1.00	1.00	1.00	1.00
5	0.99	0.98	1.00	1.00	1.00	1.00	1.00	1.00	1.00	1.00
6	0.98	1.00	1.00	1.00	1.00	1.00	1.00	1.00	1.00	1.00
7	0.80	0.88	0.79	0.89	0.89	0.86	0.93	0.95	0.98	1.00
8	0.84	0.82	0.87	0.90	0.93	0.89	0.95	0.99	0.95	0.98
9	0.77	0.79	0.89	0.82	0.89	0.91	0.96	0.96	0.96	0.97
10	0.99	0.99	0.99	1.00	1.00	1.00	1.00	1.00	1.00	1.00
11	0.99	1.00	1.00	1.00	1.00	1.00	1.00	1.00	1.00	1.00
12	1.00	1.00	1.00	1.00	1.00	1.00	1.00	1.00	1.00	1.00
13	0.04	0.18	0.31	0.43	0.68	0.79	0.82	0.92	0.97	0.98
14	0.02	0.11	0.30	0.49	0.63	0.76	0.85	0.93	0.98	0.96
15	0.25	0.31	0.37	0.55	0.56	0.59	0.72	0.86	0.87	0.93
16	1.00	1.00	1.00	1.00	1.00	1.00	1.00	1.00	1.00	1.00
17	1.00	1.00	1.00	1.00	1.00	1.00	1.00	1.00	1.00	1.00
18	0.94	0.96	0.97	1.00	1.00	1.00	1.00	1.00	1.00	1.00
19	0.47	0.67	0.82	0.92	0.98	0.98	0.99	0.99	1.00	1.00
20	0.45	0.56	0.79	0.94	0.98	0.99	0.99	1.00	1.00	1.00
21	0.46	0.56	0.63	0.79	0.92	0.93	0.97	0.99	1.00	1.00
22	0.87	0.98	1.00	1.00	1.00	1.00	1.00	1.00	1.00	1.00
23	0.88	0.98	1.00	1.00	1.00	0.99	1.00	1.00	1.00	1.00
24	0.93	0.94	0.97	1.00	1.00	1.00	1.00	1.00	1.00	1.00
25	0.34	0.56	0.66	0.85	0.96	0.99	0.99	1.00	1.00	1.00
26	0.28	0.51	0.72	0.85	0.99	0.99	0.99	1.00	1.00	1.00
27	0.58	0.62	0.79	0.80	0.80	0.77	0.82	0.83	0.88	0.95
28	0.92	0.99	1.00	0.99	1.00	1.00	1.00	1.00	1.00	1.00
29	0.92	0.97	0.98	1.00	1.00	1.00	1.00	1.00	1.00	1.00
30	0.83	0.84	0.85	0.84	0.88	0.93	0.94	0.96	0.98	0.99
31	0.07	0.14	0.34	0.36	0.65	0.80	0.93	0.96	1.00	1.00
32	0.05	0.20	0.30	0.53	0.63	0.82	0.93	0.97	1.00	1.00
33	0.22	0.34	0.37	0.40	0.63	0.71	0.79	0.92	0.96	0.99
34	0.99	1.00	1.00	1.00	1.00	1.00	1.00	1.00	1.00	1.00
35	0.98	1.00	1.00	1.00	1.00	1.00	1.00	1.00	1.00	1.00
36	0.99	0.98	0.98	1.00	0.99	1.00	1.00	1.00	1.00	1.00
37	0.03	0.09	0.29	0.46	0.75	0.85	0.94	0.99	0.97	1.00
38	0.01	0.11	0.23	0.41	0.78	0.89	0.97	0.99	1.00	1.00
39	0.07	0.10	0.36	0.42	0.62	0.83	0.91	1.00	0.99	1.00
40	0.22	0.33	0.60	0.69	0.81	0.92	1.00	0.99	1.00	1.00
41	0.23	0.30	0.58	0.71	0.87	0.93	1.00	1.00	1.00	1.00
42	0.26	0.34	0.47	0.69	0.88	0.91	0.96	1.00	1.00	1.00

Table 11: POD according to each scenario and each k2 value, k1 = 5, alpha = 0.01

	k2=1	k2=2	k2=3	k2=4	k2=5	k2=6	k2=7	k2=8	k2=9	k2=10
1	0.27	0.30	0.39	0.65	0.72	0.82	0.96	0.91	0.96	0.98
2	0.23	0.37	0.44	0.53	0.76	0.81	0.91	0.92	0.96	0.99
3	0.30	0.38	0.44	0.54	0.53	0.67	0.77	0.79	0.89	0.89
4	0.97	0.96	0.97	0.99	1.00	1.00	1.00	1.00	1.00	1.00
5	0.98	0.99	1.00	1.00	1.00	1.00	1.00	1.00	1.00	1.00
6	1.00	1.00	1.00	1.00	1.00	1.00	1.00	1.00	1.00	1.00
7	0.72	0.76	0.83	0.78	0.86	0.89	0.95	0.92	0.97	0.95
8	0.76	0.81	0.82	0.85	0.95	0.89	0.96	0.88	0.95	0.94
9	0.76	0.80	0.85	0.81	0.88	0.89	0.94	0.95	0.98	0.96
10	1.00	1.00	1.00	1.00	1.00	1.00	1.00	1.00	1.00	1.00
11	1.00	1.00	1.00	1.00	1.00	1.00	1.00	1.00	1.00	1.00
12	0.98	1.00	1.00	1.00	1.00	1.00	1.00	1.00	1.00	1.00
13	0.06	0.08	0.22	0.41	0.58	0.78	0.81	0.91	0.97	0.99
14	0.03	0.06	0.21	0.40	0.64	0.69	0.87	0.95	0.97	0.95
15	0.19	0.26	0.43	0.41	0.57	0.60	0.72	0.85	0.86	0.93
16	1.00	1.00	1.00	1.00	1.00	1.00	1.00	1.00	1.00	1.00
17	1.00	1.00	1.00	1.00	1.00	1.00	1.00	1.00	1.00	1.00
18	0.94	0.97	0.99	0.99	1.00	1.00	1.00	1.00	1.00	1.00
19	0.38	0.60	0.75	0.85	0.97	0.97	1.00	1.00	1.00	1.00
20	0.42	0.56	0.77	0.94	0.96	0.99	1.00	1.00	1.00	1.00
21	0.44	0.49	0.64	0.81	0.87	0.92	0.98	0.98	0.99	1.00
22	0.83	0.94	0.99	1.00	1.00	1.00	1.00	1.00	1.00	1.00
23	0.85	0.96	0.97	0.99	1.00	1.00	1.00	1.00	1.00	1.00
24	0.94	0.94	0.97	0.98	0.99	1.00	1.00	1.00	1.00	1.00
25	0.36	0.42	0.69	0.81	0.92	0.96	0.98	1.00	0.99	1.00
26	0.27	0.49	0.65	0.83	0.90	0.97	0.98	0.99	1.00	1.00
27	0.53	0.68	0.59	0.67	0.69	0.71	0.76	0.80	0.88	0.93
28	0.90	0.93	0.98	0.99	1.00	1.00	1.00	1.00	1.00	1.00
29	0.95	0.96	0.99	1.00	1.00	1.00	1.00	1.00	1.00	1.00
30	0.79	0.83	0.87	0.89	0.84	0.88	0.91	0.96	0.97	0.97
31	0.09	0.09	0.28	0.41	0.69	0.84	0.89	0.99	1.00	1.00
32	0.04	0.12	0.28	0.40	0.63	0.83	0.94	0.99	1.00	1.00
33	0.11	0.28	0.25	0.45	0.56	0.68	0.80	0.92	0.96	1.00
34	1.00	1.00	1.00	1.00	1.00	1.00	1.00	1.00	1.00	1.00
35	1.00	1.00	1.00	1.00	1.00	1.00	1.00	1.00	1.00	1.00
36	0.97	0.98	1.00	1.00	1.00	1.00	1.00	1.00	1.00	1.00
37	0.03	0.08	0.22	0.51	0.70	0.87	0.95	0.98	0.98	1.00
38	0.04	0.12	0.34	0.53	0.67	0.81	0.97	0.98	1.00	1.00
39	0.05	0.15	0.26	0.52	0.68	0.82	0.88	0.99	1.00	1.00
40	0.16	0.27	0.46	0.79	0.81	0.90	0.99	1.00	1.00	1.00
41	0.24	0.29	0.40	0.76	0.90	0.96	0.97	0.99	1.00	1.00
42	0.13	0.37	0.44	0.75	0.85	0.94	0.97	1.00	1.00	1.00

Table 12: POD according to each scenario and each k2 value, k1 = 10, alpha = 0.01

Overall performances of CUSUM GLM algorithm ($\alpha = 0.05$)

	FPR k1=0	FPR k1=2	FPR k1=3	FPR k1=5	FPR k1=10
1	0.05	0.05	0.05	0.05	0.04
2	0.05	0.05	0.05	0.05	0.04
3	0.14	0.13	0.13	0.13	0.12
4	0.88	0.88	0.87	0.86	0.84
5	0.95	0.95	0.95	0.94	0.93
6	1.00	0.99	0.99	0.99	0.99
7	0.10	0.09	0.09	0.09	0.08
8	0.11	0.09	0.09	0.09	0.08
9	0.12	0.11	0.10	0.10	0.09
10	0.94	0.94	0.93	0.93	0.92
11	0.95	0.95	0.95	0.95	0.95
12	0.98	0.98	0.98	0.98	0.97
13	0.01	0.01	0.01	0.01	0.01
14	0.01	0.01	0.01	0.01	0.01
15	0.10	0.10	0.10	0.09	0.08
16	1.00	1.00	1.00	1.00	1.00
17	1.00	1.00	1.00	1.00	1.00
18	0.90	0.90	0.90	0.89	0.89
19	0.25	0.24	0.23	0.22	0.20
20	0.26	0.25	0.24	0.23	0.21
21	0.24	0.24	0.23	0.22	0.21
22	0.76	0.74	0.73	0.72	0.68
23	0.80	0.78	0.78	0.76	0.71
24	0.87	0.86	0.85	0.84	0.80
25	0.14	0.14	0.13	0.13	0.12
26	0.13	0.12	0.12	0.12	0.11
27	0.52	0.51	0.51	0.50	0.49
28	0.84	0.83	0.82	0.82	0.79
29	0.84	0.83	0.82	0.81	0.77
30	0.76	0.76	0.76	0.75	0.75
31	0.01	0.01	0.01	0.01	0.01
32	0.01	0.01	0.01	0.01	0.01
33	0.05	0.05	0.05	0.04	0.04
34	0.98	0.98	0.98	0.98	0.98
35	0.99	0.99	0.99	0.99	0.98
36	0.93	0.93	0.92	0.92	0.92
37	0.01	0.01	0.01	0.01	0.01
38	0.01	0.01	0.01	0.01	0.00
39	0.02	0.02	0.02	0.02	0.02
40	0.06	0.05	0.05	0.05	0.04
41	0.06	0.05	0.05	0.05	0.04
42	0.11	0.10	0.10	0.10	0.09

Table 13: FPR according to each scenario and each k1 value, alpha = 0.05

	k2=1	k2=2	k2=3	k2=4	k2=5	k2=6	k2=7	k2=8	k2=9	k2=10
1	0.30	0.36	0.44	0.57	0.73	0.81	0.96	0.95	1.00	0.98
2	0.28	0.36	0.50	0.76	0.75	0.79	0.92	0.97	0.99	1.00
3	0.34	0.42	0.42	0.56	0.59	0.69	0.72	0.89	0.93	0.90
4	0.95	0.98	1.00	1.00	1.00	1.00	1.00	1.00	1.00	1.00
5	0.98	1.00	0.99	1.00	1.00	1.00	1.00	1.00	1.00	1.00
6	1.00	1.00	1.00	1.00	1.00	1.00	1.00	1.00	1.00	1.00
7	0.90	0.90	0.89	0.95	0.98	0.96	0.94	0.97	0.97	1.00
8	0.83	0.88	0.85	0.98	0.91	0.97	0.94	0.98	0.97	0.99
9	0.85	0.86	0.89	0.90	0.89	0.94	0.96	0.98	0.97	0.98
10	0.98	0.98	1.00	1.00	1.00	1.00	1.00	1.00	1.00	1.00
11	0.99	1.00	1.00	1.00	1.00	1.00	1.00	1.00	1.00	1.00
12	0.99	1.00	1.00	1.00	1.00	1.00	1.00	1.00	1.00	1.00
13	0.06	0.12	0.25	0.48	0.64	0.74	0.81	0.93	0.97	0.98
14	0.07	0.17	0.25	0.35	0.56	0.77	0.84	0.91	0.97	1.00
15	0.24	0.34	0.39	0.45	0.57	0.61	0.72	0.86	0.91	0.97
16	1.00	1.00	1.00	1.00	1.00	1.00	1.00	1.00	1.00	1.00
17	1.00	1.00	1.00	1.00	1.00	1.00	1.00	1.00	1.00	1.00
18	0.95	0.96	0.98	1.00	1.00	1.00	1.00	1.00	1.00	1.00
19	0.36	0.60	0.73	0.90	0.96	1.00	1.00	1.00	1.00	1.00
20	0.44	0.64	0.82	0.91	1.00	0.99	1.00	1.00	1.00	1.00
21	0.45	0.50	0.74	0.76	0.91	0.97	0.96	1.00	1.00	1.00
22	0.91	0.98	1.00	1.00	1.00	1.00	1.00	1.00	1.00	1.00
23	0.93	0.95	0.98	1.00	1.00	1.00	1.00	1.00	1.00	1.00
24	0.93	0.96	1.00	1.00	1.00	1.00	1.00	1.00	1.00	1.00
25	0.35	0.49	0.74	0.86	0.96	0.98	1.00	1.00	1.00	1.00
26	0.34	0.47	0.77	0.86	0.96	0.98	1.00	1.00	1.00	1.00
27	0.71	0.68	0.73	0.78	0.78	0.75	0.86	0.89	0.91	0.96
28	0.97	0.96	1.00	1.00	1.00	1.00	1.00	1.00	1.00	1.00
29	0.92	0.98	1.00	0.99	1.00	0.99	1.00	1.00	1.00	1.00
30	0.82	0.83	0.80	0.86	0.87	0.90	0.92	0.93	0.97	0.99
31	0.07	0.11	0.23	0.44	0.64	0.89	0.96	0.99	0.98	1.00
32	0.03	0.08	0.31	0.48	0.63	0.84	0.92	0.97	0.98	1.00
33	0.12	0.23	0.29	0.45	0.63	0.72	0.86	0.94	0.94	0.99
34	1.00	1.00	1.00	1.00	1.00	1.00	1.00	1.00	1.00	1.00
35	1.00	1.00	1.00	1.00	1.00	1.00	1.00	1.00	1.00	1.00
36	0.97	0.99	1.00	1.00	1.00	1.00	1.00	1.00	1.00	1.00
37	0.03	0.18	0.24	0.61	0.69	0.78	0.95	0.99	0.99	1.00
38	0.06	0.10	0.29	0.50	0.70	0.82	0.94	1.00	1.00	1.00
39	0.06	0.17	0.39	0.54	0.66	0.90	0.92	0.96	0.98	1.00
40	0.15	0.45	0.63	0.88	0.88	0.96	0.98	1.00	1.00	1.00
41	0.22	0.40	0.48	0.79	0.87	0.96	0.98	0.99	1.00	1.00
42	0.26	0.39	0.52	0.61	0.87	0.90	0.99	0.99	1.00	1.00

Table 14: POD according to each scenario and each k2 value, k1 = 0, alpha = 0.05

	k2=1	k2=2	k2=3	k2=4	k2=5	k2=6	k2=7	k2=8	k2=9	k2=10
1	0.22	0.40	0.55	0.55	0.80	0.76	0.88	0.97	0.94	0.96
2	0.29	0.41	0.43	0.58	0.82	0.81	0.85	0.93	0.95	0.98
3	0.29	0.39	0.44	0.59	0.57	0.75	0.83	0.80	0.84	0.93
4	0.99	0.99	1.00	1.00	1.00	1.00	1.00	1.00	1.00	1.00
5	0.99	1.00	1.00	1.00	1.00	1.00	1.00	1.00	1.00	1.00
6	1.00	1.00	1.00	1.00	1.00	1.00	1.00	1.00	1.00	1.00
7	0.76	0.78	0.87	0.90	0.91	0.93	0.95	0.96	0.95	0.98
8	0.87	0.90	0.87	0.84	0.93	0.92	0.94	0.95	0.98	0.99
9	0.76	0.84	0.89	0.90	0.88	0.93	0.96	0.99	0.97	0.99
10	1.00	1.00	1.00	1.00	1.00	1.00	1.00	1.00	1.00	1.00
11	0.99	0.99	1.00	0.99	1.00	1.00	1.00	1.00	1.00	1.00
12	1.00	1.00	1.00	1.00	1.00	1.00	1.00	1.00	1.00	1.00
13	0.03	0.11	0.26	0.46	0.69	0.69	0.91	0.92	0.97	0.99
14	0.08	0.13	0.27	0.40	0.49	0.74	0.85	0.91	0.97	0.99
15	0.24	0.26	0.43	0.47	0.60	0.67	0.72	0.85	0.82	0.92
16	1.00	1.00	1.00	1.00	1.00	1.00	1.00	1.00	1.00	1.00
17	1.00	1.00	1.00	1.00	1.00	1.00	1.00	1.00	1.00	1.00
18	0.95	0.98	0.99	1.00	1.00	1.00	1.00	1.00	1.00	1.00
19	0.43	0.62	0.75	0.91	0.99	0.99	1.00	1.00	1.00	1.00
20	0.49	0.64	0.78	0.92	0.98	1.00	1.00	1.00	1.00	1.00
21	0.40	0.47	0.76	0.75	0.91	0.98	0.99	0.99	0.99	1.00
22	0.86	0.97	1.00	1.00	1.00	1.00	1.00	1.00	1.00	1.00
23	0.90	0.92	1.00	1.00	1.00	1.00	1.00	1.00	1.00	1.00
24	0.91	0.97	0.98	1.00	1.00	1.00	1.00	1.00	1.00	1.00
25	0.32	0.52	0.61	0.88	0.97	0.98	1.00	1.00	1.00	1.00
26	0.33	0.55	0.82	0.79	0.96	1.00	1.00	1.00	1.00	1.00
27	0.58	0.61	0.75	0.73	0.82	0.82	0.83	0.85	0.93	0.95
28	0.94	0.97	0.99	1.00	1.00	1.00	1.00	1.00	1.00	1.00
29	0.94	0.98	0.99	0.99	1.00	1.00	1.00	1.00	1.00	1.00
30	0.84	0.83	0.91	0.90	0.85	0.83	0.96	0.94	0.98	0.99
31	0.08	0.13	0.24	0.46	0.68	0.86	0.92	0.99	1.00	1.00
32	0.04	0.14	0.31	0.44	0.69	0.80	0.94	0.97	0.97	0.98
33	0.09	0.22	0.38	0.49	0.61	0.78	0.84	0.87	0.97	0.97
34	1.00	1.00	1.00	1.00	1.00	1.00	1.00	1.00	1.00	1.00
35	1.00	1.00	1.00	1.00	1.00	1.00	1.00	1.00	1.00	1.00
36	1.00	0.99	1.00	1.00	1.00	1.00	1.00	1.00	1.00	1.00
37	0.08	0.15	0.26	0.53	0.78	0.80	0.91	0.98	0.99	1.00
38	0.02	0.10	0.36	0.53	0.70	0.92	0.97	0.98	0.99	1.00
39	0.06	0.15	0.35	0.46	0.66	0.84	0.94	0.98	0.98	1.00
40	0.22	0.47	0.55	0.81	0.92	0.95	0.99	1.00	0.99	1.00
41	0.21	0.24	0.60	0.75	0.84	0.97	0.99	0.98	1.00	1.00
42	0.26	0.32	0.60	0.65	0.83	0.92	0.98	1.00	1.00	1.00

Table 15: POD according to each scenario and each k2 value, k1 = 2, alpha = 0.05

	k2=1	k2=2	k2=3	k2=4	k2=5	k2=6	k2=7	k2=8	k2=9	k2=10
1	0.29	0.28	0.46	0.67	0.83	0.85	0.91	0.98	0.95	0.96
2	0.28	0.47	0.51	0.57	0.77	0.82	0.97	0.90	0.99	0.97
3	0.32	0.37	0.47	0.54	0.70	0.69	0.75	0.88	0.92	0.91
4	0.98	0.98	0.98	1.00	1.00	1.00	1.00	1.00	1.00	1.00
5	0.99	1.00	0.99	1.00	1.00	1.00	1.00	1.00	1.00	1.00
6	1.00	1.00	1.00	1.00	1.00	1.00	1.00	1.00	1.00	1.00
7	0.73	0.79	0.82	0.92	0.92	0.91	0.98	0.98	0.94	0.99
8	0.80	0.87	0.92	0.88	0.86	0.96	0.98	0.97	0.99	0.96
9	0.85	0.87	0.83	0.91	0.90	0.94	0.95	0.95	0.96	0.98
10	0.99	1.00	1.00	1.00	1.00	1.00	1.00	1.00	1.00	1.00
11	1.00	0.99	1.00	1.00	1.00	1.00	1.00	1.00	1.00	1.00
12	1.00	1.00	1.00	1.00	1.00	1.00	1.00	1.00	1.00	1.00
13	0.03	0.17	0.28	0.42	0.65	0.74	0.86	0.93	0.99	0.99
14	0.09	0.11	0.29	0.41	0.63	0.75	0.85	0.93	0.93	0.97
15	0.25	0.28	0.42	0.47	0.51	0.67	0.76	0.80	0.93	0.86
16	1.00	1.00	1.00	1.00	1.00	1.00	1.00	1.00	1.00	1.00
17	1.00	1.00	1.00	1.00	1.00	1.00	1.00	1.00	1.00	1.00
18	0.95	0.96	0.99	1.00	1.00	1.00	1.00	1.00	1.00	1.00
19	0.42	0.61	0.78	0.91	0.97	0.98	1.00	0.99	1.00	1.00
20	0.40	0.71	0.82	0.91	0.98	0.98	1.00	1.00	1.00	1.00
21	0.39	0.52	0.71	0.75	0.89	0.95	0.94	0.98	1.00	1.00
22	0.88	0.97	0.99	1.00	1.00	1.00	1.00	1.00	1.00	1.00
23	0.86	0.98	0.99	1.00	1.00	1.00	1.00	1.00	1.00	1.00
24	0.91	0.98	0.97	0.99	0.99	1.00	1.00	1.00	1.00	1.00
25	0.27	0.52	0.69	0.88	0.98	0.98	1.00	1.00	1.00	1.00
26	0.23	0.45	0.71	0.86	0.98	0.99	0.98	1.00	1.00	1.00
27	0.64	0.60	0.67	0.71	0.76	0.75	0.78	0.88	0.88	0.92
28	0.95	0.98	0.97	1.00	1.00	1.00	1.00	1.00	1.00	1.00
29	0.95	0.95	1.00	1.00	0.99	1.00	1.00	1.00	1.00	1.00
30	0.83	0.82	0.86	0.88	0.89	0.90	0.97	0.95	0.95	0.99
31	0.05	0.15	0.25	0.40	0.62	0.95	0.91	0.98	0.99	1.00
32	0.06	0.09	0.26	0.42	0.67	0.79	0.95	0.98	0.99	1.00
33	0.18	0.20	0.34	0.48	0.58	0.83	0.79	0.93	0.98	1.00
34	0.98	1.00	1.00	1.00	1.00	1.00	1.00	1.00	1.00	1.00
35	1.00	1.00	1.00	1.00	1.00	1.00	1.00	1.00	1.00	1.00
36	0.96	0.98	1.00	1.00	1.00	1.00	1.00	1.00	1.00	1.00
37	0.07	0.15	0.39	0.52	0.72	0.89	0.93	0.99	0.98	1.00
38	0.04	0.10	0.27	0.57	0.69	0.89	0.92	0.96	0.99	1.00
39	0.09	0.22	0.33	0.53	0.64	0.76	0.93	0.95	1.00	1.00
40	0.20	0.46	0.55	0.78	0.88	0.99	0.99	0.99	1.00	1.00
41	0.20	0.28	0.43	0.71	0.88	0.98	0.98	1.00	1.00	1.00
42	0.23	0.47	0.55	0.67	0.90	0.93	0.99	1.00	1.00	1.00

Table 16: POD according to each scenario and each k2 value, k1 = 3, alpha = 0.05

	k2=1	k2=2	k2=3	k2=4	k2=5	k2=6	k2=7	k2=8	k2=9	k2=10
1	0.29	0.42	0.50	0.61	0.73	0.76	0.90	0.99	0.99	0.97
2	0.19	0.37	0.54	0.63	0.70	0.86	0.88	0.96	0.95	0.98
3	0.42	0.37	0.46	0.60	0.56	0.65	0.75	0.81	0.86	0.93
4	0.96	0.94	0.99	1.00	1.00	1.00	1.00	1.00	1.00	1.00
5	0.99	0.98	1.00	1.00	1.00	1.00	1.00	1.00	1.00	1.00
6	0.98	1.00	1.00	1.00	1.00	1.00	1.00	1.00	1.00	1.00
7	0.80	0.88	0.79	0.89	0.89	0.86	0.93	0.95	0.98	1.00
8	0.84	0.82	0.87	0.90	0.93	0.89	0.95	0.99	0.95	0.98
9	0.77	0.79	0.89	0.82	0.89	0.91	0.96	0.96	0.96	0.97
10	0.99	0.99	0.99	1.00	1.00	1.00	1.00	1.00	1.00	1.00
11	0.99	1.00	1.00	1.00	1.00	1.00	1.00	1.00	1.00	1.00
12	1.00	1.00	1.00	1.00	1.00	1.00	1.00	1.00	1.00	1.00
13	0.04	0.18	0.31	0.43	0.68	0.79	0.82	0.92	0.97	0.98
14	0.02	0.11	0.30	0.49	0.63	0.76	0.85	0.93	0.98	0.96
15	0.25	0.31	0.37	0.55	0.56	0.59	0.72	0.86	0.87	0.93
16	1.00	1.00	1.00	1.00	1.00	1.00	1.00	1.00	1.00	1.00
17	1.00	1.00	1.00	1.00	1.00	1.00	1.00	1.00	1.00	1.00
18	0.94	0.96	0.97	1.00	1.00	1.00	1.00	1.00	1.00	1.00
19	0.47	0.67	0.82	0.92	0.98	0.98	0.99	0.99	1.00	1.00
20	0.45	0.56	0.79	0.94	0.98	0.99	0.99	1.00	1.00	1.00
21	0.46	0.56	0.63	0.79	0.92	0.93	0.97	0.99	1.00	1.00
22	0.87	0.98	1.00	1.00	1.00	1.00	1.00	1.00	1.00	1.00
23	0.88	0.98	1.00	1.00	1.00	0.99	1.00	1.00	1.00	1.00
24	0.93	0.94	0.97	1.00	1.00	1.00	1.00	1.00	1.00	1.00
25	0.34	0.56	0.66	0.85	0.96	0.99	0.99	1.00	1.00	1.00
26	0.28	0.51	0.72	0.85	0.99	0.99	0.99	1.00	1.00	1.00
27	0.58	0.62	0.79	0.80	0.80	0.77	0.82	0.83	0.88	0.95
28	0.92	0.99	1.00	0.99	1.00	1.00	1.00	1.00	1.00	1.00
29	0.92	0.97	0.98	1.00	1.00	1.00	1.00	1.00	1.00	1.00
30	0.83	0.84	0.85	0.84	0.88	0.93	0.94	0.96	0.98	0.99
31	0.07	0.14	0.34	0.36	0.65	0.80	0.93	0.96	1.00	1.00
32	0.05	0.20	0.30	0.53	0.63	0.82	0.93	0.97	1.00	1.00
33	0.22	0.34	0.37	0.40	0.63	0.71	0.79	0.92	0.96	0.99
34	0.99	1.00	1.00	1.00	1.00	1.00	1.00	1.00	1.00	1.00
35	0.98	1.00	1.00	1.00	1.00	1.00	1.00	1.00	1.00	1.00
36	0.99	0.98	0.98	1.00	0.99	1.00	1.00	1.00	1.00	1.00
37	0.03	0.09	0.29	0.46	0.75	0.85	0.94	0.99	0.97	1.00
38	0.01	0.11	0.23	0.41	0.78	0.89	0.97	0.99	1.00	1.00
39	0.07	0.10	0.36	0.42	0.62	0.83	0.91	1.00	0.99	1.00
40	0.22	0.33	0.60	0.69	0.81	0.92	1.00	0.99	1.00	1.00
41	0.23	0.30	0.58	0.71	0.87	0.93	1.00	1.00	1.00	1.00
42	0.26	0.34	0.47	0.69	0.88	0.91	0.96	1.00	1.00	1.00

Table 17: POD according to each scenario and each k2 value, k1 = 5, alpha = 0.05

	k2=1	k2=2	k2=3	k2=4	k2=5	k2=6	k2=7	k2=8	k2=9	k2=10
1	0.27	0.30	0.39	0.65	0.72	0.82	0.96	0.91	0.96	0.98
2	0.23	0.37	0.44	0.53	0.76	0.81	0.91	0.92	0.96	0.99
3	0.30	0.38	0.44	0.54	0.53	0.67	0.77	0.79	0.89	0.89
4	0.97	0.96	0.97	0.99	1.00	1.00	1.00	1.00	1.00	1.00
5	0.98	0.99	1.00	1.00	1.00	1.00	1.00	1.00	1.00	1.00
6	1.00	1.00	1.00	1.00	1.00	1.00	1.00	1.00	1.00	1.00
7	0.72	0.76	0.83	0.78	0.86	0.89	0.95	0.92	0.97	0.95
8	0.76	0.81	0.82	0.85	0.95	0.89	0.96	0.88	0.95	0.94
9	0.76	0.80	0.85	0.81	0.88	0.89	0.94	0.95	0.98	0.96
10	1.00	1.00	1.00	1.00	1.00	1.00	1.00	1.00	1.00	1.00
11	1.00	1.00	1.00	1.00	1.00	1.00	1.00	1.00	1.00	1.00
12	0.98	1.00	1.00	1.00	1.00	1.00	1.00	1.00	1.00	1.00
13	0.06	0.08	0.22	0.41	0.58	0.78	0.81	0.91	0.97	0.99
14	0.03	0.06	0.21	0.40	0.64	0.69	0.87	0.95	0.97	0.95
15	0.19	0.26	0.43	0.41	0.57	0.60	0.72	0.85	0.86	0.93
16	1.00	1.00	1.00	1.00	1.00	1.00	1.00	1.00	1.00	1.00
17	1.00	1.00	1.00	1.00	1.00	1.00	1.00	1.00	1.00	1.00
18	0.94	0.97	0.99	0.99	1.00	1.00	1.00	1.00	1.00	1.00
19	0.38	0.60	0.75	0.85	0.97	0.97	1.00	1.00	1.00	1.00
20	0.42	0.56	0.77	0.94	0.96	0.99	1.00	1.00	1.00	1.00
21	0.44	0.49	0.64	0.81	0.87	0.92	0.98	0.98	0.99	1.00
22	0.83	0.94	0.99	1.00	1.00	1.00	1.00	1.00	1.00	1.00
23	0.85	0.96	0.97	0.99	1.00	1.00	1.00	1.00	1.00	1.00
24	0.94	0.94	0.97	0.98	0.99	1.00	1.00	1.00	1.00	1.00
25	0.36	0.42	0.69	0.81	0.92	0.96	0.98	1.00	0.99	1.00
26	0.27	0.49	0.65	0.83	0.90	0.97	0.98	0.99	1.00	1.00
27	0.53	0.68	0.59	0.67	0.69	0.71	0.76	0.80	0.88	0.93
28	0.90	0.93	0.98	0.99	1.00	1.00	1.00	1.00	1.00	1.00
29	0.95	0.96	0.99	1.00	1.00	1.00	1.00	1.00	1.00	1.00
30	0.79	0.83	0.87	0.89	0.84	0.88	0.91	0.96	0.97	0.97
31	0.09	0.09	0.28	0.41	0.69	0.84	0.89	0.99	1.00	1.00
32	0.04	0.12	0.28	0.40	0.63	0.83	0.94	0.99	1.00	1.00
33	0.11	0.28	0.25	0.45	0.56	0.68	0.80	0.92	0.96	1.00
34	1.00	1.00	1.00	1.00	1.00	1.00	1.00	1.00	1.00	1.00
35	1.00	1.00	1.00	1.00	1.00	1.00	1.00	1.00	1.00	1.00
36	0.97	0.98	1.00	1.00	1.00	1.00	1.00	1.00	1.00	1.00
37	0.03	0.08	0.22	0.51	0.70	0.87	0.95	0.98	0.98	1.00
38	0.04	0.12	0.34	0.53	0.67	0.81	0.97	0.98	1.00	1.00
39	0.05	0.15	0.26	0.52	0.68	0.82	0.88	0.99	1.00	1.00
40	0.16	0.27	0.46	0.79	0.81	0.90	0.99	1.00	1.00	1.00
41	0.24	0.29	0.40	0.76	0.90	0.96	0.97	0.99	1.00	1.00
42	0.13	0.37	0.44	0.75	0.85	0.94	0.97	1.00	1.00	1.00

Table 18: POD according to each scenario and each k2 value, k1 = 10, alpha = 0.05
