# Supplementary material for: Evaluation and comparison of statistical methods for early temporal detection of outbreaks: A simulation-based study
Source: PLoS One. 2017 Jul 17;12(7):e0181227. doi: 10.1371/journal.pone.0181227 (PMC5513450; doi:10.1371/journal.pone.0181227)
Supplement: S10 Appendix — (PDF) [file pone.0181227.s010.pdf]

Evaluation and Comparison of Statistical Methods for Early Temporal Detection of Outbreaks: a Simulation-Based Study

Appendix S10: Overall performances of CUSUM GLM Rossi algorithm ($\alpha = 0.001, 0.01$ and 0.05)

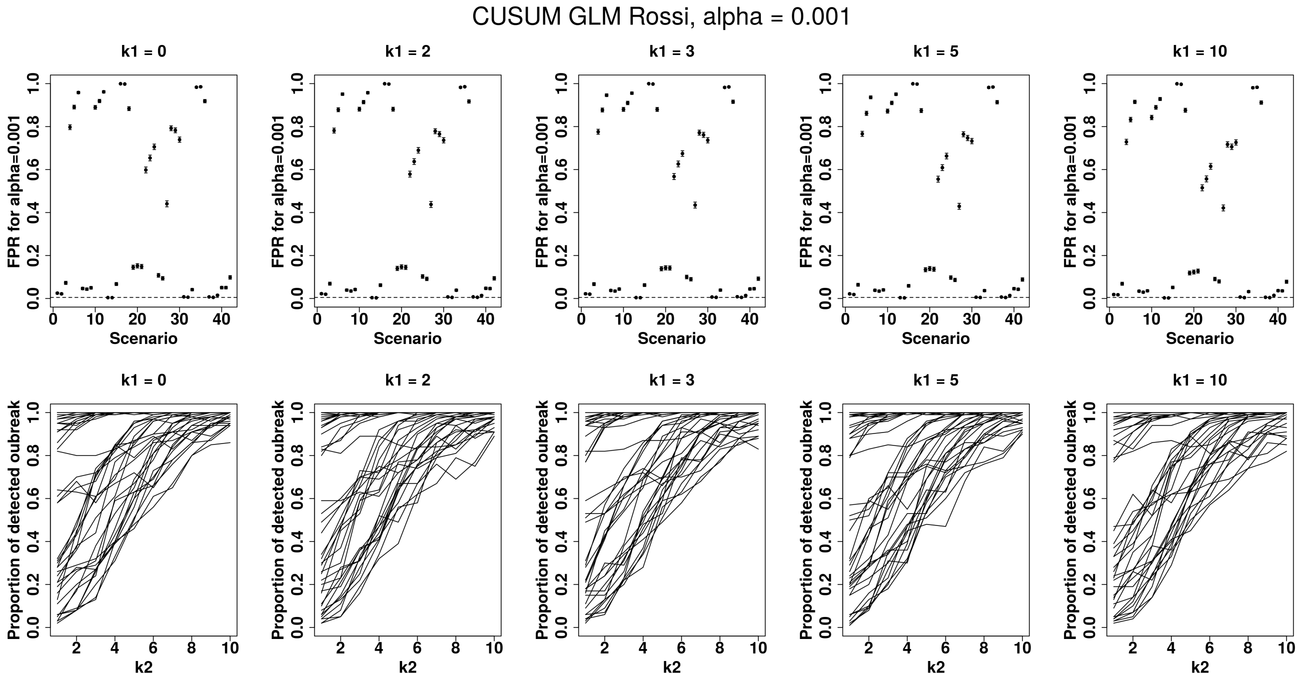

Figure 1: CUSUM GLM Rossi algorithm performances for $\alpha = 0.001$ by increasing past outbreak amplitude k_1 = 0, 2, 3, 5 or 10 with (i) on the first row: false positive rate for 42 simulated scenarios, (ii) on the second row: probability of detection for 42 simulated scenarios (each curve corresponding to a scenario) by increasing current outbreak amplitude k_2 = 1 to 10.

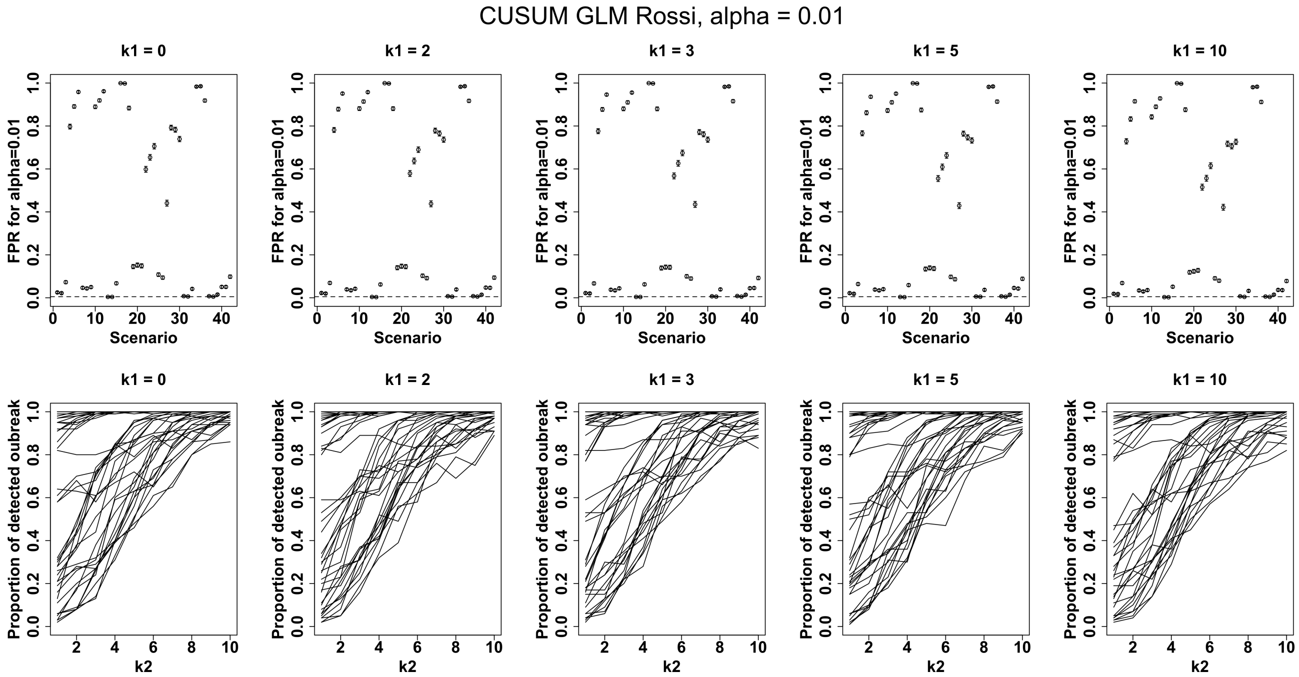

Figure 2: CUSUM GLM Rossi algorithm performances for $\alpha = 0.01$ by increasing past outbreak amplitude k_1 = 0, 2, 3, 5 or 10 with (i) on the first row: false positive rate for 42 simulated scenarios, (ii) on the second row: probability of detection for 42 simulated scenarios (each curve corresponding to a scenario) by increasing current outbreak amplitude k_2 = 1 to 10.

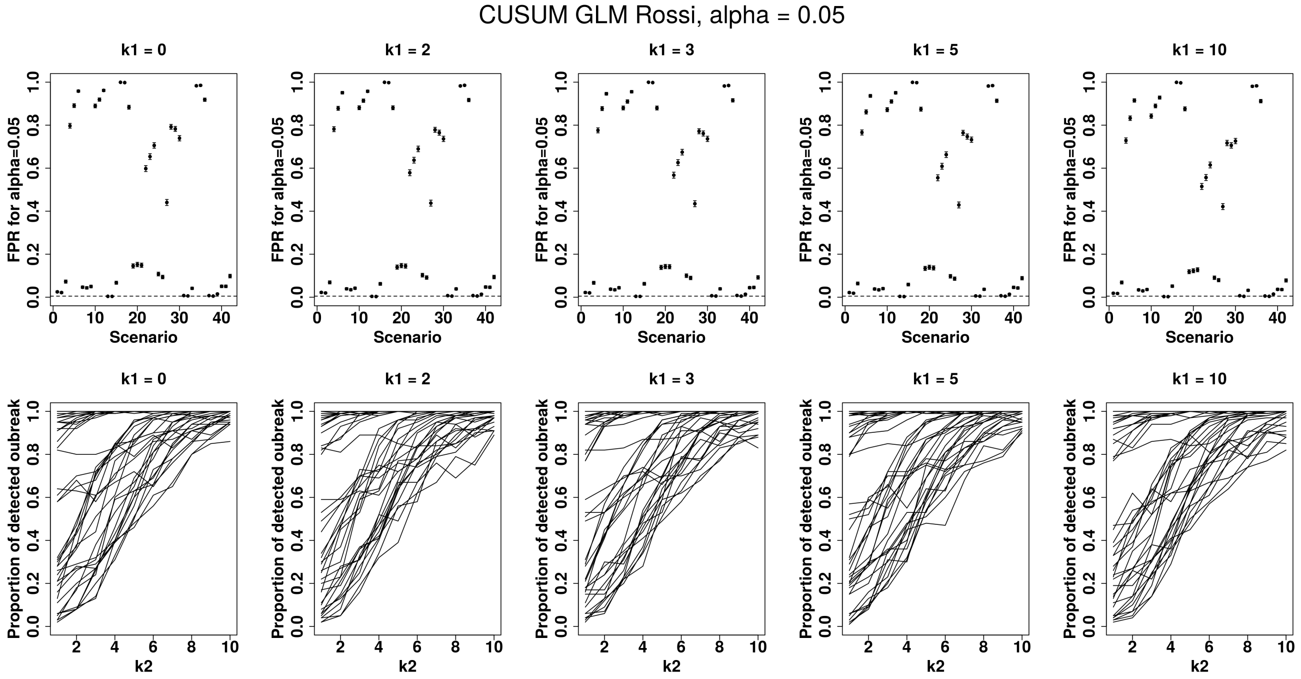

Figure 3: CUSUM GLM Rossi algorithm performances for $\alpha = 0.05$ by increasing past outbreak amplitude $k_1 = 0$, 2, 3, 5 or 10 with (i) on the first row: false positive rate for 42 simulated scenarios, (ii) on the second row: probability of detection for 42 simulated scenarios (each curve corresponding to a scenario) by increasing current outbreak amplitude $k_2 = 1$ to 10.

Overall performances of CUSUM GLM Rossi algorithm ($\alpha = 0.001$)

	FPR k1=0	FPR k1=2	FPR k1=3	FPR k1=5	FPR k1=10
1	0.02	0.02	0.02	0.02	0.02
2	0.02	0.02	0.02	0.02	0.02
3	0.07	0.07	0.07	0.06	0.07
4	0.80	0.78	0.78	0.77	0.73
5	0.89	0.88	0.88	0.86	0.83
6	0.96	0.95	0.95	0.94	0.91
7	0.05	0.04	0.04	0.04	0.03
8	0.04	0.03	0.03	0.03	0.03
9	0.05	0.04	0.04	0.04	0.04
10	0.89	0.88	0.88	0.87	0.84
11	0.92	0.91	0.91	0.91	0.89
12	0.96	0.96	0.96	0.95	0.93
13	0.00	0.00	0.00	0.00	0.00
14	0.00	0.00	0.00	0.00	0.00
15	0.07	0.06	0.06	0.06	0.05
16	1.00	1.00	1.00	1.00	1.00
17	1.00	1.00	1.00	1.00	1.00
18	0.88	0.88	0.88	0.87	0.88
19	0.15	0.14	0.14	0.13	0.12
20	0.15	0.15	0.14	0.14	0.12
21	0.15	0.14	0.14	0.14	0.13
22	0.60	0.58	0.57	0.56	0.52
23	0.65	0.64	0.63	0.61	0.56
24	0.71	0.69	0.67	0.66	0.61
25	0.11	0.10	0.10	0.10	0.09
26	0.09	0.09	0.09	0.09	0.08
27	0.44	0.44	0.43	0.43	0.42
28	0.79	0.78	0.77	0.76	0.72
29	0.78	0.77	0.76	0.75	0.71
30	0.74	0.74	0.74	0.73	0.73
31	0.01	0.01	0.01	0.01	0.01
32	0.01	0.01	0.01	0.00	0.00
33	0.04	0.04	0.04	0.04	0.03
34	0.98	0.98	0.98	0.98	0.98
35	0.99	0.99	0.98	0.98	0.98
36	0.92	0.92	0.92	0.91	0.91
37	0.01	0.01	0.01	0.01	0.01
38	0.01	0.01	0.01	0.00	0.00
39	0.02	0.01	0.01	0.01	0.01
40	0.05	0.05	0.04	0.05	0.04
41	0.05	0.05	0.05	0.04	0.04
42	0.10	0.09	0.09	0.09	0.08

Table 1: FPR according to each scenario and each k1 value, alpha = 0.001

	k2=1	k2=2	k2=3	k2=4	k2=5	k2=6	k2=7	k2=8	k2=9	k2=10
1	0.19	0.28	0.27	0.40	0.63	0.69	0.84	0.94	0.98	0.97
2	0.16	0.26	0.31	0.59	0.63	0.66	0.85	0.94	0.94	0.99
3	0.26	0.29	0.32	0.40	0.46	0.61	0.65	0.80	0.85	0.86
4	0.91	0.96	0.99	1.00	1.00	1.00	1.00	1.00	1.00	1.00
5	0.95	0.98	0.99	1.00	1.00	1.00	1.00	1.00	1.00	1.00
6	0.99	0.99	1.00	1.00	1.00	1.00	1.00	1.00	1.00	1.00
7	0.61	0.70	0.72	0.79	0.84	0.89	0.83	0.91	0.89	0.95
8	0.58	0.65	0.68	0.85	0.79	0.88	0.91	0.87	0.95	0.95
9	0.58	0.68	0.58	0.71	0.78	0.86	0.87	0.91	0.94	0.94
10	0.97	0.98	1.00	1.00	1.00	1.00	1.00	1.00	1.00	1.00
11	0.98	1.00	1.00	1.00	1.00	1.00	1.00	1.00	1.00	1.00
12	0.98	1.00	1.00	1.00	1.00	1.00	1.00	1.00	1.00	1.00
13	0.02	0.08	0.14	0.33	0.51	0.66	0.76	0.85	0.95	0.96
14	0.06	0.08	0.20	0.31	0.50	0.73	0.78	0.86	0.94	0.98
15	0.21	0.27	0.29	0.39	0.48	0.56	0.67	0.80	0.88	0.95
16	1.00	1.00	1.00	1.00	1.00	1.00	1.00	1.00	1.00	1.00
17	1.00	1.00	1.00	1.00	1.00	1.00	1.00	1.00	1.00	1.00
18	0.95	0.95	0.97	1.00	0.99	1.00	1.00	1.00	1.00	1.00
19	0.28	0.49	0.70	0.85	0.95	0.99	1.00	1.00	1.00	1.00
20	0.32	0.47	0.74	0.83	0.96	0.97	1.00	1.00	1.00	1.00
21	0.30	0.43	0.61	0.69	0.89	0.97	0.95	0.99	1.00	1.00
22	0.86	0.96	0.99	1.00	1.00	1.00	1.00	1.00	1.00	1.00
23	0.92	0.92	0.98	1.00	1.00	1.00	1.00	1.00	1.00	1.00
24	0.83	0.93	0.99	0.99	1.00	1.00	1.00	1.00	1.00	1.00
25	0.31	0.44	0.71	0.82	0.93	0.97	1.00	1.00	1.00	1.00
26	0.28	0.37	0.72	0.84	0.95	0.98	0.99	1.00	1.00	1.00
27	0.64	0.63	0.61	0.67	0.72	0.65	0.78	0.86	0.87	0.94
28	0.95	0.94	1.00	1.00	1.00	1.00	1.00	1.00	1.00	1.00
29	0.91	0.97	1.00	0.99	1.00	0.99	1.00	1.00	1.00	1.00
30	0.82	0.80	0.80	0.83	0.87	0.90	0.91	0.92	0.97	0.98
31	0.06	0.09	0.13	0.40	0.60	0.84	0.95	0.98	0.96	1.00
32	0.03	0.08	0.27	0.39	0.56	0.81	0.89	0.97	0.98	1.00
33	0.11	0.20	0.27	0.43	0.60	0.70	0.82	0.91	0.93	0.98
34	1.00	1.00	1.00	1.00	1.00	1.00	1.00	1.00	1.00	1.00
35	1.00	1.00	1.00	1.00	1.00	1.00	1.00	1.00	1.00	1.00
36	0.97	0.99	1.00	1.00	1.00	1.00	1.00	1.00	1.00	1.00
37	0.03	0.18	0.24	0.60	0.67	0.78	0.95	0.97	0.99	1.00
38	0.06	0.08	0.26	0.46	0.69	0.81	0.93	1.00	1.00	1.00
39	0.05	0.17	0.38	0.53	0.65	0.87	0.91	0.96	0.98	1.00
40	0.13	0.45	0.60	0.85	0.86	0.96	0.98	1.00	1.00	1.00
41	0.21	0.38	0.44	0.77	0.85	0.96	0.98	0.99	1.00	1.00
42	0.24	0.36	0.51	0.61	0.87	0.90	0.99	0.98	1.00	1.00

Table 2: POD according to each scenario and each k2 value, k1 = 0, alpha = 0.001

	k2=1	k2=2	k2=3	k2=4	k2=5	k2=6	k2=7	k2=8	k2=9	k2=10
1	0.11	0.23	0.37	0.41	0.67	0.67	0.81	0.94	0.88	0.93
2	0.10	0.28	0.31	0.47	0.65	0.75	0.82	0.85	0.89	0.97
3	0.22	0.27	0.31	0.52	0.49	0.67	0.76	0.69	0.77	0.91
4	0.93	0.99	0.98	1.00	1.00	1.00	1.00	1.00	1.00	1.00
5	0.96	0.99	1.00	1.00	1.00	1.00	1.00	1.00	1.00	1.00
6	0.96	1.00	0.99	1.00	1.00	1.00	1.00	1.00	1.00	0.99
7	0.49	0.57	0.65	0.75	0.72	0.78	0.89	0.83	0.92	0.91
8	0.59	0.59	0.63	0.61	0.72	0.78	0.86	0.92	0.91	0.97
9	0.44	0.57	0.70	0.73	0.78	0.84	0.84	0.85	0.92	0.95
10	0.97	0.99	1.00	1.00	1.00	1.00	1.00	1.00	1.00	1.00
11	0.98	0.99	1.00	0.99	1.00	1.00	1.00	1.00	1.00	1.00
12	1.00	0.99	1.00	1.00	1.00	1.00	1.00	1.00	1.00	1.00
13	0.02	0.05	0.16	0.34	0.56	0.58	0.86	0.91	0.92	0.97
14	0.04	0.05	0.19	0.32	0.39	0.62	0.79	0.89	0.96	0.98
15	0.17	0.19	0.36	0.42	0.55	0.62	0.67	0.79	0.75	0.89
16	1.00	1.00	1.00	1.00	1.00	1.00	1.00	1.00	1.00	1.00
17	1.00	1.00	1.00	1.00	1.00	1.00	1.00	1.00	1.00	1.00
18	0.94	0.98	0.99	0.99	1.00	1.00	1.00	1.00	1.00	1.00
19	0.34	0.48	0.66	0.87	0.96	0.98	1.00	1.00	1.00	1.00
20	0.32	0.51	0.69	0.92	0.96	0.99	1.00	1.00	1.00	1.00
21	0.30	0.37	0.62	0.64	0.87	0.93	0.98	0.98	0.99	1.00
22	0.80	0.93	0.96	1.00	1.00	1.00	1.00	1.00	1.00	1.00
23	0.84	0.87	0.99	1.00	1.00	1.00	1.00	1.00	1.00	1.00
24	0.82	0.88	0.95	0.98	1.00	0.99	1.00	1.00	1.00	1.00
25	0.25	0.47	0.55	0.84	0.96	0.96	1.00	1.00	1.00	1.00
26	0.29	0.46	0.73	0.72	0.94	1.00	1.00	1.00	1.00	1.00
27	0.53	0.60	0.70	0.69	0.76	0.74	0.79	0.81	0.90	0.91
28	0.90	0.95	0.98	1.00	1.00	1.00	1.00	1.00	1.00	1.00
29	0.93	0.98	0.99	0.98	1.00	1.00	1.00	1.00	1.00	1.00
30	0.83	0.81	0.89	0.89	0.85	0.83	0.95	0.94	0.97	0.98
31	0.08	0.13	0.22	0.40	0.63	0.83	0.91	0.97	0.98	1.00
32	0.04	0.11	0.28	0.41	0.64	0.75	0.93	0.97	0.95	0.98
33	0.06	0.21	0.36	0.47	0.58	0.76	0.80	0.87	0.97	0.97
34	1.00	1.00	1.00	1.00	1.00	1.00	1.00	1.00	1.00	1.00
35	1.00	1.00	1.00	1.00	1.00	1.00	1.00	1.00	1.00	1.00
36	0.99	0.99	1.00	1.00	1.00	1.00	1.00	1.00	1.00	1.00
37	0.06	0.14	0.25	0.52	0.77	0.78	0.90	0.98	0.99	1.00
38	0.02	0.08	0.33	0.49	0.68	0.90	0.95	0.98	0.99	1.00
39	0.05	0.15	0.32	0.44	0.65	0.84	0.93	0.98	0.98	1.00
40	0.16	0.45	0.53	0.78	0.92	0.95	0.99	1.00	0.98	1.00
41	0.20	0.23	0.59	0.72	0.82	0.97	0.99	0.98	1.00	1.00
42	0.26	0.30	0.59	0.63	0.82	0.91	0.97	0.99	1.00	1.00

Table 3: POD according to each scenario and each k2 value, k1 = 2, alpha = 0.001

	k2=1	k2=2	k2=3	k2=4	k2=5	k2=6	k2=7	k2=8	k2=9	k2=10
1	0.15	0.15	0.26	0.52	0.67	0.71	0.83	0.93	0.93	0.95
2	0.11	0.30	0.29	0.46	0.61	0.73	0.91	0.86	0.96	0.94
3	0.18	0.28	0.35	0.44	0.59	0.62	0.68	0.78	0.86	0.88
4	0.96	0.95	0.98	1.00	1.00	1.00	1.00	1.00	1.00	1.00
5	0.96	0.99	0.98	0.99	1.00	1.00	1.00	1.00	1.00	1.00
6	0.97	1.00	0.98	1.00	0.98	1.00	1.00	1.00	1.00	1.00
7	0.49	0.53	0.62	0.69	0.77	0.81	0.91	0.95	0.90	0.92
8	0.51	0.59	0.70	0.72	0.66	0.78	0.87	0.84	0.88	0.89
9	0.59	0.64	0.69	0.74	0.69	0.78	0.78	0.90	0.88	0.95
10	0.94	0.99	1.00	1.00	1.00	1.00	1.00	1.00	1.00	1.00
11	0.98	0.99	1.00	1.00	1.00	1.00	1.00	1.00	1.00	1.00
12	1.00	1.00	1.00	1.00	1.00	1.00	1.00	1.00	1.00	1.00
13	0.02	0.12	0.22	0.28	0.56	0.63	0.77	0.85	0.96	0.99
14	0.06	0.07	0.22	0.34	0.51	0.67	0.77	0.91	0.92	0.96
15	0.18	0.24	0.34	0.41	0.49	0.58	0.70	0.77	0.88	0.83
16	1.00	1.00	1.00	1.00	1.00	1.00	1.00	1.00	1.00	1.00
17	1.00	1.00	1.00	1.00	1.00	1.00	1.00	1.00	1.00	1.00
18	0.94	0.93	0.99	1.00	1.00	1.00	1.00	1.00	1.00	1.00
19	0.31	0.53	0.66	0.84	0.94	0.95	1.00	0.99	1.00	1.00
20	0.29	0.57	0.74	0.86	0.94	0.97	1.00	1.00	1.00	1.00
21	0.28	0.42	0.61	0.70	0.81	0.91	0.91	0.98	0.99	1.00
22	0.80	0.95	0.98	1.00	1.00	1.00	1.00	1.00	1.00	1.00
23	0.77	0.94	0.98	1.00	1.00	1.00	1.00	1.00	1.00	1.00
24	0.77	0.93	0.94	0.97	0.99	1.00	1.00	1.00	1.00	1.00
25	0.22	0.42	0.66	0.84	0.96	0.98	1.00	1.00	1.00	1.00
26	0.21	0.41	0.66	0.82	0.96	0.98	0.98	1.00	1.00	1.00
27	0.53	0.53	0.60	0.66	0.70	0.70	0.73	0.83	0.85	0.89
28	0.94	0.97	0.97	1.00	1.00	1.00	1.00	1.00	1.00	1.00
29	0.89	0.95	1.00	1.00	0.99	1.00	1.00	1.00	1.00	1.00
30	0.82	0.82	0.83	0.87	0.87	0.88	0.97	0.95	0.93	0.97
31	0.03	0.13	0.20	0.35	0.59	0.93	0.90	0.98	0.99	1.00
32	0.04	0.06	0.23	0.37	0.64	0.76	0.91	0.97	0.98	1.00
33	0.17	0.17	0.32	0.48	0.53	0.79	0.77	0.93	0.96	1.00
34	0.98	1.00	1.00	1.00	1.00	1.00	1.00	1.00	1.00	1.00
35	1.00	1.00	1.00	1.00	1.00	1.00	1.00	1.00	1.00	1.00
36	0.96	0.98	1.00	1.00	1.00	1.00	1.00	1.00	1.00	1.00
37	0.06	0.13	0.38	0.49	0.70	0.88	0.92	0.99	0.98	1.00
38	0.04	0.09	0.23	0.54	0.69	0.88	0.92	0.95	0.99	1.00
39	0.09	0.20	0.31	0.51	0.61	0.75	0.91	0.95	1.00	1.00
40	0.16	0.45	0.55	0.73	0.86	0.99	0.99	0.99	1.00	1.00
41	0.18	0.24	0.39	0.69	0.82	0.96	0.98	1.00	1.00	1.00
42	0.22	0.43	0.54	0.65	0.89	0.93	0.99	1.00	1.00	1.00

Table 4: POD according to each scenario and each k2 value, k1 = 3, alpha = 0.001

	k2=1	k2=2	k2=3	k2=4	k2=5	k2=6	k2=7	k2=8	k2=9	k2=10
1	0.15	0.30	0.34	0.47	0.64	0.63	0.87	0.95	0.99	0.96
2	0.15	0.20	0.40	0.53	0.53	0.73	0.83	0.88	0.93	0.97
3	0.23	0.29	0.35	0.48	0.51	0.60	0.72	0.76	0.79	0.91
4	0.88	0.94	0.97	1.00	1.00	1.00	1.00	1.00	1.00	1.00
5	0.94	0.97	1.00	1.00	1.00	0.99	1.00	1.00	1.00	1.00
6	0.98	0.99	1.00	1.00	0.99	0.99	1.00	1.00	1.00	1.00
7	0.45	0.59	0.55	0.68	0.78	0.77	0.83	0.86	0.93	0.95
8	0.57	0.58	0.70	0.70	0.75	0.72	0.80	0.90	0.89	0.92
9	0.52	0.55	0.66	0.55	0.69	0.81	0.86	0.86	0.91	0.93
10	0.99	0.98	0.99	1.00	1.00	1.00	1.00	1.00	1.00	1.00
11	0.98	0.99	0.99	1.00	1.00	1.00	1.00	1.00	1.00	1.00
12	0.99	0.99	1.00	1.00	1.00	1.00	1.00	1.00	1.00	1.00
13	0.03	0.13	0.18	0.31	0.54	0.70	0.75	0.89	0.96	0.95
14	0.01	0.09	0.21	0.32	0.54	0.64	0.80	0.92	0.97	0.95
15	0.18	0.23	0.30	0.45	0.48	0.47	0.68	0.84	0.82	0.92
16	1.00	1.00	1.00	1.00	1.00	1.00	1.00	1.00	1.00	1.00
17	1.00	1.00	1.00	1.00	1.00	1.00	1.00	1.00	1.00	1.00
18	0.93	0.96	0.97	1.00	1.00	1.00	1.00	1.00	1.00	1.00
19	0.32	0.60	0.65	0.86	0.96	0.98	0.99	0.99	1.00	1.00
20	0.31	0.43	0.72	0.88	0.95	0.99	0.99	1.00	1.00	1.00
21	0.29	0.46	0.51	0.73	0.87	0.90	0.97	0.99	1.00	1.00
22	0.80	0.92	0.99	1.00	1.00	1.00	1.00	1.00	1.00	1.00
23	0.79	0.95	0.98	1.00	1.00	0.99	1.00	1.00	1.00	1.00
24	0.88	0.90	0.91	0.96	1.00	1.00	0.99	1.00	1.00	1.00
25	0.26	0.46	0.61	0.84	0.94	0.99	0.99	1.00	1.00	1.00
26	0.20	0.43	0.64	0.81	0.95	0.97	0.99	1.00	1.00	1.00
27	0.50	0.52	0.72	0.72	0.76	0.73	0.75	0.77	0.81	0.90
28	0.90	0.98	1.00	0.99	1.00	1.00	1.00	1.00	1.00	1.00
29	0.90	0.95	0.96	1.00	0.99	1.00	1.00	1.00	1.00	1.00
30	0.80	0.83	0.85	0.84	0.88	0.92	0.93	0.95	0.98	0.99
31	0.06	0.10	0.31	0.30	0.60	0.78	0.91	0.95	1.00	1.00
32	0.05	0.16	0.23	0.47	0.59	0.79	0.92	0.96	1.00	1.00
33	0.20	0.28	0.36	0.36	0.58	0.71	0.79	0.90	0.96	0.99
34	0.99	1.00	1.00	1.00	1.00	1.00	1.00	1.00	1.00	1.00
35	0.98	1.00	1.00	1.00	1.00	1.00	1.00	1.00	1.00	1.00
36	0.99	0.98	0.98	1.00	0.99	1.00	1.00	1.00	1.00	1.00
37	0.02	0.08	0.27	0.44	0.69	0.84	0.94	0.98	0.97	1.00
38	0.01	0.10	0.20	0.39	0.75	0.89	0.94	0.99	1.00	1.00
39	0.06	0.09	0.35	0.40	0.61	0.81	0.89	0.99	0.99	1.00
40	0.19	0.28	0.59	0.69	0.79	0.92	1.00	0.99	1.00	1.00
41	0.21	0.29	0.57	0.71	0.85	0.93	1.00	1.00	1.00	1.00
42	0.24	0.32	0.43	0.68	0.87	0.91	0.96	1.00	1.00	1.00

Table 5: POD according to each scenario and each k2 value, k1 = 5, alpha = 0.001

	k2=1	k2=2	k2=3	k2=4	k2=5	k2=6	k2=7	k2=8	k2=9	k2=10
1	0.19	0.19	0.30	0.46	0.57	0.74	0.87	0.84	0.91	0.95
2	0.15	0.23	0.31	0.40	0.64	0.74	0.86	0.88	0.92	0.98
3	0.24	0.27	0.31	0.41	0.45	0.57	0.64	0.74	0.77	0.82
4	0.92	0.94	0.96	0.99	1.00	0.99	1.00	1.00	1.00	1.00
5	0.97	0.98	0.97	0.99	1.00	1.00	1.00	1.00	1.00	1.00
6	0.95	0.98	0.97	0.99	0.97	0.98	1.00	1.00	1.00	1.00
7	0.37	0.45	0.58	0.62	0.66	0.76	0.82	0.79	0.91	0.88
8	0.39	0.54	0.57	0.64	0.89	0.81	0.86	0.78	0.90	0.89
9	0.47	0.48	0.64	0.58	0.73	0.76	0.78	0.91	0.90	0.91
10	0.95	0.99	1.00	1.00	1.00	1.00	1.00	1.00	1.00	1.00
11	0.98	0.98	0.99	0.99	1.00	1.00	0.99	1.00	1.00	1.00
12	0.98	1.00	1.00	1.00	1.00	1.00	1.00	1.00	1.00	1.00
13	0.03	0.07	0.14	0.35	0.48	0.65	0.76	0.83	0.95	0.99
14	0.02	0.04	0.14	0.29	0.53	0.65	0.82	0.87	0.94	0.93
15	0.11	0.14	0.38	0.36	0.50	0.58	0.67	0.79	0.81	0.88
16	1.00	1.00	1.00	1.00	1.00	1.00	1.00	1.00	1.00	1.00
17	1.00	1.00	1.00	1.00	1.00	1.00	1.00	1.00	1.00	1.00
18	0.92	0.94	0.99	0.99	1.00	1.00	1.00	1.00	1.00	1.00
19	0.23	0.50	0.63	0.75	0.91	0.97	1.00	1.00	1.00	1.00
20	0.33	0.49	0.59	0.83	0.89	0.97	1.00	0.99	0.99	1.00
21	0.33	0.39	0.52	0.77	0.79	0.88	0.96	0.97	0.98	1.00
22	0.78	0.91	0.97	0.99	1.00	1.00	1.00	1.00	1.00	1.00
23	0.77	0.86	0.97	0.98	1.00	1.00	1.00	1.00	1.00	1.00
24	0.87	0.83	0.94	0.94	0.97	1.00	1.00	1.00	1.00	1.00
25	0.28	0.37	0.66	0.81	0.90	0.96	0.98	1.00	0.99	1.00
26	0.26	0.43	0.61	0.78	0.87	0.97	0.98	0.99	1.00	1.00
27	0.45	0.62	0.52	0.62	0.63	0.67	0.69	0.74	0.86	0.85
28	0.85	0.91	0.98	0.98	1.00	1.00	1.00	1.00	1.00	1.00
29	0.89	0.94	0.98	0.99	1.00	1.00	1.00	1.00	1.00	1.00
30	0.79	0.82	0.86	0.87	0.84	0.85	0.91	0.96	0.97	0.97
31	0.05	0.07	0.25	0.37	0.65	0.78	0.88	0.98	1.00	1.00
32	0.04	0.10	0.24	0.33	0.57	0.80	0.93	0.98	1.00	1.00
33	0.09	0.26	0.22	0.39	0.56	0.61	0.77	0.90	0.95	1.00
34	1.00	1.00	1.00	1.00	1.00	1.00	1.00	1.00	1.00	1.00
35	1.00	1.00	1.00	1.00	1.00	1.00	1.00	1.00	1.00	1.00
36	0.94	0.98	1.00	1.00	1.00	1.00	1.00	1.00	1.00	1.00
37	0.03	0.05	0.19	0.48	0.65	0.87	0.94	0.98	0.98	1.00
38	0.04	0.12	0.31	0.47	0.66	0.80	0.97	0.98	1.00	1.00
39	0.05	0.14	0.24	0.44	0.63	0.80	0.86	0.99	0.99	1.00
40	0.14	0.26	0.43	0.75	0.81	0.90	0.98	0.99	1.00	1.00
41	0.22	0.27	0.38	0.72	0.90	0.95	0.97	0.99	1.00	1.00
42	0.13	0.35	0.43	0.74	0.84	0.93	0.97	0.99	1.00	1.00

Table 6: POD according to each scenario and each k2 value, k1 = 10, alpha = 0.001

Overall performances of CUSUM GLM Rossi algorithm ($\alpha = 0.01$)

	FPR k1=0	FPR k1=2	FPR k1=3	FPR k1=5	FPR k1=10
1	0.02	0.02	0.02	0.02	0.02
2	0.02	0.02	0.02	0.02	0.02
3	0.07	0.07	0.07	0.06	0.07
4	0.80	0.78	0.78	0.77	0.73
5	0.89	0.88	0.88	0.86	0.83
6	0.96	0.95	0.95	0.94	0.91
7	0.05	0.04	0.04	0.04	0.03
8	0.04	0.03	0.03	0.03	0.03
9	0.05	0.04	0.04	0.04	0.04
10	0.89	0.88	0.88	0.87	0.84
11	0.92	0.91	0.91	0.91	0.89
12	0.96	0.96	0.96	0.95	0.93
13	0.00	0.00	0.00	0.00	0.00
14	0.00	0.00	0.00	0.00	0.00
15	0.07	0.06	0.06	0.06	0.05
16	1.00	1.00	1.00	1.00	1.00
17	1.00	1.00	1.00	1.00	1.00
18	0.88	0.88	0.88	0.87	0.88
19	0.15	0.14	0.14	0.13	0.12
20	0.15	0.15	0.14	0.14	0.12
21	0.15	0.14	0.14	0.14	0.13
22	0.60	0.58	0.57	0.56	0.52
23	0.65	0.64	0.63	0.61	0.56
24	0.71	0.69	0.67	0.66	0.61
25	0.11	0.10	0.10	0.10	0.09
26	0.09	0.09	0.09	0.09	0.08
27	0.44	0.44	0.43	0.43	0.42
28	0.79	0.78	0.77	0.76	0.72
29	0.78	0.77	0.76	0.75	0.71
30	0.74	0.74	0.74	0.73	0.73
31	0.01	0.01	0.01	0.01	0.01
32	0.01	0.01	0.01	0.00	0.00
33	0.04	0.04	0.04	0.04	0.03
34	0.98	0.98	0.98	0.98	0.98
35	0.99	0.99	0.98	0.98	0.98
36	0.92	0.92	0.92	0.91	0.91
37	0.01	0.01	0.01	0.01	0.01
38	0.01	0.01	0.01	0.00	0.00
39	0.02	0.01	0.01	0.01	0.01
40	0.05	0.05	0.04	0.05	0.04
41	0.05	0.05	0.05	0.04	0.04
42	0.10	0.09	0.09	0.09	0.08

Table 7: FPR according to each scenario and each k1 value, alpha = 0.01

	k2=1	k2=2	k2=3	k2=4	k2=5	k2=6	k2=7	k2=8	k2=9	k2=10
1	0.19	0.28	0.27	0.40	0.63	0.69	0.84	0.94	0.98	0.97
2	0.16	0.26	0.31	0.59	0.63	0.66	0.85	0.94	0.94	0.99
3	0.26	0.29	0.32	0.40	0.46	0.61	0.65	0.80	0.85	0.86
4	0.91	0.96	0.99	1.00	1.00	1.00	1.00	1.00	1.00	1.00
5	0.95	0.98	0.99	1.00	1.00	1.00	1.00	1.00	1.00	1.00
6	0.99	0.99	1.00	1.00	1.00	1.00	1.00	1.00	1.00	1.00
7	0.61	0.70	0.72	0.79	0.84	0.89	0.83	0.91	0.89	0.95
8	0.58	0.65	0.68	0.85	0.79	0.88	0.91	0.87	0.95	0.95
9	0.58	0.68	0.58	0.71	0.78	0.86	0.87	0.91	0.94	0.94
10	0.97	0.98	1.00	1.00	1.00	1.00	1.00	1.00	1.00	1.00
11	0.98	1.00	1.00	1.00	1.00	1.00	1.00	1.00	1.00	1.00
12	0.98	1.00	1.00	1.00	1.00	1.00	1.00	1.00	1.00	1.00
13	0.02	0.08	0.14	0.33	0.51	0.66	0.76	0.85	0.95	0.96
14	0.06	0.08	0.20	0.31	0.50	0.73	0.78	0.86	0.94	0.98
15	0.21	0.27	0.29	0.39	0.48	0.56	0.67	0.80	0.88	0.95
16	1.00	1.00	1.00	1.00	1.00	1.00	1.00	1.00	1.00	1.00
17	1.00	1.00	1.00	1.00	1.00	1.00	1.00	1.00	1.00	1.00
18	0.95	0.95	0.97	1.00	0.99	1.00	1.00	1.00	1.00	1.00
19	0.28	0.49	0.70	0.85	0.95	0.99	1.00	1.00	1.00	1.00
20	0.32	0.47	0.74	0.83	0.96	0.97	1.00	1.00	1.00	1.00
21	0.30	0.43	0.61	0.69	0.89	0.97	0.95	0.99	1.00	1.00
22	0.86	0.96	0.99	1.00	1.00	1.00	1.00	1.00	1.00	1.00
23	0.92	0.92	0.98	1.00	1.00	1.00	1.00	1.00	1.00	1.00
24	0.83	0.93	0.99	0.99	1.00	1.00	1.00	1.00	1.00	1.00
25	0.31	0.44	0.71	0.82	0.93	0.97	1.00	1.00	1.00	1.00
26	0.28	0.37	0.72	0.84	0.95	0.98	0.99	1.00	1.00	1.00
27	0.64	0.63	0.61	0.67	0.72	0.65	0.78	0.86	0.87	0.94
28	0.95	0.94	1.00	1.00	1.00	1.00	1.00	1.00	1.00	1.00
29	0.91	0.97	1.00	0.99	1.00	0.99	1.00	1.00	1.00	1.00
30	0.82	0.80	0.80	0.83	0.87	0.90	0.91	0.92	0.97	0.98
31	0.06	0.09	0.13	0.40	0.60	0.84	0.95	0.98	0.96	1.00
32	0.03	0.08	0.27	0.39	0.56	0.81	0.89	0.97	0.98	1.00
33	0.11	0.20	0.27	0.43	0.60	0.70	0.82	0.91	0.93	0.98
34	1.00	1.00	1.00	1.00	1.00	1.00	1.00	1.00	1.00	1.00
35	1.00	1.00	1.00	1.00	1.00	1.00	1.00	1.00	1.00	1.00
36	0.97	0.99	1.00	1.00	1.00	1.00	1.00	1.00	1.00	1.00
37	0.03	0.18	0.24	0.60	0.67	0.78	0.95	0.97	0.99	1.00
38	0.06	0.08	0.26	0.46	0.69	0.81	0.93	1.00	1.00	1.00
39	0.05	0.17	0.38	0.53	0.65	0.87	0.91	0.96	0.98	1.00
40	0.13	0.45	0.60	0.85	0.86	0.96	0.98	1.00	1.00	1.00
41	0.21	0.38	0.44	0.77	0.85	0.96	0.98	0.99	1.00	1.00
42	0.24	0.36	0.51	0.61	0.87	0.90	0.99	0.98	1.00	1.00

Table 8: POD according to each scenario and each k2 value, k1 = 0, alpha = 0.01

	k2=1	k2=2	k2=3	k2=4	k2=5	k2=6	k2=7	k2=8	k2=9	k2=10
1	0.11	0.23	0.37	0.41	0.67	0.67	0.81	0.94	0.88	0.93
2	0.10	0.28	0.31	0.47	0.65	0.75	0.82	0.85	0.89	0.97
3	0.22	0.27	0.31	0.52	0.49	0.67	0.76	0.69	0.77	0.91
4	0.93	0.99	0.98	1.00	1.00	1.00	1.00	1.00	1.00	1.00
5	0.96	0.99	1.00	1.00	1.00	1.00	1.00	1.00	1.00	1.00
6	0.96	1.00	0.99	1.00	1.00	1.00	1.00	1.00	1.00	0.99
7	0.49	0.57	0.65	0.75	0.72	0.78	0.89	0.83	0.92	0.91
8	0.59	0.59	0.63	0.61	0.72	0.78	0.86	0.92	0.91	0.97
9	0.44	0.57	0.70	0.73	0.78	0.84	0.84	0.85	0.92	0.95
10	0.97	0.99	1.00	1.00	1.00	1.00	1.00	1.00	1.00	1.00
11	0.98	0.99	1.00	0.99	1.00	1.00	1.00	1.00	1.00	1.00
12	1.00	0.99	1.00	1.00	1.00	1.00	1.00	1.00	1.00	1.00
13	0.02	0.05	0.16	0.34	0.56	0.58	0.86	0.91	0.92	0.97
14	0.04	0.05	0.19	0.32	0.39	0.62	0.79	0.89	0.96	0.98
15	0.17	0.19	0.36	0.42	0.55	0.62	0.67	0.79	0.75	0.89
16	1.00	1.00	1.00	1.00	1.00	1.00	1.00	1.00	1.00	1.00
17	1.00	1.00	1.00	1.00	1.00	1.00	1.00	1.00	1.00	1.00
18	0.94	0.98	0.99	0.99	1.00	1.00	1.00	1.00	1.00	1.00
19	0.34	0.48	0.66	0.87	0.96	0.98	1.00	1.00	1.00	1.00
20	0.32	0.51	0.69	0.92	0.96	0.99	1.00	1.00	1.00	1.00
21	0.30	0.37	0.62	0.64	0.87	0.93	0.98	0.98	0.99	1.00
22	0.80	0.93	0.96	1.00	1.00	1.00	1.00	1.00	1.00	1.00
23	0.84	0.87	0.99	1.00	1.00	1.00	1.00	1.00	1.00	1.00
24	0.82	0.88	0.95	0.98	1.00	0.99	1.00	1.00	1.00	1.00
25	0.25	0.47	0.55	0.84	0.96	0.96	1.00	1.00	1.00	1.00
26	0.29	0.46	0.73	0.72	0.94	1.00	1.00	1.00	1.00	1.00
27	0.53	0.60	0.70	0.69	0.76	0.74	0.79	0.81	0.90	0.91
28	0.90	0.95	0.98	1.00	1.00	1.00	1.00	1.00	1.00	1.00
29	0.93	0.98	0.99	0.98	1.00	1.00	1.00	1.00	1.00	1.00
30	0.83	0.81	0.89	0.89	0.85	0.83	0.95	0.94	0.97	0.98
31	0.08	0.13	0.22	0.40	0.63	0.83	0.91	0.97	0.98	1.00
32	0.04	0.11	0.28	0.41	0.64	0.75	0.93	0.97	0.95	0.98
33	0.06	0.21	0.36	0.47	0.58	0.76	0.80	0.87	0.97	0.97
34	1.00	1.00	1.00	1.00	1.00	1.00	1.00	1.00	1.00	1.00
35	1.00	1.00	1.00	1.00	1.00	1.00	1.00	1.00	1.00	1.00
36	0.99	0.99	1.00	1.00	1.00	1.00	1.00	1.00	1.00	1.00
37	0.06	0.14	0.25	0.52	0.77	0.78	0.90	0.98	0.99	1.00
38	0.02	0.08	0.33	0.49	0.68	0.90	0.95	0.98	0.99	1.00
39	0.05	0.15	0.32	0.44	0.65	0.84	0.93	0.98	0.98	1.00
40	0.16	0.45	0.53	0.78	0.92	0.95	0.99	1.00	0.98	1.00
41	0.20	0.23	0.59	0.72	0.82	0.97	0.99	0.98	1.00	1.00
42	0.26	0.30	0.59	0.63	0.82	0.91	0.97	0.99	1.00	1.00

Table 9: POD according to each scenario and each k2 value, k1 = 2, alpha = 0.01

	k2=1	k2=2	k2=3	k2=4	k2=5	k2=6	k2=7	k2=8	k2=9	k2=10
1	0.15	0.15	0.26	0.52	0.67	0.71	0.83	0.93	0.93	0.95
2	0.11	0.30	0.29	0.46	0.61	0.73	0.91	0.86	0.96	0.94
3	0.18	0.28	0.35	0.44	0.59	0.62	0.68	0.78	0.86	0.88
4	0.96	0.95	0.98	1.00	1.00	1.00	1.00	1.00	1.00	1.00
5	0.96	0.99	0.98	0.99	1.00	1.00	1.00	1.00	1.00	1.00
6	0.97	1.00	0.98	1.00	0.98	1.00	1.00	1.00	1.00	1.00
7	0.49	0.53	0.62	0.69	0.77	0.81	0.91	0.95	0.90	0.92
8	0.51	0.59	0.70	0.72	0.66	0.78	0.87	0.84	0.88	0.89
9	0.59	0.64	0.69	0.74	0.69	0.78	0.78	0.90	0.88	0.95
10	0.94	0.99	1.00	1.00	1.00	1.00	1.00	1.00	1.00	1.00
11	0.98	0.99	1.00	1.00	1.00	1.00	1.00	1.00	1.00	1.00
12	1.00	1.00	1.00	1.00	1.00	1.00	1.00	1.00	1.00	1.00
13	0.02	0.12	0.22	0.28	0.56	0.63	0.77	0.85	0.96	0.99
14	0.06	0.07	0.22	0.34	0.51	0.67	0.77	0.91	0.92	0.96
15	0.18	0.24	0.34	0.41	0.49	0.58	0.70	0.77	0.88	0.83
16	1.00	1.00	1.00	1.00	1.00	1.00	1.00	1.00	1.00	1.00
17	1.00	1.00	1.00	1.00	1.00	1.00	1.00	1.00	1.00	1.00
18	0.94	0.93	0.99	1.00	1.00	1.00	1.00	1.00	1.00	1.00
19	0.31	0.53	0.66	0.84	0.94	0.95	1.00	0.99	1.00	1.00
20	0.29	0.57	0.74	0.86	0.94	0.97	1.00	1.00	1.00	1.00
21	0.28	0.42	0.61	0.70	0.81	0.91	0.91	0.98	0.99	1.00
22	0.80	0.95	0.98	1.00	1.00	1.00	1.00	1.00	1.00	1.00
23	0.77	0.94	0.98	1.00	1.00	1.00	1.00	1.00	1.00	1.00
24	0.77	0.93	0.94	0.97	0.99	1.00	1.00	1.00	1.00	1.00
25	0.22	0.42	0.66	0.84	0.96	0.98	1.00	1.00	1.00	1.00
26	0.21	0.41	0.66	0.82	0.96	0.98	0.98	1.00	1.00	1.00
27	0.53	0.53	0.60	0.66	0.70	0.70	0.73	0.83	0.85	0.89
28	0.94	0.97	0.97	1.00	1.00	1.00	1.00	1.00	1.00	1.00
29	0.89	0.95	1.00	1.00	0.99	1.00	1.00	1.00	1.00	1.00
30	0.82	0.82	0.83	0.87	0.87	0.88	0.97	0.95	0.93	0.97
31	0.03	0.13	0.20	0.35	0.59	0.93	0.90	0.98	0.99	1.00
32	0.04	0.06	0.23	0.37	0.64	0.76	0.91	0.97	0.98	1.00
33	0.17	0.17	0.32	0.48	0.53	0.79	0.77	0.93	0.96	1.00
34	0.98	1.00	1.00	1.00	1.00	1.00	1.00	1.00	1.00	1.00
35	1.00	1.00	1.00	1.00	1.00	1.00	1.00	1.00	1.00	1.00
36	0.96	0.98	1.00	1.00	1.00	1.00	1.00	1.00	1.00	1.00
37	0.06	0.13	0.38	0.49	0.70	0.88	0.92	0.99	0.98	1.00
38	0.04	0.09	0.23	0.54	0.69	0.88	0.92	0.95	0.99	1.00
39	0.09	0.20	0.31	0.51	0.61	0.75	0.91	0.95	1.00	1.00
40	0.16	0.45	0.55	0.73	0.86	0.99	0.99	0.99	1.00	1.00
41	0.18	0.24	0.39	0.69	0.82	0.96	0.98	1.00	1.00	1.00
42	0.22	0.43	0.54	0.65	0.89	0.93	0.99	1.00	1.00	1.00

Table 10: POD according to each scenario and each k2 value, k1 = 3, alpha = 0.01

	k2=1	k2=2	k2=3	k2=4	k2=5	k2=6	k2=7	k2=8	k2=9	k2=10
1	0.15	0.30	0.34	0.47	0.64	0.63	0.87	0.95	0.99	0.96
2	0.15	0.20	0.40	0.53	0.53	0.73	0.83	0.88	0.93	0.97
3	0.23	0.29	0.35	0.48	0.51	0.60	0.72	0.76	0.79	0.91
4	0.88	0.94	0.97	1.00	1.00	1.00	1.00	1.00	1.00	1.00
5	0.94	0.97	1.00	1.00	1.00	0.99	1.00	1.00	1.00	1.00
6	0.98	0.99	1.00	1.00	0.99	0.99	1.00	1.00	1.00	1.00
7	0.45	0.59	0.55	0.68	0.78	0.77	0.83	0.86	0.93	0.95
8	0.57	0.58	0.70	0.70	0.75	0.72	0.80	0.90	0.89	0.92
9	0.52	0.55	0.66	0.55	0.69	0.81	0.86	0.86	0.91	0.93
10	0.99	0.98	0.99	1.00	1.00	1.00	1.00	1.00	1.00	1.00
11	0.98	0.99	0.99	1.00	1.00	1.00	1.00	1.00	1.00	1.00
12	0.99	0.99	1.00	1.00	1.00	1.00	1.00	1.00	1.00	1.00
13	0.03	0.13	0.18	0.31	0.54	0.70	0.75	0.89	0.96	0.95
14	0.01	0.09	0.21	0.32	0.54	0.64	0.80	0.92	0.97	0.95
15	0.18	0.23	0.30	0.45	0.48	0.47	0.68	0.84	0.82	0.92
16	1.00	1.00	1.00	1.00	1.00	1.00	1.00	1.00	1.00	1.00
17	1.00	1.00	1.00	1.00	1.00	1.00	1.00	1.00	1.00	1.00
18	0.93	0.96	0.97	1.00	1.00	1.00	1.00	1.00	1.00	1.00
19	0.32	0.60	0.65	0.86	0.96	0.98	0.99	0.99	1.00	1.00
20	0.31	0.43	0.72	0.88	0.95	0.99	0.99	1.00	1.00	1.00
21	0.29	0.46	0.51	0.73	0.87	0.90	0.97	0.99	1.00	1.00
22	0.80	0.92	0.99	1.00	1.00	1.00	1.00	1.00	1.00	1.00
23	0.79	0.95	0.98	1.00	1.00	0.99	1.00	1.00	1.00	1.00
24	0.88	0.90	0.91	0.96	1.00	1.00	0.99	1.00	1.00	1.00
25	0.26	0.46	0.61	0.84	0.94	0.99	0.99	1.00	1.00	1.00
26	0.20	0.43	0.64	0.81	0.95	0.97	0.99	1.00	1.00	1.00
27	0.50	0.52	0.72	0.72	0.76	0.73	0.75	0.77	0.81	0.90
28	0.90	0.98	1.00	0.99	1.00	1.00	1.00	1.00	1.00	1.00
29	0.90	0.95	0.96	1.00	0.99	1.00	1.00	1.00	1.00	1.00
30	0.80	0.83	0.85	0.84	0.88	0.92	0.93	0.95	0.98	0.99
31	0.06	0.10	0.31	0.30	0.60	0.78	0.91	0.95	1.00	1.00
32	0.05	0.16	0.23	0.47	0.59	0.79	0.92	0.96	1.00	1.00
33	0.20	0.28	0.36	0.36	0.58	0.71	0.79	0.90	0.96	0.99
34	0.99	1.00	1.00	1.00	1.00	1.00	1.00	1.00	1.00	1.00
35	0.98	1.00	1.00	1.00	1.00	1.00	1.00	1.00	1.00	1.00
36	0.99	0.98	0.98	1.00	0.99	1.00	1.00	1.00	1.00	1.00
37	0.02	0.08	0.27	0.44	0.69	0.84	0.94	0.98	0.97	1.00
38	0.01	0.10	0.20	0.39	0.75	0.89	0.94	0.99	1.00	1.00
39	0.06	0.09	0.35	0.40	0.61	0.81	0.89	0.99	0.99	1.00
40	0.19	0.28	0.59	0.69	0.79	0.92	1.00	0.99	1.00	1.00
41	0.21	0.29	0.57	0.71	0.85	0.93	1.00	1.00	1.00	1.00
42	0.24	0.32	0.43	0.68	0.87	0.91	0.96	1.00	1.00	1.00

Table 11: POD according to each scenario and each k2 value, k1 = 5, alpha = 0.01

	k2=1	k2=2	k2=3	k2=4	k2=5	k2=6	k2=7	k2=8	k2=9	k2=10
1	0.19	0.19	0.30	0.46	0.57	0.74	0.87	0.84	0.91	0.95
2	0.15	0.23	0.31	0.40	0.64	0.74	0.86	0.88	0.92	0.98
3	0.24	0.27	0.31	0.41	0.45	0.57	0.64	0.74	0.77	0.82
4	0.92	0.94	0.96	0.99	1.00	0.99	1.00	1.00	1.00	1.00
5	0.97	0.98	0.97	0.99	1.00	1.00	1.00	1.00	1.00	1.00
6	0.95	0.98	0.97	0.99	0.97	0.98	1.00	1.00	1.00	1.00
7	0.37	0.45	0.58	0.62	0.66	0.76	0.82	0.79	0.91	0.88
8	0.39	0.54	0.57	0.64	0.89	0.81	0.86	0.78	0.90	0.89
9	0.47	0.48	0.64	0.58	0.73	0.76	0.78	0.91	0.90	0.91
10	0.95	0.99	1.00	1.00	1.00	1.00	1.00	1.00	1.00	1.00
11	0.98	0.98	0.99	0.99	1.00	1.00	0.99	1.00	1.00	1.00
12	0.98	1.00	1.00	1.00	1.00	1.00	1.00	1.00	1.00	1.00
13	0.03	0.07	0.14	0.35	0.48	0.65	0.76	0.83	0.95	0.99
14	0.02	0.04	0.14	0.29	0.53	0.65	0.82	0.87	0.94	0.93
15	0.11	0.14	0.38	0.36	0.50	0.58	0.67	0.79	0.81	0.88
16	1.00	1.00	1.00	1.00	1.00	1.00	1.00	1.00	1.00	1.00
17	1.00	1.00	1.00	1.00	1.00	1.00	1.00	1.00	1.00	1.00
18	0.92	0.94	0.99	0.99	1.00	1.00	1.00	1.00	1.00	1.00
19	0.23	0.50	0.63	0.75	0.91	0.97	1.00	1.00	1.00	1.00
20	0.33	0.49	0.59	0.83	0.89	0.97	1.00	0.99	0.99	1.00
21	0.33	0.39	0.52	0.77	0.79	0.88	0.96	0.97	0.98	1.00
22	0.78	0.91	0.97	0.99	1.00	1.00	1.00	1.00	1.00	1.00
23	0.77	0.86	0.97	0.98	1.00	1.00	1.00	1.00	1.00	1.00
24	0.87	0.83	0.94	0.94	0.97	1.00	1.00	1.00	1.00	1.00
25	0.28	0.37	0.66	0.81	0.90	0.96	0.98	1.00	0.99	1.00
26	0.26	0.43	0.61	0.78	0.87	0.97	0.98	0.99	1.00	1.00
27	0.45	0.62	0.52	0.62	0.63	0.67	0.69	0.74	0.86	0.85
28	0.85	0.91	0.98	0.98	1.00	1.00	1.00	1.00	1.00	1.00
29	0.89	0.94	0.98	0.99	1.00	1.00	1.00	1.00	1.00	1.00
30	0.79	0.82	0.86	0.87	0.84	0.85	0.91	0.96	0.97	0.97
31	0.05	0.07	0.25	0.37	0.65	0.78	0.88	0.98	1.00	1.00
32	0.04	0.10	0.24	0.33	0.57	0.80	0.93	0.98	1.00	1.00
33	0.09	0.26	0.22	0.39	0.56	0.61	0.77	0.90	0.95	1.00
34	1.00	1.00	1.00	1.00	1.00	1.00	1.00	1.00	1.00	1.00
35	1.00	1.00	1.00	1.00	1.00	1.00	1.00	1.00	1.00	1.00
36	0.94	0.98	1.00	1.00	1.00	1.00	1.00	1.00	1.00	1.00
37	0.03	0.05	0.19	0.48	0.65	0.87	0.94	0.98	0.98	1.00
38	0.04	0.12	0.31	0.47	0.66	0.80	0.97	0.98	1.00	1.00
39	0.05	0.14	0.24	0.44	0.63	0.80	0.86	0.99	0.99	1.00
40	0.14	0.26	0.43	0.75	0.81	0.90	0.98	0.99	1.00	1.00
41	0.22	0.27	0.38	0.72	0.90	0.95	0.97	0.99	1.00	1.00
42	0.13	0.35	0.43	0.74	0.84	0.93	0.97	0.99	1.00	1.00

Table 12: POD according to each scenario and each k2 value, k1 = 10, alpha = 0.01

Overall performances of CUSUM GLM Rossi algorithm ($\alpha = 0.05$)

	FPR k1=0	FPR k1=2	FPR k1=3	FPR k1=5	FPR k1=10
1	0.02	0.02	0.02	0.02	0.02
2	0.02	0.02	0.02	0.02	0.02
3	0.07	0.07	0.07	0.06	0.07
4	0.80	0.78	0.78	0.77	0.73
5	0.89	0.88	0.88	0.86	0.83
6	0.96	0.95	0.95	0.94	0.91
7	0.05	0.04	0.04	0.04	0.03
8	0.04	0.03	0.03	0.03	0.03
9	0.05	0.04	0.04	0.04	0.04
10	0.89	0.88	0.88	0.87	0.84
11	0.92	0.91	0.91	0.91	0.89
12	0.96	0.96	0.96	0.95	0.93
13	0.00	0.00	0.00	0.00	0.00
14	0.00	0.00	0.00	0.00	0.00
15	0.07	0.06	0.06	0.06	0.05
16	1.00	1.00	1.00	1.00	1.00
17	1.00	1.00	1.00	1.00	1.00
18	0.88	0.88	0.88	0.87	0.88
19	0.15	0.14	0.14	0.13	0.12
20	0.15	0.15	0.14	0.14	0.12
21	0.15	0.14	0.14	0.14	0.13
22	0.60	0.58	0.57	0.56	0.52
23	0.65	0.64	0.63	0.61	0.56
24	0.71	0.69	0.67	0.66	0.61
25	0.11	0.10	0.10	0.10	0.09
26	0.09	0.09	0.09	0.09	0.08
27	0.44	0.44	0.43	0.43	0.42
28	0.79	0.78	0.77	0.76	0.72
29	0.78	0.77	0.76	0.75	0.71
30	0.74	0.74	0.74	0.73	0.73
31	0.01	0.01	0.01	0.01	0.01
32	0.01	0.01	0.01	0.00	0.00
33	0.04	0.04	0.04	0.04	0.03
34	0.98	0.98	0.98	0.98	0.98
35	0.99	0.99	0.98	0.98	0.98
36	0.92	0.92	0.92	0.91	0.91
37	0.01	0.01	0.01	0.01	0.01
38	0.01	0.01	0.01	0.00	0.00
39	0.02	0.01	0.01	0.01	0.01
40	0.05	0.05	0.04	0.05	0.04
41	0.05	0.05	0.05	0.04	0.04
42	0.10	0.09	0.09	0.09	0.08

Table 13: FPR according to each scenario and each k1 value, alpha = 0.05

	k2=1	k2=2	k2=3	k2=4	k2=5	k2=6	k2=7	k2=8	k2=9	k2=10
1	0.19	0.28	0.27	0.40	0.63	0.69	0.84	0.94	0.98	0.97
2	0.16	0.26	0.31	0.59	0.63	0.66	0.85	0.94	0.94	0.99
3	0.26	0.29	0.32	0.40	0.46	0.61	0.65	0.80	0.85	0.86
4	0.91	0.96	0.99	1.00	1.00	1.00	1.00	1.00	1.00	1.00
5	0.95	0.98	0.99	1.00	1.00	1.00	1.00	1.00	1.00	1.00
6	0.99	0.99	1.00	1.00	1.00	1.00	1.00	1.00	1.00	1.00
7	0.61	0.70	0.72	0.79	0.84	0.89	0.83	0.91	0.89	0.95
8	0.58	0.65	0.68	0.85	0.79	0.88	0.91	0.87	0.95	0.95
9	0.58	0.68	0.58	0.71	0.78	0.86	0.87	0.91	0.94	0.94
10	0.97	0.98	1.00	1.00	1.00	1.00	1.00	1.00	1.00	1.00
11	0.98	1.00	1.00	1.00	1.00	1.00	1.00	1.00	1.00	1.00
12	0.98	1.00	1.00	1.00	1.00	1.00	1.00	1.00	1.00	1.00
13	0.02	0.08	0.14	0.33	0.51	0.66	0.76	0.85	0.95	0.96
14	0.06	0.08	0.20	0.31	0.50	0.73	0.78	0.86	0.94	0.98
15	0.21	0.27	0.29	0.39	0.48	0.56	0.67	0.80	0.88	0.95
16	1.00	1.00	1.00	1.00	1.00	1.00	1.00	1.00	1.00	1.00
17	1.00	1.00	1.00	1.00	1.00	1.00	1.00	1.00	1.00	1.00
18	0.95	0.95	0.97	1.00	0.99	1.00	1.00	1.00	1.00	1.00
19	0.28	0.49	0.70	0.85	0.95	0.99	1.00	1.00	1.00	1.00
20	0.32	0.47	0.74	0.83	0.96	0.97	1.00	1.00	1.00	1.00
21	0.30	0.43	0.61	0.69	0.89	0.97	0.95	0.99	1.00	1.00
22	0.86	0.96	0.99	1.00	1.00	1.00	1.00	1.00	1.00	1.00
23	0.92	0.92	0.98	1.00	1.00	1.00	1.00	1.00	1.00	1.00
24	0.83	0.93	0.99	0.99	1.00	1.00	1.00	1.00	1.00	1.00
25	0.31	0.44	0.71	0.82	0.93	0.97	1.00	1.00	1.00	1.00
26	0.28	0.37	0.72	0.84	0.95	0.98	0.99	1.00	1.00	1.00
27	0.64	0.63	0.61	0.67	0.72	0.65	0.78	0.86	0.87	0.94
28	0.95	0.94	1.00	1.00	1.00	1.00	1.00	1.00	1.00	1.00
29	0.91	0.97	1.00	0.99	1.00	0.99	1.00	1.00	1.00	1.00
30	0.82	0.80	0.80	0.83	0.87	0.90	0.91	0.92	0.97	0.98
31	0.06	0.09	0.13	0.40	0.60	0.84	0.95	0.98	0.96	1.00
32	0.03	0.08	0.27	0.39	0.56	0.81	0.89	0.97	0.98	1.00
33	0.11	0.20	0.27	0.43	0.60	0.70	0.82	0.91	0.93	0.98
34	1.00	1.00	1.00	1.00	1.00	1.00	1.00	1.00	1.00	1.00
35	1.00	1.00	1.00	1.00	1.00	1.00	1.00	1.00	1.00	1.00
36	0.97	0.99	1.00	1.00	1.00	1.00	1.00	1.00	1.00	1.00
37	0.03	0.18	0.24	0.60	0.67	0.78	0.95	0.97	0.99	1.00
38	0.06	0.08	0.26	0.46	0.69	0.81	0.93	1.00	1.00	1.00
39	0.05	0.17	0.38	0.53	0.65	0.87	0.91	0.96	0.98	1.00
40	0.13	0.45	0.60	0.85	0.86	0.96	0.98	1.00	1.00	1.00
41	0.21	0.38	0.44	0.77	0.85	0.96	0.98	0.99	1.00	1.00
42	0.24	0.36	0.51	0.61	0.87	0.90	0.99	0.98	1.00	1.00

Table 14: POD according to each scenario and each k2 value, k1 = 0, alpha = 0.05

	k2=1	k2=2	k2=3	k2=4	k2=5	k2=6	k2=7	k2=8	k2=9	k2=10
1	0.11	0.23	0.37	0.41	0.67	0.67	0.81	0.94	0.88	0.93
2	0.10	0.28	0.31	0.47	0.65	0.75	0.82	0.85	0.89	0.97
3	0.22	0.27	0.31	0.52	0.49	0.67	0.76	0.69	0.77	0.91
4	0.93	0.99	0.98	1.00	1.00	1.00	1.00	1.00	1.00	1.00
5	0.96	0.99	1.00	1.00	1.00	1.00	1.00	1.00	1.00	1.00
6	0.96	1.00	0.99	1.00	1.00	1.00	1.00	1.00	1.00	0.99
7	0.49	0.57	0.65	0.75	0.72	0.78	0.89	0.83	0.92	0.91
8	0.59	0.59	0.63	0.61	0.72	0.78	0.86	0.92	0.91	0.97
9	0.44	0.57	0.70	0.73	0.78	0.84	0.84	0.85	0.92	0.95
10	0.97	0.99	1.00	1.00	1.00	1.00	1.00	1.00	1.00	1.00
11	0.98	0.99	1.00	0.99	1.00	1.00	1.00	1.00	1.00	1.00
12	1.00	0.99	1.00	1.00	1.00	1.00	1.00	1.00	1.00	1.00
13	0.02	0.05	0.16	0.34	0.56	0.58	0.86	0.91	0.92	0.97
14	0.04	0.05	0.19	0.32	0.39	0.62	0.79	0.89	0.96	0.98
15	0.17	0.19	0.36	0.42	0.55	0.62	0.67	0.79	0.75	0.89
16	1.00	1.00	1.00	1.00	1.00	1.00	1.00	1.00	1.00	1.00
17	1.00	1.00	1.00	1.00	1.00	1.00	1.00	1.00	1.00	1.00
18	0.94	0.98	0.99	0.99	1.00	1.00	1.00	1.00	1.00	1.00
19	0.34	0.48	0.66	0.87	0.96	0.98	1.00	1.00	1.00	1.00
20	0.32	0.51	0.69	0.92	0.96	0.99	1.00	1.00	1.00	1.00
21	0.30	0.37	0.62	0.64	0.87	0.93	0.98	0.98	0.99	1.00
22	0.80	0.93	0.96	1.00	1.00	1.00	1.00	1.00	1.00	1.00
23	0.84	0.87	0.99	1.00	1.00	1.00	1.00	1.00	1.00	1.00
24	0.82	0.88	0.95	0.98	1.00	0.99	1.00	1.00	1.00	1.00
25	0.25	0.47	0.55	0.84	0.96	0.96	1.00	1.00	1.00	1.00
26	0.29	0.46	0.73	0.72	0.94	1.00	1.00	1.00	1.00	1.00
27	0.53	0.60	0.70	0.69	0.76	0.74	0.79	0.81	0.90	0.91
28	0.90	0.95	0.98	1.00	1.00	1.00	1.00	1.00	1.00	1.00
29	0.93	0.98	0.99	0.98	1.00	1.00	1.00	1.00	1.00	1.00
30	0.83	0.81	0.89	0.89	0.85	0.83	0.95	0.94	0.97	0.98
31	0.08	0.13	0.22	0.40	0.63	0.83	0.91	0.97	0.98	1.00
32	0.04	0.11	0.28	0.41	0.64	0.75	0.93	0.97	0.95	0.98
33	0.06	0.21	0.36	0.47	0.58	0.76	0.80	0.87	0.97	0.97
34	1.00	1.00	1.00	1.00	1.00	1.00	1.00	1.00	1.00	1.00
35	1.00	1.00	1.00	1.00	1.00	1.00	1.00	1.00	1.00	1.00
36	0.99	0.99	1.00	1.00	1.00	1.00	1.00	1.00	1.00	1.00
37	0.06	0.14	0.25	0.52	0.77	0.78	0.90	0.98	0.99	1.00
38	0.02	0.08	0.33	0.49	0.68	0.90	0.95	0.98	0.99	1.00
39	0.05	0.15	0.32	0.44	0.65	0.84	0.93	0.98	0.98	1.00
40	0.16	0.45	0.53	0.78	0.92	0.95	0.99	1.00	0.98	1.00
41	0.20	0.23	0.59	0.72	0.82	0.97	0.99	0.98	1.00	1.00
42	0.26	0.30	0.59	0.63	0.82	0.91	0.97	0.99	1.00	1.00

Table 15: POD according to each scenario and each k2 value, k1 = 2, alpha = 0.05

	k2=1	k2=2	k2=3	k2=4	k2=5	k2=6	k2=7	k2=8	k2=9	k2=10
1	0.15	0.15	0.26	0.52	0.67	0.71	0.83	0.93	0.93	0.95
2	0.11	0.30	0.29	0.46	0.61	0.73	0.91	0.86	0.96	0.94
3	0.18	0.28	0.35	0.44	0.59	0.62	0.68	0.78	0.86	0.88
4	0.96	0.95	0.98	1.00	1.00	1.00	1.00	1.00	1.00	1.00
5	0.96	0.99	0.98	0.99	1.00	1.00	1.00	1.00	1.00	1.00
6	0.97	1.00	0.98	1.00	0.98	1.00	1.00	1.00	1.00	1.00
7	0.49	0.53	0.62	0.69	0.77	0.81	0.91	0.95	0.90	0.92
8	0.51	0.59	0.70	0.72	0.66	0.78	0.87	0.84	0.88	0.89
9	0.59	0.64	0.69	0.74	0.69	0.78	0.78	0.90	0.88	0.95
10	0.94	0.99	1.00	1.00	1.00	1.00	1.00	1.00	1.00	1.00
11	0.98	0.99	1.00	1.00	1.00	1.00	1.00	1.00	1.00	1.00
12	1.00	1.00	1.00	1.00	1.00	1.00	1.00	1.00	1.00	1.00
13	0.02	0.12	0.22	0.28	0.56	0.63	0.77	0.85	0.96	0.99
14	0.06	0.07	0.22	0.34	0.51	0.67	0.77	0.91	0.92	0.96
15	0.18	0.24	0.34	0.41	0.49	0.58	0.70	0.77	0.88	0.83
16	1.00	1.00	1.00	1.00	1.00	1.00	1.00	1.00	1.00	1.00
17	1.00	1.00	1.00	1.00	1.00	1.00	1.00	1.00	1.00	1.00
18	0.94	0.93	0.99	1.00	1.00	1.00	1.00	1.00	1.00	1.00
19	0.31	0.53	0.66	0.84	0.94	0.95	1.00	0.99	1.00	1.00
20	0.29	0.57	0.74	0.86	0.94	0.97	1.00	1.00	1.00	1.00
21	0.28	0.42	0.61	0.70	0.81	0.91	0.91	0.98	0.99	1.00
22	0.80	0.95	0.98	1.00	1.00	1.00	1.00	1.00	1.00	1.00
23	0.77	0.94	0.98	1.00	1.00	1.00	1.00	1.00	1.00	1.00
24	0.77	0.93	0.94	0.97	0.99	1.00	1.00	1.00	1.00	1.00
25	0.22	0.42	0.66	0.84	0.96	0.98	1.00	1.00	1.00	1.00
26	0.21	0.41	0.66	0.82	0.96	0.98	0.98	1.00	1.00	1.00
27	0.53	0.53	0.60	0.66	0.70	0.70	0.73	0.83	0.85	0.89
28	0.94	0.97	0.97	1.00	1.00	1.00	1.00	1.00	1.00	1.00
29	0.89	0.95	1.00	1.00	0.99	1.00	1.00	1.00	1.00	1.00
30	0.82	0.82	0.83	0.87	0.87	0.88	0.97	0.95	0.93	0.97
31	0.03	0.13	0.20	0.35	0.59	0.93	0.90	0.98	0.99	1.00
32	0.04	0.06	0.23	0.37	0.64	0.76	0.91	0.97	0.98	1.00
33	0.17	0.17	0.32	0.48	0.53	0.79	0.77	0.93	0.96	1.00
34	0.98	1.00	1.00	1.00	1.00	1.00	1.00	1.00	1.00	1.00
35	1.00	1.00	1.00	1.00	1.00	1.00	1.00	1.00	1.00	1.00
36	0.96	0.98	1.00	1.00	1.00	1.00	1.00	1.00	1.00	1.00
37	0.06	0.13	0.38	0.49	0.70	0.88	0.92	0.99	0.98	1.00
38	0.04	0.09	0.23	0.54	0.69	0.88	0.92	0.95	0.99	1.00
39	0.09	0.20	0.31	0.51	0.61	0.75	0.91	0.95	1.00	1.00
40	0.16	0.45	0.55	0.73	0.86	0.99	0.99	0.99	1.00	1.00
41	0.18	0.24	0.39	0.69	0.82	0.96	0.98	1.00	1.00	1.00
42	0.22	0.43	0.54	0.65	0.89	0.93	0.99	1.00	1.00	1.00

Table 16: POD according to each scenario and each k2 value, k1 = 3, alpha = 0.05

	k2=1	k2=2	k2=3	k2=4	k2=5	k2=6	k2=7	k2=8	k2=9	k2=10
1	0.15	0.30	0.34	0.47	0.64	0.63	0.87	0.95	0.99	0.96
2	0.15	0.20	0.40	0.53	0.53	0.73	0.83	0.88	0.93	0.97
3	0.23	0.29	0.35	0.48	0.51	0.60	0.72	0.76	0.79	0.91
4	0.88	0.94	0.97	1.00	1.00	1.00	1.00	1.00	1.00	1.00
5	0.94	0.97	1.00	1.00	1.00	0.99	1.00	1.00	1.00	1.00
6	0.98	0.99	1.00	1.00	0.99	0.99	1.00	1.00	1.00	1.00
7	0.45	0.59	0.55	0.68	0.78	0.77	0.83	0.86	0.93	0.95
8	0.57	0.58	0.70	0.70	0.75	0.72	0.80	0.90	0.89	0.92
9	0.52	0.55	0.66	0.55	0.69	0.81	0.86	0.86	0.91	0.93
10	0.99	0.98	0.99	1.00	1.00	1.00	1.00	1.00	1.00	1.00
11	0.98	0.99	0.99	1.00	1.00	1.00	1.00	1.00	1.00	1.00
12	0.99	0.99	1.00	1.00	1.00	1.00	1.00	1.00	1.00	1.00
13	0.03	0.13	0.18	0.31	0.54	0.70	0.75	0.89	0.96	0.95
14	0.01	0.09	0.21	0.32	0.54	0.64	0.80	0.92	0.97	0.95
15	0.18	0.23	0.30	0.45	0.48	0.47	0.68	0.84	0.82	0.92
16	1.00	1.00	1.00	1.00	1.00	1.00	1.00	1.00	1.00	1.00
17	1.00	1.00	1.00	1.00	1.00	1.00	1.00	1.00	1.00	1.00
18	0.93	0.96	0.97	1.00	1.00	1.00	1.00	1.00	1.00	1.00
19	0.32	0.60	0.65	0.86	0.96	0.98	0.99	0.99	1.00	1.00
20	0.31	0.43	0.72	0.88	0.95	0.99	0.99	1.00	1.00	1.00
21	0.29	0.46	0.51	0.73	0.87	0.90	0.97	0.99	1.00	1.00
22	0.80	0.92	0.99	1.00	1.00	1.00	1.00	1.00	1.00	1.00
23	0.79	0.95	0.98	1.00	1.00	0.99	1.00	1.00	1.00	1.00
24	0.88	0.90	0.91	0.96	1.00	1.00	0.99	1.00	1.00	1.00
25	0.26	0.46	0.61	0.84	0.94	0.99	0.99	1.00	1.00	1.00
26	0.20	0.43	0.64	0.81	0.95	0.97	0.99	1.00	1.00	1.00
27	0.50	0.52	0.72	0.72	0.76	0.73	0.75	0.77	0.81	0.90
28	0.90	0.98	1.00	0.99	1.00	1.00	1.00	1.00	1.00	1.00
29	0.90	0.95	0.96	1.00	0.99	1.00	1.00	1.00	1.00	1.00
30	0.80	0.83	0.85	0.84	0.88	0.92	0.93	0.95	0.98	0.99
31	0.06	0.10	0.31	0.30	0.60	0.78	0.91	0.95	1.00	1.00
32	0.05	0.16	0.23	0.47	0.59	0.79	0.92	0.96	1.00	1.00
33	0.20	0.28	0.36	0.36	0.58	0.71	0.79	0.90	0.96	0.99
34	0.99	1.00	1.00	1.00	1.00	1.00	1.00	1.00	1.00	1.00
35	0.98	1.00	1.00	1.00	1.00	1.00	1.00	1.00	1.00	1.00
36	0.99	0.98	0.98	1.00	0.99	1.00	1.00	1.00	1.00	1.00
37	0.02	0.08	0.27	0.44	0.69	0.84	0.94	0.98	0.97	1.00
38	0.01	0.10	0.20	0.39	0.75	0.89	0.94	0.99	1.00	1.00
39	0.06	0.09	0.35	0.40	0.61	0.81	0.89	0.99	0.99	1.00
40	0.19	0.28	0.59	0.69	0.79	0.92	1.00	0.99	1.00	1.00
41	0.21	0.29	0.57	0.71	0.85	0.93	1.00	1.00	1.00	1.00
42	0.24	0.32	0.43	0.68	0.87	0.91	0.96	1.00	1.00	1.00

Table 17: POD according to each scenario and each k2 value, k1 = 5, alpha = 0.05

	k2=1	k2=2	k2=3	k2=4	k2=5	k2=6	k2=7	k2=8	k2=9	k2=10
1	0.19	0.19	0.30	0.46	0.57	0.74	0.87	0.84	0.91	0.95
2	0.15	0.23	0.31	0.40	0.64	0.74	0.86	0.88	0.92	0.98
3	0.24	0.27	0.31	0.41	0.45	0.57	0.64	0.74	0.77	0.82
4	0.92	0.94	0.96	0.99	1.00	0.99	1.00	1.00	1.00	1.00
5	0.97	0.98	0.97	0.99	1.00	1.00	1.00	1.00	1.00	1.00
6	0.95	0.98	0.97	0.99	0.97	0.98	1.00	1.00	1.00	1.00
7	0.37	0.45	0.58	0.62	0.66	0.76	0.82	0.79	0.91	0.88
8	0.39	0.54	0.57	0.64	0.89	0.81	0.86	0.78	0.90	0.89
9	0.47	0.48	0.64	0.58	0.73	0.76	0.78	0.91	0.90	0.91
10	0.95	0.99	1.00	1.00	1.00	1.00	1.00	1.00	1.00	1.00
11	0.98	0.98	0.99	0.99	1.00	1.00	0.99	1.00	1.00	1.00
12	0.98	1.00	1.00	1.00	1.00	1.00	1.00	1.00	1.00	1.00
13	0.03	0.07	0.14	0.35	0.48	0.65	0.76	0.83	0.95	0.99
14	0.02	0.04	0.14	0.29	0.53	0.65	0.82	0.87	0.94	0.93
15	0.11	0.14	0.38	0.36	0.50	0.58	0.67	0.79	0.81	0.88
16	1.00	1.00	1.00	1.00	1.00	1.00	1.00	1.00	1.00	1.00
17	1.00	1.00	1.00	1.00	1.00	1.00	1.00	1.00	1.00	1.00
18	0.92	0.94	0.99	0.99	1.00	1.00	1.00	1.00	1.00	1.00
19	0.23	0.50	0.63	0.75	0.91	0.97	1.00	1.00	1.00	1.00
20	0.33	0.49	0.59	0.83	0.89	0.97	1.00	0.99	0.99	1.00
21	0.33	0.39	0.52	0.77	0.79	0.88	0.96	0.97	0.98	1.00
22	0.78	0.91	0.97	0.99	1.00	1.00	1.00	1.00	1.00	1.00
23	0.77	0.86	0.97	0.98	1.00	1.00	1.00	1.00	1.00	1.00
24	0.87	0.83	0.94	0.94	0.97	1.00	1.00	1.00	1.00	1.00
25	0.28	0.37	0.66	0.81	0.90	0.96	0.98	1.00	0.99	1.00
26	0.26	0.43	0.61	0.78	0.87	0.97	0.98	0.99	1.00	1.00
27	0.45	0.62	0.52	0.62	0.63	0.67	0.69	0.74	0.86	0.85
28	0.85	0.91	0.98	0.98	1.00	1.00	1.00	1.00	1.00	1.00
29	0.89	0.94	0.98	0.99	1.00	1.00	1.00	1.00	1.00	1.00
30	0.79	0.82	0.86	0.87	0.84	0.85	0.91	0.96	0.97	0.97
31	0.05	0.07	0.25	0.37	0.65	0.78	0.88	0.98	1.00	1.00
32	0.04	0.10	0.24	0.33	0.57	0.80	0.93	0.98	1.00	1.00
33	0.09	0.26	0.22	0.39	0.56	0.61	0.77	0.90	0.95	1.00
34	1.00	1.00	1.00	1.00	1.00	1.00	1.00	1.00	1.00	1.00
35	1.00	1.00	1.00	1.00	1.00	1.00	1.00	1.00	1.00	1.00
36	0.94	0.98	1.00	1.00	1.00	1.00	1.00	1.00	1.00	1.00
37	0.03	0.05	0.19	0.48	0.65	0.87	0.94	0.98	0.98	1.00
38	0.04	0.12	0.31	0.47	0.66	0.80	0.97	0.98	1.00	1.00
39	0.05	0.14	0.24	0.44	0.63	0.80	0.86	0.99	0.99	1.00
40	0.14	0.26	0.43	0.75	0.81	0.90	0.98	0.99	1.00	1.00
41	0.22	0.27	0.38	0.72	0.90	0.95	0.97	0.99	1.00	1.00
42	0.13	0.35	0.43	0.74	0.84	0.93	0.97	0.99	1.00	1.00

Table 18: POD according to each scenario and each k2 value, k1 = 10, alpha = 0.05
