# Supplementary material for: Evaluation and comparison of statistical methods for early temporal detection of outbreaks: A simulation-based study
Source: PLoS One. 2017 Jul 17;12(7):e0181227. doi: 10.1371/journal.pone.0181227 (PMC5513450; doi:10.1371/journal.pone.0181227)
Supplement: S11 Appendix — (PDF) [file pone.0181227.s011.pdf]

Evaluation and Comparison of Statistical Methods for Early Temporal Detection of Outbreaks: a Simulation-Based Study

Appendix S11: Overall performances of Bayes 1 algorithm ($\alpha = 0.05$)

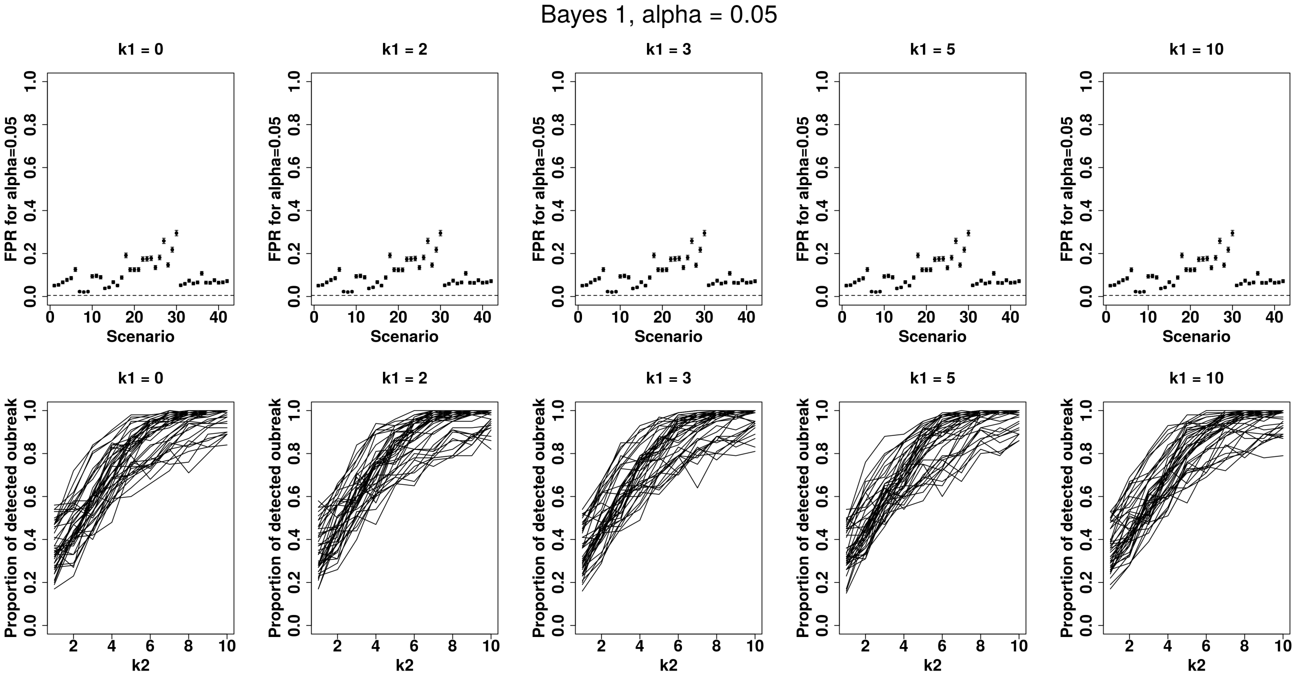

Figure 1: Bayes 1 algorithm performances for $\alpha = 0.05$ by increasing past outbreak amplitude $k_1 = 0$, 2, 3, 5 or 10 with (i) on the first row: false positive rate for 42 simulated scenarios, (ii) on the second row: probability of detection for 42 simulated scenarios (each curve corresponding to a scenario) by increasing current outbreak amplitude $k_2 = 1$ to 10.

Overall performances of Bayes 1 algorithm ($\alpha = 0.05$)

	FPR k1=0	FPR k1=2	FPR k1=3	FPR k1=5	FPR k1=10
1	0.05	0.05	0.05	0.05	0.05
2	0.05	0.05	0.05	0.05	0.05
3	0.07	0.07	0.07	0.07	0.07
4	0.08	0.08	0.08	0.08	0.08
5	0.09	0.09	0.08	0.08	0.08
6	0.13	0.13	0.13	0.13	0.12
7	0.02	0.02	0.02	0.02	0.02
8	0.02	0.02	0.02	0.02	0.02
9	0.02	0.02	0.02	0.02	0.02
10	0.09	0.09	0.09	0.09	0.09
11	0.10	0.10	0.10	0.10	0.10
12	0.09	0.09	0.09	0.09	0.09
13	0.04	0.04	0.04	0.04	0.04
14	0.04	0.04	0.04	0.04	0.04
15	0.07	0.07	0.07	0.07	0.07
16	0.05	0.05	0.05	0.05	0.05
17	0.09	0.09	0.09	0.09	0.09
18	0.19	0.19	0.19	0.19	0.19
19	0.12	0.12	0.12	0.12	0.12
20	0.12	0.12	0.12	0.12	0.12
21	0.13	0.12	0.12	0.12	0.12
22	0.17	0.17	0.17	0.17	0.17
23	0.18	0.17	0.18	0.17	0.17
24	0.18	0.18	0.18	0.18	0.18
25	0.13	0.13	0.13	0.13	0.13
26	0.18	0.18	0.18	0.18	0.18
27	0.26	0.26	0.26	0.26	0.26
28	0.15	0.15	0.15	0.15	0.15
29	0.22	0.22	0.22	0.22	0.22
30	0.29	0.29	0.29	0.29	0.29
31	0.05	0.05	0.05	0.05	0.05
32	0.06	0.06	0.06	0.06	0.06
33	0.07	0.07	0.07	0.07	0.07
34	0.06	0.06	0.06	0.06	0.06
35	0.07	0.07	0.07	0.07	0.07
36	0.11	0.11	0.11	0.11	0.11
37	0.06	0.06	0.06	0.06	0.06
38	0.06	0.06	0.06	0.06	0.06
39	0.08	0.08	0.08	0.08	0.08
40	0.06	0.06	0.06	0.06	0.06
41	0.07	0.07	0.07	0.07	0.07
42	0.07	0.07	0.07	0.07	0.07

Table 1: FPR according to each scenario and each k1 value, alpha = 0.05

	k2=1	k2=2	k2=3	k2=4	k2=5	k2=6	k2=7	k2=8	k2=9	k2=10
1	0.32	0.41	0.42	0.48	0.74	0.74	0.87	0.94	0.94	0.94
2	0.36	0.33	0.43	0.62	0.76	0.77	0.81	0.94	0.92	0.97
3	0.28	0.29	0.45	0.59	0.60	0.66	0.72	0.84	0.86	0.90
4	0.34	0.41	0.61	0.71	0.83	0.89	0.95	0.98	0.98	1.00
5	0.32	0.41	0.60	0.65	0.71	0.79	0.89	0.95	0.94	0.99
6	0.41	0.39	0.49	0.57	0.73	0.75	0.82	0.84	0.92	0.92
7	0.53	0.53	0.58	0.68	0.79	0.68	0.77	0.86	0.83	0.89
8	0.54	0.54	0.55	0.69	0.70	0.78	0.75	0.76	0.89	0.90
9	0.45	0.57	0.51	0.63	0.71	0.75	0.77	0.81	0.83	0.84
10	0.34	0.47	0.55	0.68	0.88	0.86	0.94	0.95	0.99	1.00
11	0.36	0.38	0.64	0.77	0.86	0.94	0.98	0.99	0.98	1.00
12	0.34	0.38	0.60	0.71	0.80	0.90	0.92	0.97	0.97	1.00
13	0.17	0.23	0.45	0.64	0.75	0.84	0.86	0.95	1.00	0.99
14	0.27	0.28	0.45	0.57	0.64	0.72	0.83	0.92	0.95	0.98
15	0.31	0.47	0.40	0.59	0.68	0.78	0.87	0.91	0.96	0.97
16	0.24	0.40	0.48	0.60	0.80	0.91	0.94	1.00	1.00	1.00
17	0.33	0.33	0.52	0.74	0.74	0.83	0.89	0.96	0.97	1.00
18	0.46	0.48	0.61	0.57	0.76	0.73	0.84	0.87	0.93	0.95
19	0.37	0.53	0.71	0.79	0.95	0.95	0.98	0.99	1.00	1.00
20	0.49	0.56	0.70	0.84	0.87	0.95	0.97	0.99	1.00	1.00
21	0.47	0.55	0.73	0.82	0.85	0.95	0.97	1.00	1.00	1.00
22	0.47	0.66	0.84	0.90	0.96	0.98	0.99	1.00	1.00	1.00
23	0.47	0.61	0.81	0.86	0.97	0.97	1.00	0.99	1.00	1.00
24	0.56	0.57	0.71	0.85	0.82	0.94	0.99	0.98	1.00	1.00
25	0.54	0.62	0.80	0.86	0.94	0.97	1.00	0.99	0.99	1.00
26	0.43	0.60	0.68	0.82	0.91	0.95	0.95	0.99	0.98	1.00
27	0.47	0.58	0.58	0.68	0.70	0.75	0.85	0.83	0.87	0.89
28	0.47	0.72	0.83	0.91	0.98	0.98	0.99	1.00	1.00	1.00
29	0.40	0.51	0.61	0.79	0.85	0.88	0.96	0.98	0.97	1.00
30	0.50	0.55	0.50	0.63	0.69	0.74	0.82	0.71	0.80	0.89
31	0.20	0.43	0.44	0.67	0.87	0.92	0.97	1.00	0.99	1.00
32	0.27	0.41	0.53	0.68	0.74	0.94	0.95	0.98	0.99	1.00
33	0.26	0.42	0.58	0.71	0.79	0.89	0.95	0.97	0.99	1.00
34	0.19	0.40	0.53	0.68	0.90	0.96	0.96	0.99	1.00	1.00
35	0.29	0.41	0.57	0.75	0.82	0.91	0.94	0.97	1.00	1.00
36	0.37	0.37	0.53	0.65	0.84	0.89	0.94	0.97	0.99	1.00
37	0.24	0.51	0.60	0.83	0.81	0.91	1.00	0.98	1.00	1.00
38	0.35	0.27	0.62	0.79	0.87	0.96	0.99	0.99	1.00	1.00
39	0.29	0.49	0.60	0.75	0.87	0.92	0.93	0.99	1.00	1.00
40	0.32	0.47	0.56	0.84	0.84	0.96	0.96	1.00	1.00	1.00
41	0.33	0.43	0.58	0.76	0.88	0.94	0.95	1.00	1.00	1.00
42	0.21	0.44	0.55	0.74	0.85	0.93	0.99	0.99	1.00	1.00

Table 2: POD according to each scenario and each k2 value, k1 = 0, alpha = 0.05

	k2=1	k2=2	k2=3	k2=4	k2=5	k2=6	k2=7	k2=8	k2=9	k2=10
1	0.24	0.29	0.53	0.54	0.66	0.72	0.84	0.91	0.87	0.96
2	0.33	0.44	0.44	0.61	0.69	0.80	0.79	0.90	0.89	0.92
3	0.28	0.35	0.51	0.47	0.65	0.74	0.78	0.87	0.84	0.94
4	0.29	0.44	0.55	0.76	0.81	0.86	0.95	0.99	0.98	1.00
5	0.38	0.39	0.64	0.66	0.72	0.90	0.89	0.95	0.95	0.99
6	0.21	0.51	0.54	0.61	0.66	0.65	0.80	0.82	0.88	0.92
7	0.49	0.56	0.60	0.66	0.73	0.78	0.78	0.79	0.88	0.86
8	0.58	0.50	0.60	0.64	0.69	0.67	0.77	0.78	0.84	0.95
9	0.42	0.46	0.64	0.60	0.66	0.77	0.74	0.79	0.79	0.91
10	0.28	0.51	0.60	0.75	0.77	0.94	0.95	0.96	0.98	1.00
11	0.37	0.41	0.71	0.62	0.85	0.91	0.97	0.98	1.00	1.00
12	0.28	0.56	0.55	0.72	0.86	0.94	1.00	0.97	0.98	0.96
13	0.23	0.26	0.40	0.62	0.72	0.82	0.90	0.95	0.98	0.99
14	0.17	0.40	0.51	0.61	0.66	0.77	0.90	0.95	0.99	0.98
15	0.35	0.38	0.46	0.60	0.84	0.82	0.81	0.92	0.92	0.93
16	0.30	0.31	0.48	0.64	0.85	0.94	0.97	0.97	1.00	1.00
17	0.28	0.34	0.60	0.74	0.79	0.87	0.94	0.97	0.98	0.99
18	0.41	0.47	0.69	0.57	0.77	0.82	0.81	0.86	0.86	0.96
19	0.46	0.56	0.65	0.89	0.91	0.95	0.98	0.99	0.99	1.00
20	0.39	0.56	0.71	0.84	0.90	0.91	0.97	0.97	1.00	1.00
21	0.43	0.45	0.70	0.78	0.91	0.96	0.95	1.00	0.99	1.00
22	0.46	0.66	0.77	0.94	0.93	0.95	1.00	1.00	1.00	1.00
23	0.53	0.61	0.84	0.89	0.93	0.98	1.00	1.00	1.00	1.00
24	0.55	0.64	0.80	0.91	0.91	0.94	0.97	1.00	1.00	1.00
25	0.45	0.59	0.77	0.91	0.95	0.96	0.99	0.99	1.00	1.00
26	0.41	0.63	0.66	0.77	0.87	0.91	0.97	0.96	1.00	1.00
27	0.45	0.46	0.63	0.74	0.76	0.82	0.82	0.91	0.89	0.88
28	0.49	0.65	0.74	0.91	0.96	1.00	1.00	1.00	1.00	1.00
29	0.55	0.56	0.67	0.73	0.84	0.89	0.96	0.95	0.99	1.00
30	0.51	0.59	0.58	0.70	0.70	0.71	0.78	0.79	0.89	0.82
31	0.25	0.35	0.50	0.70	0.84	0.91	0.96	0.99	1.00	0.99
32	0.25	0.38	0.65	0.69	0.87	0.90	0.97	0.98	0.99	0.99
33	0.27	0.44	0.55	0.75	0.86	0.86	0.92	0.98	1.00	1.00
34	0.28	0.46	0.60	0.77	0.81	0.90	0.97	1.00	0.99	1.00
35	0.22	0.46	0.51	0.65	0.87	0.91	0.99	0.99	1.00	1.00
36	0.37	0.44	0.56	0.76	0.75	0.90	0.96	0.97	0.99	0.99
37	0.27	0.38	0.65	0.77	0.90	0.90	0.98	1.00	1.00	1.00
38	0.17	0.43	0.57	0.68	0.90	0.95	0.99	1.00	1.00	0.99
39	0.22	0.47	0.60	0.81	0.82	0.94	0.99	1.00	0.98	1.00
40	0.33	0.58	0.57	0.77	0.93	0.98	0.99	0.99	1.00	1.00
41	0.34	0.31	0.63	0.75	0.82	0.97	1.00	0.99	1.00	1.00
42	0.28	0.38	0.58	0.75	0.80	0.96	0.97	0.99	0.99	0.99

Table 3: POD according to each scenario and each k2 value, k1 = 2, alpha = 0.05

	k2=1	k2=2	k2=3	k2=4	k2=5	k2=6	k2=7	k2=8	k2=9	k2=10
1	0.27	0.34	0.48	0.61	0.78	0.71	0.86	0.93	0.91	0.95
2	0.27	0.45	0.49	0.54	0.66	0.78	0.87	0.85	0.89	0.93
3	0.29	0.43	0.44	0.49	0.71	0.70	0.81	0.86	0.86	0.94
4	0.24	0.45	0.53	0.68	0.78	0.93	0.93	0.97	0.99	1.00
5	0.36	0.43	0.49	0.77	0.77	0.81	0.90	0.98	0.97	1.00
6	0.29	0.46	0.46	0.62	0.61	0.71	0.82	0.80	0.87	0.95
7	0.41	0.46	0.60	0.60	0.70	0.79	0.78	0.88	0.83	0.87
8	0.51	0.62	0.65	0.65	0.64	0.71	0.83	0.77	0.85	0.89
9	0.52	0.57	0.52	0.64	0.70	0.79	0.64	0.81	0.86	0.83
10	0.37	0.47	0.64	0.77	0.83	0.97	0.97	0.99	0.98	1.00
11	0.32	0.45	0.55	0.77	0.88	0.86	0.95	0.98	0.99	0.99
12	0.41	0.40	0.63	0.63	0.88	0.85	0.91	0.99	0.97	0.99
13	0.23	0.32	0.45	0.56	0.70	0.78	0.90	0.95	0.96	1.00
14	0.21	0.30	0.48	0.57	0.73	0.79	0.86	0.90	0.97	0.95
15	0.19	0.33	0.60	0.64	0.65	0.74	0.82	0.86	0.98	0.94
16	0.16	0.29	0.56	0.76	0.77	0.89	0.94	0.97	0.99	1.00
17	0.24	0.46	0.53	0.70	0.81	0.84	0.92	0.95	0.97	1.00
18	0.38	0.44	0.58	0.67	0.70	0.83	0.79	0.88	0.85	0.92
19	0.43	0.66	0.77	0.87	0.93	0.92	0.99	0.99	1.00	1.00
20	0.47	0.52	0.73	0.84	0.91	0.94	0.97	1.00	1.00	1.00
21	0.47	0.61	0.75	0.77	0.93	0.96	0.98	0.98	1.00	1.00
22	0.54	0.67	0.82	0.93	0.96	0.99	1.00	1.00	1.00	1.00
23	0.44	0.64	0.85	0.85	0.97	0.95	0.99	1.00	1.00	1.00
24	0.50	0.64	0.74	0.85	0.95	0.95	0.97	0.96	1.00	1.00
25	0.36	0.62	0.77	0.91	0.94	0.99	1.00	1.00	0.99	1.00
26	0.48	0.58	0.73	0.72	0.85	0.94	0.95	0.97	1.00	1.00
27	0.52	0.48	0.60	0.66	0.71	0.79	0.75	0.88	0.88	0.93
28	0.45	0.67	0.83	0.90	0.96	0.99	1.00	1.00	1.00	1.00
29	0.45	0.54	0.79	0.76	0.86	0.92	0.90	0.96	1.00	1.00
30	0.47	0.62	0.61	0.66	0.63	0.73	0.79	0.80	0.79	0.81
31	0.29	0.39	0.53	0.72	0.79	0.96	0.96	1.00	0.98	1.00
32	0.20	0.48	0.50	0.77	0.80	0.89	0.94	0.99	1.00	1.00
33	0.31	0.43	0.58	0.65	0.73	0.90	0.93	0.98	0.97	0.99
34	0.24	0.40	0.56	0.75	0.84	0.95	0.96	0.99	1.00	1.00
35	0.30	0.46	0.65	0.69	0.87	0.91	0.98	0.99	0.98	1.00
36	0.36	0.48	0.63	0.74	0.84	0.88	0.93	0.98	1.00	0.99
37	0.26	0.43	0.65	0.75	0.86	0.98	0.99	0.98	1.00	1.00
38	0.30	0.45	0.55	0.77	0.81	0.96	0.99	0.98	1.00	1.00
39	0.27	0.41	0.65	0.78	0.84	0.94	0.95	0.98	1.00	1.00
40	0.28	0.53	0.57	0.79	0.85	0.93	0.98	0.99	1.00	1.00
41	0.23	0.41	0.63	0.79	0.81	0.92	0.96	1.00	1.00	1.00
42	0.29	0.52	0.59	0.74	0.88	0.98	0.97	0.98	1.00	1.00

Table 4: POD according to each scenario and each k2 value, k1 = 3, alpha = 0.05

	k2=1	k2=2	k2=3	k2=4	k2=5	k2=6	k2=7	k2=8	k2=9	k2=10
1	0.30	0.36	0.50	0.65	0.65	0.73	0.91	0.97	0.95	0.97
2	0.28	0.36	0.49	0.62	0.68	0.79	0.79	0.92	0.90	0.95
3	0.36	0.41	0.52	0.61	0.66	0.65	0.79	0.84	0.88	0.91
4	0.36	0.40	0.58	0.69	0.79	0.90	0.94	0.97	1.00	1.00
5	0.31	0.45	0.58	0.60	0.79	0.86	0.87	0.95	0.97	1.00
6	0.32	0.33	0.52	0.55	0.58	0.72	0.81	0.87	0.89	0.94
7	0.46	0.62	0.48	0.75	0.75	0.60	0.74	0.86	0.92	0.89
8	0.50	0.43	0.62	0.60	0.62	0.73	0.67	0.80	0.81	0.86
9	0.45	0.46	0.64	0.54	0.66	0.77	0.71	0.86	0.80	0.86
10	0.23	0.45	0.61	0.69	0.84	0.94	0.97	0.94	1.00	1.00
11	0.32	0.41	0.57	0.76	0.92	0.90	0.96	0.98	1.00	0.98
12	0.31	0.54	0.62	0.71	0.84	0.95	0.95	0.99	0.99	0.98
13	0.26	0.34	0.46	0.61	0.69	0.83	0.90	0.94	0.99	0.99
14	0.15	0.34	0.53	0.60	0.75	0.72	0.82	0.98	0.99	0.99
15	0.29	0.33	0.57	0.56	0.70	0.78	0.81	0.92	0.94	0.98
16	0.26	0.31	0.54	0.71	0.87	0.90	0.88	0.99	1.00	1.00
17	0.34	0.45	0.65	0.67	0.79	0.86	0.91	0.97	1.00	1.00
18	0.45	0.50	0.47	0.58	0.68	0.74	0.88	0.91	0.88	0.92
19	0.33	0.67	0.69	0.86	0.91	0.98	0.98	0.99	0.99	1.00
20	0.50	0.54	0.68	0.79	0.91	0.93	0.97	0.99	1.00	1.00
21	0.42	0.42	0.62	0.82	0.89	0.95	0.99	1.00	1.00	1.00
22	0.48	0.76	0.88	0.89	0.95	0.98	0.99	1.00	1.00	1.00
23	0.45	0.64	0.79	0.86	0.93	0.97	0.99	1.00	1.00	1.00
24	0.47	0.62	0.71	0.85	0.95	0.98	1.00	0.99	1.00	1.00
25	0.43	0.62	0.76	0.86	0.94	0.97	0.99	1.00	1.00	1.00
26	0.46	0.58	0.74	0.80	0.88	0.92	0.94	1.00	1.00	1.00
27	0.44	0.51	0.59	0.64	0.77	0.77	0.80	0.91	0.85	0.89
28	0.49	0.68	0.80	0.85	0.90	0.98	0.99	1.00	1.00	1.00
29	0.54	0.55	0.68	0.73	0.85	0.91	0.97	0.98	1.00	0.99
30	0.41	0.58	0.64	0.59	0.69	0.67	0.73	0.82	0.79	0.89
31	0.26	0.39	0.49	0.68	0.78	0.90	0.92	0.98	1.00	1.00
32	0.31	0.40	0.55	0.64	0.83	0.88	0.97	0.98	1.00	1.00
33	0.29	0.47	0.56	0.65	0.89	0.88	0.95	1.00	0.99	0.99
34	0.16	0.42	0.57	0.71	0.92	0.88	0.97	0.99	1.00	1.00
35	0.28	0.44	0.62	0.65	0.81	0.96	0.98	0.98	1.00	1.00
36	0.32	0.46	0.66	0.69	0.82	0.85	0.94	0.97	1.00	1.00
37	0.29	0.37	0.62	0.73	0.87	0.95	0.98	1.00	0.98	1.00
38	0.17	0.45	0.56	0.69	0.92	0.99	0.99	0.99	1.00	1.00
39	0.32	0.31	0.66	0.72	0.83	0.98	0.97	0.99	1.00	1.00
40	0.29	0.47	0.60	0.77	0.86	0.93	0.97	0.99	1.00	1.00
41	0.28	0.40	0.61	0.75	0.85	0.94	0.97	0.99	1.00	1.00
42	0.30	0.49	0.57	0.80	0.88	0.98	0.98	1.00	1.00	1.00

Table 5: POD according to each scenario and each k2 value, k1 = 5, alpha = 0.05

	k2=1	k2=2	k2=3	k2=4	k2=5	k2=6	k2=7	k2=8	k2=9	k2=10
1	0.38	0.39	0.43	0.61	0.71	0.74	0.93	0.87	0.87	0.95
2	0.30	0.34	0.54	0.59	0.72	0.82	0.83	0.90	0.89	0.93
3	0.39	0.41	0.49	0.61	0.57	0.74	0.77	0.90	0.85	0.92
4	0.32	0.43	0.52	0.70	0.80	0.91	0.93	0.98	0.98	0.99
5	0.27	0.44	0.45	0.54	0.78	0.88	0.92	0.90	0.97	0.96
6	0.31	0.42	0.54	0.64	0.64	0.71	0.84	0.81	0.89	0.88
7	0.49	0.45	0.56	0.62	0.72	0.74	0.72	0.80	0.89	0.87
8	0.45	0.54	0.57	0.60	0.76	0.71	0.84	0.78	0.86	0.88
9	0.53	0.44	0.61	0.59	0.73	0.73	0.78	0.80	0.85	0.89
10	0.35	0.44	0.68	0.70	0.82	0.90	0.95	0.97	0.99	0.99
11	0.29	0.39	0.58	0.70	0.80	0.86	0.94	0.96	0.97	0.99
12	0.26	0.38	0.61	0.68	0.84	0.86	0.92	0.98	1.00	0.99
13	0.17	0.28	0.45	0.55	0.75	0.81	0.89	0.97	0.95	1.00
14	0.26	0.29	0.39	0.54	0.76	0.82	0.88	0.92	0.97	0.97
15	0.19	0.28	0.48	0.51	0.67	0.83	0.90	0.90	0.93	0.94
16	0.22	0.33	0.52	0.65	0.80	0.87	0.93	0.99	0.99	1.00
17	0.32	0.41	0.53	0.62	0.80	0.91	0.94	0.94	0.98	0.99
18	0.36	0.50	0.50	0.63	0.71	0.73	0.88	0.94	0.91	0.92
19	0.39	0.67	0.71	0.80	0.94	0.97	0.98	1.00	1.00	0.99
20	0.48	0.57	0.71	0.82	0.90	0.93	0.98	1.00	1.00	1.00
21	0.49	0.66	0.68	0.79	0.91	0.95	0.98	1.00	0.98	1.00
22	0.50	0.69	0.80	0.91	0.95	1.00	1.00	1.00	0.99	1.00
23	0.53	0.64	0.81	0.93	0.94	0.99	0.99	0.99	1.00	1.00
24	0.38	0.61	0.70	0.81	0.95	0.92	0.96	0.99	1.00	1.00
25	0.50	0.61	0.78	0.89	0.93	0.97	0.98	1.00	1.00	1.00
26	0.48	0.53	0.67	0.75	0.83	0.88	0.98	0.98	0.99	1.00
27	0.40	0.52	0.48	0.73	0.66	0.76	0.80	0.81	0.89	0.89
28	0.50	0.65	0.79	0.87	0.98	0.98	0.99	1.00	1.00	1.00
29	0.53	0.55	0.67	0.84	0.89	0.93	0.94	0.99	0.98	1.00
30	0.45	0.60	0.58	0.60	0.64	0.69	0.77	0.81	0.78	0.79
31	0.29	0.37	0.62	0.71	0.87	0.93	0.93	0.99	1.00	1.00
32	0.24	0.44	0.53	0.64	0.79	0.91	0.98	0.98	0.99	1.00
33	0.29	0.51	0.47	0.71	0.78	0.89	0.92	0.98	0.97	1.00
34	0.28	0.32	0.57	0.69	0.87	0.90	0.98	1.00	1.00	1.00
35	0.30	0.41	0.55	0.72	0.83	0.92	0.96	0.98	1.00	1.00
36	0.34	0.42	0.58	0.67	0.79	0.87	0.92	0.97	0.98	0.99
37	0.25	0.41	0.63	0.72	0.94	0.94	0.98	0.99	1.00	1.00
38	0.27	0.33	0.63	0.71	0.83	0.92	0.99	0.99	1.00	1.00
39	0.25	0.53	0.51	0.75	0.88	0.92	0.97	1.00	1.00	1.00
40	0.25	0.37	0.60	0.72	0.92	0.93	0.98	1.00	0.99	1.00
41	0.37	0.48	0.52	0.75	0.91	0.92	1.00	1.00	0.99	1.00
42	0.36	0.40	0.50	0.78	0.85	0.93	0.97	0.99	1.00	0.99

Table 6: POD according to each scenario and each k2 value, k1 = 10, alpha = 0.05
