# Supplementary material for: Evaluation and comparison of statistical methods for early temporal detection of outbreaks: A simulation-based study
Source: PLoS One. 2017 Jul 17;12(7):e0181227. doi: 10.1371/journal.pone.0181227 (PMC5513450; doi:10.1371/journal.pone.0181227)
Supplement: S12 Appendix — (PDF) [file pone.0181227.s012.pdf]

Evaluation and Comparison of Statistical Methods for Early Temporal Detection of Outbreaks: a Simulation-Based Study

Appendix S12: Overall performances of Bayes 2 algorithm ($\alpha = 0.05$)

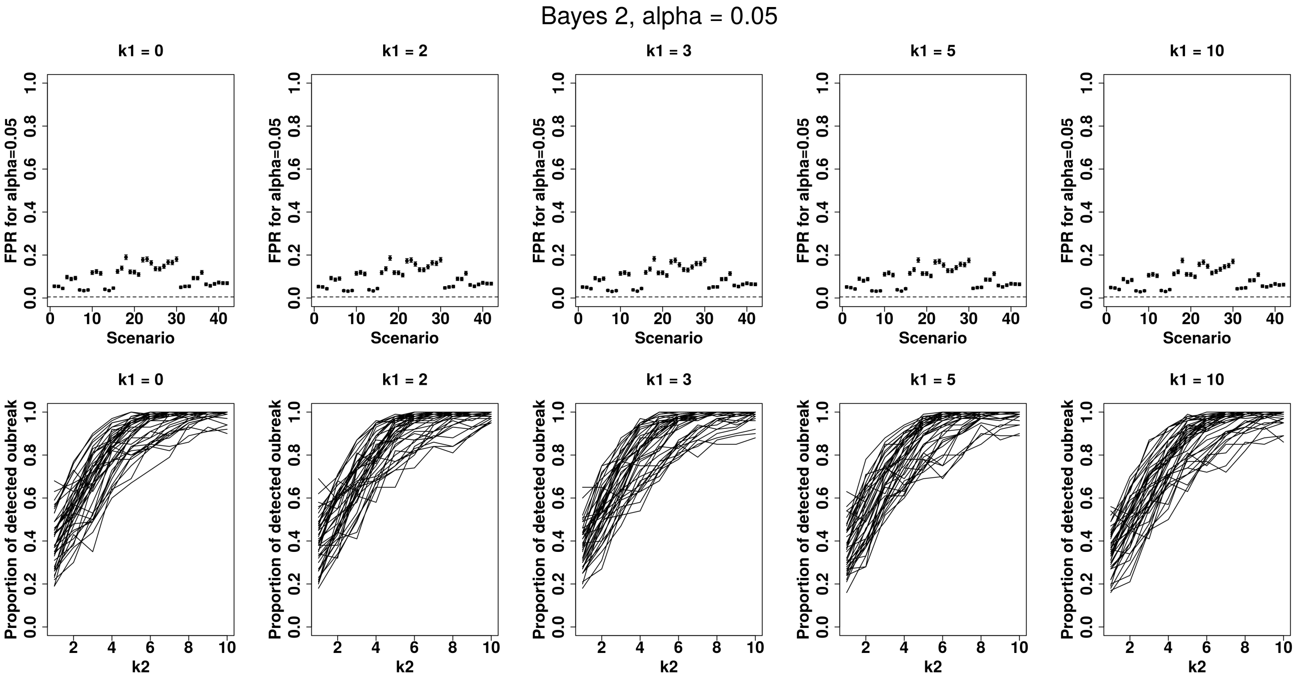

Figure 1: Bayes 2 algorithm performances for $\alpha = 0.05$ by increasing past outbreak amplitude $k_1 = 0$, 2, 3, 5 or 10 with (i) on the first row: false positive rate for 42 simulated scenarios, (ii) on the second row: probability of detection for 42 simulated scenarios (each curve corresponding to a scenario) by increasing current outbreak amplitude $k_2 = 1$ to 10.

Overall performances of Bayes 2 algorithm ($\alpha = 0.05$)

	FPR k1=0	FPR k1=2	FPR k1=3	FPR k1=5	FPR k1=10
1	0.05	0.05	0.05	0.05	0.05
2	0.05	0.05	0.05	0.05	0.05
3	0.04	0.04	0.04	0.04	0.04
4	0.10	0.09	0.09	0.09	0.09
5	0.09	0.09	0.08	0.08	0.07
6	0.09	0.09	0.09	0.09	0.08
7	0.04	0.03	0.04	0.03	0.03
8	0.03	0.03	0.03	0.03	0.03
9	0.04	0.03	0.03	0.03	0.03
10	0.12	0.11	0.11	0.11	0.11
11	0.12	0.12	0.12	0.12	0.11
12	0.11	0.11	0.11	0.11	0.10
13	0.04	0.04	0.04	0.04	0.03
14	0.03	0.03	0.03	0.03	0.03
15	0.05	0.04	0.04	0.04	0.04
16	0.12	0.12	0.12	0.11	0.11
17	0.14	0.14	0.13	0.13	0.12
18	0.19	0.19	0.18	0.18	0.17
19	0.12	0.12	0.12	0.11	0.11
20	0.12	0.12	0.12	0.11	0.11
21	0.11	0.11	0.10	0.10	0.10
22	0.18	0.17	0.17	0.17	0.16
23	0.18	0.18	0.18	0.17	0.17
24	0.16	0.16	0.15	0.15	0.15
25	0.14	0.13	0.13	0.13	0.12
26	0.14	0.13	0.13	0.13	0.12
27	0.15	0.15	0.14	0.14	0.13
28	0.17	0.16	0.16	0.16	0.15
29	0.17	0.16	0.16	0.16	0.15
30	0.18	0.18	0.18	0.17	0.17
31	0.05	0.05	0.05	0.04	0.04
32	0.05	0.05	0.05	0.05	0.05
33	0.05	0.05	0.05	0.05	0.05
34	0.09	0.09	0.09	0.09	0.08
35	0.09	0.09	0.09	0.08	0.08
36	0.12	0.12	0.11	0.11	0.11
37	0.06	0.06	0.06	0.06	0.06
38	0.06	0.05	0.05	0.05	0.05
39	0.07	0.06	0.06	0.06	0.06
40	0.07	0.07	0.07	0.07	0.07
41	0.07	0.07	0.06	0.06	0.06
42	0.07	0.07	0.06	0.06	0.06

Table 1: FPR according to each scenario and each k1 value, alpha = 0.05

	k2=1	k2=2	k2=3	k2=4	k2=5	k2=6	k2=7	k2=8	k2=9	k2=10
1	0.37	0.45	0.49	0.63	0.79	0.86	0.91	0.96	0.98	0.97
2	0.35	0.41	0.52	0.82	0.80	0.83	0.89	0.95	0.97	0.99
3	0.26	0.37	0.44	0.60	0.67	0.73	0.79	0.94	0.97	0.94
4	0.35	0.53	0.74	0.84	0.89	0.96	0.96	1.00	1.00	1.00
5	0.44	0.51	0.70	0.86	0.85	0.91	0.95	0.99	0.99	1.00
6	0.34	0.44	0.50	0.69	0.84	0.88	0.93	0.96	1.00	0.99
7	0.63	0.67	0.72	0.77	0.82	0.83	0.90	0.93	0.91	0.94
8	0.68	0.63	0.67	0.84	0.80	0.85	0.84	0.86	0.93	0.90
9	0.59	0.73	0.63	0.78	0.78	0.84	0.82	0.89	0.91	0.92
10	0.45	0.57	0.71	0.86	0.93	0.93	0.96	0.98	1.00	1.00
11	0.46	0.55	0.80	0.91	0.95	0.97	1.00	0.99	0.97	1.00
12	0.43	0.52	0.76	0.83	0.94	0.98	1.00	1.00	1.00	1.00
13	0.19	0.34	0.49	0.73	0.83	0.91	0.89	0.97	1.00	1.00
14	0.22	0.30	0.53	0.64	0.69	0.86	0.88	0.95	0.97	1.00
15	0.24	0.43	0.35	0.65	0.75	0.83	0.91	0.96	0.98	1.00
16	0.42	0.68	0.81	0.87	0.95	0.99	0.99	1.00	1.00	1.00
17	0.49	0.58	0.74	0.89	0.95	0.99	1.00	1.00	0.99	1.00
18	0.57	0.65	0.75	0.77	0.88	0.88	0.99	0.97	1.00	1.00
19	0.37	0.58	0.83	0.87	0.98	0.99	0.99	1.00	1.00	1.00
20	0.45	0.59	0.74	0.90	0.96	0.99	1.00	1.00	1.00	1.00
21	0.34	0.53	0.70	0.86	0.95	1.00	0.98	1.00	1.00	1.00
22	0.53	0.77	0.89	0.96	1.00	0.99	1.00	1.00	1.00	1.00
23	0.55	0.72	0.87	0.95	0.97	1.00	1.00	1.00	1.00	1.00
24	0.49	0.63	0.81	0.95	0.97	1.00	1.00	1.00	1.00	1.00
25	0.56	0.72	0.86	0.93	0.95	0.99	1.00	1.00	1.00	1.00
26	0.45	0.58	0.81	0.86	0.96	0.98	1.00	1.00	1.00	1.00
27	0.37	0.57	0.53	0.71	0.84	0.85	0.94	0.97	0.98	1.00
28	0.53	0.76	0.90	0.97	1.00	0.98	1.00	1.00	1.00	1.00
29	0.45	0.61	0.84	0.93	0.97	0.97	0.98	1.00	1.00	1.00
30	0.38	0.48	0.49	0.66	0.79	0.85	0.91	0.92	0.99	1.00
31	0.19	0.53	0.50	0.71	0.90	0.98	1.00	1.00	0.99	1.00
32	0.26	0.47	0.67	0.76	0.88	0.97	0.99	0.99	0.99	1.00
33	0.27	0.39	0.58	0.78	0.90	0.96	0.96	0.99	1.00	1.00
34	0.37	0.61	0.74	0.87	0.98	1.00	1.00	1.00	1.00	1.00
35	0.43	0.63	0.77	0.88	0.94	0.99	0.99	1.00	1.00	1.00
36	0.43	0.53	0.72	0.85	0.96	0.98	0.99	1.00	1.00	1.00
37	0.23	0.53	0.65	0.83	0.92	0.98	0.98	0.99	1.00	1.00
38	0.31	0.38	0.67	0.82	0.89	0.96	1.00	1.00	1.00	1.00
39	0.27	0.56	0.70	0.83	0.96	0.98	1.00	0.99	1.00	1.00
40	0.33	0.57	0.67	0.92	0.91	0.96	0.98	1.00	1.00	1.00
41	0.38	0.50	0.64	0.85	0.95	0.97	0.98	1.00	1.00	1.00
42	0.28	0.50	0.66	0.76	0.88	0.97	1.00	1.00	1.00	1.00

Table 2: POD according to each scenario and each k2 value, k1 = 0, alpha = 0.05

	k2=1	k2=2	k2=3	k2=4	k2=5	k2=6	k2=7	k2=8	k2=9	k2=10
1	0.33	0.45	0.61	0.58	0.82	0.81	0.91	0.96	0.93	0.98
2	0.38	0.44	0.41	0.68	0.85	0.90	0.88	0.93	0.92	0.97
3	0.22	0.37	0.51	0.65	0.65	0.84	0.89	0.88	0.93	0.95
4	0.39	0.61	0.69	0.83	0.93	0.96	0.96	1.00	0.99	1.00
5	0.40	0.51	0.67	0.80	0.89	0.95	0.95	1.00	0.98	0.99
6	0.21	0.51	0.61	0.69	0.75	0.87	0.91	0.94	0.98	1.00
7	0.56	0.60	0.68	0.70	0.77	0.82	0.84	0.84	0.90	0.95
8	0.69	0.59	0.67	0.68	0.73	0.74	0.84	0.81	0.90	0.96
9	0.58	0.55	0.65	0.68	0.75	0.83	0.86	0.84	0.89	0.96
10	0.45	0.61	0.77	0.87	0.86	0.97	0.99	0.98	1.00	1.00
11	0.47	0.55	0.81	0.76	0.96	0.96	0.99	1.00	1.00	1.00
12	0.35	0.63	0.73	0.83	0.95	0.99	0.99	0.99	1.00	1.00
13	0.23	0.34	0.49	0.71	0.80	0.86	0.95	0.97	0.99	0.99
14	0.18	0.33	0.50	0.67	0.73	0.80	0.94	0.98	0.99	0.98
15	0.33	0.41	0.44	0.64	0.88	0.87	0.88	0.97	0.96	1.00
16	0.49	0.62	0.75	0.89	0.98	0.98	1.00	1.00	1.00	1.00
17	0.45	0.52	0.80	0.91	0.96	1.00	1.00	0.99	1.00	1.00
18	0.46	0.60	0.74	0.77	0.87	0.92	0.94	0.99	0.98	1.00
19	0.49	0.59	0.74	0.93	0.97	1.00	1.00	1.00	1.00	1.00
20	0.43	0.59	0.75	0.95	0.96	0.99	1.00	0.98	1.00	1.00
21	0.36	0.44	0.73	0.80	0.97	0.97	0.99	0.99	1.00	1.00
22	0.48	0.71	0.87	0.96	0.95	1.00	1.00	1.00	1.00	1.00
23	0.53	0.68	0.87	0.94	0.98	1.00	1.00	1.00	1.00	1.00
24	0.62	0.70	0.85	0.94	0.98	0.99	1.00	1.00	1.00	1.00
25	0.51	0.62	0.75	0.95	0.99	0.98	1.00	1.00	1.00	1.00
26	0.45	0.63	0.77	0.82	0.95	0.99	1.00	1.00	1.00	1.00
27	0.38	0.38	0.63	0.73	0.84	0.91	0.91	0.98	0.98	0.98
28	0.47	0.71	0.82	0.95	0.98	1.00	1.00	1.00	1.00	1.00
29	0.55	0.66	0.74	0.89	0.97	0.97	1.00	1.00	1.00	1.00
30	0.39	0.52	0.58	0.79	0.77	0.81	0.88	0.94	0.97	0.97
31	0.27	0.40	0.53	0.78	0.91	0.97	0.97	1.00	1.00	1.00
32	0.26	0.40	0.64	0.75	0.90	0.95	0.98	0.99	0.99	0.99
33	0.26	0.47	0.62	0.80	0.91	0.92	0.97	0.99	1.00	1.00
34	0.42	0.62	0.75	0.89	0.93	0.98	0.99	1.00	1.00	1.00
35	0.38	0.60	0.67	0.80	0.97	0.98	0.99	1.00	1.00	1.00
36	0.41	0.48	0.74	0.84	0.93	0.95	1.00	0.99	1.00	1.00
37	0.30	0.43	0.67	0.78	0.94	0.97	0.98	1.00	1.00	1.00
38	0.21	0.46	0.65	0.79	0.93	0.99	0.99	1.00	1.00	1.00
39	0.20	0.47	0.61	0.85	0.87	0.98	1.00	1.00	1.00	1.00
40	0.35	0.68	0.63	0.88	0.97	0.99	1.00	0.99	1.00	1.00
41	0.34	0.32	0.73	0.82	0.91	0.99	1.00	0.99	1.00	1.00
42	0.32	0.44	0.59	0.78	0.88	0.99	0.99	0.99	1.00	1.00

Table 3: POD according to each scenario and each k2 value, k1 = 2, alpha = 0.05

	k2=1	k2=2	k2=3	k2=4	k2=5	k2=6	k2=7	k2=8	k2=9	k2=10
1	0.28	0.38	0.55	0.67	0.84	0.83	0.92	0.97	0.94	0.97
2	0.31	0.49	0.56	0.58	0.73	0.85	0.94	0.92	0.96	0.96
3	0.25	0.38	0.52	0.54	0.73	0.78	0.87	0.92	0.93	0.99
4	0.36	0.50	0.66	0.84	0.89	0.96	0.97	0.99	0.98	1.00
5	0.43	0.46	0.60	0.88	0.88	0.92	0.97	0.99	1.00	1.00
6	0.32	0.50	0.52	0.68	0.72	0.87	0.92	0.97	0.96	1.00
7	0.51	0.53	0.64	0.70	0.74	0.79	0.85	0.88	0.90	0.92
8	0.60	0.69	0.77	0.75	0.75	0.84	0.87	0.85	0.85	0.88
9	0.65	0.65	0.60	0.73	0.80	0.85	0.79	0.87	0.89	0.90
10	0.49	0.53	0.76	0.89	0.92	0.98	0.99	1.00	0.99	1.00
11	0.41	0.58	0.77	0.92	0.95	0.97	0.97	1.00	1.00	1.00
12	0.46	0.51	0.68	0.80	0.93	0.94	0.98	1.00	1.00	0.99
13	0.25	0.35	0.47	0.65	0.77	0.85	0.93	0.94	0.99	1.00
14	0.21	0.27	0.54	0.64	0.77	0.80	0.90	0.97	0.98	0.99
15	0.18	0.31	0.59	0.63	0.68	0.82	0.89	0.96	0.99	0.98
16	0.44	0.55	0.83	0.97	0.95	0.97	1.00	1.00	1.00	1.00
17	0.46	0.75	0.78	0.94	0.96	0.97	1.00	0.98	0.99	1.00
18	0.37	0.57	0.74	0.78	0.82	0.96	0.96	1.00	1.00	1.00
19	0.35	0.66	0.77	0.93	0.95	0.99	1.00	1.00	1.00	1.00
20	0.38	0.64	0.77	0.87	0.98	0.97	0.99	1.00	1.00	1.00
21	0.32	0.62	0.77	0.86	0.95	0.96	0.98	1.00	1.00	1.00
22	0.52	0.75	0.86	0.95	1.00	1.00	1.00	1.00	1.00	1.00
23	0.44	0.68	0.88	0.94	1.00	0.99	1.00	1.00	1.00	1.00
24	0.43	0.72	0.81	0.94	0.99	1.00	1.00	1.00	1.00	1.00
25	0.38	0.63	0.80	0.95	0.98	0.99	1.00	1.00	1.00	1.00
26	0.43	0.55	0.76	0.83	0.95	0.97	0.97	1.00	1.00	1.00
27	0.39	0.40	0.64	0.63	0.75	0.86	0.89	0.96	0.95	1.00
28	0.50	0.71	0.85	0.94	0.99	1.00	1.00	1.00	1.00	1.00
29	0.49	0.62	0.82	0.90	0.97	0.99	1.00	1.00	1.00	1.00
30	0.41	0.53	0.60	0.66	0.70	0.81	0.84	0.90	0.92	0.98
31	0.25	0.40	0.51	0.72	0.85	0.98	0.96	1.00	1.00	1.00
32	0.20	0.50	0.59	0.79	0.83	0.93	0.97	1.00	1.00	1.00
33	0.28	0.42	0.59	0.77	0.82	0.94	0.98	1.00	1.00	1.00
34	0.32	0.57	0.71	0.81	0.94	0.98	1.00	1.00	1.00	1.00
35	0.43	0.59	0.74	0.86	0.95	0.97	1.00	1.00	1.00	1.00
36	0.39	0.54	0.77	0.83	0.96	0.96	1.00	1.00	1.00	1.00
37	0.30	0.47	0.72	0.79	0.87	0.96	1.00	0.98	1.00	1.00
38	0.27	0.46	0.62	0.83	0.90	0.97	0.98	1.00	1.00	1.00
39	0.30	0.45	0.65	0.84	0.91	0.96	0.98	1.00	1.00	1.00
40	0.37	0.58	0.68	0.83	0.90	0.99	1.00	1.00	1.00	1.00
41	0.27	0.50	0.68	0.80	0.91	0.98	0.98	1.00	1.00	1.00
42	0.32	0.56	0.63	0.83	0.93	0.98	0.99	1.00	1.00	1.00

Table 4: POD according to each scenario and each k2 value, k1 = 3, alpha = 0.05

	k2=1	k2=2	k2=3	k2=4	k2=5	k2=6	k2=7	k2=8	k2=9	k2=10
1	0.30	0.39	0.54	0.68	0.72	0.79	0.93	0.99	0.97	0.98
2	0.31	0.33	0.56	0.66	0.76	0.86	0.90	0.95	0.93	0.98
3	0.25	0.38	0.49	0.62	0.78	0.75	0.82	0.89	0.94	0.94
4	0.42	0.49	0.66	0.78	0.89	0.94	0.94	1.00	1.00	1.00
5	0.31	0.50	0.67	0.75	0.89	0.93	0.97	0.98	1.00	1.00
6	0.28	0.37	0.58	0.74	0.73	0.91	0.92	0.97	0.97	0.99
7	0.51	0.66	0.62	0.78	0.78	0.69	0.81	0.85	0.92	0.94
8	0.63	0.58	0.75	0.66	0.69	0.70	0.79	0.94	0.87	0.90
9	0.54	0.49	0.65	0.63	0.75	0.80	0.80	0.90	0.89	0.89
10	0.32	0.54	0.73	0.80	0.96	0.98	0.97	0.99	1.00	1.00
11	0.45	0.53	0.71	0.88	0.97	0.96	0.98	0.99	1.00	1.00
12	0.38	0.59	0.73	0.86	0.93	0.97	1.00	1.00	0.99	1.00
13	0.24	0.37	0.53	0.65	0.74	0.86	0.90	0.98	0.99	0.99
14	0.16	0.33	0.60	0.64	0.79	0.84	0.91	0.97	1.00	1.00
15	0.24	0.28	0.52	0.60	0.73	0.87	0.90	0.97	0.96	0.99
16	0.45	0.63	0.71	0.91	0.96	1.00	0.98	1.00	1.00	1.00
17	0.44	0.61	0.79	0.87	0.94	0.99	0.99	1.00	1.00	1.00
18	0.48	0.64	0.74	0.69	0.81	0.89	0.98	0.97	1.00	0.99
19	0.36	0.71	0.74	0.87	0.94	0.99	1.00	1.00	1.00	1.00
20	0.44	0.56	0.76	0.85	0.96	0.99	0.99	1.00	1.00	1.00
21	0.35	0.45	0.69	0.87	0.95	0.97	1.00	0.98	1.00	1.00
22	0.47	0.78	0.88	0.94	0.98	0.99	1.00	1.00	1.00	1.00
23	0.45	0.67	0.86	0.93	0.97	1.00	1.00	1.00	1.00	1.00
24	0.56	0.65	0.81	0.90	0.99	1.00	1.00	1.00	1.00	1.00
25	0.45	0.65	0.82	0.90	0.94	0.99	0.99	1.00	1.00	1.00
26	0.39	0.58	0.85	0.85	0.96	1.00	0.99	1.00	1.00	1.00
27	0.37	0.41	0.59	0.64	0.85	0.88	0.90	0.97	0.97	0.99
28	0.48	0.70	0.84	0.91	0.93	0.98	1.00	1.00	1.00	1.00
29	0.54	0.61	0.79	0.88	0.96	0.99	1.00	1.00	1.00	1.00
30	0.37	0.52	0.61	0.61	0.81	0.75	0.93	0.97	0.97	0.96
31	0.26	0.41	0.59	0.74	0.84	0.94	0.99	0.99	1.00	1.00
32	0.29	0.45	0.66	0.71	0.85	0.91	0.97	0.97	1.00	1.00
33	0.22	0.46	0.56	0.72	0.90	0.94	0.97	0.99	1.00	1.00
34	0.24	0.58	0.75	0.87	0.98	0.99	0.99	1.00	1.00	1.00
35	0.36	0.66	0.75	0.87	0.93	1.00	1.00	1.00	1.00	1.00
36	0.39	0.53	0.71	0.82	0.91	0.96	0.98	0.99	1.00	1.00
37	0.27	0.38	0.66	0.79	0.93	0.98	0.99	1.00	0.99	1.00
38	0.21	0.41	0.57	0.77	0.95	1.00	1.00	1.00	1.00	1.00
39	0.27	0.28	0.73	0.79	0.89	0.99	0.99	1.00	1.00	1.00
40	0.32	0.51	0.72	0.79	0.98	0.97	1.00	1.00	1.00	1.00
41	0.30	0.39	0.66	0.83	0.89	0.96	1.00	1.00	1.00	1.00
42	0.29	0.41	0.59	0.84	0.93	0.99	0.99	1.00	1.00	1.00

Table 5: POD according to each scenario and each k2 value, k1 = 5, alpha = 0.05

	k2=1	k2=2	k2=3	k2=4	k2=5	k2=6	k2=7	k2=8	k2=9	k2=10
1	0.37	0.45	0.49	0.71	0.74	0.82	0.90	0.93	0.92	0.95
2	0.27	0.37	0.59	0.57	0.78	0.84	0.91	0.90	0.93	0.97
3	0.36	0.34	0.48	0.67	0.63	0.80	0.81	0.91	0.95	0.95
4	0.35	0.49	0.59	0.78	0.82	0.92	0.97	1.00	0.99	1.00
5	0.33	0.51	0.56	0.66	0.89	0.95	0.95	0.98	1.00	1.00
6	0.22	0.39	0.56	0.65	0.71	0.80	0.91	0.93	0.99	1.00
7	0.56	0.52	0.66	0.71	0.77	0.77	0.72	0.82	0.93	0.86
8	0.44	0.59	0.65	0.70	0.81	0.75	0.85	0.85	0.88	0.89
9	0.54	0.46	0.63	0.71	0.74	0.72	0.75	0.86	0.85	0.89
10	0.37	0.52	0.74	0.80	0.90	0.97	0.98	0.97	0.98	1.00
11	0.36	0.55	0.72	0.80	0.88	0.92	0.96	0.98	1.00	0.99
12	0.31	0.54	0.69	0.77	0.91	0.94	0.98	0.99	1.00	1.00
13	0.16	0.29	0.44	0.60	0.80	0.87	0.90	0.95	0.97	1.00
14	0.20	0.24	0.46	0.55	0.79	0.83	0.89	0.95	0.99	0.99
15	0.17	0.21	0.45	0.50	0.65	0.78	0.89	0.96	0.94	0.97
16	0.34	0.55	0.77	0.87	0.94	0.93	0.99	1.00	1.00	1.00
17	0.43	0.65	0.76	0.84	0.96	0.98	0.98	1.00	1.00	1.00
18	0.39	0.52	0.62	0.77	0.89	0.91	0.95	0.98	0.99	0.98
19	0.31	0.61	0.67	0.79	0.96	0.97	0.98	1.00	1.00	1.00
20	0.42	0.57	0.70	0.85	0.94	0.97	1.00	0.99	1.00	1.00
21	0.38	0.53	0.72	0.80	0.86	0.97	0.98	1.00	0.97	1.00
22	0.49	0.70	0.81	0.93	0.98	0.98	1.00	1.00	1.00	1.00
23	0.52	0.63	0.87	0.91	0.97	1.00	1.00	0.99	1.00	1.00
24	0.41	0.65	0.78	0.93	0.97	0.96	1.00	0.99	1.00	1.00
25	0.45	0.60	0.75	0.87	0.98	0.99	0.99	1.00	1.00	1.00
26	0.44	0.52	0.70	0.81	0.89	0.96	0.98	0.99	1.00	1.00
27	0.30	0.45	0.41	0.70	0.66	0.79	0.87	0.91	0.98	0.95
28	0.49	0.64	0.86	0.93	0.96	0.99	1.00	1.00	1.00	1.00
29	0.42	0.68	0.79	0.90	0.99	0.96	1.00	1.00	1.00	1.00
30	0.47	0.53	0.53	0.67	0.71	0.76	0.86	0.93	0.98	0.97
31	0.27	0.31	0.55	0.69	0.85	0.97	0.95	0.99	1.00	1.00
32	0.22	0.43	0.55	0.73	0.79	0.93	0.99	0.99	1.00	1.00
33	0.18	0.34	0.45	0.73	0.79	0.89	0.93	0.99	1.00	1.00
34	0.34	0.49	0.65	0.82	0.93	0.97	0.99	1.00	1.00	1.00
35	0.36	0.55	0.71	0.81	0.92	0.98	0.99	1.00	1.00	1.00
36	0.39	0.44	0.71	0.77	0.87	0.95	0.99	0.99	1.00	1.00
37	0.27	0.39	0.58	0.78	0.92	0.93	0.97	0.99	1.00	1.00
38	0.28	0.37	0.71	0.78	0.86	0.93	1.00	0.99	1.00	1.00
39	0.19	0.48	0.58	0.70	0.89	0.93	0.99	1.00	1.00	1.00
40	0.30	0.44	0.59	0.87	0.92	0.95	0.99	0.99	1.00	1.00
41	0.33	0.45	0.58	0.76	0.94	0.91	1.00	1.00	1.00	1.00
42	0.29	0.46	0.55	0.82	0.89	0.97	0.98	1.00	1.00	1.00

Table 6: POD according to each scenario and each k2 value, k1 = 10, alpha = 0.05
