# Supplementary material for: Evaluation and comparison of statistical methods for early temporal detection of outbreaks: A simulation-based study
Source: PLoS One. 2017 Jul 17;12(7):e0181227. doi: 10.1371/journal.pone.0181227 (PMC5513450; doi:10.1371/journal.pone.0181227)
Supplement: S13 Appendix — (PDF) [file pone.0181227.s013.pdf]

Evaluation and Comparison of Statistical Methods for Early Temporal Detection of Outbreaks: a Simulation-Based Study

Appendix S13: Overall performances of Bayes 3 algorithm ($\alpha = 0.05$)

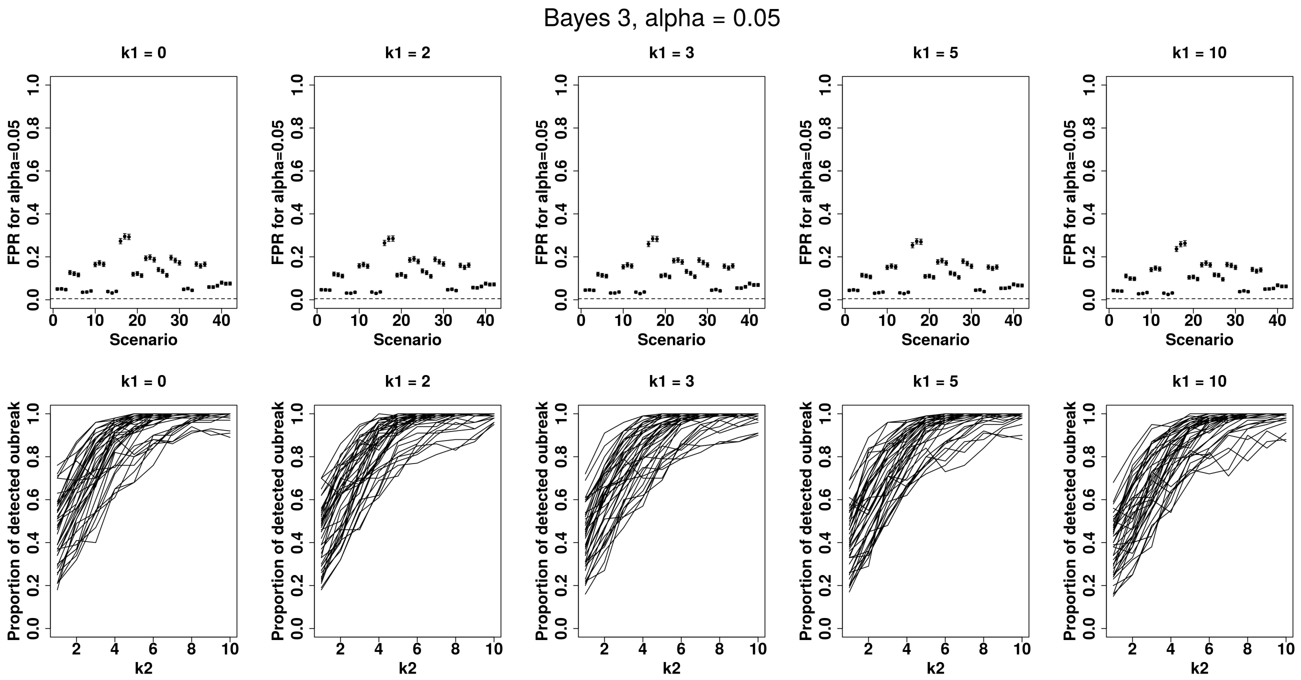

Figure 1: Bayes 3 algorithm performances for $\alpha = 0.05$ by increasing past outbreak amplitude $k_1 = 0$, 2, 3, 5 or 10 with (i) on the first row: false positive rate for 42 simulated scenarios, (ii) on the second row: probability of detection for 42 simulated scenarios (each curve corresponding to a scenario) by increasing current outbreak amplitude $k_2 = 1$ to 10.

Overall performances of Bayes 3 algorithm ($\alpha = 0.05$)

	FPR k1=0	FPR k1=2	FPR k1=3	FPR k1=5	FPR k1=10
1	0.05	0.05	0.04	0.04	0.04
2	0.05	0.05	0.05	0.05	0.04
3	0.05	0.04	0.04	0.04	0.04
4	0.13	0.12	0.12	0.11	0.11
5	0.12	0.12	0.11	0.11	0.10
6	0.12	0.11	0.11	0.11	0.10
7	0.03	0.03	0.03	0.03	0.03
8	0.04	0.03	0.03	0.03	0.03
9	0.04	0.03	0.04	0.04	0.03
10	0.16	0.16	0.15	0.15	0.14
11	0.17	0.16	0.16	0.16	0.15
12	0.17	0.16	0.16	0.15	0.14
13	0.04	0.04	0.04	0.03	0.03
14	0.03	0.03	0.03	0.03	0.03
15	0.04	0.04	0.04	0.04	0.03
16	0.27	0.26	0.26	0.25	0.24
17	0.30	0.28	0.28	0.27	0.26
18	0.29	0.29	0.28	0.27	0.26
19	0.12	0.11	0.11	0.11	0.10
20	0.12	0.12	0.11	0.11	0.11
21	0.11	0.11	0.11	0.10	0.10
22	0.19	0.19	0.18	0.18	0.16
23	0.20	0.19	0.19	0.18	0.17
24	0.19	0.18	0.18	0.17	0.16
25	0.14	0.13	0.13	0.12	0.12
26	0.13	0.13	0.12	0.12	0.11
27	0.11	0.11	0.11	0.10	0.10
28	0.20	0.19	0.18	0.18	0.16
29	0.18	0.18	0.17	0.17	0.16
30	0.17	0.17	0.16	0.16	0.15
31	0.05	0.05	0.04	0.04	0.04
32	0.05	0.05	0.05	0.05	0.04
33	0.04	0.04	0.04	0.04	0.04
34	0.17	0.16	0.16	0.15	0.14
35	0.16	0.15	0.15	0.15	0.13
36	0.17	0.16	0.16	0.15	0.14
37	0.06	0.06	0.05	0.05	0.05
38	0.06	0.06	0.05	0.05	0.05
39	0.07	0.06	0.06	0.06	0.05
40	0.08	0.08	0.08	0.07	0.07
41	0.07	0.07	0.07	0.07	0.06
42	0.08	0.07	0.07	0.07	0.06

Table 1: FPR according to each scenario and each k1 value, alpha = 0.05

	k2=1	k2=2	k2=3	k2=4	k2=5	k2=6	k2=7	k2=8	k2=9	k2=10
1	0.36	0.45	0.54	0.61	0.80	0.84	0.94	0.96	0.99	0.98
2	0.30	0.37	0.54	0.82	0.80	0.86	0.92	0.98	0.98	0.99
3	0.37	0.39	0.47	0.65	0.68	0.76	0.91	0.97	0.97	0.97
4	0.47	0.66	0.75	0.91	0.92	1.00	0.99	1.00	1.00	1.00
5	0.51	0.58	0.77	0.90	0.91	0.94	1.00	1.00	1.00	1.00
6	0.50	0.56	0.71	0.88	0.91	0.97	0.98	0.98	1.00	1.00
7	0.70	0.69	0.74	0.75	0.85	0.88	0.87	0.94	0.90	0.91
8	0.63	0.65	0.74	0.89	0.81	0.88	0.86	0.91	0.93	0.92
9	0.71	0.78	0.70	0.76	0.76	0.86	0.87	0.92	0.92	0.89
10	0.58	0.68	0.84	0.97	0.95	0.97	0.99	1.00	1.00	1.00
11	0.55	0.70	0.86	0.96	0.99	0.99	1.00	1.00	0.99	1.00
12	0.54	0.69	0.86	0.92	0.97	1.00	1.00	1.00	1.00	1.00
13	0.21	0.32	0.51	0.72	0.83	0.90	0.90	0.96	1.00	1.00
14	0.26	0.34	0.55	0.63	0.68	0.90	0.93	0.96	0.96	1.00
15	0.28	0.41	0.40	0.65	0.73	0.84	0.93	0.97	1.00	1.00
16	0.70	0.86	0.96	0.98	1.00	0.99	1.00	1.00	1.00	1.00
17	0.72	0.87	0.96	0.97	1.00	1.00	1.00	1.00	1.00	1.00
18	0.76	0.82	0.91	0.95	0.99	0.98	1.00	1.00	1.00	1.00
19	0.39	0.58	0.83	0.89	0.97	1.00	1.00	1.00	1.00	1.00
20	0.48	0.63	0.81	0.91	0.98	0.99	1.00	0.99	1.00	1.00
21	0.37	0.53	0.79	0.87	0.94	1.00	0.99	1.00	1.00	1.00
22	0.54	0.81	0.93	0.97	1.00	1.00	1.00	1.00	1.00	1.00
23	0.59	0.79	0.89	0.96	0.99	1.00	1.00	1.00	1.00	1.00
24	0.55	0.71	0.88	0.98	1.00	1.00	1.00	1.00	1.00	1.00
25	0.51	0.68	0.83	0.94	0.97	0.99	1.00	1.00	1.00	1.00
26	0.46	0.66	0.85	0.92	0.99	0.98	0.99	1.00	1.00	1.00
27	0.39	0.63	0.68	0.86	0.93	0.95	0.99	1.00	1.00	1.00
28	0.59	0.80	0.93	0.96	1.00	1.00	1.00	1.00	1.00	1.00
29	0.59	0.69	0.89	0.94	0.97	0.99	0.99	1.00	1.00	1.00
30	0.44	0.69	0.70	0.92	0.96	1.00	0.99	1.00	1.00	1.00
31	0.21	0.49	0.54	0.73	0.87	1.00	1.00	1.00	0.99	1.00
32	0.25	0.46	0.68	0.76	0.92	0.97	0.99	1.00	0.99	1.00
33	0.21	0.36	0.60	0.83	0.94	0.95	0.97	0.99	1.00	1.00
34	0.58	0.74	0.89	0.94	0.99	1.00	1.00	1.00	1.00	1.00
35	0.57	0.72	0.93	0.97	0.99	1.00	0.99	1.00	1.00	1.00
36	0.52	0.66	0.86	0.94	0.98	0.99	0.99	1.00	1.00	1.00
37	0.18	0.51	0.67	0.84	0.93	0.97	0.98	0.99	0.99	1.00
38	0.30	0.37	0.67	0.83	0.92	0.97	1.00	1.00	1.00	1.00
39	0.29	0.51	0.69	0.91	0.97	0.99	1.00	1.00	1.00	1.00
40	0.34	0.55	0.73	0.95	0.91	0.97	1.00	1.00	1.00	1.00
41	0.39	0.56	0.66	0.92	0.97	0.97	0.99	1.00	1.00	1.00
42	0.34	0.53	0.68	0.83	0.92	0.97	1.00	1.00	1.00	1.00

Table 2: POD according to each scenario and each k2 value, k1 = 0, alpha = 0.05

	k2=1	k2=2	k2=3	k2=4	k2=5	k2=6	k2=7	k2=8	k2=9	k2=10
1	0.29	0.43	0.60	0.62	0.80	0.82	0.90	0.96	0.93	0.98
2	0.30	0.46	0.46	0.65	0.87	0.91	0.91	0.92	0.93	0.98
3	0.24	0.37	0.57	0.69	0.71	0.86	0.96	0.95	0.96	0.98
4	0.48	0.70	0.78	0.92	0.94	0.98	0.98	1.00	1.00	1.00
5	0.50	0.64	0.80	0.89	0.93	0.99	0.98	1.00	0.99	1.00
6	0.35	0.57	0.71	0.80	0.87	0.94	0.94	1.00	1.00	1.00
7	0.49	0.63	0.67	0.74	0.78	0.81	0.87	0.88	0.88	0.95
8	0.70	0.62	0.70	0.70	0.76	0.77	0.81	0.84	0.87	0.96
9	0.56	0.65	0.69	0.75	0.80	0.85	0.86	0.83	0.90	0.96
10	0.53	0.68	0.85	0.91	0.91	0.99	0.99	0.99	1.00	1.00
11	0.49	0.69	0.88	0.83	0.97	1.00	0.99	1.00	1.00	1.00
12	0.56	0.73	0.82	0.87	0.97	1.00	1.00	0.99	1.00	1.00
13	0.19	0.32	0.53	0.73	0.82	0.86	0.96	0.98	1.00	0.99
14	0.18	0.32	0.54	0.61	0.73	0.83	0.92	0.93	0.99	0.99
15	0.27	0.41	0.47	0.64	0.85	0.88	0.93	0.98	0.96	0.99
16	0.70	0.86	0.95	0.98	0.99	1.00	1.00	1.00	1.00	1.00
17	0.70	0.77	0.92	1.00	0.99	1.00	1.00	1.00	1.00	1.00
18	0.65	0.80	0.94	0.98	0.96	1.00	1.00	1.00	1.00	1.00
19	0.45	0.66	0.75	0.92	0.96	0.99	1.00	1.00	1.00	1.00
20	0.45	0.62	0.73	0.95	0.94	0.99	0.99	0.99	1.00	1.00
21	0.39	0.47	0.74	0.87	0.97	0.96	1.00	0.99	1.00	1.00
22	0.48	0.76	0.85	0.97	0.98	1.00	1.00	1.00	1.00	1.00
23	0.55	0.73	0.86	0.97	0.99	1.00	1.00	1.00	1.00	1.00
24	0.62	0.77	0.89	0.93	0.99	1.00	1.00	1.00	1.00	1.00
25	0.47	0.66	0.80	0.94	1.00	0.99	1.00	1.00	1.00	1.00
26	0.42	0.61	0.86	0.85	0.96	1.00	1.00	1.00	1.00	1.00
27	0.46	0.50	0.68	0.86	0.94	0.99	0.99	1.00	0.99	1.00
28	0.50	0.76	0.82	0.97	1.00	1.00	1.00	1.00	1.00	1.00
29	0.62	0.74	0.81	0.95	0.99	1.00	1.00	1.00	1.00	1.00
30	0.39	0.62	0.71	0.93	0.95	0.97	1.00	1.00	1.00	1.00
31	0.26	0.36	0.58	0.80	0.91	0.97	0.97	1.00	1.00	1.00
32	0.27	0.40	0.68	0.77	0.92	0.98	0.97	1.00	0.99	1.00
33	0.22	0.47	0.59	0.81	0.91	0.95	0.96	0.99	1.00	1.00
34	0.52	0.81	0.89	0.96	0.97	0.99	0.99	1.00	1.00	1.00
35	0.54	0.76	0.79	0.87	0.98	0.99	1.00	1.00	1.00	1.00
36	0.51	0.63	0.90	0.93	0.98	0.98	1.00	1.00	1.00	1.00
37	0.29	0.42	0.67	0.78	0.96	0.95	0.99	1.00	1.00	1.00
38	0.21	0.45	0.67	0.79	0.93	0.98	0.99	1.00	1.00	1.00
39	0.23	0.55	0.63	0.89	0.90	0.97	1.00	1.00	1.00	1.00
40	0.33	0.70	0.67	0.92	0.98	0.99	0.99	1.00	1.00	1.00
41	0.33	0.40	0.76	0.88	0.94	0.99	0.99	0.99	1.00	1.00
42	0.37	0.48	0.68	0.79	0.91	0.99	0.99	1.00	1.00	1.00

Table 3: POD according to each scenario and each k2 value, k1 = 2, alpha = 0.05

	k2=1	k2=2	k2=3	k2=4	k2=5	k2=6	k2=7	k2=8	k2=9	k2=10
1	0.31	0.38	0.56	0.67	0.85	0.83	0.90	0.97	0.94	0.98
2	0.34	0.51	0.52	0.57	0.79	0.85	0.95	0.91	0.94	0.96
3	0.29	0.43	0.52	0.61	0.79	0.79	0.88	0.92	0.96	0.99
4	0.46	0.55	0.71	0.84	0.93	0.99	0.98	0.99	0.99	1.00
5	0.48	0.65	0.69	0.89	0.94	0.98	0.98	0.99	1.00	1.00
6	0.37	0.60	0.71	0.80	0.79	0.96	0.98	1.00	0.98	1.00
7	0.51	0.57	0.65	0.75	0.74	0.79	0.82	0.87	0.87	0.91
8	0.59	0.72	0.79	0.70	0.70	0.86	0.85	0.86	0.87	0.90
9	0.61	0.66	0.69	0.71	0.75	0.81	0.85	0.86	0.89	0.91
10	0.59	0.68	0.83	0.92	0.96	0.99	1.00	1.00	0.99	1.00
11	0.60	0.72	0.83	0.96	0.98	1.00	0.99	1.00	1.00	1.00
12	0.54	0.59	0.82	0.86	0.95	0.95	0.99	1.00	1.00	1.00
13	0.22	0.36	0.47	0.61	0.77	0.84	0.92	0.94	0.99	0.99
14	0.22	0.27	0.56	0.64	0.80	0.85	0.93	0.98	0.99	0.99
15	0.16	0.31	0.60	0.62	0.69	0.87	0.95	0.97	1.00	0.97
16	0.69	0.85	0.92	0.99	1.00	0.99	1.00	1.00	1.00	1.00
17	0.72	0.91	0.96	0.99	0.98	1.00	1.00	1.00	0.99	1.00
18	0.61	0.80	0.92	0.94	0.98	1.00	1.00	1.00	1.00	1.00
19	0.38	0.63	0.78	0.92	0.96	0.99	1.00	0.99	1.00	1.00
20	0.44	0.65	0.75	0.84	0.96	0.97	1.00	1.00	1.00	1.00
21	0.31	0.62	0.79	0.88	0.95	0.97	0.98	1.00	1.00	1.00
22	0.52	0.73	0.90	0.99	0.99	1.00	1.00	1.00	1.00	1.00
23	0.49	0.76	0.92	0.97	1.00	1.00	1.00	1.00	1.00	1.00
24	0.46	0.73	0.85	0.96	1.00	1.00	1.00	1.00	1.00	1.00
25	0.39	0.62	0.83	0.96	0.98	0.99	1.00	1.00	1.00	1.00
26	0.42	0.60	0.81	0.91	0.96	0.99	1.00	1.00	1.00	1.00
27	0.34	0.52	0.67	0.80	0.92	0.97	0.98	1.00	1.00	1.00
28	0.53	0.76	0.88	0.96	1.00	1.00	1.00	1.00	1.00	1.00
29	0.59	0.73	0.87	0.95	0.99	1.00	1.00	1.00	1.00	1.00
30	0.46	0.63	0.76	0.88	0.91	0.97	1.00	0.99	1.00	1.00
31	0.21	0.40	0.54	0.72	0.87	0.98	0.96	1.00	0.99	1.00
32	0.20	0.41	0.59	0.74	0.83	0.93	0.96	1.00	1.00	1.00
33	0.29	0.39	0.58	0.79	0.85	0.95	0.99	1.00	1.00	1.00
34	0.43	0.71	0.79	0.93	0.96	1.00	1.00	1.00	1.00	1.00
35	0.59	0.74	0.88	0.91	0.99	0.99	1.00	1.00	1.00	1.00
36	0.45	0.69	0.88	0.93	1.00	0.99	1.00	1.00	1.00	1.00
37	0.27	0.53	0.76	0.79	0.87	0.96	0.99	1.00	1.00	1.00
38	0.30	0.47	0.62	0.83	0.88	0.96	0.99	0.99	1.00	1.00
39	0.23	0.46	0.68	0.81	0.93	0.97	0.98	1.00	1.00	1.00
40	0.35	0.65	0.71	0.84	0.90	0.98	0.99	1.00	1.00	1.00
41	0.31	0.46	0.67	0.85	0.93	0.99	0.99	1.00	1.00	1.00
42	0.38	0.62	0.68	0.85	0.95	0.98	0.99	1.00	1.00	1.00

Table 4: POD according to each scenario and each k2 value, k1 = 3, alpha = 0.05

	k2=1	k2=2	k2=3	k2=4	k2=5	k2=6	k2=7	k2=8	k2=9	k2=10
1	0.33	0.36	0.59	0.62	0.74	0.82	0.91	0.99	0.97	0.98
2	0.33	0.34	0.51	0.67	0.76	0.87	0.89	0.95	0.95	0.99
3	0.25	0.45	0.48	0.65	0.80	0.78	0.87	0.88	0.94	0.98
4	0.50	0.60	0.71	0.83	0.96	0.95	0.97	1.00	1.00	1.00
5	0.39	0.59	0.76	0.85	0.94	0.97	0.99	0.99	1.00	1.00
6	0.37	0.51	0.67	0.84	0.87	0.97	0.97	1.00	1.00	1.00
7	0.55	0.61	0.62	0.78	0.84	0.73	0.76	0.84	0.93	0.95
8	0.61	0.55	0.71	0.66	0.72	0.76	0.85	0.92	0.89	0.90
9	0.58	0.53	0.71	0.69	0.76	0.84	0.82	0.91	0.90	0.88
10	0.41	0.71	0.86	0.87	0.98	0.99	0.99	0.99	1.00	1.00
11	0.56	0.66	0.82	0.94	0.99	0.97	1.00	1.00	1.00	0.99
12	0.46	0.69	0.80	0.93	0.97	0.98	1.00	1.00	0.99	1.00
13	0.20	0.36	0.52	0.66	0.77	0.86	0.90	0.98	0.99	0.99
14	0.17	0.36	0.51	0.65	0.77	0.86	0.94	0.96	0.99	0.99
15	0.20	0.33	0.55	0.66	0.80	0.87	0.93	0.97	0.96	1.00
16	0.69	0.89	0.96	0.96	0.99	1.00	1.00	1.00	1.00	1.00
17	0.69	0.80	0.96	0.97	0.99	0.99	1.00	1.00	1.00	1.00
18	0.65	0.84	0.87	0.94	0.97	0.99	0.99	0.99	1.00	1.00
19	0.38	0.68	0.77	0.87	0.96	0.98	0.99	1.00	1.00	1.00
20	0.42	0.54	0.71	0.91	0.97	0.98	0.99	1.00	1.00	1.00
21	0.38	0.51	0.67	0.88	0.97	0.98	0.99	1.00	1.00	1.00
22	0.48	0.83	0.90	0.96	0.99	0.99	1.00	1.00	1.00	1.00
23	0.51	0.71	0.88	0.97	0.99	0.99	1.00	1.00	1.00	1.00
24	0.54	0.69	0.86	0.92	0.99	0.99	0.99	1.00	1.00	1.00
25	0.44	0.61	0.80	0.90	0.97	0.99	1.00	1.00	1.00	1.00
26	0.40	0.58	0.83	0.90	0.98	1.00	1.00	1.00	1.00	1.00
27	0.34	0.51	0.66	0.79	0.93	0.97	0.98	1.00	1.00	1.00
28	0.54	0.78	0.85	0.92	0.96	0.99	1.00	1.00	1.00	1.00
29	0.57	0.74	0.84	0.91	0.96	0.98	1.00	1.00	1.00	1.00
30	0.46	0.66	0.79	0.86	0.94	0.96	1.00	1.00	1.00	1.00
31	0.23	0.39	0.56	0.73	0.82	0.95	1.00	1.00	1.00	1.00
32	0.30	0.44	0.63	0.70	0.87	0.91	0.96	1.00	1.00	1.00
33	0.19	0.38	0.56	0.74	0.92	0.93	0.96	0.98	1.00	1.00
34	0.46	0.71	0.88	0.96	0.98	1.00	0.99	1.00	1.00	1.00
35	0.51	0.82	0.83	0.89	0.98	1.00	1.00	1.00	1.00	1.00
36	0.46	0.67	0.80	0.91	0.97	0.99	0.99	1.00	1.00	1.00
37	0.26	0.35	0.68	0.77	0.94	0.97	0.99	1.00	0.98	1.00
38	0.20	0.45	0.60	0.78	0.94	1.00	1.00	0.99	1.00	1.00
39	0.26	0.29	0.71	0.80	0.88	0.98	0.98	1.00	1.00	1.00
40	0.33	0.54	0.73	0.82	0.95	0.97	1.00	1.00	1.00	1.00
41	0.36	0.44	0.65	0.81	0.91	0.96	1.00	1.00	1.00	1.00
42	0.25	0.47	0.63	0.84	0.91	0.99	0.99	1.00	1.00	1.00

Table 5: POD according to each scenario and each k2 value, k1 = 5, alpha = 0.05

	k2=1	k2=2	k2=3	k2=4	k2=5	k2=6	k2=7	k2=8	k2=9	k2=10
1	0.39	0.40	0.43	0.64	0.73	0.80	0.90	0.88	0.93	0.96
2	0.28	0.38	0.60	0.54	0.77	0.81	0.91	0.93	0.95	0.98
3	0.38	0.35	0.50	0.64	0.72	0.76	0.82	0.93	0.97	0.98
4	0.40	0.56	0.65	0.86	0.84	0.98	0.98	1.00	1.00	1.00
5	0.34	0.61	0.61	0.74	0.94	0.97	0.97	0.99	1.00	1.00
6	0.32	0.43	0.69	0.81	0.87	0.92	0.96	1.00	1.00	1.00
7	0.50	0.46	0.69	0.63	0.73	0.82	0.71	0.82	0.92	0.87
8	0.47	0.57	0.60	0.70	0.83	0.79	0.84	0.78	0.87	0.88
9	0.56	0.51	0.73	0.66	0.74	0.72	0.74	0.90	0.84	0.91
10	0.45	0.63	0.78	0.91	0.96	0.97	0.98	0.98	0.99	1.00
11	0.46	0.66	0.81	0.86	0.94	0.97	0.96	1.00	1.00	1.00
12	0.45	0.67	0.76	0.91	0.94	0.99	0.98	0.99	1.00	1.00
13	0.15	0.28	0.43	0.62	0.80	0.84	0.92	0.95	0.97	1.00
14	0.20	0.25	0.47	0.59	0.81	0.81	0.91	0.97	0.97	0.98
15	0.16	0.25	0.48	0.55	0.69	0.84	0.91	0.95	0.96	0.97
16	0.59	0.79	0.93	0.96	0.99	0.98	0.99	1.00	1.00	1.00
17	0.68	0.83	0.95	0.94	1.00	1.00	1.00	1.00	1.00	1.00
18	0.58	0.74	0.86	0.92	0.98	0.96	0.98	1.00	1.00	1.00
19	0.29	0.56	0.73	0.78	0.94	0.98	0.97	0.99	1.00	1.00
20	0.43	0.53	0.64	0.84	0.93	0.96	1.00	0.98	1.00	1.00
21	0.38	0.47	0.76	0.81	0.86	0.97	0.97	0.98	0.98	1.00
22	0.48	0.73	0.82	0.94	0.98	1.00	1.00	1.00	1.00	1.00
23	0.54	0.66	0.87	0.93	0.98	1.00	1.00	1.00	1.00	1.00
24	0.50	0.67	0.80	0.93	0.97	0.99	1.00	1.00	1.00	1.00
25	0.40	0.59	0.71	0.88	0.95	0.99	1.00	1.00	1.00	1.00
26	0.43	0.50	0.70	0.83	0.92	0.98	0.98	0.98	1.00	1.00
27	0.30	0.46	0.58	0.79	0.80	0.88	0.97	0.98	0.99	1.00
28	0.50	0.68	0.86	0.93	0.97	1.00	1.00	1.00	1.00	1.00
29	0.44	0.76	0.86	0.91	0.98	0.96	0.99	1.00	1.00	1.00
30	0.52	0.61	0.67	0.86	0.92	0.97	0.98	1.00	1.00	0.99
31	0.25	0.33	0.55	0.71	0.86	0.96	0.98	0.99	1.00	1.00
32	0.16	0.41	0.55	0.76	0.82	0.95	0.98	0.99	1.00	1.00
33	0.23	0.32	0.38	0.73	0.81	0.88	0.94	0.99	1.00	1.00
34	0.48	0.71	0.80	0.87	0.96	0.97	1.00	1.00	1.00	1.00
35	0.47	0.68	0.83	0.88	0.96	0.99	1.00	1.00	1.00	1.00
36	0.51	0.59	0.87	0.90	0.94	0.98	0.99	1.00	1.00	1.00
37	0.24	0.43	0.61	0.79	0.94	0.91	0.98	0.99	1.00	1.00
38	0.24	0.38	0.73	0.82	0.88	0.92	0.99	0.99	1.00	1.00
39	0.25	0.40	0.56	0.70	0.89	0.94	0.99	1.00	1.00	1.00
40	0.30	0.42	0.59	0.87	0.91	0.93	0.99	0.98	1.00	1.00
41	0.34	0.43	0.63	0.76	0.94	0.96	0.99	0.99	1.00	1.00
42	0.26	0.54	0.58	0.80	0.93	0.98	0.99	1.00	1.00	1.00

Table 6: POD according to each scenario and each k2 value, k1 = 10, alpha = 0.05
