# Supplementary material for: Evaluation and comparison of statistical methods for early temporal detection of outbreaks: A simulation-based study
Source: PLoS One. 2017 Jul 17;12(7):e0181227. doi: 10.1371/journal.pone.0181227 (PMC5513450; doi:10.1371/journal.pone.0181227)
Supplement: S14 Appendix — (PDF) [file pone.0181227.s014.pdf]

Evaluation and Comparison of Statistical Methods for Early Temporal Detection of Outbreaks: a Simulation-Based Study

Appendix S14: Overall performances of RKI 1 algorithm

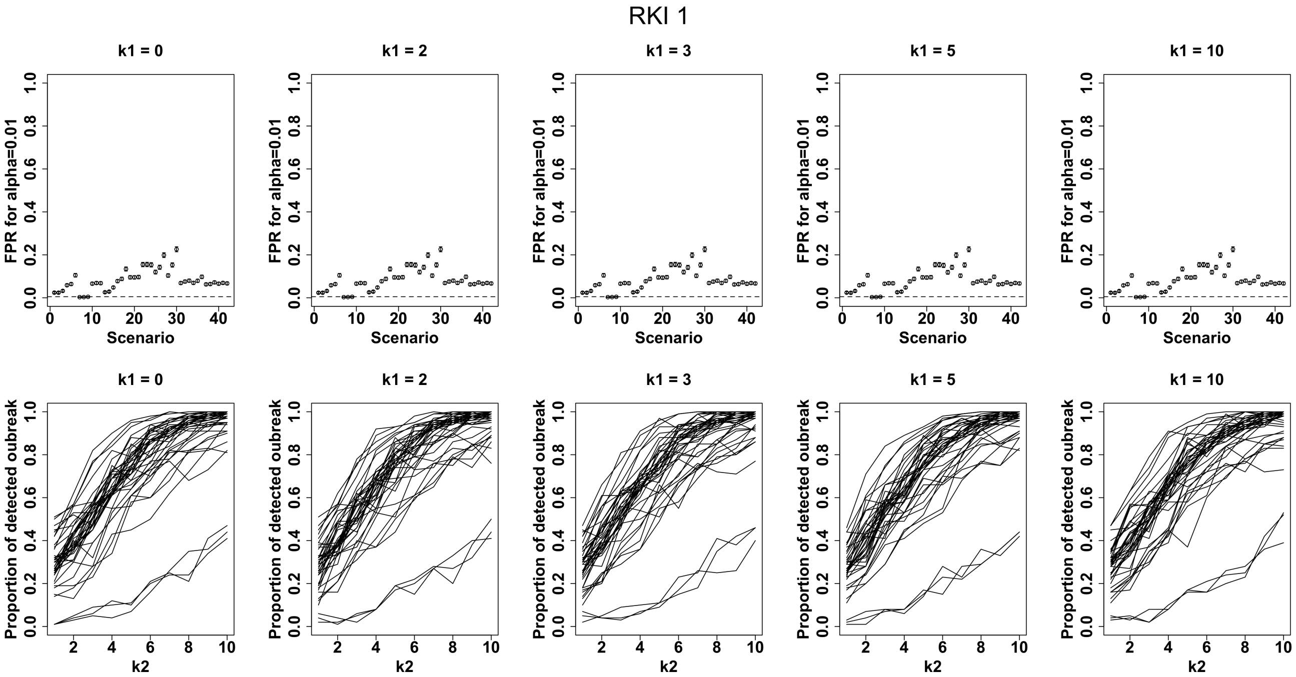

Figure 1: RKI 1 algorithm performances by increasing past outbreak amplitude $k_1 = 0$, 2, 3, 5 or 10 with (i) on the first row: false positive rate for 42 simulated scenarios, (ii) on the second row: probability of detection for 42 simulated scenarios (each curve corresponding to a scenario) by increasing current outbreak amplitude $k_2 = 1$ to 10.

Overall performances of RKI 1 algorithm

	FPR k1=0	FPR k1=2	FPR k1=3	FPR k1=5	FPR k1=10
1	0.02	0.02	0.02	0.02	0.02
2	0.02	0.02	0.02	0.02	0.02
3	0.03	0.03	0.03	0.03	0.03
4	0.06	0.06	0.06	0.06	0.06
5	0.06	0.06	0.06	0.06	0.06
6	0.10	0.10	0.10	0.10	0.10
7	0.00	0.00	0.00	0.00	0.00
8	0.00	0.00	0.00	0.00	0.00
9	0.00	0.00	0.00	0.00	0.00
10	0.07	0.07	0.07	0.07	0.07
11	0.07	0.07	0.07	0.07	0.07
12	0.07	0.07	0.07	0.07	0.07
13	0.03	0.03	0.03	0.03	0.02
14	0.03	0.03	0.03	0.03	0.03
15	0.05	0.05	0.05	0.05	0.05
16	0.08	0.08	0.08	0.08	0.08
17	0.09	0.09	0.09	0.09	0.09
18	0.13	0.13	0.13	0.13	0.13
19	0.10	0.10	0.10	0.10	0.10
20	0.10	0.09	0.09	0.09	0.09
21	0.10	0.10	0.10	0.10	0.10
22	0.15	0.15	0.15	0.15	0.15
23	0.16	0.15	0.16	0.15	0.15
24	0.15	0.15	0.15	0.15	0.15
25	0.12	0.12	0.12	0.12	0.12
26	0.14	0.14	0.14	0.14	0.14
27	0.20	0.20	0.20	0.20	0.20
28	0.10	0.10	0.10	0.10	0.10
29	0.15	0.15	0.15	0.15	0.15
30	0.23	0.23	0.23	0.23	0.23
31	0.07	0.07	0.07	0.07	0.07
32	0.08	0.08	0.08	0.08	0.08
33	0.08	0.08	0.08	0.08	0.08
34	0.07	0.07	0.07	0.07	0.07
35	0.08	0.08	0.08	0.08	0.08
36	0.10	0.10	0.10	0.10	0.10
37	0.06	0.06	0.06	0.06	0.06
38	0.06	0.06	0.06	0.06	0.06
39	0.07	0.07	0.07	0.07	0.07
40	0.07	0.07	0.07	0.06	0.07
41	0.07	0.07	0.07	0.07	0.07
42	0.07	0.07	0.07	0.07	0.07

Table 1: FPR according to each scenario and each k1 value

	k2=1	k2=2	k2=3	k2=4	k2=5	k2=6	k2=7	k2=8	k2=9	k2=10
1	0.20	0.30	0.28	0.34	0.58	0.60	0.72	0.81	0.88	0.91
2	0.19	0.19	0.23	0.39	0.61	0.60	0.68	0.82	0.83	0.95
3	0.15	0.13	0.26	0.43	0.45	0.50	0.62	0.72	0.83	0.81
4	0.27	0.37	0.53	0.62	0.78	0.85	0.91	0.98	0.97	1.00
5	0.26	0.35	0.54	0.63	0.66	0.74	0.86	0.92	0.93	0.98
6	0.30	0.37	0.39	0.50	0.61	0.70	0.81	0.82	0.87	0.90
7	0.01	0.05	0.09	0.10	0.11	0.20	0.24	0.24	0.34	0.41
8	0.01	0.04	0.06	0.12	0.10	0.21	0.25	0.21	0.41	0.47
9	0.01	0.03	0.05	0.04	0.07	0.16	0.26	0.35	0.36	0.44
10	0.31	0.38	0.45	0.63	0.85	0.79	0.89	0.94	0.99	0.99
11	0.30	0.31	0.51	0.69	0.80	0.91	0.95	0.97	0.97	1.00
12	0.26	0.31	0.50	0.63	0.75	0.87	0.90	0.96	0.96	0.99
13	0.14	0.18	0.28	0.53	0.64	0.77	0.78	0.88	0.99	0.97
14	0.18	0.23	0.39	0.50	0.61	0.68	0.82	0.87	0.94	0.95
15	0.25	0.40	0.32	0.55	0.56	0.72	0.83	0.88	0.95	0.95
16	0.28	0.44	0.55	0.64	0.74	0.94	0.91	0.97	0.99	1.00
17	0.33	0.30	0.48	0.75	0.68	0.82	0.88	0.93	0.95	0.97
18	0.45	0.47	0.57	0.55	0.74	0.70	0.78	0.86	0.91	0.91
19	0.26	0.47	0.62	0.72	0.89	0.94	0.96	0.99	1.00	1.00
20	0.37	0.44	0.58	0.78	0.84	0.92	0.96	0.99	1.00	0.99
21	0.28	0.43	0.56	0.73	0.77	0.92	0.95	0.99	0.99	1.00
22	0.42	0.62	0.82	0.90	0.96	0.98	0.99	1.00	1.00	1.00
23	0.42	0.57	0.76	0.86	0.95	0.96	1.00	0.99	1.00	1.00
24	0.51	0.56	0.70	0.82	0.79	0.91	0.97	0.97	0.98	1.00
25	0.50	0.57	0.77	0.84	0.93	0.97	1.00	0.99	0.99	1.00
26	0.38	0.49	0.58	0.72	0.88	0.87	0.89	0.95	0.96	0.98
27	0.44	0.53	0.53	0.63	0.64	0.74	0.82	0.81	0.82	0.86
28	0.36	0.56	0.58	0.69	0.77	0.82	0.93	0.94	0.96	0.99
29	0.31	0.44	0.50	0.69	0.71	0.80	0.84	0.95	0.93	0.95
30	0.47	0.52	0.47	0.61	0.68	0.73	0.77	0.68	0.73	0.82
31	0.21	0.41	0.47	0.66	0.79	0.89	0.91	0.98	0.96	0.98
32	0.24	0.44	0.56	0.65	0.71	0.92	0.88	0.96	0.97	0.97
33	0.26	0.42	0.56	0.62	0.75	0.87	0.86	0.98	0.97	0.99
34	0.27	0.39	0.45	0.61	0.83	0.90	0.94	0.94	1.00	0.99
35	0.30	0.36	0.51	0.72	0.73	0.86	0.92	0.97	0.98	0.99
36	0.31	0.34	0.47	0.61	0.73	0.76	0.92	0.93	0.94	0.94
37	0.26	0.43	0.54	0.69	0.75	0.79	0.96	0.98	0.98	0.99
38	0.32	0.36	0.57	0.72	0.79	0.87	0.93	0.95	0.98	0.99
39	0.24	0.38	0.57	0.61	0.81	0.86	0.89	0.96	0.98	1.00
40	0.32	0.38	0.45	0.71	0.71	0.86	0.91	0.98	1.00	1.00
41	0.32	0.36	0.45	0.62	0.74	0.91	0.91	0.97	0.99	0.97
42	0.25	0.36	0.48	0.62	0.71	0.87	0.93	0.91	0.91	1.00

Table 2: POD according to each scenario and each k2 value, k1 = 0

	k2=1	k2=2	k2=3	k2=4	k2=5	k2=6	k2=7	k2=8	k2=9	k2=10
1	0.12	0.21	0.40	0.37	0.50	0.59	0.70	0.80	0.84	0.91
2	0.18	0.24	0.31	0.41	0.53	0.61	0.67	0.78	0.78	0.89
3	0.13	0.21	0.35	0.37	0.44	0.60	0.65	0.79	0.74	0.86
4	0.24	0.33	0.43	0.69	0.77	0.86	0.94	0.96	0.96	1.00
5	0.32	0.38	0.57	0.59	0.67	0.84	0.86	0.92	0.95	0.96
6	0.17	0.40	0.50	0.55	0.57	0.61	0.76	0.78	0.84	0.88
7	0.06	0.04	0.02	0.08	0.19	0.15	0.27	0.33	0.40	0.41
8	0.02	0.02	0.05	0.08	0.19	0.22	0.28	0.27	0.32	0.44
9	0.04	0.01	0.06	0.08	0.17	0.20	0.27	0.20	0.38	0.50
10	0.20	0.38	0.56	0.68	0.75	0.94	0.94	0.96	0.98	0.99
11	0.31	0.35	0.59	0.53	0.77	0.87	0.92	0.98	1.00	1.00
12	0.19	0.48	0.44	0.59	0.81	0.84	0.96	0.96	0.94	0.96
13	0.16	0.16	0.34	0.51	0.66	0.76	0.85	0.90	0.98	0.96
14	0.10	0.34	0.39	0.53	0.51	0.73	0.86	0.89	0.98	0.97
15	0.29	0.34	0.37	0.55	0.80	0.73	0.74	0.86	0.88	0.93
16	0.37	0.37	0.46	0.65	0.81	0.89	0.92	0.94	1.00	1.00
17	0.26	0.29	0.60	0.66	0.77	0.82	0.89	0.95	0.96	0.97
18	0.36	0.49	0.62	0.55	0.75	0.79	0.77	0.83	0.82	0.89
19	0.32	0.48	0.58	0.82	0.89	0.94	0.96	0.99	0.99	1.00
20	0.27	0.46	0.65	0.80	0.86	0.89	0.96	0.95	1.00	1.00
21	0.33	0.35	0.61	0.69	0.86	0.91	0.92	0.99	0.98	1.00
22	0.43	0.62	0.75	0.92	0.93	0.95	0.99	1.00	1.00	1.00
23	0.47	0.56	0.78	0.86	0.91	0.98	1.00	0.99	1.00	1.00
24	0.51	0.57	0.76	0.83	0.90	0.93	0.96	1.00	0.99	0.99
25	0.39	0.55	0.70	0.89	0.93	0.95	0.99	0.99	1.00	1.00
26	0.32	0.52	0.60	0.70	0.80	0.88	0.93	0.91	0.98	0.97
27	0.39	0.37	0.63	0.72	0.76	0.79	0.81	0.90	0.85	0.83
28	0.29	0.46	0.51	0.70	0.83	0.91	0.91	0.97	0.96	0.99
29	0.41	0.54	0.56	0.61	0.74	0.74	0.89	0.88	0.93	0.92
30	0.45	0.57	0.56	0.68	0.69	0.70	0.76	0.75	0.85	0.76
31	0.28	0.37	0.50	0.73	0.81	0.86	0.96	0.95	0.98	1.00
32	0.38	0.39	0.59	0.61	0.78	0.83	0.91	0.96	1.00	0.97
33	0.30	0.38	0.53	0.72	0.78	0.84	0.85	0.96	0.97	0.98
34	0.30	0.39	0.53	0.68	0.74	0.84	0.94	0.97	0.98	0.99
35	0.24	0.41	0.51	0.60	0.83	0.80	0.95	0.97	0.99	1.00
36	0.31	0.38	0.46	0.67	0.63	0.81	0.91	0.98	0.94	0.95
37	0.24	0.39	0.53	0.68	0.88	0.79	0.94	0.94	1.00	1.00
38	0.19	0.35	0.51	0.64	0.74	0.84	0.95	0.95	0.99	0.99
39	0.25	0.45	0.48	0.65	0.75	0.88	0.93	0.96	0.97	0.99
40	0.29	0.49	0.49	0.62	0.85	0.87	0.97	0.98	0.97	1.00
41	0.33	0.33	0.60	0.68	0.67	0.93	0.94	0.94	0.98	1.00
42	0.26	0.38	0.50	0.67	0.80	0.88	0.92	0.95	0.94	0.99

Table 3: POD according to each scenario and each k2 value, k1 = 2

	k2=1	k2=2	k2=3	k2=4	k2=5	k2=6	k2=7	k2=8	k2=9	k2=10
1	0.16	0.21	0.34	0.40	0.66	0.55	0.74	0.83	0.87	0.93
2	0.14	0.25	0.29	0.36	0.49	0.67	0.74	0.76	0.81	0.86
3	0.13	0.24	0.33	0.36	0.55	0.58	0.69	0.79	0.80	0.86
4	0.18	0.37	0.47	0.64	0.71	0.89	0.91	0.93	0.99	1.00
5	0.30	0.33	0.46	0.71	0.71	0.79	0.84	0.96	0.95	1.00
6	0.26	0.39	0.40	0.54	0.51	0.65	0.75	0.78	0.83	0.94
7	0.07	0.04	0.04	0.06	0.11	0.15	0.18	0.35	0.42	0.46
8	0.05	0.04	0.03	0.07	0.09	0.17	0.26	0.25	0.26	0.40
9	0.02	0.05	0.09	0.10	0.11	0.23	0.25	0.41	0.38	0.46
10	0.25	0.40	0.53	0.69	0.73	0.94	0.95	0.96	0.97	1.00
11	0.26	0.36	0.48	0.71	0.82	0.81	0.90	0.96	0.95	0.98
12	0.29	0.28	0.51	0.60	0.76	0.82	0.89	0.96	0.97	0.99
13	0.10	0.24	0.36	0.46	0.63	0.70	0.85	0.85	0.94	0.99
14	0.17	0.20	0.39	0.47	0.60	0.71	0.81	0.85	0.91	0.92
15	0.18	0.21	0.48	0.50	0.56	0.67	0.73	0.85	0.95	0.91
16	0.32	0.37	0.61	0.70	0.74	0.88	0.92	0.97	0.97	0.98
17	0.26	0.40	0.53	0.65	0.74	0.78	0.92	0.92	0.94	0.98
18	0.32	0.34	0.50	0.58	0.63	0.80	0.79	0.87	0.80	0.88
19	0.33	0.56	0.62	0.82	0.90	0.91	0.99	0.99	1.00	1.00
20	0.35	0.43	0.64	0.74	0.88	0.88	0.97	1.00	0.99	1.00
21	0.25	0.49	0.63	0.68	0.86	0.94	0.93	0.98	0.99	1.00
22	0.47	0.66	0.77	0.91	0.96	0.99	1.00	1.00	1.00	1.00
23	0.39	0.60	0.81	0.84	0.97	0.92	0.99	1.00	1.00	1.00
24	0.42	0.56	0.67	0.82	0.93	0.93	0.96	0.94	1.00	1.00
25	0.36	0.54	0.72	0.89	0.94	0.99	1.00	0.99	0.99	1.00
26	0.44	0.49	0.62	0.63	0.78	0.88	0.89	0.95	0.95	1.00
27	0.45	0.41	0.54	0.64	0.70	0.78	0.73	0.81	0.85	0.88
28	0.30	0.46	0.60	0.70	0.77	0.92	0.92	0.99	0.97	0.99
29	0.35	0.44	0.63	0.65	0.76	0.81	0.79	0.87	0.92	0.92
30	0.42	0.61	0.61	0.66	0.62	0.68	0.76	0.72	0.71	0.77
31	0.31	0.40	0.51	0.62	0.83	0.93	0.95	0.96	0.97	0.99
32	0.24	0.46	0.52	0.72	0.81	0.86	0.92	0.95	0.96	0.99
33	0.30	0.42	0.56	0.54	0.68	0.84	0.87	0.91	0.93	0.97
34	0.26	0.38	0.47	0.64	0.81	0.91	0.93	0.99	0.99	0.99
35	0.30	0.47	0.60	0.68	0.84	0.87	0.94	0.97	0.94	0.98
36	0.37	0.44	0.57	0.64	0.77	0.85	0.86	0.95	0.96	0.97
37	0.18	0.40	0.53	0.64	0.71	0.89	0.94	0.95	0.97	1.00
38	0.25	0.35	0.49	0.68	0.76	0.91	0.99	0.93	1.00	1.00
39	0.23	0.38	0.52	0.66	0.73	0.88	0.88	0.92	1.00	0.99
40	0.29	0.37	0.57	0.69	0.67	0.86	0.91	1.00	0.98	0.99
41	0.22	0.35	0.55	0.67	0.71	0.86	0.94	0.96	0.99	1.00
42	0.31	0.47	0.51	0.64	0.81	0.86	0.93	0.95	1.00	0.99

Table 4: POD according to each scenario and each k2 value, k1 = 3

	k2=1	k2=2	k2=3	k2=4	k2=5	k2=6	k2=7	k2=8	k2=9	k2=10
1	0.13	0.20	0.29	0.45	0.50	0.55	0.80	0.92	0.85	0.91
2	0.18	0.20	0.29	0.44	0.52	0.70	0.69	0.76	0.84	0.90
3	0.19	0.24	0.38	0.41	0.48	0.53	0.68	0.77	0.75	0.88
4	0.30	0.32	0.45	0.61	0.75	0.85	0.90	0.97	0.99	1.00
5	0.27	0.33	0.52	0.53	0.70	0.80	0.83	0.92	0.96	0.98
6	0.29	0.28	0.47	0.51	0.55	0.67	0.74	0.84	0.86	0.91
7	0.01	0.01	0.08	0.08	0.17	0.15	0.28	0.29	0.36	0.44
8	0.02	0.04	0.07	0.08	0.15	0.23	0.22	0.29	0.36	0.44
9	0.03	0.07	0.08	0.06	0.14	0.28	0.23	0.36	0.33	0.42
10	0.22	0.34	0.56	0.63	0.76	0.89	0.95	0.93	1.00	1.00
11	0.25	0.36	0.54	0.70	0.83	0.89	0.95	0.96	1.00	0.98
12	0.24	0.46	0.56	0.65	0.75	0.92	0.91	0.98	0.98	0.98
13	0.17	0.25	0.40	0.53	0.58	0.70	0.83	0.91	0.95	0.97
14	0.11	0.25	0.43	0.53	0.69	0.67	0.79	0.95	0.94	0.99
15	0.25	0.28	0.49	0.49	0.63	0.72	0.77	0.87	0.92	0.96
16	0.27	0.37	0.59	0.70	0.82	0.87	0.88	0.98	0.98	1.00
17	0.26	0.37	0.63	0.61	0.77	0.84	0.88	0.91	0.96	0.97
18	0.32	0.48	0.43	0.59	0.62	0.73	0.81	0.85	0.85	0.88
19	0.23	0.59	0.57	0.80	0.88	0.97	0.98	0.98	0.99	1.00
20	0.37	0.41	0.59	0.76	0.87	0.93	0.96	0.99	1.00	1.00
21	0.27	0.36	0.55	0.73	0.85	0.93	0.95	0.97	0.99	1.00
22	0.46	0.71	0.84	0.89	0.93	0.98	0.99	1.00	1.00	1.00
23	0.38	0.61	0.74	0.86	0.92	0.96	0.99	1.00	1.00	0.99
24	0.44	0.60	0.67	0.78	0.87	0.96	0.98	0.98	1.00	0.99
25	0.38	0.55	0.75	0.85	0.90	0.96	0.99	1.00	1.00	1.00
26	0.34	0.48	0.66	0.68	0.85	0.87	0.89	0.94	0.94	0.98
27	0.35	0.49	0.58	0.63	0.73	0.75	0.81	0.88	0.84	0.82
28	0.35	0.54	0.56	0.69	0.75	0.85	0.88	0.91	1.00	1.00
29	0.44	0.43	0.56	0.58	0.73	0.85	0.90	0.89	0.94	0.93
30	0.39	0.54	0.59	0.53	0.66	0.66	0.73	0.76	0.75	0.83
31	0.30	0.30	0.58	0.61	0.73	0.83	0.91	0.97	0.99	0.99
32	0.30	0.46	0.59	0.60	0.80	0.79	0.89	0.93	1.00	0.98
33	0.31	0.43	0.55	0.62	0.80	0.83	0.90	0.95	0.96	0.99
34	0.25	0.40	0.51	0.66	0.89	0.81	0.93	0.97	0.96	0.99
35	0.29	0.44	0.51	0.63	0.76	0.91	0.97	0.94	0.99	0.97
36	0.26	0.39	0.66	0.70	0.75	0.82	0.87	0.93	0.95	0.96
37	0.25	0.36	0.51	0.64	0.79	0.90	0.94	0.96	0.97	0.99
38	0.25	0.43	0.47	0.58	0.85	0.92	0.97	0.93	0.98	1.00
39	0.23	0.28	0.57	0.58	0.76	0.88	0.88	0.95	0.97	1.00
40	0.29	0.44	0.53	0.69	0.74	0.83	0.93	0.96	0.99	1.00
41	0.26	0.34	0.53	0.64	0.73	0.82	0.91	0.96	0.98	1.00
42	0.26	0.42	0.41	0.71	0.79	0.86	0.91	0.94	0.98	0.97

Table 5: POD according to each scenario and each k2 value, k1 = 5

	k2=1	k2=2	k2=3	k2=4	k2=5	k2=6	k2=7	k2=8	k2=9	k2=10
1	0.23	0.26	0.30	0.43	0.56	0.61	0.84	0.78	0.82	0.88
2	0.13	0.22	0.38	0.45	0.62	0.65	0.69	0.80	0.88	0.91
3	0.16	0.17	0.33	0.45	0.37	0.60	0.70	0.78	0.83	0.83
4	0.20	0.37	0.47	0.63	0.71	0.86	0.91	0.97	0.98	0.99
5	0.22	0.35	0.37	0.46	0.71	0.85	0.89	0.87	0.95	0.94
6	0.25	0.33	0.47	0.58	0.58	0.68	0.76	0.72	0.85	0.88
7	0.05	0.03	0.08	0.08	0.17	0.16	0.20	0.23	0.42	0.52
8	0.04	0.05	0.02	0.08	0.17	0.21	0.24	0.25	0.38	0.53
9	0.03	0.04	0.02	0.10	0.16	0.16	0.26	0.28	0.36	0.39
10	0.28	0.36	0.58	0.59	0.76	0.85	0.90	0.94	0.95	0.98
11	0.25	0.34	0.50	0.65	0.75	0.82	0.93	0.93	0.97	0.99
12	0.18	0.24	0.58	0.61	0.77	0.81	0.88	0.93	1.00	0.99
13	0.11	0.21	0.35	0.39	0.64	0.74	0.79	0.90	0.95	1.00
14	0.16	0.23	0.29	0.44	0.71	0.77	0.84	0.90	0.97	0.95
15	0.16	0.22	0.35	0.42	0.59	0.74	0.84	0.87	0.89	0.90
16	0.30	0.39	0.56	0.66	0.79	0.83	0.89	0.98	0.99	1.00
17	0.31	0.41	0.56	0.64	0.77	0.88	0.89	0.94	0.96	0.98
18	0.29	0.44	0.50	0.59	0.68	0.72	0.82	0.87	0.85	0.87
19	0.32	0.55	0.62	0.72	0.93	0.95	0.97	1.00	1.00	0.99
20	0.35	0.49	0.56	0.68	0.87	0.89	0.94	1.00	1.00	0.99
21	0.30	0.56	0.57	0.71	0.85	0.94	0.94	1.00	0.98	1.00
22	0.47	0.63	0.74	0.90	0.95	0.99	1.00	1.00	0.99	1.00
23	0.47	0.63	0.79	0.91	0.89	0.97	0.98	0.97	1.00	1.00
24	0.34	0.56	0.67	0.78	0.90	0.89	0.96	0.97	1.00	1.00
25	0.47	0.59	0.71	0.88	0.93	0.95	0.98	1.00	1.00	1.00
26	0.45	0.46	0.56	0.67	0.79	0.81	0.95	0.94	0.94	0.93
27	0.36	0.49	0.44	0.69	0.65	0.72	0.75	0.76	0.85	0.84
28	0.36	0.48	0.63	0.61	0.80	0.89	0.92	0.95	1.00	0.99
29	0.40	0.46	0.52	0.70	0.76	0.83	0.86	0.92	0.94	0.99
30	0.35	0.57	0.56	0.54	0.63	0.67	0.74	0.77	0.72	0.73
31	0.31	0.35	0.53	0.70	0.81	0.87	0.92	0.96	1.00	0.99
32	0.28	0.43	0.50	0.69	0.72	0.86	0.86	0.93	0.96	0.98
33	0.30	0.43	0.51	0.71	0.71	0.83	0.89	0.92	0.93	1.00
34	0.30	0.33	0.49	0.64	0.88	0.83	0.92	0.96	0.99	0.99
35	0.28	0.40	0.56	0.69	0.81	0.88	0.88	0.96	0.98	0.99
36	0.35	0.39	0.55	0.59	0.70	0.81	0.89	0.93	0.95	0.98
37	0.29	0.38	0.58	0.65	0.82	0.86	0.90	0.96	0.95	0.99
38	0.24	0.36	0.49	0.65	0.78	0.86	0.99	0.93	0.98	0.96
39	0.24	0.44	0.41	0.66	0.76	0.83	0.93	0.95	0.97	1.00
40	0.28	0.27	0.50	0.69	0.85	0.81	0.94	0.92	0.99	0.98
41	0.32	0.40	0.50	0.59	0.86	0.79	0.91	0.92	0.97	1.00
42	0.32	0.36	0.46	0.67	0.74	0.83	0.91	0.95	0.97	0.99

Table 6: POD according to each scenario and each k2 value, k1 = 10
