# Supplementary material for: Evaluation and comparison of statistical methods for early temporal detection of outbreaks: A simulation-based study
Source: PLoS One. 2017 Jul 17;12(7):e0181227. doi: 10.1371/journal.pone.0181227 (PMC5513450; doi:10.1371/journal.pone.0181227)
Supplement: S15 Appendix — (PDF) [file pone.0181227.s015.pdf]

Evaluation and Comparison of Statistical Methods for Early Temporal Detection of Outbreaks: a Simulation-Based Study

Appendix S15: Overall performances of RKI 2 algorithm

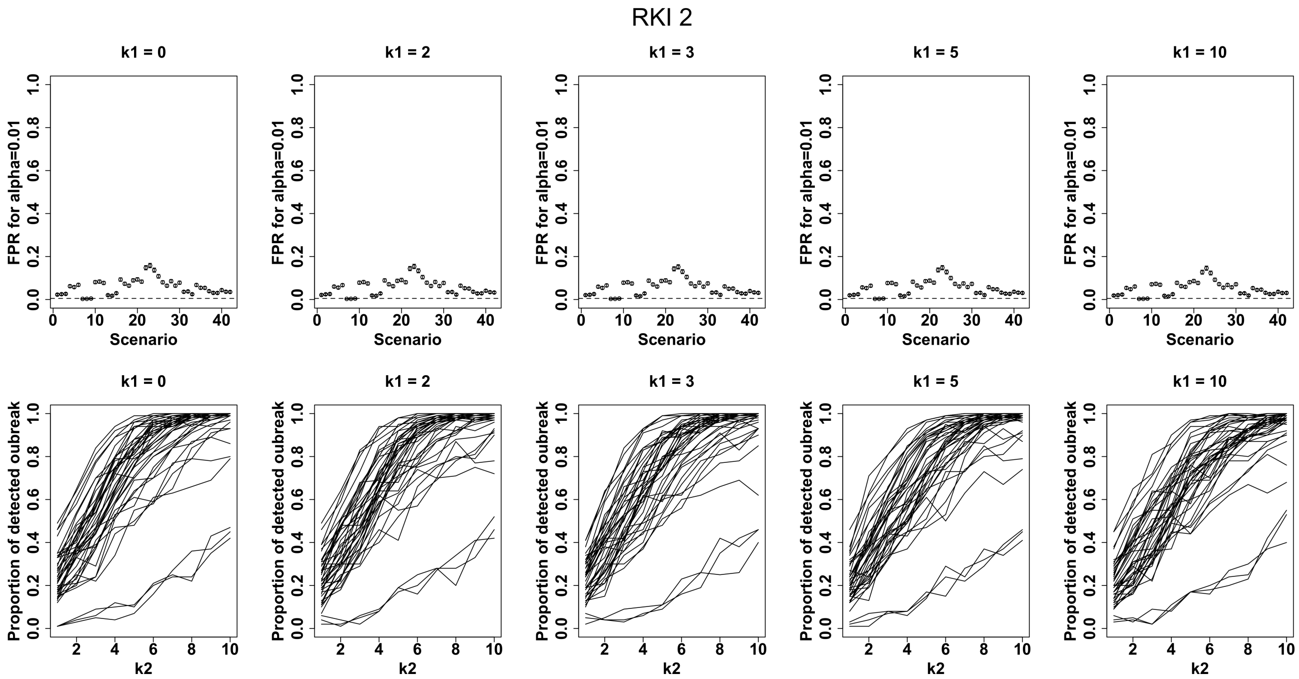

Figure 1: RKI 2 algorithm performances by increasing past outbreak amplitude $k_1 = 0$, 2, 3, 5 or 10 with (i) on the first row: false positive rate for 42 simulated scenarios, (ii) on the second row: probability of detection for 42 simulated scenarios (each curve corresponding to a scenario) by increasing current outbreak amplitude $k_2 = 1$ to 10.

Overall performances of RKI 2 algorithm

	FPR k1=0	FPR k1=2	FPR k1=3	FPR k1=5	FPR k1=10
1	0.02	0.02	0.02	0.02	0.02
2	0.02	0.02	0.02	0.02	0.02
3	0.03	0.03	0.03	0.02	0.02
4	0.06	0.06	0.06	0.06	0.05
5	0.06	0.06	0.05	0.05	0.05
6	0.07	0.07	0.07	0.06	0.06
7	0.00	0.00	0.00	0.00	0.00
8	0.00	0.00	0.00	0.00	0.00
9	0.00	0.00	0.00	0.00	0.00
10	0.08	0.08	0.08	0.08	0.07
11	0.08	0.08	0.08	0.08	0.07
12	0.08	0.07	0.07	0.07	0.07
13	0.02	0.02	0.02	0.02	0.02
14	0.02	0.02	0.02	0.02	0.01
15	0.03	0.03	0.03	0.03	0.02
16	0.09	0.09	0.09	0.08	0.08
17	0.07	0.07	0.07	0.07	0.06
18	0.06	0.06	0.06	0.06	0.06
19	0.09	0.09	0.09	0.09	0.08
20	0.09	0.09	0.09	0.09	0.09
21	0.08	0.08	0.08	0.08	0.08
22	0.15	0.14	0.14	0.14	0.13
23	0.16	0.15	0.15	0.15	0.15
24	0.14	0.13	0.13	0.13	0.12
25	0.11	0.10	0.10	0.10	0.09
26	0.08	0.08	0.07	0.07	0.07
27	0.06	0.06	0.06	0.06	0.06
28	0.09	0.08	0.08	0.07	0.07
29	0.06	0.06	0.06	0.06	0.06
30	0.08	0.08	0.07	0.07	0.07
31	0.04	0.03	0.03	0.03	0.03
32	0.04	0.03	0.03	0.03	0.03
33	0.02	0.02	0.02	0.02	0.02
34	0.07	0.06	0.06	0.06	0.05
35	0.05	0.05	0.05	0.05	0.04
36	0.05	0.05	0.05	0.05	0.05
37	0.04	0.04	0.03	0.03	0.03
38	0.03	0.03	0.03	0.03	0.03
39	0.03	0.03	0.03	0.03	0.03
40	0.04	0.04	0.04	0.04	0.04
41	0.04	0.03	0.03	0.03	0.03
42	0.04	0.03	0.03	0.03	0.03

Table 1: FPR according to each scenario and each k1 value

	k2=1	k2=2	k2=3	k2=4	k2=5	k2=6	k2=7	k2=8	k2=9	k2=10
1	0.17	0.25	0.22	0.34	0.55	0.58	0.72	0.79	0.91	0.93
2	0.20	0.21	0.24	0.51	0.64	0.70	0.76	0.87	0.89	0.96
3	0.15	0.19	0.24	0.44	0.50	0.59	0.65	0.84	0.89	0.86
4	0.27	0.44	0.58	0.78	0.81	0.92	0.96	1.00	0.97	1.00
5	0.35	0.40	0.59	0.79	0.79	0.84	0.92	0.99	0.99	1.00
6	0.28	0.37	0.44	0.62	0.75	0.82	0.87	0.92	0.99	0.99
7	0.01	0.05	0.09	0.10	0.11	0.20	0.24	0.24	0.35	0.42
8	0.01	0.04	0.06	0.12	0.10	0.21	0.25	0.22	0.43	0.47
9	0.01	0.03	0.05	0.04	0.07	0.16	0.27	0.36	0.37	0.45
10	0.33	0.46	0.57	0.73	0.88	0.92	0.94	0.97	1.00	1.00
11	0.33	0.41	0.66	0.85	0.94	0.94	0.99	0.99	0.97	1.00
12	0.30	0.43	0.64	0.75	0.85	0.95	0.97	1.00	0.97	1.00
13	0.12	0.24	0.37	0.58	0.70	0.80	0.85	0.92	0.99	0.99
14	0.16	0.20	0.41	0.51	0.63	0.73	0.84	0.91	0.95	0.97
15	0.17	0.33	0.29	0.53	0.57	0.75	0.84	0.93	0.97	1.00
16	0.35	0.54	0.70	0.79	0.93	0.99	0.98	1.00	1.00	1.00
17	0.34	0.38	0.56	0.80	0.86	0.94	0.94	0.99	0.98	1.00
18	0.33	0.36	0.47	0.52	0.69	0.70	0.84	0.86	0.93	0.93
19	0.27	0.44	0.66	0.80	0.96	0.97	0.99	1.00	1.00	1.00
20	0.33	0.49	0.60	0.80	0.88	0.97	0.99	0.98	1.00	1.00
21	0.21	0.44	0.54	0.75	0.84	0.97	0.95	1.00	1.00	1.00
22	0.46	0.65	0.84	0.94	0.99	0.99	1.00	1.00	1.00	1.00
23	0.49	0.64	0.81	0.92	0.96	0.99	1.00	1.00	1.00	1.00
24	0.43	0.61	0.78	0.89	0.97	1.00	1.00	1.00	1.00	1.00
25	0.46	0.58	0.77	0.89	0.93	0.98	1.00	1.00	1.00	1.00
26	0.26	0.42	0.57	0.70	0.85	0.91	0.95	0.98	1.00	0.99
27	0.23	0.36	0.38	0.55	0.61	0.59	0.77	0.79	0.78	0.80
28	0.29	0.53	0.70	0.74	0.82	0.95	0.97	0.99	0.99	1.00
29	0.21	0.37	0.47	0.72	0.70	0.80	0.86	0.95	0.94	0.99
30	0.22	0.35	0.39	0.47	0.48	0.61	0.63	0.66	0.69	0.79
31	0.13	0.36	0.44	0.64	0.84	0.96	0.97	0.99	0.99	1.00
32	0.18	0.34	0.46	0.62	0.75	0.96	0.94	0.98	0.99	1.00
33	0.14	0.21	0.37	0.58	0.71	0.82	0.92	0.96	0.97	0.99
34	0.24	0.48	0.54	0.74	0.94	0.96	0.98	1.00	1.00	1.00
35	0.28	0.37	0.57	0.72	0.88	0.90	0.96	0.99	0.99	1.00
36	0.19	0.29	0.45	0.59	0.76	0.86	0.92	0.95	0.99	0.99
37	0.14	0.39	0.49	0.67	0.80	0.87	0.96	0.98	0.99	1.00
38	0.18	0.23	0.47	0.63	0.78	0.87	0.96	0.97	1.00	1.00
39	0.16	0.34	0.51	0.65	0.78	0.86	0.93	0.99	1.00	1.00
40	0.24	0.38	0.53	0.75	0.81	0.93	0.97	1.00	0.99	1.00
41	0.23	0.33	0.44	0.65	0.83	0.89	0.97	1.00	1.00	1.00
42	0.14	0.26	0.38	0.58	0.76	0.90	0.98	0.94	0.99	1.00

Table 2: POD according to each scenario and each k2 value, k1 = 0

	k2=1	k2=2	k2=3	k2=4	k2=5	k2=6	k2=7	k2=8	k2=9	k2=10
1	0.13	0.18	0.35	0.40	0.52	0.62	0.69	0.78	0.83	0.92
2	0.15	0.25	0.29	0.43	0.62	0.70	0.76	0.79	0.81	0.93
3	0.11	0.21	0.34	0.46	0.41	0.66	0.75	0.80	0.79	0.90
4	0.32	0.43	0.55	0.68	0.86	0.90	0.96	1.00	0.99	1.00
5	0.29	0.43	0.56	0.73	0.82	0.91	0.92	0.98	0.98	0.99
6	0.16	0.41	0.51	0.64	0.64	0.81	0.88	0.91	0.97	0.97
7	0.06	0.04	0.02	0.08	0.19	0.16	0.27	0.34	0.41	0.42
8	0.02	0.02	0.05	0.08	0.19	0.25	0.28	0.28	0.33	0.46
9	0.04	0.01	0.06	0.09	0.17	0.20	0.28	0.20	0.40	0.52
10	0.31	0.50	0.66	0.80	0.83	0.95	0.96	0.98	1.00	1.00
11	0.37	0.46	0.68	0.69	0.92	0.94	0.97	0.98	1.00	1.00
12	0.25	0.51	0.57	0.71	0.89	0.96	0.98	0.99	0.97	0.99
13	0.17	0.19	0.36	0.56	0.77	0.79	0.87	0.90	0.98	0.98
14	0.07	0.24	0.37	0.52	0.58	0.76	0.87	0.92	0.98	0.98
15	0.22	0.30	0.30	0.53	0.76	0.75	0.78	0.94	0.92	0.96
16	0.39	0.51	0.67	0.80	0.95	0.96	0.99	1.00	1.00	1.00
17	0.31	0.35	0.64	0.78	0.88	0.89	0.96	0.98	1.00	0.99
18	0.23	0.36	0.45	0.57	0.71	0.76	0.81	0.88	0.89	0.91
19	0.32	0.48	0.61	0.85	0.92	0.98	1.00	1.00	1.00	1.00
20	0.29	0.47	0.65	0.83	0.88	0.97	0.99	0.97	1.00	1.00
21	0.30	0.30	0.56	0.72	0.83	0.91	0.98	0.99	0.99	1.00
22	0.39	0.62	0.80	0.94	0.94	1.00	1.00	1.00	1.00	1.00
23	0.46	0.59	0.83	0.90	0.98	1.00	1.00	1.00	1.00	1.00
24	0.49	0.64	0.82	0.88	0.94	0.99	1.00	1.00	1.00	1.00
25	0.36	0.56	0.73	0.93	0.98	0.98	1.00	1.00	1.00	1.00
26	0.26	0.48	0.59	0.70	0.82	0.93	0.96	0.97	1.00	0.99
27	0.18	0.23	0.42	0.59	0.63	0.74	0.70	0.87	0.77	0.78
28	0.27	0.46	0.52	0.74	0.88	0.94	0.99	0.98	0.99	1.00
29	0.26	0.39	0.47	0.58	0.70	0.77	0.93	0.92	0.95	0.97
30	0.27	0.30	0.37	0.62	0.55	0.57	0.70	0.71	0.75	0.72
31	0.20	0.33	0.44	0.69	0.79	0.93	0.97	1.00	1.00	1.00
32	0.18	0.34	0.56	0.64	0.86	0.89	0.93	0.98	0.97	0.98
33	0.10	0.23	0.40	0.57	0.74	0.80	0.90	0.97	0.99	0.99
34	0.32	0.48	0.63	0.82	0.87	0.95	0.98	1.00	1.00	1.00
35	0.20	0.38	0.47	0.65	0.82	0.92	0.98	0.99	1.00	1.00
36	0.22	0.31	0.46	0.68	0.68	0.85	0.94	0.97	0.96	0.98
37	0.19	0.29	0.42	0.63	0.85	0.87	0.95	0.98	1.00	1.00
38	0.12	0.30	0.46	0.65	0.78	0.91	0.97	0.99	1.00	1.00
39	0.11	0.30	0.37	0.65	0.76	0.89	0.96	0.97	0.99	0.99
40	0.22	0.48	0.48	0.67	0.89	0.95	0.98	0.98	0.99	1.00
41	0.21	0.22	0.52	0.61	0.74	0.94	0.99	0.98	1.00	1.00
42	0.19	0.28	0.42	0.65	0.71	0.89	0.96	0.98	0.98	0.99

Table 3: POD according to each scenario and each k2 value, k1 = 2

	k2=1	k2=2	k2=3	k2=4	k2=5	k2=6	k2=7	k2=8	k2=9	k2=10
1	0.19	0.21	0.34	0.40	0.62	0.62	0.75	0.86	0.89	0.93
2	0.10	0.30	0.30	0.37	0.53	0.67	0.78	0.81	0.86	0.90
3	0.11	0.23	0.28	0.38	0.51	0.56	0.72	0.83	0.86	0.93
4	0.24	0.43	0.53	0.72	0.77	0.95	0.97	0.97	0.98	0.99
5	0.30	0.40	0.53	0.75	0.79	0.84	0.93	0.98	0.99	0.99
6	0.25	0.42	0.42	0.62	0.59	0.77	0.87	0.94	0.93	0.99
7	0.07	0.04	0.04	0.06	0.11	0.16	0.19	0.35	0.42	0.46
8	0.05	0.04	0.03	0.07	0.09	0.17	0.26	0.25	0.26	0.40
9	0.02	0.05	0.09	0.10	0.11	0.23	0.26	0.42	0.38	0.46
10	0.32	0.44	0.61	0.83	0.87	0.97	0.98	0.99	0.99	1.00
11	0.31	0.46	0.63	0.83	0.87	0.94	0.96	0.99	1.00	1.00
12	0.34	0.38	0.59	0.73	0.92	0.90	0.94	1.00	0.99	0.99
13	0.12	0.27	0.36	0.50	0.69	0.78	0.87	0.93	0.97	1.00
14	0.13	0.15	0.39	0.44	0.61	0.75	0.80	0.88	0.93	0.96
15	0.13	0.18	0.47	0.49	0.54	0.70	0.81	0.87	0.97	0.93
16	0.35	0.48	0.74	0.84	0.87	0.94	0.97	0.98	1.00	1.00
17	0.25	0.53	0.63	0.77	0.89	0.89	0.95	0.97	0.99	1.00
18	0.15	0.24	0.42	0.61	0.63	0.76	0.82	0.91	0.83	0.93
19	0.28	0.51	0.69	0.84	0.90	0.94	1.00	0.99	1.00	1.00
20	0.32	0.49	0.68	0.79	0.91	0.93	0.99	1.00	1.00	1.00
21	0.24	0.48	0.57	0.68	0.88	0.94	0.96	1.00	1.00	1.00
22	0.41	0.63	0.81	0.92	0.99	1.00	1.00	1.00	1.00	1.00
23	0.41	0.60	0.84	0.93	0.99	0.99	1.00	1.00	1.00	1.00
24	0.38	0.64	0.74	0.93	0.97	0.99	0.99	1.00	1.00	1.00
25	0.30	0.59	0.73	0.91	0.94	0.99	1.00	1.00	1.00	1.00
26	0.31	0.42	0.59	0.63	0.86	0.93	0.91	0.98	0.97	1.00
27	0.19	0.26	0.40	0.50	0.60	0.63	0.69	0.77	0.78	0.85
28	0.28	0.41	0.66	0.69	0.85	0.95	0.97	1.00	1.00	1.00
29	0.25	0.33	0.53	0.59	0.80	0.81	0.84	0.90	0.95	0.97
30	0.24	0.38	0.36	0.45	0.48	0.60	0.62	0.66	0.69	0.62
31	0.19	0.31	0.44	0.58	0.78	0.94	0.94	0.99	0.99	1.00
32	0.14	0.37	0.41	0.67	0.75	0.83	0.91	0.98	0.99	1.00
33	0.15	0.21	0.43	0.46	0.66	0.82	0.94	0.94	0.97	0.98
34	0.24	0.42	0.53	0.75	0.87	0.96	1.00	0.98	1.00	1.00
35	0.26	0.44	0.61	0.67	0.88	0.90	0.97	0.99	1.00	1.00
36	0.18	0.31	0.51	0.65	0.74	0.86	0.92	0.97	0.96	0.99
37	0.18	0.30	0.52	0.61	0.75	0.92	0.98	0.96	0.99	1.00
38	0.15	0.24	0.40	0.66	0.74	0.89	0.94	0.96	1.00	1.00
39	0.16	0.26	0.45	0.61	0.77	0.91	0.91	0.97	1.00	0.99
40	0.22	0.40	0.50	0.73	0.74	0.87	0.95	1.00	0.99	1.00
41	0.12	0.28	0.47	0.64	0.71	0.90	0.95	0.96	0.99	1.00
42	0.20	0.40	0.42	0.54	0.80	0.85	0.96	0.97	0.99	1.00

Table 4: POD according to each scenario and each k2 value, k1 = 3

	k2=1	k2=2	k2=3	k2=4	k2=5	k2=6	k2=7	k2=8	k2=9	k2=10
1	0.12	0.19	0.33	0.45	0.47	0.53	0.80	0.94	0.88	0.91
2	0.18	0.19	0.32	0.45	0.51	0.73	0.73	0.83	0.86	0.92
3	0.15	0.25	0.34	0.37	0.49	0.59	0.74	0.80	0.81	0.90
4	0.31	0.38	0.53	0.68	0.84	0.91	0.92	1.00	1.00	1.00
5	0.18	0.42	0.59	0.66	0.83	0.89	0.92	0.97	0.99	0.98
6	0.20	0.34	0.46	0.57	0.64	0.84	0.89	0.95	0.96	0.98
7	0.01	0.01	0.08	0.08	0.17	0.15	0.28	0.32	0.37	0.45
8	0.02	0.04	0.07	0.08	0.15	0.24	0.22	0.29	0.39	0.46
9	0.03	0.07	0.08	0.06	0.14	0.29	0.24	0.37	0.34	0.41
10	0.24	0.40	0.62	0.69	0.88	0.92	0.97	0.98	1.00	1.00
11	0.30	0.42	0.66	0.79	0.90	0.94	0.96	0.98	1.00	0.99
12	0.29	0.49	0.61	0.79	0.89	0.94	0.97	0.99	0.98	1.00
13	0.12	0.27	0.35	0.52	0.63	0.76	0.86	0.93	0.98	0.98
14	0.08	0.20	0.40	0.45	0.73	0.74	0.83	0.95	0.97	0.97
15	0.19	0.21	0.40	0.49	0.63	0.77	0.82	0.91	0.96	0.97
16	0.34	0.49	0.65	0.82	0.92	0.96	0.96	0.99	0.98	0.99
17	0.24	0.38	0.60	0.68	0.82	0.95	0.91	0.97	1.00	1.00
18	0.21	0.33	0.44	0.43	0.56	0.69	0.87	0.86	0.93	0.87
19	0.25	0.56	0.65	0.85	0.91	0.97	0.98	1.00	1.00	1.00
20	0.32	0.39	0.62	0.80	0.91	0.96	0.99	0.99	1.00	1.00
21	0.24	0.37	0.52	0.77	0.89	0.93	0.98	0.97	1.00	1.00
22	0.36	0.71	0.81	0.93	0.97	0.99	1.00	1.00	1.00	1.00
23	0.38	0.63	0.81	0.91	0.97	0.99	1.00	1.00	1.00	1.00
24	0.46	0.62	0.74	0.84	0.96	0.98	0.99	0.99	1.00	1.00
25	0.35	0.58	0.72	0.84	0.93	0.99	0.99	1.00	1.00	1.00
26	0.22	0.39	0.57	0.70	0.87	0.90	0.96	0.98	0.98	1.00
27	0.27	0.24	0.41	0.50	0.65	0.69	0.73	0.85	0.78	0.79
28	0.24	0.42	0.54	0.65	0.73	0.86	0.95	0.97	0.98	1.00
29	0.30	0.38	0.45	0.49	0.67	0.79	0.90	0.94	0.99	0.96
30	0.20	0.34	0.44	0.50	0.61	0.50	0.63	0.73	0.67	0.74
31	0.23	0.27	0.47	0.56	0.73	0.85	0.93	0.97	0.99	1.00
32	0.15	0.33	0.55	0.58	0.73	0.85	0.92	0.96	0.99	0.98
33	0.12	0.29	0.39	0.49	0.72	0.76	0.88	0.96	0.98	0.98
34	0.18	0.45	0.54	0.72	0.89	0.92	0.96	1.00	0.99	0.99
35	0.22	0.39	0.57	0.68	0.84	0.94	0.96	0.98	1.00	1.00
36	0.12	0.31	0.45	0.60	0.71	0.80	0.85	0.94	0.95	0.99
37	0.13	0.22	0.52	0.65	0.72	0.88	0.95	0.99	0.97	0.99
38	0.15	0.24	0.37	0.55	0.74	0.88	0.96	0.97	1.00	0.99
39	0.15	0.13	0.43	0.51	0.68	0.87	0.89	0.96	0.99	0.99
40	0.17	0.35	0.53	0.59	0.79	0.85	0.96	0.98	0.99	1.00
41	0.20	0.27	0.44	0.58	0.68	0.87	0.95	0.97	1.00	1.00
42	0.16	0.23	0.32	0.63	0.76	0.84	0.90	0.96	0.99	1.00

Table 5: POD according to each scenario and each k2 value, k1 = 5

	k2=1	k2=2	k2=3	k2=4	k2=5	k2=6	k2=7	k2=8	k2=9	k2=10
1	0.20	0.20	0.34	0.44	0.48	0.62	0.81	0.80	0.85	0.91
2	0.13	0.22	0.36	0.37	0.60	0.64	0.78	0.78	0.87	0.90
3	0.16	0.16	0.32	0.45	0.44	0.59	0.66	0.73	0.83	0.87
4	0.22	0.37	0.47	0.70	0.74	0.91	0.96	0.99	0.99	0.99
5	0.26	0.41	0.45	0.57	0.82	0.92	0.94	0.92	0.99	0.99
6	0.14	0.32	0.46	0.58	0.60	0.75	0.89	0.89	0.95	0.98
7	0.06	0.03	0.09	0.08	0.17	0.18	0.20	0.23	0.42	0.55
8	0.04	0.05	0.02	0.09	0.17	0.20	0.24	0.25	0.38	0.53
9	0.03	0.04	0.02	0.11	0.17	0.16	0.27	0.30	0.37	0.40
10	0.29	0.40	0.64	0.63	0.83	0.92	0.96	0.97	0.97	1.00
11	0.29	0.43	0.63	0.67	0.81	0.84	0.95	0.97	0.98	0.99
12	0.22	0.40	0.59	0.67	0.86	0.89	0.96	0.97	1.00	1.00
13	0.11	0.18	0.35	0.49	0.69	0.77	0.86	0.93	0.96	1.00
14	0.10	0.17	0.31	0.44	0.73	0.78	0.81	0.89	0.98	0.97
15	0.12	0.16	0.31	0.42	0.50	0.67	0.84	0.90	0.90	0.92
16	0.24	0.48	0.57	0.72	0.79	0.84	0.90	0.98	0.99	1.00
17	0.27	0.42	0.56	0.63	0.80	0.91	0.92	0.91	0.98	1.00
18	0.19	0.31	0.35	0.50	0.56	0.68	0.78	0.87	0.87	0.90
19	0.25	0.46	0.57	0.68	0.91	0.95	0.98	0.98	1.00	1.00
20	0.30	0.51	0.55	0.71	0.89	0.92	1.00	0.98	1.00	1.00
21	0.29	0.42	0.61	0.75	0.81	0.93	0.97	0.97	0.97	1.00
22	0.45	0.65	0.72	0.90	0.97	0.98	1.00	1.00	0.99	1.00
23	0.45	0.57	0.81	0.86	0.96	0.97	1.00	0.99	1.00	1.00
24	0.37	0.59	0.71	0.84	0.94	0.94	1.00	0.99	1.00	1.00
25	0.38	0.55	0.66	0.86	0.96	0.99	0.99	0.99	1.00	1.00
26	0.31	0.40	0.48	0.67	0.76	0.85	0.88	0.93	0.96	0.98
27	0.17	0.29	0.22	0.49	0.47	0.58	0.64	0.74	0.81	0.76
28	0.23	0.38	0.55	0.55	0.80	0.83	0.84	0.95	0.96	0.95
29	0.19	0.28	0.47	0.66	0.71	0.80	0.83	0.90	0.91	0.98
30	0.32	0.36	0.37	0.53	0.44	0.54	0.62	0.67	0.63	0.68
31	0.16	0.20	0.45	0.52	0.69	0.78	0.80	0.93	0.99	1.00
32	0.14	0.31	0.33	0.55	0.67	0.79	0.86	0.93	0.94	1.00
33	0.09	0.19	0.23	0.44	0.61	0.77	0.88	0.87	0.90	0.97
34	0.28	0.38	0.43	0.65	0.78	0.84	0.92	0.93	0.97	0.98
35	0.20	0.35	0.51	0.52	0.69	0.83	0.84	0.93	0.99	0.99
36	0.20	0.23	0.42	0.55	0.65	0.76	0.83	0.89	0.91	0.96
37	0.14	0.21	0.39	0.53	0.72	0.78	0.85	0.89	0.92	0.98
38	0.17	0.25	0.48	0.57	0.69	0.69	0.88	0.93	0.93	0.99
39	0.09	0.25	0.29	0.54	0.67	0.71	0.88	0.92	0.97	0.97
40	0.12	0.25	0.35	0.55	0.72	0.81	0.89	0.87	0.96	0.95
41	0.17	0.30	0.35	0.50	0.76	0.70	0.80	0.92	0.96	0.97
42	0.17	0.26	0.30	0.64	0.58	0.80	0.85	0.93	0.95	0.97

Table 6: POD according to each scenario and each k2 value, k1 = 10
