# Supplementary material for: Evaluation and comparison of statistical methods for early temporal detection of outbreaks: A simulation-based study
Source: PLoS One. 2017 Jul 17;12(7):e0181227. doi: 10.1371/journal.pone.0181227 (PMC5513450; doi:10.1371/journal.pone.0181227)
Supplement: S16 Appendix — (PDF) [file pone.0181227.s016.pdf]

Evaluation and Comparison of Statistical Methods for Early Temporal Detection of Outbreaks: a Simulation-Based Study

Appendix S16: Overall performances of RKI 3 algorithm

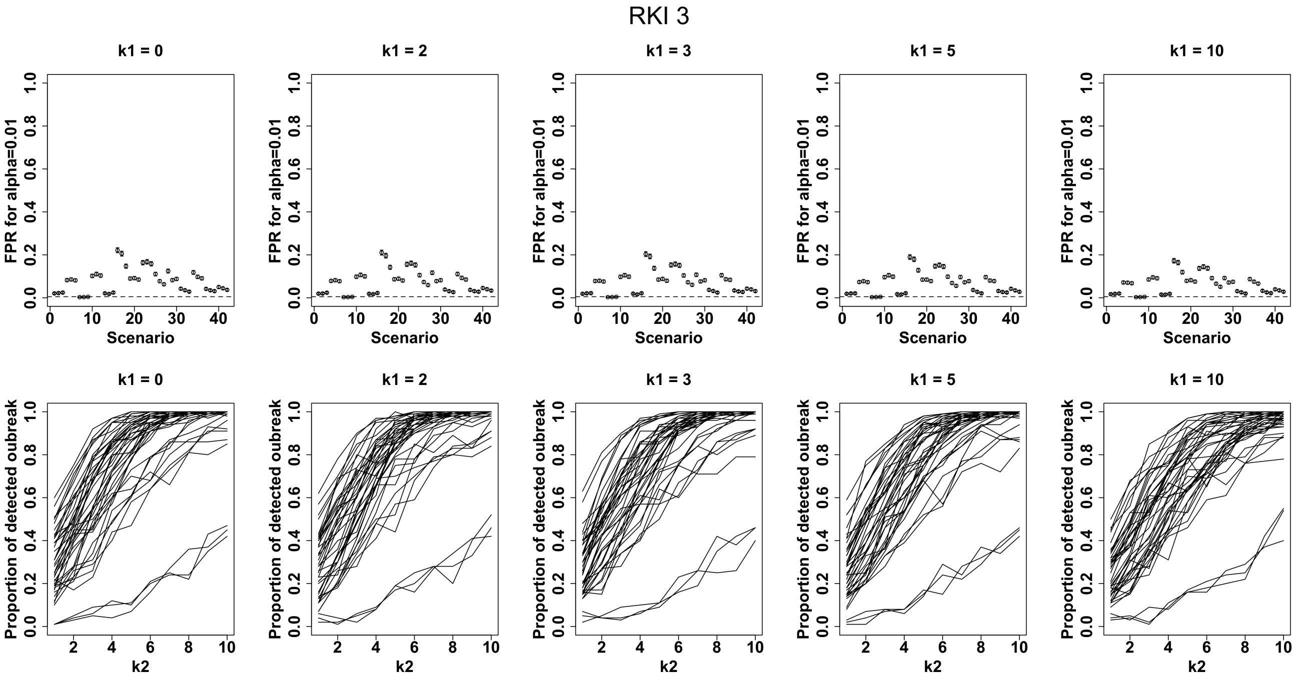

Figure 1: RKI 3 algorithm performances by increasing past outbreak amplitude $k_1 = 0$, 2, 3, 5 or 10 with (i) on the first row: false positive rate for 42 simulated scenarios, (ii) on the second row: probability of detection for 42 simulated scenarios (each curve corresponding to a scenario) by increasing current outbreak amplitude $k_2 = 1$ to 10.

Overall performances of RKI 3 algorithm

	FPR k1=0	FPR k1=2	FPR k1=3	FPR k1=5	FPR k1=10
1	0.02	0.02	0.02	0.02	0.02
2	0.02	0.02	0.02	0.02	0.02
3	0.02	0.02	0.02	0.02	0.02
4	0.08	0.08	0.08	0.07	0.07
5	0.09	0.08	0.08	0.08	0.07
6	0.08	0.08	0.08	0.07	0.07
7	0.00	0.00	0.00	0.00	0.00
8	0.00	0.00	0.00	0.00	0.00
9	0.00	0.00	0.00	0.00	0.00
10	0.10	0.10	0.10	0.10	0.09
11	0.11	0.11	0.11	0.11	0.10
12	0.10	0.10	0.10	0.10	0.09
13	0.02	0.02	0.02	0.02	0.02
14	0.02	0.02	0.02	0.02	0.01
15	0.02	0.02	0.02	0.02	0.02
16	0.22	0.21	0.20	0.19	0.17
17	0.21	0.20	0.19	0.18	0.16
18	0.15	0.14	0.14	0.13	0.12
19	0.09	0.09	0.08	0.08	0.08
20	0.09	0.09	0.09	0.08	0.08
21	0.08	0.08	0.08	0.08	0.08
22	0.16	0.16	0.15	0.15	0.14
23	0.17	0.16	0.16	0.15	0.14
24	0.16	0.15	0.15	0.15	0.14
25	0.11	0.11	0.10	0.10	0.09
26	0.08	0.07	0.07	0.07	0.07
27	0.06	0.06	0.06	0.06	0.05
28	0.12	0.12	0.11	0.10	0.09
29	0.08	0.08	0.08	0.07	0.07
30	0.09	0.08	0.08	0.08	0.07
31	0.04	0.04	0.04	0.04	0.03
32	0.04	0.03	0.03	0.03	0.03
33	0.03	0.03	0.02	0.02	0.02
34	0.12	0.11	0.10	0.10	0.09
35	0.10	0.09	0.09	0.08	0.07
36	0.09	0.08	0.08	0.08	0.07
37	0.04	0.04	0.03	0.03	0.03
38	0.03	0.03	0.03	0.03	0.03
39	0.03	0.03	0.03	0.02	0.02
40	0.05	0.05	0.04	0.04	0.04
41	0.04	0.04	0.04	0.03	0.03
42	0.04	0.03	0.03	0.03	0.03

Table 1: FPR according to each scenario and each k1 value

	k2=1	k2=2	k2=3	k2=4	k2=5	k2=6	k2=7	k2=8	k2=9	k2=10
1	0.12	0.26	0.29	0.40	0.58	0.64	0.73	0.81	0.93	0.92
2	0.19	0.22	0.26	0.54	0.61	0.68	0.80	0.87	0.90	0.96
3	0.22	0.17	0.23	0.44	0.47	0.64	0.75	0.82	0.91	0.91
4	0.31	0.53	0.67	0.86	0.88	0.98	0.97	1.00	1.00	1.00
5	0.43	0.48	0.71	0.87	0.87	0.91	0.97	1.00	0.99	1.00
6	0.39	0.46	0.59	0.73	0.86	0.95	0.94	0.95	1.00	1.00
7	0.01	0.05	0.09	0.10	0.11	0.20	0.24	0.24	0.35	0.42
8	0.01	0.04	0.06	0.12	0.10	0.21	0.25	0.22	0.43	0.47
9	0.01	0.03	0.05	0.04	0.07	0.16	0.27	0.36	0.37	0.45
10	0.37	0.59	0.69	0.84	0.92	0.95	0.99	0.99	1.00	1.00
11	0.39	0.49	0.77	0.95	0.95	0.97	0.99	1.00	0.97	1.00
12	0.39	0.50	0.77	0.83	0.94	0.99	1.00	1.00	0.99	1.00
13	0.10	0.23	0.34	0.59	0.73	0.79	0.85	0.94	0.99	0.99
14	0.16	0.21	0.39	0.48	0.61	0.78	0.87	0.90	0.94	0.99
15	0.20	0.28	0.31	0.49	0.60	0.79	0.83	0.96	0.98	1.00
16	0.60	0.75	0.92	0.97	1.00	1.00	1.00	1.00	1.00	1.00
17	0.56	0.73	0.87	0.91	0.99	1.00	1.00	1.00	1.00	1.00
18	0.51	0.60	0.74	0.82	0.90	0.92	0.98	0.99	1.00	1.00
19	0.29	0.44	0.68	0.80	0.93	0.98	0.98	1.00	1.00	1.00
20	0.31	0.51	0.67	0.85	0.94	0.98	0.98	0.98	1.00	1.00
21	0.25	0.47	0.65	0.77	0.91	0.97	0.97	0.99	1.00	1.00
22	0.48	0.69	0.88	0.97	0.98	0.98	1.00	1.00	1.00	1.00
23	0.50	0.71	0.85	0.95	0.97	1.00	1.00	1.00	1.00	1.00
24	0.50	0.67	0.86	0.95	1.00	1.00	1.00	1.00	1.00	1.00
25	0.45	0.59	0.78	0.87	0.95	0.99	1.00	1.00	1.00	1.00
26	0.28	0.41	0.69	0.75	0.90	0.93	0.99	1.00	1.00	0.98
27	0.23	0.43	0.44	0.60	0.70	0.68	0.86	0.86	0.86	0.87
28	0.41	0.61	0.72	0.85	0.92	0.97	0.98	1.00	0.99	1.00
29	0.35	0.43	0.55	0.79	0.87	0.93	0.98	1.00	1.00	1.00
30	0.25	0.47	0.50	0.62	0.64	0.72	0.66	0.81	0.80	0.85
31	0.17	0.45	0.45	0.66	0.81	0.99	0.99	1.00	0.99	1.00
32	0.19	0.40	0.55	0.64	0.77	0.95	0.94	0.99	0.99	1.00
33	0.19	0.27	0.49	0.60	0.78	0.91	0.93	0.98	1.00	1.00
34	0.42	0.63	0.79	0.87	0.99	1.00	1.00	1.00	1.00	1.00
35	0.41	0.55	0.80	0.90	0.96	1.00	0.99	1.00	1.00	1.00
36	0.31	0.48	0.71	0.86	0.94	0.95	0.99	0.99	1.00	1.00
37	0.11	0.39	0.52	0.70	0.80	0.94	0.95	0.97	0.99	1.00
38	0.18	0.23	0.56	0.70	0.80	0.87	1.00	0.99	1.00	1.00
39	0.14	0.28	0.54	0.72	0.82	0.91	0.94	1.00	1.00	1.00
40	0.25	0.41	0.55	0.80	0.81	0.94	0.98	1.00	1.00	1.00
41	0.24	0.37	0.46	0.72	0.86	0.93	0.97	1.00	1.00	1.00
42	0.21	0.35	0.47	0.57	0.78	0.89	0.99	0.99	0.99	1.00

Table 2: POD according to each scenario and each k2 value, k1 = 0

	k2=1	k2=2	k2=3	k2=4	k2=5	k2=6	k2=7	k2=8	k2=9	k2=10
1	0.14	0.18	0.32	0.41	0.57	0.59	0.74	0.84	0.85	0.91
2	0.11	0.26	0.28	0.48	0.62	0.70	0.78	0.83	0.83	0.96
3	0.11	0.20	0.34	0.48	0.44	0.68	0.79	0.80	0.86	0.91
4	0.42	0.59	0.66	0.81	0.90	0.97	0.95	1.00	1.00	1.00
5	0.41	0.53	0.69	0.83	0.87	0.94	0.95	1.00	0.99	0.99
6	0.23	0.46	0.61	0.74	0.82	0.90	0.93	1.00	0.98	1.00
7	0.06	0.04	0.02	0.08	0.19	0.16	0.27	0.34	0.41	0.42
8	0.02	0.02	0.05	0.08	0.19	0.25	0.28	0.28	0.33	0.46
9	0.04	0.01	0.06	0.09	0.17	0.20	0.28	0.20	0.40	0.52
10	0.40	0.53	0.74	0.87	0.88	0.96	0.99	0.98	1.00	1.00
11	0.42	0.57	0.80	0.76	0.96	0.96	0.98	1.00	1.00	1.00
12	0.33	0.55	0.72	0.80	0.95	0.98	0.99	0.99	0.99	1.00
13	0.13	0.19	0.38	0.58	0.75	0.76	0.92	0.95	0.98	0.98
14	0.07	0.21	0.38	0.50	0.52	0.78	0.89	0.91	0.97	0.98
15	0.20	0.27	0.35	0.53	0.78	0.78	0.86	0.95	0.93	0.97
16	0.62	0.78	0.90	0.96	0.98	0.99	1.00	1.00	1.00	1.00
17	0.52	0.67	0.89	0.97	0.97	1.00	1.00	1.00	1.00	1.00
18	0.43	0.60	0.71	0.84	0.91	0.94	0.97	0.99	1.00	1.00
19	0.31	0.51	0.64	0.86	0.94	0.98	0.99	0.99	1.00	1.00
20	0.34	0.48	0.60	0.84	0.85	0.99	0.98	0.99	1.00	1.00
21	0.28	0.36	0.61	0.77	0.86	0.94	1.00	0.99	1.00	1.00
22	0.43	0.67	0.83	0.95	0.96	1.00	1.00	1.00	1.00	1.00
23	0.50	0.66	0.79	0.96	0.98	1.00	1.00	1.00	1.00	1.00
24	0.57	0.70	0.85	0.89	0.97	1.00	1.00	1.00	1.00	1.00
25	0.41	0.59	0.70	0.92	1.00	0.97	1.00	1.00	1.00	1.00
26	0.29	0.49	0.73	0.70	0.85	0.98	0.98	0.98	1.00	1.00
27	0.16	0.33	0.48	0.66	0.66	0.85	0.81	0.89	0.83	0.88
28	0.40	0.56	0.60	0.85	0.93	0.96	0.98	0.99	1.00	1.00
29	0.33	0.44	0.53	0.76	0.83	0.92	0.99	0.98	1.00	1.00
30	0.28	0.40	0.44	0.69	0.70	0.74	0.77	0.80	0.79	0.84
31	0.26	0.30	0.49	0.66	0.86	0.92	0.97	0.98	1.00	1.00
32	0.15	0.34	0.56	0.71	0.83	0.91	0.96	0.98	0.95	0.99
33	0.07	0.21	0.41	0.66	0.79	0.84	0.93	0.97	0.99	1.00
34	0.37	0.69	0.74	0.87	0.94	0.96	0.98	1.00	1.00	1.00
35	0.38	0.54	0.62	0.80	0.92	0.98	1.00	1.00	1.00	1.00
36	0.37	0.44	0.67	0.78	0.90	0.92	0.99	0.99	1.00	1.00
37	0.21	0.30	0.52	0.61	0.92	0.92	0.99	0.98	1.00	1.00
38	0.13	0.30	0.48	0.69	0.89	0.96	0.97	0.99	1.00	1.00
39	0.11	0.34	0.42	0.65	0.85	0.90	0.95	0.96	1.00	1.00
40	0.25	0.50	0.54	0.75	0.87	0.94	1.00	0.98	0.98	1.00
41	0.23	0.24	0.54	0.75	0.82	0.97	0.99	0.99	1.00	1.00
42	0.21	0.30	0.54	0.66	0.80	0.92	0.98	0.98	1.00	1.00

Table 3: POD according to each scenario and each k2 value, k1 = 2

	k2=1	k2=2	k2=3	k2=4	k2=5	k2=6	k2=7	k2=8	k2=9	k2=10
1	0.17	0.17	0.34	0.41	0.64	0.60	0.72	0.86	0.90	0.92
2	0.13	0.27	0.34	0.40	0.57	0.61	0.77	0.82	0.89	0.92
3	0.15	0.27	0.28	0.43	0.50	0.67	0.73	0.86	0.87	0.92
4	0.34	0.45	0.62	0.79	0.89	0.97	0.97	0.98	0.99	0.99
5	0.40	0.50	0.62	0.85	0.85	0.92	0.97	0.99	1.00	1.00
6	0.28	0.49	0.61	0.68	0.74	0.92	0.96	0.97	0.96	1.00
7	0.07	0.04	0.04	0.06	0.11	0.16	0.19	0.35	0.42	0.46
8	0.05	0.04	0.03	0.07	0.09	0.17	0.26	0.25	0.26	0.40
9	0.02	0.05	0.09	0.10	0.11	0.23	0.26	0.42	0.38	0.46
10	0.40	0.48	0.71	0.86	0.90	0.98	0.99	1.00	0.99	1.00
11	0.48	0.57	0.70	0.91	0.91	0.97	0.99	0.99	1.00	1.00
12	0.38	0.49	0.70	0.80	0.93	0.93	0.98	1.00	0.99	1.00
13	0.13	0.27	0.38	0.47	0.72	0.75	0.88	0.91	0.98	0.99
14	0.16	0.15	0.40	0.49	0.66	0.75	0.89	0.94	0.96	0.96
15	0.13	0.22	0.46	0.57	0.57	0.76	0.84	0.92	0.96	0.96
16	0.58	0.77	0.87	0.97	0.98	0.99	1.00	1.00	1.00	1.00
17	0.63	0.81	0.90	0.96	0.97	1.00	1.00	1.00	0.99	1.00
18	0.33	0.56	0.73	0.81	0.89	0.95	0.97	1.00	1.00	1.00
19	0.30	0.50	0.65	0.86	0.86	0.93	1.00	0.99	1.00	1.00
20	0.25	0.47	0.65	0.72	0.88	0.95	0.99	1.00	1.00	1.00
21	0.24	0.51	0.65	0.71	0.86	0.93	0.94	0.99	1.00	1.00
22	0.44	0.68	0.87	0.95	0.99	1.00	1.00	1.00	1.00	1.00
23	0.40	0.69	0.89	0.94	0.98	1.00	1.00	1.00	1.00	1.00
24	0.38	0.63	0.79	0.93	0.97	0.99	1.00	1.00	1.00	1.00
25	0.34	0.58	0.78	0.94	0.97	0.99	1.00	1.00	1.00	1.00
26	0.30	0.42	0.61	0.70	0.90	0.93	0.93	1.00	1.00	1.00
27	0.20	0.29	0.50	0.59	0.73	0.77	0.84	0.80	0.86	0.89
28	0.40	0.46	0.69	0.75	0.90	0.95	0.99	0.99	1.00	1.00
29	0.33	0.48	0.58	0.75	0.88	0.89	0.94	0.98	1.00	0.99
30	0.30	0.43	0.50	0.61	0.59	0.75	0.71	0.71	0.79	0.79
31	0.20	0.35	0.43	0.57	0.77	0.94	0.96	1.00	0.99	1.00
32	0.15	0.35	0.44	0.63	0.77	0.83	0.93	1.00	0.98	1.00
33	0.21	0.26	0.40	0.57	0.69	0.91	0.97	0.99	1.00	1.00
34	0.34	0.57	0.70	0.85	0.93	0.98	1.00	1.00	1.00	1.00
35	0.43	0.53	0.78	0.79	0.94	0.95	1.00	1.00	1.00	1.00
36	0.26	0.55	0.67	0.79	0.94	0.95	0.98	0.99	0.98	1.00
37	0.15	0.42	0.55	0.57	0.78	0.91	0.96	0.96	0.99	1.00
38	0.15	0.21	0.39	0.69	0.78	0.92	0.95	0.98	0.99	1.00
39	0.16	0.30	0.50	0.64	0.79	0.91	0.93	1.00	1.00	1.00
40	0.18	0.42	0.53	0.78	0.81	0.95	0.95	0.99	1.00	1.00
41	0.18	0.36	0.45	0.68	0.79	0.90	0.94	0.99	1.00	1.00
42	0.20	0.39	0.51	0.67	0.86	0.92	0.98	0.99	1.00	1.00

Table 4: POD according to each scenario and each k2 value, k1 = 3

	k2=1	k2=2	k2=3	k2=4	k2=5	k2=6	k2=7	k2=8	k2=9	k2=10
1	0.15	0.20	0.28	0.43	0.52	0.56	0.78	0.94	0.89	0.94
2	0.13	0.20	0.33	0.41	0.55	0.70	0.75	0.82	0.89	0.86
3	0.14	0.20	0.33	0.39	0.50	0.60	0.77	0.79	0.87	0.88
4	0.36	0.49	0.63	0.78	0.87	0.93	0.95	1.00	1.00	1.00
5	0.28	0.48	0.65	0.75	0.92	0.94	0.97	0.98	1.00	0.98
6	0.24	0.39	0.56	0.75	0.78	0.93	0.95	0.97	0.98	1.00
7	0.01	0.01	0.08	0.08	0.17	0.15	0.28	0.32	0.37	0.45
8	0.02	0.04	0.07	0.08	0.15	0.24	0.22	0.29	0.39	0.46
9	0.03	0.07	0.08	0.06	0.14	0.29	0.24	0.37	0.34	0.42
10	0.29	0.48	0.74	0.78	0.92	0.98	0.98	0.98	1.00	1.00
11	0.43	0.48	0.76	0.86	0.94	0.95	0.97	1.00	1.00	0.99
12	0.36	0.54	0.70	0.86	0.90	0.96	0.99	1.00	0.99	1.00
13	0.14	0.28	0.37	0.54	0.66	0.78	0.84	0.93	0.97	0.98
14	0.08	0.22	0.35	0.53	0.70	0.75	0.88	0.93	0.98	0.97
15	0.14	0.22	0.39	0.50	0.66	0.78	0.86	0.94	0.94	1.00
16	0.59	0.75	0.83	0.92	0.98	0.99	0.99	1.00	1.00	1.00
17	0.52	0.65	0.83	0.90	0.96	0.98	1.00	1.00	1.00	1.00
18	0.41	0.53	0.68	0.77	0.88	0.91	0.99	0.97	0.99	0.99
19	0.28	0.55	0.63	0.80	0.86	0.97	0.97	1.00	1.00	1.00
20	0.29	0.39	0.60	0.82	0.93	0.95	0.99	0.98	1.00	1.00
21	0.28	0.35	0.52	0.73	0.95	0.94	0.98	0.97	1.00	1.00
22	0.41	0.77	0.85	0.92	0.98	0.99	1.00	1.00	1.00	1.00
23	0.46	0.66	0.79	0.94	0.98	0.99	1.00	1.00	1.00	1.00
24	0.52	0.65	0.81	0.89	0.97	0.99	0.99	1.00	1.00	1.00
25	0.39	0.57	0.74	0.87	0.95	0.99	0.99	1.00	1.00	1.00
26	0.20	0.39	0.56	0.72	0.87	0.92	0.98	0.98	1.00	1.00
27	0.25	0.34	0.49	0.54	0.69	0.77	0.84	0.91	0.87	0.87
28	0.33	0.52	0.62	0.74	0.77	0.92	0.94	0.97	0.99	1.00
29	0.35	0.49	0.55	0.70	0.81	0.86	0.97	0.99	1.00	1.00
30	0.24	0.42	0.53	0.60	0.68	0.57	0.72	0.76	0.72	0.83
31	0.18	0.29	0.46	0.54	0.68	0.85	0.92	0.98	1.00	1.00
32	0.20	0.34	0.55	0.60	0.75	0.85	0.93	0.97	0.99	1.00
33	0.09	0.26	0.40	0.59	0.78	0.83	0.90	0.95	1.00	0.99
34	0.30	0.51	0.72	0.83	0.95	0.93	0.98	1.00	0.99	1.00
35	0.33	0.54	0.62	0.80	0.91	0.97	0.99	1.00	0.99	1.00
36	0.22	0.45	0.66	0.82	0.90	0.91	0.95	0.97	0.99	1.00
37	0.17	0.24	0.52	0.68	0.79	0.87	0.98	0.99	0.97	1.00
38	0.15	0.33	0.36	0.56	0.82	0.88	0.97	0.98	1.00	0.99
39	0.15	0.20	0.51	0.53	0.75	0.89	0.92	0.98	0.99	1.00
40	0.18	0.35	0.52	0.62	0.75	0.89	0.96	0.97	0.99	1.00
41	0.24	0.25	0.46	0.63	0.73	0.90	0.96	0.96	1.00	1.00
42	0.15	0.25	0.37	0.71	0.75	0.89	0.95	0.99	0.99	1.00

Table 5: POD according to each scenario and each k2 value, k1 = 5

	k2=1	k2=2	k2=3	k2=4	k2=5	k2=6	k2=7	k2=8	k2=9	k2=10
1	0.15	0.21	0.31	0.45	0.49	0.66	0.81	0.76	0.81	0.89
2	0.11	0.24	0.36	0.31	0.58	0.69	0.78	0.79	0.89	0.90
3	0.18	0.15	0.30	0.42	0.46	0.59	0.61	0.76	0.88	0.88
4	0.29	0.42	0.55	0.73	0.79	0.94	0.96	0.98	0.99	0.99
5	0.25	0.47	0.52	0.69	0.86	0.95	0.95	0.95	0.98	0.99
6	0.18	0.32	0.57	0.66	0.78	0.87	0.95	0.96	0.99	0.99
7	0.06	0.03	0.09	0.08	0.16	0.18	0.20	0.22	0.42	0.55
8	0.04	0.05	0.02	0.09	0.17	0.21	0.24	0.25	0.38	0.54
9	0.03	0.04	0.01	0.11	0.16	0.16	0.26	0.29	0.37	0.40
10	0.32	0.48	0.71	0.77	0.90	0.96	0.98	0.97	0.98	1.00
11	0.34	0.49	0.73	0.76	0.89	0.93	0.94	0.99	1.00	0.99
12	0.30	0.46	0.65	0.75	0.88	0.95	0.96	0.98	1.00	1.00
13	0.12	0.17	0.34	0.51	0.65	0.72	0.85	0.92	0.97	1.00
14	0.09	0.16	0.37	0.45	0.73	0.73	0.85	0.91	0.96	0.97
15	0.11	0.15	0.35	0.43	0.54	0.73	0.85	0.87	0.92	0.93
16	0.39	0.60	0.73	0.75	0.93	0.89	0.92	1.00	1.00	0.99
17	0.50	0.67	0.79	0.84	0.96	0.96	0.99	0.98	1.00	1.00
18	0.34	0.53	0.61	0.72	0.79	0.88	0.92	0.95	0.99	0.98
19	0.21	0.43	0.54	0.69	0.88	0.95	0.97	0.99	1.00	1.00
20	0.30	0.43	0.51	0.72	0.87	0.89	0.99	0.98	1.00	1.00
21	0.28	0.37	0.64	0.80	0.83	0.94	0.95	0.96	0.97	1.00
22	0.45	0.68	0.74	0.91	0.97	0.99	1.00	1.00	1.00	1.00
23	0.46	0.56	0.85	0.90	0.97	0.99	1.00	1.00	1.00	1.00
24	0.44	0.63	0.72	0.88	0.93	0.99	0.99	1.00	0.99	1.00
25	0.36	0.47	0.69	0.83	0.89	0.97	1.00	0.99	0.99	1.00
26	0.29	0.39	0.54	0.72	0.76	0.91	0.89	0.94	0.97	0.97
27	0.19	0.31	0.27	0.54	0.53	0.69	0.72	0.83	0.85	0.88
28	0.31	0.38	0.55	0.63	0.78	0.79	0.80	0.90	0.96	0.96
29	0.20	0.34	0.61	0.71	0.81	0.88	0.90	0.90	0.92	0.98
30	0.30	0.37	0.46	0.67	0.63	0.72	0.66	0.76	0.77	0.78
31	0.19	0.22	0.37	0.53	0.63	0.75	0.83	0.91	0.94	1.00
32	0.12	0.30	0.42	0.55	0.64	0.76	0.90	0.89	0.91	0.97
33	0.15	0.22	0.24	0.44	0.58	0.72	0.82	0.88	0.91	0.96
34	0.35	0.53	0.53	0.73	0.83	0.88	0.93	0.94	0.97	1.00
35	0.27	0.44	0.61	0.60	0.79	0.85	0.87	0.93	0.98	0.98
36	0.32	0.37	0.59	0.63	0.73	0.84	0.87	0.94	0.97	0.96
37	0.11	0.24	0.38	0.54	0.65	0.71	0.79	0.92	0.92	0.95
38	0.15	0.25	0.50	0.58	0.70	0.69	0.83	0.90	0.90	0.99
39	0.11	0.26	0.28	0.46	0.64	0.75	0.86	0.89	0.95	0.94
40	0.14	0.30	0.39	0.60	0.67	0.76	0.77	0.93	0.93	0.93
41	0.16	0.26	0.33	0.50	0.79	0.65	0.84	0.89	0.95	0.94
42	0.16	0.33	0.41	0.61	0.63	0.77	0.85	0.96	0.94	0.97

Table 6: POD according to each scenario and each k2 value, k1 = 10
