# Supplementary material for: Evaluation and comparison of statistical methods for early temporal detection of outbreaks: A simulation-based study
Source: PLoS One. 2017 Jul 17;12(7):e0181227. doi: 10.1371/journal.pone.0181227 (PMC5513450; doi:10.1371/journal.pone.0181227)
Supplement: S17 Appendix — (PDF) [file pone.0181227.s017.pdf]

Evaluation and Comparison of Statistical Methods for Early Temporal Detection of Outbreaks: a Simulation-Based Study

Appendix S17: Overall performances of GLR Negative Binomial algorithm

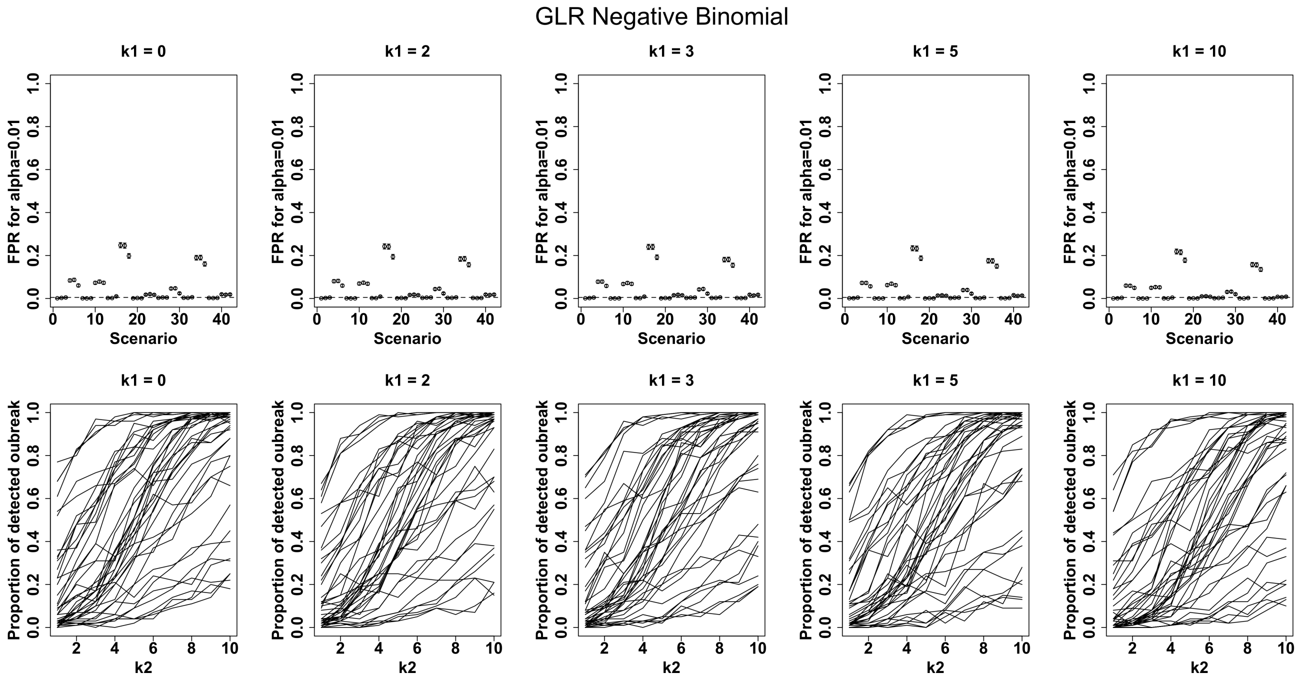

Figure 1: GLR Negative Binomial algorithm performances by increasing past outbreak amplitude $k_1 = 0$, 2, 3, 5 or 10 with (i) on the first row: false positive rate for 42 simulated scenarios, (ii) on the second row: probability of detection for 42 simulated scenarios (each curve corresponding to a scenario) by increasing current outbreak amplitude $k_2 = 1$ to 10.

Overall performances of GLR Negative Binomial algorithm

	FPR k1=0	FPR k1=2	FPR k1=3	FPR k1=5	FPR k1=10
1	0.00	0.00	0.00	0.00	0.00
2	0.00	0.00	0.00	0.00	0.00
3	0.00	0.00	0.00	0.00	0.00
4	0.08	0.08	0.08	0.07	0.06
5	0.09	0.08	0.08	0.07	0.06
6	0.06	0.06	0.06	0.06	0.05
7	0.00	0.00	0.00	0.00	0.00
8	0.00	0.00	0.00	0.00	0.00
9	0.00	0.00	0.00	0.00	0.00
10	0.07	0.07	0.07	0.06	0.05
11	0.08	0.07	0.07	0.07	0.05
12	0.07	0.07	0.07	0.06	0.05
13	0.00	0.00	0.00	0.00	0.00
14	0.00	0.00	0.00	0.00	0.00
15	0.01	0.01	0.01	0.01	0.00
16	0.25	0.24	0.24	0.23	0.22
17	0.25	0.24	0.24	0.23	0.22
18	0.20	0.19	0.19	0.19	0.18
19	0.00	0.00	0.00	0.00	0.00
20	0.00	0.00	0.00	0.00	0.00
21	0.00	0.00	0.00	0.00	0.00
22	0.02	0.02	0.02	0.01	0.01
23	0.02	0.02	0.02	0.01	0.01
24	0.02	0.02	0.01	0.01	0.01
25	0.00	0.00	0.00	0.00	0.00
26	0.00	0.00	0.00	0.00	0.00
27	0.00	0.00	0.00	0.00	0.00
28	0.05	0.04	0.04	0.04	0.03
29	0.05	0.05	0.04	0.04	0.03
30	0.02	0.02	0.02	0.02	0.02
31	0.00	0.00	0.00	0.00	0.00
32	0.00	0.00	0.00	0.00	0.00
33	0.01	0.01	0.01	0.00	0.00
34	0.19	0.18	0.18	0.18	0.16
35	0.19	0.18	0.18	0.17	0.16
36	0.16	0.16	0.15	0.15	0.14
37	0.00	0.00	0.00	0.00	0.00
38	0.00	0.00	0.00	0.00	0.00
39	0.00	0.00	0.00	0.00	0.00
40	0.02	0.02	0.02	0.02	0.01
41	0.02	0.02	0.01	0.01	0.01
42	0.02	0.02	0.02	0.01	0.01

Table 1: FPR according to each scenario and each k1 value

	k2=1	k2=2	k2=3	k2=4	k2=5	k2=6	k2=7	k2=8	k2=9	k2=10
1	0.00	0.01	0.05	0.12	0.20	0.37	0.41	0.61	0.77	0.80
2	0.03	0.04	0.08	0.20	0.33	0.35	0.53	0.67	0.79	0.88
3	0.06	0.07	0.07	0.06	0.20	0.27	0.29	0.34	0.43	0.57
4	0.32	0.52	0.57	0.80	0.91	0.94	0.97	0.99	0.99	1.00
5	0.27	0.48	0.65	0.82	0.84	0.92	0.96	0.99	0.99	1.00
6	0.23	0.27	0.39	0.33	0.43	0.62	0.57	0.58	0.70	0.66
7	0.03	0.03	0.03	0.06	0.04	0.06	0.09	0.11	0.14	0.25
8	0.03	0.02	0.00	0.04	0.04	0.04	0.13	0.13	0.20	0.18
9	0.01	0.01	0.02	0.04	0.04	0.08	0.14	0.17	0.16	0.25
10	0.33	0.48	0.59	0.72	0.84	0.83	0.92	0.95	1.00	0.95
11	0.31	0.41	0.57	0.74	0.90	0.87	0.97	0.98	0.96	1.00
12	0.36	0.37	0.57	0.69	0.76	0.90	0.96	0.98	0.98	0.98
13	0.01	0.05	0.10	0.27	0.46	0.58	0.73	0.82	0.93	0.96
14	0.04	0.07	0.14	0.24	0.39	0.68	0.69	0.81	0.92	0.97
15	0.06	0.15	0.12	0.20	0.32	0.39	0.47	0.53	0.61	0.80
16	0.68	0.82	0.91	0.96	0.96	1.00	1.00	1.00	1.00	1.00
17	0.77	0.80	0.97	0.96	0.99	0.99	0.99	1.00	0.99	1.00
18	0.54	0.61	0.66	0.67	0.74	0.66	0.80	0.86	0.86	0.94
19	0.01	0.02	0.01	0.00	0.02	0.07	0.10	0.14	0.29	0.32
20	0.02	0.02	0.02	0.03	0.09	0.21	0.28	0.37	0.39	0.40
21	0.02	0.02	0.02	0.04	0.09	0.13	0.18	0.27	0.32	0.31
22	0.09	0.14	0.21	0.27	0.50	0.60	0.79	0.89	0.93	0.98
23	0.15	0.27	0.31	0.31	0.60	0.72	0.74	0.88	0.86	0.93
24	0.08	0.20	0.17	0.33	0.39	0.42	0.47	0.61	0.70	0.75
25	0.02	0.04	0.10	0.13	0.31	0.55	0.72	0.83	0.97	0.98
26	0.02	0.04	0.12	0.23	0.43	0.54	0.73	0.83	0.90	0.92
27	0.03	0.04	0.04	0.12	0.11	0.17	0.19	0.23	0.21	0.22
28	0.23	0.48	0.49	0.74	0.83	0.96	0.97	1.00	1.00	1.00
29	0.20	0.38	0.53	0.77	0.80	0.94	0.96	0.98	1.00	0.99
30	0.09	0.15	0.19	0.21	0.14	0.24	0.22	0.20	0.34	0.45
31	0.03	0.07	0.12	0.36	0.51	0.76	0.87	0.97	0.97	0.98
32	0.02	0.07	0.22	0.35	0.51	0.72	0.83	0.97	0.98	1.00
33	0.02	0.09	0.17	0.23	0.34	0.49	0.64	0.76	0.80	0.88
34	0.68	0.82	0.94	0.94	1.00	1.00	0.99	1.00	1.00	1.00
35	0.61	0.83	0.90	0.98	1.00	0.98	1.00	1.00	1.00	1.00
36	0.52	0.68	0.72	0.75	0.95	0.93	0.97	0.98	0.99	0.99
37	0.00	0.12	0.17	0.44	0.58	0.68	0.89	0.97	0.99	0.98
38	0.00	0.07	0.14	0.32	0.51	0.75	0.88	0.98	0.99	0.99
39	0.00	0.10	0.17	0.36	0.45	0.69	0.80	0.94	0.97	0.99
40	0.11	0.31	0.48	0.66	0.73	0.92	0.96	1.00	0.99	1.00
41	0.10	0.26	0.39	0.67	0.77	0.92	0.96	0.97	1.00	1.00
42	0.06	0.21	0.31	0.46	0.65	0.77	0.92	0.93	0.99	1.00

Table 2: POD according to each scenario and each k2 value, k1 = 0

	k2=1	k2=2	k2=3	k2=4	k2=5	k2=6	k2=7	k2=8	k2=9	k2=10
1	0.00	0.03	0.07	0.09	0.21	0.26	0.36	0.59	0.61	0.68
2	0.00	0.09	0.06	0.18	0.37	0.36	0.47	0.60	0.69	0.83
3	0.03	0.05	0.04	0.09	0.21	0.31	0.32	0.41	0.47	0.57
4	0.36	0.44	0.66	0.76	0.90	0.95	0.95	1.00	0.99	1.00
5	0.37	0.58	0.65	0.83	0.86	0.96	0.94	0.99	0.99	0.99
6	0.14	0.21	0.32	0.36	0.43	0.54	0.57	0.59	0.75	0.63
7	0.02	0.02	0.01	0.02	0.06	0.07	0.10	0.14	0.23	0.15
8	0.01	0.02	0.00	0.01	0.05	0.06	0.09	0.08	0.07	0.21
9	0.00	0.01	0.02	0.03	0.03	0.05	0.09	0.06	0.10	0.16
10	0.24	0.39	0.59	0.74	0.76	0.87	0.92	0.97	0.99	1.00
11	0.32	0.38	0.64	0.61	0.87	0.83	0.93	1.00	0.98	0.99
12	0.30	0.40	0.56	0.69	0.72	0.89	0.90	0.94	0.98	0.98
13	0.02	0.04	0.10	0.25	0.45	0.46	0.75	0.87	0.90	0.96
14	0.04	0.04	0.16	0.23	0.34	0.55	0.71	0.85	0.93	0.97
15	0.06	0.07	0.13	0.19	0.35	0.43	0.49	0.59	0.55	0.70
16	0.64	0.85	0.94	0.99	0.98	0.99	1.00	1.00	1.00	1.00
17	0.67	0.81	0.90	0.98	0.99	1.00	1.00	1.00	1.00	1.00
18	0.47	0.58	0.61	0.74	0.70	0.77	0.71	0.88	0.84	0.97
19	0.00	0.00	0.00	0.02	0.03	0.10	0.11	0.21	0.29	0.37
20	0.01	0.03	0.02	0.05	0.07	0.15	0.21	0.30	0.41	0.55
21	0.01	0.02	0.00	0.05	0.07	0.10	0.13	0.20	0.26	0.34
22	0.10	0.12	0.20	0.36	0.43	0.59	0.77	0.87	0.92	0.97
23	0.11	0.25	0.20	0.39	0.49	0.64	0.81	0.88	0.89	0.93
24	0.12	0.15	0.19	0.25	0.33	0.47	0.50	0.63	0.64	0.70
25	0.02	0.05	0.09	0.17	0.36	0.41	0.63	0.86	0.96	0.97
26	0.03	0.08	0.18	0.16	0.37	0.56	0.70	0.75	0.89	0.97
27	0.04	0.03	0.11	0.17	0.14	0.23	0.22	0.22	0.23	0.21
28	0.20	0.37	0.50	0.81	0.87	0.93	0.98	1.00	1.00	0.99
29	0.22	0.36	0.41	0.68	0.84	0.92	0.98	0.98	1.00	0.99
30	0.08	0.09	0.20	0.25	0.25	0.23	0.33	0.31	0.39	0.38
31	0.04	0.11	0.18	0.34	0.47	0.73	0.90	0.94	0.97	0.99
32	0.02	0.09	0.24	0.33	0.62	0.72	0.83	0.96	0.94	0.98
33	0.04	0.05	0.19	0.20	0.36	0.55	0.60	0.70	0.81	0.93
34	0.61	0.88	0.90	0.96	0.99	1.00	1.00	1.00	1.00	1.00
35	0.58	0.81	0.87	0.94	1.00	0.99	1.00	1.00	1.00	1.00
36	0.53	0.58	0.72	0.79	0.89	0.88	0.98	0.97	0.98	1.00
37	0.04	0.05	0.17	0.37	0.65	0.68	0.87	0.96	0.99	1.00
38	0.01	0.05	0.16	0.38	0.56	0.80	0.90	0.95	0.99	1.00
39	0.02	0.07	0.15	0.27	0.46	0.68	0.83	0.90	0.97	1.00
40	0.09	0.30	0.42	0.66	0.83	0.90	0.98	1.00	0.98	1.00
41	0.11	0.15	0.41	0.55	0.70	0.94	0.97	0.98	1.00	1.00
42	0.12	0.10	0.27	0.46	0.63	0.76	0.91	0.94	0.98	0.99

Table 3: POD according to each scenario and each k2 value, k1 = 2

	k2=1	k2=2	k2=3	k2=4	k2=5	k2=6	k2=7	k2=8	k2=9	k2=10
1	0.03	0.04	0.04	0.09	0.20	0.26	0.38	0.57	0.68	0.80
2	0.01	0.05	0.08	0.16	0.20	0.36	0.48	0.57	0.70	0.74
3	0.00	0.07	0.08	0.12	0.23	0.27	0.28	0.34	0.38	0.48
4	0.36	0.44	0.58	0.73	0.90	0.93	0.97	0.98	0.99	1.00
5	0.36	0.55	0.59	0.80	0.86	0.93	0.94	0.99	1.00	1.00
6	0.12	0.35	0.25	0.37	0.40	0.54	0.60	0.63	0.70	0.76
7	0.03	0.02	0.01	0.03	0.03	0.06	0.05	0.11	0.15	0.20
8	0.00	0.01	0.01	0.03	0.02	0.10	0.07	0.06	0.13	0.19
9	0.02	0.01	0.03	0.03	0.05	0.05	0.07	0.12	0.12	0.19
10	0.32	0.39	0.59	0.62	0.76	0.91	0.91	0.93	0.99	1.00
11	0.35	0.42	0.56	0.64	0.80	0.89	0.95	0.97	0.97	0.99
12	0.28	0.42	0.51	0.65	0.84	0.88	0.84	0.94	0.97	0.99
13	0.01	0.07	0.12	0.21	0.44	0.53	0.73	0.77	0.92	0.97
14	0.03	0.04	0.12	0.24	0.45	0.55	0.72	0.89	0.91	0.95
15	0.07	0.09	0.14	0.24	0.29	0.41	0.52	0.59	0.65	0.63
16	0.70	0.82	0.92	0.98	0.97	0.99	1.00	1.00	1.00	0.99
17	0.71	0.81	0.93	0.96	0.99	1.00	1.00	1.00	1.00	1.00
18	0.47	0.54	0.67	0.60	0.64	0.81	0.79	0.84	0.87	0.93
19	0.00	0.00	0.02	0.00	0.03	0.08	0.12	0.21	0.21	0.38
20	0.03	0.01	0.02	0.06	0.04	0.12	0.19	0.30	0.43	0.42
21	0.00	0.02	0.03	0.02	0.05	0.12	0.17	0.20	0.25	0.40
22	0.06	0.12	0.15	0.35	0.44	0.57	0.70	0.81	0.92	0.95
23	0.06	0.11	0.25	0.37	0.55	0.58	0.79	0.85	0.91	0.91
24	0.08	0.10	0.23	0.35	0.40	0.46	0.49	0.55	0.68	0.69
25	0.00	0.04	0.06	0.14	0.32	0.42	0.60	0.83	0.90	0.97
26	0.07	0.09	0.12	0.18	0.31	0.53	0.53	0.74	0.86	0.96
27	0.01	0.02	0.12	0.13	0.11	0.16	0.19	0.22	0.20	0.24
28	0.17	0.30	0.51	0.68	0.84	0.93	0.96	1.00	1.00	1.00
29	0.19	0.32	0.44	0.73	0.85	0.84	0.93	0.99	0.99	1.00
30	0.07	0.13	0.21	0.22	0.17	0.23	0.33	0.31	0.36	0.33
31	0.01	0.10	0.13	0.30	0.53	0.86	0.84	0.97	0.97	1.00
32	0.03	0.03	0.11	0.27	0.58	0.70	0.91	0.94	0.97	1.00
33	0.03	0.09	0.20	0.22	0.32	0.48	0.62	0.76	0.84	0.93
34	0.60	0.68	0.90	0.98	0.99	1.00	0.99	1.00	1.00	1.00
35	0.65	0.79	0.96	0.94	0.98	0.97	0.99	1.00	1.00	1.00
36	0.45	0.58	0.82	0.81	0.88	0.87	0.97	0.98	0.99	1.00
37	0.04	0.07	0.25	0.36	0.61	0.80	0.86	0.95	0.97	1.00
38	0.02	0.07	0.15	0.38	0.60	0.82	0.89	0.94	0.99	1.00
39	0.04	0.10	0.17	0.28	0.42	0.61	0.79	0.89	0.98	1.00
40	0.11	0.31	0.35	0.62	0.79	0.92	0.97	0.97	0.99	1.00
41	0.12	0.18	0.32	0.57	0.72	0.90	0.97	1.00	1.00	1.00
42	0.08	0.28	0.31	0.50	0.63	0.73	0.93	0.95	1.00	1.00

Table 4: POD according to each scenario and each k2 value, k1 = 3

	k2=1	k2=2	k2=3	k2=4	k2=5	k2=6	k2=7	k2=8	k2=9	k2=10
1	0.01	0.01	0.02	0.12	0.24	0.19	0.46	0.52	0.60	0.74
2	0.00	0.00	0.04	0.11	0.17	0.37	0.47	0.57	0.68	0.74
3	0.02	0.04	0.10	0.20	0.16	0.11	0.25	0.35	0.38	0.45
4	0.32	0.50	0.60	0.74	0.85	0.89	0.96	0.99	1.00	1.00
5	0.31	0.46	0.69	0.73	0.85	0.94	0.98	0.97	1.00	0.99
6	0.13	0.36	0.33	0.48	0.39	0.44	0.61	0.63	0.67	0.68
7	0.01	0.01	0.00	0.02	0.08	0.02	0.08	0.11	0.14	0.13
8	0.02	0.01	0.00	0.01	0.00	0.08	0.07	0.09	0.15	0.14
9	0.02	0.02	0.04	0.03	0.02	0.07	0.03	0.12	0.09	0.09
10	0.17	0.40	0.57	0.64	0.81	0.83	0.92	0.97	0.98	0.97
11	0.27	0.37	0.48	0.71	0.79	0.81	0.93	0.97	0.97	0.97
12	0.25	0.44	0.49	0.60	0.72	0.80	0.87	0.94	0.91	0.99
13	0.02	0.06	0.14	0.24	0.43	0.59	0.70	0.82	0.92	0.93
14	0.01	0.04	0.13	0.23	0.42	0.50	0.75	0.87	0.94	0.94
15	0.04	0.08	0.16	0.29	0.22	0.34	0.36	0.67	0.62	0.74
16	0.66	0.82	0.92	0.99	0.98	0.99	1.00	1.00	1.00	1.00
17	0.67	0.81	0.92	0.93	0.98	1.00	1.00	1.00	1.00	1.00
18	0.49	0.53	0.65	0.57	0.66	0.70	0.77	0.82	0.89	0.94
19	0.00	0.00	0.01	0.02	0.00	0.05	0.10	0.15	0.11	0.28
20	0.00	0.00	0.04	0.06	0.07	0.08	0.18	0.27	0.33	0.42
21	0.00	0.01	0.01	0.05	0.02	0.09	0.19	0.14	0.16	0.22
22	0.01	0.10	0.17	0.31	0.34	0.53	0.69	0.79	0.91	0.96
23	0.10	0.13	0.20	0.32	0.46	0.56	0.72	0.81	0.92	0.94
24	0.11	0.12	0.16	0.23	0.35	0.39	0.44	0.54	0.60	0.71
25	0.02	0.02	0.06	0.13	0.21	0.47	0.61	0.87	0.91	0.96
26	0.01	0.07	0.10	0.17	0.40	0.51	0.65	0.79	0.82	0.83
27	0.03	0.07	0.11	0.13	0.17	0.15	0.27	0.27	0.23	0.20
28	0.17	0.36	0.44	0.66	0.80	0.87	0.96	0.99	0.99	1.00
29	0.17	0.38	0.58	0.66	0.74	0.88	0.94	0.98	1.00	1.00
30	0.06	0.11	0.23	0.19	0.25	0.26	0.29	0.35	0.34	0.38
31	0.02	0.07	0.25	0.19	0.44	0.67	0.88	0.93	1.00	0.98
32	0.01	0.07	0.14	0.34	0.51	0.67	0.84	0.92	0.99	0.99
33	0.05	0.10	0.14	0.16	0.37	0.46	0.61	0.65	0.84	0.89
34	0.50	0.81	0.89	0.93	0.99	1.00	1.00	1.00	1.00	1.00
35	0.63	0.80	0.89	0.96	0.98	0.99	0.99	1.00	1.00	1.00
36	0.50	0.56	0.73	0.83	0.88	0.89	0.92	0.97	0.98	0.99
37	0.02	0.03	0.17	0.35	0.53	0.76	0.88	0.96	0.95	0.99
38	0.01	0.07	0.10	0.26	0.63	0.73	0.86	0.96	1.00	1.00
39	0.02	0.04	0.14	0.20	0.35	0.61	0.74	0.87	0.95	1.00
40	0.11	0.17	0.39	0.50	0.69	0.84	0.96	0.96	1.00	1.00
41	0.12	0.16	0.42	0.54	0.63	0.85	0.98	1.00	0.99	1.00
42	0.09	0.14	0.23	0.53	0.68	0.74	0.91	0.93	0.97	1.00

Table 5: POD according to each scenario and each k2 value, k1 = 5

	k2=1	k2=2	k2=3	k2=4	k2=5	k2=6	k2=7	k2=8	k2=9	k2=10
1	0.01	0.01	0.03	0.08	0.12	0.16	0.27	0.37	0.46	0.66
2	0.00	0.04	0.05	0.05	0.20	0.27	0.33	0.46	0.64	0.71
3	0.07	0.01	0.06	0.08	0.09	0.20	0.24	0.29	0.42	0.41
4	0.31	0.31	0.53	0.68	0.73	0.82	0.93	0.94	0.99	0.98
5	0.25	0.44	0.50	0.61	0.76	0.92	0.93	0.96	0.99	1.00
6	0.18	0.22	0.33	0.39	0.34	0.44	0.48	0.47	0.62	0.72
7	0.01	0.01	0.03	0.03	0.01	0.05	0.02	0.03	0.08	0.13
8	0.00	0.00	0.00	0.01	0.02	0.02	0.06	0.05	0.11	0.14
9	0.01	0.02	0.00	0.01	0.03	0.03	0.02	0.10	0.13	0.10
10	0.18	0.32	0.48	0.50	0.67	0.83	0.82	0.87	0.95	0.93
11	0.29	0.38	0.42	0.53	0.74	0.73	0.90	0.91	0.97	0.98
12	0.22	0.39	0.43	0.54	0.63	0.74	0.81	0.90	0.97	0.96
13	0.01	0.02	0.06	0.15	0.23	0.45	0.53	0.70	0.84	0.91
14	0.00	0.03	0.07	0.11	0.31	0.38	0.60	0.74	0.89	0.87
15	0.01	0.05	0.15	0.14	0.22	0.36	0.43	0.48	0.50	0.66
16	0.64	0.85	0.90	0.96	0.97	0.98	1.00	1.00	1.00	1.00
17	0.71	0.82	0.84	0.96	0.98	0.99	1.00	0.99	0.99	0.99
18	0.44	0.50	0.56	0.63	0.68	0.70	0.84	0.81	0.86	0.86
19	0.00	0.00	0.00	0.01	0.02	0.05	0.10	0.06	0.15	0.20
20	0.00	0.00	0.00	0.01	0.04	0.04	0.11	0.16	0.30	0.33
21	0.00	0.01	0.04	0.01	0.07	0.06	0.12	0.12	0.21	0.20
22	0.04	0.11	0.16	0.24	0.30	0.44	0.62	0.66	0.83	0.86
23	0.04	0.08	0.16	0.28	0.36	0.39	0.57	0.70	0.86	0.88
24	0.01	0.04	0.11	0.31	0.27	0.28	0.45	0.47	0.58	0.63
25	0.00	0.02	0.03	0.05	0.14	0.34	0.46	0.66	0.83	0.95
26	0.04	0.03	0.08	0.15	0.23	0.37	0.55	0.69	0.77	0.83
27	0.03	0.02	0.04	0.10	0.09	0.08	0.16	0.23	0.17	0.22
28	0.13	0.22	0.33	0.54	0.71	0.87	0.96	1.00	1.00	1.00
29	0.15	0.26	0.45	0.57	0.70	0.83	0.93	0.94	0.97	0.99
30	0.10	0.17	0.15	0.22	0.24	0.28	0.30	0.38	0.33	0.37
31	0.01	0.01	0.07	0.14	0.40	0.51	0.73	0.91	0.96	0.99
32	0.03	0.02	0.07	0.10	0.35	0.56	0.71	0.88	0.99	1.00
33	0.01	0.09	0.08	0.25	0.28	0.34	0.48	0.68	0.78	0.89
34	0.54	0.77	0.85	0.97	0.96	0.98	0.97	0.99	1.00	1.00
35	0.54	0.78	0.92	0.93	0.95	1.00	1.00	1.00	1.00	1.00
36	0.43	0.51	0.68	0.70	0.78	0.84	0.93	0.99	0.99	1.00
37	0.01	0.01	0.05	0.17	0.35	0.64	0.75	0.91	0.95	1.00
38	0.01	0.02	0.08	0.19	0.29	0.59	0.74	0.94	0.95	1.00
39	0.00	0.02	0.04	0.15	0.35	0.50	0.67	0.85	0.90	0.94
40	0.08	0.09	0.18	0.45	0.58	0.70	0.88	0.90	1.00	1.00
41	0.04	0.15	0.21	0.42	0.66	0.72	0.88	0.94	0.98	1.00
42	0.03	0.12	0.18	0.50	0.45	0.71	0.80	0.89	0.99	0.99

Table 6: POD according to each scenario and each k2 value, k1 = 10
