# Supplementary material for: Evaluation and comparison of statistical methods for early temporal detection of outbreaks: A simulation-based study
Source: PLoS One. 2017 Jul 17;12(7):e0181227. doi: 10.1371/journal.pone.0181227 (PMC5513450; doi:10.1371/journal.pone.0181227)
Supplement: S18 Appendix — (PDF) [file pone.0181227.s018.pdf]

# Evaluation and Comparison of Statistical Methods for Early Temporal Detection of Outbreaks: a Simulation-Based Study

## Appendix S18: Overall performances of GLR Poisson algorithm

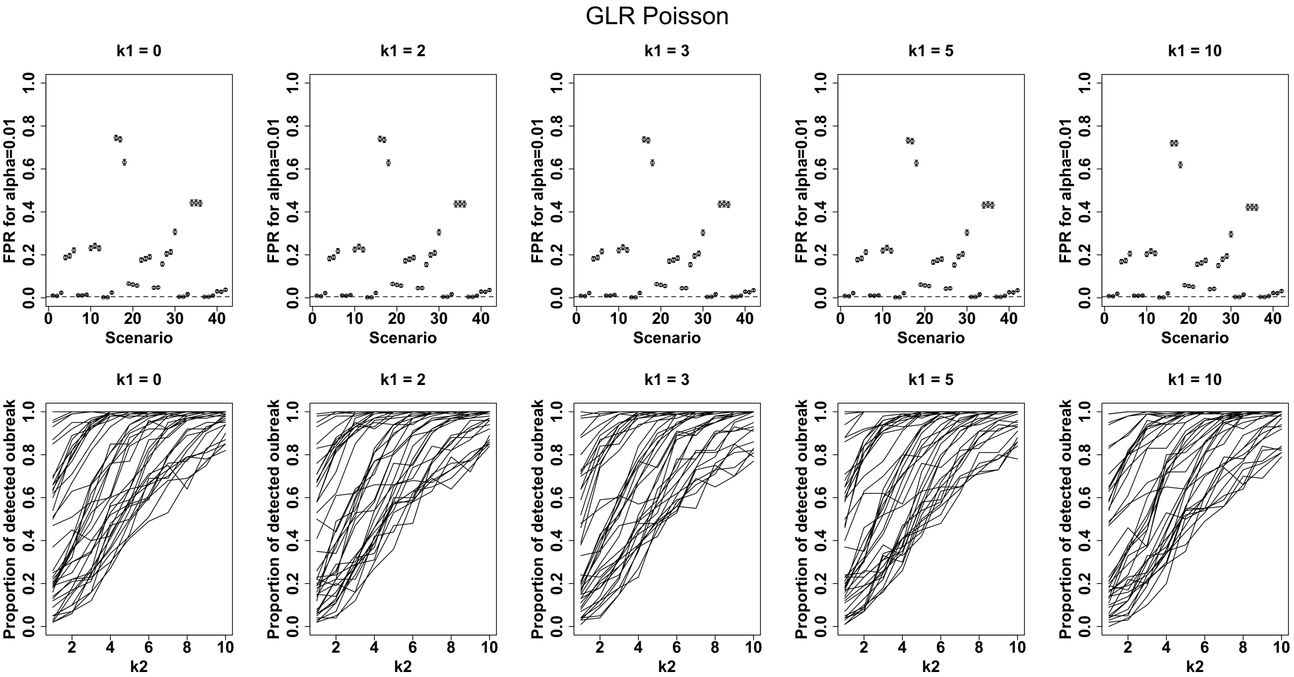


Figure 1: GLR Poisson algorithm performances by increasing past outbreak amplitude $k_1 = 0$, 2, 3, 5 or 10 with (i) on the first row: false positive rate for 42 simulated scenarios, (ii) on the second row: probability of detection for 42 simulated scenarios (each curve corresponding to a scenario) by increasing current outbreak amplitude $k_2 = 1$ to 10.

## Overall performances of GLR Poisson algorithm

| | FPR k1=0 | FPR k1=2 | FPR k1=3 | FPR k1=5 | FPR k1=10 |
|---|---|---|---|---|---|
| 1 | 0.01 | 0.01 | 0.01 | 0.01 | 0.01 |
| 2 | 0.01 | 0.01 | 0.01 | 0.01 | 0.01 |
| 3 | 0.02 | 0.02 | 0.02 | 0.02 | 0.02 |
| 4 | 0.19 | 0.18 | 0.18 | 0.18 | 0.17 |
| 5 | 0.20 | 0.19 | 0.19 | 0.18 | 0.17 |
| 6 | 0.22 | 0.22 | 0.22 | 0.21 | 0.21 |
| 7 | 0.01 | 0.01 | 0.01 | 0.01 | 0.01 |
| 8 | 0.01 | 0.01 | 0.01 | 0.01 | 0.01 |
| 9 | 0.01 | 0.01 | 0.01 | 0.01 | 0.01 |
| 10 | 0.23 | 0.22 | 0.22 | 0.22 | 0.20 |
| 11 | 0.24 | 0.24 | 0.24 | 0.23 | 0.22 |
| 12 | 0.23 | 0.22 | 0.22 | 0.22 | 0.21 |
| 13 | 0.00 | 0.00 | 0.00 | 0.00 | 0.00 |
| 14 | 0.00 | 0.00 | 0.00 | 0.00 | 0.00 |
| 15 | 0.02 | 0.02 | 0.02 | 0.02 | 0.02 |
| 16 | 0.74 | 0.74 | 0.74 | 0.73 | 0.72 |
| 17 | 0.74 | 0.74 | 0.73 | 0.73 | 0.72 |
| 18 | 0.63 | 0.63 | 0.63 | 0.63 | 0.62 |
| 19 | 0.07 | 0.06 | 0.06 | 0.06 | 0.06 |
| 20 | 0.06 | 0.06 | 0.06 | 0.06 | 0.05 |
| 21 | 0.06 | 0.06 | 0.06 | 0.05 | 0.05 |
| 22 | 0.18 | 0.17 | 0.17 | 0.17 | 0.16 |
| 23 | 0.18 | 0.18 | 0.18 | 0.17 | 0.16 |
| 24 | 0.19 | 0.19 | 0.18 | 0.18 | 0.17 |
| 25 | 0.05 | 0.05 | 0.04 | 0.04 | 0.04 |
| 26 | 0.05 | 0.05 | 0.04 | 0.04 | 0.04 |
| 27 | 0.16 | 0.15 | 0.15 | 0.15 | 0.15 |
| 28 | 0.20 | 0.20 | 0.19 | 0.19 | 0.18 |
| 29 | 0.21 | 0.21 | 0.21 | 0.20 | 0.19 |
| 30 | 0.31 | 0.30 | 0.30 | 0.30 | 0.30 |
| 31 | 0.00 | 0.00 | 0.00 | 0.00 | 0.00 |
| 32 | 0.00 | 0.00 | 0.00 | 0.00 | 0.00 |
| 33 | 0.02 | 0.02 | 0.02 | 0.02 | 0.01 |
| 34 | 0.44 | 0.44 | 0.44 | 0.43 | 0.42 |
| 35 | 0.44 | 0.44 | 0.44 | 0.43 | 0.42 |
| 36 | 0.44 | 0.44 | 0.43 | 0.43 | 0.42 |
| 37 | 0.00 | 0.00 | 0.00 | 0.00 | 0.00 |
| 38 | 0.00 | 0.00 | 0.00 | 0.00 | 0.00 |
| 39 | 0.01 | 0.01 | 0.01 | 0.01 | 0.01 |
| 40 | 0.03 | 0.03 | 0.03 | 0.03 | 0.02 |
| 41 | 0.03 | 0.03 | 0.03 | 0.02 | 0.02 |
| 42 | 0.04 | 0.04 | 0.04 | 0.03 | 0.03 |

Table 1: FPR according to each scenario and each k1 value

| | k2=1 | k2=2 | k2=3 | k2=4 | k2=5 | k2=6 | k2=7 | k2=8 | k2=9 | k2=10 |
|---|---|---|---|---|---|---|---|---|---|---|
| 1 | 0.13 | 0.22 | 0.25 | 0.36 | 0.50 | 0.61 | 0.79 | 0.91 | 0.95 | 0.95 |
| 2 | 0.12 | 0.17 | 0.21 | 0.48 | 0.56 | 0.59 | 0.80 | 0.89 | 0.91 | 0.99 |
| 3 | 0.19 | 0.22 | 0.23 | 0.30 | 0.41 | 0.49 | 0.53 | 0.68 | 0.77 | 0.85 |
| 4 | 0.66 | 0.84 | 0.93 | 0.99 | 0.97 | 1.00 | 0.99 | 1.00 | 1.00 | 1.00 |
| 5 | 0.62 | 0.71 | 0.85 | 0.98 | 0.98 | 0.98 | 1.00 | 1.00 | 0.99 | 1.00 |
| 6 | 0.51 | 0.63 | 0.66 | 0.76 | 0.77 | 0.96 | 0.92 | 0.96 | 0.98 | 0.98 |
| 7 | 0.21 | 0.25 | 0.40 | 0.42 | 0.59 | 0.66 | 0.70 | 0.79 | 0.78 | 0.82 |
| 8 | 0.25 | 0.30 | 0.34 | 0.56 | 0.59 | 0.71 | 0.75 | 0.64 | 0.83 | 0.87 |
| 9 | 0.18 | 0.30 | 0.35 | 0.47 | 0.50 | 0.67 | 0.73 | 0.79 | 0.81 | 0.85 |
| 10 | 0.70 | 0.82 | 0.92 | 0.98 | 0.99 | 0.99 | 1.00 | 1.00 | 1.00 | 1.00 |
| 11 | 0.69 | 0.89 | 0.92 | 0.99 | 1.00 | 1.00 | 1.00 | 1.00 | 1.00 | 1.00 |
| 12 | 0.64 | 0.79 | 0.95 | 0.99 | 1.00 | 1.00 | 1.00 | 1.00 | 1.00 | 1.00 |
| 13 | 0.02 | 0.07 | 0.12 | 0.29 | 0.49 | 0.60 | 0.73 | 0.84 | 0.93 | 0.96 |
| 14 | 0.05 | 0.07 | 0.16 | 0.27 | 0.43 | 0.69 | 0.69 | 0.82 | 0.92 | 0.97 |
| 15 | 0.14 | 0.21 | 0.22 | 0.34 | 0.42 | 0.51 | 0.66 | 0.78 | 0.82 | 0.94 |
| 16 | 1.00 | 1.00 | 1.00 | 1.00 | 1.00 | 1.00 | 1.00 | 1.00 | 1.00 | 1.00 |
| 17 | 0.96 | 1.00 | 1.00 | 1.00 | 1.00 | 1.00 | 1.00 | 1.00 | 1.00 | 1.00 |
| 18 | 0.87 | 0.95 | 0.95 | 1.00 | 1.00 | 0.99 | 1.00 | 1.00 | 1.00 | 1.00 |
| 19 | 0.20 | 0.37 | 0.55 | 0.75 | 0.94 | 0.99 | 1.00 | 1.00 | 1.00 | 1.00 |
| 20 | 0.23 | 0.36 | 0.62 | 0.75 | 0.90 | 0.94 | 0.99 | 0.98 | 1.00 | 1.00 |
| 21 | 0.19 | 0.35 | 0.53 | 0.61 | 0.88 | 0.92 | 0.92 | 0.98 | 1.00 | 1.00 |
| 22 | 0.54 | 0.82 | 0.96 | 0.99 | 1.00 | 1.00 | 1.00 | 1.00 | 1.00 | 1.00 |
| 23 | 0.60 | 0.80 | 0.94 | 1.00 | 1.00 | 1.00 | 1.00 | 1.00 | 1.00 | 1.00 |
| 24 | 0.50 | 0.67 | 0.82 | 0.93 | 0.99 | 1.00 | 1.00 | 1.00 | 1.00 | 1.00 |
| 25 | 0.26 | 0.41 | 0.67 | 0.81 | 0.91 | 0.97 | 1.00 | 1.00 | 1.00 | 1.00 |
| 26 | 0.22 | 0.32 | 0.66 | 0.81 | 0.94 | 0.98 | 0.99 | 1.00 | 1.00 | 1.00 |
| 27 | 0.37 | 0.45 | 0.40 | 0.52 | 0.57 | 0.60 | 0.68 | 0.69 | 0.75 | 0.90 |
| 28 | 0.63 | 0.87 | 0.96 | 0.99 | 1.00 | 1.00 | 1.00 | 1.00 | 1.00 | 1.00 |
| 29 | 0.66 | 0.77 | 0.93 | 0.96 | 1.00 | 0.99 | 1.00 | 1.00 | 1.00 | 1.00 |
| 30 | 0.47 | 0.51 | 0.58 | 0.60 | 0.64 | 0.66 | 0.76 | 0.77 | 0.90 | 0.94 |
| 31 | 0.05 | 0.10 | 0.16 | 0.43 | 0.58 | 0.81 | 0.92 | 0.97 | 0.97 | 0.99 |
| 32 | 0.02 | 0.06 | 0.25 | 0.37 | 0.54 | 0.79 | 0.89 | 0.97 | 0.98 | 1.00 |
| 33 | 0.09 | 0.16 | 0.27 | 0.41 | 0.57 | 0.70 | 0.80 | 0.92 | 0.92 | 0.98 |
| 34 | 0.93 | 0.99 | 1.00 | 0.99 | 1.00 | 1.00 | 1.00 | 1.00 | 1.00 | 1.00 |
| 35 | 0.95 | 0.99 | 1.00 | 1.00 | 1.00 | 1.00 | 1.00 | 1.00 | 1.00 | 1.00 |
| 36 | 0.85 | 0.92 | 0.97 | 0.99 | 1.00 | 1.00 | 1.00 | 1.00 | 1.00 | 1.00 |
| 37 | 0.02 | 0.15 | 0.26 | 0.57 | 0.67 | 0.77 | 0.95 | 0.97 | 0.99 | 1.00 |
| 38 | 0.05 | 0.10 | 0.24 | 0.46 | 0.64 | 0.83 | 0.93 | 0.99 | 1.00 | 0.99 |
| 39 | 0.03 | 0.18 | 0.35 | 0.53 | 0.64 | 0.87 | 0.88 | 0.96 | 0.98 | 1.00 |
| 40 | 0.16 | 0.41 | 0.58 | 0.85 | 0.85 | 0.95 | 0.98 | 1.00 | 1.00 | 1.00 |
| 41 | 0.18 | 0.35 | 0.49 | 0.75 | 0.85 | 0.95 | 0.97 | 0.99 | 1.00 | 1.00 |
| 42 | 0.19 | 0.36 | 0.45 | 0.60 | 0.84 | 0.89 | 0.98 | 0.98 | 1.00 | 1.00 |

Table 2: POD according to each scenario and each k2 value, k1 = 0

| | k2=1 | k2=2 | k2=3 | k2=4 | k2=5 | k2=6 | k2=7 | k2=8 | k2=9 | k2=10 |
|---|---|---|---|---|---|---|---|---|---|---|
| 1 | 0.08 | 0.16 | 0.29 | 0.31 | 0.60 | 0.60 | 0.77 | 0.88 | 0.88 | 0.92 |
| 2 | 0.07 | 0.22 | 0.22 | 0.39 | 0.57 | 0.63 | 0.70 | 0.82 | 0.87 | 0.96 |
| 3 | 0.15 | 0.20 | 0.16 | 0.42 | 0.44 | 0.62 | 0.69 | 0.64 | 0.74 | 0.84 |
| 4 | 0.68 | 0.83 | 0.90 | 0.97 | 1.00 | 1.00 | 0.99 | 1.00 | 1.00 | 1.00 |
| 5 | 0.65 | 0.81 | 0.91 | 0.95 | 0.94 | 0.99 | 0.99 | 1.00 | 1.00 | 1.00 |
| 6 | 0.41 | 0.61 | 0.62 | 0.77 | 0.80 | 0.89 | 0.91 | 0.95 | 0.99 | 0.99 |
| 7 | 0.14 | 0.29 | 0.30 | 0.46 | 0.58 | 0.58 | 0.71 | 0.76 | 0.77 | 0.85 |
| 8 | 0.23 | 0.24 | 0.29 | 0.39 | 0.57 | 0.59 | 0.68 | 0.73 | 0.78 | 0.86 |
| 9 | 0.22 | 0.19 | 0.27 | 0.41 | 0.57 | 0.63 | 0.65 | 0.69 | 0.78 | 0.87 |
| 10 | 0.71 | 0.88 | 0.94 | 1.00 | 1.00 | 1.00 | 1.00 | 1.00 | 1.00 | 1.00 |
| 11 | 0.71 | 0.87 | 0.93 | 0.93 | 1.00 | 1.00 | 1.00 | 1.00 | 1.00 | 1.00 |
| 12 | 0.76 | 0.84 | 0.95 | 0.92 | 0.98 | 1.00 | 0.99 | 1.00 | 1.00 | 1.00 |
| 13 | 0.02 | 0.05 | 0.12 | 0.30 | 0.47 | 0.48 | 0.77 | 0.89 | 0.91 | 0.97 |
| 14 | 0.04 | 0.04 | 0.16 | 0.27 | 0.36 | 0.59 | 0.76 | 0.86 | 0.93 | 0.97 |
| 15 | 0.15 | 0.14 | 0.32 | 0.34 | 0.53 | 0.58 | 0.63 | 0.79 | 0.72 | 0.89 |
| 16 | 0.99 | 1.00 | 1.00 | 1.00 | 1.00 | 1.00 | 1.00 | 1.00 | 1.00 | 1.00 |
| 17 | 0.98 | 0.99 | 1.00 | 1.00 | 1.00 | 1.00 | 1.00 | 1.00 | 1.00 | 1.00 |
| 18 | 0.85 | 0.93 | 0.99 | 1.00 | 1.00 | 1.00 | 1.00 | 1.00 | 1.00 | 1.00 |
| 19 | 0.26 | 0.39 | 0.56 | 0.81 | 0.94 | 0.97 | 1.00 | 1.00 | 1.00 | 1.00 |
| 20 | 0.19 | 0.37 | 0.60 | 0.81 | 0.89 | 0.97 | 0.99 | 1.00 | 1.00 | 1.00 |
| 21 | 0.19 | 0.26 | 0.47 | 0.58 | 0.81 | 0.90 | 0.96 | 0.97 | 0.99 | 1.00 |
| 22 | 0.58 | 0.82 | 0.91 | 1.00 | 0.99 | 1.00 | 1.00 | 1.00 | 1.00 | 1.00 |
| 23 | 0.58 | 0.72 | 0.97 | 1.00 | 1.00 | 1.00 | 1.00 | 1.00 | 1.00 | 1.00 |
| 24 | 0.53 | 0.62 | 0.85 | 0.96 | 0.97 | 0.98 | 0.99 | 1.00 | 1.00 | 1.00 |
| 25 | 0.16 | 0.39 | 0.51 | 0.83 | 0.94 | 0.94 | 1.00 | 1.00 | 1.00 | 1.00 |
| 26 | 0.22 | 0.45 | 0.64 | 0.71 | 0.92 | 1.00 | 1.00 | 0.99 | 1.00 | 1.00 |
| 27 | 0.35 | 0.34 | 0.53 | 0.54 | 0.66 | 0.68 | 0.67 | 0.73 | 0.81 | 0.85 |
| 28 | 0.58 | 0.83 | 0.93 | 0.99 | 1.00 | 1.00 | 1.00 | 1.00 | 1.00 | 1.00 |
| 29 | 0.67 | 0.86 | 0.93 | 0.97 | 0.98 | 1.00 | 1.00 | 1.00 | 1.00 | 1.00 |
| 30 | 0.50 | 0.44 | 0.63 | 0.66 | 0.66 | 0.69 | 0.84 | 0.82 | 0.92 | 0.94 |
| 31 | 0.06 | 0.12 | 0.24 | 0.41 | 0.58 | 0.78 | 0.92 | 0.96 | 0.97 | 1.00 |
| 32 | 0.02 | 0.11 | 0.28 | 0.37 | 0.65 | 0.76 | 0.90 | 0.97 | 0.97 | 0.98 |
| 33 | 0.06 | 0.22 | 0.33 | 0.44 | 0.54 | 0.74 | 0.78 | 0.85 | 0.96 | 0.96 |
| 34 | 0.95 | 1.00 | 0.99 | 1.00 | 1.00 | 1.00 | 1.00 | 1.00 | 1.00 | 1.00 |
| 35 | 0.93 | 0.98 | 0.98 | 1.00 | 1.00 | 1.00 | 1.00 | 1.00 | 1.00 | 1.00 |
| 36 | 0.83 | 0.85 | 1.00 | 1.00 | 1.00 | 1.00 | 1.00 | 1.00 | 1.00 | 1.00 |
| 37 | 0.06 | 0.11 | 0.27 | 0.54 | 0.76 | 0.75 | 0.90 | 0.97 | 0.99 | 1.00 |
| 38 | 0.03 | 0.07 | 0.28 | 0.50 | 0.68 | 0.91 | 0.96 | 0.98 | 0.99 | 1.00 |
| 39 | 0.05 | 0.13 | 0.32 | 0.46 | 0.62 | 0.84 | 0.92 | 0.97 | 0.98 | 1.00 |
| 40 | 0.12 | 0.49 | 0.54 | 0.73 | 0.90 | 0.95 | 0.98 | 1.00 | 0.98 | 1.00 |
| 41 | 0.18 | 0.22 | 0.59 | 0.77 | 0.82 | 0.97 | 0.99 | 1.00 | 1.00 | 1.00 |
| 42 | 0.20 | 0.25 | 0.50 | 0.59 | 0.81 | 0.90 | 0.97 | 0.98 | 1.00 | 1.00 |

Table 3: POD according to each scenario and each k2 value, k1 = 2

| | k2=1 | k2=2 | k2=3 | k2=4 | k2=5 | k2=6 | k2=7 | k2=8 | k2=9 | k2=10 |
|---|---|---|---|---|---|---|---|---|---|---|
| 1 | 0.07 | 0.12 | 0.23 | 0.44 | 0.57 | 0.67 | 0.78 | 0.91 | 0.91 | 0.93 |
| 2 | 0.08 | 0.20 | 0.24 | 0.38 | 0.49 | 0.66 | 0.82 | 0.82 | 0.93 | 0.91 |
| 3 | 0.11 | 0.21 | 0.28 | 0.37 | 0.50 | 0.59 | 0.62 | 0.73 | 0.83 | 0.86 |
| 4 | 0.70 | 0.76 | 0.90 | 0.93 | 0.99 | 1.00 | 1.00 | 1.00 | 1.00 | 1.00 |
| 5 | 0.59 | 0.84 | 0.85 | 0.99 | 0.98 | 0.99 | 0.99 | 1.00 | 1.00 | 1.00 |
| 6 | 0.38 | 0.59 | 0.60 | 0.77 | 0.74 | 0.90 | 0.93 | 0.95 | 0.98 | 1.00 |
| 7 | 0.13 | 0.26 | 0.31 | 0.38 | 0.47 | 0.53 | 0.73 | 0.75 | 0.70 | 0.83 |
| 8 | 0.24 | 0.23 | 0.32 | 0.48 | 0.48 | 0.56 | 0.69 | 0.65 | 0.70 | 0.77 |
| 9 | 0.18 | 0.27 | 0.35 | 0.43 | 0.49 | 0.56 | 0.55 | 0.76 | 0.81 | 0.82 |
| 10 | 0.72 | 0.89 | 0.95 | 0.97 | 0.98 | 1.00 | 1.00 | 1.00 | 1.00 | 1.00 |
| 11 | 0.71 | 0.86 | 0.94 | 0.99 | 1.00 | 1.00 | 1.00 | 1.00 | 1.00 | 1.00 |
| 12 | 0.73 | 0.80 | 0.89 | 0.97 | 0.99 | 0.99 | 0.99 | 1.00 | 1.00 | 1.00 |
| 13 | 0.01 | 0.09 | 0.18 | 0.23 | 0.48 | 0.54 | 0.74 | 0.79 | 0.95 | 0.98 |
| 14 | 0.04 | 0.05 | 0.15 | 0.27 | 0.45 | 0.61 | 0.74 | 0.90 | 0.91 | 0.95 |
| 15 | 0.13 | 0.18 | 0.28 | 0.37 | 0.42 | 0.58 | 0.66 | 0.71 | 0.85 | 0.79 |
| 16 | 1.00 | 0.99 | 1.00 | 1.00 | 1.00 | 1.00 | 1.00 | 1.00 | 1.00 | 1.00 |
| 17 | 0.98 | 1.00 | 1.00 | 1.00 | 1.00 | 1.00 | 1.00 | 1.00 | 1.00 | 1.00 |
| 18 | 0.88 | 0.94 | 0.99 | 0.98 | 1.00 | 1.00 | 1.00 | 1.00 | 1.00 | 1.00 |
| 19 | 0.20 | 0.40 | 0.57 | 0.77 | 0.88 | 0.94 | 0.98 | 0.99 | 1.00 | 1.00 |
| 20 | 0.21 | 0.42 | 0.60 | 0.75 | 0.90 | 0.94 | 1.00 | 1.00 | 1.00 | 1.00 |
| 21 | 0.17 | 0.36 | 0.53 | 0.66 | 0.75 | 0.91 | 0.89 | 0.98 | 0.99 | 1.00 |
| 22 | 0.52 | 0.80 | 0.94 | 1.00 | 1.00 | 1.00 | 1.00 | 1.00 | 1.00 | 1.00 |
| 23 | 0.46 | 0.75 | 0.92 | 1.00 | 1.00 | 1.00 | 1.00 | 1.00 | 1.00 | 1.00 |
| 24 | 0.39 | 0.74 | 0.82 | 0.94 | 0.96 | 0.99 | 1.00 | 1.00 | 1.00 | 1.00 |
| 25 | 0.16 | 0.38 | 0.58 | 0.84 | 0.96 | 0.97 | 1.00 | 1.00 | 1.00 | 1.00 |
| 26 | 0.19 | 0.34 | 0.62 | 0.79 | 0.92 | 0.99 | 0.97 | 1.00 | 1.00 | 1.00 |
| 27 | 0.33 | 0.35 | 0.47 | 0.49 | 0.56 | 0.62 | 0.68 | 0.73 | 0.74 | 0.81 |
| 28 | 0.64 | 0.87 | 0.90 | 0.99 | 1.00 | 1.00 | 1.00 | 1.00 | 1.00 | 1.00 |
| 29 | 0.64 | 0.78 | 0.94 | 1.00 | 0.99 | 1.00 | 1.00 | 1.00 | 1.00 | 1.00 |
| 30 | 0.48 | 0.55 | 0.61 | 0.57 | 0.60 | 0.72 | 0.81 | 0.81 | 0.85 | 0.92 |
| 31 | 0.03 | 0.12 | 0.18 | 0.35 | 0.59 | 0.89 | 0.89 | 0.98 | 0.99 | 1.00 |
| 32 | 0.03 | 0.04 | 0.15 | 0.36 | 0.64 | 0.73 | 0.93 | 0.97 | 0.98 | 1.00 |
| 33 | 0.15 | 0.18 | 0.31 | 0.45 | 0.53 | 0.78 | 0.78 | 0.89 | 0.96 | 1.00 |
| 34 | 0.89 | 0.99 | 1.00 | 1.00 | 1.00 | 1.00 | 1.00 | 1.00 | 1.00 | 1.00 |
| 35 | 0.97 | 0.98 | 1.00 | 1.00 | 1.00 | 1.00 | 1.00 | 1.00 | 1.00 | 1.00 |
| 36 | 0.80 | 0.91 | 0.99 | 1.00 | 1.00 | 1.00 | 1.00 | 1.00 | 1.00 | 1.00 |
| 37 | 0.04 | 0.14 | 0.32 | 0.48 | 0.70 | 0.87 | 0.91 | 0.98 | 0.98 | 1.00 |
| 38 | 0.04 | 0.10 | 0.22 | 0.52 | 0.68 | 0.89 | 0.92 | 0.95 | 0.99 | 1.00 |
| 39 | 0.09 | 0.22 | 0.31 | 0.50 | 0.59 | 0.76 | 0.91 | 0.95 | 1.00 | 1.00 |
| 40 | 0.20 | 0.40 | 0.51 | 0.74 | 0.85 | 0.99 | 0.99 | 0.99 | 1.00 | 1.00 |
| 41 | 0.15 | 0.29 | 0.42 | 0.68 | 0.82 | 0.95 | 0.98 | 1.00 | 1.00 | 1.00 |
| 42 | 0.20 | 0.43 | 0.52 | 0.68 | 0.86 | 0.93 | 0.99 | 1.00 | 1.00 | 1.00 |

Table 4: POD according to each scenario and each k2 value, k1 = 31

| | k2=1 | k2=2 | k2=3 | k2=4 | k2=5 | k2=6 | k2=7 | k2=8 | k2=9 | k2=10 |
|---|---|---|---|---|---|---|---|---|---|---|
| 1 | 0.07 | 0.17 | 0.26 | 0.34 | 0.58 | 0.59 | 0.83 | 0.91 | 0.91 | 0.96 |
| 2 | 0.11 | 0.17 | 0.25 | 0.37 | 0.44 | 0.67 | 0.79 | 0.85 | 0.89 | 0.93 |
| 3 | 0.17 | 0.19 | 0.28 | 0.41 | 0.47 | 0.52 | 0.69 | 0.71 | 0.73 | 0.88 |
| 4 | 0.65 | 0.78 | 0.90 | 0.92 | 1.00 | 0.99 | 1.00 | 1.00 | 1.00 | 1.00 |
| 5 | 0.60 | 0.78 | 0.94 | 0.93 | 0.94 | 0.99 | 1.00 | 1.00 | 1.00 | 1.00 |
| 6 | 0.40 | 0.63 | 0.70 | 0.80 | 0.79 | 0.88 | 0.93 | 0.94 | 0.97 | 1.00 |
| 7 | 0.16 | 0.26 | 0.25 | 0.47 | 0.54 | 0.58 | 0.70 | 0.74 | 0.78 | 0.86 |
| 8 | 0.23 | 0.23 | 0.31 | 0.41 | 0.49 | 0.56 | 0.64 | 0.76 | 0.78 | 0.84 |
| 9 | 0.24 | 0.25 | 0.38 | 0.33 | 0.42 | 0.63 | 0.65 | 0.72 | 0.80 | 0.78 |
| 10 | 0.65 | 0.87 | 0.96 | 0.98 | 1.00 | 1.00 | 1.00 | 1.00 | 1.00 | 1.00 |
| 11 | 0.71 | 0.78 | 0.93 | 0.98 | 1.00 | 1.00 | 1.00 | 1.00 | 1.00 | 1.00 |
| 12 | 0.62 | 0.81 | 0.94 | 0.96 | 0.98 | 0.99 | 1.00 | 1.00 | 1.00 | 1.00 |
| 13 | 0.03 | 0.10 | 0.16 | 0.29 | 0.52 | 0.65 | 0.74 | 0.88 | 0.94 | 0.93 |
| 14 | 0.01 | 0.07 | 0.17 | 0.26 | 0.50 | 0.57 | 0.77 | 0.88 | 0.96 | 0.95 |
| 15 | 0.12 | 0.19 | 0.23 | 0.41 | 0.45 | 0.48 | 0.62 | 0.81 | 0.81 | 0.91 |
| 16 | 0.99 | 1.00 | 1.00 | 1.00 | 1.00 | 1.00 | 1.00 | 1.00 | 1.00 | 1.00 |
| 17 | 1.00 | 1.00 | 1.00 | 1.00 | 1.00 | 1.00 | 1.00 | 1.00 | 1.00 | 1.00 |
| 18 | 0.88 | 0.92 | 0.94 | 0.98 | 0.98 | 1.00 | 1.00 | 1.00 | 1.00 | 1.00 |
| 19 | 0.18 | 0.46 | 0.52 | 0.74 | 0.90 | 0.98 | 0.95 | 0.99 | 1.00 | 1.00 |
| 20 | 0.20 | 0.30 | 0.64 | 0.80 | 0.93 | 0.99 | 0.99 | 1.00 | 1.00 | 1.00 |
| 21 | 0.19 | 0.31 | 0.43 | 0.63 | 0.83 | 0.87 | 0.97 | 0.99 | 1.00 | 1.00 |
| 22 | 0.46 | 0.84 | 0.96 | 0.98 | 1.00 | 0.99 | 1.00 | 1.00 | 1.00 | 1.00 |
| 23 | 0.47 | 0.81 | 0.91 | 0.95 | 1.00 | 0.99 | 1.00 | 1.00 | 1.00 | 1.00 |
| 24 | 0.59 | 0.70 | 0.77 | 0.91 | 0.99 | 1.00 | 0.99 | 1.00 | 1.00 | 1.00 |
| 25 | 0.21 | 0.40 | 0.55 | 0.78 | 0.92 | 0.99 | 0.99 | 1.00 | 1.00 | 1.00 |
| 26 | 0.18 | 0.36 | 0.60 | 0.77 | 0.93 | 0.97 | 0.99 | 1.00 | 1.00 | 1.00 |
| 27 | 0.37 | 0.35 | 0.52 | 0.56 | 0.63 | 0.65 | 0.65 | 0.72 | 0.73 | 0.86 |
| 28 | 0.58 | 0.87 | 0.98 | 0.99 | 0.97 | 1.00 | 1.00 | 1.00 | 1.00 | 1.00 |
| 29 | 0.68 | 0.84 | 0.91 | 0.97 | 0.99 | 1.00 | 1.00 | 1.00 | 1.00 | 1.00 |
| 30 | 0.50 | 0.62 | 0.62 | 0.57 | 0.76 | 0.74 | 0.82 | 0.83 | 0.92 | 0.94 |
| 31 | 0.04 | 0.07 | 0.33 | 0.30 | 0.55 | 0.78 | 0.91 | 0.95 | 1.00 | 1.00 |
| 32 | 0.04 | 0.13 | 0.22 | 0.45 | 0.58 | 0.78 | 0.91 | 0.96 | 1.00 | 1.00 |
| 33 | 0.13 | 0.23 | 0.31 | 0.35 | 0.57 | 0.69 | 0.76 | 0.91 | 0.96 | 0.99 |
| 34 | 0.88 | 1.00 | 1.00 | 1.00 | 1.00 | 1.00 | 1.00 | 1.00 | 1.00 | 1.00 |
| 35 | 0.94 | 1.00 | 1.00 | 1.00 | 1.00 | 1.00 | 1.00 | 1.00 | 1.00 | 1.00 |
| 36 | 0.86 | 0.91 | 0.94 | 0.99 | 0.99 | 1.00 | 1.00 | 1.00 | 1.00 | 1.00 |
| 37 | 0.04 | 0.08 | 0.25 | 0.45 | 0.66 | 0.85 | 0.93 | 0.97 | 0.97 | 1.00 |
| 38 | 0.01 | 0.09 | 0.18 | 0.38 | 0.72 | 0.88 | 0.94 | 0.99 | 1.00 | 1.00 |
| 39 | 0.05 | 0.09 | 0.34 | 0.37 | 0.63 | 0.80 | 0.88 | 0.99 | 0.99 | 1.00 |
| 40 | 0.17 | 0.36 | 0.57 | 0.71 | 0.79 | 0.91 | 1.00 | 0.99 | 1.00 | 1.00 |
| 41 | 0.14 | 0.31 | 0.60 | 0.70 | 0.84 | 0.94 | 0.99 | 1.00 | 1.00 | 1.00 |
| 42 | 0.23 | 0.32 | 0.45 | 0.68 | 0.87 | 0.91 | 0.96 | 0.99 | 1.00 | 1.00 |

Table 5: POD according to each scenario and each k2 value, k1 = 5

| | k2=1 | k2=2 | k2=3 | k2=4 | k2=5 | k2=6 | k2=7 | k2=8 | k2=9 | k2=10 |
|---|---|---|---|---|---|---|---|---|---|---|
| 1 | 0.14 | 0.16 | 0.21 | 0.34 | 0.47 | 0.61 | 0.79 | 0.78 | 0.87 | 0.92 |
| 2 | 0.10 | 0.14 | 0.21 | 0.30 | 0.56 | 0.70 | 0.76 | 0.80 | 0.87 | 0.97 |
| 3 | 0.17 | 0.19 | 0.24 | 0.35 | 0.38 | 0.49 | 0.56 | 0.70 | 0.69 | 0.79 |
| 4 | 0.63 | 0.74 | 0.83 | 0.94 | 0.95 | 1.00 | 1.00 | 1.00 | 1.00 | 1.00 |
| 5 | 0.64 | 0.80 | 0.85 | 0.91 | 0.98 | 1.00 | 0.99 | 0.99 | 1.00 | 1.00 |
| 6 | 0.47 | 0.56 | 0.67 | 0.71 | 0.78 | 0.85 | 0.95 | 0.92 | 0.98 | 0.98 |
| 7 | 0.14 | 0.20 | 0.31 | 0.41 | 0.52 | 0.54 | 0.61 | 0.64 | 0.82 | 0.83 |
| 8 | 0.16 | 0.23 | 0.25 | 0.40 | 0.55 | 0.55 | 0.74 | 0.66 | 0.84 | 0.82 |
| 9 | 0.19 | 0.17 | 0.31 | 0.37 | 0.54 | 0.55 | 0.59 | 0.73 | 0.76 | 0.81 |
| 10 | 0.65 | 0.81 | 0.92 | 0.95 | 1.00 | 0.99 | 1.00 | 1.00 | 1.00 | 1.00 |
| 11 | 0.71 | 0.80 | 0.96 | 0.94 | 0.99 | 0.99 | 0.99 | 1.00 | 1.00 | 1.00 |
| 12 | 0.65 | 0.80 | 0.89 | 0.95 | 0.99 | 1.00 | 1.00 | 1.00 | 1.00 | 1.00 |
| 13 | 0.02 | 0.03 | 0.14 | 0.29 | 0.35 | 0.61 | 0.70 | 0.77 | 0.92 | 0.99 |
| 14 | 0.00 | 0.04 | 0.10 | 0.20 | 0.51 | 0.60 | 0.75 | 0.83 | 0.93 | 0.93 |
| 15 | 0.09 | 0.12 | 0.33 | 0.34 | 0.44 | 0.53 | 0.65 | 0.76 | 0.77 | 0.84 |
| 16 | 0.99 | 1.00 | 1.00 | 1.00 | 1.00 | 1.00 | 1.00 | 1.00 | 1.00 | 1.00 |
| 17 | 0.99 | 1.00 | 1.00 | 1.00 | 1.00 | 1.00 | 1.00 | 1.00 | 1.00 | 1.00 |
| 18 | 0.84 | 0.89 | 0.99 | 0.97 | 1.00 | 0.99 | 1.00 | 1.00 | 1.00 | 1.00 |
| 19 | 0.13 | 0.37 | 0.47 | 0.68 | 0.89 | 0.96 | 0.97 | 0.99 | 1.00 | 1.00 |
| 20 | 0.23 | 0.36 | 0.45 | 0.76 | 0.84 | 0.93 | 0.99 | 0.99 | 0.99 | 1.00 |
| 21 | 0.18 | 0.35 | 0.50 | 0.72 | 0.72 | 0.86 | 0.91 | 0.95 | 0.97 | 1.00 |
| 22 | 0.54 | 0.81 | 0.94 | 0.98 | 1.00 | 1.00 | 1.00 | 1.00 | 1.00 | 1.00 |
| 23 | 0.55 | 0.71 | 0.93 | 0.95 | 0.99 | 1.00 | 1.00 | 1.00 | 1.00 | 1.00 |
| 24 | 0.48 | 0.61 | 0.83 | 0.83 | 0.95 | 1.00 | 0.99 | 1.00 | 1.00 | 1.00 |
| 25 | 0.21 | 0.35 | 0.60 | 0.76 | 0.89 | 0.94 | 0.98 | 1.00 | 0.99 | 1.00 |
| 26 | 0.21 | 0.38 | 0.52 | 0.72 | 0.86 | 0.96 | 0.98 | 0.99 | 1.00 | 1.00 |
| 27 | 0.33 | 0.46 | 0.37 | 0.53 | 0.50 | 0.55 | 0.56 | 0.66 | 0.74 | 0.81 |
| 28 | 0.59 | 0.78 | 0.93 | 0.95 | 1.00 | 1.00 | 1.00 | 1.00 | 1.00 | 1.00 |
| 29 | 0.58 | 0.82 | 0.93 | 0.98 | 1.00 | 1.00 | 1.00 | 1.00 | 1.00 | 1.00 |
| 30 | 0.49 | 0.57 | 0.62 | 0.65 | 0.64 | 0.71 | 0.75 | 0.83 | 0.90 | 0.94 |
| 31 | 0.05 | 0.05 | 0.24 | 0.34 | 0.63 | 0.76 | 0.89 | 0.98 | 1.00 | 1.00 |
| 32 | 0.04 | 0.08 | 0.22 | 0.29 | 0.55 | 0.80 | 0.94 | 0.98 | 1.00 | 1.00 |
| 33 | 0.06 | 0.22 | 0.20 | 0.36 | 0.54 | 0.60 | 0.75 | 0.89 | 0.93 | 0.99 |
| 34 | 0.94 | 0.98 | 1.00 | 0.99 | 1.00 | 1.00 | 1.00 | 1.00 | 1.00 | 1.00 |
| 35 | 0.95 | 0.98 | 0.99 | 0.99 | 1.00 | 1.00 | 1.00 | 1.00 | 1.00 | 1.00 |
| 36 | 0.83 | 0.90 | 0.99 | 0.98 | 0.98 | 1.00 | 1.00 | 1.00 | 1.00 | 1.00 |
| 37 | 0.03 | 0.06 | 0.19 | 0.44 | 0.64 | 0.86 | 0.95 | 0.97 | 0.98 | 1.00 |
| 38 | 0.04 | 0.12 | 0.30 | 0.49 | 0.66 | 0.81 | 0.96 | 0.98 | 1.00 | 1.00 |
| 39 | 0.05 | 0.12 | 0.26 | 0.45 | 0.64 | 0.81 | 0.85 | 0.99 | 1.00 | 1.00 |
| 40 | 0.16 | 0.24 | 0.45 | 0.80 | 0.81 | 0.90 | 0.98 | 0.98 | 1.00 | 1.00 |
| 41 | 0.20 | 0.29 | 0.37 | 0.77 | 0.91 | 0.97 | 0.96 | 0.99 | 1.00 | 1.00 |
| 42 | 0.11 | 0.33 | 0.38 | 0.73 | 0.83 | 0.89 | 0.96 | 1.00 | 1.00 | 1.00 |

Table 6: POD according to each scenario and each k2 value, k1 = 10
