# Supplementary material for: Evaluation and comparison of statistical methods for early temporal detection of outbreaks: A simulation-based study
Source: PLoS One. 2017 Jul 17;12(7):e0181227. doi: 10.1371/journal.pone.0181227 (PMC5513450; doi:10.1371/journal.pone.0181227)
Supplement: S20 Appendix — (PDF) [file pone.0181227.s020.pdf]

Evaluation and Comparison of Statistical Methods for Early Temporal Detection of Outbreaks: a Simulation-Based Study

Appendix S20: Overall performances of EARS C2 algorithm ($\alpha = 0.001, 0.01$ and 0.05)

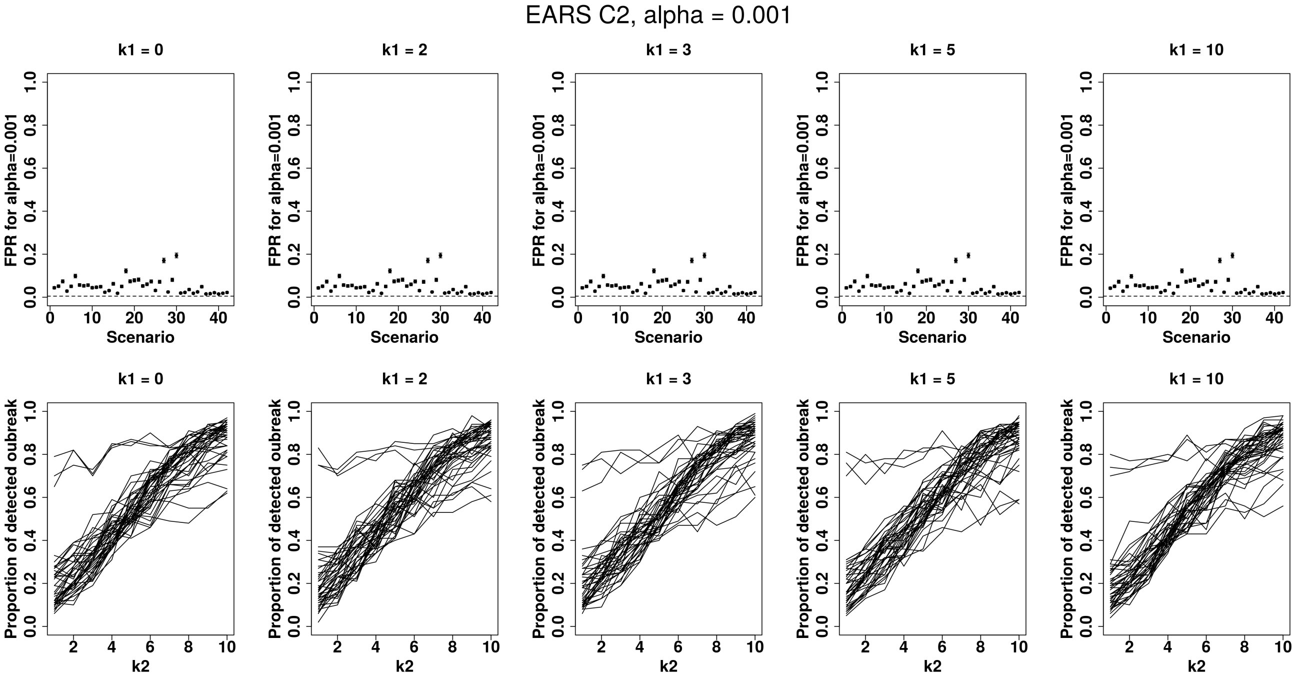

Figure 1: EARS C2 algorithm performances for $\alpha = 0.001$ by increasing past outbreak amplitude $k_1 = 0$, 2, 3, 5 or 10 with (i) on the first row: false positive rate for 42 simulated scenarios, (ii) on the second row: probability of detection for 42 simulated scenarios (each curve corresponding to a scenario) by increasing current outbreak amplitude $k_2 = 1$ to 10.

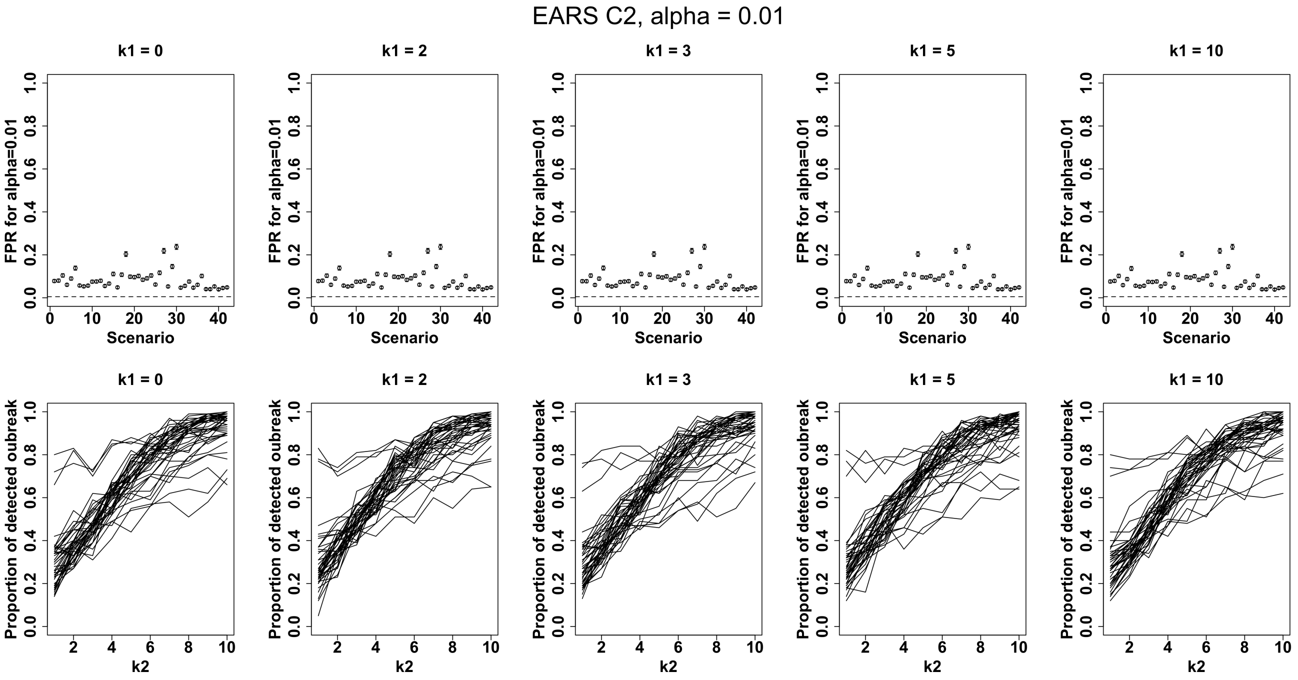

Figure 2: EARS C2 algorithm performances for $\alpha = 0.01$ by increasing past outbreak amplitude $k_1 = 0$, 2, 3, 5 or 10 with (i) on the first row: false positive rate for 42 simulated scenarios, (ii) on the second row: probability of detection for 42 simulated scenarios (each curve corresponding to a scenario) by increasing current outbreak amplitude $k_2 = 1$ to 10.

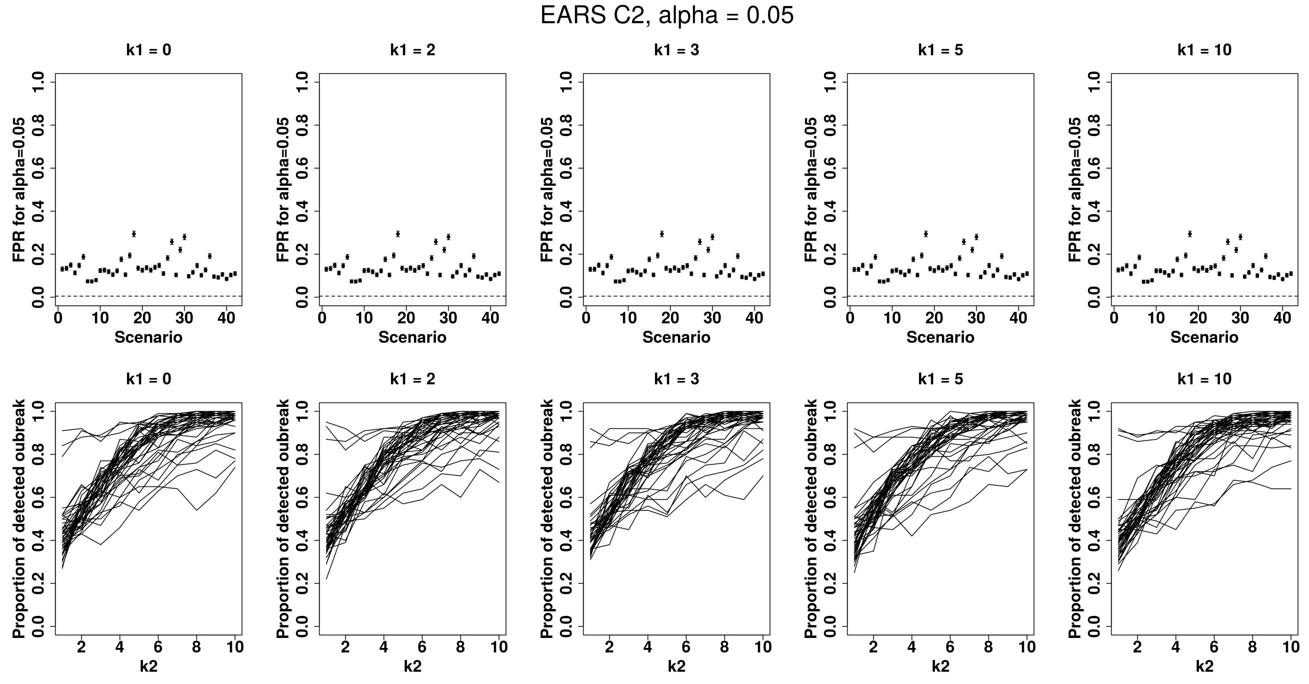

Figure 3: EARS C2 algorithm performances for $\alpha = 0.05$ by increasing past outbreak amplitude $k_1 = 0$, 2, 3, 5 or 10 with (i) on the first row: false positive rate for 42 simulated scenarios, (ii) on the second row: probability of detection for 42 simulated scenarios (each curve corresponding to a scenario) by increasing current outbreak amplitude $k_2 = 1$ to 10.

Overall performances of EARS C2 algorithm ($\alpha = 0.001$)

	FPR k1=0	FPR k1=2	FPR k1=3	FPR k1=5	FPR k1=10
1	0.04	0.04	0.04	0.04	0.04
2	0.05	0.05	0.05	0.05	0.05
3	0.07	0.07	0.07	0.07	0.07
4	0.03	0.03	0.03	0.03	0.03
5	0.05	0.05	0.05	0.05	0.05
6	0.10	0.10	0.10	0.10	0.10
7	0.06	0.06	0.06	0.06	0.06
8	0.05	0.05	0.05	0.05	0.05
9	0.06	0.05	0.06	0.06	0.06
10	0.04	0.04	0.04	0.04	0.04
11	0.05	0.05	0.05	0.05	0.04
12	0.05	0.05	0.05	0.05	0.05
13	0.02	0.02	0.02	0.02	0.02
14	0.03	0.03	0.03	0.03	0.03
15	0.06	0.06	0.06	0.06	0.06
16	0.02	0.02	0.02	0.02	0.02
17	0.05	0.05	0.05	0.05	0.05
18	0.12	0.12	0.12	0.12	0.12
19	0.07	0.07	0.07	0.07	0.07
20	0.08	0.08	0.08	0.08	0.08
21	0.08	0.08	0.08	0.08	0.08
22	0.05	0.05	0.05	0.05	0.05
23	0.06	0.06	0.06	0.06	0.06
24	0.07	0.07	0.07	0.07	0.07
25	0.03	0.03	0.03	0.03	0.03
26	0.07	0.07	0.07	0.07	0.07
27	0.17	0.17	0.17	0.17	0.17
28	0.02	0.02	0.02	0.02	0.02
29	0.08	0.08	0.08	0.08	0.08
30	0.19	0.19	0.19	0.19	0.19
31	0.02	0.02	0.02	0.02	0.02
32	0.02	0.02	0.02	0.02	0.02
33	0.04	0.04	0.04	0.04	0.04
34	0.02	0.02	0.02	0.02	0.02
35	0.02	0.02	0.02	0.02	0.02
36	0.05	0.05	0.05	0.05	0.05
37	0.02	0.02	0.02	0.01	0.02
38	0.02	0.02	0.02	0.02	0.02
39	0.02	0.02	0.02	0.02	0.02
40	0.02	0.02	0.02	0.02	0.01
41	0.02	0.02	0.02	0.02	0.02
42	0.02	0.02	0.02	0.02	0.02

Table 1: FPR according to each scenario and each k1 value, alpha = 0.001

	k2=1	k2=2	k2=3	k2=4	k2=5	k2=6	k2=7	k2=8	k2=9	k2=10
1	0.22	0.34	0.34	0.38	0.59	0.61	0.78	0.87	0.92	0.90
2	0.28	0.33	0.33	0.56	0.61	0.67	0.73	0.80	0.86	0.93
3	0.26	0.22	0.41	0.47	0.49	0.58	0.60	0.63	0.72	0.82
4	0.12	0.19	0.27	0.36	0.48	0.69	0.74	0.85	0.91	0.91
5	0.16	0.27	0.35	0.40	0.49	0.56	0.67	0.71	0.83	0.84
6	0.23	0.30	0.30	0.40	0.49	0.46	0.63	0.64	0.67	0.64
7	0.70	0.75	0.73	0.83	0.86	0.84	0.84	0.89	0.87	0.89
8	0.79	0.82	0.71	0.85	0.84	0.90	0.83	0.84	0.92	0.90
9	0.65	0.82	0.70	0.84	0.87	0.85	0.83	0.89	0.90	0.89
10	0.23	0.24	0.32	0.54	0.67	0.68	0.82	0.84	0.87	0.96
11	0.15	0.26	0.34	0.43	0.63	0.77	0.75	0.83	0.83	0.95
12	0.12	0.20	0.35	0.44	0.53	0.69	0.72	0.77	0.85	0.89
13	0.10	0.20	0.31	0.51	0.49	0.65	0.66	0.80	0.94	0.91
14	0.23	0.19	0.34	0.43	0.50	0.68	0.73	0.79	0.88	0.84
15	0.27	0.38	0.35	0.49	0.50	0.61	0.67	0.75	0.77	0.82
16	0.12	0.27	0.25	0.38	0.46	0.71	0.77	0.87	0.93	0.96
17	0.19	0.17	0.32	0.52	0.50	0.67	0.75	0.70	0.83	0.84
18	0.33	0.30	0.46	0.43	0.55	0.52	0.68	0.68	0.71	0.73
19	0.28	0.33	0.52	0.53	0.70	0.72	0.74	0.85	0.87	0.94
20	0.24	0.34	0.43	0.51	0.58	0.69	0.70	0.84	0.82	0.84
21	0.27	0.39	0.44	0.59	0.55	0.73	0.73	0.72	0.89	0.88
22	0.09	0.28	0.39	0.46	0.56	0.60	0.76	0.82	0.91	0.94
23	0.19	0.28	0.42	0.45	0.62	0.62	0.68	0.75	0.86	0.87
24	0.28	0.23	0.35	0.45	0.53	0.49	0.66	0.65	0.82	0.79
25	0.14	0.18	0.31	0.43	0.50	0.69	0.76	0.85	0.88	0.92
26	0.24	0.23	0.35	0.48	0.51	0.59	0.58	0.67	0.74	0.79
27	0.30	0.39	0.41	0.43	0.41	0.46	0.58	0.55	0.55	0.63
28	0.08	0.21	0.30	0.49	0.48	0.55	0.74	0.85	0.88	0.94
29	0.24	0.30	0.37	0.39	0.50	0.47	0.66	0.66	0.76	0.75
30	0.27	0.33	0.21	0.37	0.52	0.51	0.49	0.48	0.56	0.62
31	0.10	0.17	0.25	0.36	0.56	0.69	0.76	0.91	0.89	0.94
32	0.06	0.15	0.24	0.46	0.42	0.72	0.76	0.89	0.90	0.94
33	0.11	0.21	0.30	0.39	0.49	0.63	0.70	0.82	0.81	0.88
34	0.08	0.15	0.22	0.31	0.62	0.67	0.78	0.79	0.94	0.95
35	0.10	0.20	0.29	0.38	0.54	0.65	0.77	0.87	0.92	0.97
36	0.15	0.18	0.30	0.38	0.56	0.55	0.78	0.81	0.72	0.81
37	0.07	0.20	0.21	0.43	0.48	0.57	0.82	0.85	0.88	0.93
38	0.11	0.10	0.26	0.41	0.52	0.65	0.75	0.84	0.91	0.93
39	0.12	0.21	0.28	0.37	0.46	0.59	0.66	0.75	0.84	0.92
40	0.11	0.16	0.23	0.37	0.50	0.58	0.75	0.89	0.93	0.96
41	0.12	0.12	0.23	0.38	0.57	0.70	0.73	0.81	0.88	0.96
42	0.10	0.14	0.19	0.33	0.42	0.61	0.79	0.81	0.84	0.91

Table 2: POD according to each scenario and each k2 value, k1 = 0, alpha = 0.001

	k2=1	k2=2	k2=3	k2=4	k2=5	k2=6	k2=7	k2=8	k2=9	k2=10
1	0.21	0.31	0.43	0.44	0.63	0.59	0.75	0.85	0.86	0.87
2	0.31	0.34	0.44	0.50	0.60	0.68	0.69	0.79	0.79	0.86
3	0.28	0.29	0.40	0.48	0.58	0.59	0.55	0.73	0.74	0.84
4	0.14	0.23	0.22	0.46	0.55	0.59	0.75	0.82	0.90	0.91
5	0.18	0.28	0.37	0.45	0.44	0.56	0.67	0.74	0.79	0.84
6	0.16	0.36	0.37	0.36	0.40	0.43	0.58	0.59	0.67	0.72
7	0.75	0.73	0.81	0.82	0.84	0.82	0.83	0.83	0.98	0.92
8	0.83	0.70	0.75	0.80	0.82	0.75	0.89	0.92	0.87	0.92
9	0.75	0.71	0.79	0.77	0.86	0.85	0.85	0.88	0.88	0.95
10	0.16	0.28	0.42	0.52	0.62	0.75	0.79	0.83	0.90	0.96
11	0.17	0.24	0.45	0.37	0.60	0.58	0.73	0.83	0.86	0.89
12	0.17	0.32	0.25	0.39	0.56	0.68	0.82	0.80	0.88	0.87
13	0.15	0.17	0.36	0.42	0.67	0.65	0.79	0.80	0.90	0.94
14	0.13	0.24	0.32	0.40	0.51	0.66	0.75	0.88	0.90	0.91
15	0.34	0.31	0.37	0.50	0.61	0.66	0.60	0.81	0.77	0.86
16	0.13	0.18	0.27	0.44	0.51	0.67	0.79	0.78	0.94	0.94
17	0.11	0.14	0.47	0.47	0.57	0.51	0.65	0.76	0.82	0.79
18	0.23	0.36	0.46	0.36	0.51	0.60	0.51	0.65	0.68	0.77
19	0.37	0.37	0.43	0.58	0.68	0.68	0.66	0.77	0.83	0.84
20	0.22	0.34	0.44	0.55	0.65	0.65	0.73	0.68	0.83	0.85
21	0.27	0.32	0.51	0.57	0.53	0.76	0.68	0.84	0.87	0.88
22	0.13	0.25	0.38	0.43	0.65	0.68	0.84	0.88	0.87	0.91
23	0.21	0.26	0.34	0.48	0.59	0.71	0.74	0.78	0.83	0.83
24	0.25	0.26	0.41	0.45	0.52	0.58	0.65	0.70	0.72	0.82
25	0.12	0.19	0.30	0.38	0.68	0.59	0.75	0.84	0.91	0.90
26	0.18	0.25	0.36	0.47	0.56	0.52	0.72	0.65	0.76	0.83
27	0.31	0.29	0.49	0.47	0.49	0.61	0.55	0.62	0.65	0.58
28	0.06	0.20	0.27	0.45	0.62	0.67	0.72	0.84	0.87	0.96
29	0.24	0.29	0.37	0.41	0.46	0.52	0.61	0.61	0.67	0.64
30	0.35	0.34	0.37	0.48	0.47	0.43	0.53	0.53	0.58	0.61
31	0.11	0.16	0.25	0.41	0.48	0.66	0.82	0.85	0.91	0.93
32	0.14	0.13	0.28	0.30	0.47	0.63	0.76	0.86	0.82	0.90
33	0.15	0.20	0.35	0.41	0.54	0.69	0.74	0.80	0.82	0.88
34	0.12	0.15	0.30	0.39	0.46	0.64	0.79	0.89	0.92	0.95
35	0.08	0.23	0.28	0.34	0.55	0.57	0.78	0.85	0.93	0.93
36	0.19	0.17	0.32	0.49	0.43	0.60	0.74	0.79	0.76	0.83
37	0.07	0.16	0.25	0.36	0.58	0.58	0.71	0.78	0.87	0.94
38	0.02	0.15	0.23	0.41	0.41	0.60	0.78	0.85	0.89	0.96
39	0.09	0.23	0.25	0.34	0.41	0.63	0.74	0.83	0.86	0.95
40	0.06	0.23	0.21	0.44	0.52	0.66	0.74	0.90	0.80	0.91
41	0.10	0.11	0.32	0.34	0.42	0.70	0.84	0.83	0.91	0.95
42	0.08	0.10	0.30	0.37	0.57	0.62	0.71	0.80	0.82	0.95

Table 3: POD according to each scenario and each k2 value, k1 = 2, alpha = 0.001

	k2=1	k2=2	k2=3	k2=4	k2=5	k2=6	k2=7	k2=8	k2=9	k2=10
1	0.24	0.29	0.41	0.49	0.72	0.65	0.85	0.84	0.86	0.89
2	0.24	0.29	0.38	0.45	0.51	0.63	0.74	0.76	0.81	0.84
3	0.29	0.40	0.39	0.44	0.59	0.57	0.61	0.62	0.72	0.76
4	0.12	0.23	0.37	0.42	0.52	0.67	0.71	0.76	0.92	0.91
5	0.18	0.22	0.34	0.48	0.46	0.57	0.64	0.78	0.80	0.91
6	0.20	0.29	0.26	0.38	0.41	0.47	0.47	0.56	0.62	0.72
7	0.63	0.67	0.81	0.76	0.76	0.87	0.86	0.90	0.89	0.90
8	0.73	0.81	0.82	0.82	0.76	0.83	0.93	0.89	0.89	0.95
9	0.75	0.78	0.71	0.77	0.83	0.89	0.75	0.85	0.87	0.91
10	0.20	0.26	0.40	0.46	0.51	0.78	0.78	0.86	0.86	0.94
11	0.14	0.27	0.37	0.49	0.59	0.62	0.67	0.83	0.82	0.93
12	0.21	0.24	0.37	0.42	0.53	0.65	0.69	0.79	0.84	0.88
13	0.09	0.24	0.29	0.39	0.58	0.61	0.75	0.81	0.92	0.96
14	0.18	0.16	0.34	0.39	0.56	0.59	0.74	0.84	0.89	0.89
15	0.23	0.22	0.49	0.55	0.49	0.61	0.64	0.77	0.82	0.81
16	0.09	0.13	0.31	0.50	0.50	0.58	0.78	0.86	0.91	0.93
17	0.09	0.33	0.31	0.46	0.50	0.57	0.65	0.73	0.82	0.88
18	0.27	0.30	0.36	0.43	0.45	0.59	0.62	0.70	0.66	0.81
19	0.24	0.39	0.51	0.54	0.66	0.71	0.79	0.81	0.81	0.92
20	0.31	0.34	0.37	0.60	0.63	0.69	0.80	0.81	0.88	0.89
21	0.28	0.39	0.48	0.48	0.63	0.71	0.76	0.82	0.78	0.85
22	0.18	0.30	0.33	0.52	0.52	0.66	0.77	0.79	0.92	0.92
23	0.15	0.29	0.31	0.48	0.50	0.55	0.65	0.87	0.88	0.85
24	0.24	0.25	0.27	0.53	0.61	0.61	0.67	0.73	0.74	0.78
25	0.09	0.20	0.25	0.39	0.54	0.65	0.75	0.83	0.89	0.92
26	0.27	0.27	0.30	0.38	0.46	0.58	0.57	0.66	0.76	0.84
27	0.33	0.31	0.38	0.38	0.40	0.55	0.44	0.59	0.62	0.65
28	0.11	0.21	0.32	0.39	0.56	0.74	0.79	0.91	0.89	0.95
29	0.20	0.26	0.43	0.41	0.46	0.49	0.58	0.57	0.75	0.61
30	0.36	0.38	0.44	0.44	0.42	0.48	0.53	0.47	0.51	0.60
31	0.09	0.24	0.25	0.36	0.50	0.67	0.75	0.81	0.94	0.95
32	0.08	0.19	0.25	0.33	0.47	0.66	0.73	0.82	0.91	0.97
33	0.13	0.22	0.41	0.42	0.44	0.64	0.66	0.74	0.81	0.86
34	0.09	0.13	0.19	0.30	0.47	0.64	0.77	0.84	0.94	0.99
35	0.09	0.19	0.32	0.42	0.51	0.62	0.79	0.85	0.90	0.91
36	0.19	0.21	0.39	0.44	0.46	0.55	0.72	0.77	0.82	0.86
37	0.08	0.09	0.26	0.35	0.47	0.65	0.76	0.79	0.92	0.96
38	0.10	0.13	0.19	0.34	0.53	0.70	0.77	0.81	0.91	0.98
39	0.12	0.15	0.22	0.34	0.43	0.65	0.72	0.83	0.92	0.93
40	0.10	0.22	0.28	0.31	0.49	0.63	0.74	0.89	0.93	0.93
41	0.11	0.18	0.21	0.40	0.44	0.59	0.73	0.89	0.93	0.94
42	0.06	0.23	0.25	0.30	0.54	0.53	0.72	0.77	0.90	0.95

Table 4: POD according to each scenario and each k2 value, k1 = 3, alpha = 0.001

	k2=1	k2=2	k2=3	k2=4	k2=5	k2=6	k2=7	k2=8	k2=9	k2=10
1	0.20	0.32	0.41	0.50	0.58	0.60	0.79	0.91	0.90	0.85
2	0.27	0.26	0.42	0.54	0.64	0.65	0.62	0.75	0.82	0.86
3	0.31	0.36	0.46	0.56	0.55	0.57	0.73	0.70	0.67	0.75
4	0.20	0.23	0.28	0.37	0.51	0.62	0.71	0.87	0.91	0.94
5	0.20	0.26	0.35	0.45	0.46	0.66	0.54	0.79	0.80	0.83
6	0.28	0.31	0.36	0.36	0.35	0.44	0.61	0.47	0.63	0.57
7	0.70	0.80	0.71	0.82	0.81	0.84	0.82	0.90	0.92	0.90
8	0.81	0.76	0.78	0.78	0.83	0.83	0.74	0.90	0.87	0.89
9	0.76	0.66	0.76	0.74	0.78	0.91	0.80	0.91	0.81	0.94
10	0.15	0.23	0.42	0.44	0.68	0.75	0.75	0.78	0.92	0.89
11	0.22	0.20	0.41	0.43	0.59	0.69	0.70	0.84	0.88	0.89
12	0.15	0.30	0.38	0.52	0.56	0.68	0.73	0.85	0.86	0.89
13	0.12	0.23	0.37	0.46	0.52	0.60	0.79	0.88	0.87	0.89
14	0.12	0.22	0.36	0.45	0.64	0.61	0.70	0.83	0.86	0.98
15	0.24	0.30	0.44	0.49	0.58	0.60	0.68	0.80	0.83	0.85
16	0.09	0.15	0.27	0.44	0.54	0.68	0.72	0.91	0.93	0.95
17	0.16	0.25	0.36	0.43	0.56	0.61	0.68	0.63	0.80	0.84
18	0.22	0.31	0.36	0.37	0.51	0.53	0.68	0.72	0.66	0.78
19	0.23	0.38	0.50	0.54	0.63	0.75	0.80	0.84	0.85	0.91
20	0.27	0.37	0.46	0.53	0.67	0.70	0.80	0.74	0.90	0.91
21	0.31	0.36	0.34	0.53	0.63	0.72	0.71	0.77	0.81	0.87
22	0.16	0.29	0.37	0.37	0.61	0.61	0.76	0.79	0.91	0.92
23	0.13	0.23	0.41	0.53	0.58	0.70	0.70	0.76	0.91	0.84
24	0.26	0.31	0.31	0.46	0.53	0.63	0.62	0.60	0.79	0.82
25	0.16	0.19	0.24	0.43	0.47	0.71	0.67	0.89	0.88	0.95
26	0.15	0.24	0.39	0.47	0.59	0.55	0.62	0.72	0.66	0.88
27	0.29	0.34	0.40	0.45	0.49	0.49	0.60	0.66	0.52	0.59
28	0.11	0.25	0.24	0.40	0.48	0.64	0.75	0.81	0.89	0.95
29	0.24	0.25	0.32	0.33	0.49	0.62	0.59	0.68	0.75	0.72
30	0.30	0.34	0.42	0.28	0.42	0.46	0.44	0.50	0.56	0.59
31	0.07	0.17	0.31	0.38	0.48	0.61	0.78	0.79	0.90	0.92
32	0.07	0.16	0.28	0.39	0.50	0.62	0.76	0.72	0.87	0.94
33	0.11	0.24	0.31	0.44	0.58	0.54	0.72	0.75	0.84	0.95
34	0.08	0.16	0.23	0.32	0.62	0.57	0.80	0.81	0.88	0.93
35	0.11	0.23	0.28	0.33	0.53	0.69	0.80	0.84	0.94	0.87
36	0.09	0.23	0.40	0.40	0.55	0.54	0.62	0.78	0.76	0.83
37	0.10	0.15	0.22	0.34	0.51	0.62	0.82	0.87	0.91	0.95
38	0.05	0.13	0.17	0.34	0.51	0.69	0.84	0.80	0.91	0.94
39	0.07	0.14	0.32	0.32	0.36	0.61	0.68	0.81	0.90	0.93
40	0.06	0.15	0.26	0.32	0.45	0.52	0.75	0.83	0.90	0.97
41	0.12	0.20	0.32	0.33	0.47	0.63	0.77	0.88	0.94	0.94
42	0.14	0.18	0.20	0.42	0.49	0.54	0.75	0.78	0.84	0.88

Table 5: POD according to each scenario and each k2 value, k1 = 5, alpha = 0.001

	k2=1	k2=2	k2=3	k2=4	k2=5	k2=6	k2=7	k2=8	k2=9	k2=10
1	0.31	0.30	0.37	0.58	0.66	0.68	0.82	0.78	0.80	0.87
2	0.27	0.26	0.41	0.52	0.55	0.67	0.75	0.81	0.81	0.85
3	0.26	0.38	0.44	0.58	0.50	0.59	0.68	0.69	0.72	0.79
4	0.15	0.20	0.28	0.45	0.51	0.67	0.76	0.89	0.88	0.92
5	0.17	0.24	0.24	0.35	0.49	0.64	0.72	0.72	0.80	0.78
6	0.18	0.24	0.35	0.40	0.44	0.56	0.62	0.50	0.65	0.72
7	0.80	0.77	0.78	0.77	0.87	0.81	0.87	0.87	0.89	0.91
8	0.74	0.73	0.75	0.77	0.89	0.77	0.87	0.86	0.87	0.92
9	0.70	0.72	0.78	0.80	0.77	0.84	0.85	0.81	0.90	0.91
10	0.19	0.24	0.32	0.41	0.61	0.71	0.73	0.83	0.86	0.92
11	0.18	0.18	0.40	0.47	0.57	0.61	0.74	0.80	0.85	0.89
12	0.11	0.21	0.34	0.42	0.57	0.60	0.73	0.78	0.86	0.92
13	0.14	0.16	0.26	0.36	0.62	0.64	0.72	0.83	0.82	0.98
14	0.15	0.18	0.27	0.42	0.66	0.65	0.81	0.77	0.88	0.88
15	0.20	0.20	0.34	0.41	0.57	0.69	0.72	0.75	0.79	0.84
16	0.08	0.13	0.29	0.38	0.49	0.65	0.79	0.92	0.97	0.98
17	0.21	0.29	0.36	0.38	0.55	0.65	0.71	0.71	0.77	0.90
18	0.28	0.29	0.36	0.47	0.48	0.55	0.71	0.66	0.70	0.79
19	0.29	0.49	0.48	0.57	0.64	0.78	0.71	0.80	0.84	0.81
20	0.29	0.28	0.44	0.50	0.65	0.64	0.74	0.79	0.81	0.88
21	0.26	0.31	0.44	0.52	0.65	0.65	0.73	0.70	0.76	0.87
22	0.21	0.25	0.35	0.51	0.60	0.66	0.78	0.86	0.90	0.89
23	0.20	0.27	0.37	0.50	0.55	0.63	0.73	0.80	0.82	0.90
24	0.15	0.21	0.34	0.43	0.53	0.55	0.67	0.64	0.82	0.83
25	0.09	0.26	0.31	0.41	0.51	0.66	0.75	0.84	0.91	0.92
26	0.22	0.26	0.28	0.37	0.46	0.48	0.71	0.74	0.71	0.73
27	0.25	0.34	0.27	0.48	0.43	0.43	0.55	0.53	0.57	0.66
28	0.09	0.20	0.30	0.40	0.55	0.69	0.75	0.79	0.94	0.92
29	0.19	0.21	0.37	0.46	0.54	0.54	0.56	0.76	0.72	0.68
30	0.24	0.36	0.44	0.37	0.48	0.48	0.59	0.56	0.51	0.56
31	0.12	0.10	0.24	0.39	0.56	0.61	0.71	0.82	0.96	0.96
32	0.13	0.18	0.26	0.46	0.44	0.62	0.80	0.87	0.86	0.92
33	0.12	0.23	0.24	0.41	0.46	0.64	0.66	0.76	0.79	0.92
34	0.08	0.20	0.27	0.35	0.53	0.63	0.72	0.83	0.95	0.91
35	0.12	0.23	0.31	0.42	0.56	0.65	0.71	0.86	0.88	0.94
36	0.13	0.21	0.37	0.39	0.46	0.57	0.67	0.78	0.81	0.83
37	0.07	0.19	0.22	0.40	0.57	0.68	0.79	0.84	0.85	0.95
38	0.08	0.14	0.22	0.38	0.43	0.62	0.75	0.78	0.93	0.94
39	0.06	0.14	0.20	0.40	0.53	0.55	0.70	0.84	0.84	0.92
40	0.04	0.13	0.22	0.43	0.55	0.45	0.76	0.75	0.89	0.92
41	0.08	0.18	0.22	0.34	0.59	0.54	0.77	0.86	0.89	0.95
42	0.11	0.14	0.24	0.46	0.44	0.59	0.72	0.80	0.83	0.93

Table 6: POD according to each scenario and each k2 value, k1 = 10, alpha = 0.001

Overall performances of EARS C2 algorithm ($\alpha = 0.01$)

	FPR k1=0	FPR k1=2	FPR k1=3	FPR k1=5	FPR k1=10
1	0.08	0.08	0.08	0.08	0.08
2	0.08	0.08	0.08	0.08	0.08
3	0.10	0.10	0.10	0.10	0.10
4	0.06	0.06	0.06	0.06	0.06
5	0.09	0.09	0.09	0.09	0.09
6	0.14	0.14	0.14	0.14	0.14
7	0.06	0.06	0.06	0.06	0.06
8	0.05	0.05	0.05	0.05	0.05
9	0.06	0.06	0.06	0.06	0.06
10	0.07	0.07	0.07	0.07	0.07
11	0.08	0.08	0.08	0.08	0.07
12	0.08	0.08	0.08	0.08	0.08
13	0.05	0.05	0.05	0.05	0.05
14	0.07	0.07	0.07	0.07	0.07
15	0.11	0.11	0.11	0.11	0.11
16	0.05	0.05	0.05	0.05	0.05
17	0.11	0.11	0.11	0.11	0.11
18	0.20	0.20	0.20	0.20	0.20
19	0.10	0.10	0.10	0.10	0.10
20	0.10	0.10	0.09	0.09	0.09
21	0.10	0.10	0.10	0.10	0.10
22	0.08	0.08	0.08	0.08	0.08
23	0.09	0.09	0.09	0.09	0.09
24	0.10	0.10	0.10	0.10	0.10
25	0.06	0.06	0.06	0.06	0.06
26	0.12	0.12	0.12	0.12	0.12
27	0.22	0.22	0.22	0.22	0.22
28	0.05	0.05	0.05	0.05	0.05
29	0.15	0.15	0.15	0.15	0.15
30	0.24	0.24	0.24	0.24	0.24
31	0.05	0.05	0.05	0.04	0.05
32	0.06	0.06	0.06	0.06	0.06
33	0.08	0.08	0.08	0.08	0.08
34	0.05	0.05	0.05	0.05	0.05
35	0.06	0.06	0.06	0.06	0.06
36	0.10	0.10	0.10	0.10	0.10
37	0.04	0.04	0.04	0.04	0.04
38	0.04	0.04	0.04	0.04	0.04
39	0.05	0.05	0.05	0.05	0.05
40	0.04	0.04	0.04	0.04	0.04
41	0.05	0.05	0.04	0.05	0.05
42	0.05	0.05	0.05	0.05	0.05

Table 7: FPR according to each scenario and each k1 value, alpha = 0.01

	k2=1	k2=2	k2=3	k2=4	k2=5	k2=6	k2=7	k2=8	k2=9	k2=10
1	0.37	0.54	0.47	0.53	0.70	0.77	0.87	0.93	0.97	0.96
2	0.36	0.45	0.46	0.63	0.80	0.80	0.87	0.88	0.90	0.96
3	0.33	0.38	0.54	0.63	0.59	0.70	0.67	0.79	0.81	0.86
4	0.19	0.33	0.42	0.55	0.69	0.83	0.88	0.94	0.97	0.98
5	0.29	0.37	0.46	0.55	0.64	0.70	0.81	0.82	0.92	0.93
6	0.36	0.40	0.36	0.49	0.54	0.56	0.70	0.69	0.74	0.66
7	0.72	0.76	0.73	0.85	0.86	0.84	0.84	0.90	0.88	0.89
8	0.80	0.83	0.72	0.87	0.85	0.90	0.83	0.86	0.92	0.92
9	0.66	0.82	0.70	0.84	0.88	0.85	0.85	0.90	0.92	0.90
10	0.37	0.36	0.46	0.68	0.82	0.77	0.93	0.93	0.96	1.00
11	0.31	0.43	0.52	0.63	0.76	0.87	0.91	0.90	0.91	0.98
12	0.24	0.32	0.48	0.61	0.69	0.80	0.87	0.91	0.96	0.94
13	0.26	0.30	0.46	0.63	0.71	0.83	0.80	0.93	0.99	0.96
14	0.34	0.35	0.47	0.61	0.67	0.78	0.88	0.92	0.97	0.97
15	0.37	0.49	0.48	0.61	0.62	0.70	0.80	0.85	0.88	0.90
16	0.22	0.37	0.49	0.56	0.74	0.85	0.91	0.99	0.99	0.99
17	0.37	0.33	0.48	0.67	0.67	0.76	0.79	0.84	0.87	0.97
18	0.42	0.45	0.55	0.52	0.63	0.63	0.74	0.76	0.80	0.78
19	0.34	0.46	0.62	0.61	0.78	0.82	0.83	0.92	0.99	0.97
20	0.30	0.40	0.55	0.64	0.71	0.83	0.82	0.92	0.92	0.91
21	0.35	0.47	0.53	0.66	0.64	0.81	0.83	0.81	0.96	0.94
22	0.17	0.40	0.57	0.63	0.77	0.76	0.89	0.94	0.96	0.98
23	0.30	0.39	0.59	0.55	0.74	0.80	0.81	0.91	0.92	0.94
24	0.38	0.33	0.44	0.54	0.58	0.61	0.77	0.77	0.87	0.89
25	0.25	0.39	0.43	0.65	0.76	0.86	0.97	0.91	0.96	0.96
26	0.35	0.33	0.43	0.56	0.67	0.71	0.70	0.79	0.86	0.90
27	0.34	0.45	0.44	0.49	0.44	0.56	0.62	0.64	0.62	0.73
28	0.14	0.38	0.49	0.67	0.67	0.79	0.91	0.95	0.95	0.98
29	0.26	0.40	0.48	0.47	0.62	0.59	0.72	0.75	0.80	0.81
30	0.34	0.37	0.31	0.41	0.55	0.57	0.58	0.51	0.58	0.69
31	0.14	0.33	0.47	0.61	0.75	0.89	0.94	0.95	0.97	0.99
32	0.18	0.36	0.46	0.59	0.65	0.88	0.89	0.95	0.97	0.99
33	0.23	0.38	0.47	0.59	0.70	0.80	0.77	0.92	0.93	0.98
34	0.17	0.33	0.39	0.52	0.78	0.87	0.91	0.95	0.99	0.99
35	0.24	0.30	0.48	0.60	0.71	0.83	0.90	0.93	0.98	0.98
36	0.28	0.29	0.48	0.53	0.70	0.73	0.84	0.92	0.85	0.90
37	0.16	0.35	0.33	0.64	0.70	0.77	0.96	0.96	0.98	0.98
38	0.20	0.27	0.45	0.62	0.77	0.82	0.91	0.97	0.97	1.00
39	0.19	0.35	0.47	0.59	0.71	0.79	0.88	0.92	0.98	0.99
40	0.23	0.28	0.42	0.62	0.69	0.80	0.88	0.98	0.99	1.00
41	0.25	0.27	0.39	0.66	0.73	0.87	0.91	0.93	0.99	0.99
42	0.15	0.31	0.39	0.54	0.67	0.75	0.87	0.88	0.90	0.98

Table 8: POD according to each scenario and each k2 value, k1 = 0, alpha = 0.01

	k2=1	k2=2	k2=3	k2=4	k2=5	k2=6	k2=7	k2=8	k2=9	k2=10
1	0.37	0.48	0.53	0.62	0.80	0.74	0.87	0.92	0.93	0.93
2	0.47	0.51	0.51	0.62	0.75	0.78	0.83	0.86	0.91	0.94
3	0.34	0.41	0.48	0.53	0.67	0.69	0.75	0.82	0.80	0.88
4	0.22	0.41	0.39	0.64	0.74	0.76	0.90	0.93	0.94	0.95
5	0.26	0.36	0.50	0.59	0.58	0.68	0.80	0.80	0.87	0.92
6	0.22	0.43	0.45	0.42	0.51	0.51	0.65	0.68	0.75	0.77
7	0.78	0.74	0.81	0.82	0.87	0.83	0.83	0.83	0.98	0.93
8	0.83	0.70	0.75	0.81	0.83	0.77	0.91	0.92	0.90	0.94
9	0.77	0.72	0.79	0.79	0.87	0.85	0.85	0.89	0.89	0.97
10	0.25	0.40	0.56	0.66	0.76	0.85	0.92	0.93	0.97	0.97
11	0.27	0.38	0.57	0.53	0.75	0.79	0.84	0.91	0.93	0.96
12	0.26	0.45	0.40	0.48	0.73	0.88	0.93	0.92	0.93	0.94
13	0.25	0.34	0.48	0.63	0.76	0.81	0.90	0.92	0.97	0.99
14	0.25	0.42	0.49	0.56	0.64	0.79	0.88	0.95	0.96	0.94
15	0.41	0.43	0.48	0.61	0.81	0.74	0.73	0.85	0.82	0.94
16	0.23	0.36	0.47	0.58	0.78	0.86	0.92	0.94	0.99	1.00
17	0.21	0.28	0.63	0.67	0.68	0.70	0.77	0.88	0.91	0.91
18	0.37	0.50	0.57	0.55	0.66	0.69	0.63	0.77	0.75	0.84
19	0.42	0.43	0.56	0.68	0.79	0.80	0.81	0.87	0.90	0.92
20	0.27	0.41	0.52	0.65	0.74	0.72	0.88	0.83	0.93	0.94
21	0.31	0.40	0.57	0.64	0.64	0.79	0.76	0.91	0.93	0.94
22	0.20	0.41	0.49	0.63	0.78	0.79	0.95	0.98	0.95	0.97
23	0.29	0.38	0.54	0.63	0.70	0.83	0.85	0.87	0.93	0.95
24	0.35	0.35	0.51	0.60	0.65	0.67	0.77	0.81	0.84	0.88
25	0.23	0.35	0.47	0.64	0.78	0.75	0.90	0.95	0.99	0.98
26	0.24	0.35	0.45	0.53	0.60	0.69	0.83	0.77	0.85	0.90
27	0.36	0.35	0.53	0.53	0.60	0.68	0.68	0.72	0.70	0.65
28	0.16	0.35	0.38	0.60	0.71	0.86	0.85	0.93	0.98	0.99
29	0.37	0.40	0.44	0.48	0.56	0.63	0.70	0.67	0.76	0.78
30	0.43	0.45	0.43	0.57	0.54	0.48	0.60	0.55	0.64	0.65
31	0.22	0.30	0.46	0.69	0.73	0.82	0.95	0.93	0.98	1.00
32	0.24	0.27	0.52	0.52	0.68	0.77	0.85	0.90	0.93	0.97
33	0.25	0.31	0.54	0.57	0.70	0.77	0.85	0.87	0.95	0.95
34	0.23	0.30	0.49	0.60	0.72	0.86	0.92	0.98	0.98	1.00
35	0.22	0.36	0.44	0.54	0.74	0.74	0.92	0.96	0.97	0.99
36	0.31	0.34	0.43	0.65	0.63	0.72	0.84	0.86	0.85	0.89
37	0.12	0.28	0.48	0.62	0.84	0.79	0.92	0.93	0.97	0.99
38	0.05	0.34	0.45	0.58	0.64	0.84	0.89	0.96	0.99	0.98
39	0.13	0.34	0.43	0.56	0.69	0.82	0.90	0.95	0.95	0.99
40	0.20	0.33	0.40	0.63	0.79	0.82	0.95	0.98	0.96	0.99
41	0.21	0.23	0.51	0.59	0.63	0.88	0.91	0.94	0.96	1.00
42	0.18	0.24	0.45	0.54	0.77	0.82	0.86	0.88	0.96	0.98

Table 9: POD according to each scenario and each k2 value, k1 = 2, alpha = 0.01

	k2=1	k2=2	k2=3	k2=4	k2=5	k2=6	k2=7	k2=8	k2=9	k2=10
1	0.44	0.48	0.59	0.59	0.84	0.79	0.90	0.91	0.90	0.93
2	0.36	0.47	0.53	0.61	0.68	0.75	0.84	0.84	0.88	0.91
3	0.37	0.52	0.51	0.52	0.65	0.73	0.73	0.76	0.86	0.83
4	0.20	0.35	0.51	0.61	0.71	0.81	0.84	0.87	0.97	0.97
5	0.29	0.40	0.41	0.60	0.63	0.69	0.79	0.94	0.89	0.94
6	0.28	0.34	0.35	0.49	0.48	0.54	0.57	0.66	0.73	0.80
7	0.63	0.69	0.81	0.77	0.77	0.88	0.89	0.92	0.89	0.93
8	0.74	0.82	0.84	0.84	0.77	0.83	0.93	0.90	0.92	0.95
9	0.76	0.78	0.73	0.78	0.83	0.90	0.75	0.87	0.88	0.91
10	0.27	0.35	0.57	0.65	0.67	0.90	0.88	0.95	0.93	0.98
11	0.23	0.35	0.47	0.63	0.79	0.79	0.81	0.94	0.92	0.98
12	0.36	0.37	0.51	0.58	0.74	0.81	0.86	0.94	0.92	0.93
13	0.27	0.34	0.52	0.63	0.76	0.73	0.89	0.94	0.95	0.99
14	0.25	0.33	0.48	0.56	0.69	0.78	0.87	0.93	0.96	0.93
15	0.33	0.40	0.59	0.64	0.66	0.74	0.74	0.80	0.93	0.91
16	0.19	0.31	0.60	0.65	0.69	0.82	0.89	0.94	0.98	0.99
17	0.22	0.44	0.47	0.65	0.62	0.70	0.80	0.88	0.91	0.95
18	0.37	0.42	0.51	0.53	0.56	0.72	0.72	0.76	0.71	0.86
19	0.34	0.50	0.61	0.68	0.81	0.78	0.89	0.87	0.88	0.98
20	0.38	0.44	0.57	0.70	0.72	0.76	0.87	0.90	0.93	0.93
21	0.33	0.46	0.59	0.58	0.70	0.79	0.88	0.89	0.88	0.90
22	0.24	0.39	0.46	0.67	0.75	0.83	0.88	0.90	0.97	0.98
23	0.20	0.37	0.51	0.61	0.69	0.70	0.80	0.93	0.94	0.95
24	0.31	0.38	0.43	0.58	0.71	0.74	0.79	0.80	0.81	0.89
25	0.18	0.32	0.41	0.54	0.71	0.79	0.86	0.93	0.98	0.98
26	0.38	0.36	0.45	0.50	0.52	0.68	0.68	0.77	0.83	0.92
27	0.37	0.37	0.44	0.47	0.48	0.60	0.49	0.62	0.69	0.72
28	0.21	0.33	0.50	0.60	0.75	0.91	0.91	0.98	0.96	0.99
29	0.27	0.29	0.51	0.45	0.53	0.59	0.67	0.68	0.78	0.74
30	0.39	0.45	0.47	0.47	0.46	0.54	0.58	0.51	0.55	0.67
31	0.17	0.33	0.45	0.60	0.77	0.93	0.90	0.92	0.98	0.98
32	0.13	0.36	0.45	0.55	0.70	0.86	0.89	0.97	0.97	0.99
33	0.25	0.39	0.56	0.56	0.67	0.74	0.79	0.88	0.90	0.96
34	0.18	0.27	0.37	0.57	0.69	0.89	0.89	0.92	0.98	1.00
35	0.30	0.44	0.54	0.58	0.74	0.84	0.96	0.93	0.97	0.97
36	0.31	0.37	0.57	0.58	0.65	0.74	0.86	0.87	0.91	0.91
37	0.19	0.23	0.44	0.53	0.70	0.85	0.93	0.95	0.99	1.00
38	0.19	0.27	0.45	0.57	0.74	0.86	0.98	0.94	1.00	1.00
39	0.17	0.30	0.40	0.64	0.62	0.82	0.85	0.91	0.98	0.99
40	0.23	0.32	0.47	0.54	0.73	0.85	0.86	0.97	0.97	0.99
41	0.20	0.31	0.40	0.57	0.68	0.78	0.95	0.96	0.99	0.99
42	0.15	0.38	0.39	0.46	0.69	0.77	0.88	0.88	0.98	0.99

Table 10: POD according to each scenario and each k2 value, k1 = 3, alpha = 0.01

	k2=1	k2=2	k2=3	k2=4	k2=5	k2=6	k2=7	k2=8	k2=9	k2=10
1	0.38	0.54	0.57	0.67	0.72	0.79	0.90	0.98	0.96	0.95
2	0.38	0.39	0.58	0.67	0.70	0.77	0.77	0.84	0.88	0.93
3	0.39	0.50	0.54	0.61	0.60	0.63	0.79	0.80	0.76	0.81
4	0.32	0.35	0.41	0.67	0.71	0.80	0.86	0.94	0.96	1.00
5	0.27	0.38	0.45	0.54	0.62	0.75	0.78	0.88	0.91	0.89
6	0.30	0.35	0.43	0.46	0.43	0.51	0.67	0.63	0.71	0.68
7	0.70	0.82	0.71	0.83	0.81	0.84	0.84	0.92	0.93	0.92
8	0.82	0.76	0.79	0.79	0.84	0.84	0.76	0.91	0.88	0.91
9	0.77	0.67	0.78	0.75	0.79	0.91	0.81	0.93	0.82	0.94
10	0.20	0.39	0.57	0.61	0.76	0.88	0.90	0.93	0.94	0.99
11	0.31	0.35	0.55	0.63	0.71	0.83	0.83	0.92	0.94	0.94
12	0.22	0.44	0.56	0.67	0.75	0.87	0.81	0.93	0.95	0.94
13	0.24	0.35	0.50	0.66	0.73	0.77	0.89	0.94	0.95	0.96
14	0.22	0.39	0.56	0.68	0.76	0.73	0.86	0.96	0.93	1.00
15	0.35	0.39	0.57	0.62	0.69	0.70	0.72	0.84	0.89	0.92
16	0.20	0.29	0.50	0.61	0.77	0.87	0.88	0.97	0.97	0.99
17	0.27	0.46	0.61	0.60	0.73	0.76	0.80	0.75	0.88	0.92
18	0.36	0.45	0.44	0.46	0.60	0.66	0.74	0.77	0.76	0.84
19	0.27	0.52	0.59	0.60	0.72	0.89	0.92	0.92	0.91	0.96
20	0.32	0.45	0.56	0.65	0.75	0.79	0.89	0.83	0.96	0.95
21	0.37	0.42	0.43	0.67	0.71	0.77	0.82	0.84	0.89	0.97
22	0.22	0.44	0.54	0.57	0.76	0.80	0.90	0.93	0.96	0.98
23	0.19	0.31	0.52	0.62	0.71	0.80	0.82	0.90	0.97	0.91
24	0.32	0.42	0.43	0.54	0.68	0.71	0.75	0.78	0.92	0.93
25	0.20	0.37	0.40	0.57	0.71	0.83	0.85	0.96	0.92	1.00
26	0.24	0.39	0.49	0.53	0.75	0.65	0.70	0.77	0.79	0.91
27	0.32	0.37	0.45	0.52	0.55	0.53	0.66	0.72	0.64	0.64
28	0.23	0.36	0.48	0.64	0.69	0.83	0.89	0.87	0.98	1.00
29	0.34	0.35	0.38	0.43	0.57	0.67	0.67	0.76	0.84	0.79
30	0.33	0.40	0.49	0.36	0.46	0.51	0.50	0.60	0.59	0.65
31	0.19	0.31	0.45	0.52	0.67	0.78	0.87	0.95	0.97	0.98
32	0.23	0.34	0.49	0.57	0.73	0.74	0.93	0.89	0.99	0.96
33	0.28	0.45	0.49	0.61	0.72	0.76	0.84	0.85	0.89	0.99
34	0.12	0.26	0.48	0.55	0.81	0.79	0.95	0.98	0.96	1.00
35	0.25	0.39	0.50	0.57	0.69	0.85	0.91	0.92	0.96	0.99
36	0.26	0.38	0.56	0.59	0.67	0.76	0.82	0.88	0.86	0.88
37	0.17	0.32	0.41	0.58	0.69	0.86	0.95	0.95	0.94	1.00
38	0.14	0.34	0.39	0.55	0.80	0.84	0.95	0.91	0.98	0.99
39	0.18	0.16	0.46	0.51	0.60	0.83	0.85	0.96	0.95	0.99
40	0.18	0.27	0.45	0.63	0.70	0.78	0.96	0.93	0.97	1.00
41	0.22	0.32	0.52	0.58	0.67	0.79	0.91	0.96	0.98	1.00
42	0.25	0.31	0.37	0.57	0.70	0.83	0.90	0.94	0.93	0.97

Table 11: POD according to each scenario and each k2 value, k1 = 5, alpha = 0.01

	k2=1	k2=2	k2=3	k2=4	k2=5	k2=6	k2=7	k2=8	k2=9	k2=10
1	0.44	0.44	0.51	0.72	0.76	0.82	0.94	0.87	0.88	0.95
2	0.37	0.40	0.58	0.68	0.74	0.81	0.79	0.91	0.92	0.91
3	0.37	0.49	0.52	0.64	0.62	0.72	0.81	0.82	0.79	0.85
4	0.26	0.37	0.42	0.59	0.68	0.81	0.86	0.96	0.95	0.97
5	0.27	0.36	0.34	0.47	0.67	0.78	0.82	0.84	0.93	0.90
6	0.27	0.33	0.46	0.50	0.49	0.68	0.65	0.59	0.77	0.77
7	0.80	0.78	0.78	0.78	0.88	0.81	0.87	0.88	0.90	0.92
8	0.74	0.73	0.75	0.81	0.89	0.80	0.87	0.87	0.91	0.94
9	0.70	0.73	0.79	0.81	0.80	0.86	0.86	0.82	0.92	0.92
10	0.27	0.34	0.52	0.59	0.75	0.82	0.87	0.92	0.92	0.98
11	0.28	0.32	0.55	0.65	0.71	0.74	0.87	0.90	0.93	0.97
12	0.19	0.31	0.47	0.61	0.69	0.75	0.83	0.91	0.93	0.97
13	0.25	0.29	0.45	0.53	0.78	0.82	0.83	0.91	0.94	0.99
14	0.26	0.35	0.45	0.58	0.77	0.82	0.89	0.91	0.95	0.96
15	0.31	0.36	0.53	0.55	0.67	0.78	0.84	0.86	0.85	0.92
16	0.19	0.29	0.48	0.58	0.73	0.83	0.94	0.97	0.98	1.00
17	0.28	0.38	0.53	0.52	0.72	0.75	0.83	0.83	0.84	0.96
18	0.40	0.40	0.46	0.56	0.60	0.65	0.78	0.72	0.77	0.82
19	0.36	0.56	0.63	0.64	0.78	0.92	0.82	0.89	0.93	0.89
20	0.34	0.40	0.55	0.56	0.73	0.77	0.78	0.87	0.92	0.91
21	0.33	0.38	0.52	0.59	0.73	0.79	0.84	0.83	0.85	0.92
22	0.30	0.36	0.42	0.70	0.80	0.80	0.88	0.95	0.95	0.96
23	0.32	0.38	0.53	0.64	0.70	0.73	0.84	0.90	0.91	0.96
24	0.22	0.29	0.40	0.54	0.66	0.65	0.79	0.72	0.86	0.89
25	0.20	0.34	0.46	0.61	0.68	0.76	0.89	0.92	0.94	0.95
26	0.35	0.36	0.41	0.47	0.59	0.59	0.81	0.84	0.78	0.83
27	0.31	0.39	0.32	0.49	0.48	0.52	0.60	0.63	0.68	0.71
28	0.20	0.30	0.47	0.59	0.78	0.84	0.87	0.94	0.99	1.00
29	0.33	0.34	0.44	0.54	0.64	0.60	0.62	0.82	0.79	0.78
30	0.33	0.40	0.46	0.42	0.55	0.51	0.65	0.61	0.60	0.62
31	0.22	0.28	0.45	0.65	0.74	0.84	0.86	0.97	1.00	1.00
32	0.27	0.32	0.44	0.55	0.67	0.81	0.91	0.92	0.93	0.98
33	0.28	0.39	0.48	0.65	0.61	0.75	0.81	0.88	0.87	0.98
34	0.16	0.31	0.43	0.58	0.73	0.80	0.88	0.92	0.99	0.97
35	0.26	0.37	0.50	0.57	0.71	0.78	0.85	0.95	0.93	0.96
36	0.23	0.37	0.44	0.53	0.62	0.73	0.77	0.86	0.88	0.92
37	0.15	0.24	0.45	0.62	0.76	0.81	0.92	0.95	0.95	1.00
38	0.14	0.27	0.36	0.59	0.61	0.81	0.89	0.90	1.00	0.95
39	0.14	0.28	0.38	0.58	0.67	0.75	0.86	0.94	0.93	1.00
40	0.12	0.23	0.39	0.62	0.76	0.77	0.92	0.93	0.97	0.97
41	0.18	0.29	0.42	0.56	0.78	0.78	0.88	0.94	0.95	1.00
42	0.20	0.33	0.38	0.64	0.65	0.75	0.89	0.89	0.95	0.97

Table 12: POD according to each scenario and each k2 value, k1 = 10, alpha = 0.01

Overall performances of EARS C2 algorithm ($\alpha = 0.05$)

	FPR k1=0	FPR k1=2	FPR k1=3	FPR k1=5	FPR k1=10
1	0.13	0.13	0.13	0.13	0.13
2	0.13	0.13	0.13	0.13	0.13
3	0.15	0.15	0.15	0.15	0.15
4	0.11	0.11	0.11	0.11	0.11
5	0.15	0.15	0.15	0.14	0.14
6	0.19	0.19	0.19	0.19	0.19
7	0.07	0.07	0.07	0.07	0.07
8	0.07	0.07	0.07	0.07	0.07
9	0.08	0.08	0.08	0.08	0.08
10	0.12	0.12	0.12	0.12	0.12
11	0.13	0.13	0.13	0.13	0.12
12	0.12	0.12	0.12	0.12	0.11
13	0.10	0.10	0.10	0.10	0.10
14	0.12	0.12	0.12	0.12	0.12
15	0.18	0.18	0.18	0.18	0.18
16	0.10	0.10	0.10	0.10	0.10
17	0.19	0.19	0.19	0.19	0.19
18	0.29	0.29	0.29	0.29	0.29
19	0.14	0.13	0.13	0.13	0.13
20	0.13	0.13	0.12	0.12	0.12
21	0.14	0.14	0.14	0.14	0.14
22	0.13	0.12	0.12	0.12	0.12
23	0.14	0.14	0.14	0.14	0.14
24	0.15	0.15	0.15	0.15	0.15
25	0.11	0.11	0.11	0.11	0.11
26	0.18	0.18	0.18	0.18	0.18
27	0.26	0.26	0.26	0.26	0.26
28	0.10	0.10	0.10	0.10	0.10
29	0.22	0.22	0.22	0.22	0.22
30	0.28	0.28	0.28	0.28	0.28
31	0.10	0.10	0.10	0.09	0.10
32	0.12	0.12	0.12	0.12	0.12
33	0.15	0.15	0.15	0.15	0.15
34	0.10	0.10	0.10	0.10	0.10
35	0.13	0.13	0.13	0.13	0.13
36	0.19	0.19	0.19	0.19	0.19
37	0.10	0.10	0.09	0.09	0.09
38	0.09	0.09	0.09	0.09	0.09
39	0.11	0.11	0.11	0.11	0.11
40	0.09	0.08	0.08	0.08	0.08
41	0.10	0.10	0.10	0.10	0.10
42	0.11	0.11	0.11	0.11	0.11

Table 13: FPR according to each scenario and each k1 value, alpha = 0.05

	k2=1	k2=2	k2=3	k2=4	k2=5	k2=6	k2=7	k2=8	k2=9	k2=10
1	0.52	0.65	0.61	0.73	0.84	0.90	0.95	0.99	0.99	1.00
2	0.55	0.57	0.69	0.79	0.93	0.89	0.93	0.95	0.95	0.99
3	0.45	0.57	0.62	0.70	0.71	0.81	0.75	0.84	0.86	0.90
4	0.34	0.52	0.57	0.77	0.85	0.93	0.98	1.00	0.98	1.00
5	0.46	0.49	0.60	0.66	0.74	0.77	0.89	0.93	0.95	0.98
6	0.43	0.50	0.47	0.60	0.65	0.65	0.75	0.76	0.82	0.78
7	0.84	0.88	0.89	0.94	0.95	0.94	0.95	0.96	0.95	0.97
8	0.91	0.92	0.86	0.95	0.94	0.94	0.92	0.93	0.99	0.96
9	0.79	0.91	0.88	0.90	0.94	0.92	0.93	0.95	0.93	0.96
10	0.50	0.54	0.64	0.76	0.91	0.83	0.99	0.98	0.99	1.00
11	0.43	0.56	0.69	0.77	0.86	0.92	0.98	0.99	0.94	0.99
12	0.34	0.44	0.61	0.76	0.83	0.92	0.96	0.97	0.98	0.98
13	0.43	0.47	0.65	0.79	0.92	0.93	0.94	0.99	1.00	1.00
14	0.48	0.50	0.72	0.74	0.79	0.90	0.91	0.98	0.99	1.00
15	0.52	0.66	0.58	0.69	0.70	0.79	0.89	0.92	0.93	0.96
16	0.37	0.56	0.70	0.76	0.94	0.99	0.99	1.00	1.00	1.00
17	0.50	0.48	0.60	0.82	0.76	0.88	0.91	0.93	0.93	0.98
18	0.52	0.58	0.63	0.60	0.73	0.68	0.80	0.82	0.85	0.82
19	0.45	0.63	0.70	0.73	0.83	0.90	0.89	0.96	1.00	0.98
20	0.44	0.55	0.65	0.72	0.83	0.90	0.91	0.94	0.97	0.97
21	0.51	0.57	0.61	0.73	0.76	0.89	0.89	0.88	0.97	0.96
22	0.34	0.51	0.68	0.78	0.86	0.90	0.98	0.98	0.99	0.99
23	0.40	0.47	0.74	0.70	0.83	0.88	0.92	0.95	0.99	0.98
24	0.52	0.45	0.54	0.72	0.62	0.74	0.87	0.84	0.95	0.93
25	0.41	0.55	0.62	0.87	0.86	0.95	0.98	0.98	1.00	1.00
26	0.38	0.43	0.52	0.67	0.74	0.82	0.81	0.86	0.92	0.96
27	0.40	0.50	0.50	0.58	0.54	0.63	0.70	0.73	0.69	0.77
28	0.27	0.61	0.69	0.82	0.91	0.95	0.98	0.98	0.98	1.00
29	0.36	0.47	0.53	0.56	0.68	0.73	0.79	0.86	0.89	0.90
30	0.41	0.43	0.38	0.46	0.58	0.65	0.64	0.54	0.62	0.74
31	0.30	0.57	0.73	0.79	0.90	0.98	0.99	1.00	0.99	1.00
32	0.33	0.52	0.68	0.82	0.82	0.95	0.95	0.97	0.99	1.00
33	0.39	0.53	0.67	0.73	0.83	0.89	0.89	0.97	0.97	0.99
34	0.36	0.52	0.60	0.81	0.95	0.98	0.97	1.00	1.00	1.00
35	0.42	0.46	0.71	0.76	0.84	0.90	0.99	0.97	0.99	1.00
36	0.44	0.50	0.57	0.68	0.79	0.83	0.88	0.95	0.94	0.97
37	0.34	0.56	0.63	0.82	0.88	0.90	0.99	0.98	1.00	1.00
38	0.41	0.52	0.68	0.84	0.90	0.96	0.98	1.00	1.00	1.00
39	0.28	0.54	0.77	0.77	0.83	0.94	0.93	0.96	0.99	0.99
40	0.39	0.52	0.65	0.83	0.84	0.95	0.99	1.00	1.00	1.00
41	0.39	0.45	0.67	0.81	0.91	0.98	0.97	0.99	1.00	1.00
42	0.31	0.52	0.60	0.74	0.86	0.90	0.97	0.97	0.98	1.00

Table 14: POD according to each scenario and each k2 value, k1 = 0, alpha = 0.05

	k2=1	k2=2	k2=3	k2=4	k2=5	k2=6	k2=7	k2=8	k2=9	k2=10
1	0.54	0.64	0.74	0.71	0.89	0.87	0.92	0.96	0.99	0.97
2	0.62	0.60	0.64	0.78	0.84	0.91	0.90	0.92	0.95	0.99
3	0.52	0.52	0.62	0.66	0.79	0.80	0.84	0.88	0.85	0.94
4	0.39	0.53	0.63	0.79	0.89	0.88	0.96	0.99	0.98	1.00
5	0.46	0.48	0.64	0.70	0.78	0.81	0.90	0.92	0.93	0.98
6	0.29	0.56	0.53	0.55	0.62	0.64	0.71	0.70	0.82	0.81
7	0.95	0.92	0.86	0.91	0.90	0.95	0.91	0.93	0.99	0.94
8	0.93	0.82	0.89	0.92	0.93	0.91	0.94	0.96	0.94	0.98
9	0.87	0.86	0.91	0.92	0.92	0.95	0.94	0.95	0.95	0.97
10	0.37	0.54	0.65	0.82	0.83	0.96	0.95	0.98	0.99	1.00
11	0.43	0.60	0.75	0.66	0.86	0.92	0.94	0.98	0.99	0.99
12	0.35	0.57	0.62	0.69	0.84	0.97	0.98	0.99	0.95	0.98
13	0.44	0.54	0.65	0.81	0.86	0.94	0.97	0.99	0.99	0.99
14	0.39	0.58	0.71	0.76	0.80	0.88	0.95	0.98	1.00	0.98
15	0.50	0.58	0.61	0.75	0.85	0.87	0.82	0.90	0.90	0.97
16	0.46	0.54	0.71	0.81	0.95	0.96	0.98	1.00	1.00	1.00
17	0.47	0.48	0.70	0.77	0.81	0.82	0.92	0.94	0.96	0.99
18	0.47	0.56	0.69	0.61	0.78	0.77	0.72	0.84	0.79	0.88
19	0.50	0.54	0.67	0.81	0.84	0.90	0.91	0.98	0.96	0.99
20	0.39	0.54	0.62	0.78	0.85	0.82	0.93	0.89	0.98	0.99
21	0.41	0.54	0.64	0.74	0.81	0.85	0.88	0.95	0.99	0.97
22	0.34	0.58	0.60	0.80	0.89	0.87	0.96	0.98	0.99	0.99
23	0.36	0.47	0.73	0.75	0.82	0.89	0.92	0.96	0.98	0.98
24	0.43	0.44	0.65	0.71	0.75	0.77	0.86	0.94	0.89	0.93
25	0.35	0.52	0.62	0.83	0.89	0.88	0.97	0.99	1.00	1.00
26	0.32	0.47	0.53	0.60	0.69	0.77	0.91	0.85	0.94	0.98
27	0.43	0.40	0.58	0.60	0.68	0.76	0.74	0.77	0.78	0.73
28	0.34	0.53	0.60	0.79	0.89	0.96	0.96	0.99	1.00	1.00
29	0.46	0.49	0.50	0.58	0.65	0.72	0.80	0.83	0.90	0.86
30	0.51	0.51	0.53	0.62	0.57	0.59	0.66	0.60	0.73	0.67
31	0.36	0.45	0.64	0.88	0.88	0.92	0.99	0.96	1.00	1.00
32	0.39	0.51	0.69	0.77	0.88	0.93	0.95	0.97	0.98	0.98
33	0.37	0.49	0.64	0.81	0.80	0.87	0.92	0.94	0.96	0.98
34	0.40	0.55	0.63	0.84	0.90	0.95	0.99	1.00	1.00	1.00
35	0.36	0.52	0.59	0.75	0.90	0.90	0.99	1.00	0.99	1.00
36	0.45	0.52	0.60	0.76	0.77	0.82	0.90	0.93	0.89	0.95
37	0.37	0.51	0.72	0.85	0.92	0.93	0.99	0.98	1.00	1.00
38	0.22	0.47	0.64	0.80	0.84	0.96	0.97	0.99	1.00	1.00
39	0.29	0.53	0.60	0.75	0.79	0.94	0.98	0.98	1.00	1.00
40	0.37	0.65	0.61	0.79	0.93	0.97	0.99	0.99	1.00	1.00
41	0.44	0.48	0.75	0.78	0.87	0.95	0.97	0.99	0.98	1.00
42	0.36	0.39	0.64	0.70	0.84	0.89	0.96	0.99	0.99	0.99

Table 15: POD according to each scenario and each k2 value, k1 = 2, alpha = 0.05

	k2=1	k2=2	k2=3	k2=4	k2=5	k2=6	k2=7	k2=8	k2=9	k2=10
1	0.57	0.64	0.72	0.75	0.91	0.92	0.93	0.97	0.96	0.97
2	0.51	0.60	0.71	0.72	0.82	0.86	0.93	0.91	0.95	0.97
3	0.52	0.61	0.61	0.63	0.74	0.81	0.85	0.83	0.92	0.92
4	0.35	0.55	0.69	0.76	0.84	0.93	0.94	0.98	0.99	0.99
5	0.40	0.47	0.56	0.75	0.76	0.83	0.89	0.98	0.97	0.98
6	0.35	0.47	0.45	0.61	0.53	0.66	0.70	0.72	0.75	0.82
7	0.86	0.84	0.90	0.90	0.93	0.95	0.96	0.95	0.97	0.98
8	0.83	0.92	0.92	0.92	0.89	0.93	0.96	0.94	0.97	0.97
9	0.92	0.87	0.85	0.91	0.92	0.97	0.90	0.95	0.96	0.95
10	0.42	0.51	0.68	0.79	0.84	0.96	0.96	0.99	0.98	1.00
11	0.39	0.51	0.65	0.81	0.90	0.93	0.91	0.97	0.99	0.98
12	0.48	0.48	0.62	0.73	0.88	0.92	0.93	0.98	0.96	0.97
13	0.48	0.51	0.65	0.78	0.88	0.88	0.96	0.99	0.99	1.00
14	0.49	0.58	0.67	0.75	0.82	0.93	0.93	0.97	0.98	0.97
15	0.45	0.53	0.72	0.71	0.75	0.80	0.87	0.87	0.99	0.91
16	0.33	0.55	0.76	0.90	0.82	0.95	0.96	0.97	0.99	0.99
17	0.34	0.62	0.61	0.77	0.80	0.80	0.89	0.92	0.96	0.99
18	0.45	0.51	0.64	0.64	0.63	0.79	0.76	0.84	0.77	0.87
19	0.41	0.67	0.74	0.78	0.92	0.90	0.96	0.97	0.96	0.99
20	0.45	0.52	0.64	0.78	0.85	0.83	0.92	0.97	0.99	0.97
21	0.47	0.55	0.74	0.69	0.80	0.86	0.98	0.92	0.95	0.95
22	0.34	0.52	0.62	0.83	0.92	0.95	0.94	1.00	0.99	1.00
23	0.34	0.49	0.71	0.77	0.79	0.82	0.94	0.99	0.99	0.99
24	0.39	0.56	0.55	0.70	0.85	0.86	0.88	0.85	0.92	0.98
25	0.31	0.48	0.70	0.78	0.83	0.93	0.97	1.00	0.98	1.00
26	0.46	0.46	0.53	0.60	0.65	0.77	0.80	0.85	0.93	0.97
27	0.41	0.45	0.52	0.56	0.52	0.70	0.60	0.68	0.73	0.78
28	0.36	0.52	0.68	0.82	0.90	0.97	0.98	0.99	0.99	1.00
29	0.36	0.38	0.61	0.59	0.59	0.69	0.75	0.76	0.91	0.85
30	0.43	0.53	0.54	0.54	0.51	0.59	0.66	0.61	0.59	0.70
31	0.35	0.52	0.75	0.80	0.90	0.99	0.96	0.98	0.99	1.00
32	0.32	0.61	0.61	0.83	0.86	0.94	0.96	0.99	1.00	0.99
33	0.40	0.49	0.71	0.70	0.74	0.83	0.89	0.96	0.96	0.99
34	0.34	0.53	0.63	0.76	0.89	0.99	0.97	1.00	0.99	1.00
35	0.42	0.62	0.72	0.77	0.88	0.93	0.98	0.98	0.99	0.99
36	0.44	0.56	0.72	0.75	0.79	0.82	0.89	0.92	0.94	0.95
37	0.35	0.51	0.74	0.76	0.87	0.95	0.97	0.98	1.00	1.00
38	0.41	0.50	0.61	0.84	0.84	0.94	0.99	0.98	1.00	1.00
39	0.35	0.47	0.65	0.81	0.84	0.92	0.95	0.97	1.00	0.99
40	0.34	0.55	0.65	0.85	0.86	0.96	0.98	1.00	1.00	1.00
41	0.39	0.49	0.70	0.79	0.87	0.92	0.98	0.99	1.00	1.00
42	0.35	0.53	0.57	0.70	0.89	0.95	0.95	0.95	1.00	1.00

Table 16: POD according to each scenario and each k2 value, k1 = 3, alpha = 0.05

	k2=1	k2=2	k2=3	k2=4	k2=5	k2=6	k2=7	k2=8	k2=9	k2=10
1	0.55	0.67	0.74	0.84	0.86	0.92	0.96	0.99	0.98	0.98
2	0.48	0.54	0.74	0.82	0.82	0.89	0.91	0.92	0.94	0.96
3	0.55	0.60	0.67	0.72	0.74	0.73	0.84	0.86	0.88	0.90
4	0.43	0.50	0.67	0.74	0.84	0.90	0.95	0.98	1.00	1.00
5	0.46	0.49	0.61	0.61	0.70	0.85	0.84	0.90	0.96	0.97
6	0.32	0.41	0.52	0.58	0.54	0.60	0.74	0.72	0.80	0.83
7	0.83	0.88	0.88	0.92	0.92	0.92	0.91	0.97	0.97	0.95
8	0.92	0.88	0.91	0.93	0.92	0.93	0.92	0.98	0.96	0.95
9	0.90	0.81	0.88	0.88	0.87	0.98	0.91	0.96	0.91	0.97
10	0.33	0.56	0.72	0.77	0.89	0.95	0.98	0.96	0.99	1.00
11	0.44	0.47	0.69	0.75	0.90	0.88	0.95	0.96	0.98	0.98
12	0.31	0.59	0.68	0.78	0.87	0.93	0.94	0.98	0.98	0.98
13	0.46	0.52	0.70	0.80	0.88	0.91	0.97	0.99	0.98	1.00
14	0.39	0.45	0.70	0.84	0.83	0.87	0.93	0.98	0.97	1.00
15	0.51	0.48	0.70	0.73	0.79	0.79	0.84	0.92	0.95	0.97
16	0.39	0.57	0.67	0.82	0.93	0.97	0.95	0.99	0.99	1.00
17	0.47	0.59	0.73	0.73	0.79	0.90	0.88	0.87	0.96	0.96
18	0.50	0.58	0.52	0.60	0.67	0.73	0.79	0.80	0.82	0.86
19	0.39	0.61	0.67	0.75	0.83	0.95	0.96	0.99	0.96	0.97
20	0.46	0.56	0.62	0.73	0.87	0.88	0.95	0.92	0.98	0.99
21	0.50	0.54	0.51	0.72	0.80	0.86	0.94	0.93	0.93	0.99
22	0.34	0.54	0.72	0.71	0.86	0.93	0.96	0.99	0.99	1.00
23	0.30	0.48	0.62	0.74	0.82	0.87	0.92	0.96	0.99	0.99
24	0.43	0.59	0.53	0.65	0.82	0.81	0.86	0.85	0.95	0.99
25	0.36	0.52	0.61	0.79	0.83	0.94	0.95	0.99	0.99	1.00
26	0.32	0.47	0.60	0.65	0.84	0.77	0.77	0.88	0.91	0.98
27	0.39	0.43	0.51	0.57	0.62	0.64	0.72	0.79	0.71	0.73
28	0.37	0.54	0.67	0.75	0.82	0.95	0.97	0.99	1.00	1.00
29	0.48	0.46	0.45	0.52	0.66	0.70	0.84	0.86	0.92	0.85
30	0.37	0.51	0.52	0.42	0.52	0.54	0.58	0.66	0.65	0.73
31	0.37	0.53	0.73	0.73	0.82	0.93	0.94	0.99	1.00	1.00
32	0.42	0.58	0.66	0.72	0.92	0.87	0.97	0.96	1.00	1.00
33	0.51	0.61	0.71	0.73	0.82	0.88	0.91	0.96	0.94	0.99
34	0.25	0.56	0.69	0.81	0.96	0.91	0.99	1.00	0.99	1.00
35	0.35	0.54	0.72	0.76	0.86	0.95	0.97	0.97	1.00	1.00
36	0.42	0.49	0.70	0.70	0.80	0.87	0.87	0.92	0.94	0.95
37	0.31	0.46	0.63	0.74	0.89	0.95	0.97	0.98	0.97	1.00
38	0.28	0.54	0.64	0.79	0.92	1.00	0.99	0.98	1.00	1.00
39	0.33	0.35	0.68	0.74	0.79	0.96	0.95	0.98	0.99	1.00
40	0.34	0.53	0.71	0.84	0.87	0.92	0.99	1.00	1.00	1.00
41	0.41	0.49	0.74	0.79	0.86	0.95	0.96	1.00	1.00	1.00
42	0.38	0.58	0.65	0.81	0.91	0.93	0.97	0.99	0.99	1.00

Table 17: POD according to each scenario and each k2 value, k1 = 5, alpha = 0.05

	k2=1	k2=2	k2=3	k2=4	k2=5	k2=6	k2=7	k2=8	k2=9	k2=10
1	0.59	0.59	0.67	0.83	0.87	0.93	0.97	0.94	0.94	0.96
2	0.49	0.55	0.74	0.78	0.89	0.91	0.90	0.96	0.94	0.96
3	0.50	0.59	0.64	0.72	0.77	0.85	0.89	0.87	0.89	0.91
4	0.45	0.53	0.60	0.76	0.82	0.92	0.97	0.98	0.99	0.99
5	0.36	0.50	0.46	0.62	0.83	0.90	0.89	0.90	0.98	0.95
6	0.33	0.43	0.56	0.60	0.59	0.73	0.75	0.72	0.88	0.83
7	0.92	0.88	0.91	0.90	0.95	0.96	0.94	0.97	0.95	0.98
8	0.89	0.86	0.87	0.93	0.93	0.91	0.93	0.94	0.93	0.98
9	0.91	0.89	0.90	0.91	0.89	0.93	0.95	0.91	0.95	0.96
10	0.39	0.55	0.70	0.74	0.85	0.92	0.94	0.97	0.95	1.00
11	0.39	0.47	0.66	0.78	0.84	0.85	0.94	0.95	0.97	0.98
12	0.34	0.48	0.63	0.68	0.83	0.90	0.93	0.97	0.99	0.98
13	0.40	0.54	0.61	0.77	0.89	0.95	0.91	0.98	0.98	1.00
14	0.41	0.52	0.59	0.73	0.87	0.90	0.96	0.97	0.98	0.98
15	0.42	0.49	0.65	0.67	0.78	0.86	0.91	0.91	0.90	0.96
16	0.32	0.57	0.64	0.86	0.83	0.96	0.97	0.99	0.98	1.00
17	0.44	0.55	0.67	0.68	0.81	0.89	0.93	0.93	0.94	0.98
18	0.47	0.50	0.52	0.69	0.70	0.74	0.84	0.84	0.83	0.84
19	0.47	0.69	0.75	0.73	0.89	0.96	0.92	0.96	0.95	0.95
20	0.44	0.48	0.66	0.75	0.82	0.88	0.85	0.94	0.97	0.97
21	0.44	0.59	0.59	0.72	0.80	0.89	0.92	0.93	0.93	0.99
22	0.41	0.48	0.63	0.79	0.91	0.89	0.97	0.96	0.98	1.00
23	0.39	0.52	0.68	0.78	0.77	0.87	0.90	0.96	0.96	0.98
24	0.31	0.45	0.52	0.66	0.78	0.78	0.86	0.78	0.94	0.94
25	0.35	0.50	0.69	0.79	0.85	0.94	0.98	0.98	1.00	0.98
26	0.45	0.45	0.52	0.60	0.72	0.70	0.94	0.91	0.87	0.92
27	0.33	0.45	0.43	0.54	0.55	0.57	0.66	0.68	0.74	0.77
28	0.30	0.46	0.68	0.77	0.89	0.94	0.98	0.99	0.99	1.00
29	0.47	0.46	0.55	0.69	0.72	0.72	0.76	0.89	0.90	0.89
30	0.37	0.51	0.49	0.45	0.58	0.56	0.68	0.67	0.64	0.64
31	0.40	0.47	0.70	0.78	0.93	0.98	0.97	0.99	1.00	1.00
32	0.40	0.50	0.64	0.76	0.79	0.91	0.97	0.95	0.95	0.99
33	0.44	0.59	0.64	0.80	0.77	0.87	0.90	0.95	0.92	1.00
34	0.36	0.48	0.66	0.81	0.90	0.94	0.99	1.00	1.00	0.99
35	0.44	0.56	0.74	0.77	0.82	0.91	0.94	0.99	0.99	0.99
36	0.43	0.51	0.56	0.63	0.75	0.81	0.89	0.95	0.93	0.97
37	0.34	0.49	0.69	0.82	0.95	0.94	0.96	0.99	0.99	1.00
38	0.26	0.44	0.63	0.73	0.85	0.91	0.99	0.98	1.00	0.97
39	0.30	0.51	0.54	0.72	0.83	0.88	0.96	0.99	1.00	1.00
40	0.29	0.39	0.62	0.85	0.92	0.93	0.98	0.98	0.99	0.99
41	0.38	0.58	0.65	0.74	0.93	0.94	0.94	0.99	1.00	1.00
42	0.39	0.50	0.55	0.78	0.86	0.93	0.94	0.96	0.97	0.99

Table 18: POD according to each scenario and each k2 value, k1 = 10, alpha = 0.05
