# Supplementary material for: Evaluation and comparison of statistical methods for early temporal detection of outbreaks: A simulation-based study
Source: PLoS One. 2017 Jul 17;12(7):e0181227. doi: 10.1371/journal.pone.0181227 (PMC5513450; doi:10.1371/journal.pone.0181227)
Supplement: S21 Appendix — (PDF) [file pone.0181227.s021.pdf]

Evaluation and Comparison of Statistical Methods for Early Temporal Detection of Outbreaks: a Simulation-Based Study

Appendix S21: Overall performances of EARS C3 algorithm ($\alpha = 0.001, 0.01$ and 0.05)

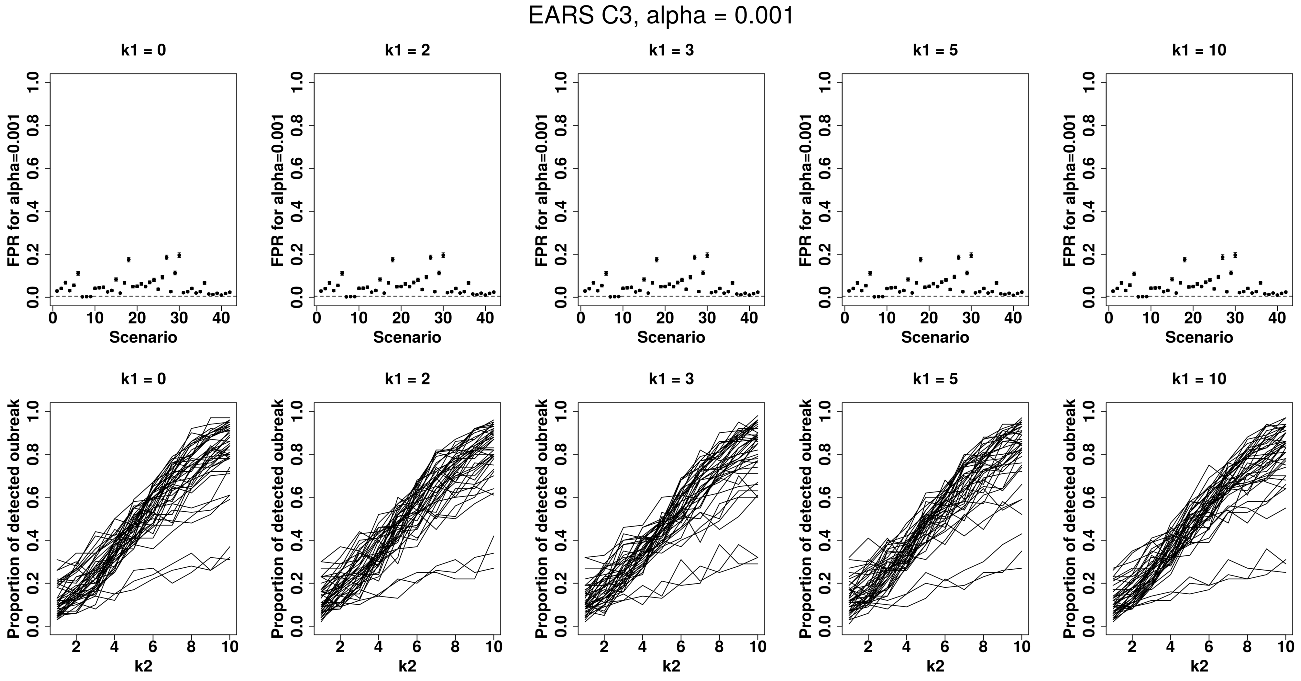

Figure 1: EARS C3 algorithm performances for $\alpha = 0.001$ by increasing past outbreak amplitude $k_1 = 0$, 2, 3, 5 or 10 with (i) on the first row: false positive rate for 42 simulated scenarios, (ii) on the second row: probability of detection for 42 simulated scenarios (each curve corresponding to a scenario) by increasing current outbreak amplitude $k_2 = 1$ to 10.

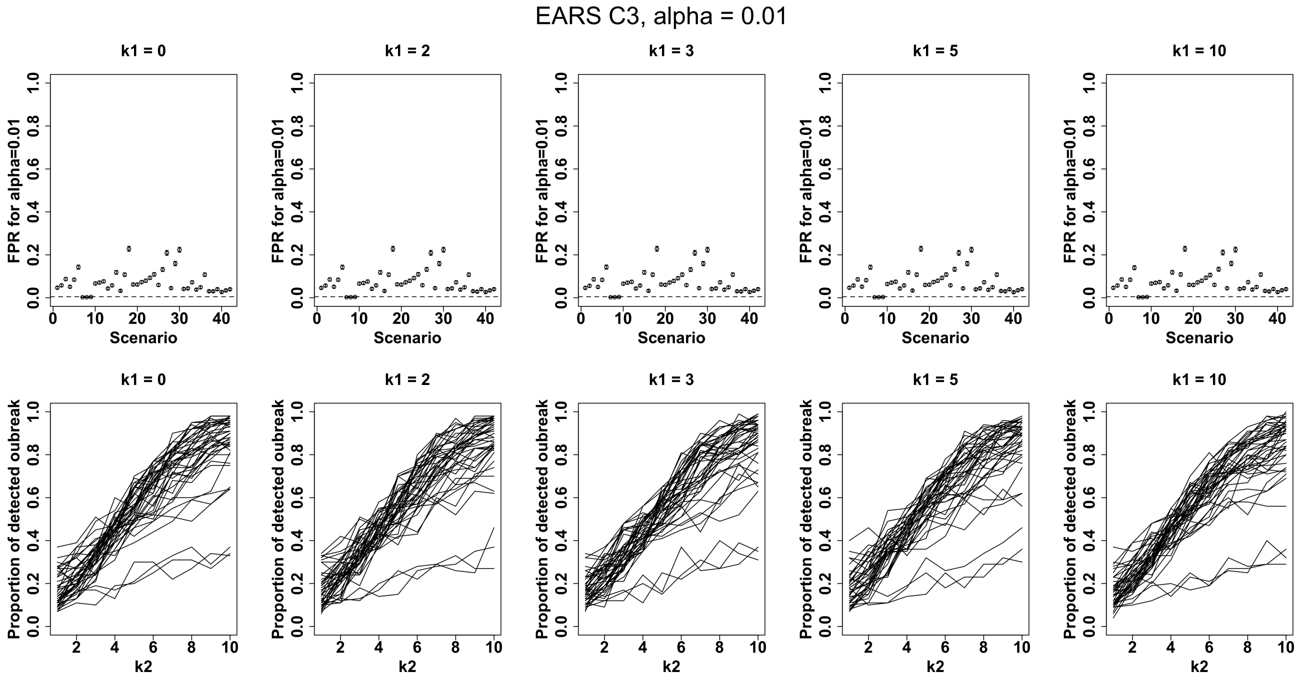

Figure 2: EARS C3 algorithm performances for $\alpha = 0.01$ by increasing past outbreak amplitude $k_1 = 0$, 2, 3, 5 or 10 with (i) on the first row: false positive rate for 42 simulated scenarios, (ii) on the second row: probability of detection for 42 simulated scenarios (each curve corresponding to a scenario) by increasing current outbreak amplitude $k_2 = 1$ to 10.

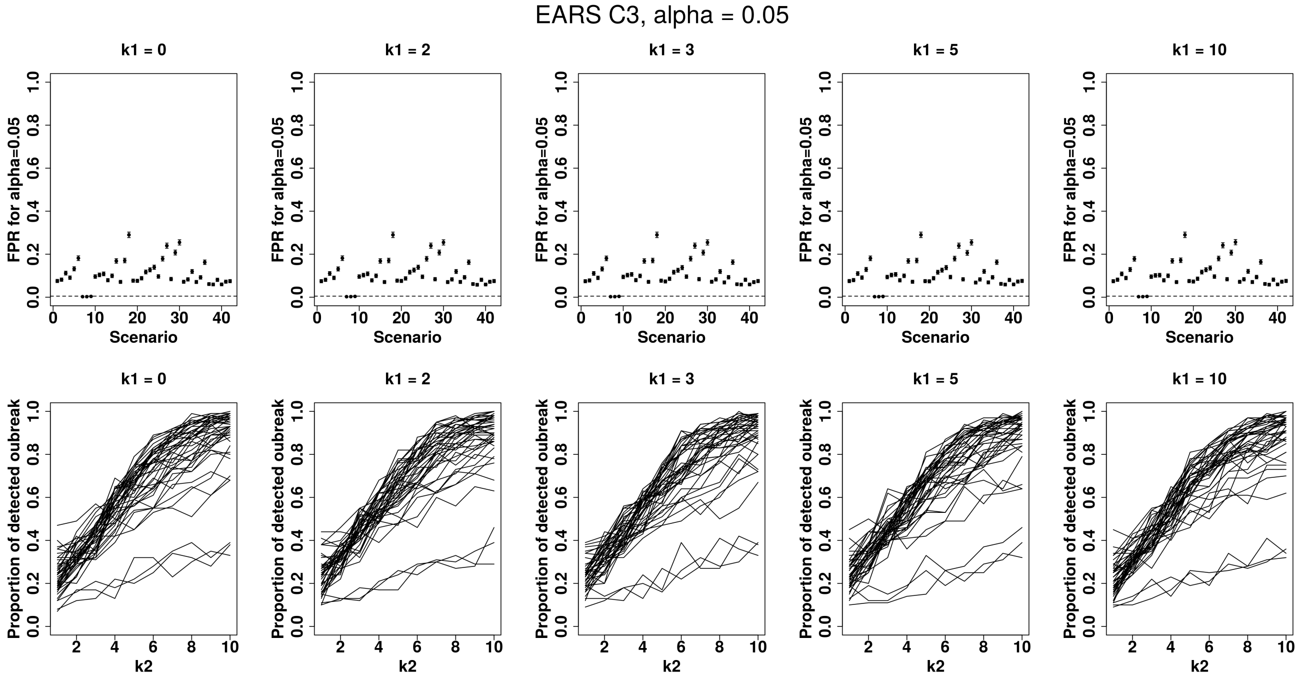

Figure 3: EARS C3 algorithm performances for $\alpha = 0.05$ by increasing past outbreak amplitude $k_1 = 0$, 2, 3, 5 or 10 with (i) on the first row: false positive rate for 42 simulated scenarios, (ii) on the second row: probability of detection for 42 simulated scenarios (each curve corresponding to a scenario) by increasing current outbreak amplitude $k_2 = 1$ to 10.

Overall performances of EARS C3 algorithm ($\alpha = 0.001$)

	FPR k1=0	FPR k1=2	FPR k1=3	FPR k1=5	FPR k1=10
1	0.03	0.03	0.03	0.03	0.03
2	0.04	0.04	0.04	0.04	0.04
3	0.07	0.07	0.07	0.07	0.07
4	0.03	0.03	0.03	0.03	0.03
5	0.06	0.06	0.05	0.05	0.06
6	0.11	0.11	0.11	0.11	0.11
7	0.00	0.00	0.00	0.00	0.00
8	0.00	0.00	0.00	0.00	0.00
9	0.00	0.00	0.00	0.00	0.00
10	0.04	0.04	0.04	0.04	0.04
11	0.04	0.04	0.04	0.04	0.04
12	0.05	0.05	0.05	0.05	0.04
13	0.02	0.02	0.02	0.02	0.03
14	0.03	0.03	0.03	0.03	0.03
15	0.08	0.08	0.08	0.08	0.08
16	0.02	0.02	0.02	0.02	0.02
17	0.07	0.07	0.07	0.07	0.07
18	0.18	0.18	0.18	0.18	0.18
19	0.05	0.05	0.05	0.05	0.05
20	0.05	0.05	0.05	0.05	0.05
21	0.06	0.06	0.06	0.06	0.06
22	0.05	0.05	0.05	0.05	0.05
23	0.07	0.07	0.07	0.07	0.07
24	0.08	0.08	0.08	0.08	0.08
25	0.04	0.04	0.04	0.04	0.04
26	0.09	0.09	0.09	0.09	0.09
27	0.18	0.18	0.18	0.19	0.19
28	0.03	0.03	0.03	0.03	0.03
29	0.11	0.11	0.11	0.11	0.11
30	0.20	0.20	0.20	0.20	0.20
31	0.02	0.02	0.02	0.02	0.02
32	0.03	0.03	0.03	0.03	0.03
33	0.04	0.04	0.04	0.04	0.04
34	0.02	0.02	0.02	0.02	0.02
35	0.03	0.03	0.03	0.03	0.03
36	0.07	0.07	0.07	0.07	0.07
37	0.02	0.01	0.02	0.02	0.02
38	0.01	0.01	0.01	0.01	0.01
39	0.02	0.02	0.02	0.02	0.02
40	0.01	0.01	0.01	0.01	0.01
41	0.02	0.02	0.02	0.02	0.02
42	0.02	0.02	0.02	0.02	0.02

Table 1: FPR according to each scenario and each k1 value, alpha = 0.001

	k2=1	k2=2	k2=3	k2=4	k2=5	k2=6	k2=7	k2=8	k2=9	k2=10
1	0.10	0.15	0.18	0.25	0.42	0.48	0.69	0.75	0.76	0.81
2	0.12	0.16	0.22	0.30	0.38	0.47	0.47	0.64	0.74	0.84
3	0.20	0.16	0.30	0.31	0.38	0.36	0.44	0.51	0.58	0.74
4	0.05	0.12	0.18	0.32	0.48	0.55	0.71	0.82	0.87	0.86
5	0.09	0.14	0.22	0.25	0.40	0.48	0.63	0.68	0.79	0.80
6	0.11	0.23	0.25	0.35	0.41	0.41	0.57	0.55	0.58	0.61
7	0.05	0.15	0.14	0.12	0.26	0.27	0.20	0.25	0.32	0.31
8	0.07	0.11	0.08	0.15	0.17	0.22	0.26	0.28	0.26	0.32
9	0.10	0.13	0.17	0.15	0.21	0.24	0.29	0.34	0.26	0.37
10	0.13	0.19	0.20	0.34	0.55	0.62	0.71	0.75	0.81	0.91
11	0.12	0.12	0.25	0.39	0.47	0.62	0.67	0.69	0.79	0.88
12	0.09	0.18	0.25	0.28	0.36	0.60	0.58	0.74	0.82	0.82
13	0.05	0.06	0.10	0.32	0.39	0.52	0.52	0.68	0.78	0.85
14	0.10	0.14	0.26	0.30	0.40	0.55	0.59	0.73	0.82	0.79
15	0.21	0.27	0.20	0.36	0.36	0.55	0.57	0.64	0.74	0.78
16	0.04	0.17	0.16	0.28	0.43	0.66	0.79	0.84	0.90	0.95
17	0.18	0.15	0.33	0.50	0.47	0.65	0.70	0.66	0.82	0.86
18	0.31	0.28	0.44	0.42	0.55	0.53	0.66	0.64	0.72	0.72
19	0.18	0.27	0.38	0.50	0.58	0.59	0.64	0.80	0.89	0.91
20	0.20	0.26	0.36	0.43	0.55	0.60	0.61	0.69	0.75	0.84
21	0.19	0.25	0.34	0.40	0.49	0.63	0.72	0.72	0.84	0.78
22	0.10	0.23	0.26	0.38	0.52	0.53	0.71	0.85	0.90	0.94
23	0.13	0.29	0.42	0.40	0.55	0.60	0.66	0.76	0.83	0.80
24	0.22	0.21	0.31	0.39	0.48	0.43	0.64	0.65	0.78	0.78
25	0.09	0.13	0.28	0.32	0.52	0.62	0.75	0.80	0.88	0.92
26	0.21	0.20	0.32	0.45	0.41	0.53	0.48	0.68	0.72	0.79
27	0.26	0.34	0.34	0.43	0.39	0.46	0.54	0.53	0.54	0.59
28	0.04	0.14	0.22	0.38	0.44	0.58	0.70	0.85	0.88	0.94
29	0.21	0.26	0.34	0.41	0.48	0.47	0.65	0.62	0.70	0.71
30	0.26	0.30	0.22	0.33	0.47	0.50	0.49	0.48	0.52	0.61
31	0.07	0.07	0.19	0.32	0.48	0.65	0.77	0.90	0.88	0.96
32	0.06	0.06	0.18	0.34	0.41	0.67	0.73	0.86	0.90	0.93
33	0.08	0.17	0.27	0.38	0.41	0.63	0.67	0.78	0.77	0.84
34	0.03	0.09	0.16	0.26	0.51	0.60	0.68	0.79	0.97	0.97
35	0.08	0.16	0.24	0.34	0.50	0.62	0.77	0.86	0.92	0.91
36	0.12	0.18	0.28	0.39	0.56	0.56	0.76	0.74	0.74	0.79
37	0.04	0.12	0.15	0.40	0.41	0.51	0.76	0.80	0.88	0.92
38	0.07	0.13	0.19	0.40	0.47	0.63	0.74	0.79	0.88	0.93
39	0.03	0.13	0.22	0.34	0.45	0.59	0.67	0.73	0.83	0.90
40	0.06	0.06	0.19	0.37	0.46	0.53	0.72	0.92	0.94	0.95
41	0.08	0.10	0.14	0.34	0.59	0.67	0.78	0.79	0.91	0.96
42	0.06	0.12	0.17	0.27	0.47	0.56	0.76	0.77	0.79	0.89

Table 2: POD according to each scenario and each k2 value, k1 = 0, alpha = 0.001

	k2=1	k2=2	k2=3	k2=4	k2=5	k2=6	k2=7	k2=8	k2=9	k2=10
1	0.08	0.19	0.27	0.29	0.42	0.40	0.57	0.69	0.73	0.80
2	0.13	0.25	0.23	0.33	0.41	0.52	0.59	0.67	0.66	0.78
3	0.20	0.23	0.19	0.40	0.29	0.53	0.45	0.56	0.60	0.72
4	0.06	0.14	0.12	0.33	0.40	0.52	0.64	0.75	0.83	0.89
5	0.09	0.23	0.25	0.33	0.36	0.46	0.64	0.70	0.74	0.80
6	0.17	0.29	0.34	0.30	0.36	0.37	0.52	0.51	0.66	0.70
7	0.09	0.09	0.17	0.14	0.13	0.21	0.26	0.25	0.32	0.34
8	0.15	0.09	0.12	0.14	0.22	0.20	0.28	0.31	0.25	0.27
9	0.09	0.11	0.10	0.15	0.21	0.25	0.25	0.22	0.22	0.42
10	0.16	0.14	0.26	0.34	0.52	0.60	0.72	0.67	0.84	0.90
11	0.07	0.16	0.35	0.28	0.44	0.45	0.61	0.74	0.80	0.87
12	0.09	0.20	0.13	0.29	0.49	0.54	0.74	0.67	0.80	0.80
13	0.04	0.11	0.19	0.28	0.47	0.46	0.60	0.72	0.81	0.88
14	0.09	0.16	0.22	0.29	0.36	0.52	0.66	0.77	0.83	0.83
15	0.23	0.23	0.25	0.41	0.49	0.54	0.53	0.72	0.68	0.80
16	0.04	0.14	0.21	0.36	0.48	0.64	0.79	0.81	0.92	0.94
17	0.14	0.18	0.36	0.43	0.52	0.49	0.64	0.71	0.82	0.79
18	0.23	0.32	0.44	0.39	0.56	0.58	0.51	0.64	0.64	0.73
19	0.30	0.29	0.31	0.52	0.54	0.66	0.62	0.73	0.82	0.82
20	0.16	0.26	0.31	0.48	0.57	0.62	0.65	0.62	0.76	0.75
21	0.17	0.25	0.38	0.44	0.48	0.58	0.61	0.76	0.82	0.82
22	0.09	0.22	0.31	0.44	0.57	0.65	0.81	0.83	0.88	0.92
23	0.14	0.20	0.28	0.41	0.53	0.67	0.73	0.77	0.83	0.82
24	0.23	0.18	0.32	0.34	0.43	0.53	0.58	0.70	0.70	0.79
25	0.10	0.17	0.24	0.31	0.60	0.54	0.69	0.80	0.92	0.89
26	0.16	0.23	0.29	0.42	0.48	0.53	0.69	0.60	0.72	0.79
27	0.27	0.27	0.43	0.44	0.47	0.57	0.50	0.62	0.65	0.61
28	0.03	0.11	0.17	0.37	0.52	0.63	0.70	0.86	0.87	0.94
29	0.23	0.25	0.40	0.41	0.45	0.47	0.58	0.60	0.61	0.64
30	0.30	0.37	0.35	0.46	0.46	0.41	0.51	0.50	0.57	0.62
31	0.06	0.10	0.19	0.36	0.42	0.63	0.82	0.82	0.87	0.94
32	0.08	0.08	0.25	0.21	0.42	0.59	0.68	0.80	0.84	0.88
33	0.11	0.17	0.34	0.31	0.47	0.68	0.72	0.78	0.78	0.87
34	0.11	0.11	0.21	0.32	0.42	0.67	0.80	0.87	0.91	0.96
35	0.06	0.22	0.22	0.25	0.52	0.58	0.81	0.82	0.92	0.93
36	0.16	0.18	0.28	0.46	0.47	0.57	0.70	0.74	0.74	0.82
37	0.09	0.18	0.25	0.34	0.51	0.53	0.70	0.76	0.92	0.95
38	0.02	0.14	0.18	0.34	0.39	0.62	0.75	0.84	0.90	0.93
39	0.05	0.17	0.24	0.24	0.47	0.58	0.69	0.76	0.85	0.95
40	0.04	0.15	0.15	0.33	0.47	0.61	0.76	0.82	0.81	0.91
41	0.08	0.08	0.20	0.23	0.32	0.66	0.81	0.78	0.85	0.91
42	0.07	0.08	0.25	0.31	0.55	0.63	0.68	0.79	0.82	0.92

Table 3: POD according to each scenario and each k2 value, k1 = 2, alpha = 0.001

	k2=1	k2=2	k2=3	k2=4	k2=5	k2=6	k2=7	k2=8	k2=9	k2=10
1	0.12	0.17	0.16	0.27	0.51	0.48	0.65	0.71	0.77	0.80
2	0.17	0.22	0.23	0.29	0.37	0.41	0.60	0.62	0.65	0.66
3	0.14	0.18	0.26	0.31	0.40	0.37	0.46	0.53	0.63	0.63
4	0.05	0.09	0.28	0.32	0.46	0.58	0.63	0.68	0.83	0.89
5	0.09	0.19	0.20	0.41	0.40	0.46	0.59	0.71	0.75	0.85
6	0.19	0.27	0.25	0.35	0.34	0.40	0.44	0.49	0.60	0.67
7	0.09	0.09	0.12	0.19	0.14	0.31	0.21	0.26	0.38	0.32
8	0.08	0.11	0.12	0.14	0.13	0.20	0.28	0.25	0.28	0.32
9	0.16	0.11	0.15	0.10	0.21	0.19	0.20	0.38	0.29	0.29
10	0.10	0.14	0.28	0.34	0.38	0.61	0.68	0.70	0.81	0.90
11	0.09	0.14	0.29	0.39	0.44	0.55	0.62	0.76	0.80	0.82
12	0.14	0.23	0.24	0.30	0.38	0.54	0.59	0.70	0.73	0.77
13	0.09	0.13	0.18	0.27	0.36	0.49	0.63	0.73	0.83	0.88
14	0.10	0.07	0.21	0.27	0.42	0.49	0.56	0.73	0.77	0.76
15	0.15	0.15	0.38	0.39	0.41	0.54	0.55	0.73	0.71	0.71
16	0.07	0.11	0.25	0.41	0.42	0.55	0.75	0.82	0.88	0.94
17	0.11	0.22	0.25	0.41	0.47	0.50	0.62	0.69	0.73	0.88
18	0.32	0.29	0.38	0.40	0.42	0.61	0.59	0.69	0.66	0.77
19	0.16	0.27	0.46	0.47	0.60	0.61	0.82	0.77	0.80	0.89
20	0.13	0.29	0.29	0.46	0.50	0.61	0.72	0.74	0.88	0.87
21	0.15	0.26	0.40	0.41	0.49	0.58	0.73	0.73	0.77	0.78
22	0.10	0.28	0.29	0.46	0.49	0.63	0.77	0.78	0.89	0.89
23	0.10	0.23	0.31	0.36	0.48	0.53	0.69	0.85	0.87	0.86
24	0.22	0.24	0.26	0.42	0.49	0.58	0.63	0.67	0.73	0.74
25	0.09	0.13	0.21	0.34	0.49	0.64	0.69	0.79	0.87	0.93
26	0.19	0.21	0.30	0.32	0.44	0.51	0.50	0.63	0.75	0.79
27	0.27	0.27	0.32	0.33	0.39	0.55	0.39	0.57	0.60	0.60
28	0.08	0.19	0.25	0.35	0.48	0.69	0.74	0.85	0.89	0.95
29	0.19	0.19	0.35	0.33	0.39	0.46	0.53	0.50	0.70	0.60
30	0.32	0.33	0.40	0.42	0.38	0.45	0.53	0.45	0.51	0.61
31	0.05	0.13	0.19	0.33	0.46	0.68	0.74	0.82	0.89	0.96
32	0.04	0.10	0.23	0.32	0.48	0.60	0.70	0.76	0.85	0.96
33	0.10	0.20	0.33	0.36	0.45	0.52	0.64	0.73	0.78	0.85
34	0.02	0.10	0.19	0.25	0.45	0.63	0.77	0.79	0.90	0.98
35	0.08	0.14	0.24	0.40	0.49	0.68	0.77	0.86	0.86	0.87
36	0.15	0.18	0.43	0.41	0.47	0.54	0.68	0.74	0.78	0.87
37	0.07	0.05	0.22	0.28	0.46	0.68	0.79	0.81	0.88	0.95
38	0.11	0.06	0.18	0.37	0.47	0.64	0.73	0.80	0.92	0.92
39	0.08	0.16	0.20	0.29	0.38	0.58	0.72	0.80	0.91	0.92
40	0.03	0.14	0.22	0.28	0.46	0.60	0.72	0.90	0.91	0.88
41	0.06	0.13	0.16	0.36	0.44	0.53	0.72	0.82	0.95	0.90
42	0.04	0.19	0.22	0.24	0.53	0.50	0.68	0.73	0.87	0.96

Table 4: POD according to each scenario and each k2 value, k1 = 3, alpha = 0.001

	k2=1	k2=2	k2=3	k2=4	k2=5	k2=6	k2=7	k2=8	k2=9	k2=10
1	0.07	0.21	0.27	0.31	0.49	0.46	0.63	0.75	0.75	0.82
2	0.18	0.10	0.35	0.34	0.39	0.44	0.51	0.61	0.64	0.75
3	0.17	0.24	0.26	0.37	0.43	0.38	0.51	0.58	0.56	0.66
4	0.11	0.11	0.14	0.32	0.39	0.45	0.67	0.79	0.83	0.93
5	0.12	0.17	0.27	0.30	0.35	0.54	0.49	0.68	0.75	0.76
6	0.21	0.15	0.31	0.33	0.31	0.40	0.52	0.45	0.58	0.52
7	0.06	0.11	0.10	0.09	0.13	0.22	0.17	0.25	0.25	0.35
8	0.12	0.14	0.12	0.15	0.20	0.18	0.19	0.23	0.26	0.27
9	0.17	0.10	0.08	0.15	0.25	0.21	0.26	0.30	0.38	0.43
10	0.11	0.20	0.23	0.41	0.54	0.65	0.74	0.76	0.88	0.85
11	0.17	0.14	0.26	0.33	0.48	0.58	0.63	0.73	0.75	0.85
12	0.11	0.18	0.25	0.40	0.44	0.53	0.57	0.79	0.80	0.82
13	0.05	0.14	0.30	0.27	0.38	0.48	0.71	0.72	0.75	0.83
14	0.07	0.12	0.24	0.38	0.50	0.55	0.58	0.71	0.77	0.89
15	0.13	0.31	0.26	0.35	0.50	0.50	0.55	0.73	0.72	0.81
16	0.07	0.09	0.21	0.32	0.49	0.59	0.67	0.80	0.89	0.93
17	0.17	0.19	0.36	0.38	0.50	0.57	0.64	0.62	0.79	0.82
18	0.32	0.41	0.34	0.35	0.48	0.54	0.65	0.68	0.63	0.74
19	0.19	0.28	0.38	0.46	0.60	0.60	0.77	0.80	0.83	0.86
20	0.19	0.27	0.32	0.43	0.62	0.63	0.70	0.66	0.86	0.85
21	0.22	0.26	0.27	0.44	0.52	0.62	0.63	0.72	0.78	0.85
22	0.14	0.26	0.31	0.36	0.52	0.61	0.76	0.76	0.89	0.90
23	0.12	0.16	0.35	0.48	0.56	0.63	0.69	0.75	0.89	0.84
24	0.23	0.29	0.27	0.39	0.49	0.56	0.58	0.57	0.74	0.79
25	0.12	0.11	0.18	0.38	0.47	0.68	0.59	0.88	0.88	0.93
26	0.15	0.23	0.38	0.39	0.52	0.51	0.59	0.65	0.64	0.83
27	0.27	0.29	0.39	0.42	0.47	0.49	0.56	0.64	0.54	0.59
28	0.09	0.14	0.22	0.34	0.50	0.58	0.74	0.85	0.87	0.96
29	0.25	0.24	0.30	0.29	0.47	0.59	0.54	0.69	0.71	0.72
30	0.31	0.30	0.41	0.29	0.42	0.48	0.42	0.49	0.55	0.59
31	0.04	0.10	0.26	0.27	0.48	0.64	0.75	0.78	0.87	0.95
32	0.07	0.10	0.22	0.37	0.39	0.62	0.71	0.75	0.89	0.90
33	0.13	0.20	0.26	0.32	0.49	0.54	0.65	0.77	0.78	0.91
34	0.06	0.14	0.17	0.27	0.58	0.56	0.75	0.79	0.91	0.94
35	0.09	0.12	0.22	0.37	0.54	0.65	0.76	0.84	0.91	0.89
36	0.10	0.18	0.40	0.38	0.51	0.55	0.61	0.76	0.72	0.81
37	0.01	0.12	0.18	0.31	0.51	0.60	0.79	0.87	0.89	0.97
38	0.03	0.14	0.14	0.28	0.45	0.61	0.80	0.79	0.94	0.94
39	0.09	0.11	0.26	0.26	0.38	0.59	0.68	0.78	0.86	0.87
40	0.03	0.06	0.17	0.30	0.42	0.50	0.75	0.82	0.89	0.91
41	0.09	0.12	0.27	0.27	0.43	0.62	0.76	0.87	0.92	0.95
42	0.07	0.15	0.17	0.36	0.46	0.53	0.74	0.81	0.75	0.85

Table 5: POD according to each scenario and each k2 value, k1 = 5, alpha = 0.001

	k2=1	k2=2	k2=3	k2=4	k2=5	k2=6	k2=7	k2=8	k2=9	k2=10
1	0.12	0.14	0.28	0.36	0.49	0.49	0.64	0.66	0.68	0.77
2	0.09	0.18	0.21	0.32	0.33	0.46	0.55	0.62	0.69	0.68
3	0.17	0.17	0.26	0.33	0.38	0.41	0.53	0.58	0.58	0.64
4	0.06	0.15	0.22	0.31	0.42	0.56	0.62	0.79	0.84	0.86
5	0.13	0.18	0.17	0.24	0.42	0.51	0.61	0.66	0.71	0.74
6	0.13	0.20	0.30	0.32	0.39	0.51	0.54	0.48	0.60	0.70
7	0.09	0.11	0.14	0.16	0.15	0.17	0.24	0.22	0.24	0.31
8	0.09	0.09	0.11	0.15	0.23	0.19	0.31	0.27	0.26	0.25
9	0.07	0.09	0.12	0.12	0.20	0.19	0.22	0.22	0.36	0.29
10	0.10	0.15	0.20	0.30	0.49	0.62	0.70	0.78	0.78	0.85
11	0.10	0.09	0.33	0.28	0.44	0.51	0.66	0.72	0.81	0.86
12	0.04	0.12	0.25	0.29	0.44	0.56	0.62	0.73	0.79	0.90
13	0.09	0.08	0.19	0.26	0.44	0.57	0.62	0.76	0.74	0.86
14	0.05	0.12	0.21	0.38	0.58	0.57	0.72	0.61	0.80	0.81
15	0.13	0.22	0.27	0.31	0.49	0.58	0.63	0.71	0.72	0.81
16	0.05	0.12	0.23	0.34	0.44	0.61	0.77	0.88	0.93	0.94
17	0.22	0.20	0.35	0.32	0.53	0.65	0.64	0.70	0.75	0.86
18	0.27	0.30	0.34	0.42	0.44	0.54	0.67	0.66	0.69	0.78
19	0.26	0.35	0.39	0.45	0.59	0.75	0.68	0.72	0.78	0.75
20	0.16	0.24	0.41	0.44	0.52	0.55	0.69	0.68	0.74	0.81
21	0.21	0.26	0.39	0.42	0.58	0.56	0.67	0.66	0.69	0.83
22	0.10	0.21	0.26	0.48	0.58	0.64	0.77	0.85	0.88	0.83
23	0.12	0.18	0.28	0.40	0.51	0.59	0.72	0.79	0.80	0.85
24	0.14	0.15	0.27	0.39	0.43	0.50	0.64	0.63	0.76	0.78
25	0.07	0.13	0.26	0.36	0.48	0.63	0.74	0.78	0.88	0.91
26	0.19	0.24	0.32	0.31	0.40	0.45	0.65	0.67	0.70	0.70
27	0.22	0.27	0.27	0.46	0.39	0.39	0.53	0.53	0.57	0.66
28	0.06	0.15	0.26	0.40	0.56	0.65	0.72	0.83	0.94	0.91
29	0.23	0.23	0.29	0.44	0.50	0.53	0.48	0.71	0.67	0.64
30	0.23	0.34	0.40	0.31	0.46	0.46	0.56	0.55	0.50	0.55
31	0.06	0.08	0.18	0.32	0.54	0.61	0.71	0.82	0.93	0.97
32	0.12	0.08	0.18	0.35	0.41	0.59	0.67	0.80	0.83	0.90
33	0.08	0.18	0.24	0.43	0.38	0.56	0.63	0.70	0.77	0.90
34	0.06	0.13	0.22	0.29	0.47	0.56	0.74	0.79	0.94	0.91
35	0.10	0.17	0.27	0.39	0.53	0.62	0.70	0.83	0.85	0.93
36	0.14	0.21	0.36	0.41	0.47	0.56	0.65	0.74	0.79	0.84
37	0.03	0.12	0.20	0.37	0.54	0.69	0.76	0.82	0.83	0.94
38	0.03	0.09	0.19	0.32	0.40	0.64	0.70	0.79	0.91	0.93
39	0.03	0.16	0.21	0.39	0.47	0.58	0.66	0.81	0.87	0.86
40	0.02	0.10	0.19	0.34	0.51	0.45	0.76	0.73	0.89	0.93
41	0.07	0.11	0.20	0.30	0.52	0.51	0.71	0.87	0.89	0.97
42	0.05	0.11	0.19	0.42	0.41	0.55	0.72	0.80	0.86	0.94

Table 6: POD according to each scenario and each k2 value, k1 = 10, alpha = 0.001

Overall performances of EARS C3 algorithm ($\alpha = 0.01$)

	FPR k1=0	FPR k1=2	FPR k1=3	FPR k1=5	FPR k1=10
1	0.05	0.05	0.05	0.05	0.05
2	0.06	0.06	0.06	0.06	0.06
3	0.09	0.09	0.09	0.09	0.09
4	0.05	0.05	0.05	0.05	0.05
5	0.08	0.08	0.08	0.08	0.08
6	0.14	0.14	0.14	0.14	0.14
7	0.00	0.00	0.00	0.00	0.00
8	0.00	0.00	0.00	0.00	0.00
9	0.00	0.00	0.00	0.00	0.00
10	0.07	0.07	0.07	0.06	0.07
11	0.07	0.07	0.07	0.07	0.07
12	0.08	0.08	0.07	0.07	0.07
13	0.04	0.04	0.04	0.04	0.04
14	0.06	0.06	0.06	0.06	0.06
15	0.12	0.12	0.12	0.12	0.12
16	0.03	0.03	0.03	0.03	0.03
17	0.11	0.11	0.11	0.11	0.11
18	0.23	0.23	0.23	0.23	0.23
19	0.06	0.06	0.06	0.06	0.06
20	0.06	0.06	0.06	0.06	0.06
21	0.07	0.07	0.07	0.07	0.07
22	0.08	0.08	0.08	0.08	0.08
23	0.09	0.09	0.09	0.09	0.09
24	0.11	0.11	0.11	0.11	0.11
25	0.06	0.06	0.06	0.06	0.06
26	0.13	0.13	0.13	0.13	0.13
27	0.21	0.21	0.21	0.21	0.21
28	0.05	0.04	0.04	0.04	0.04
29	0.16	0.16	0.16	0.16	0.16
30	0.22	0.22	0.22	0.22	0.22
31	0.04	0.04	0.04	0.04	0.04
32	0.04	0.04	0.04	0.04	0.04
33	0.07	0.07	0.07	0.07	0.07
34	0.04	0.04	0.04	0.04	0.04
35	0.05	0.05	0.05	0.05	0.05
36	0.11	0.11	0.11	0.11	0.11
37	0.03	0.03	0.03	0.03	0.03
38	0.03	0.03	0.03	0.03	0.03
39	0.04	0.04	0.04	0.04	0.04
40	0.03	0.03	0.03	0.03	0.03
41	0.03	0.03	0.03	0.03	0.03
42	0.04	0.04	0.04	0.04	0.04

Table 7: FPR according to each scenario and each k1 value, alpha = 0.01

	k2=1	k2=2	k2=3	k2=4	k2=5	k2=6	k2=7	k2=8	k2=9	k2=10
1	0.14	0.22	0.25	0.31	0.50	0.54	0.77	0.82	0.83	0.86
2	0.21	0.24	0.24	0.36	0.50	0.53	0.58	0.71	0.81	0.88
3	0.26	0.21	0.33	0.35	0.39	0.44	0.49	0.59	0.63	0.81
4	0.08	0.13	0.25	0.39	0.52	0.68	0.79	0.87	0.90	0.91
5	0.17	0.20	0.35	0.33	0.46	0.57	0.71	0.73	0.84	0.85
6	0.18	0.27	0.29	0.39	0.46	0.49	0.60	0.60	0.62	0.64
7	0.07	0.17	0.17	0.13	0.30	0.30	0.22	0.27	0.34	0.33
8	0.07	0.11	0.10	0.19	0.20	0.25	0.31	0.31	0.27	0.34
9	0.12	0.16	0.19	0.17	0.21	0.28	0.33	0.37	0.29	0.37
10	0.19	0.23	0.29	0.48	0.67	0.66	0.81	0.80	0.87	0.96
11	0.18	0.20	0.34	0.48	0.55	0.77	0.77	0.79	0.83	0.90
12	0.15	0.24	0.31	0.45	0.50	0.70	0.77	0.80	0.86	0.88
13	0.11	0.13	0.22	0.45	0.49	0.66	0.62	0.79	0.90	0.91
14	0.14	0.19	0.32	0.41	0.48	0.60	0.74	0.85	0.88	0.91
15	0.32	0.34	0.33	0.46	0.48	0.60	0.65	0.72	0.80	0.80
16	0.09	0.25	0.29	0.40	0.56	0.75	0.82	0.92	0.93	0.98
17	0.28	0.18	0.36	0.60	0.55	0.68	0.74	0.74	0.84	0.90
18	0.37	0.39	0.51	0.44	0.62	0.56	0.71	0.70	0.75	0.75
19	0.23	0.34	0.49	0.54	0.69	0.67	0.71	0.85	0.91	0.93
20	0.24	0.33	0.40	0.48	0.61	0.72	0.69	0.75	0.84	0.87
21	0.23	0.28	0.37	0.48	0.56	0.71	0.76	0.74	0.89	0.82
22	0.11	0.28	0.33	0.49	0.65	0.62	0.83	0.92	0.94	0.95
23	0.16	0.37	0.45	0.47	0.67	0.72	0.75	0.82	0.87	0.84
24	0.27	0.27	0.38	0.45	0.53	0.52	0.72	0.69	0.81	0.84
25	0.15	0.24	0.37	0.45	0.65	0.72	0.86	0.86	0.93	0.95
26	0.30	0.27	0.37	0.51	0.49	0.61	0.59	0.73	0.78	0.86
27	0.27	0.37	0.38	0.48	0.42	0.49	0.55	0.59	0.57	0.65
28	0.11	0.24	0.34	0.53	0.56	0.69	0.81	0.89	0.93	0.97
29	0.27	0.32	0.41	0.42	0.52	0.50	0.68	0.67	0.77	0.76
30	0.28	0.32	0.24	0.36	0.49	0.52	0.51	0.49	0.57	0.64
31	0.13	0.19	0.34	0.43	0.64	0.77	0.86	0.94	0.90	0.98
32	0.11	0.13	0.30	0.45	0.54	0.77	0.82	0.88	0.95	0.97
33	0.11	0.28	0.36	0.46	0.53	0.72	0.74	0.81	0.88	0.91
34	0.08	0.17	0.26	0.40	0.70	0.75	0.83	0.92	0.98	0.98
35	0.11	0.19	0.36	0.49	0.62	0.73	0.83	0.89	0.97	0.93
36	0.19	0.23	0.35	0.44	0.65	0.64	0.80	0.83	0.80	0.85
37	0.09	0.16	0.21	0.50	0.57	0.62	0.90	0.92	0.95	0.96
38	0.12	0.19	0.35	0.49	0.62	0.74	0.79	0.95	0.95	0.94
39	0.09	0.20	0.33	0.45	0.56	0.67	0.76	0.83	0.94	0.95
40	0.10	0.15	0.32	0.49	0.59	0.67	0.81	0.95	0.96	0.97
41	0.12	0.18	0.30	0.52	0.65	0.77	0.82	0.85	0.98	0.98
42	0.12	0.20	0.24	0.43	0.55	0.66	0.82	0.80	0.86	0.95

Table 8: POD according to each scenario and each k2 value, k1 = 0, alpha = 0.01

	k2=1	k2=2	k2=3	k2=4	k2=5	k2=6	k2=7	k2=8	k2=9	k2=10
1	0.17	0.21	0.35	0.36	0.48	0.52	0.66	0.76	0.79	0.85
2	0.19	0.30	0.29	0.45	0.50	0.59	0.70	0.73	0.74	0.84
3	0.25	0.24	0.24	0.44	0.38	0.56	0.57	0.63	0.68	0.77
4	0.11	0.24	0.19	0.39	0.53	0.61	0.75	0.84	0.89	0.91
5	0.12	0.27	0.34	0.43	0.41	0.55	0.71	0.75	0.83	0.84
6	0.20	0.32	0.39	0.36	0.42	0.43	0.60	0.56	0.71	0.74
7	0.10	0.11	0.18	0.14	0.15	0.26	0.28	0.29	0.35	0.37
8	0.15	0.12	0.13	0.17	0.26	0.22	0.29	0.33	0.27	0.27
9	0.10	0.13	0.12	0.20	0.24	0.28	0.29	0.26	0.25	0.46
10	0.17	0.22	0.32	0.37	0.60	0.69	0.83	0.77	0.91	0.96
11	0.08	0.20	0.42	0.37	0.55	0.57	0.72	0.80	0.87	0.88
12	0.16	0.30	0.24	0.37	0.54	0.62	0.79	0.78	0.88	0.92
13	0.11	0.15	0.27	0.41	0.55	0.61	0.74	0.82	0.89	0.92
14	0.12	0.20	0.30	0.40	0.47	0.62	0.73	0.85	0.85	0.90
15	0.31	0.34	0.36	0.55	0.57	0.57	0.61	0.76	0.72	0.85
16	0.09	0.22	0.29	0.46	0.59	0.77	0.84	0.86	0.98	0.98
17	0.19	0.26	0.46	0.54	0.58	0.59	0.71	0.80	0.83	0.83
18	0.32	0.39	0.50	0.46	0.63	0.61	0.57	0.69	0.69	0.81
19	0.33	0.35	0.40	0.58	0.63	0.70	0.70	0.83	0.84	0.87
20	0.24	0.31	0.33	0.53	0.63	0.68	0.75	0.71	0.83	0.82
21	0.20	0.30	0.43	0.50	0.53	0.67	0.68	0.82	0.87	0.86
22	0.14	0.29	0.36	0.50	0.70	0.73	0.88	0.95	0.91	0.95
23	0.21	0.25	0.36	0.51	0.58	0.76	0.80	0.83	0.92	0.88
24	0.30	0.23	0.38	0.41	0.49	0.60	0.67	0.77	0.74	0.83
25	0.17	0.21	0.33	0.39	0.70	0.64	0.82	0.83	0.95	0.98
26	0.22	0.25	0.36	0.48	0.53	0.56	0.75	0.66	0.78	0.86
27	0.30	0.30	0.51	0.47	0.54	0.65	0.58	0.66	0.67	0.63
28	0.07	0.14	0.30	0.44	0.63	0.71	0.80	0.92	0.95	0.96
29	0.33	0.31	0.42	0.46	0.48	0.55	0.62	0.64	0.70	0.70
30	0.34	0.42	0.39	0.50	0.48	0.44	0.58	0.52	0.63	0.62
31	0.10	0.17	0.32	0.53	0.57	0.72	0.90	0.88	0.97	0.97
32	0.13	0.15	0.38	0.37	0.56	0.67	0.80	0.83	0.89	0.93
33	0.18	0.24	0.45	0.47	0.57	0.73	0.79	0.82	0.85	0.90
34	0.15	0.21	0.32	0.46	0.57	0.77	0.89	0.92	0.96	0.96
35	0.15	0.26	0.33	0.44	0.66	0.67	0.88	0.92	0.96	0.97
36	0.24	0.21	0.41	0.55	0.55	0.66	0.76	0.81	0.77	0.84
37	0.11	0.23	0.31	0.50	0.70	0.74	0.83	0.87	0.94	0.98
38	0.06	0.22	0.30	0.47	0.48	0.78	0.83	0.94	0.93	0.94
39	0.08	0.24	0.32	0.42	0.55	0.73	0.81	0.90	0.90	0.98
40	0.07	0.25	0.25	0.48	0.64	0.71	0.85	0.97	0.89	0.97
41	0.12	0.11	0.36	0.42	0.43	0.80	0.89	0.85	0.93	0.97
42	0.10	0.13	0.35	0.42	0.71	0.75	0.76	0.84	0.88	0.94

Table 9: POD according to each scenario and each k2 value, k1 = 2, alpha = 0.01

	k2=1	k2=2	k2=3	k2=4	k2=5	k2=6	k2=7	k2=8	k2=9	k2=10
1	0.19	0.21	0.21	0.39	0.60	0.56	0.79	0.76	0.81	0.87
2	0.23	0.25	0.29	0.35	0.45	0.51	0.66	0.72	0.75	0.71
3	0.20	0.28	0.34	0.39	0.48	0.47	0.52	0.59	0.71	0.67
4	0.10	0.19	0.31	0.40	0.54	0.62	0.67	0.77	0.92	0.92
5	0.17	0.23	0.26	0.49	0.47	0.55	0.63	0.84	0.80	0.89
6	0.26	0.29	0.28	0.39	0.41	0.45	0.48	0.56	0.66	0.73
7	0.13	0.12	0.12	0.24	0.16	0.37	0.26	0.27	0.39	0.35
8	0.09	0.12	0.16	0.20	0.15	0.24	0.31	0.27	0.29	0.37
9	0.17	0.14	0.17	0.11	0.25	0.22	0.28	0.40	0.34	0.31
10	0.15	0.24	0.36	0.45	0.49	0.70	0.81	0.80	0.88	0.94
11	0.17	0.17	0.35	0.50	0.56	0.68	0.70	0.84	0.86	0.90
12	0.18	0.28	0.28	0.39	0.50	0.63	0.68	0.77	0.81	0.86
13	0.12	0.18	0.23	0.35	0.52	0.59	0.72	0.80	0.90	0.94
14	0.15	0.13	0.29	0.39	0.49	0.57	0.68	0.84	0.86	0.84
15	0.26	0.22	0.48	0.53	0.50	0.60	0.63	0.77	0.82	0.76
16	0.09	0.17	0.38	0.46	0.55	0.70	0.84	0.86	0.93	0.96
17	0.17	0.33	0.35	0.52	0.53	0.63	0.68	0.81	0.80	0.93
18	0.34	0.32	0.45	0.47	0.48	0.62	0.65	0.73	0.68	0.81
19	0.23	0.36	0.48	0.56	0.65	0.68	0.85	0.83	0.83	0.94
20	0.19	0.34	0.33	0.50	0.58	0.63	0.82	0.80	0.89	0.92
21	0.19	0.32	0.46	0.47	0.56	0.63	0.77	0.78	0.78	0.83
22	0.17	0.30	0.34	0.56	0.63	0.73	0.85	0.81	0.92	0.92
23	0.15	0.27	0.37	0.42	0.54	0.59	0.77	0.89	0.92	0.90
24	0.23	0.28	0.32	0.50	0.59	0.66	0.68	0.72	0.76	0.81
25	0.12	0.19	0.27	0.44	0.63	0.71	0.80	0.86	0.93	0.96
26	0.28	0.26	0.38	0.41	0.49	0.58	0.59	0.69	0.80	0.87
27	0.32	0.31	0.37	0.41	0.44	0.56	0.44	0.60	0.64	0.67
28	0.12	0.21	0.33	0.48	0.61	0.80	0.87	0.96	0.92	0.96
29	0.23	0.25	0.43	0.41	0.46	0.51	0.59	0.57	0.76	0.65
30	0.32	0.37	0.45	0.43	0.42	0.48	0.53	0.49	0.52	0.63
31	0.10	0.21	0.31	0.43	0.60	0.81	0.77	0.87	0.94	0.98
32	0.08	0.17	0.33	0.41	0.54	0.71	0.84	0.88	0.91	0.98
33	0.16	0.26	0.45	0.49	0.57	0.63	0.70	0.78	0.83	0.88
34	0.10	0.15	0.27	0.38	0.57	0.75	0.85	0.88	0.94	0.99
35	0.14	0.25	0.36	0.50	0.59	0.75	0.90	0.88	0.92	0.91
36	0.22	0.28	0.48	0.51	0.54	0.56	0.79	0.80	0.83	0.90
37	0.11	0.09	0.33	0.36	0.59	0.73	0.87	0.93	0.94	0.99
38	0.15	0.16	0.28	0.48	0.58	0.77	0.86	0.83	0.99	0.94
39	0.13	0.22	0.27	0.44	0.47	0.73	0.77	0.88	0.95	0.96
40	0.13	0.22	0.31	0.35	0.65	0.73	0.84	0.93	0.94	0.94
41	0.10	0.19	0.26	0.49	0.60	0.67	0.84	0.92	0.96	0.94
42	0.07	0.29	0.29	0.37	0.63	0.64	0.81	0.83	0.93	0.99

Table 10: POD according to each scenario and each k2 value, k1 = 3, alpha = 0.01

	k2=1	k2=2	k2=3	k2=4	k2=5	k2=6	k2=7	k2=8	k2=9	k2=10
1	0.14	0.30	0.36	0.36	0.56	0.52	0.69	0.82	0.84	0.86
2	0.23	0.18	0.36	0.41	0.50	0.54	0.57	0.70	0.74	0.80
3	0.19	0.27	0.34	0.39	0.50	0.47	0.62	0.59	0.59	0.74
4	0.17	0.20	0.24	0.43	0.49	0.61	0.73	0.85	0.89	0.96
5	0.18	0.24	0.35	0.38	0.41	0.66	0.59	0.75	0.81	0.80
6	0.26	0.24	0.36	0.36	0.36	0.42	0.59	0.52	0.64	0.56
7	0.10	0.11	0.11	0.14	0.15	0.25	0.18	0.29	0.29	0.36
8	0.13	0.18	0.14	0.19	0.25	0.18	0.23	0.23	0.32	0.30
9	0.18	0.10	0.11	0.18	0.32	0.26	0.28	0.34	0.39	0.46
10	0.18	0.24	0.36	0.47	0.65	0.70	0.78	0.79	0.90	0.92
11	0.22	0.20	0.36	0.41	0.55	0.69	0.70	0.85	0.80	0.89
12	0.15	0.26	0.33	0.45	0.53	0.65	0.66	0.83	0.88	0.89
13	0.12	0.18	0.41	0.36	0.50	0.65	0.77	0.82	0.84	0.92
14	0.09	0.15	0.38	0.49	0.61	0.62	0.68	0.81	0.83	0.95
15	0.20	0.33	0.40	0.44	0.57	0.58	0.63	0.78	0.78	0.85
16	0.11	0.13	0.32	0.48	0.61	0.73	0.77	0.92	0.95	0.95
17	0.21	0.32	0.49	0.51	0.59	0.71	0.72	0.69	0.80	0.84
18	0.40	0.46	0.38	0.41	0.55	0.60	0.72	0.74	0.72	0.79
19	0.23	0.36	0.43	0.53	0.64	0.72	0.80	0.87	0.87	0.90
20	0.24	0.32	0.37	0.53	0.68	0.73	0.84	0.74	0.92	0.89
21	0.24	0.35	0.31	0.48	0.57	0.67	0.72	0.77	0.82	0.90
22	0.20	0.33	0.43	0.50	0.62	0.72	0.83	0.84	0.93	0.93
23	0.19	0.26	0.40	0.56	0.63	0.71	0.77	0.84	0.93	0.90
24	0.29	0.38	0.36	0.43	0.56	0.61	0.62	0.66	0.81	0.86
25	0.16	0.20	0.29	0.51	0.56	0.74	0.76	0.92	0.90	0.96
26	0.19	0.27	0.45	0.49	0.58	0.58	0.62	0.69	0.71	0.87
27	0.32	0.30	0.41	0.47	0.52	0.50	0.60	0.66	0.59	0.62
28	0.15	0.22	0.34	0.47	0.57	0.72	0.78	0.86	0.92	0.97
29	0.32	0.26	0.35	0.35	0.51	0.62	0.60	0.70	0.78	0.76
30	0.35	0.33	0.43	0.33	0.44	0.48	0.45	0.55	0.57	0.62
31	0.11	0.19	0.35	0.35	0.57	0.71	0.85	0.85	0.94	0.97
32	0.09	0.18	0.34	0.46	0.58	0.69	0.81	0.77	0.94	0.93
33	0.19	0.27	0.38	0.46	0.58	0.64	0.75	0.83	0.84	0.93
34	0.08	0.17	0.25	0.41	0.69	0.68	0.87	0.93	0.93	0.97
35	0.11	0.23	0.34	0.47	0.64	0.73	0.84	0.89	0.93	0.93
36	0.21	0.27	0.54	0.55	0.59	0.63	0.70	0.82	0.79	0.82
37	0.07	0.17	0.25	0.45	0.59	0.78	0.85	0.91	0.94	0.98
38	0.07	0.20	0.24	0.40	0.69	0.75	0.89	0.87	0.96	0.95
39	0.14	0.15	0.34	0.36	0.47	0.71	0.75	0.86	0.90	0.93
40	0.08	0.11	0.26	0.44	0.57	0.63	0.91	0.87	0.92	0.93
41	0.16	0.20	0.42	0.48	0.56	0.73	0.84	0.94	0.93	0.97
42	0.15	0.22	0.23	0.45	0.59	0.67	0.85	0.91	0.83	0.92

Table 11: POD according to each scenario and each k2 value, k1 = 5, alpha = 0.01

	k2=1	k2=2	k2=3	k2=4	k2=5	k2=6	k2=7	k2=8	k2=9	k2=10
1	0.14	0.23	0.32	0.47	0.56	0.61	0.76	0.71	0.72	0.84
2	0.16	0.26	0.24	0.36	0.43	0.54	0.67	0.75	0.75	0.76
3	0.21	0.21	0.35	0.39	0.42	0.49	0.63	0.64	0.63	0.71
4	0.12	0.19	0.29	0.44	0.50	0.66	0.73	0.85	0.89	0.91
5	0.17	0.24	0.20	0.31	0.49	0.62	0.67	0.75	0.77	0.80
6	0.19	0.25	0.35	0.38	0.44	0.54	0.60	0.52	0.67	0.74
7	0.11	0.13	0.19	0.20	0.17	0.19	0.27	0.27	0.29	0.36
8	0.09	0.10	0.12	0.16	0.25	0.19	0.32	0.28	0.29	0.29
9	0.09	0.11	0.21	0.14	0.23	0.21	0.26	0.25	0.40	0.32
10	0.14	0.20	0.28	0.38	0.61	0.72	0.75	0.85	0.85	0.92
11	0.17	0.16	0.38	0.41	0.54	0.57	0.70	0.76	0.85	0.94
12	0.09	0.22	0.33	0.38	0.55	0.64	0.70	0.78	0.86	0.94
13	0.15	0.16	0.33	0.33	0.53	0.65	0.70	0.82	0.86	0.90
14	0.11	0.17	0.23	0.46	0.67	0.64	0.78	0.73	0.86	0.87
15	0.18	0.28	0.33	0.42	0.53	0.70	0.70	0.78	0.78	0.86
16	0.10	0.21	0.32	0.41	0.57	0.72	0.86	0.93	0.96	0.98
17	0.28	0.31	0.44	0.41	0.58	0.71	0.72	0.73	0.78	0.92
18	0.37	0.35	0.39	0.52	0.52	0.59	0.74	0.69	0.72	0.80
19	0.29	0.39	0.48	0.51	0.70	0.81	0.71	0.82	0.84	0.82
20	0.17	0.29	0.48	0.52	0.62	0.62	0.72	0.75	0.82	0.84
21	0.27	0.29	0.43	0.46	0.63	0.64	0.71	0.72	0.76	0.84
22	0.12	0.27	0.39	0.55	0.66	0.73	0.81	0.90	0.94	0.92
23	0.16	0.23	0.40	0.45	0.55	0.66	0.78	0.87	0.88	0.90
24	0.23	0.21	0.31	0.46	0.52	0.58	0.66	0.67	0.80	0.83
25	0.10	0.25	0.35	0.46	0.60	0.68	0.83	0.86	0.90	0.93
26	0.23	0.28	0.36	0.36	0.46	0.51	0.72	0.75	0.71	0.73
27	0.23	0.31	0.30	0.48	0.43	0.49	0.56	0.60	0.63	0.69
28	0.11	0.21	0.35	0.50	0.63	0.72	0.79	0.88	0.98	0.97
29	0.30	0.28	0.33	0.49	0.55	0.58	0.55	0.80	0.73	0.72
30	0.25	0.39	0.41	0.36	0.49	0.47	0.58	0.58	0.56	0.56
31	0.10	0.15	0.32	0.46	0.63	0.76	0.79	0.89	0.96	0.99
32	0.17	0.17	0.29	0.45	0.54	0.71	0.81	0.85	0.89	0.93
33	0.17	0.26	0.33	0.55	0.49	0.70	0.72	0.80	0.78	0.96
34	0.09	0.20	0.35	0.46	0.58	0.68	0.79	0.88	0.98	0.94
35	0.14	0.29	0.37	0.50	0.65	0.72	0.80	0.87	0.91	0.95
36	0.18	0.32	0.42	0.44	0.54	0.63	0.71	0.78	0.83	0.87
37	0.07	0.24	0.33	0.47	0.62	0.77	0.86	0.85	0.88	1.00
38	0.11	0.20	0.30	0.45	0.51	0.77	0.85	0.85	0.95	0.93
39	0.06	0.19	0.28	0.50	0.60	0.68	0.79	0.85	0.89	0.93
40	0.04	0.18	0.27	0.50	0.63	0.60	0.82	0.83	0.95	0.95
41	0.10	0.18	0.27	0.41	0.66	0.62	0.81	0.91	0.91	0.98
42	0.10	0.15	0.29	0.51	0.52	0.67	0.79	0.84	0.90	0.95

Table 12: POD according to each scenario and each k2 value, k1 = 10, alpha = 0.01

Overall performances of EARS C3 algorithm ($\alpha = 0.05$)

	FPR k1=0	FPR k1=2	FPR k1=3	FPR k1=5	FPR k1=10
1	0.08	0.07	0.07	0.08	0.07
2	0.08	0.08	0.08	0.08	0.08
3	0.11	0.11	0.11	0.11	0.11
4	0.09	0.09	0.09	0.09	0.09
5	0.13	0.13	0.13	0.13	0.13
6	0.18	0.18	0.18	0.18	0.18
7	0.00	0.00	0.00	0.00	0.00
8	0.00	0.00	0.00	0.00	0.00
9	0.00	0.00	0.00	0.00	0.00
10	0.10	0.10	0.09	0.09	0.10
11	0.10	0.10	0.10	0.10	0.10
12	0.11	0.11	0.11	0.11	0.10
13	0.08	0.08	0.08	0.08	0.08
14	0.10	0.10	0.10	0.10	0.10
15	0.17	0.17	0.17	0.17	0.17
16	0.07	0.07	0.07	0.07	0.07
17	0.17	0.17	0.17	0.17	0.17
18	0.29	0.29	0.29	0.29	0.29
19	0.08	0.08	0.08	0.07	0.08
20	0.08	0.08	0.07	0.07	0.07
21	0.09	0.09	0.09	0.09	0.09
22	0.12	0.12	0.12	0.12	0.12
23	0.13	0.13	0.13	0.13	0.13
24	0.14	0.14	0.14	0.14	0.14
25	0.10	0.10	0.10	0.09	0.10
26	0.18	0.18	0.18	0.18	0.18
27	0.24	0.24	0.24	0.24	0.24
28	0.08	0.08	0.08	0.08	0.08
29	0.21	0.21	0.21	0.21	0.21
30	0.25	0.25	0.25	0.25	0.26
31	0.07	0.07	0.07	0.07	0.07
32	0.08	0.08	0.08	0.08	0.08
33	0.12	0.12	0.12	0.12	0.12
34	0.07	0.07	0.07	0.07	0.07
35	0.09	0.09	0.09	0.09	0.09
36	0.16	0.16	0.16	0.16	0.16
37	0.06	0.06	0.06	0.06	0.06
38	0.06	0.06	0.06	0.06	0.06
39	0.08	0.08	0.08	0.08	0.08
40	0.06	0.06	0.06	0.06	0.06
41	0.07	0.07	0.07	0.07	0.07
42	0.07	0.08	0.08	0.08	0.08

Table 13: FPR according to each scenario and each k1 value, alpha = 0.05

	k2=1	k2=2	k2=3	k2=4	k2=5	k2=6	k2=7	k2=8	k2=9	k2=10
1	0.25	0.31	0.32	0.42	0.59	0.64	0.80	0.90	0.84	0.93
2	0.26	0.34	0.34	0.44	0.64	0.66	0.68	0.76	0.83	0.92
3	0.31	0.31	0.42	0.42	0.45	0.53	0.56	0.64	0.69	0.84
4	0.13	0.24	0.37	0.47	0.70	0.80	0.87	0.95	0.95	0.94
5	0.23	0.29	0.41	0.48	0.57	0.61	0.78	0.77	0.89	0.90
6	0.29	0.42	0.35	0.45	0.54	0.55	0.66	0.67	0.72	0.68
7	0.07	0.17	0.17	0.13	0.32	0.32	0.23	0.30	0.35	0.33
8	0.08	0.12	0.13	0.22	0.20	0.25	0.34	0.32	0.28	0.38
9	0.12	0.16	0.21	0.18	0.22	0.28	0.35	0.39	0.32	0.39
10	0.24	0.33	0.36	0.60	0.74	0.76	0.87	0.88	0.93	0.98
11	0.24	0.35	0.44	0.58	0.71	0.81	0.84	0.84	0.89	0.93
12	0.22	0.30	0.41	0.58	0.62	0.78	0.83	0.91	0.90	0.93
13	0.21	0.20	0.34	0.56	0.68	0.78	0.73	0.86	0.96	0.94
14	0.21	0.32	0.40	0.49	0.59	0.68	0.84	0.88	0.94	0.97
15	0.36	0.41	0.43	0.55	0.56	0.65	0.73	0.80	0.82	0.80
16	0.19	0.36	0.47	0.54	0.71	0.82	0.86	0.93	0.93	0.99
17	0.40	0.32	0.45	0.69	0.63	0.72	0.75	0.80	0.86	0.93
18	0.47	0.49	0.57	0.50	0.66	0.64	0.74	0.72	0.82	0.78
19	0.26	0.40	0.53	0.62	0.75	0.76	0.82	0.89	0.98	0.95
20	0.27	0.39	0.45	0.54	0.71	0.76	0.78	0.82	0.89	0.89
21	0.28	0.33	0.44	0.61	0.60	0.74	0.81	0.77	0.94	0.86
22	0.18	0.39	0.47	0.58	0.72	0.76	0.87	0.96	0.95	0.98
23	0.24	0.44	0.55	0.58	0.73	0.80	0.83	0.88	0.92	0.93
24	0.34	0.35	0.39	0.53	0.56	0.63	0.73	0.74	0.87	0.89
25	0.20	0.29	0.47	0.64	0.75	0.85	0.90	0.90	0.96	0.99
26	0.36	0.34	0.45	0.56	0.59	0.69	0.64	0.77	0.84	0.88
27	0.32	0.39	0.42	0.50	0.45	0.55	0.60	0.65	0.61	0.70
28	0.18	0.33	0.43	0.64	0.67	0.81	0.91	0.94	0.96	0.98
29	0.37	0.39	0.47	0.48	0.59	0.55	0.71	0.72	0.80	0.81
30	0.32	0.32	0.31	0.39	0.53	0.55	0.55	0.51	0.61	0.68
31	0.16	0.30	0.48	0.59	0.74	0.88	0.92	0.96	0.95	0.98
32	0.17	0.23	0.43	0.54	0.65	0.82	0.88	0.93	0.97	0.99
33	0.20	0.38	0.51	0.56	0.66	0.78	0.76	0.88	0.95	0.95
34	0.12	0.24	0.38	0.58	0.79	0.87	0.91	0.95	0.98	1.00
35	0.24	0.31	0.46	0.64	0.70	0.80	0.89	0.90	0.97	0.96
36	0.29	0.30	0.42	0.52	0.70	0.72	0.82	0.86	0.83	0.91
37	0.18	0.29	0.32	0.65	0.68	0.73	0.92	0.94	0.98	0.97
38	0.17	0.31	0.46	0.61	0.74	0.83	0.89	0.99	0.97	0.96
39	0.19	0.33	0.44	0.59	0.69	0.82	0.87	0.91	0.95	0.95
40	0.15	0.23	0.43	0.64	0.66	0.83	0.89	0.97	0.99	0.99
41	0.17	0.28	0.34	0.64	0.75	0.89	0.91	0.94	0.99	0.99
42	0.17	0.27	0.36	0.56	0.67	0.77	0.89	0.87	0.90	0.97

Table 14: POD according to each scenario and each k2 value, k1 = 0, alpha = 0.05

	k2=1	k2=2	k2=3	k2=4	k2=5	k2=6	k2=7	k2=8	k2=9	k2=10
1	0.27	0.33	0.40	0.48	0.61	0.67	0.77	0.85	0.87	0.87
2	0.24	0.38	0.35	0.52	0.57	0.67	0.75	0.79	0.83	0.90
3	0.29	0.27	0.30	0.46	0.48	0.60	0.62	0.66	0.71	0.79
4	0.13	0.35	0.34	0.53	0.62	0.69	0.82	0.94	0.93	0.92
5	0.20	0.36	0.44	0.49	0.47	0.65	0.77	0.77	0.87	0.86
6	0.27	0.38	0.44	0.39	0.48	0.51	0.63	0.66	0.74	0.76
7	0.11	0.12	0.18	0.17	0.17	0.28	0.31	0.30	0.35	0.39
8	0.15	0.13	0.13	0.19	0.26	0.24	0.30	0.33	0.29	0.29
9	0.10	0.13	0.12	0.21	0.24	0.29	0.30	0.27	0.28	0.46
10	0.25	0.31	0.44	0.51	0.70	0.75	0.90	0.87	0.94	0.97
11	0.14	0.26	0.50	0.49	0.64	0.67	0.78	0.90	0.93	0.93
12	0.19	0.39	0.32	0.49	0.67	0.76	0.85	0.83	0.91	0.94
13	0.16	0.26	0.35	0.55	0.63	0.75	0.85	0.91	0.94	0.95
14	0.22	0.33	0.40	0.49	0.59	0.73	0.81	0.90	0.90	0.92
15	0.38	0.37	0.48	0.59	0.66	0.64	0.68	0.81	0.78	0.88
16	0.15	0.34	0.41	0.58	0.74	0.82	0.91	0.95	0.99	1.00
17	0.28	0.33	0.53	0.63	0.69	0.71	0.80	0.83	0.90	0.90
18	0.38	0.46	0.55	0.50	0.68	0.66	0.62	0.73	0.75	0.83
19	0.38	0.40	0.51	0.68	0.73	0.77	0.81	0.90	0.89	0.89
20	0.28	0.36	0.42	0.58	0.69	0.75	0.83	0.82	0.89	0.84
21	0.24	0.34	0.51	0.54	0.60	0.74	0.74	0.88	0.92	0.89
22	0.19	0.39	0.48	0.63	0.78	0.78	0.95	0.97	0.96	0.96
23	0.26	0.31	0.45	0.62	0.64	0.82	0.88	0.88	0.93	0.89
24	0.34	0.28	0.47	0.53	0.56	0.67	0.75	0.83	0.84	0.86
25	0.27	0.30	0.42	0.54	0.77	0.78	0.87	0.92	0.97	1.00
26	0.27	0.33	0.41	0.54	0.58	0.65	0.81	0.75	0.82	0.89
27	0.32	0.33	0.52	0.51	0.59	0.67	0.62	0.72	0.71	0.68
28	0.15	0.28	0.38	0.57	0.71	0.79	0.87	0.97	0.96	0.97
29	0.41	0.37	0.46	0.51	0.53	0.62	0.68	0.71	0.80	0.78
30	0.44	0.44	0.42	0.55	0.51	0.46	0.60	0.56	0.65	0.63
31	0.19	0.28	0.44	0.63	0.71	0.79	0.95	0.92	0.98	1.00
32	0.24	0.24	0.55	0.48	0.70	0.82	0.86	0.89	0.91	0.94
33	0.26	0.32	0.55	0.59	0.66	0.78	0.85	0.85	0.92	0.94
34	0.28	0.31	0.47	0.62	0.76	0.86	0.92	0.95	0.98	0.98
35	0.23	0.36	0.44	0.63	0.76	0.77	0.92	0.95	0.97	0.98
36	0.33	0.30	0.49	0.62	0.62	0.70	0.81	0.85	0.82	0.86
37	0.24	0.32	0.47	0.62	0.82	0.82	0.94	0.92	0.98	0.98
38	0.10	0.33	0.41	0.55	0.67	0.86	0.92	0.96	0.96	0.95
39	0.15	0.32	0.46	0.58	0.68	0.82	0.88	0.94	0.92	0.98
40	0.17	0.34	0.40	0.62	0.76	0.85	0.95	0.98	0.92	0.98
41	0.16	0.22	0.47	0.58	0.60	0.88	0.90	0.90	0.94	0.99
42	0.20	0.25	0.48	0.55	0.72	0.83	0.84	0.92	0.94	0.98

Table 15: POD according to each scenario and each k2 value, k1 = 2, alpha = 0.05

	k2=1	k2=2	k2=3	k2=4	k2=5	k2=6	k2=7	k2=8	k2=9	k2=10
1	0.25	0.31	0.35	0.51	0.68	0.66	0.84	0.83	0.85	0.91
2	0.32	0.30	0.34	0.47	0.56	0.58	0.70	0.76	0.85	0.80
3	0.25	0.38	0.40	0.45	0.54	0.58	0.61	0.65	0.78	0.72
4	0.16	0.25	0.41	0.54	0.64	0.73	0.78	0.79	0.97	0.95
5	0.21	0.31	0.37	0.54	0.60	0.68	0.71	0.93	0.85	0.90
6	0.29	0.32	0.32	0.41	0.47	0.49	0.56	0.60	0.72	0.80
7	0.13	0.13	0.12	0.24	0.17	0.39	0.27	0.27	0.42	0.38
8	0.09	0.12	0.18	0.20	0.16	0.26	0.32	0.28	0.30	0.39
9	0.19	0.14	0.18	0.13	0.27	0.22	0.28	0.41	0.36	0.33
10	0.21	0.31	0.45	0.59	0.57	0.82	0.83	0.89	0.90	0.96
11	0.26	0.25	0.42	0.58	0.67	0.76	0.75	0.92	0.92	0.92
12	0.21	0.36	0.38	0.43	0.68	0.72	0.78	0.86	0.91	0.91
13	0.22	0.24	0.37	0.45	0.63	0.74	0.82	0.86	0.92	0.96
14	0.19	0.20	0.38	0.49	0.56	0.69	0.81	0.89	0.93	0.87
15	0.35	0.37	0.53	0.61	0.61	0.68	0.75	0.79	0.89	0.85
16	0.18	0.29	0.50	0.59	0.67	0.81	0.89	0.95	0.99	0.97
17	0.23	0.44	0.42	0.59	0.62	0.67	0.78	0.86	0.87	0.94
18	0.38	0.40	0.49	0.50	0.58	0.74	0.70	0.77	0.71	0.86
19	0.26	0.41	0.55	0.59	0.74	0.71	0.90	0.87	0.86	0.98
20	0.24	0.39	0.45	0.56	0.63	0.69	0.85	0.84	0.94	0.93
21	0.25	0.38	0.55	0.55	0.64	0.72	0.83	0.83	0.84	0.88
22	0.25	0.36	0.46	0.64	0.71	0.84	0.87	0.88	0.96	0.95
23	0.21	0.37	0.47	0.53	0.66	0.67	0.81	0.91	0.93	0.94
24	0.28	0.36	0.41	0.56	0.68	0.73	0.78	0.79	0.81	0.89
25	0.19	0.32	0.40	0.56	0.75	0.80	0.87	0.90	0.95	0.98
26	0.34	0.33	0.44	0.48	0.53	0.64	0.64	0.78	0.85	0.89
27	0.34	0.38	0.41	0.45	0.47	0.60	0.52	0.62	0.68	0.72
28	0.21	0.36	0.44	0.60	0.76	0.87	0.92	0.97	0.95	0.98
29	0.27	0.29	0.48	0.47	0.51	0.60	0.64	0.67	0.80	0.73
30	0.39	0.42	0.47	0.44	0.49	0.54	0.59	0.50	0.55	0.67
31	0.16	0.34	0.49	0.60	0.73	0.91	0.85	0.95	0.97	0.99
32	0.14	0.26	0.40	0.62	0.71	0.88	0.91	0.92	0.92	0.98
33	0.23	0.35	0.56	0.58	0.66	0.74	0.77	0.85	0.89	0.95
34	0.17	0.26	0.43	0.53	0.71	0.91	0.89	0.93	0.94	0.99
35	0.26	0.39	0.50	0.59	0.69	0.81	0.92	0.92	0.96	0.95
36	0.37	0.38	0.52	0.59	0.63	0.71	0.82	0.85	0.89	0.93
37	0.25	0.20	0.51	0.55	0.75	0.87	0.90	0.94	0.97	0.99
38	0.25	0.28	0.39	0.53	0.71	0.86	0.92	0.91	1.00	0.95
39	0.19	0.31	0.38	0.63	0.61	0.84	0.85	0.92	0.98	0.96
40	0.24	0.32	0.45	0.58	0.72	0.84	0.94	0.95	0.96	0.99
41	0.20	0.29	0.36	0.58	0.71	0.81	0.94	0.95	0.99	0.98
42	0.12	0.35	0.40	0.44	0.74	0.80	0.87	0.88	0.98	0.99

Table 16: POD according to each scenario and each k2 value, k1 = 3, alpha = 0.05

	k2=1	k2=2	k2=3	k2=4	k2=5	k2=6	k2=7	k2=8	k2=9	k2=10
1	0.18	0.36	0.44	0.51	0.67	0.67	0.81	0.86	0.89	0.92
2	0.29	0.27	0.42	0.46	0.59	0.60	0.69	0.76	0.81	0.84
3	0.23	0.34	0.39	0.45	0.54	0.53	0.66	0.68	0.64	0.79
4	0.23	0.26	0.32	0.60	0.59	0.71	0.80	0.90	0.94	0.97
5	0.31	0.31	0.39	0.46	0.53	0.70	0.67	0.80	0.87	0.87
6	0.28	0.28	0.40	0.41	0.42	0.49	0.65	0.57	0.68	0.64
7	0.10	0.11	0.11	0.14	0.15	0.26	0.21	0.29	0.31	0.39
8	0.13	0.19	0.15	0.19	0.26	0.19	0.25	0.25	0.34	0.32
9	0.19	0.12	0.12	0.18	0.33	0.26	0.28	0.37	0.39	0.46
10	0.20	0.32	0.43	0.58	0.71	0.81	0.89	0.89	0.93	0.97
11	0.27	0.32	0.45	0.46	0.66	0.76	0.76	0.89	0.87	0.92
12	0.23	0.35	0.45	0.53	0.59	0.75	0.75	0.89	0.90	0.89
13	0.18	0.25	0.48	0.50	0.68	0.74	0.83	0.91	0.90	0.97
14	0.22	0.26	0.46	0.55	0.69	0.69	0.76	0.88	0.90	0.97
15	0.26	0.39	0.50	0.49	0.63	0.67	0.66	0.81	0.82	0.89
16	0.22	0.26	0.46	0.60	0.73	0.80	0.87	0.94	0.98	0.97
17	0.30	0.38	0.64	0.61	0.66	0.78	0.79	0.74	0.84	0.85
18	0.45	0.50	0.44	0.48	0.59	0.62	0.73	0.75	0.77	0.84
19	0.31	0.44	0.51	0.59	0.68	0.80	0.87	0.92	0.92	0.92
20	0.28	0.44	0.44	0.58	0.73	0.74	0.86	0.78	0.96	0.93
21	0.28	0.39	0.38	0.55	0.61	0.71	0.78	0.82	0.84	0.94
22	0.28	0.42	0.54	0.62	0.73	0.82	0.91	0.91	0.97	0.97
23	0.24	0.34	0.47	0.62	0.71	0.78	0.81	0.89	0.94	0.94
24	0.36	0.44	0.43	0.49	0.62	0.69	0.71	0.74	0.86	0.94
25	0.26	0.38	0.42	0.63	0.68	0.80	0.84	0.96	0.94	1.00
26	0.23	0.38	0.50	0.53	0.70	0.64	0.68	0.78	0.81	0.91
27	0.33	0.34	0.44	0.49	0.53	0.53	0.66	0.73	0.63	0.66
28	0.19	0.33	0.44	0.65	0.68	0.77	0.89	0.93	0.95	0.98
29	0.41	0.34	0.40	0.42	0.54	0.66	0.69	0.77	0.87	0.81
30	0.35	0.35	0.49	0.36	0.45	0.49	0.49	0.61	0.62	0.64
31	0.23	0.32	0.44	0.45	0.67	0.82	0.89	0.94	0.95	1.00
32	0.18	0.31	0.42	0.57	0.74	0.74	0.88	0.89	0.96	0.97
33	0.30	0.37	0.47	0.58	0.67	0.73	0.83	0.85	0.86	0.95
34	0.12	0.27	0.45	0.58	0.79	0.81	0.93	0.97	0.97	0.98
35	0.22	0.35	0.48	0.55	0.73	0.84	0.89	0.92	0.94	0.98
36	0.28	0.37	0.62	0.57	0.67	0.77	0.76	0.85	0.86	0.87
37	0.16	0.29	0.41	0.56	0.70	0.87	0.92	0.93	0.95	0.99
38	0.15	0.34	0.38	0.52	0.81	0.86	0.92	0.91	0.99	0.96
39	0.22	0.20	0.46	0.49	0.61	0.84	0.85	0.94	0.95	0.96
40	0.20	0.25	0.44	0.64	0.71	0.81	0.94	0.91	0.96	0.94
41	0.23	0.32	0.56	0.60	0.69	0.82	0.89	0.95	0.95	0.98
42	0.21	0.33	0.37	0.59	0.69	0.81	0.90	0.94	0.89	0.94

Table 17: POD according to each scenario and each k2 value, k1 = 5, alpha = 0.05

	k2=1	k2=2	k2=3	k2=4	k2=5	k2=6	k2=7	k2=8	k2=9	k2=10
1	0.26	0.32	0.38	0.58	0.66	0.71	0.87	0.76	0.83	0.91
2	0.23	0.32	0.35	0.49	0.58	0.63	0.72	0.79	0.80	0.85
3	0.27	0.28	0.40	0.45	0.53	0.56	0.69	0.69	0.73	0.73
4	0.19	0.27	0.41	0.51	0.66	0.73	0.85	0.90	0.92	0.95
5	0.21	0.34	0.27	0.41	0.60	0.74	0.76	0.79	0.84	0.91
6	0.21	0.28	0.40	0.43	0.46	0.62	0.64	0.57	0.75	0.75
7	0.11	0.14	0.19	0.23	0.19	0.21	0.29	0.27	0.30	0.36
8	0.10	0.10	0.13	0.17	0.25	0.19	0.34	0.28	0.31	0.32
9	0.09	0.12	0.22	0.14	0.26	0.25	0.26	0.28	0.41	0.34
10	0.17	0.31	0.37	0.46	0.65	0.79	0.81	0.89	0.93	0.96
11	0.25	0.30	0.44	0.52	0.66	0.65	0.79	0.82	0.90	0.96
12	0.12	0.32	0.44	0.49	0.64	0.73	0.79	0.86	0.91	0.94
13	0.21	0.25	0.37	0.42	0.66	0.76	0.76	0.90	0.89	0.91
14	0.18	0.24	0.33	0.51	0.75	0.70	0.81	0.81	0.93	0.94
15	0.34	0.34	0.42	0.47	0.67	0.74	0.78	0.85	0.82	0.88
16	0.16	0.29	0.45	0.54	0.71	0.84	0.91	0.97	0.97	0.98
17	0.33	0.40	0.54	0.51	0.70	0.77	0.77	0.82	0.81	0.93
18	0.45	0.41	0.43	0.55	0.62	0.65	0.78	0.71	0.76	0.81
19	0.33	0.44	0.55	0.57	0.75	0.84	0.78	0.89	0.90	0.86
20	0.26	0.34	0.52	0.55	0.69	0.72	0.77	0.81	0.89	0.90
21	0.31	0.38	0.47	0.49	0.67	0.72	0.81	0.81	0.82	0.87
22	0.18	0.33	0.47	0.61	0.74	0.80	0.88	0.93	0.96	0.98
23	0.23	0.30	0.50	0.59	0.64	0.73	0.82	0.89	0.91	0.94
24	0.29	0.25	0.34	0.51	0.58	0.63	0.72	0.73	0.86	0.87
25	0.16	0.32	0.45	0.60	0.70	0.74	0.86	0.90	0.91	0.95
26	0.29	0.34	0.42	0.45	0.57	0.53	0.80	0.83	0.76	0.81
27	0.25	0.33	0.37	0.51	0.47	0.54	0.60	0.64	0.69	0.70
28	0.16	0.28	0.45	0.55	0.81	0.83	0.86	0.92	0.98	0.98
29	0.37	0.35	0.37	0.56	0.61	0.59	0.61	0.83	0.79	0.76
30	0.28	0.43	0.47	0.40	0.54	0.50	0.61	0.61	0.59	0.62
31	0.16	0.24	0.48	0.63	0.76	0.84	0.88	0.95	0.98	1.00
32	0.23	0.31	0.38	0.59	0.63	0.77	0.87	0.89	0.93	0.97
33	0.25	0.35	0.44	0.65	0.61	0.74	0.80	0.86	0.84	0.97
34	0.14	0.28	0.54	0.59	0.75	0.77	0.91	0.92	0.99	0.97
35	0.25	0.41	0.45	0.60	0.73	0.80	0.87	0.93	0.95	0.96
36	0.27	0.39	0.45	0.49	0.66	0.69	0.81	0.84	0.87	0.91
37	0.15	0.31	0.45	0.62	0.77	0.85	0.91	0.92	0.94	1.00
38	0.18	0.29	0.42	0.53	0.62	0.85	0.92	0.94	0.98	0.93
39	0.11	0.31	0.36	0.56	0.71	0.73	0.87	0.92	0.95	0.95
40	0.12	0.26	0.40	0.63	0.73	0.79	0.89	0.90	0.98	0.97
41	0.20	0.30	0.39	0.54	0.78	0.79	0.86	0.97	0.95	1.00
42	0.15	0.30	0.37	0.62	0.70	0.75	0.86	0.89	0.90	0.97

Table 18: POD according to each scenario and each k2 value, k1 = 10, alpha = 0.05
