# Supplementary material for: Evaluation and comparison of statistical methods for early temporal detection of outbreaks: A simulation-based study
Source: PLoS One. 2017 Jul 17;12(7):e0181227. doi: 10.1371/journal.pone.0181227 (PMC5513450; doi:10.1371/journal.pone.0181227)
Supplement: S22 Appendix — (PDF) [file pone.0181227.s022.pdf]

Evaluation and Comparison of Statistical Methods for Early Temporal Detection of Outbreaks: a Simulation-Based Study

Appendix S22: Overall performances of OutbreakP algorithm

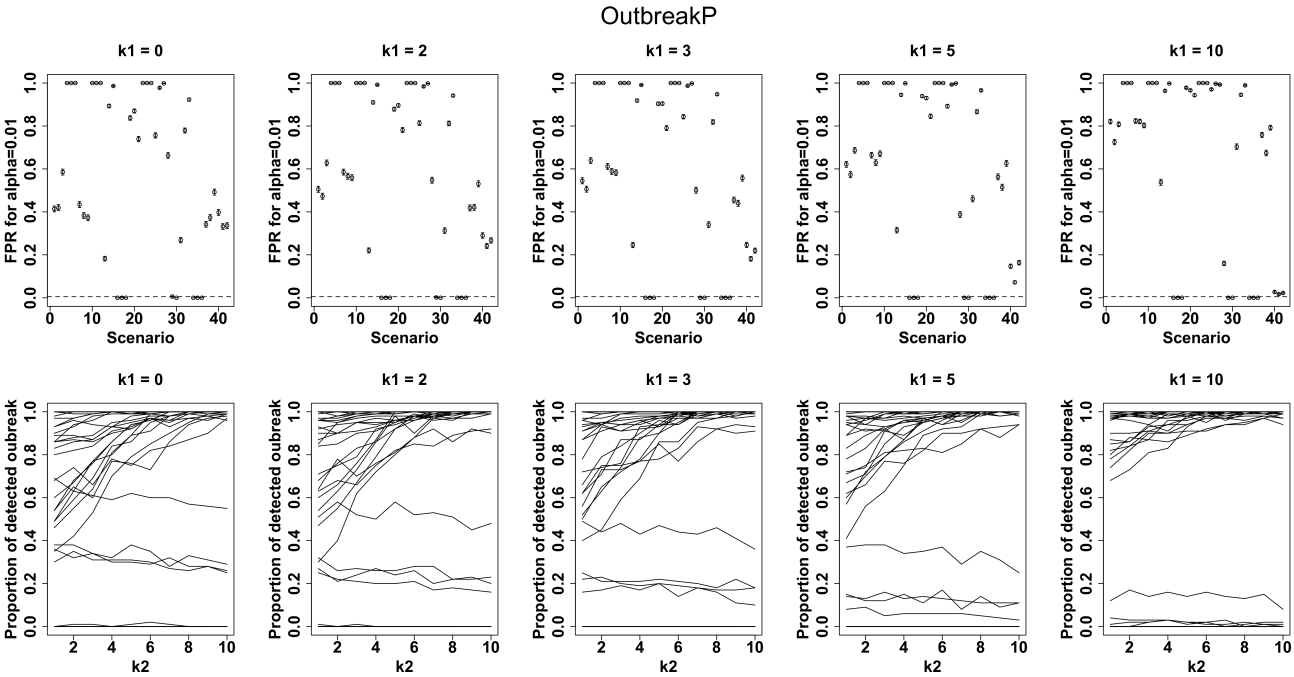

Figure 1: OutbreakP algorithm performances by increasing past outbreak amplitude $k_1 = 0$, 2, 3, 5 or 10 with (i) on the first row: false positive rate for 42 simulated scenarios, (ii) on the second row: probability of detection for 42 simulated scenarios (each curve corresponding to a scenario) by increasing current outbreak amplitude $k_2 = 1$ to 10.

Overall performances of OutbreakP algorithm

	FPR k1=0	FPR k1=2	FPR k1=3	FPR k1=5	FPR k1=10
1	0.41	0.51	0.54	0.62	0.82
2	0.42	0.47	0.51	0.57	0.73
3	0.59	0.63	0.64	0.69	0.81
4	1.00	1.00	1.00	1.00	1.00
5	1.00	1.00	1.00	1.00	1.00
6	1.00	1.00	1.00	1.00	1.00
7	0.43	0.59	0.61	0.66	0.82
8	0.38	0.57	0.59	0.63	0.82
9	0.37	0.56	0.58	0.67	0.80
10	1.00	1.00	1.00	1.00	1.00
11	1.00	1.00	1.00	1.00	1.00
12	1.00	1.00	1.00	1.00	1.00
13	0.18	0.22	0.25	0.31	0.54
14	0.89	0.91	0.92	0.94	0.96
15	0.99	0.99	0.99	1.00	1.00
16	0.00	0.00	0.00	0.00	0.00
17	0.00	0.00	0.00	0.00	0.00
18	0.00	0.00	0.00	0.00	0.00
19	0.84	0.88	0.90	0.94	0.98
20	0.87	0.90	0.90	0.93	0.97
21	0.74	0.78	0.79	0.85	0.94
22	1.00	1.00	1.00	1.00	1.00
23	1.00	1.00	1.00	1.00	1.00
24	1.00	1.00	1.00	1.00	1.00
25	0.76	0.81	0.84	0.89	0.97
26	0.98	0.98	0.99	0.99	1.00
27	1.00	1.00	1.00	1.00	0.99
28	0.66	0.55	0.50	0.39	0.16
29	0.01	0.00	0.00	0.00	0.00
30	0.00	0.00	0.00	0.00	0.00
31	0.27	0.31	0.34	0.46	0.70
32	0.78	0.81	0.82	0.87	0.95
33	0.92	0.94	0.95	0.97	0.99
34	0.00	0.00	0.00	0.00	0.00
35	0.00	0.00	0.00	0.00	0.00
36	0.00	0.00	0.00	0.00	0.00
37	0.34	0.42	0.46	0.56	0.76
38	0.37	0.42	0.44	0.51	0.67
39	0.49	0.53	0.56	0.63	0.79
40	0.40	0.29	0.25	0.15	0.03
41	0.33	0.24	0.18	0.07	0.02
42	0.34	0.27	0.22	0.16	0.02

Table 1: FPR according to each scenario and each k1 value

	k2=1	k2=2	k2=3	k2=4	k2=5	k2=6	k2=7	k2=8	k2=9	k2=10
1	0.60	0.67	0.77	0.80	0.89	0.91	0.98	1.00	1.00	1.00
2	0.54	0.65	0.60	0.77	0.76	0.73	0.86	0.94	0.97	0.98
3	0.68	0.74	0.66	0.78	0.75	0.82	0.84	0.87	0.90	0.97
4	1.00	1.00	1.00	1.00	1.00	1.00	1.00	1.00	1.00	1.00
5	1.00	1.00	1.00	1.00	1.00	1.00	1.00	1.00	1.00	1.00
6	1.00	1.00	1.00	1.00	1.00	1.00	1.00	1.00	1.00	1.00
7	0.89	0.89	0.87	0.93	0.92	0.97	0.93	0.97	0.96	0.99
8	0.83	0.86	0.87	0.93	0.95	0.97	0.95	0.99	0.98	0.96
9	0.86	0.87	0.86	0.90	0.92	0.97	0.96	0.97	0.98	0.99
10	1.00	1.00	1.00	1.00	1.00	1.00	1.00	1.00	1.00	1.00
11	1.00	1.00	1.00	1.00	1.00	1.00	1.00	1.00	1.00	1.00
12	1.00	1.00	1.00	1.00	1.00	1.00	1.00	1.00	1.00	1.00
13	0.35	0.42	0.53	0.70	0.79	0.85	0.90	0.95	0.99	0.99
14	0.93	0.96	0.94	0.93	0.98	0.99	0.98	0.99	1.00	1.00
15	1.00	0.99	0.99	1.00	0.99	1.00	1.00	1.00	1.00	1.00
16	0.00	0.00	0.00	0.00	0.00	0.00	0.00	0.00	0.00	0.00
17	0.00	0.00	0.00	0.00	0.00	0.00	0.00	0.00	0.00	0.00
18	0.00	0.00	0.00	0.00	0.00	0.00	0.00	0.00	0.00	0.00
19	0.90	0.93	0.98	0.99	0.99	1.00	1.00	1.00	1.00	1.00
20	0.93	0.97	0.99	1.00	0.99	1.00	1.00	1.00	1.00	1.00
21	0.80	0.82	0.84	0.91	0.93	0.97	0.96	1.00	1.00	1.00
22	1.00	1.00	1.00	1.00	1.00	1.00	1.00	1.00	1.00	1.00
23	1.00	1.00	1.00	1.00	1.00	1.00	1.00	1.00	1.00	1.00
24	1.00	1.00	1.00	1.00	1.00	1.00	1.00	1.00	1.00	1.00
25	0.86	0.94	0.96	1.00	0.99	0.99	1.00	1.00	1.00	1.00
26	0.98	1.00	1.00	1.00	0.99	1.00	1.00	1.00	1.00	1.00
27	1.00	1.00	1.00	1.00	1.00	1.00	1.00	1.00	1.00	1.00
28	0.69	0.63	0.61	0.59	0.62	0.60	0.60	0.57	0.56	0.55
29	0.00	0.01	0.01	0.00	0.01	0.02	0.01	0.00	0.00	0.00
30	0.00	0.00	0.00	0.00	0.00	0.00	0.00	0.00	0.00	
31	0.46	0.55	0.64	0.81	0.89	0.99	1.00	1.00	1.00	1.00
32	0.86	0.88	0.89	0.95	0.96	0.98	0.98	1.00	1.00	1.00
33	0.97	0.97	0.99	1.00	1.00	0.99	1.00	1.00	1.00	1.00
34	0.00	0.00	0.00	0.00	0.00	0.00	0.00	0.00	0.00	0.00
35	0.00	0.00	0.00	0.00	0.00	0.00	0.00	0.00	0.00	0.00
36	0.00	0.00	0.00	0.00	0.00	0.00	0.00	0.00	0.00	0.00
37	0.49	0.63	0.77	0.86	0.94	0.96	1.00	1.00	1.00	1.00
38	0.49	0.59	0.69	0.84	0.86	0.95	0.98	1.00	1.00	1.00
39	0.54	0.68	0.76	0.86	0.94	0.96	1.00	1.00	0.99	1.00
40	0.38	0.38	0.34	0.32	0.38	0.35	0.28	0.33	0.31	0.29
41	0.30	0.35	0.31	0.31	0.31	0.30	0.27	0.26	0.28	0.25
42	0.36	0.32	0.34	0.30	0.30	0.29	0.32	0.28	0.28	0.26

Table 2: POD according to each scenario and each k2 value, k1 = 0

	k2=1	k2=2	k2=3	k2=4	k2=5	k2=6	k2=7	k2=8	k2=9	k2=10
1	0.68	0.76	0.83	0.86	0.98	0.90	0.98	0.99	0.99	0.99
2	0.63	0.68	0.66	0.75	0.82	0.85	0.84	0.89	0.91	0.92
3	0.64	0.78	0.70	0.76	0.81	0.88	0.92	0.86	0.92	0.90
4	1.00	1.00	1.00	1.00	1.00	1.00	1.00	1.00	1.00	1.00
5	1.00	1.00	1.00	1.00	1.00	1.00	1.00	1.00	1.00	1.00
6	1.00	1.00	1.00	1.00	1.00	1.00	1.00	1.00	1.00	1.00
7	0.87	0.90	0.91	0.93	0.96	0.98	0.96	0.97	0.97	0.99
8	0.93	0.90	0.95	0.94	0.96	0.98	0.98	0.97	0.99	1.00
9	0.92	0.94	0.95	0.95	0.95	0.98	0.97	0.99	0.99	0.99
10	1.00	1.00	1.00	1.00	1.00	1.00	1.00	1.00	1.00	1.00
11	1.00	1.00	1.00	1.00	1.00	1.00	1.00	1.00	1.00	1.00
12	1.00	1.00	1.00	1.00	1.00	1.00	1.00	1.00	1.00	1.00
13	0.30	0.40	0.62	0.72	0.80	0.88	0.97	0.96	1.00	1.00
14	0.96	0.95	0.97	0.98	0.98	0.98	1.00	0.99	1.00	1.00
15	0.99	1.00	0.99	0.99	1.00	1.00	1.00	1.00	1.00	1.00
16	0.00	0.00	0.00	0.00	0.00	0.00	0.00	0.00	0.00	0.00
17	0.00	0.00	0.00	0.00	0.00	0.00	0.00	0.00	0.00	0.00
18	0.00	0.00	0.00	0.00	0.00	0.00	0.00	0.00	0.00	0.00
19	0.96	0.98	0.99	1.00	0.99	1.00	1.00	1.00	1.00	1.00
20	0.96	0.96	0.98	0.99	0.99	1.00	1.00	1.00	1.00	1.00
21	0.84	0.85	0.90	0.91	0.95	0.99	0.97	1.00	1.00	1.00
22	1.00	1.00	1.00	1.00	1.00	1.00	1.00	1.00	1.00	1.00
23	1.00	1.00	1.00	1.00	1.00	1.00	1.00	1.00	1.00	1.00
24	1.00	1.00	1.00	1.00	1.00	1.00	1.00	1.00	1.00	1.00
25	0.85	0.95	0.98	0.99	1.00	1.00	1.00	1.00	1.00	1.00
26	1.00	0.98	1.00	1.00	1.00	1.00	1.00	1.00	1.00	1.00
27	1.00	1.00	1.00	1.00	1.00	1.00	1.00	0.99	1.00	1.00
28	0.51	0.58	0.52	0.50	0.58	0.52	0.53	0.51	0.45	0.48
29	0.01	0.00	0.01	0.00	0.00	0.00	0.00	0.00	0.00	0.00
30	0.00	0.00	0.00	0.00	0.00	0.00	0.00	0.00		0.00
31	0.47	0.55	0.67	0.79	0.92	0.98	0.99	1.00	1.00	1.00
32	0.90	0.90	0.95	0.97	0.96	0.96	0.99	0.98	1.00	1.00
33	0.97	0.97	0.99	0.99	0.99	1.00	1.00	1.00	1.00	1.00
34	0.00	0.00	0.00	0.00	0.00	0.00	0.00	0.00	0.00	0.00
35	0.00	0.00	0.00	0.00	0.00	0.00	0.00	0.00	0.00	0.00
36	0.00	0.00	0.00	0.00	0.00	0.00	0.00	0.00	0.00	0.00
37	0.60	0.66	0.76	0.87	1.00	1.00	0.99	1.00	1.00	1.00
38	0.54	0.63	0.73	0.86	0.92	0.96	0.98	1.00	1.00	1.00
39	0.71	0.75	0.79	0.92	0.94	0.97	0.99	1.00	1.00	1.00
40	0.32	0.26	0.27	0.26	0.26	0.28	0.28	0.22	0.22	0.23
41	0.25	0.22	0.21	0.20	0.20	0.21	0.17	0.18	0.17	0.16
42	0.27	0.21	0.24	0.27	0.24	0.26	0.20	0.22	0.23	0.20

Table 3: POD according to each scenario and each k2 value, k1 = 2

	k2=1	k2=2	k2=3	k2=4	k2=5	k2=6	k2=7	k2=8	k2=9	k2=10
1	0.72	0.74	0.76	0.93	0.94	0.94	1.00	1.00	0.98	1.00
2	0.56	0.73	0.73	0.77	0.78	0.83	0.93	0.92	0.94	0.93
3	0.62	0.75	0.75	0.77	0.85	0.77	0.86	0.92	0.90	0.91
4	1.00	1.00	1.00	1.00	1.00	1.00	1.00	1.00	1.00	1.00
5	1.00	1.00	1.00	1.00	1.00	1.00	1.00	1.00	1.00	1.00
6	1.00	1.00	1.00	1.00	1.00	1.00	1.00	1.00	1.00	1.00
7	0.87	0.91	0.94	0.96	0.93	0.99	0.98	0.99	0.97	0.98
8	0.94	0.95	0.94	0.96	0.97	0.98	0.97	0.99	0.99	0.99
9	0.91	0.95	0.94	0.94	0.97	0.97	0.97	0.97	0.98	1.00
10	1.00	1.00	1.00	1.00	1.00	1.00	1.00	1.00	1.00	1.00
11	1.00	1.00	1.00	1.00	1.00	1.00	1.00	1.00	1.00	1.00
12	1.00	1.00	1.00	1.00	1.00	1.00	1.00	1.00	1.00	1.00
13	0.40	0.45	0.59	0.69	0.86	0.86	0.95	0.97	1.00	0.99
14	0.96	0.94	0.97	0.98	0.98	0.98	1.00	1.00	1.00	0.99
15	1.00	0.99	1.00	1.00	1.00	1.00	1.00	1.00	1.00	1.00
16	0.00	0.00	0.00	0.00	0.00	0.00	0.00	0.00	0.00	0.00
17	0.00	0.00	0.00	0.00	0.00	0.00	0.00	0.00	0.00	0.00
18	0.00	0.00	0.00	0.00	0.00	0.00	0.00	0.00	0.00	0.00
19	0.97	0.97	0.99	1.00	1.00	1.00	1.00	1.00	1.00	1.00
20	0.93	0.95	0.96	0.97	0.98	0.99	0.99	1.00	1.00	1.00
21	0.78	0.93	0.91	0.92	0.93	0.97	0.97	0.99	1.00	1.00
22	1.00	1.00	1.00	1.00	1.00	1.00	1.00	1.00	1.00	1.00
23	1.00	1.00	1.00	1.00	1.00	1.00	1.00	1.00	1.00	1.00
24	1.00	1.00	1.00	1.00	1.00	1.00	1.00	1.00	1.00	1.00
25	0.91	0.94	0.99	0.99	0.99	1.00	1.00	1.00	1.00	1.00
26	1.00	1.00	1.00	1.00	1.00	1.00	1.00	1.00	1.00	1.00
27	1.00	1.00	1.00	1.00	1.00	1.00	0.99	1.00	1.00	1.00
28	0.49	0.44	0.48	0.43	0.47	0.44	0.43	0.46	0.41	0.36
29	0.00	0.00	0.00	0.00	0.00	0.00	0.00	0.00	0.00	0.00
30	0.00	0.00	0.00	0.00	0.00	0.00	0.00	0.00	0.00	
31	0.50	0.65	0.72	0.78	0.90	1.00	0.98	1.00	1.00	1.00
32	0.87	0.94	0.91	0.95	0.98	0.99	1.00	1.00	1.00	1.00
33	0.96	0.99	1.00	1.00	1.00	0.99	1.00	1.00	1.00	1.00
34	0.00	0.00	0.00	0.00	0.00	0.00	0.00	0.00	0.00	0.00
35	0.00	0.00	0.00	0.00	0.00	0.00	0.00	0.00	0.00	0.00
36	0.00	0.00	0.00	0.00	0.00	0.00	0.00	0.00	0.00	0.00
37	0.62	0.71	0.87	0.87	0.94	1.00	0.99	1.00	1.00	1.00
38	0.52	0.63	0.74	0.88	0.90	0.95	1.00	1.00	1.00	1.00
39	0.66	0.79	0.84	0.90	0.96	0.94	0.99	1.00	1.00	1.00
40	0.25	0.21	0.21	0.21	0.22	0.21	0.20	0.18	0.22	0.18
41	0.16	0.17	0.19	0.17	0.20	0.14	0.18	0.16	0.11	0.10
42	0.22	0.23	0.20	0.19	0.20	0.19	0.18	0.17	0.17	0.18

Table 4: POD according to each scenario and each k2 value, k1 = 3

	k2=1	k2=2	k2=3	k2=4	k2=5	k2=6	k2=7	k2=8	k2=9	k2=10
1	0.78	0.83	0.92	0.87	0.97	0.94	0.96	1.00	1.00	1.00
2	0.62	0.66	0.77	0.76	0.82	0.90	0.90	0.92	0.93	0.94
3	0.71	0.75	0.81	0.82	0.83	0.81	0.85	0.92	0.88	0.94
4	1.00	1.00	1.00	1.00	1.00	1.00	1.00	1.00	1.00	1.00
5	1.00	1.00	1.00	1.00	1.00	1.00	1.00	1.00	1.00	1.00
6	1.00	1.00	1.00	1.00	1.00	1.00	1.00	1.00	1.00	1.00
7	0.89	0.92	0.91	0.96	0.95	0.97	0.98	0.98	1.00	1.00
8	0.94	0.91	0.92	0.96	0.97	0.98	0.95	1.00	1.00	0.98
9	0.98	0.94	0.93	0.95	0.96	0.98	0.98	1.00	0.98	0.99
10	1.00	1.00	1.00	1.00	1.00	1.00	1.00	1.00	1.00	1.00
11	1.00	1.00	1.00	1.00	1.00	1.00	1.00	1.00	1.00	1.00
12	1.00	1.00	1.00	1.00	1.00	1.00	1.00	1.00	1.00	1.00
13	0.41	0.56	0.63	0.75	0.89	0.92	0.93	0.99	0.98	0.99
14	0.96	0.97	0.97	0.98	0.97	0.98	1.00	0.99	1.00	1.00
15	0.99	1.00	1.00	1.00	1.00	1.00	1.00	1.00	1.00	1.00
16	0.00	0.00	0.00	0.00	0.00	0.00	0.00	0.00	0.00	0.00
17	0.00	0.00	0.00	0.00	0.00	0.00	0.00	0.00	0.00	0.00
18	0.00	0.00	0.00	0.00	0.00	0.00	0.00	0.00	0.00	0.00
19	0.98	0.97	1.00	0.99	1.00	1.00	1.00	1.00	1.00	1.00
20	0.95	0.94	0.99	0.99	0.99	1.00	1.00	1.00	1.00	1.00
21	0.83	0.89	0.93	0.95	0.97	0.94	0.99	1.00	1.00	1.00
22	1.00	1.00	1.00	1.00	1.00	1.00	1.00	1.00	1.00	1.00
23	1.00	1.00	1.00	1.00	1.00	1.00	1.00	1.00	1.00	1.00
24	1.00	1.00	1.00	1.00	1.00	1.00	1.00	1.00	1.00	1.00
25	0.94	0.96	0.99	1.00	0.99	1.00	1.00	1.00	1.00	1.00
26	1.00	1.00	1.00	1.00	1.00	1.00	1.00	1.00	1.00	1.00
27	1.00	1.00	1.00	1.00	1.00	1.00	1.00	1.00	1.00	1.00
28	0.37	0.38	0.38	0.34	0.35	0.37	0.29	0.35	0.31	0.25
29	0.00	0.00	0.00	0.00	0.00	0.00	0.00	0.00	0.00	0.00
30	0.00	0.00	0.00	0.00	0.00	0.00	0.00	0.00		
31	0.57	0.72	0.79	0.86	0.91	0.96	1.00	0.99	1.00	1.00
32	0.89	0.97	0.96	0.97	0.98	0.99	0.99	1.00	1.00	0.99
33	0.98	0.98	0.97	1.00	1.00	1.00	1.00	1.00	1.00	1.00
34	0.00	0.00	0.00	0.00	0.00	0.00	0.00	0.00	0.00	0.00
35	0.00	0.00	0.00	0.00	0.00	0.00	0.00	0.00	0.00	0.00
36	0.00	0.00	0.00	0.00	0.00	0.00	0.00	0.00	0.00	0.00
37	0.67	0.71	0.85	0.93	0.95	1.00	0.99	1.00	1.00	1.00
38	0.60	0.70	0.79	0.87	0.96	0.98	1.00	0.99	1.00	1.00
39	0.72	0.74	0.91	0.89	0.95	0.97	1.00	1.00	1.00	1.00
40	0.15	0.12	0.12	0.15	0.11	0.17	0.08	0.14	0.09	0.11
41	0.08	0.09	0.05	0.06	0.06	0.06	0.06	0.05	0.04	0.03
42	0.14	0.13	0.16	0.13	0.14	0.13	0.12	0.11	0.11	0.11

Table 5: POD according to each scenario and each k2 value, k1 = 5

	k2=1	k2=2	k2=3	k2=4	k2=5	k2=6	k2=7	k2=8	k2=9	k2=10
1	0.90	0.90	0.91	0.94	0.97	1.00	1.00	1.00	0.99	1.00
2	0.78	0.84	0.87	0.86	0.89	0.92	0.94	0.94	0.97	0.99
3	0.82	0.84	0.91	0.90	0.91	0.91	0.94	0.95	0.97	0.94
4	1.00	1.00	1.00	1.00	1.00	1.00	1.00	1.00	1.00	1.00
5	1.00	1.00	1.00	1.00	1.00	1.00	1.00	1.00	1.00	1.00
6	1.00	1.00	1.00	1.00	1.00	1.00	1.00	1.00	1.00	1.00
7	0.98	0.98	0.97	0.97	0.98	0.95	1.00	0.99	1.00	1.00
8	0.97	0.99	0.98	0.97	0.99	0.98	1.00	0.99	1.00	1.00
9	0.99	0.99	0.99	0.99	0.99	1.00	0.96	0.99	1.00	0.99
10	1.00	1.00	1.00	1.00	1.00	1.00	1.00	1.00	1.00	1.00
11	1.00	1.00	1.00	1.00	1.00	1.00	1.00	1.00	1.00	1.00
12	1.00	1.00	1.00	1.00	1.00	1.00	1.00	1.00	1.00	1.00
13	0.68	0.73	0.81	0.83	0.93	0.98	0.98	0.99	0.99	1.00
14	0.96	0.99	0.97	1.00	1.00	1.00	0.98	1.00	1.00	1.00
15	1.00	1.00	1.00	1.00	1.00	1.00	1.00	1.00	1.00	1.00
16	0.00	0.00	0.00	0.00	0.00	0.00	0.00	0.00	0.00	0.00
17	0.00	0.00	0.00	0.00	0.00	0.00	0.00	0.00	0.00	0.00
18	0.00	0.00	0.00	0.00	0.00	0.00	0.00	0.00	0.00	0.00
19	0.99	1.00	1.00	1.00	1.00	1.00	1.00	1.00	1.00	1.00
20	0.99	0.99	1.00	1.00	1.00	1.00	1.00	1.00	1.00	1.00
21	0.97	0.96	0.94	0.99	0.98	1.00	0.99	1.00	1.00	1.00
22	1.00	1.00	1.00	1.00	1.00	1.00	1.00	1.00	1.00	1.00
23	1.00	1.00	1.00	1.00	1.00	1.00	1.00	1.00	1.00	1.00
24	1.00	1.00	1.00	1.00	1.00	1.00	1.00	1.00	1.00	1.00
25	0.99	0.99	1.00	1.00	1.00	1.00	1.00	1.00	1.00	1.00
26	1.00	1.00	1.00	1.00	1.00	1.00	1.00	1.00	1.00	1.00
27	0.98	0.99	1.00	0.98	1.00	0.99	0.99	1.00	0.99	0.99
28	0.12	0.17	0.14	0.16	0.14	0.16	0.14	0.13	0.15	0.08
29	0.00	0.00	0.00	0.00	0.00	0.00	0.00	0.00	0.00	0.00
30	0.00	0.00	0.00	0.00	0.00	0.00	0.00	0.00	0.00	0.00
31	0.85	0.86	0.94	0.91	0.98	0.99	1.00	0.99	1.00	1.00
32	0.97	0.98	0.98	1.00	1.00	1.00	1.00	1.00	1.00	1.00
33	1.00	1.00	0.99	1.00	1.00	1.00	1.00	1.00	1.00	1.00
34	0.00	0.00	0.00	0.00	0.00	0.00	0.00	0.00	0.00	0.00
35	0.00	0.00	0.00	0.00	0.00	0.00	0.00	0.00	0.00	0.00
36	0.00	0.00	0.00	0.00	0.00	0.00	0.00	0.00	0.00	0.00
37	0.80	0.88	0.87	0.96	0.96	0.98	0.97	1.00	0.99	0.97
38	0.74	0.82	0.90	0.90	0.94	0.97	0.97	0.96	0.98	0.97
39	0.87	0.86	0.93	0.94	0.94	0.97	0.97	0.98	0.98	0.97
40	0.01	0.02	0.02	0.03	0.01	0.02	0.03	0.00	0.01	0.00
41	0.00	0.00	0.02	0.03	0.02	0.01	0.01	0.02	0.01	0.01
42	0.04	0.03	0.03	0.03	0.02	0.02	0.00	0.01	0.02	0.02

Table 6: POD according to each scenario and each k2 value, k1 = 10
