# Supplementary material for: Evaluation and comparison of statistical methods for early temporal detection of outbreaks: A simulation-based study
Source: PLoS One. 2017 Jul 17;12(7):e0181227. doi: 10.1371/journal.pone.0181227 (PMC5513450; doi:10.1371/journal.pone.0181227)
Supplement: S23 Appendix — (PDF) [file pone.0181227.s023.pdf]

Evaluation and Comparison of Statistical Methods for Early Temporal Detection of Outbreaks: a Simulation-Based Study

Appendix S23: Radar charts of performances indicators : POD1week, POD, PPV, NPV, 1-FPR, Sp and Se for all 21 methods ($\alpha = 0.01$ for Improved Farrington, Original Farrington, Periodic Poisson GLM and Neg Binomial GLM, CDC and EARS C1-C3. $\alpha = 0.05$ for Bayes 1-3)

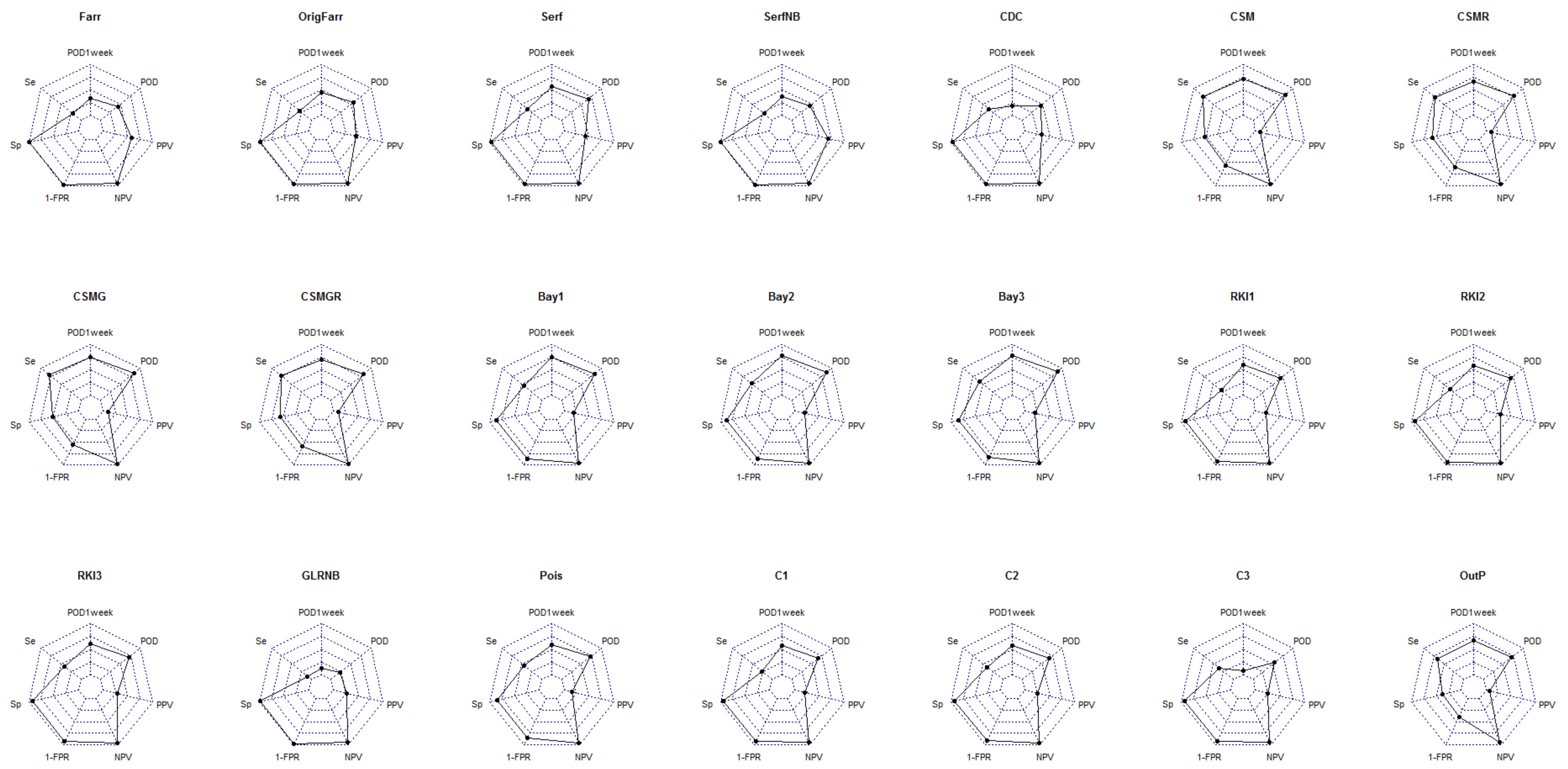

Figure 1: **Radar charts of performances indicators: POD1week, POD, PPV, NPV, 1-FPR, Sp and Se for all 21 methods ($\alpha = 0.01$ for Improved Farrington, Original Farrington, Periodic Poisson GLM and Neg Binomial GLM, CDC and EARS C1-C3. $\alpha = 0.05$ for Bayes 1-3). Radarplot center = 0,maximum = 1.** (Farr = Improved Farrington, OrigFarr = Original Farrington, Serf = Periodic Poisson GLM, SerfNB = Periodic Negative Binomial GLM, CDC = CDC algorithm, CUSUM = CUSUM, CUSUMR = CUSUM Rossi, CUSUMG = CUSUM GLM, CSMGR = CUSUM GLM Rossi, Bay1 = Bayes 1, Bay2 = Bayes 2, Bay3 = Bayes 3, RKI1 = RKI 1, RKI2 = RKI 2, RKI3 = RKI 3, Pois = GLR Poisson, GLRNB = GLR Negative Binomial, C1 = EARS C1, C2 = EARS C2, C3 = EARS C3, OutP = Outbreak P).
