# Supplementary material for: Evaluation and comparison of statistical methods for early temporal detection of outbreaks: A simulation-based study
Source: PLoS One. 2017 Jul 17;12(7):e0181227. doi: 10.1371/journal.pone.0181227 (PMC5513450; doi:10.1371/journal.pone.0181227)
Supplement: S24 Appendix — (PDF) [file pone.0181227.s024.pdf]

Evaluation and Comparison of Statistical Methods for Early Temporal Detection of Outbreaks: a Simulation-Based Study

Appendix S24: R code of periodic Poisson GLM algorithm and periodic negative binomial GLM algorithm.

R code of periodic Poisson GLM algorithm

```
AlarmSerfling <- function(disProgObj=NULL){
  observed  <- c(disProgObj$observed)
  p <- disProgObj$freq
  alarm <- NA
  for(i in 1:50){
    df <- data.frame(x=observed[1:(573+i)],t=1:(573+i))
    model <- glm(x ~ 1+t + sin(2 * pi* t/p ) + cos(2 * pi* t/p ) +
                 sin(4 * pi/p * t) + cos(4 * pi/p * t), family=poisson(),data=df)
    predict <- predict.glm(model,newdata=data.frame(x=observed[1:(574+i)],t=1:(574+i)),
                           se.fit=T,type="response")
    yhat <- predict$fit
    alarm[i] <- ifelse(observed[574+i]>qpois(1-alpha,yhat[574+i]),1,0)
  }
  return(alarm)
}
```

R code of periodic negative binomial GLM algorithm

```
AlarmSerflingNB <- function(disProgObj=NULL){
  observed  <- c(disProgObj$observed)
  p <- disProgObj$freq
  alarm <- NA
  for(i in 1:50){
    df <- data.frame(x=observed[1:(573+i)],t=1:(573+i))
    model <- glm.nb(x ~ 1+t + sin(2 * pi* t/p ) + cos(2 * pi* t/p ) +
                        sin(4 * pi/p * t) + cos(4 * pi/p * t),data=df)
    predict <- predict.glm(model,newdata=data.frame(x=observed[1:(574+i)],t=1:(574+i)),
                           se.fit=T,type="response")
    yhat <- predict$fit
    dispersion <- model$theta
    alarm[i] <- ifelse(observed[574+i]>
                  qnbinom(1-alpha,size = dispersion,mu=yhat[574+i]),1,0)
  }
  return(alarm)
}
```
