# Supplementary material for: Evaluation and comparison of statistical methods for early temporal detection of outbreaks: A simulation-based study
Source: PLoS One. 2017 Jul 17;12(7):e0181227. doi: 10.1371/journal.pone.0181227 (PMC5513450; doi:10.1371/journal.pone.0181227)
Supplement: S1 Table — (PDF) [file pone.0181227.s025.pdf]

# Evaluation and Comparison of Statistical Methods for Early Temporal Detection of Outbreaks: a Simulation-Based Study

## Table S1: FPR, specificity, POD, POD1week, sensitivity, negative predictive value, positive predictive value and $F_1$-measure for 12 evaluated methods and $\alpha = 0.001$ (for past outbreak constant $k_1 = 0, 2, 3, 5, 10$ and current outbreak $k_2 = 1$ to $10$ for POD and sensitivity).

| Method | FPR | Specificity | POD | POD1week | Sensitivity | NPV⋆ | PPV⋆⋆ | $F_1$-measure |
|---|---|---|---|---|---|---|---|---|
| Improved Farrington | 0.2% | 99.8% | 25.7% | 19.7% | 11.0% | 94.4% | 75.8% | 0.19 |
| Original Farrington | 0.9% | 99.1% | 41.1% | 31.8% | 18.9% | 94.9% | 59.1% | 0.29 |
| Periodic Poisson GLM | 1.5% | 98.6% | 52.1% | 42.6% | 25.1% | 95.2% | 53.7% | 0.34 |
| Periodic Neg Binomial GLM | 0.1% | 99.9% | 24.2% | 19.5% | 10.1% | 94.4% | 88.7% | 0.18 |
| CDC | 1.3% | 98.1% | 32.2% | 9.8% | 22.6% | 95.1% | 44.4% | 0.30 |
| CUSUM | 44.0% | 52.7% | 80.5% | 70.5% | 75.4% | 97.0% | 9.5% | 0.17 |
| CUSUM Rossi | 39.5% | 57.6% | 77.0% | 65.9% | 71.8% | 96.9% | 10.1% | 0.18 |
| CUSUM GLM | 44.2% | 52.0% | 84.4% | 73.8% | 79.5% | 97.5% | 9.9% | 0.18 |
| CUSUM GLM Rossi | 39.9% | 56.8% | 81.1% | 69.5% | 76.1% | 97.3% | 10.4% | 0.18 |
| EARS C1 | 4.0% | 96.4% | 52.9% | 46.5% | 18.7% | 94.7% | 25.4% | 0.22 |
| EARS C2 | 5.3% | 95.3% | 57.0% | 46.8% | 30.6% | 95.4% | 29.9% | 0.30 |
| EARS C3 | 5.2% | 95.0% | 47.4% | 6.0% | 29.2% | 95.3% | 27.8% | 0.28 |

Table 1: FPR, specificity, POD, POD1week, sensitivity, negative predictive value⋆, positive predictive value⋆⋆ and $F_1$-measure for 12 evaluated methods and $\alpha = 0.001$ (for past outbreak constant $k_1 = 0, 2, 3, 5, 10$ and current outbreak $k_2 = 1$ to 10 for POD and sensitivity).
