# Supplementary material for: Evaluation and comparison of statistical methods for early temporal detection of outbreaks: A simulation-based study
Source: PLoS One. 2017 Jul 17;12(7):e0181227. doi: 10.1371/journal.pone.0181227 (PMC5513450; doi:10.1371/journal.pone.0181227)
Supplement: S2 Table — (PDF) [file pone.0181227.s026.pdf]

Evaluation and Comparison of Statistical Methods for Early Temporal Detection of Outbreaks: a Simulation-Based Study

Table S2: FPR, specificity, POD, POD1week, sensitivity, negative predictive value, positive predictive value and F_1-measure for 15 evaluated methods and $\alpha = 0.05$ (for past outbreak constant $k_1 = 0, 2, 3, 5, 10$ and current outbreak $k_2 = 1$ to 10 for POD and sensitivity).

Method	FPR	Specificity	POD	POD1week	Sensitivity	NPV*	PPV**	F_1-measure
Improved Farrington	3.1%	96.9%	60.4%	48.9%	31.8%	95.6%	40.3%	0.36
Original Farrington	5.6%	94.4%	70.4%	57.8%	39.7%	96.0%	31.9%	0.35
Periodic Poisson GLM	7.1%	93.0%	81.0%	69.0%	46.5%	96.3%	30.6%	0.37
Periodic Neg Binomial GLM	3.3%	96.9%	69.1%	57.3%	36.0%	95.8%	43.4 %	0.39
CDC	8.4%	90.4%	57.6%	30.3%	46.7%	96.3%	24.4%	0.32
CUSUM	44.0%	52.7%	80.5%	70.5%	75.4%	97.0%	9.5%	0.17
CUSUM Rossi	39.5%	57.6%	77.0%	65.9%	71.8%	96.9%	10.1%	0.18
CUSUM GLM	44.2%	52.0%	84.4%	73.8%	79.5%	97.5%	9.9%	0.18
CUSUM GLM Rossi	39.9%	56.8%	81.1%	69.5%	76.1%	97.3%	10.4%	0.18
Bayes 1	10.1%	90.5%	76.2%	66.2%	39.1%	95.7%	21.4%	0.28
Bayes 2	9.4%	91.0%	80.8%	69.4%	45.7%	96.2%	25.0%	0.32
Bayes 3	11.1%	88.9%	83.4%	71.9%	51.8%	96.5%	23.6%	0.32
EARS C1	12.0%	88.9%	78.7%	68.5%	35.5%	95.4%	17.5%	0.23
EARS C2	13.7%	87.6%	78.3%	67.7%	48.4%	96.3%	20.5%	0.29
EARS C3	10.8%	89.8%	60.9%	12.2%	41.8%	95.9%	21.3%	0.28

Table 1: FPR, specificity, POD, POD1week, sensitivity, negative predictive value*, positive predictive value** and F_1-measure for 15 evaluated methods and $\alpha = 0.05$ (for past outbreak constant $k_1 = 0, 2, 3, 5, 10$ and current outbreak $k_2 = 1$ to 10 for POD and sensitivity).
