# Supplementary material for: Evaluation and comparison of statistical methods for early temporal detection of outbreaks: A simulation-based study
Source: PLoS One. 2017 Jul 17;12(7):e0181227. doi: 10.1371/journal.pone.0181227 (PMC5513450; doi:10.1371/journal.pone.0181227)
Supplement: S3 Table — (PDF) [file pone.0181227.s027.pdf]

Evaluation and Comparison of Statistical Methods for Early Temporal Detection of Outbreaks: a Simulation-Based Study

Table S3: Other performance ratios, adjusted on past and current outbreak duration and amplitude, trend, seasonality, dispersion and baseline frequency ($\alpha = 0.01$ for Improved Farrington, Original Farrington, Periodic Poisson GLM and Neg Binomial GLM, CDC and EARS C1-C3. $\alpha = 0.05$ for Bayes 1-3).

**Covariates**	**Categories/values**	**Specificity ratio*** (CI 95%)	**Sensitivity ratio*** (CI 95%)	**Negative predictive value ratio*** (CI 95%)	**Positive predictive value ratio*** (CI 95%)
Methods	Improved Farrington	2.65 (2.63-2.66)	0.95 (0.94-0.95)	1.00 (1.00-1.00)	8.31 (8.27-8.34)
	Original Farrington	2.61 (2.60-2.62)	1.34 (1.33-1.35)	1.01 (1.01-1.01)	7.06 (7.03-7.09)
	Periodic Poisson GLM	2.59 (2.57-2.60)	1.64 (1.64-1.65)	1.01 (1.01-1.01)	6.75 (6.72-6.77)
	Periodic Neg Binomial GLM	2.66 (2.64-2.67)	0.96 (0.95-0.96)	1.00 (1.00-1.00)	9.01 (8.96-9.04)
	CDC	2.55 (2.54-2.56)	1.58 (1.57-1.59)	1.01 (1.01-1.01)	5.56 (5.53-5.59)
	CUSUM	1.41 (1.40-1.42)	3.49 (3.47-3.50)	1.02 (1.02-1.02)	1.85 (1.85-1.86)
	CUSUM Rossi	1.54 (1.53-1.55)	3.32 (3.30-3.34)	1.02 (1.02-1.02)	1.99 (1.98-2.00)
	CUSUM GLM	1.39 (1.38-1.40)	3.68 (3.66-3.69)	1.03 (1.03-1.03)	1.94 (1.94-1.95)
	CUSUM GLM Rossi	1.52 (1.51-1.53)	3.52 (3.50-3.54)	1.03 (1.03-1.03)	2.09 (2.09-2.10)
	Bayes 1	2.42 (2.41-2.43)	1.81 (1.80-1.81)	1.01 (1.01-1.01)	3.61 (3.60-3.62)
	Bayes 2	2.43 (2.42-2.44)	2.11 (2.10-2.12)	1.01 (1.01-1.01)	4.21 (4.20-4.23)
	Bayes 3	2.38 (2.36-2.39)	2.93 (2.38-2.40)	1.02 (1.02-1.02)	4.11 (4.10-4.12)
	RKI 1	2.47 (2.45-2.48)	1.41 (1.40-1.41)	1.00 (1.00-1.00)	3.45 (3.44-3.47)
	RKI 2	2.53 (2.52-2.54)	1.60 (1.59-1.60)	1.01 (1.01-1.01)	5.03 (5.01-5.05)
	RKI 3	2.49 (2.47-2.50)	1.93 (1.92-1.94)	1.01 (1.01-1.01)	4.91 (4.90-4.93)
	GLR Negative Binomial	2.55 (2.54-2.57)	Ref (-)	Ref (-)	4.73 (4.71-4.76)
	GLR Poisson	2.26 (2.25-2.27)	2.12 (2.11-2.13)	1.01 (1.01-1.01)	3.34 (3.33-3.36)
	EARS C1	2.50 (2.49-2.52)	1.18 (1.18-1.19)	1.00 (1.00-1.00)	3.45 (3.44-3.47
	EARS C2	2.47 (2.46-2.48)	1.80 (1.79-1.81)	1.01 (1.01-1.01)	4.16 (4.15-4.18)
	EARS C3	2.48 (2.47-2.50)	1.63 (1.62-1.64)	1.01 (1.01-1.01)	4.15 (4.13-4.17)
	OutbreakP	Ref (-)	3.06 (3.04-3.07)	1.00 (1.00-1.00)	Ref (-)
k_1	0	Ref (-)	Ref (-)	Ref (-)	Ref (-)
	2	1.00 (1.00-1.00)	0.99 (0.99-0.99)	1.00 (1.00-1.00)	1.01 (1.01-1.01)
	3	1.00 (1.00-1.00)	0.98 (0.98-0.98)	1.00 (1.00-1.00)	1.02 (1.02-1.02)
	5	1.01 (1.01-1.01)	0.96 (0.96-0.97)	1.00 (1.00-1.00)	1.02 (1.02-1.02)
	10	1.01 (1.01-1.01)	0.93 (0.93-0.93)	1.00 (1.00-1.00)	1.03 (1.03-1.03)
k_2	1	Ref (-)	Ref (-)	Ref (-)	Ref (-)
	2	1.00 (1.00 - 1.00)	1.23 (1.23 - 1.23)	1.00 (1.00-1.00)	1.33 (1.32-1.33)
	3	1.00 (1.00 - 1.00)	1.47 (1.47 - 1.48)	1.00 (1.00-1.00)	1.63 (1.63-1.64)
	4	1.00 (1.00 - 1.00)	1.73 (1.73 - 1.73)	1.00 (1.00-1.00)	1.91 (1.91-1.92)
	5	1.00 (0.99 - 1.00)	1.99 (1.98 - 1.99)	1.00 (1.00-1.00)	2.17 (2.16-2.17)
	6	0.99 (0.99 - 0.99)	2.23 (2.22 - 2.24)	1.00 (1.00-1.00)	2.38 (2.38-2.39)
	7	0.99 (0.99 - 0.99)	2.44 (2.44 - 2.45)	1.00 (1.00-1.01)	2.56 (2.55-2.57)
	8	0.99 (0.99 - 0.99)	2.62 (2.61 - 2.63)	1.01 (1.01-1.01)	2.70 (2.69-2.71)
	9	0.99 (0.99 - 0.99)	2.75 (2.74 - 2.76)	1.01 (1.01-1.01)	2.79 (2.78-2.80)
	10	0.99 (0.99 - 0.99)	2.82 (2.81 - 2.83)	1.01 (1.01-1.01)	2.84 (2.83-2.85)
Trend	No	Ref (-)	Ref (-)	Ref (-)	Ref (-)
	Yes	0.84 (0.84 - 0.84)	1.20 (1.20 - 1.20)	1.00 (1.00-1.00)	0.61 (0.61-0.61)
Seasonality	No	Ref (-)	Ref (-)	Ref (-)	Ref (-)
	Annual	0.99 (0.99 - 0.99)	0.97 (0.97 - 0.97)	1.00 (1.00-1.00)	0.95 (0.95-0.95)
	Biannual	0.98 (0.98 - 0.98)	0.92 (0.92 - 0.92)	1.00 (1.00-1.00)	0.86 (0.86-0.86)
Dispersion	1	Ref (-)	Ref (-)	Ref (-)	Ref (-)
	1.1	1.00 (1.00 - 1.00)	1.00 (1.00 - 1.00)	1.00 (1.00-1.00)	1.00 (0.99-1.00)
	1.2	1.00 (1.00 - 1.00)	0.99 (0.99 - 0.99)	1.00 (1.00-1.00)	0.99 (0.99-0.99)
	1.5	0.99 (0.99 - 0.99)	0.98 (0.98 - 0.98)	1.00 (1.00-1.00)	0.97 (0.97-0.97)
	2	0.99 (0.99 - 0.99)	0.98 (0.97 - 0.98)	1.00 (1.00-1.00)	0.94 (0.94-0.95)
	3	0.98 (0.98 - 0.98)	0.98 (0.98 - 0.99)	1.00 (1.00-1.00)	0.90 (0.89-0.90)
	5	0.97 (0.97 - 0.97)	1.11 (1.11 - 1.11)	1.00 (1.00-1.00)	0.95 (0.95-0.95)
Frequency	-2 (0,14 cases)	Ref (-)	Ref (-)	Ref (-)	Ref (-)
	0.1 (1.1 cases)	0.99 (0.99 - 0.99)	0.95 (0.94 - 0.95)	1.00 (1.00-1.00)	0.94 (0.94-0.94)
	0.5 (1.65 cases)	0.98 (0.98 - 0.98)	0.94 (0.94 - 0.94)	0.99 (0.99-0.99)	0.92 (0.92-0.92)
	1.5 (4.48 cases)	0.97 (0.97 - 0.98)	0.92 (0.92 - 0.93)	0.99 (0.99-0.99)	0.90 (0.90-0.90)
	2.5 (12.18 cases)	0.98 (0.98 - 0.98)	0.90 (0.89 - 0.90)	0.99 (0.99-0.99)	0.92 (0.92-0.92)
	3.75 (42.52 cases)	1.02 (1.02 - 1.02)	0.84 (0.84 - 0.84)	0.98 (0.98-0.98)	1.10 (1.10-1.10)
	5 (148.41 cases)	1.13 (1.13 - 1.13)	0.77 (0.77 - 0.77)	0.98 (0.98-0.98)	1.71 (1.71-1.71)

Table 1: Other performance ratios, adjusted on past and current outbreak duration and amplitude, trend, seasonality, dispersion and baseline frequency. (baseline frequency of cases between brackets) ($\alpha = 0.01$ for Improved Farrington, Original Farrington, Periodic Poisson GLM and Neg Binomial GLM, CDC and EARS C1-C3. $\alpha = 0.05$ for Bayes 1-3). * Each ratio was statistically significant with $p \leq 10e-3$.
